# Local rainfall is more likely than distant thunderstorms to affect movement behaviour in Northern Kenyan elephants

**DOI:** 10.1371/journal.pone.0307520

**Published:** 2024-12-23

**Authors:** Tom Mulder, Beth Mortimer, Jelle Ferwerda, Fritz Vollrath

**Affiliations:** 1 Department of Biology, University of Oxford, Oxford, United Kingdom; 2 University of Twente, Enschede, Netherlands; 3 Save the Elephants, Nairobi, Kenya; University of Mississippi, UNITED STATES OF AMERICA

## Abstract

Previous research indicates that African savanna elephants change their movements preceding or coincident with local rainfall and it has been suggested that they respond to thunder in remote storms–perhaps reading seismic cues. We therefore aimed to test if elephants in Northern Kenya adhere to distinct daytime movement states between the wet and dry periods, and whether their abrupt movement changes precede local wet periods in response to lightning strikes from a specific compass heading. In our study site, lightning to the North and East often preceded local rainfall and could possibly be used to anticipate local wet periods, but local rainfall appears a more likely trigger of behavioural change. While some abrupt movement changes occurred ahead of local wet periods, they were only particularly frequent shortly following the onset of wet periods. These findings do not concur with reports of Namibian elephants that generally changed their movement behaviour preceding local rainfall, and the additional exploration of individual behaviours in the present study likewise did not provide compelling evidence of a generic reliance on remote thunder cues by Northern-Kenyan elephants. Nonetheless, the GPS tracks of elephants indicated that daytime movement velocities differed between wet and dry periods. Specifically, elephants were generally in a slow-moving state during the day through wet periods, and in a fast-moving state during the day through dry periods. There is a further indication that some elephants compensated for slow daytime speeds by moving faster at night. This shift towards increased nocturnal activity may become more common with climate change and may slightly reduce elephant foraging efficiency. We conclude that climate change makes a strong case for studying elephant behaviours in response to environmental cues during the day and night, especially in dry-land study sites like Northern Kenya.

## Introduction

Elephant conservation relies increasingly on a comprehensive understanding of their behaviour, specifically decisions related to movements in space and time, i.e. their motility.

Known drivers of elephant motility include internal factors like reproductive state, and anthropogenic factors like poaching and competition at man-made permanent watering holes, as well as environmental factors including rainfall and greening [1–11]. Seasonal trends in spatial behaviour of *Loxodonta africana* have been researched extensively throughout Africa [2–11] and some movement trends are shared by elephants across ranges. For example, elephants generally have larger wet season ranges than dry season ranges [2–9, 11–14]. Motility variations within and between populations have various suitable ecological explanations: a period without rainfall can (i) constrain movements to minimise energy expended and water lost [2], (ii)”tether” elephants to water sources [8], or conversely (iii) force elephants to cover greater areas to find sustenance [15]. Crucially, such motility variations are of behavioural interest irrespective of whether elephants change their motility inside a fixed and/or small home range, or partake in large-scale migration between distinct wet and dry ranges.

However this may be, savanna elephants in Northern Kenya seemed to ignore seasons in a comparison of day-to-night speed ratios using a uniform season proxy (i.e. months of the year) and instead demonstrated faster nighttime movements when experiencing elevated poaching levels [10]. Nonetheless, as obligate evaporative coolers [16], elephants are expected to be affected by wet and dry periods, such that travel and consequent moisture loss is minimised during hot daytime hours through droughts when water is scarce, when compared to wet periods when water is readily available. Given the conservation potential of daytime and nighttime travel speeds as indicators of poaching levels [10], a more in-depth exploration of travel speed in relation to local wet and dry periods in the range of each elephant (as opposed to a universal time proxy, e.g. month of the year) is warranted to inform anti-poaching efforts. In addition, if daytime velocities are indeed affected by dry and wet periods, then the increased rainfall irregularity, elevated temperatures and extensive droughts associated with climate change in Africa [17] could theoretically hamper the daytime movements of elephants in the long term. Indeed, a shift towards increased nocturnal activity due to daytime heat stress has been observed for other species like the *Capra ibex* [18]. Worryingly, in this Ibex the increased nocturnal activity was associated with reduced foraging efficiency [18]. It should be noted however, that while this reduction of foraging efficiency may be mirrored to some degree by ‘our’ elephants, the impact of a heat-driven nocturnal shift on feeding efficiency in elephants is likely less severe because elephants have poor vision, and are instead primarily reliant on olfactory cues to locate foods [19–21].

In short, exploring daytime movement velocities in relation to rainfall periods may aid elephant conservation efforts and provide valuable insights into the potential effects of climate change on movements by elephants.

In addition to behavioural trends, several studies explore abrupt behavioural change points (BCPs) occurring at transitions between dry and wet seasons [2, 6, 8, 9, 13, 22]. Behavioural trends and BCPs describe distinct events with trends denoting gradual departures from past averages, while BCPs mark abrupt and structural changes in the properties of time series data [23, 24]. BCPs commonly occur before rains reached the elephants’ locations, and often closely coincide with dry-to-wet season transitions despite annual variation in rainfall [2, 6, 13, 22]. Garstang et al. in particular, detected abrupt changes in persistence velocity (V_p_, a combined measure of speed or displacement and direction of movement) by Namibian elephants using the behavioural change point analysis (BCPA) [6, 25, 26]. Crucially, this analysis proved a robust means of detecting abrupt behavioural changes even when elephants did not migrate in relation to the seasons [6, 25]. Notably, elephants in Northern Kenya also seem to make behavioural changes in response to remote rainfall [14]. An explicit exploration of whether elephants Northern Kenya also change their spatial behaviour preceding the onset of local rains should give a valuable indication whether the behaviours observed in Namibia are common in populations across Africa, or not, and what cues may serve as behavioural triggers.

While the various environmental cues that could triggers these abrupt changes are debated, it appears that thunder cues associated with distant lightning storms provide likely cues [6, 27–29]. Acoustic and seismic thunder cues that are salient in the environment and may singly or jointly trigger widespread behavioural change in elephants–but remain untested. Firstly, acoustic thunder intensities have been modelled up to 142 dB at the source, with power peaks most commonly < 20 Hz [30–37]. This sound wave in turn vibrates the ground, yielding seismic thunder waves with power peaks between 10–40 Hz [28, 34, 36, 38–40]. Elephants are theoretically sensitive to thunder cues in both media [27, 41], but it remains unknown if they respond to natural seismic cues. Our factual knowledge of the audible spectrum of elephants is limited to a singular audiogram of an Asian elephant (*Elephas maximus*) denoting a sensitivity range from 17 Hz—10.5 kHz, peaking at 1000 Hz [42]. However, because Asian and African elephants have the same lower frequency range in their vocalisations (14 Hz, [43–46]), their audible spectrum in the lower frequency range is likely to be similar. The seismic sensitivity of elephants is much less understood as a seismic equivalent of an audiogram does not exist. Seismic playback experiments nonetheless do demonstrate that African elephants are sensitive to at least some seismic vibrations (SVs) between ~ 15–60 Hz [47–49]. Despite this knowledge, the range at which elephants may detect acoustic and seismic thunder remains unclear as waves in both media scatter, attenuate and refract through the environment [6, 27, 28]. Although behavioural changes in response to the salient and reliable acoustic and seismic thunder cues are the focus of this study, it should be noted that other storm cues may also be detected. For example, lightning strikes also produce bright light flashes that could be detected at night despite the poor eyesight of elephants, and rainstorms produce smells that could be detected if they occur in a high concentration with favourable wind speed and direction [6, 50]. Despite the potential role of remote thunder in this context, nobody has thus far correlated the occurrence of remote lightning events (km range) with spatial behaviour changes in African elephants.

In this context, this study first explores whether the daytime movement behaviour of African savanna elephants in Northern Kenya differs between the wet and dry periods perceived by each individual based on local rainfall. For those elephants that are rainfall-sensitive, we subsequently examine when abrupt changes in movement behaviour occur at seasonal transitions points, and if they are most likely triggered by local rainfall or remote lightning as proposed in Garstang et al. [6]. Logically, abrupt changes may be particularly expected in association with the initiation of long-range seasonal migrations, but existing research indicates that local behavioural changes can likewise be expected preceding local rainfall regardless if movements are changed within a small home range [6]. We therefore aimed to apply our analysis to all elephants regardless of, and blind to, whether the subjects perform large-scale migrations or behaviour changes within a smaller home range.

Specifically, the following two hypotheses are tested:

1. During daylight hours, elephants are generally in a fast-moving behavioural state during wet periods and in a slow-moving state during dry periods.
2. Elephants commonly change their behaviour (i) preceding the local arrival of wet periods, and (ii) following lightning strikes from a specific compass heading closely preceding wet periods.

## Methods

### Study area

The studied area spans the Laikipia, Samburu, Meru, Isiolo and Marsabit counties of Northern Kenya (0.3°S–2.3°N, 36.8°E–39.0°E, ~ 62,500 km^2^). The Samburu ecosystem is marked by a semi-arid shrubby landscape, which is comparable to parts of Isiolo and Meru counties [14, 51, 52]. Laikipia includes regions with much agricultural activity and man-made permanent water sources, as well as the lush foothills of Mt Kenya [12, 14, 52]. Parts of Meru and Marsabit are likewise lush because of Mt Kenya and Mt Marsabit [4, 52]. Given the widespread nature of the study area, it encompasses great variety in the amount of rainfall received. For example, historically most rain in the Samburu ecosystem fell through two wet seasons (March–May and October–December) averaging approximately 350 mm per annum with some temporal variation [53]. Mt Marsabit instead received 800–1000 mm annual precipitation, and only 50–250 mm in the semi-arid shrublands surrounding the mountain during between April–May and October–December [4].

### Elephant tracking

The elephants (*n*♀ = 40) in this study were tracked using GPS-collars through a collaborative effort between the Kenya Wildlife Service (KWS) and Save the Elephants conservation charity (STE). Because the elephant tracking data were already existent and were originally obtained for conservation purposes, required no fieldwork, and because this work has been completed in collaboration with STE, permits or ethical approvals were not required.

The individuals are exclusively adult or semi-adults identified by tusk and ear features. Tracked females are each reliable indicators of the location of their respective family groups [10, 54]. In the present study, respective family groups occasionally congregated at rivers and water holes, but generally moved independently through the landscape and did not band together as stable or long-term herds. All positional data were projected on the Universal Transverse Mercator (UTM) WGS-84 reference system for spatial analysis.

### GPS-collar data preparation

Raw positional data were extracted from the STE tracking server and cropped to elephants primarily resident in Marsabit, Isiolo, Meru, Laikipia and Samburu counties that were tracked anytime from 01-01-2015 to 31-12-2019. For each elephant the temporal interval (dT) and straight-line travel speed or velocity (V) were calculated between consecutive fixes. V was subsequently used to exclude erroneous fixes caused by GPS errors, much like in [55]. For consistency, the data of elephants that were tracked once per 15 or 30 minutes (*n* = 13) were down sampled by exclusively retaining the first fix in each hour (thus max. fixes per day, *n* = 24). Fixes that still yielded short dT values (dT < 45 minutes) were secondarily filtered out of the dataset.

To calculate V, the X and Y coordinates were converted to a complex number (X+1iY) to calculate step vectors as (location n-1)–(location n). Step length was in turn calculated as the length of the mean vector. Finally, V was calculated as V = step length/dT. This velocity calculation process is identical to that used for the *bcpa* package [25] for consistency, and yields V in degrees / hour. V was subsequently converted to km / h (from degree/h) by multiplying it by 111, as 1 degree at equatorial latitude ~ 111km [56]. Data gaps indicate substantial GPS errors caused by events like empty batteries, or collar replacements, and are therefore of no use for data validation. The 24 h cut off was selected because the data were ultimately downsampled to one fix per day for the behavioural change point analysis (see Behavioural change point analysis section in Methods).

Once dT and V were calculated, the GPS data were cleaned (see S1 File ii) for more detail) and the standard 7 km / h straight line travel speed threshold was applied as elephants do not travel faster than 6.5km / h [55], resulting in the removal of only 66 datapoints and retention of > 1 million datapoints. Straight line velocities between consecutive fixes were finally recalculated regardless of data gaps as *V = step length/dT* in km / h.

### Selection of comprehensively tracked females

Females (*n* = 40) were the focus because and their family units are more resource-driven with a greater dependency on water to sustain younger members [2, 4, 6, 57], whereas males are mostly solitary and are known to drastically change their behaviour in response to factors such as heightened sexual activity known as musth [1]. Individuals that were tracked for ≥329 days in any one calendar year from 2015–2019 with ≥ 7884 hourly position fixes were retained (328.5 days equals 90% of days in one year and 7884 fixes equals 328.5 days with one fix per hour). Any years during which these females were tracked for < 329 days were also excluded from further analysis yielding 101 years of tracking data across 36 individuals.

### Classifying rainfall events

Daily rainfall data were obtained from the Integrated Multi-Satellite Retrievals for GPM final run (IMERG Final Run, aka IMERG V06) dataset, provided by the National Aeronautics and Space Administration (NASA). Data were extracted from the website for the 5-year period from 2015–2019 between, 4.319°N—3.188°S and 33.795°E—41.251°E in a raster format, with a 0.1°×0.1° spatial resolution.

Most studies with a similar focus assess behavioural changes in relation to wet and dry seasons **(e.g. 12,51)**. However, our study covers a large area with variable rainfall regimes and the areas occupied by elephants in this study do not experience regular seasonality during our study period. Instead, many experience 1–5 periods of substantial rainfall per year (**S2 File)–**possibly a consequence of climate change. We therefore opted to refer to wet and dry “*periods*” instead of “*seasons*” to reflect the irregularities and avoid confusion.

**Fig 1:**
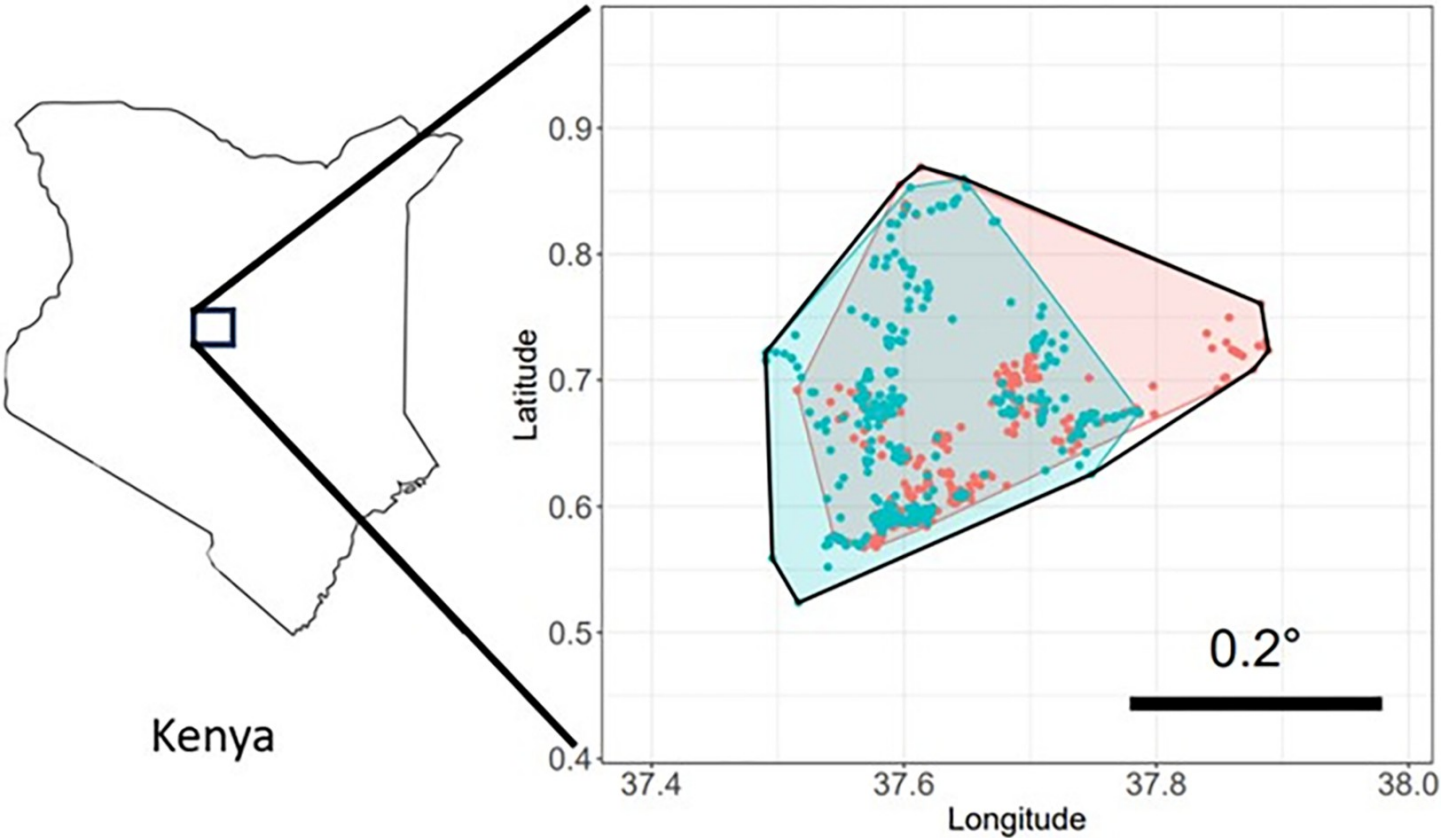
Annabelle’s rectangular home range (RHR) for rainfall extraction, and all daily position fixes recorded during 2015–2017. Blue and red markers respectively indicate position fixes from wet and dry period days (*n*wet = 403, *n*dry = 664), as defined below this figure. The areas populated with wet and dry period fixes are each outlined by their convex hulls (wet area = 748 km^2^, dry area = 804 km^2^), and shaded blue and red respectively. The black lined polygon indicates the total convex hull for all position fixes. The total hull is 995 km^2^ and covers 24.7% of the RHR (4025 km^2^). The RHR itself is visualised by the borders of the Latitude / Longitude border components in the figure. Scale bar indicates 0.2°, or approximately 22 km on the equator [56].

The criteria by which rainfall events were defined were based on distributions of rainfall recorded within each elephant’s rectangular home range (RHR). The RHR was defined as a rectangular area marked by the minimum and maximum latitudes and longitudes of positional fixes recorded for a given elephant from 01-01-2015 to 31-12-2019, with an additional 0.1° (~ 11.1 km) buffer in all directions (e.g. Fig 1). We opted to include 0.1° buffers as elephants may have wandered beyond the recorded latitudes and longitudes between sequential fixes. Additionally, RHRs were selected in favour of complex convex hull polygons, as rainfall volumes were extracted from raster datasets, and RHRs simplified rainfall extraction. It should be noted however, that as a result of this approach, the fraction of the RHR covered by the 0.1 border area is relatively large for individuals that cover smaller regions, and relatively small for those that occupy large regions. To give an indication of the space used by each elephant inside its respective RHR, a total convex hull (smallest convex shape enclosing a set of spatial fixes) was established for each elephant using all positional fixes, and while wet and dry period convex hulls were produced using wet and dry period fixes (Fig 1).

We defined three types of rainfall events (*rainy day*; *wet spell*; *wet period*) within each RHR using distribution-based and semi-flexible criteria. This is because an elephant occupying an extremely dry region may perceive a few of intermittent rainy days as a substantial wet period, whilst another elephant in a wetter region may perceive twice that amount of rainfall as merely a minor wet spell. This method thus encompasses both temporal and spatial flexibility.

In short, *rainy days* are defined as days on which a certain amount of rainfall was recorded in a single raster cell within the RHR. *Wet spells* consist of sufficiently large clusters of rainy days that were also in close temporal proximity to each other. *Wet periods* were a subset of wet spells which were particularly extensive in terms of the number of rainy days and/or were dense terms of the percentage of rainy days within the wet spell. Finally, periods that were not captured as wet periods were by default defined as *dry periods* and could thus include wet spells and rainy days. See S1 File iii) for detailed definitions of rainfall events.

### Rainfall period associated behavioural states

Using the tracks of 36 comprehensively tracked females allowed us to establish whether, during daylight hours, elephants were more commonly in a fast-moving behavioural state through wet periods and in a slow-moving state through dry periods (Introduction, hypothesis 1).

To test this hypothesis, a fast or slow behavioural state had to be estimated for each day. Firstly, the hourly tracks were cropped to daylight hours from > 7:00 and < 18:00 as sunrise and sunset occur at approximately 6:30 and 18:30 respectively, and calculated the mean daytime velocity per day per elephant (throughout we use EAT = UTC + 3). To estimate on which days elephants were in their fast or slow state using the *Viterbi* algorithm of the *moveHMM* package in *R*, we firstly performed simple two-state Hidden Markov Models (HMMs) on the mean daytime velocity data of each elephant with rainfall period (wet or dry) included as a covariate.

HMMs require estimated starting values for the mean and standard deviation values of state (wet & dry) to obtain accurate state estimates. To obtain suitable starting values, the daily mean daytime velocity data of each elephant was divided at the median velocity (yielding ‘slow days’ and ‘fast days’ subsets), and the mean and standard deviations were calculated for each subset. Once the requisite HMM step was completed, the *Viterbi* algorithm was applied to obtain the desired outputs to estimate in which behavioural state (fast or slow) each elephant most likely occurred each day based on the daytime velocity. The assumption of normality of residuals was met based on visual inspection of all QQ plots (**S3 File**). In combination with the daily rainfall for each elephant, days were designated as: 1) fast & wet period (WF), 2) fast & dry period (DF), 3) slow & wet period (WS), 4) slow & dry period (DS) for each elephant.

In line with our hypothesis were wet period days spent in the fast state, and dry period days spent in the slow state (WF & DS). Contrary to our hypothesis are dry period days spent in the fast state, and wet period days spent in the slow state (DF & WS). We therefore grouped the total number of days an elephant behaved according to Tactic A as hypothesised, or Tactic B, per annum, per elephant. See S1 File iv) for visual aid denoting tactic groupings.

A Linear Mixed Model (LMM) compared the number of days per year that the 36 elephants behaved according to Tactic A and Tactic B (***LMM1***). In other words: (*n*DS+*n*WF) vs (*n*DF+*n*WS). The binary Tactic variable was included as fixed effect variable (A or B), whilst the random effect variables (year and ID) were included to account for time effects and non-independence of repeated measures. Inclusion of two random effect variables caused some overfitting, but removal of either variable did not change the model outputs. To accurately reflect the measurements, both variables were retained in the model. Although count data would conventionally be analysed using Poisson models to account for right skewed data, we analysed the data with a LMM because the count data and residual error values were normally distributed based on visual inspection of the residual error Q-Q plot.

### Selection of rainfall-sensitive individuals

Here we focussed on individuals that demonstrated distinct spatial behaviours through wet and dry periods based on the *Viterbi* algorithm outputs, because the BCPA component of this study examines behavioural changes in relation to rainfall (Introduction, hypothesis 2). Hence, we excluded individuals that, during any tracked year, spent a non-substantially different number of days moving fast and slow on wet and dry period days, such that nTactic A days = nTactic B days. Rainfall period-dependent behavioural variations were not necessarily expected to be uniform across all years. This approach was robust for identifying individuals with a behavioural response to rainfall even if the focal individuals do not move large distances to inhabit distinct wet and dry ranges in the studied population.

Four elephants were excluded from further analysis (see Results for more detail). From ***LMM1*** standard error outputs we know that the number of Tactic B days vs Tactic A days would be non-significantly different (*p* > 0.05) if they differed by less than 5.3 (SE Tactic A)+7.5 (SE Tactic B) ~ 13 days (see Results). We opted to remove an elephant from the sample to be used in the BCPA when the absolute difference between *n*Tactic A days and *n*Tactic B days ≤ 13 for any year during which an elephant was tracked.

### Behavioural change point analysis (BCPA)

BCPAs were performed on annual tracks of daily fixes for the 32 retained individuals that were deemed sensitive to rainfall periods (see S1 File v) for BCPA method justification). Tracks of daily fixes were obtained by downsampling the hourly tracking data such that only the fix closest to 12:00 was retained each day. Noon-to-noon daily step lengths, velocity and turning angle were subsequently calculated according to the methods outlined in the GPS-collar data preparation section.

Once reformatted, the *WindowSweep* function and ‘flat’ BCPA [25, 26] were applied (settings: *K* = 2, *Windowsize* = 30 days, *ClusterWidth* = 10 days) to the tracking data. This yielded a series of dates denoting significant and abrupt changes in the mean, variance and autocorrelation of V_p,_ and estimate parameter values between sequential these change points. Points of change were henceforth referred to as Behavioural Change Point Dates (BCPDs). The settings resulted in approximately normal error distributions for all elephants, albeit with a tendency towards thin tails when many BCPDs were detected (**S4 File**). See S1 File iv) for further information about the BCPA process and additional justifications of the selected settings.

The outputs of the *bcpa* package can be interpreted as follows (based on examples in [25]). Firstly, generally positive V_p_ values and high autocorrelation may indicate **directional travel**. Secondly, near-zero mean V_p_, low variance and low autocorrelation may be indicative of **foraging**. Thirdly, a near-zero mean V_p_, greater variance than during foraging, and low autocorrelation, may be indicative of **active random searching,** where a large area is covered with larger movements punctuated by stops and random turns. If instead, an animal forages whilst moving from location A to B during **directional foraging** we may see decreased variance compared to active searching, as well as V_p_ mean and autocorrelation. See S1 File vii) for extensive reasoning behind these interpretations.

### BCPDs and wet period onsets

To test hypothesis 2 i), that elephants commonly change their behaviour preceding the local arrival of wet periods, BCPDs were correlated with rainfall events. Firstly, absolute BCPD counts in the wet and dry periods were compared using a Poisson generalised linear mixed model (GLMM) because the count data were right skewed. This GLMM included BCPA count as the dependent variable, rainfall period (wet or dry) as a binary fixed effect variable, and elephant ID, year, and number of wet and dry period days, as random effect variables (***GLMM1***). The model was checked for overdispersion per the methods in [58] and none was detected (*p* = 0.99), thus meeting assumptions.

Second, we created a BCPD rate variable to adjust for the number of days in the wet and dry periods per year per elephant, and compared these rates. Daily BCPD rates were calculated as nBCPD/nWetPeriodDays OR nBCPD/nDryPeriodDays per elephant per year. After the addition of a constant (*c* = 1) to all rates (a common practice with zeros in datasets [59]), the data were log transformed and analysed with ***LMM2***. For LMM2, BCPD rate was the dependent variable, rainfall period was a binary fixed effect variable (wet or dry) and elephant ID and year were random effect variables. LMM2 suffered from positive skewness of residuals. The exclusion of one outlier (Songa) resolved positive skewness of residuals but did not change model outcomes. The model including Songa was therefore informative despite positive skewness of residuals and used going forward.

Third, we assessed whether BCPD count in 10-, 20- and 24-day windows (henceforth “intra-window” BCPDs) preceding the onset of wet periods, differed significantly from the BCPD count in an equal number of random dry period days outside these windows (henceforth “extra-window” BCPDs). The maximum window size of 24 days was used because it was utilised in a comparable study of elephant movements preceding the onset of rainfall [6]. Pseudo-random selection of an equal sample of extra-window dry days was achieved using the *SetSeed* function in *R* which random to operators, yet is repeatable. Intra- and extra-window BCPD counts were compared using a Poisson GLMM that included BCDP count as the dependent variable, window (intra- or extra-) as a binary fixed effect variable, and year and elephant ID as random effect variables (***GLMM2***). GLMM2 was checked for overdispersion for each window length and none was detected (10-day, *p* = 1.0; 20-day: *p* = 0.69; 24-day, *p* = 0.71), thus meeting assumptions.

Fourth, to remain consistent with the preceding tests, we aimed to assess whether there was a difference in BCPD counts immediately following the onset of wet periods, compared to the remainder of the wet periods, using the same 3 window sizes as noted above. However, this test could not be performed for 20- and 24-day windows because elephants commonly experienced insufficient extra-window wet period days for comparisons with equal sample sizes. As such, we only assessed whether there was a statistically significant difference in BCPD counts in the first 10 days of wet periods, compared to an equal number of random wet period days outside this window, selected using the *SetSeed* function. BCPD counts of intra- and extra-window wet period days were compared using a Poisson GLMM that included BCDP count as the dependent variable, window (intra- or extra-) as a binary fixed effect variable, and year and elephant ID as random effect variables (***GLMM3***). No overdispersion was detected (*p* = 0.66).

Finally, we assessed whether there was a statistically significant difference in BCPD occurrence in the 10 in-window days preceding and following the onset of wet periods. Specifically, BCPD counts in all 10-day windows preceding and following the wet period onset were summed for each year, per elephant. Subsequently BCPD counts in windows preceding and following the on set of wet periods were compared using a Poisson GLMM that included BCDP count as the dependent variable, rainfall period (wet or dry) as a binary fixed effect variable, and year and elephant ID as random effect variables (***GLMM4***). No overdispersion was detected (*p* = 0.95).

### Individuals of interest

To explore their behavioural changes preceding local rainfall, and link behavioural changes with lightning events (hypothesis 2, ii), Delaware and Laresoro were selected as focal individuals based on the outputs of GLMM2 (see results). High numbers of intra-window BCPDs were detected for these individuals (*n* = 3 and *n* = 5 respectively), whilst 0 extra-window BCPDs were detected in an equal number of randomly selected extra-window dry period days. See justification for this approach in S1 File viii.

### Lightning

Distances from individuals of interest to lightning strikes were calculated using the elephants’ daily position. The dataset was then divided into Northern (315° - 44°), Eastern (45° - 134°), Southern (135° - 224°) and Western (225° - 314°) quadrants per the direction of lightning relative to the elephant on the day of the lightning strike. Highly accurate lightning data from the Global Lightning Detection Network (GLD 360) was provided by Vaisala for the 5-year period from 2015–2019 between 4.344°N—3.213°S and 33.770°E—41.276°E, encompassing most of Kenya. Vaisala GLD 360 lightning data for East Africa of this period has a median spatial accuracy of ~ 5 km (personal communication) and captured 11,548,883 cloud-to-ground lightning strikes for the study period.

## Results

### Rainfall associated behavioural states and rainfall sensitive individuals

**Fig 2:**
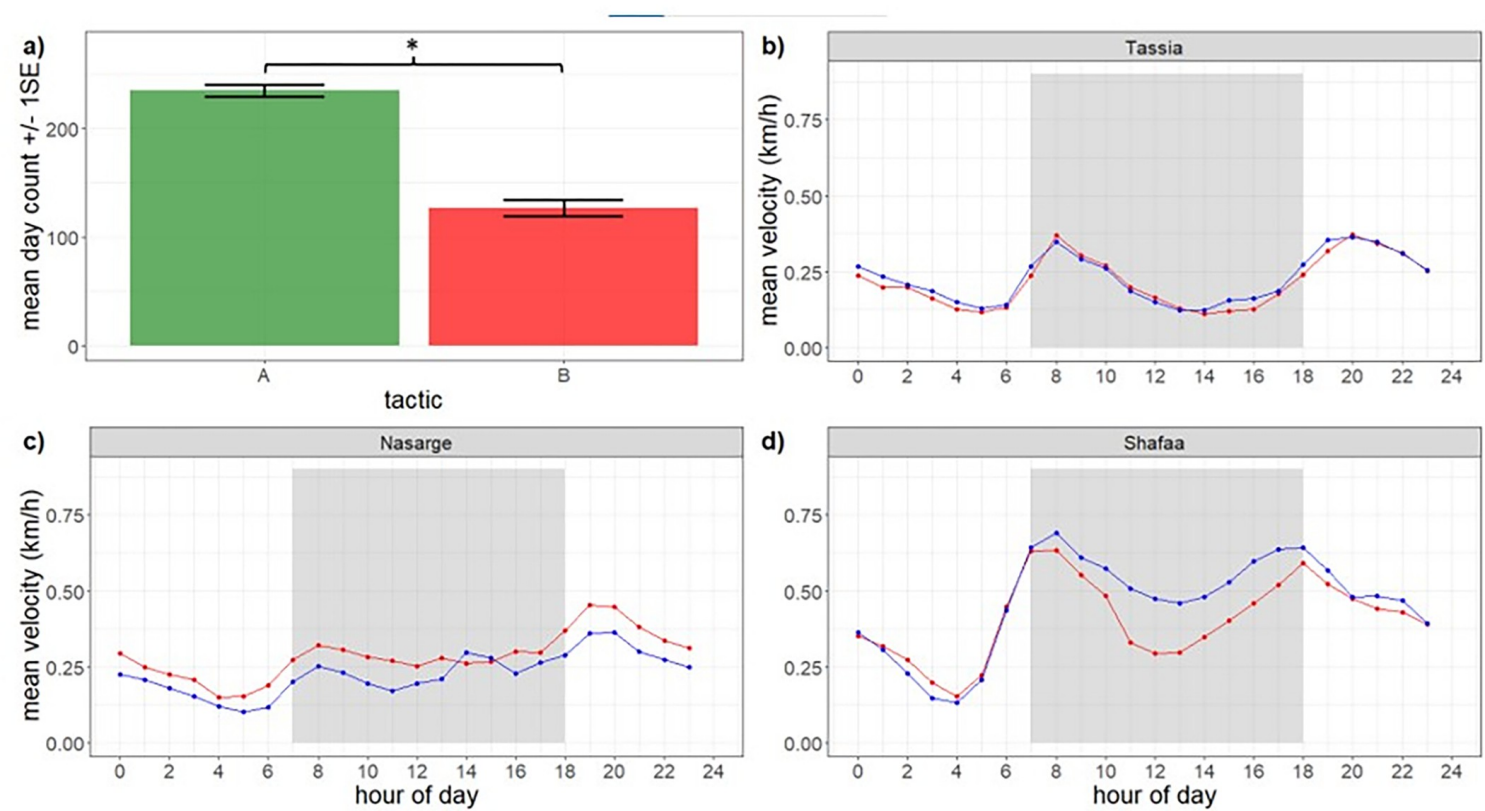
Daytime mean velocity and behavioural states in relation to rainfall periods. Panel a): Mean number of days per year ± 1 standard error (SE) during which female elephants (*n* = 36) behaved according to Tactic A (green) or Tactic B (red). Tactic A describes elephants in their fast state during wet periods and slow state during dry periods (DS&WF), whereas Tactic B describes elephants in their fast state during dry periods and slow state during wet periods (DF&WS). Mean and SE values in bar plot obtained from LMM1 outputs. Panels b—d): Mean velocities for 3 example elephants, across the 24hrs of the day, for the wet periods (blue) and the dry periods (red). Gray shaded area indicates 7:00–18:00 daylight hours from which data were extracted for analysis in association with wet and dry periods.

The focal population showed a propensity for fast movements in wet periods and slow movements through dry periods during the daytime, without notably distinct wet and dry ranges. In total, 211 wet periods and 229 dry periods were detected in the RHRs of 36 sufficiently tracked females during 101 years of collective tracking data from 01-01-2015 to 31-12-2019 (**S2 File).** Elephants collectively spent an estimated 41796 days in the slow state and 31196 days in the fast state (**S3 File**). Moreover, elephants on average spent significantly more days adhering to Tactic A (234.7+/-5.3 days; fast in the wet periods and slow in the wet periods) than Tactic B (126.7+/-7.5 days; fast in the dry periods and slow in the wet periods) (Fig 2a), whilst all elephants except Turungu showed substantial overlap in the areas occupied during wet and dry periods (S5 File). Our results thus failed to reject our first hypothesis, that elephants are generally in a fast-moving behavioural state during wet periods and in a slow-moving state during dry periods.

As per the rainfall sensitivity criterion outlined in the methods (Selection of rainfall-sensitive individuals), only 4/36 individuals were deemed insensitive to wet periods and excluded from further analysis. Tassia is an exemplar elephant that did not adhere more commonly to either one movement tactic and was excluded from further analysis (Fig 2B). Twenty-five individuals exclusively behaved according to one tactic (*n*TacticB = 1, *n*TacticA = 24). Nasarge is the singular elephant that more commonly adhered to Tactic B and generally travelled faster during the day through dry periods (Fig 2C). Shafaa exemplifies the most common rainfall-sensitive spatial behaviour where elephants preferentially adhered to Tactic A such that daytime velocities were generally higher in wet periods than dry periods (Fig 2D). The remaining 7 individuals more commonly adhered to Tactic A or B each year but did not consistently adhere to one tactic across all years. These individuals were also retained for further analysis but are not visualised below (see **S6 File** for wet vs. dry velocity plots for all 36 elephants). Ultimately 32 individuals were deemed rainfall-sensitive and provided 88 years of tracking data to for the BCPA.

Finally, a general lack of spatially distinct wet and dry period ranges was observed, with large-scale overlap of wet and dry period position fixes and convex hulls, except for Turungu (S5 File). Despite wet and dry convex hull overlap, Turungu showed substantial fix clustering towards the west during dry periods and towards the east during wet periods (S5 File, p34). Her total convex hull at 7730 km^2^ was relatively large compared to most elephants in the present study. It spanned 102 km from East-West, and 40 km North-South. Furthermore, based on the range plots (RX) and assuming the same definition of elephant migration as Purdon et al. [9] as “a movement between two non-overlapping seasonal ranges”, Turungu’s behaviour can be classed as migratory. Assuming the extreme ends of the positional fixes, Turungu’s migration is approximately 100km. Movements irrespective of rainfall across the population (established by the total convex hulls, Fig 1) covered areas ranging 300–11700 km^2^ (median = 1428, IQR = 2480), and smaller convex hulls generally covered a smaller fraction of the associated RHR per visual inspection of S5 File and S7 File. In addition, the elephant with the largest RHR and total convex hull (Magado, 29000 km^2^, S5 File, S7 File) showed no distinct with wet and dry ranges, based on the great deal of overlap observed amongst wet and dry period fixes and their respective convex hulls.

### BCPDs and their relationship with wet period onset

**Fig 3:**
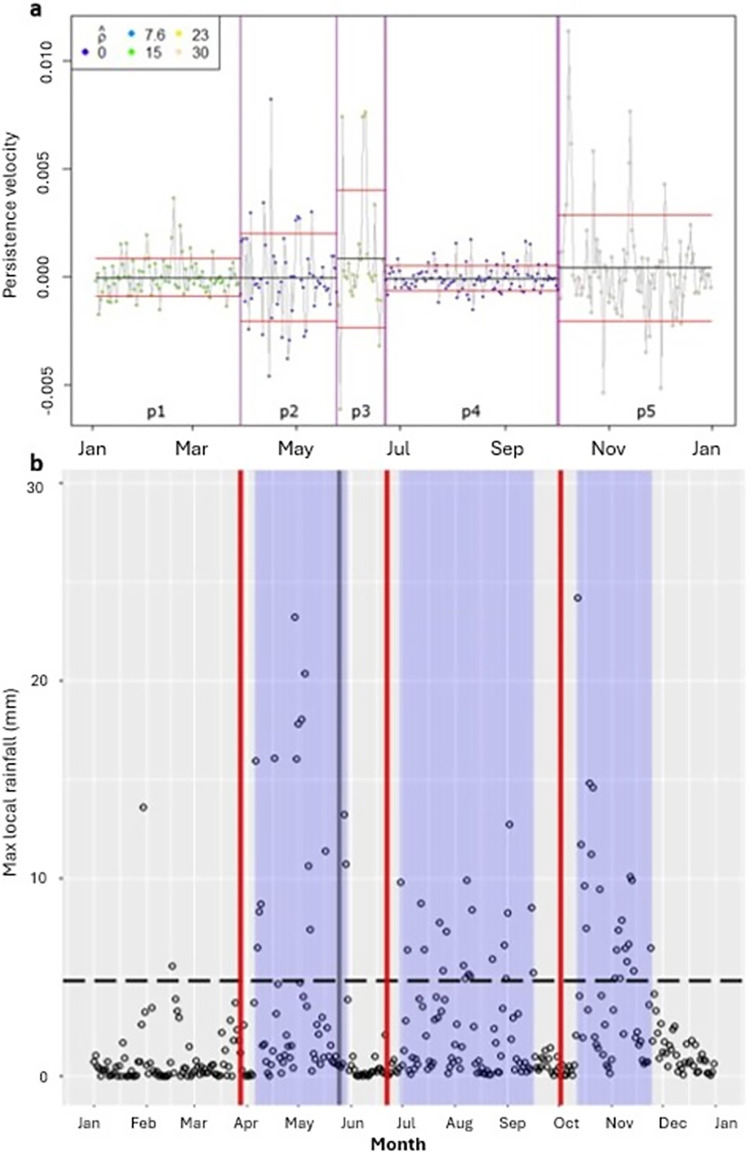
Example behavioural change point analysis output for Laresoro, 2017. a: Four behavioural change point days (BCPDs) vertical lines separate five behavioural periods (p1-p5). Autocorrelation values are indicated by point colours, as outlined in the legend (top left). Mean variances are indicated by the black and red horizontal lines respectively. b: Wet periods in blue. *Rainfall volume criterion* marked by dashed line. Three intra-window BCPDs that closely precede wet periods (within 10-day window) marked by red vertical lines. One extra-window BCPD wet period marked by vertical grey line.

In total 266 Behavioural Change Point Days (BCPDs) were detected by the Behavioural Change Point Analysis (BCPA, **S4 File**) across 88 years of daily tracking data associated with the 32 rainfall-sensitive elephants. Below an example is provided with the changes that occurred. Four BCPDs were detected for Laresoro in 2017 (Fig 3A). BCPD 1 (p1-p2) marks increased *V*_*p*_ variance and decreased autocorrelation but no mean shift. BCPD 2 (p2-p3) marks increased an increased V_p_ mean, variance and autocorrelation. BCPD 3 (p3-p4) marks decreased V_p_ mean, variance and autocorrelation. Finally, BCPD 4 (p4-p5) marks increased V_p_ mean, autocorrelation and variance. BCPD 1,3 and 4 are of interest as intra-window BCPDs (assuming a 10-day window), whereas BCPD 2 is an extra-window wet period BCPD (Fig 3B).

Significantly more BCPDs (*n* = 168) occurred during dry periods than in wet periods (n = 98) (GLMM1: z = - 4.25, p < 0.005). However, BCPD rates were not significantly different (nBCPD/nWetPeriodDays versus nBCP/nDryPeriodDays) when adjusted for the relative length of wet and dry periods (LMM1: df = 143, t = 1.17, *p* = 0.24).

**Fig 4:**
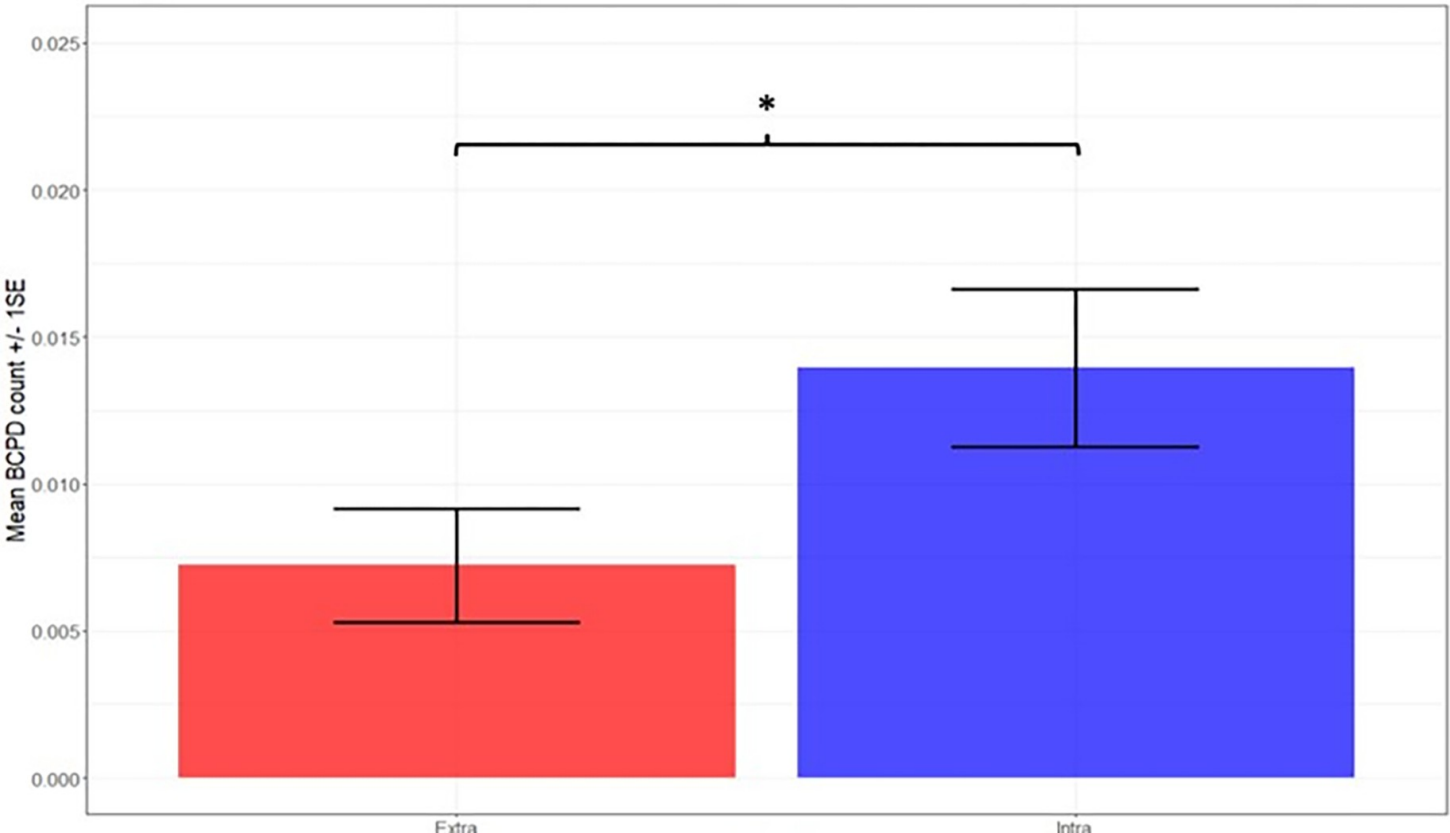
Wet period intra-window BCPD vs. wet period extra-window BCPD occurrence in wet periods. Values obtained from backtransformed estimates of GLMM3 (*p* = 0.043, Table 1). * Indicates significance where p < 0.05 assuming α = 0.05.

Furthermore, there was no propensity for behavioural change closely preceding local wet periods. Intra-window BCPD occurrence preceding wet period onset did not differ significantly from BCPD occurrence in equal number of pseudo-random extra-window dry period days for the 10-day, 20-day and 24-day windows tested (Table 1, GLMM2). The occurrence of intra-window BCPDs following wet period onset was significantly higher than in an equal number of extra-window wet period days (Table 1, GLMM3; Fig 4). However, there was no significant difference in BCPD occurrence between the 10-day windows preceding and following the onset of wet periods (Table 1, GLMM4). Collectively these results reject the first part of our second hypothesis, that elephants commonly change their behaviour preceding the local arrival of wet periods.

**Table 1:** Outputs for GLMM2—GLMM4 comparing BPCD occurrence.

**GLMM2:** dry period intra-window BCPD vs. dry period extra-window BCPD					
Window Size (days)	*n*Days_Total_	*n*Days_Intra_	*n*Days_Extra_	*z*	*p*
10	3936	1968	1968	0.61	0.54
20	7860	3930	3930	1.73	0.08
24	9394	4697	4697	1.32	0.19
**GLMM3:** wet period intra-window BCPD vs. wet period extra-window BCPD					
Window Size (days)	*n*Days_Total_	*n*Days_Intra_	*n*Days_Extra_	*z*	*p*
10	3870	1935	1935	2.00	0.046*
**GLMM4:** dry period intra-window BCPD vs. wet period intra-window BCPD					
Window Size (days)	*n*Days_Total_	*n*Days_Preceding_	*n*Days_Following_	*z*	*p*
10	3903	1968	1935	0.57	0.57

BCPD = behavioural change point days. *n*Days_Preceding_ = intra-window dry period days. *n*Days_Following_ = intra-window wet period days. Indicates significance where p < 0.05 assuming α = 0.05.

### Individual descriptive assessment

Some individuals were notable within the study population and demonstrated a strong propensity for changing their behaviour closely preceding wet periods. To test what may drive these changes and test hypothesis 2 ii), that elephants change their behaviour following lightning strikes from a specific compass heading closely preceding wet periods, the behaviours of individuals were described in relation to remote lightning and local rainfall events. Given the hand-picked nature of these data, it was analysed descriptively and statistical tests were not utilised.

For Delaware and Laresoro, intra-window dry period BCPDs were generally preceded by a rain pulse, happened concurrently with a rain pulse, or as rainfall increased towards their local wet periods (7/8) (**S8 File**). Only in one instance, there is no evidence of local rain before or during the dry intra-window BCPD (Delaware, 2018-02-23, **S9 File**). Extra-window dry period BCPDs also commonly coincided or closely followed rainfall pulses. For Delaware this was particularly common (7/8), whilst it was substantially less common for Laresoro (1/3) (**S8 File**).

Furthermore, for Delaware and Laresoro, Eastern lightning exclusively occurred during pre-BCPD periods (4/8) but never occurred on days of intra-window BCPDs, and Northern lightning was also substantially more common in days preceding behavioural changes (5/8) than on days of BCPDs (2/8). Southern and Western lightning were instead common throughout the year (**S9 File**).

Lightning is generally closer in days preceding BCPDs than on the BCPD itself. In all cases except Laresoro, 2016-10-23, the closest lightning in the 5 days preceding the BCPDs (green points) were substantially closer to the elephant (< 50 km) than the strikes on the days of behavioural change (> 100 km, red points). In case of the exception, the closest lightning strikes were roughly equidistant (45 km vs 50 km) on the day of the behavioural change point through the days preceding the BCPD respectively. Note that this BCPD was marked by elevated local rainfall (**S9 File**).

The direction of changes in V_p_ parameter estimates for each dry intra-window BCPD were remarkably different for Laresoro and Delaware. Laresoro’s intra-window BCPDs (assuming a 10-day window), were mostly marked by a decreased V_p_ mean, variance and autocorrelation values, except for the final instance where all values increased (Table 2, **S10 File**). In contrast, Delawere’s intra-window BCPDs generally displayed a V_p_ mean increase to just above zero, medium/high variance, and low autocorrelation (Table 2, **S10 File**).

**Table 2:** Directional description of changes of V_p_ components for intra-window BCPDs (assuming 10-day window).

Identity	BCPD date	Mean	Variance	Autocorrelation
Laresoro	13-04-2016	**↓**	**↓**	**↓**
	23-10-2016	**↓**	**↓**	**↓**
	28-03-2017	**--**	↑	**↓**
	22-06-2017	**↓**	**↓**	**↓**
	02-10-2017	**↑**	**↑**	**↑**
Delaware	27-03-2016	↑	↑	**↓**
	09-10-2017	↑	↑	**↓**
	23-02-2018	**↓**	↑	**--**

↑ = increase, **↓** = decrease,– = no substantial change.

The behavioural states associated following dry period intra-window BCPDs were also markedly different between these two elephants. See **S11 File** for descriptions of when these elephants theoretically performed stints of foraging, directed travel, directed foraging and variations of active searching, based on the BCPA outputs.

## Discussion

### Rainfall associated behavioural states

The results failed to reject our first hypothesis, that elephants are generally in a fast-moving behavioural state during wet periods and in a slow-moving state during dry periods because elephants spent significantly more days adhering to Tactic A (fast in the wet periods and slow in the dry periods) than Tactic B (fast in the dry periods and slow in the wet periods) (Fig 2A). The range plots (S5 File) further show that the elephant population in the present study does not make large scale movements between distinct wet and dry ranges, with the exception of Turungu. Instead the elephants had a singular local home range that was used during the wet and dry periods alike. Notably, the associated total convex hulls could be very large (up to 29000 km^2^, S7 File)

Movements by most elephants in the present study were likely restricted by resource availability or driven by the minimisation of water loss based on behavioural tactics that have been associated with wet and dry periods, and water and forage availability [2, 8, 15]. Some elephants also appear to compensate for reduced daytime travel by covering more ground at night when it is dark and cool during dry periods. For example, Malkadaka and Siginte demonstrate increased velocities at night during dry periods compared to wet periods, whilst their daytime velocities are reduced compared to wet periods **(S6 File)**. This observed shift towards increased nocturnal activity mirrors the shift observed for ibex in association with heat stress [18]. Given the association this nocturnal shift and reduced foraging efficiency in *Capra*. *ibex* [18], we also urge for the study of elephant foraging in association with increased nocturnal activity and daytime heat stress, so as to best understand the potential implications of global warming.

Counter to the restricted dry period movements observed for Malkadaka and others, man-made watering holes [8] and rainfall pulses may have reduced the physical constraints and have enabled or driven Nasarge to move large distances between scattered water sources and search for patches of fresh forage. Such behaviour is possible in Laikipia because substantial rainfall pulses occur through the dry periods in and at the foothills of Mt Kenya (**S3 File**) and Laikipia has numerous man-made watering holes. Collectively our findings indicate that megafaunal movement studies that separate daytime and nighttime movements may serve a crucial role in ecological behaviour studies when weather patterns become increasingly irregular due to climate change.

The present study also highlights the importance of defining seasons based on individual weather patterns because a universal proxy can falsely assume that rainfall received by all elephants is comparable each month and therefore yield no significant seasonal effect. Defining wet and dry periods based on individual and local rainfall distributions may have been a major driver behind the disagreements with another study utilising a similar study population [10]. In the present study, the irregularity in rainfall across years and across a large study area indicate variable wet and dry periods between individuals and years **(S2 File).** Ihwagi and colleagues [10] instead explored whether there is a seasonal effect on night-day speed ratio using ‘month’ as a proxy for seasonality for all elephants. However, it cannot be ruled out that there is a genuine change in behaviour between studies as they do not temporally overlap, or that the night-day speed ratio measure and statistical tests deployed in [10] were more conservative. Moreover, Ihwagi and colleagues did not exclude males from the study (comprising nearly half of the total sample) which may have influenced their results compared to those presented here. Nonetheless, research is likely to be more informative if the climate variables in climate related ecology studies incorporate some degree of individual flexibility–particularly in face of climate change.

### BCPDs and wet period onset

Our results also reject the first part of our second hypothesis, that elephants commonly change their behaviour preceding the local arrival of wet periods. As such, the temporal association between BCPDs and rainfall in the present study differs from that described for the non-migratory elephants in the Kunene region of Namibia that do not leave their dry ranges [6]. Most notably, behavioural change points preceding the onset of wet seasons was twice as common as following the onset (assuming a 24-day window) [6], whereas BCPD occurrence in the present study was not significantly different preceding and following onset (assuming a 10-day window) (Table 1, GLMM4). In addition, during dry periods we find no evidence for a high concentration of BCPDs closely preceding wet periods compared to the rest of the dry period (Table 1, GLMM2)–which is perhaps not unexpected if no large distance has to be covered to reach another feeding range.

Present findings are instead more in alike the behaviours observed in South African elephants, of which the majority increased their speed shortly following the onset of wet periods (in the study referred to as *regional rainfall breakpoints*) [2]. We specifically found that elephants do not simply change their behaviour at a random point during wet periods but are statistically more likely to do so within the first 10 days following the onset of wet periods (Fig 4, Table 1 GLMM3). A quick response to rainfall also aligns with the close tracking of precipitation-driven vegetation changes in by elephants Northern Kenya [4]. It should be noted that in case of this the South African elephants [2], it is unclear whether the focal elephants generally migrate between distinct wet and dry regions or show breakpoints in their local behaviour, and therefore may pertain to a different movement behaviour than that which is denoted in the present study.

BCPDs in the present study may be less common preceding wet periods compared to [6] for numerous reasons. Firstly, and potentially most important, is that wet periods experienced by elephants in their RHRs in Northern Kenya described in this study, and the South African Kruger region covered by Birkett and colleagues [2] are more temporally-irregular and less defined than the seasons experienced in Namibia [6]. As observed in **S2 File**, wet periods with substantial rainfall occur at various points in the year, changing between individuals and across years for our study population. Moreover, even when a period is generally dry, wet spells and rainy days are not uncommon in our study region and Kruger park [2] compared to Namibia [6]. As such, the predictive power of cues from remote rainfall and associated with thunderstorms postulated by Garstang and colleagues [6, 27], is theoretically lower in Northern Kenya. Secondly, our discrepancies may be due to a behavioural difference between males and females. In [6] that 6/9 elephants changed their behaviour preceding local rainfall, and that all these individuals were male. The present study instead exclusively explores female behaviour because females are theoretically more resource-driven with a greater dependency on water to sustain younger members [2, 4, 6, 57]. Thirdly, given the more ephemeral nature of some wet periods in the present study (**S2 File**) compared to those noted in [6], we were unable to robustly compare pre- and post-onset BCPD occurrence for 20- and 24-day windows whilst maintaining equal sample sizes. We therefore utilised a substantially smaller window size (10 days) than Garstang and colleagues [6]. Finally, our sample size is 4 times larger and thus less prone to behavioural outliers. Although we argue that our behavioural findings are likely different from those described by Garstang and colleagues due to environmental differences between the studied regions, it can thus not be ruled out that our discrepancies in findings are (in part) due to true behavioural differences between sexes, or a difference in window size or sample size discrepancies.

### Individual descriptive assessment

Delaware and Laresoro demonstrated a strong propensity for changing their behaviour closely preceding wet periods. For these elephants, the co-occurrence of rain pulses and intra- and extra-window dry period BCPDs (**S8 File**) suggests that small local rainfall pulses during dry periods rain can trigger a behavioural change for these elephants; further supporting findings by [2, 4] that even ephemeral local rainfall may trigger behavioural changes. Note that we refer to rainfall pulses instead of wet spells or rainy days, as rainfall amounts observed during such pulses were commonly much lower than the *rainfall volume criterion* to denote a *rainy day* for that elephant (**S2 File and S8 File**). Despite such indications however, it ultimately remains unknown if this pattern is due to an actual response behaviour or simply because minor local rainfall pulses are common throughout dry periods, as many rainfall pulses are also not followed by, or coincident with, BCPDs. In short, although Delaware and Laresoro appear to respond to rainfall pulses, the limited data does do not allow for definitive conclusions.

Delaware and Laresoro may alternatively/also have changed their behaviour several days after remote thunder cues from the North and East. Where Southern and Western lightning were common throughout the year, Northern and Eastern lightning was particularly common closely preceding wet periods, and during pre-BCPD periods, and was therefore theoretically more informative to elephants (**S9 File**). Elephants may thus have responded to environmental events where a local rainfall pulse and lightning in a Northerly and/or Easterly direction coincide to anticipate a local wet period. Statistical exploration of this complex three-way interaction (preferably in a seasonally-simpler environment like Namibia) is required to draw conclusions with certainty.

The distance at which elephants may detect/respond to lightning and associated thunder remains uncertain. Some speculate that elephant could respond to the acoustic thunder cues from storms up to 300 km away [6], whilst others indicate that acoustic thunder is inaudible at distances > 30 km as a result of upward refraction causing the acoustic waves to travel high above the elephants [28, 37, 60]. We are the first to assess the relation between behavioural changes in elephants and actual lightning events (as opposed to the occurrence of a rainstorm) and have not found an indication of responses to lightning at distances approaching 300 km. Our data indicates that, if elephants respond to lightning events at all, it is probably restricted to lightning within 50 km during the days preceding dry intra-window BCPDs. At 50 km the acoustic and seismic components of lightning strikes may both be detectable by elephants if upward refraction of acoustic waves is minimal. Moreover, at night lightning flashes may be visible to elephants even despite their poor eyesight [6, 50]. To draw robust about thunder sensing however, the acoustic, seismic and visual sensitivity of African savanna elephants must be firmly established, and the magnitude of thunder cues within their sensitivity range (presumably > 14 Hz for acoustic waves) must be recorded over long distances.

The behavioural data also offers little evidence for the use of remote thunder cues to instigate behavioural change. Much like the elephants studied in Namibia [6], Laresoro and Delaware do not display consistency in the direction of change in each of the three components assessed by the BCPA for intra-window BCPDs preceding wet periods (mean, variance and autocorrelation of V_p_, **S10 File** and **S11 File**). In addition, the behaviours that were transitioned into preceding wet periods were also inconsistent between the two elephants, and while intra-window BCPDs of Delaware consistently initiated active searching, some other periods were not spent in an active searching state (**S10 File** and **S11 File**). Laresoro’s adherence to a foraging behaviour between June–October each year, despite the absence of a wet period at the start of that period in 2018, likewise suggests that neither the changes to that specific behavioural mode nor maintaining that behavioural mode, was necessarily related to a wet period (**S11 File**). Instead the Laresoro may simply change to a certain behaviour at a fixed point in the year regardless of external triggers, or respond to rainfall pulses in the dry period around June. Note however that this is hard to demonstrate conclusively as many rainfall pulses do not result in BCPDs. Ultimately, these findings reject the second component of our second hypothesis, as there is little evidence for the use of remote thunder cues to trigger behavioural change closely preceding local wet periods.

Finally, our results demonstrate that BCPA is a useful tool to establish behavioural changes for individuals, and how behavioural changes quantitatively relate to changes in resource availability for a larger group of animals. However, it should be note that many insights from estimated behaviour states between points of change remain theoretical until *in-situ* behavioural observations are quantitatively linked to BCPA outputs. As such we strongly urge such a study to take place–potentially starting in an environment like North-West Namibia with extensive dry periods, few wet spells and abrupt starts to wet periods [6], so as to obtain the clearest behavioural representations in the future.

## Conclusion

A number of previous studies proposed that abrupt movement changes by African savanna elephants at seasonal transition points commonly precede the arrival of local rains. Some also suggested that the acoustic and seismic thunder cues from remote storms were a likely trigger of these abrupt changes in movement behaviour and ecology. Our study tested this thesis by analysing the movement behaviour of a population of 40 female elephant matriarch-led elephant groups in Northern Kenya (*n* = 40) in relation to local rainfall and distant lightning strikes.

In summary, these elephants moved differently in the wet and dry periods, and our analysis suggests that behavioural change was most likely caused by local rainfall pulses–not by remote lightning. Firstly, we failed to reject our first hypothesis that: elephants are generally in a fast-moving behavioural state through wet periods and in a slow-moving state through dry periods, during daylight hours. Despite notable variations between individuals, the population had an overwhelming propensity for faster daytime movements in the wet periods. Secondly, we rejected both parts of the second hypothesis that: elephants change their behaviour (i) preceding the local arrival of wet periods, and (ii) following lightning strikes from a specific compass heading closely preceding wet periods. (i) There was no indication that behavioural changes were more likely to occur closely preceding the onset of wet periods. Instead, we detected a propensity for behavioural changes shortly after the start of wet periods compared to any other time during the wet periods. This notably mirrors behaviour patterns previously described for elephants in South Africa, where wet periods were also irregular. (ii) The individual assessment further highlights a propensity for behavioural changes to occur at the same time as local rainfall pulses during dry periods. Although Northerly and Easterly lightning often precedes local rainfall, and thus could be used by elephants to anticipate a local wet period, the remarkable co-occurrence of local rainfall and behavioural change points suggests that in this population rainfall was the more likely cue to adjust movement behaviour and this ecology. The irregularity in rainfall periods in Northern Kenya may have undermined the predictive power of remote thunder, and minimised reliance on this environmental cue compared to other regions, like Namibia, with more regular rains. A follow-up study that empirically links estimated behaviour states from behavioural change point analyses with observed behaviours, completed in a region with regular seasons, would provide invaluable insights into the environmental drivers of movement ecology of elephants and possibly also other megafauna.

On a broader scale we have also demonstrated that in a world facing rapid climate change, we should no longer assume that seasons remain temporally regular, or uniform across study regions. Instead, our findings show the importance of defining perceived wet periods as experienced by individual elephants based on rainfall distributions incorporating temporal and spatial flexibility. Furthermore, we demonstrate that animals can behave very differently between the wet and dry periods when the daytime behaviour is separated from the nighttime behaviour, and how this behavioural separation may improve our understanding of the potential impact of climate change on large herbivorous endotherms. This demonstrates not only the importance of considering seasonal variation as perceived by individual elephants, but also the importance of separating day- and nighttime behaviours of individuals to further our understanding of elephant movement ecology and conservation.

## Supporting information

S1 File Supplementary methods. Additional information and justification of methods where noted in the main body of the manuscript.
(PDF)

S2 File Annual local rainfall plots for all elephants. Maximum local rainfall volume in mm. Blue blocks indicate wet periods. Horizontal black line indicates rainfall volume criterion for a day to be denoted as ‘rainy’. Thick black vertical line indicates the start and end of elephant tracking period.
(PDF)

S3 File Individual behavioural state plots, state maps, residual plots and Q-Q plots. State plot indicates per observation (i.e. day) if elephant was in the fast or slow state. Velocity plot indicates the associated average daytime velocity. State 1 = slow state, State 2 = fast state. Latitude-longitude plot indicates when where each elephant was in state 1 or 2. Residual and QQ plot indicates model fit.
(PDF)

S4 File Annual Behavioural Change Point Analysis (BCPA) outputs. For each elephant and each year the Q-Q plot, residual plot, and persistence velocity BCPA output plots are provided. BCPA output plots indicate abrupt annual changes in persistence velocity marked by vertical purple lines. Colours of points in the BCPA output plots indicate autocorrelation value.
(PDF)

S5 File Ranges of all 36 comprehensively tracked females. Blue and red markers respectively indicate position fixes from wet and dry period days. The areas populated with wet and dry period fixes are each outlined by their convex hull and shaded blue and red respectively. The black lined polygon indicates the total convex hull for all position fixes. The RHR itself is approximately visualised by the borders of the Latitude / Longitude border component in the figure. Scale bar indicates 0.2°, or approximately 22 km on the equator (53).
(PDF)

S6 File Average hourly, wet versus dry period mean velocity for all elephants. Blue indicates wet period velocity, and red indicates dry period velocity. Gray block indicates nighttime hours.
(PDF)

S7 File Range sizes for all 36 comprehensively tracked females. Range sizes in km^2^.
(PDF)

S8 File BCPD occurrence in relation to rainfall. Blue blocks indicate wet periods. Red vertical lines indicate intra-window dry period BCPDs. Gray vertical lines indicate extra-window BCPDs. Blue line indicates daily rainfall amount. Horizontal dashed line indicates rainfall volume criterion for a day to be denoted as ‘rainy’.
(PDF)

S9 File Lightning distance, intra-window BCPDs and local rainfall. Four upper plots indicate the distance to lightning in each of the 4 quadrants (North, East, South, West) from the position of the focal elephant (up to 300 km), for each year. To facilitate easy relation to local rainfall, two copies of rainfall plots are also provided below these quadrants. Vertical red lines indicate an intra-window dry period BCPD.
(PDF)

S10 File BCPA outputs and rainfall periods. Upper plots on each page indicate the annual changes in persistence velocity for each elephant. The colours of points indicate the respective autocorrelation value. The lower plots indicate the associated local rainfall, with red vertical lines indicating intra-window BCPDs and gray vertical lines indicating extra-window BCPDs.
(PDF)

S11 File Behavioural descriptions based on BCPA outputs. Detailed description of the theoretical behaviours based on the estimated behaviours of the BCPA states for Delaware and Laresoro.
(PDF)
