# Supplementary material for: Local rainfall is more likely than distant thunderstorms to affect movement behaviour in Northern Kenyan elephants
Source: PLoS One. 2024 Dec 23;19(12):e0307520. doi: 10.1371/journal.pone.0307520 (PMC11666045; doi:10.1371/journal.pone.0307520)
Supplement: S1 File — Additional information and justification of methods where noted in the main body of the manuscript. (PDF) [file pone.0307520.s001.pdf]

Supplementary methods

i) Skipping data gaps

V was not calculated for the first fix in the 2015 - 2019 GPS track of each elephant as the preceding location is an unknown and was likewise not calculate for the first fix following a substantial data gap, > 24 hours or 1440 minutes.

ii) Data cleaning elaborated:

First, fixes that occurred within 45 minutes after the preceding fix were deleted because short temporal intervals between fixes are more likely to cause high velocity outliers due to ephemeral behaviours, like short-duration sprints. This is a problem given that short sprints captured by short dT fixes can yield hourly speeds which are unachievable for elephants for a full hour. In cases where dT exceeded 60 minutes the fixes were generally obtained at a multiple of the fix-collection-frequency of the collar (e.g. 120 or 180 minutes for collars collecting fixes every 60 minutes). Whilst longer time intervals are suboptimal, high value velocity outliers such as those caused by short inter-fix intervals cannot occur and were therefore not removed. The exception to this retention is where dT > 24 h as noted above, where the fix location following this gap was retained but V was not calculated. Subsequently the standard 7 km / h straight line travel speed threshold was applied as elephants do not travel faster than 6.5km / h (1).

iii) Detailed definitions of rainfall events

Rainy days: A day was considered rainy when the maximum rainfall recorded in the RHR of the focal elephant exceeds the *rainfall volume criterion* (Table 1). Daily rainfall maximums are the maximum rainfall recorded by a single raster cell ($0.1° \times 0.1°$) within the RHR of a focal elephant. ***Wet spells***: Clusters of rainy days were considered part of a wet spell if they were temporally separated by less than days than *cluster criterion A* (Table 1), and the number of rainy days in the temporally close cluster exceeded *cluster criterion B* (= 4 rainy days). One to four rainy days that were temporally removed from other rainy days were hence considered a small cluster, but not a wet spell. Because *cluster criterion A* was distribution-based it was inherently flexible, however, we applied upper and lower bounds to its value to avoid extremes. Specifically, after calculating and compiling the *cluster criterion A* values for all 36 elephants, the population-wide first quantile and median values were used as the lower and upper bounds. Hence if the cluster criterion A of an elephant was < 19 (Q1) it was rounded up to 19, if > 28 (median) it was rounded down to 28, and if it ranged from 19-28 it was unaltered. The value of *cluster criterion B* was likewise distribution based. Specifically, it was obtained by calculating and collating the number of rainy days in the rainy clusters established across the population, and extracting the population-wide median value (= 4). ***Wet periods***: If wet spells either included more rainy days than 2 × *cluster criterion B* (i.e. >8), OR exceeded the *concentration criterion* in terms of rainy days, they were deemed wet periods. To meet the concentration requirement, the percentage of rainy days in a wet spell had to exceed the *concentration criterion* calculated per individual (Table 1). Ultimately the combination of these criteria was able to identify short and intense wet periods, as well as temporally extensive wet periods with a lower concentration of rainy days.

Note that the criteria applied for each component were obtained from rainfall distributions and are therefore (semi-) flexible. The points identified on the distribution (e.g. mean, mean-1SD, Q1,

median) were manually chosen to sensibly describe rainfall experienced by elephants in the present study, based on visual inspection of rainfall plots. It is thus likely that these points would be adjusted to yield sensible results if studying rainfall at another location or time.

Table 1. Rainfall event type criteria. SD = standard deviation.

**Criterion**	**Criterion calculation**	**Additional information**
Rainfall volume criterion	$= mean + 0.5\ SD\ max\ daily\ rainfall\ volume\ recorded\ across\ study\ period$	N/A
Cluster criterion A	$= \frac{(mean + 1SD\ no.dry\ days\ preceding\ rainy\ days) + (mean + 1SD\ no.dry\ days\ following\ rainy\ days)}{2}$	Bounded at Lower: Q1 = 19 Upper: Q2 = 28
Cluster criterion B	$= Q2\ value\ of\ the\ no.rainy\ days\ in\ all\ rainy\ day\ clusters\ recorded\ across\ the\ elephant\ population$	Q2 = 4
Concentration criterion	$= mean - 1\ SD\ percentage\ of\ rainy\ days\ within\ all\ wet\ spells\ recorded\ for\ that\ elephant$	N/A

iv) Visual aid for grouping of wet and dry period behaviour

		Period	
		Dry	Wet
State	Slow	DS	WS
	Fast	DF	WF

Figure 2. Possible rainfall period and behavioural state combinations. Days consistent with hypothesis in green (collectively Tactic A), days inconsistent with hypothesis in red (collectively Tactic B).

v) BCPA justification

The behavioural change point analysis was identified as the most suitable method to detect when behavioural changes occurred for multiple reasons. Firstly, it was specifically designed to be robust to data gaps in GPS tracking data as is common when tracking wildlife (2,3). Secondly, it estimates the importance or significance of changes within time-series data without the need to estimate the number, and composition or nature of discrete states, as is the case with HMMs (2). And finally, BCPA was used to determine points of behavioural change in association with rainfall events in (4) to which we want to compare our results. Applying the BCPA to day-to-day tracking data ensured that our results were directly comparable to those by Garstang et al. (4).

vi) BCPA settings

The *WindowSweep* function of the *BCPA* package (3,5) in *R* was applied to identify abrupt changes, or breaks in V_p. As outlined in the introduction, V_p captures both speed of the animal (or more

accurately, day-to-day displacement), as well as tendency for that movement to continue in the current direction. V_p is calculated as $V \times cos(Turning\ Angle)$ where turning angle describes the change in compass heading between subsequent fixes. To identify points of change, the *WindowSweep* function firstly shifts an analytical window (supported by the *BestBreak* function) sequentially through the data to establish significant break points in the mean, variance or autocorrelation of V_p. Secondly the “flat” analysis used in this study identifies Behavioural Change Point Days (BCPDs) that it deems significant by clustering closely neighbouring breaks, and estimates parameter values (mean, variance and autocorrelation of V_p) between sequential BCPDs. For more information about this these processes see (3,5).

To apply the *WindowSweep* function correctly, one must provide appropriate *WindowSize*, *sensitivity* (*K*) and *ClusterWidth* settings. *WindowSize* determines the size of the analytical window that is shifted through the data frame where larger windows identify fewer but more robust change points (the creators of the BCPA package recommend *WindowSize* be set to no less than 20). *K* is a secondary sensitivity setting (standard setting = 2), where lower values reduce sensitivity to changes. *ClusterWidth* determines the temporal distance at which breaks are deemed to be in the same cluster. For our study we set *Windowsize* = 30 (i.e. 30 days) and *K*=2 as standard, to analyse the tracks of all 32 females. *WindowSize* and *K* values were set such that they yielded approximately normal error distributions for all elephants, albeit with a tendency towards thin tails when many BCPDs were detected (Additional File 4). *ClusterWidth* was finally set such that breaks occurring within 10 days of each other were considered part of one cluster. This *ClusterWidth* setting was selected as we are interested in BCPDs associated with wet periods across whole years and not BCPDs occurring on consecutive days.

vii) Interpretation of BCPA outputs

The outputs of the *bcpa* package can be interpreted as follows (based on examples in (3)). Fast and directed movements yield high mean V_p values. High variance values are caused by highly variable movements with directional changes and stop/start events. Finally, high autocorrelation values indicate directed and correlated movements such that behaviour from a previous data point is a more reliable indicator for the following data point (e.g. if an individual moves fast with minimal turning one day, it is likely to do the same the following day). Given the formula of V_p ($V \times cos(Turning\ Angle)$), V_p values can be negative. As velocity is fundamentally nonnegative, any negative V_p values result from turning events where $270° < turning\ angle > 90°$ such that $cos(Turning\ Angle)$ is negative - indicating that the animal turned back on itself. Positive V_p values instead indicate directional changes in a forward direction such that $90° < turning\ angle > 270°$. Variance around a near-zero mean V_p consequently indicate that direction changes occurred equally commonly in both the forward and backward direction, or randomly. The highest variance is achieved when an animal moves fast each day, in a straight forward direction (0°), or directly backward direction (180°) such that $cos(turning\ angle) = +1\ and\ -1$ respectively. Fast undirected movements with random directional changes are thus likely to have a near-zero V_p mean, but high variance and low autocorrelation. Slow undirected movements are likely to have a near-zero V_p mean with low variance and low autocorrelation. Fast directional movements instead would yield generally positive V_p values and high autocorrelation, where a greater mean V_p indicates a higher speed and/or less turning. A generally positive set of V_p values with high variance indicates that while movements were generally in the forward direction, the velocity of travel may have been varied and/or direction of travel was less consistent. Conversely, if an animal travelled fast but commonly

turned back on itself one may see a set of strongly negative V_p values. Furthermore, if the animal instead were to vary its velocity and/or turning angles between 90° - 270°, V_p values would generally remain negative, but variance would be higher. From this information various behavioural modes can be established, based on examples provided in (3). Firstly, generally positive V_p values and high autocorrelation may indicate **directional travel**. Secondly, near-zero mean V_p, low variance and low autocorrelation may be indicative of **foraging**. Thirdly, a near-zero mean V_p , greater variance than during foraging, and low autocorrelation, may be indicative of **active random searching** where a large area is covered with larger movements punctuated by stops and random turns. If instead, an animal forages whilst moving from location A to B during **directional foraging** we may see decreased variance compared to active searching, as well as V_p mean and autocorrelation.

viii) Selection of individuals of interest

Because this population did not display a statistical propensity for behavioural change preceding the onset of wet periods (outlined in the results), we opted to specifically focus on two hand-picked individuals that did display such a propensity, and explore their behavioural relation to rainfall and lightning events to uncover what may have driven their behavioural changes preceding wet periods. Given the hand-picked nature of these individuals, we describe the associations and provide summary statistics instead of performing statistical tests.

Bibliography

1. Okita-Ouma B, Koskei M, Tiller L, Lala F, King L, Moller R, et al. Effectiveness of wildlife underpasses and culverts in connecting elephant habitats : a case study of new railway through Kenya ' s Tsavo National Parks. 2021;(July 2020):624–40.

2. Edelhoff H, Signer J, Balkenhol N. Path segmentation for beginners: An overview of current methods for detecting changes in animal movement patterns. *Mov Ecol*. 2016;4(1).

3. Gurarie E, Andrews RD, Laidre KL. A novel method for identifying behavioural changes in animal movement data. *Ecol Lett*. 2009;12(5):395–408.

4. Garstang M, Davis RE, Leggett K, Frauenfeld OW, Greco S, Zipser E, et al. Response of African elephants (Loxodonta africana) to seasonal changes in rainfall. *PLoS One*. 2014;9(10).

5. Gurarie E. Behavioral Change Point Analysis in R : The bcpa package. 2013;1–16.
