# Supplementary material for: Local rainfall is more likely than distant thunderstorms to affect movement behaviour in Northern Kenyan elephants
Source: PLoS One. 2024 Dec 23;19(12):e0307520. doi: 10.1371/journal.pone.0307520 (PMC11666045; doi:10.1371/journal.pone.0307520)
Supplement: S2 File — Maximum local rainfall volume in mm. Blue blocks indicate wet periods. Horizontal black line indicates rainfall volume criterion for a day to be denoted as ‘rainy’. Thick black vertical line indicates the start and end of elephant tracking period. (PDF) [file pone.0307520.s002.pdf]

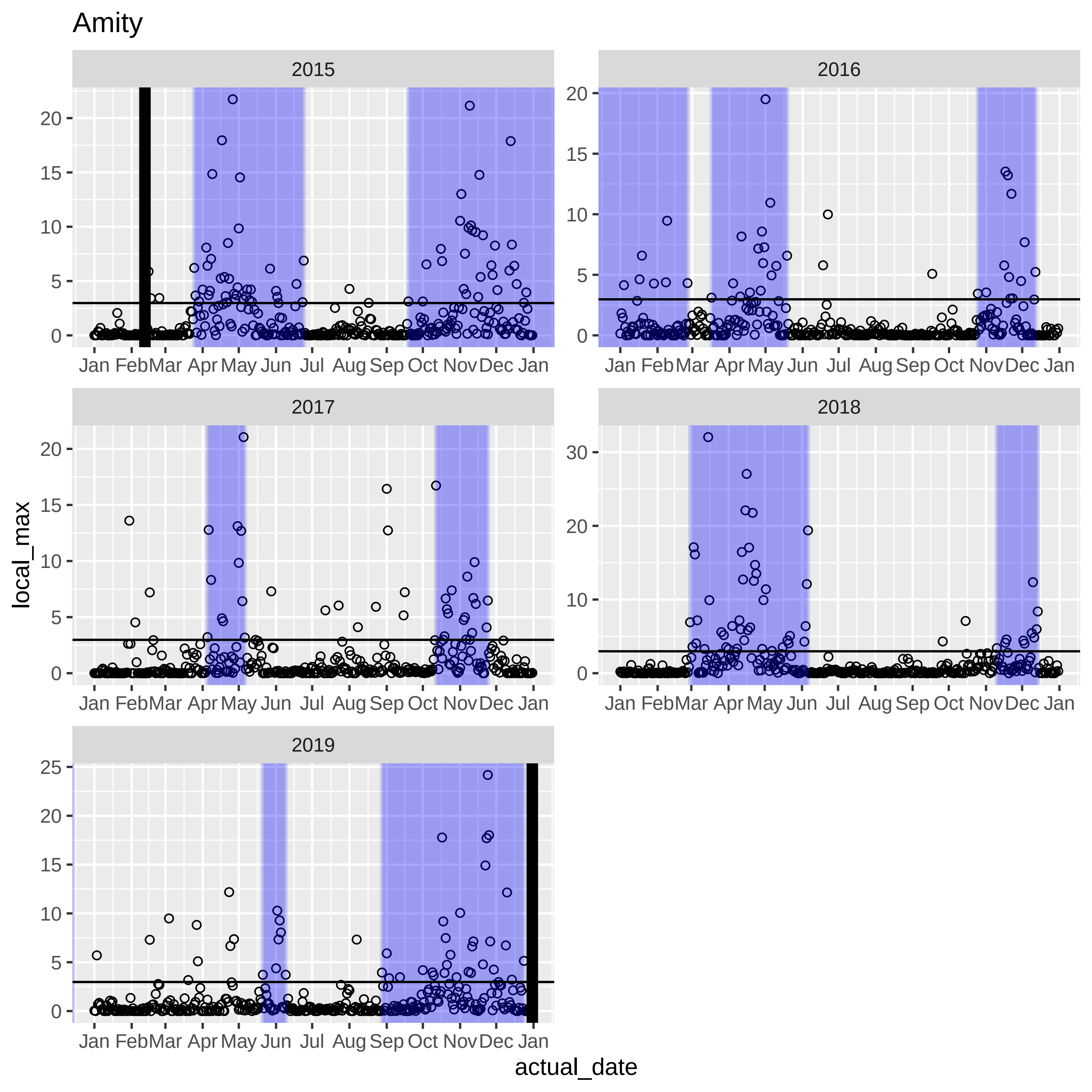

Amity
2015
2016
2017
2018
2019
local_max
actual_date
Jan Feb Mar Apr May Jun Jul Aug Sep Oct Nov Dec Jan

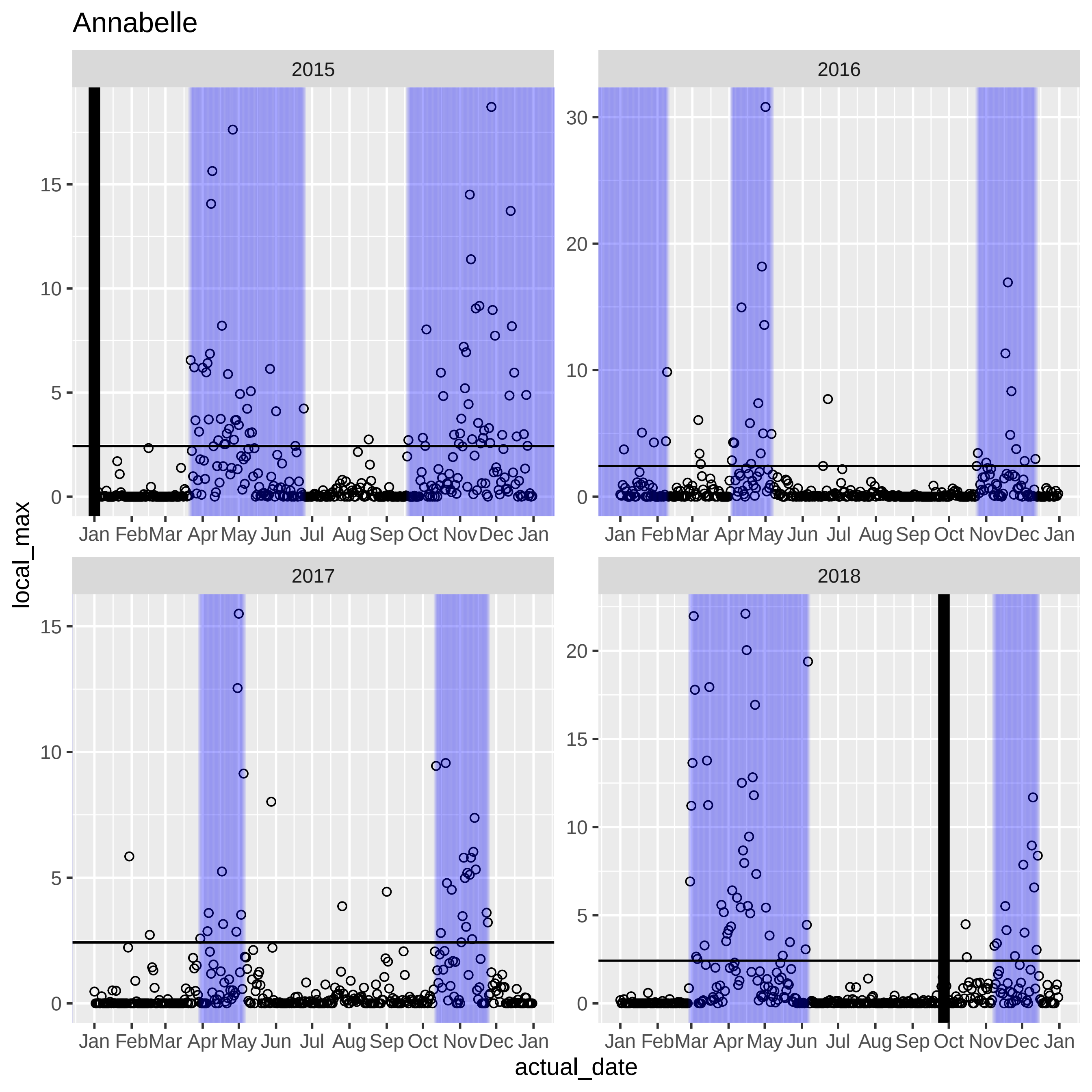

Annabelle
2015
2016
2017
2018
local_max
actual_date
Jan Feb Mar Apr May Jun Jul Aug Sep Oct Nov Dec Jan
0
5
10
15
20
30

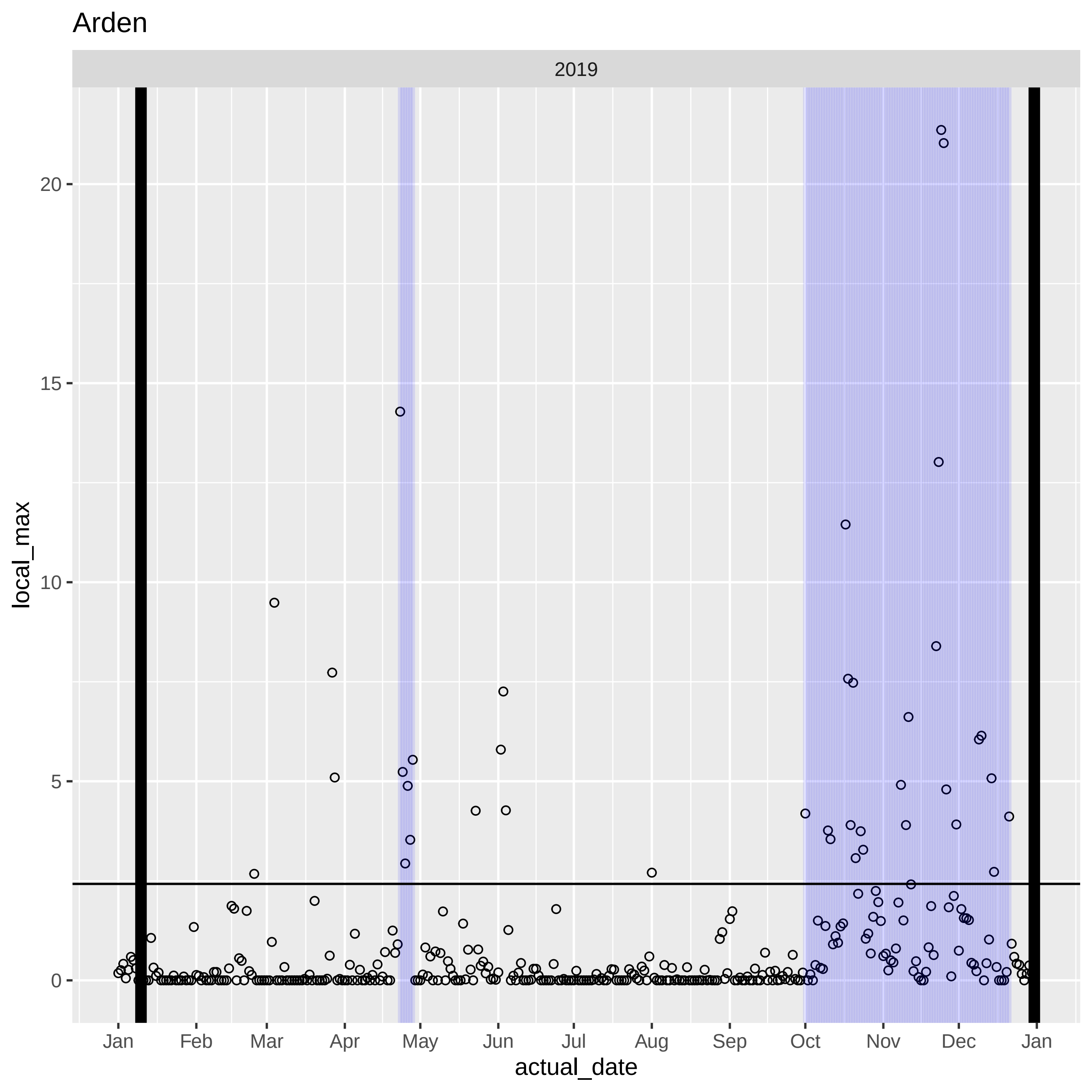

Arden
2019
local_max
actual_date
0
5
10
15
20
Jan
Feb
Mar
Apr
May
Jun
Jul
Aug
Sep
Oct
Nov
Dec
Jan

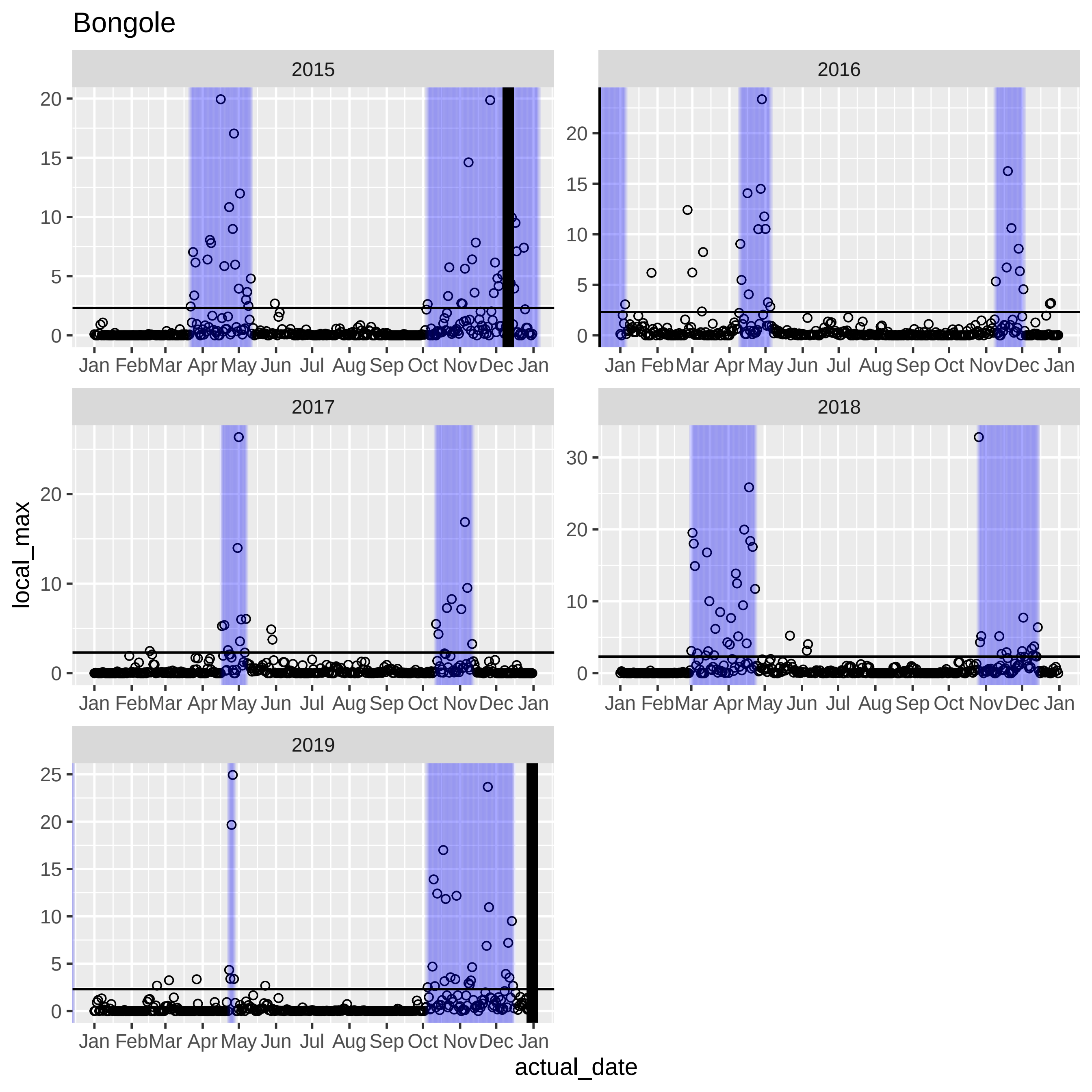

Bongole
2015
2016
2017
2018
2019
local_max
actual_date
Jan
Feb
Mar
Apr
May
Jun
Jul
Aug
Sep
Oct
Nov
Dec
0
5
10
15
20
25
30

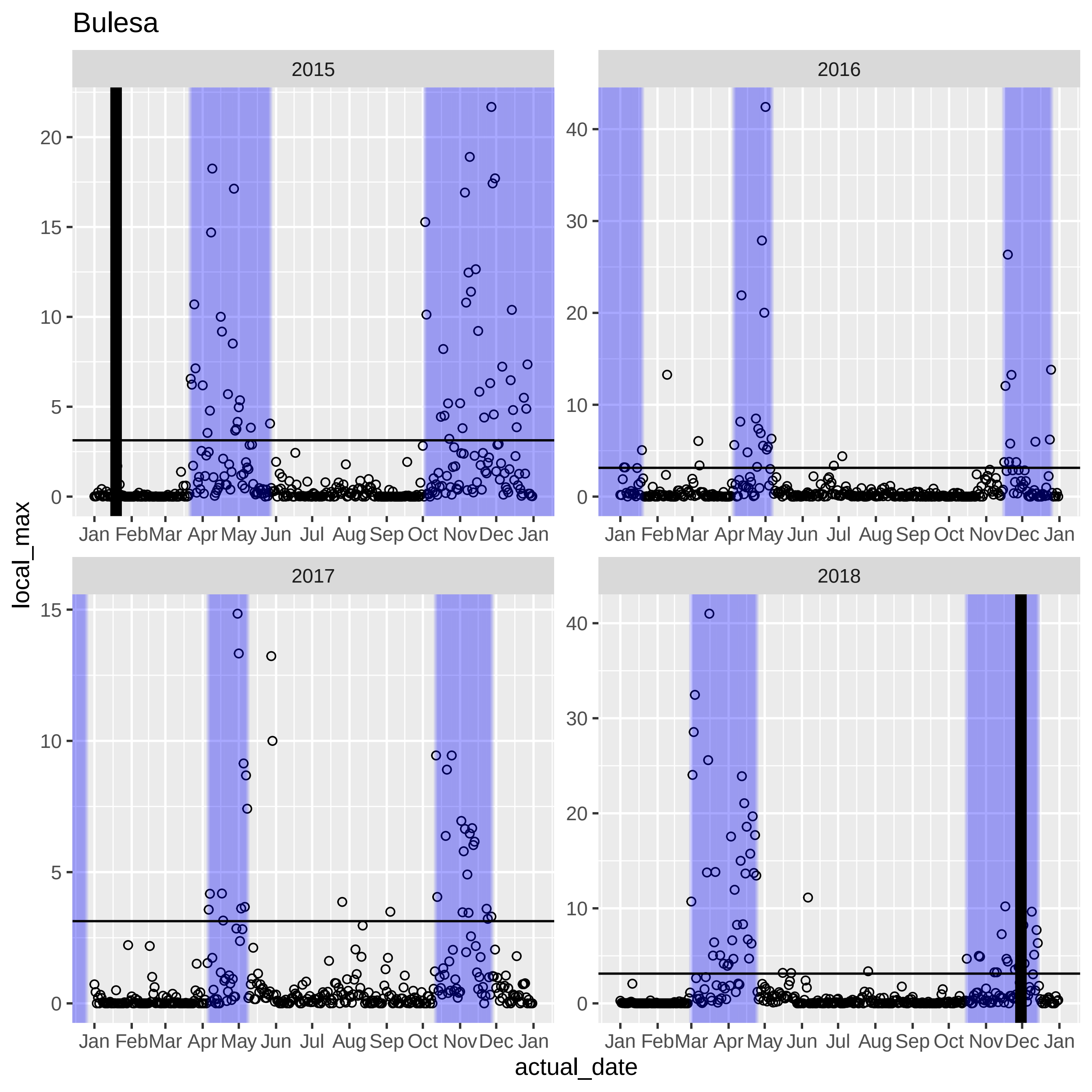

Bulesa
2015
2016
2017
2018
local_max
actual_date
Jan Feb Mar Apr May Jun Jul Aug Sep Oct Nov Dec Jan
0
5
10
15
20
30
40

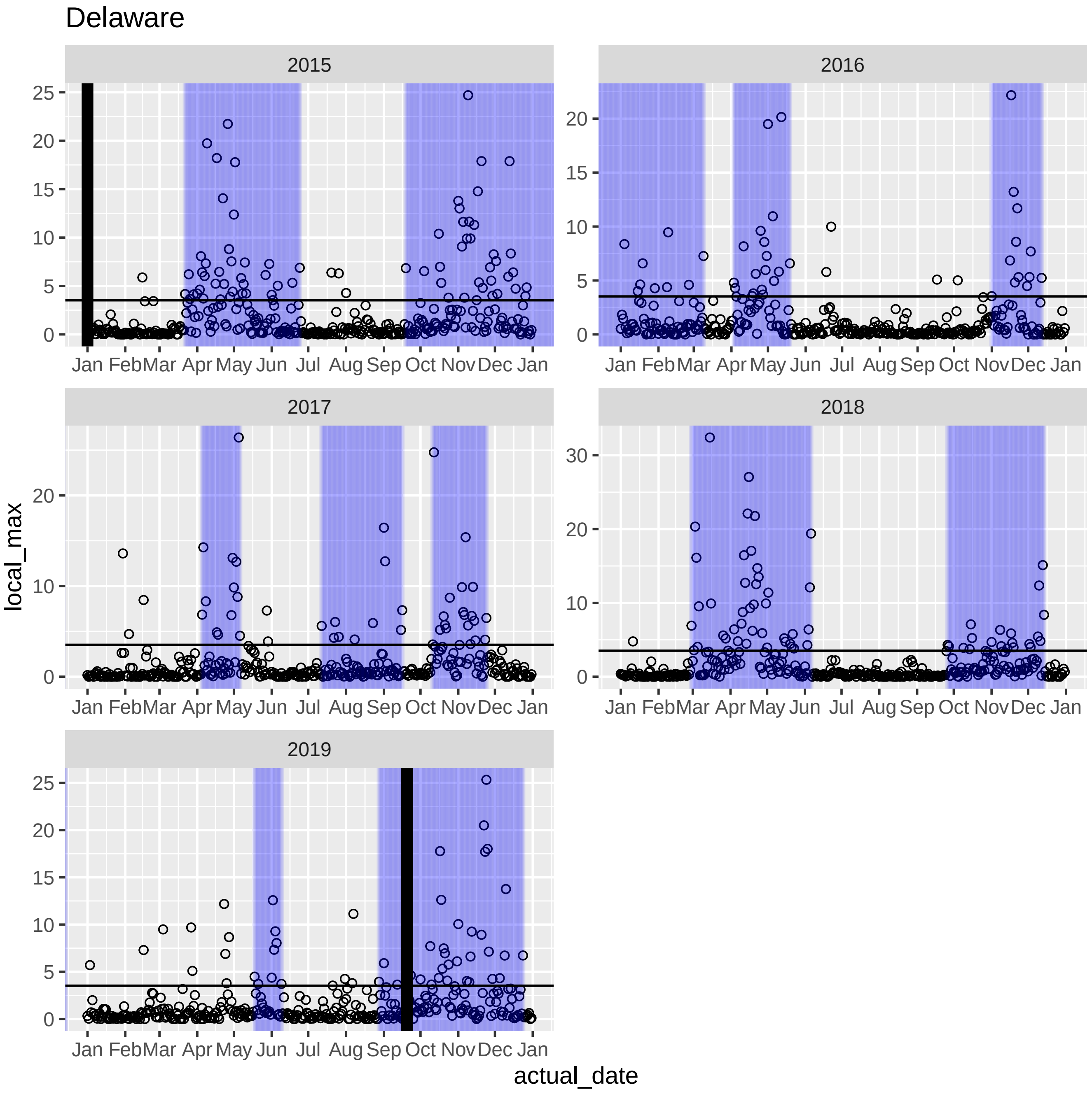

Delaware
2015
2016
2017
2018
2019
local_max
actual_date
Jan
Feb
Mar
Apr
May
Jun
Jul
Aug
Sep
Oct
Nov
Dec

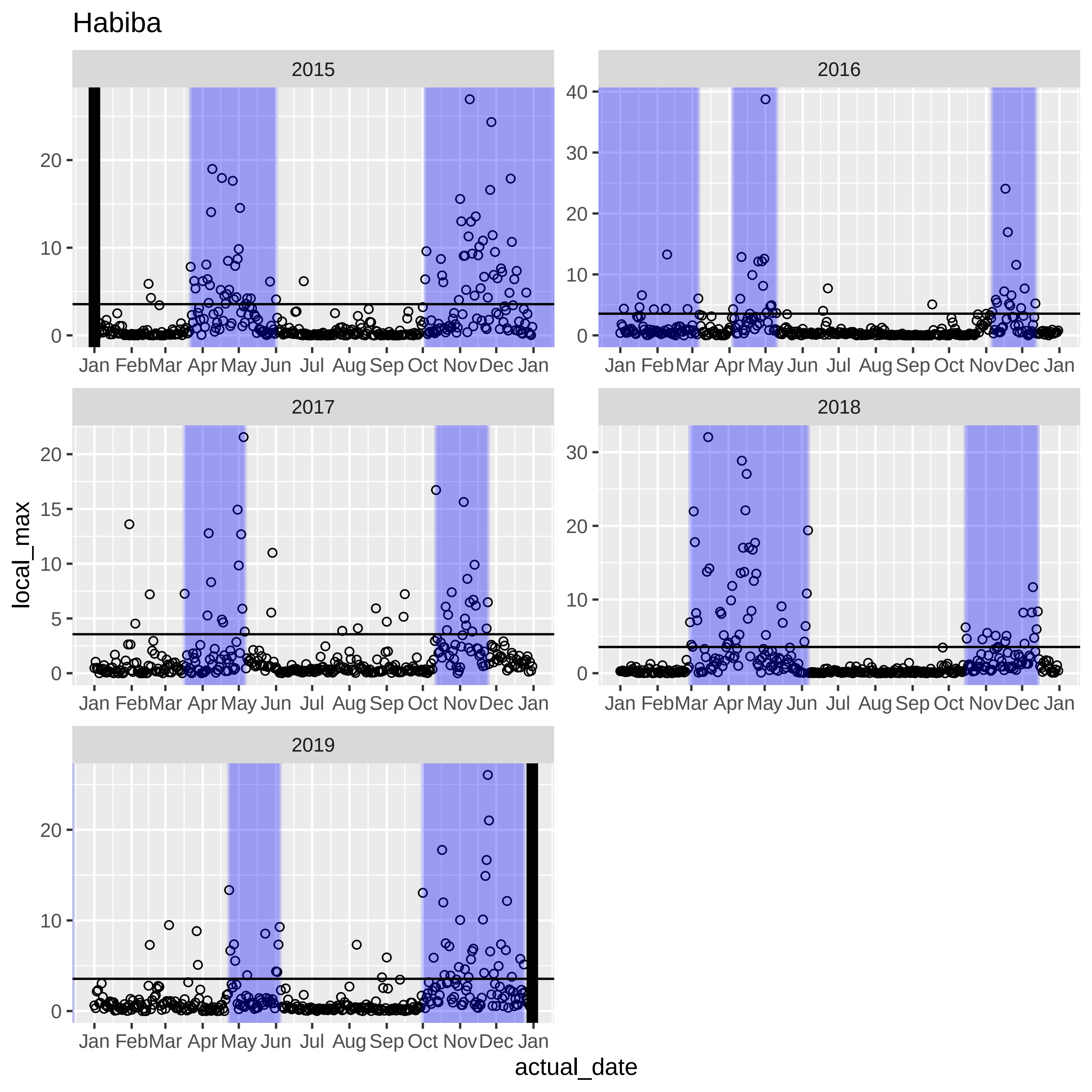

Habiba
2015
2016
2017
2018
2019
local_max
actual_date
Jan Feb Mar Apr May Jun Jul Aug Sep Oct Nov Dec Jan
0
10
20
30
40
5
15

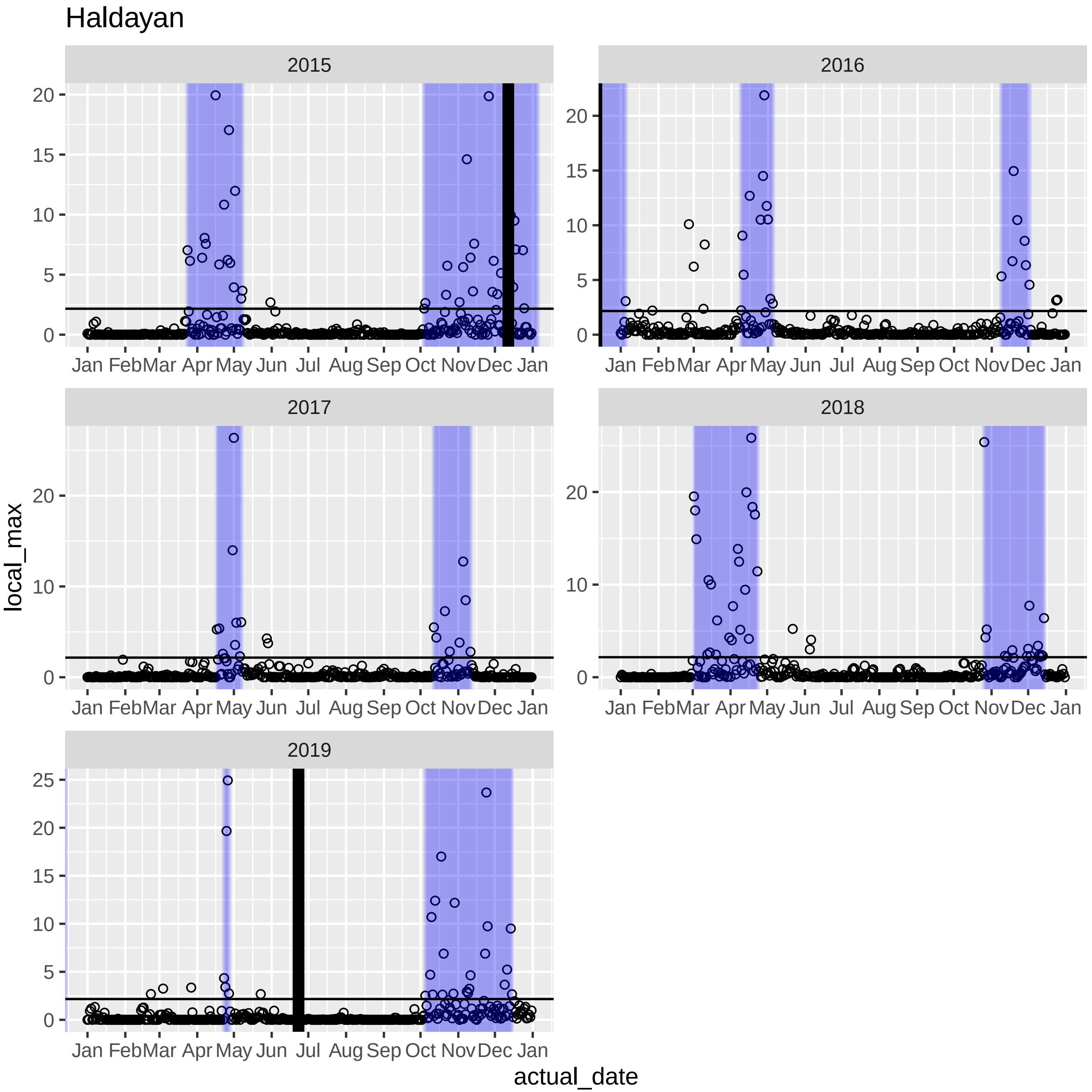

Haldayan
2015
2016
2017
2018
2019
local_max
actual_date
Jan Feb Mar Apr May Jun Jul Aug Sep Oct Nov Dec Jan
0
5
10
15
20
25

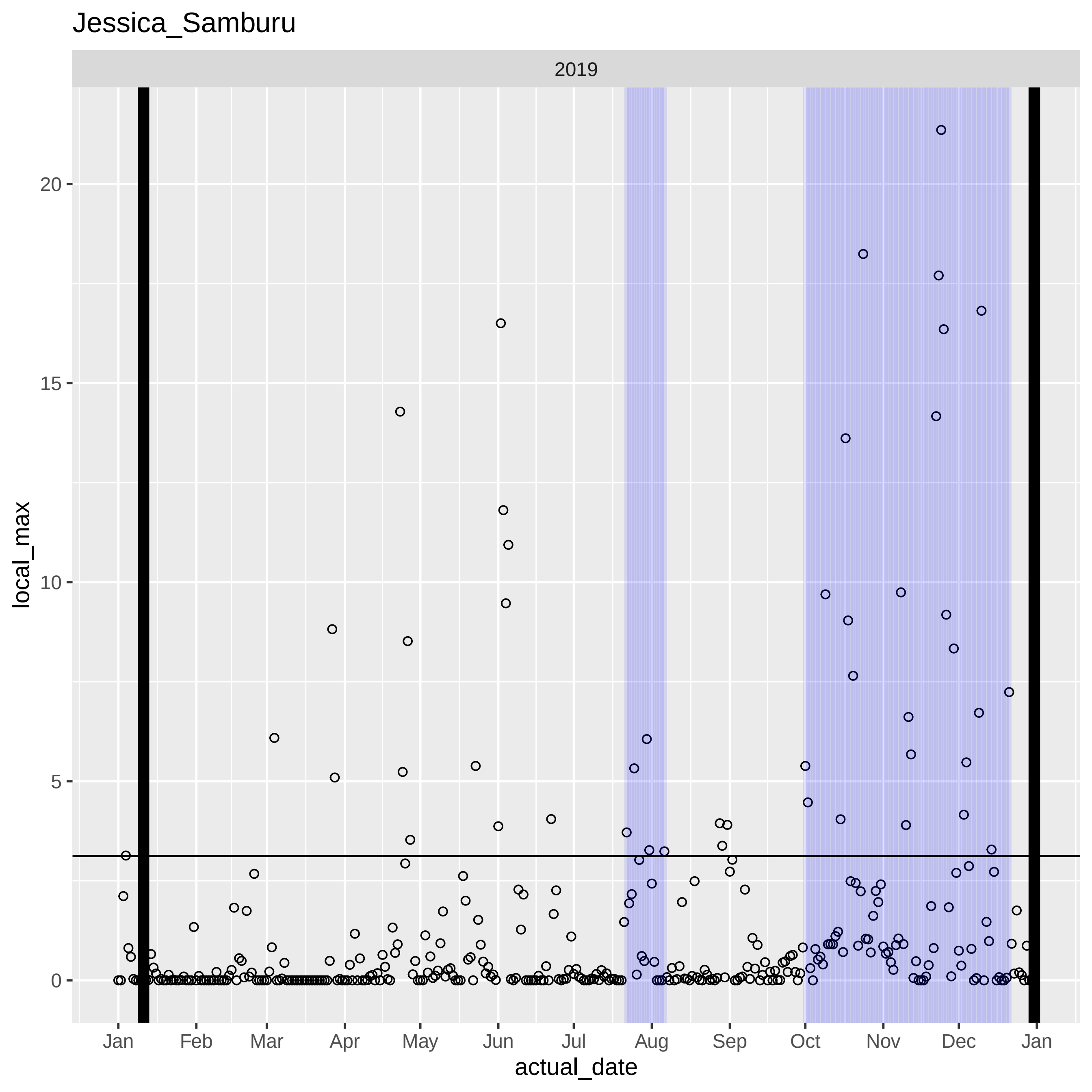

Jessica_Samburu
2019
local_max
0
5
10
15
20
actual_date
Jan
Feb
Mar
Apr
May
Jun
Jul
Aug
Sep
Oct
Nov
Dec
Jan

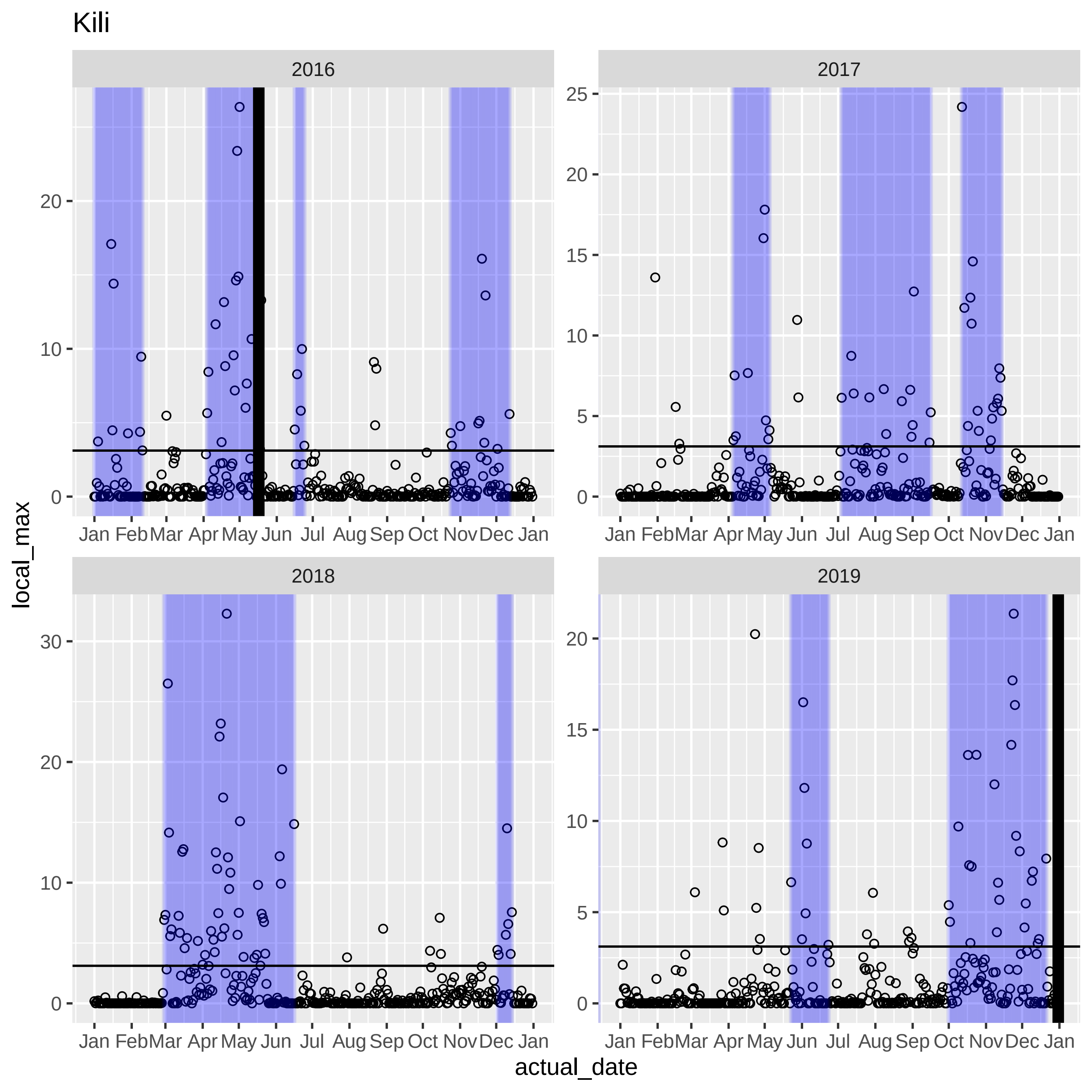

Kili
2016
2017
2018
2019
local_max
actual_date
Jan Feb Mar Apr May Jun Jul Aug Sep Oct Nov Dec Jan

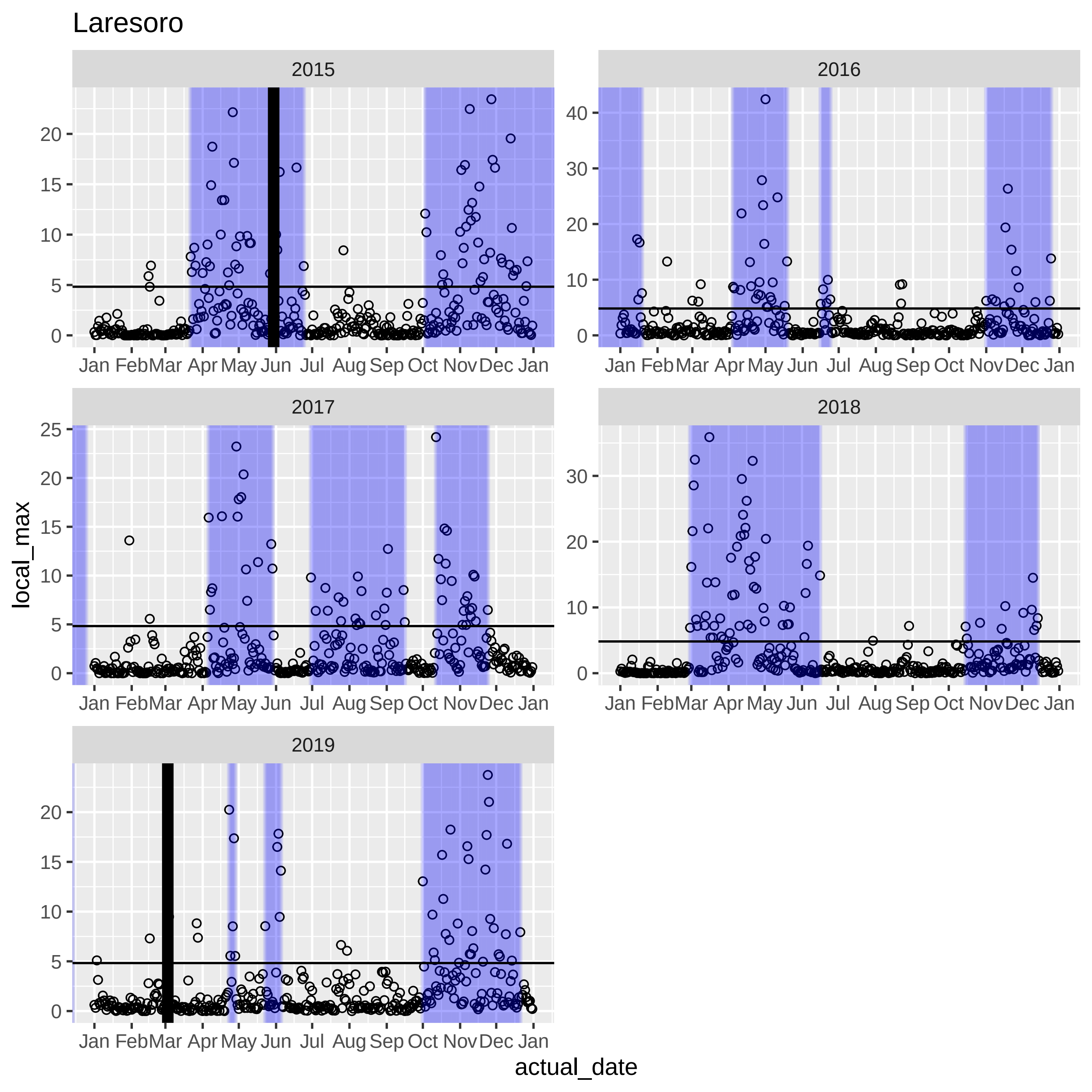

Laresoro
2015
2016
2017
2018
2019
local_max
actual_date
Jan Feb Mar Apr May Jun Jul Aug Sep Oct Nov Dec Jan
0
5
10
15
20
25
30
40

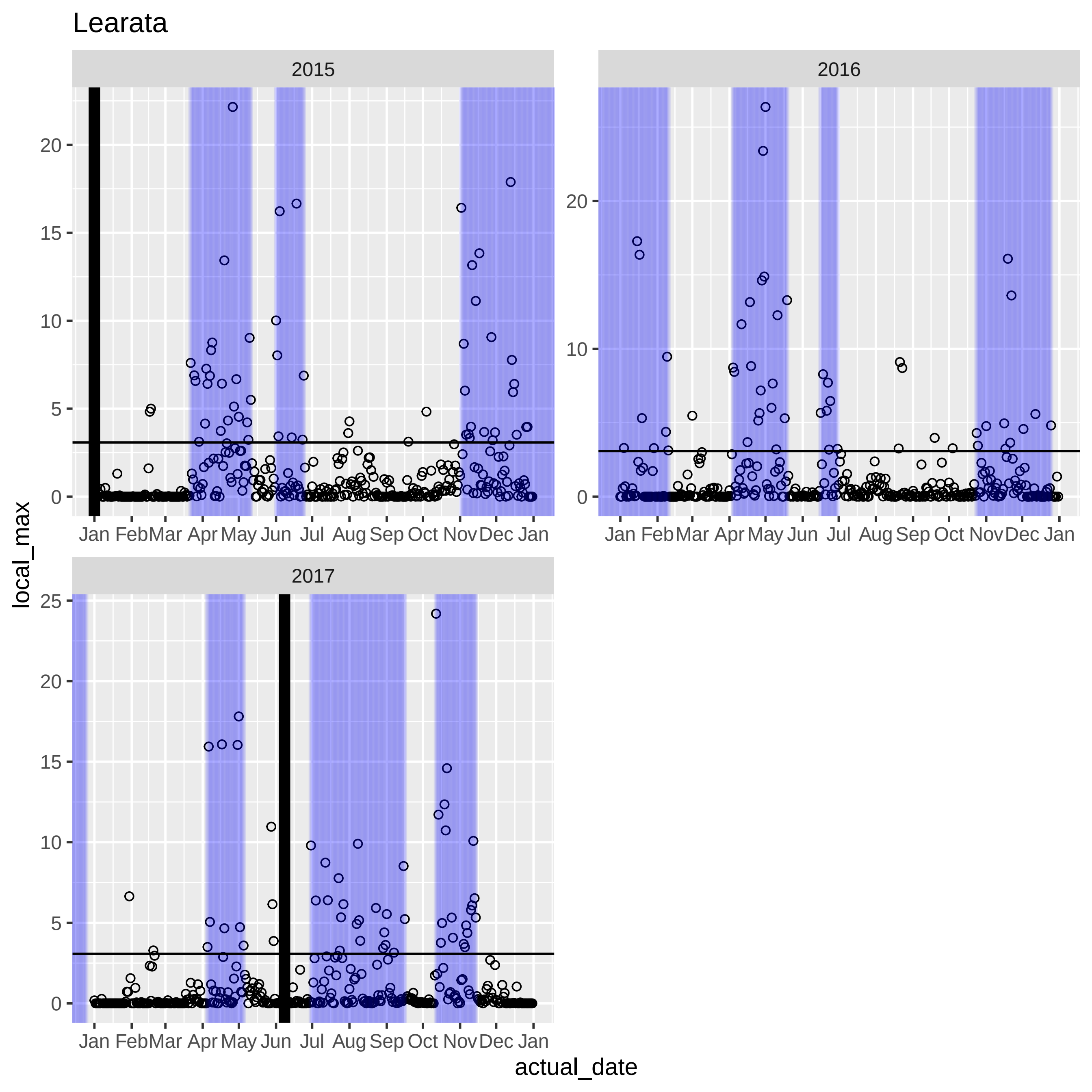

Learata
2015
2016
2017
local_max
actual_date
0
5
10
15
20
25
Jan Feb Mar Apr May Jun Jul Aug Sep Oct Nov Dec Jan

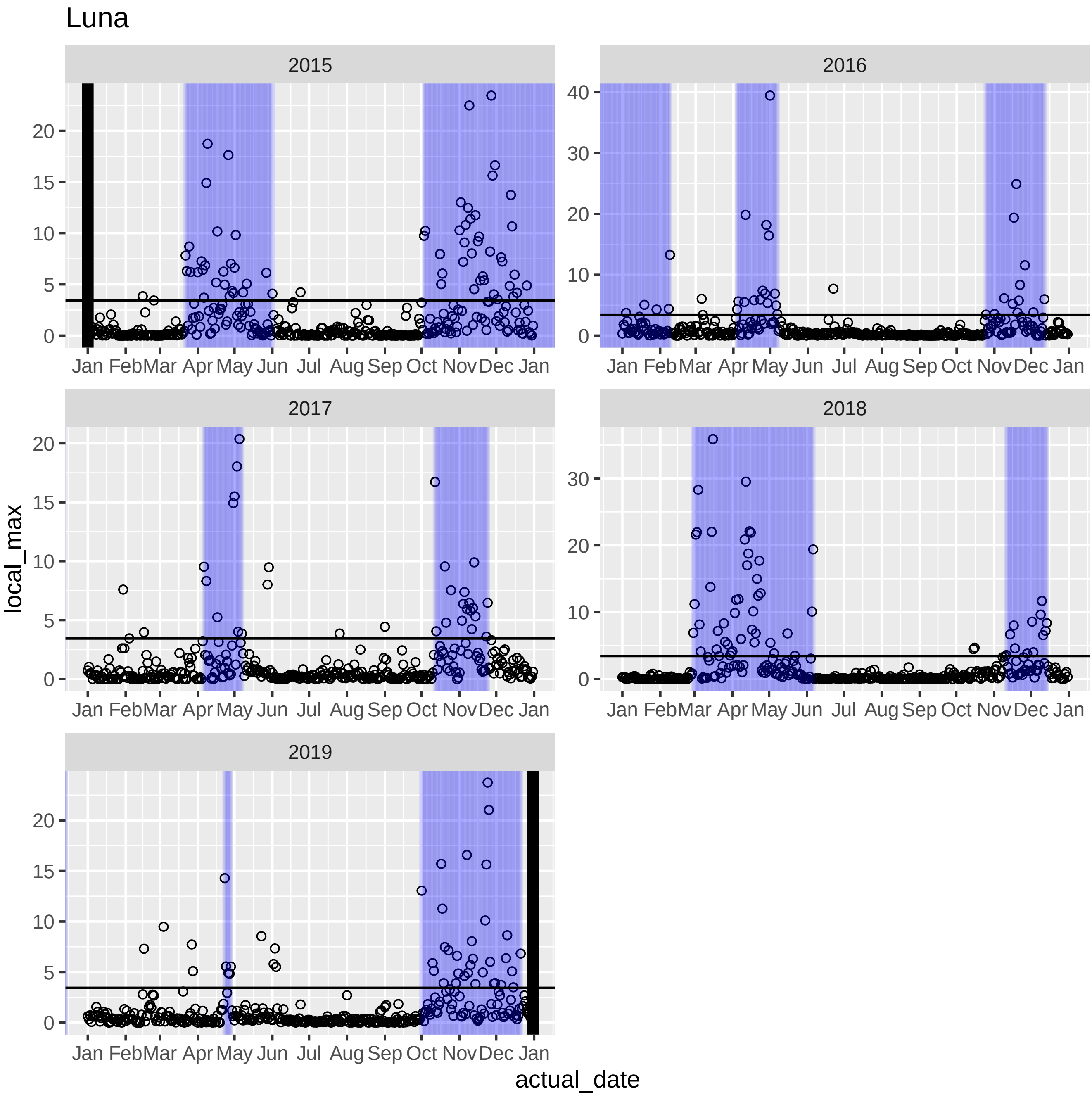

Luna
2015
2016
2017
2018
2019
local_max
actual_date
Jan Feb Mar Apr May Jun Jul Aug Sep Oct Nov Dec Jan
0 5 10 15 20
0 10 20 30 40

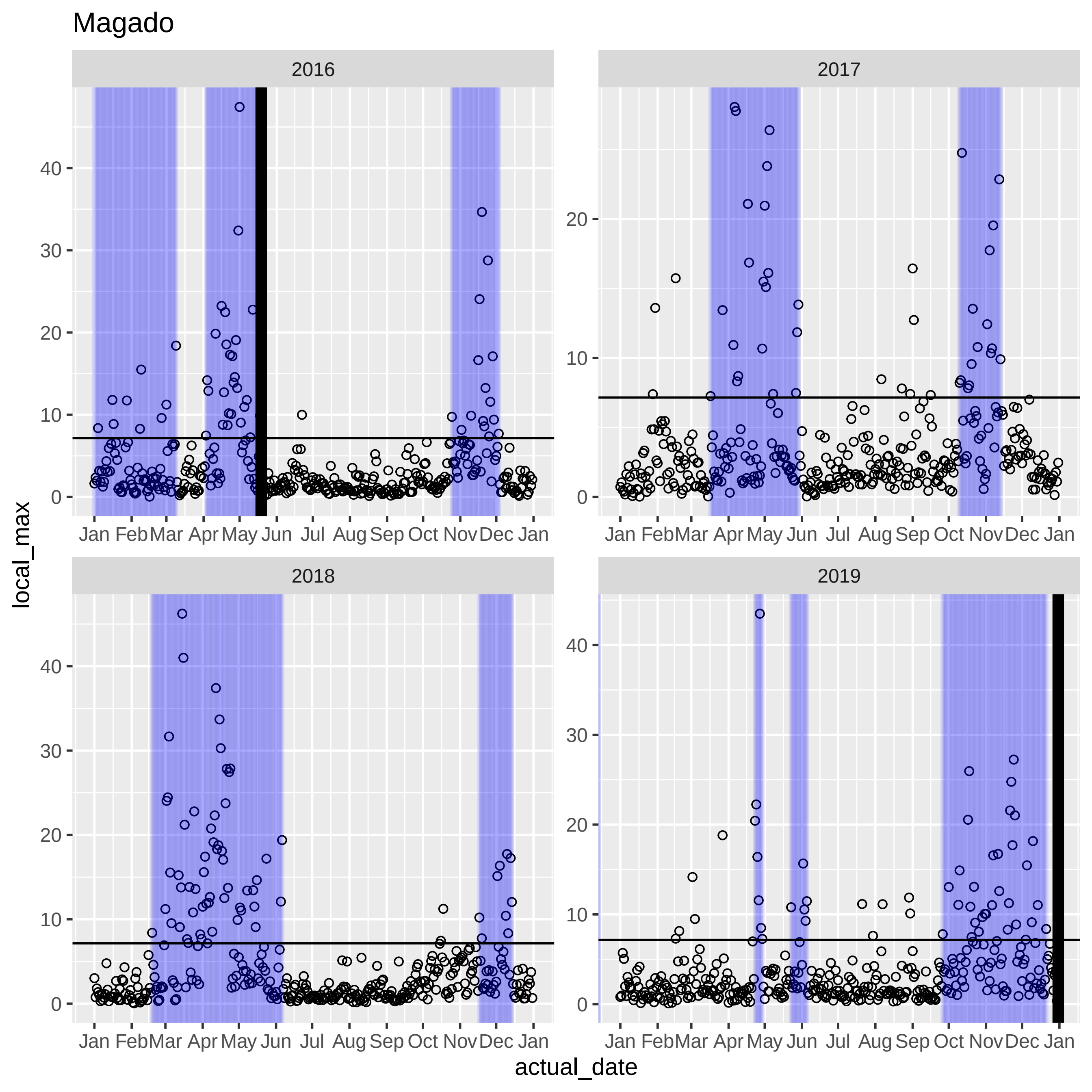
Magado
2016
2017
2018
2019
local_max
actual_date
Jan Feb Mar Apr May Jun Jul Aug Sep Oct Nov Dec Jan
0
10
20
30
40

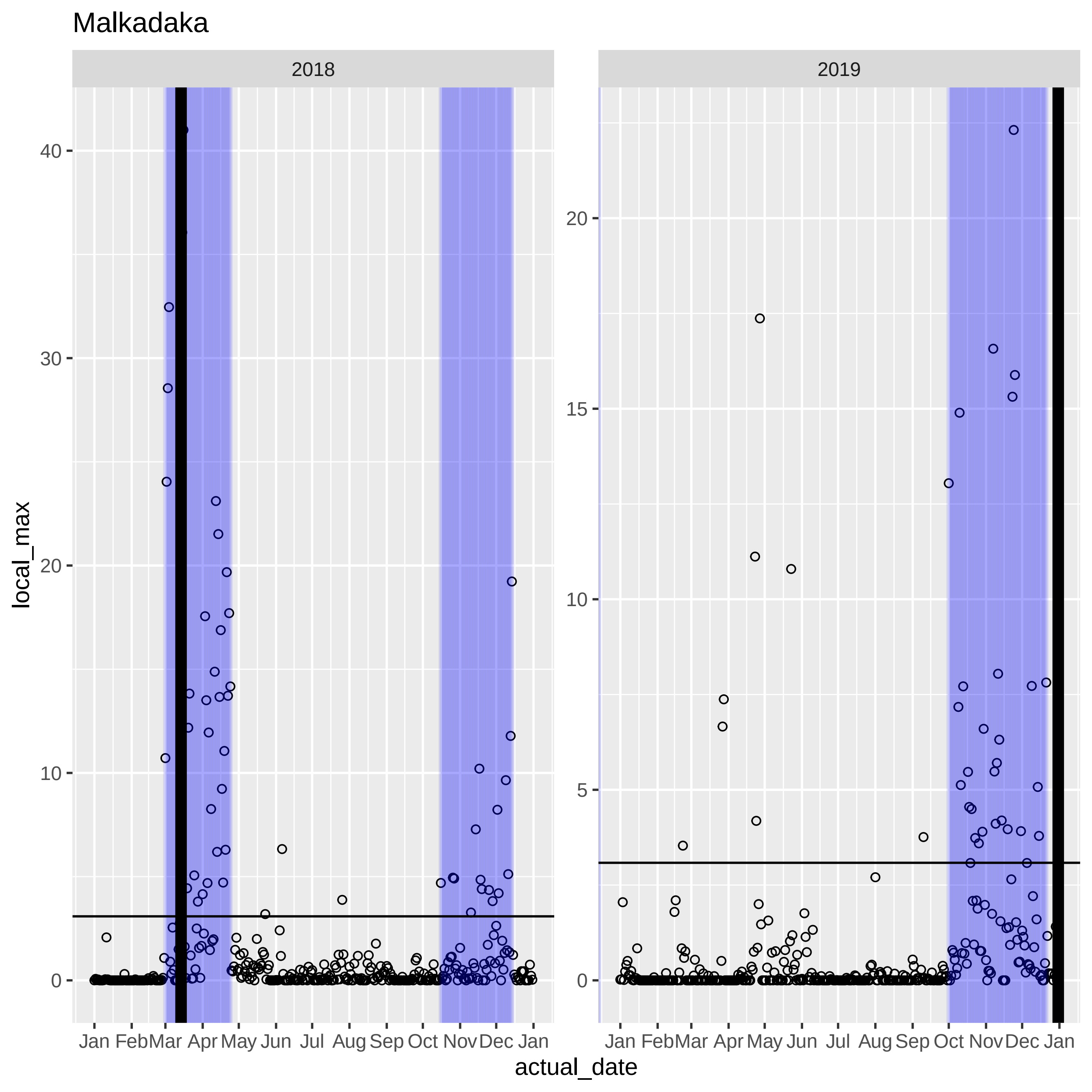

Malkadaka
2018
2019
local_max
actual_date
0
10
20
30
40
0
5
10
15
20
Jan Feb Mar Apr May Jun Jul Aug Sep Oct Nov Dec Jan
Jan Feb Mar Apr May Jun Jul Aug Sep Oct Nov Dec Jan

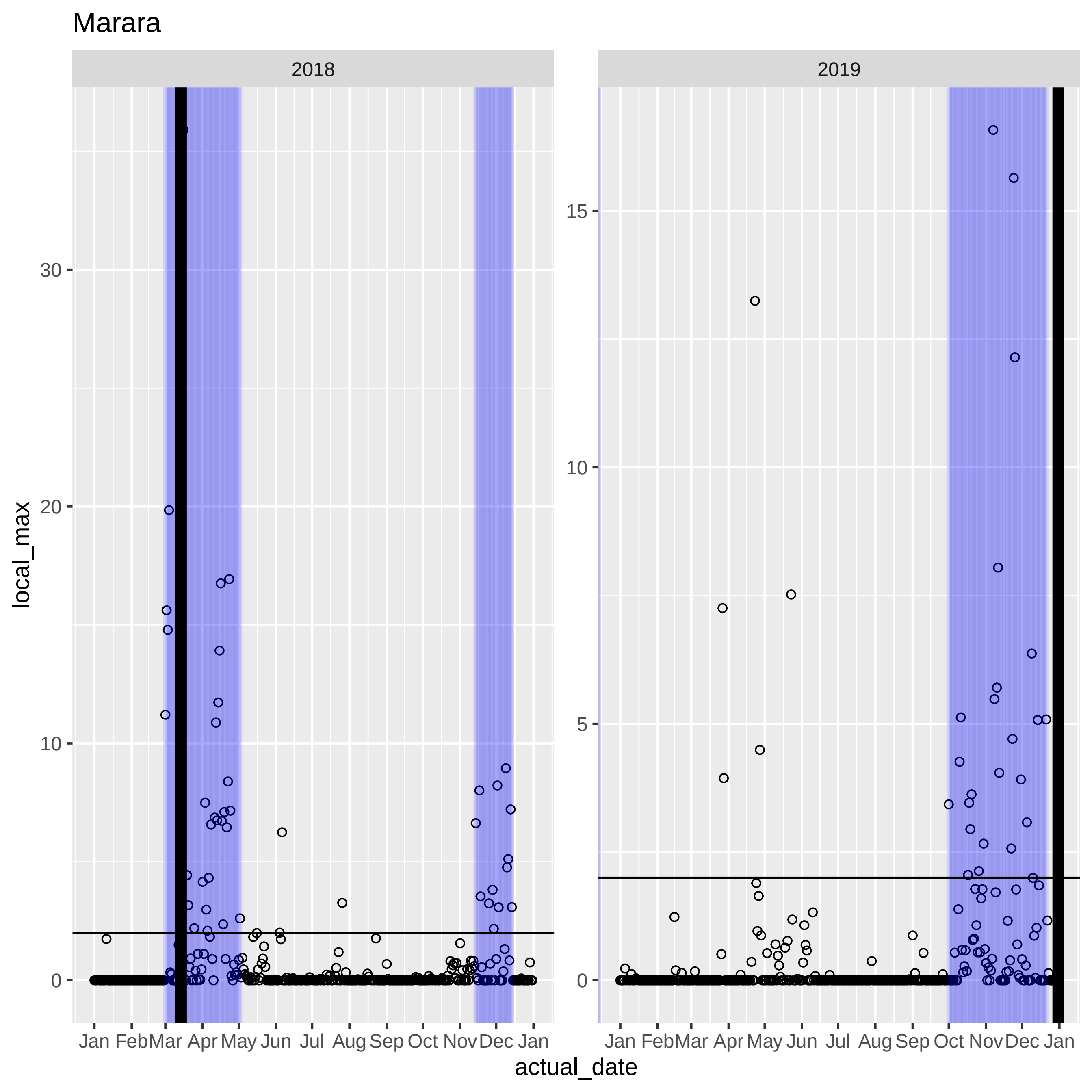

Marara
2018
2019
local_max
actual_date
0
10
20
30
5
15
Jan Feb Mar Apr May Jun Jul Aug Sep Oct Nov Dec Jan

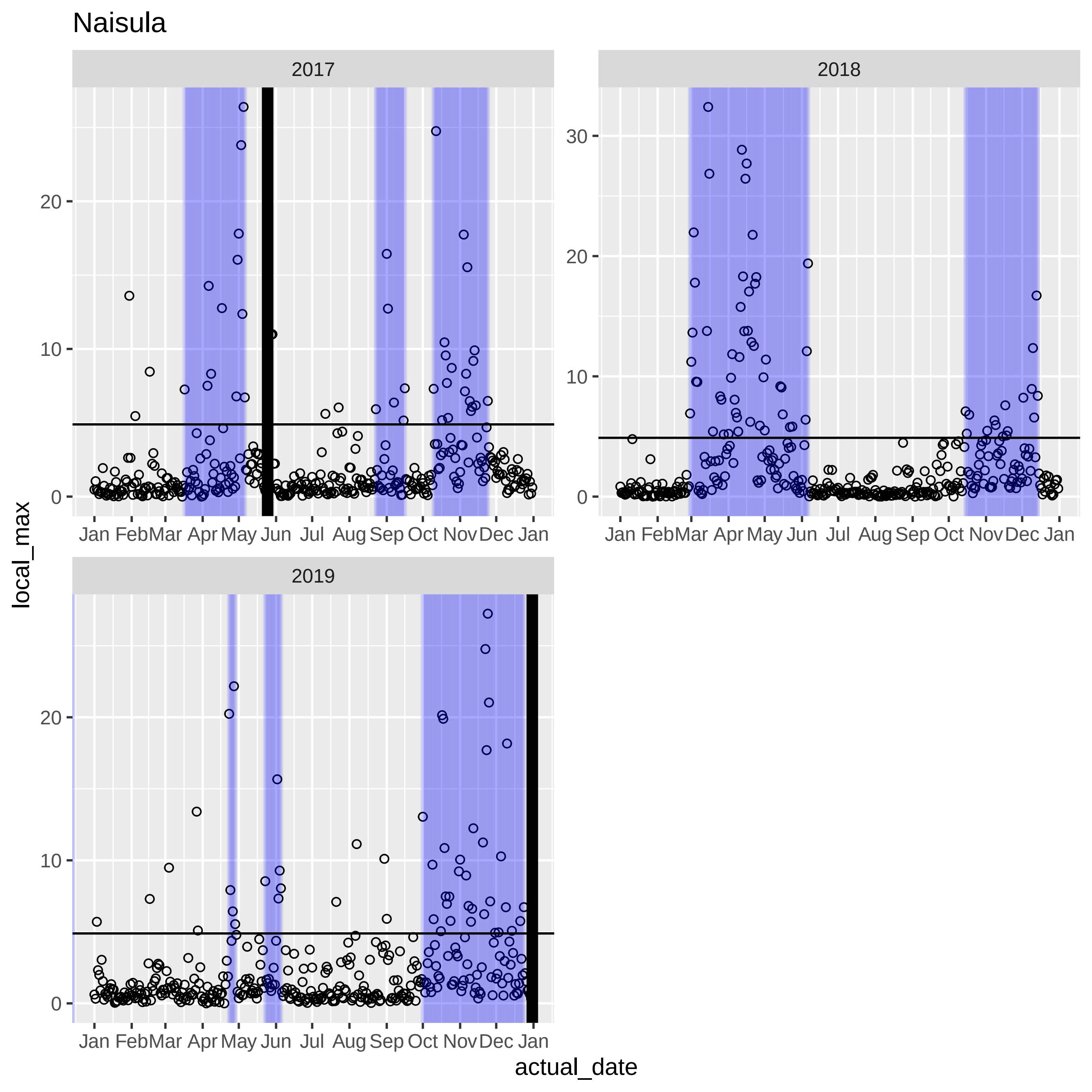

Naisula
2017
2018
2019
local_max
actual_date
0
10
20
30
Jan
Feb
Mar
Apr
May
Jun
Jul
Aug
Sep
Oct
Nov
Dec

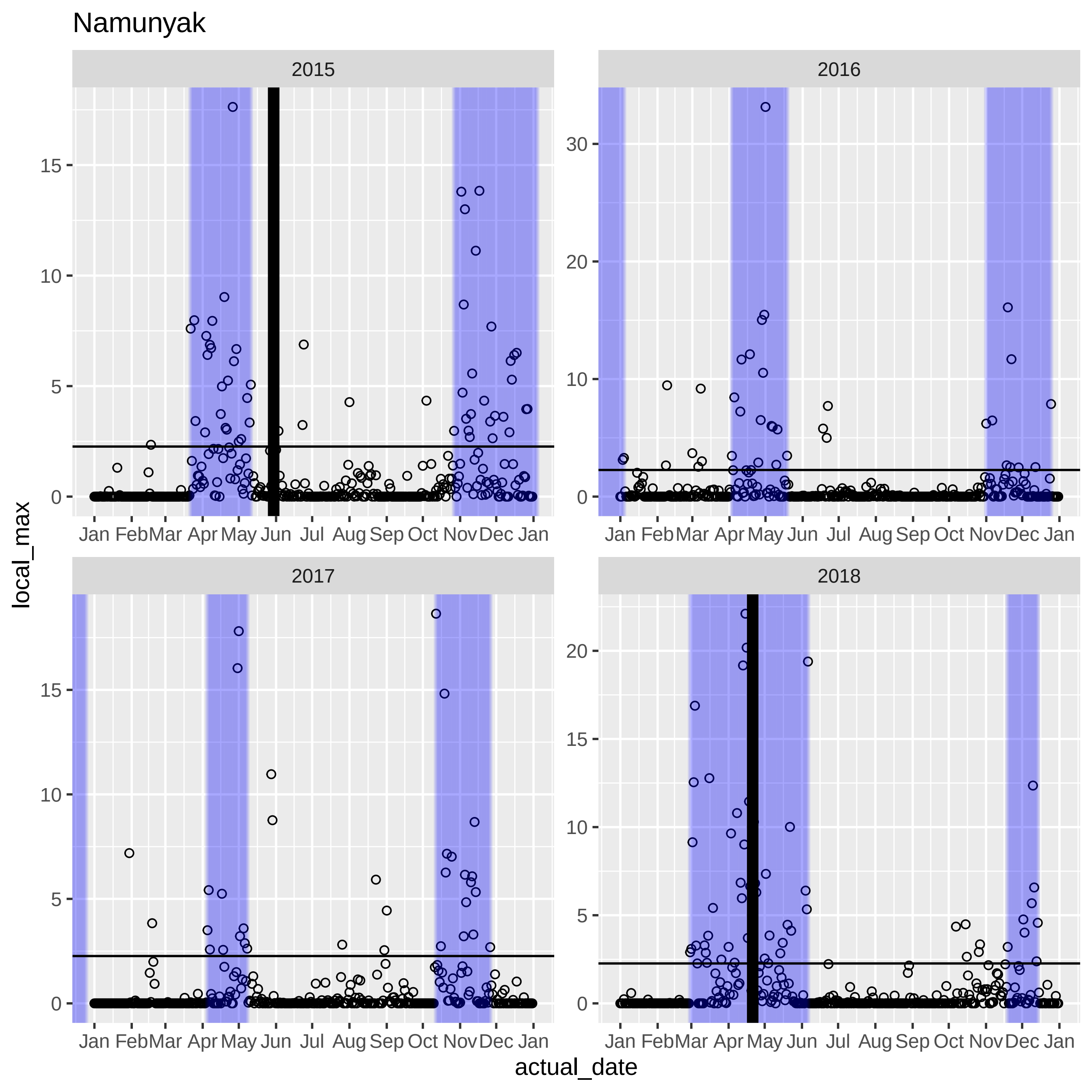
Namunyak
2015
2016
2017
2018
local_max
actual_date
Jan Feb Mar Apr May Jun Jul Aug Sep Oct Nov Dec Jan
0
5
10
15
20
30

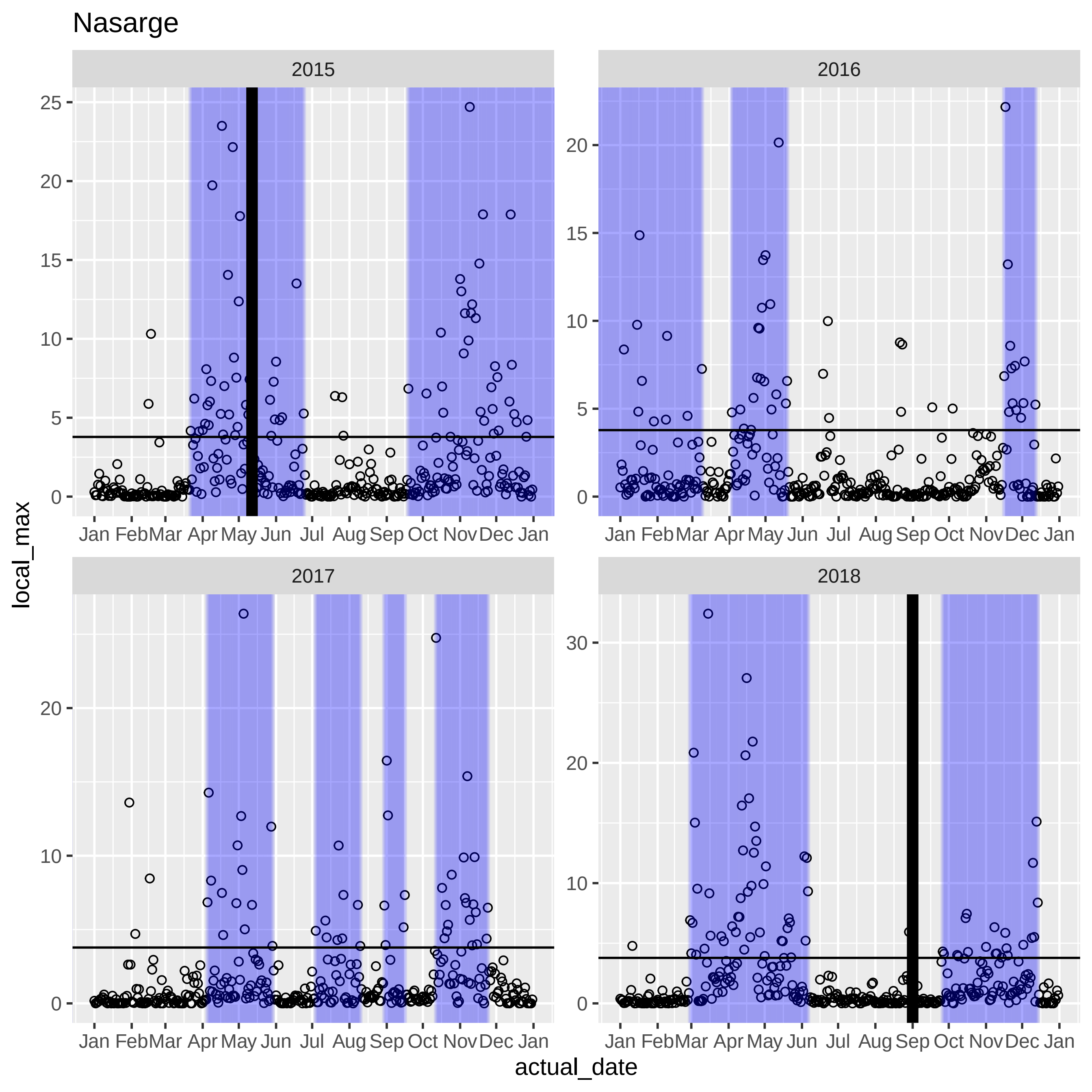

Nasarge
2015
2016
2017
2018
local_max
actual_date
0
5
10
15
20
25
30
Jan Feb Mar Apr May Jun Jul Aug Sep Oct Nov Dec Jan

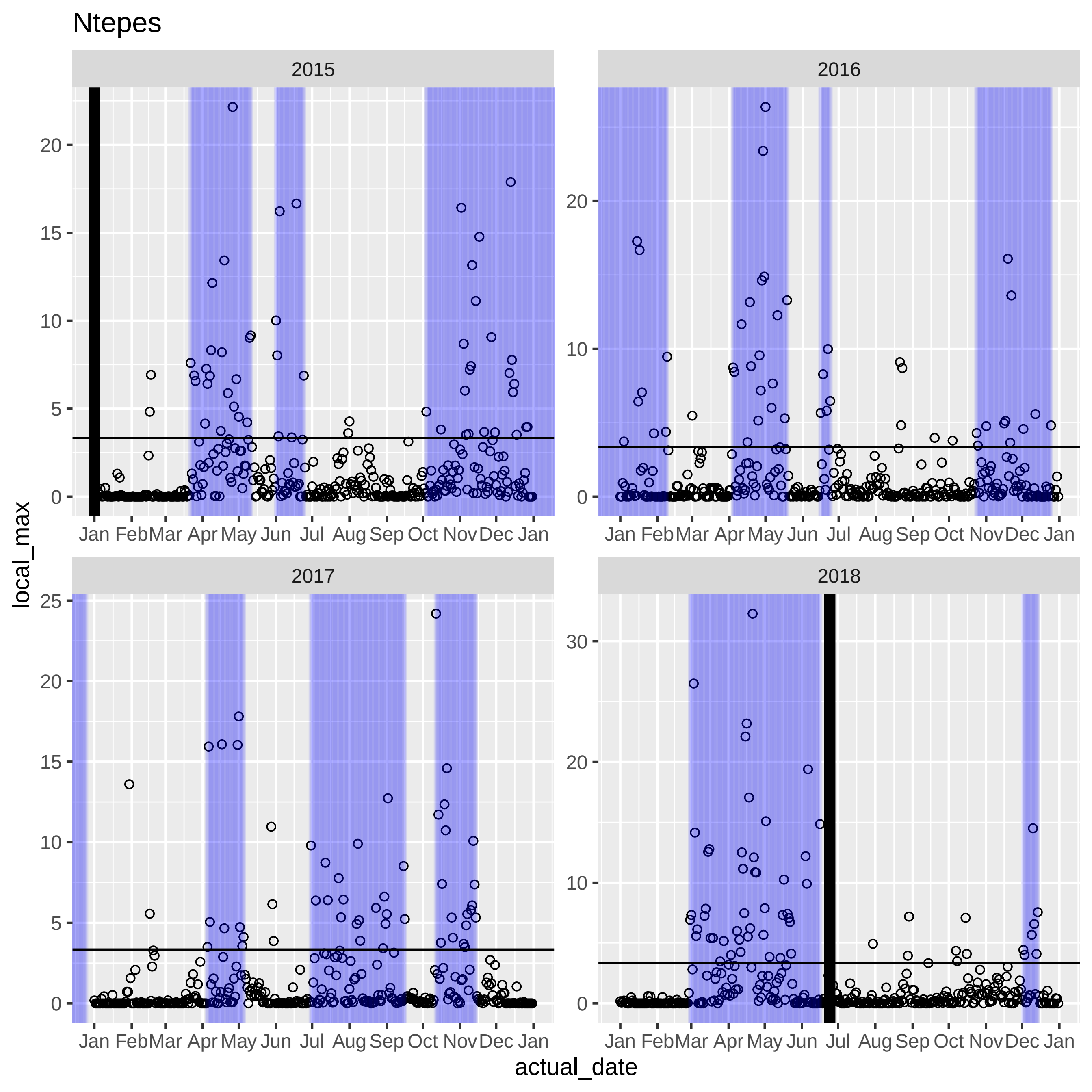

Ntepes
2015
2016
2017
2018
local_max
actual_date
Jan Feb Mar Apr May Jun Jul Aug Sep Oct Nov Dec Jan

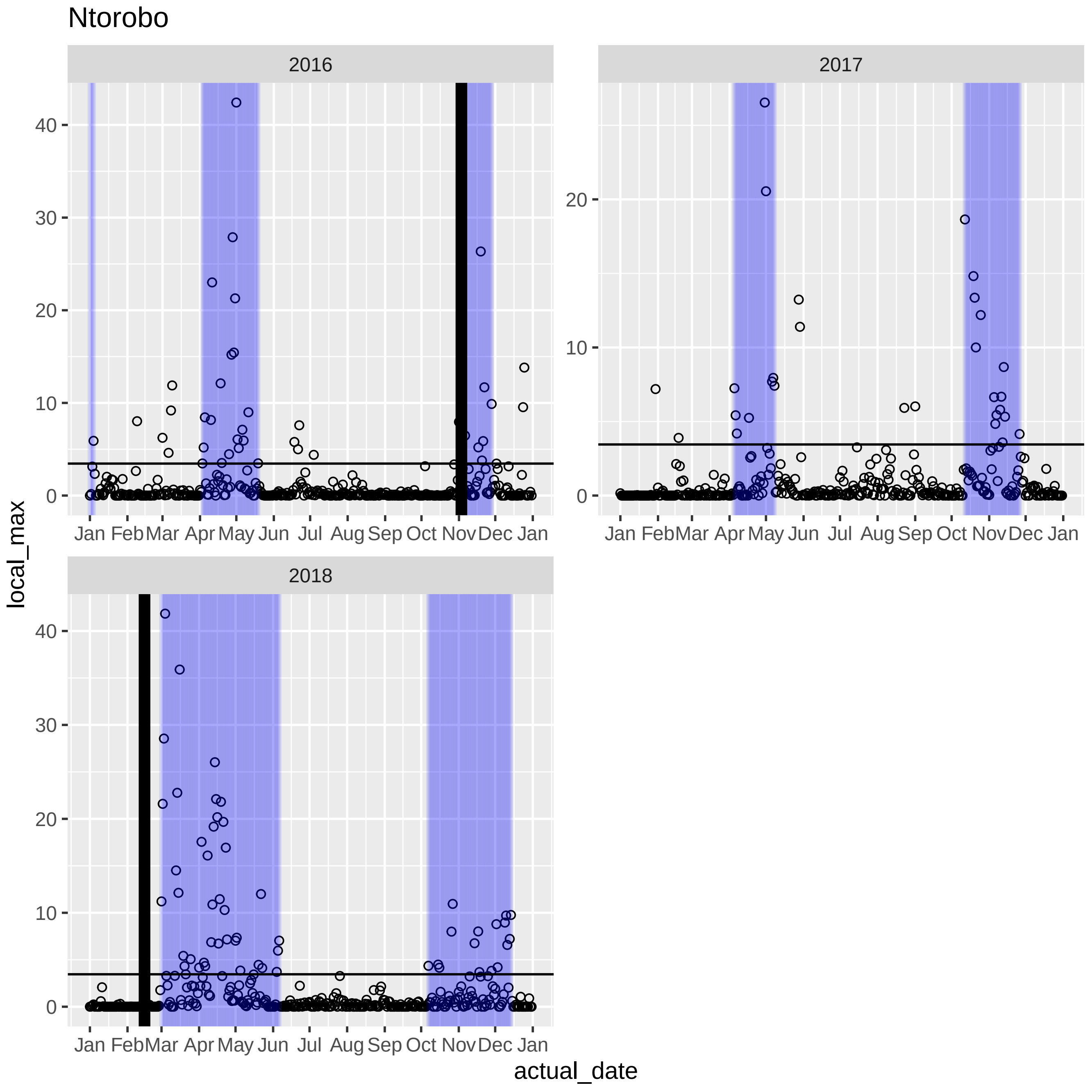

Ntorobo
2016
2017
2018
local_max
actual_date
0
10
20
30
40
Jan
Feb
Mar
Apr
May
Jun
Jul
Aug
Sep
Oct
Nov
Dec

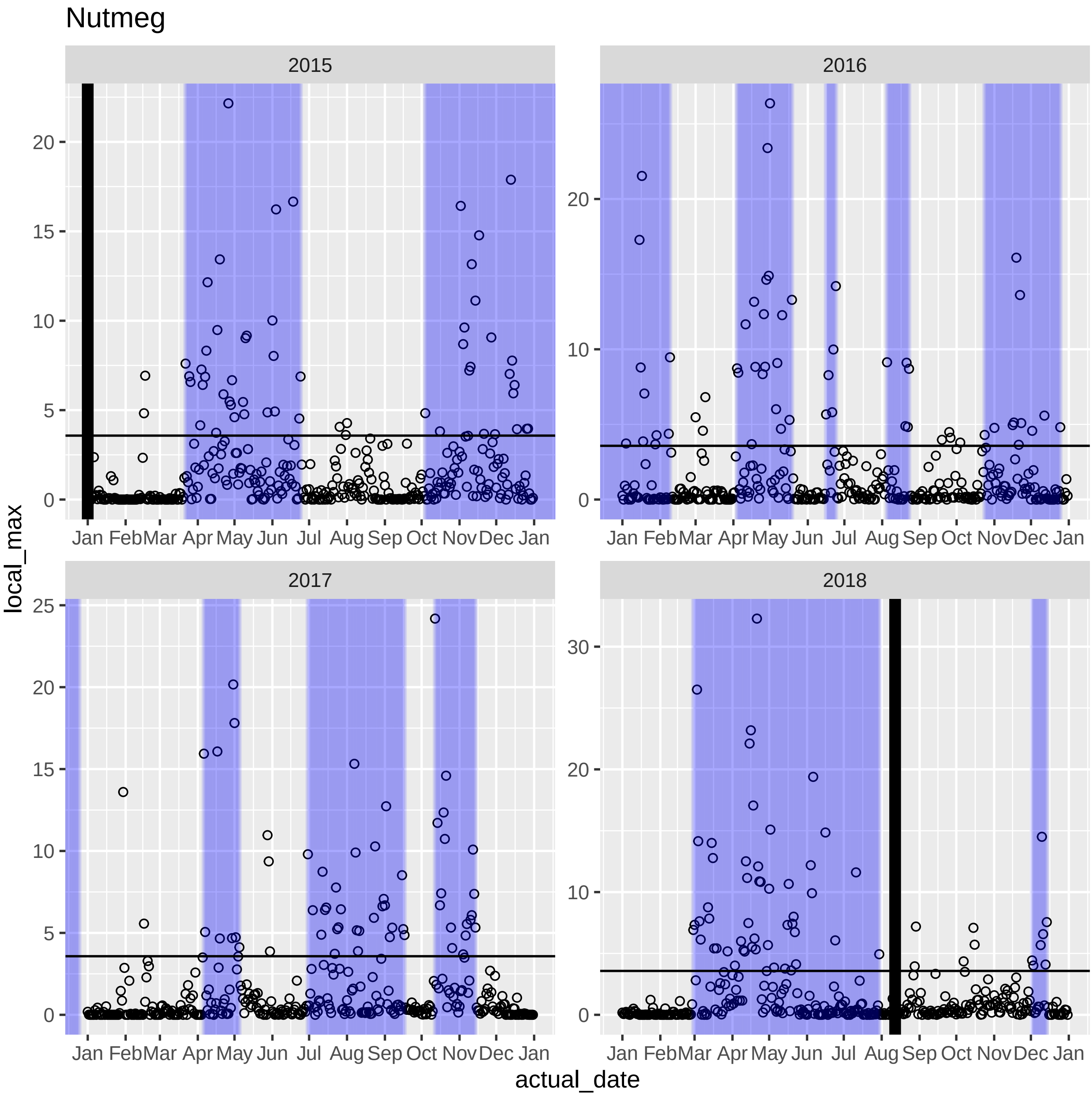

Nutmeg
2015
2016
2017
2018
local_max
actual_date
Jan Feb Mar Apr May Jun Jul Aug Sep Oct Nov Dec Jan

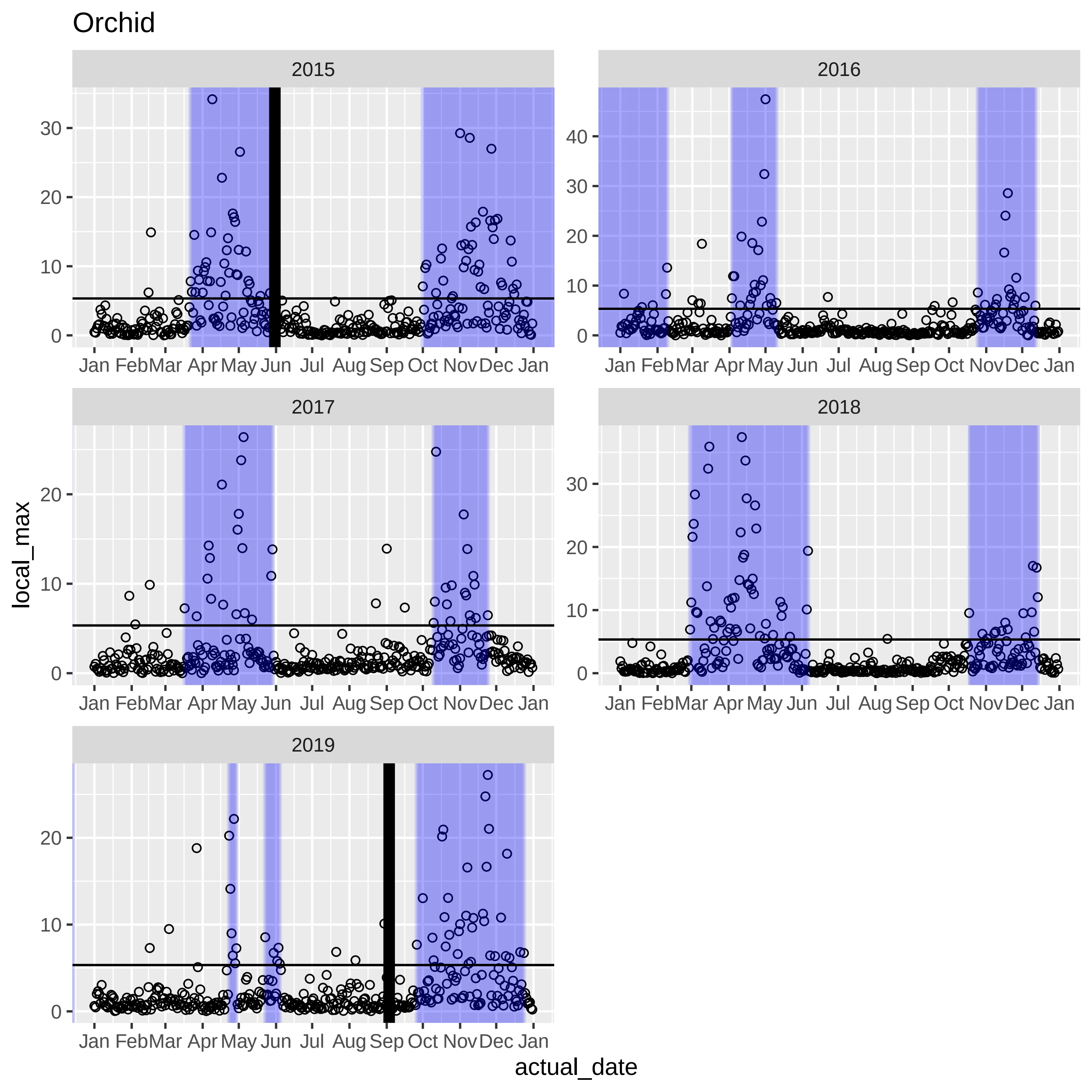
Orchid
2015
2016
2017
2018
2019
local_max
actual_date
Jan
Feb
Mar
Apr
May
Jun
Jul
Aug
Sep
Oct
Nov
Dec
0
10
20
30
40

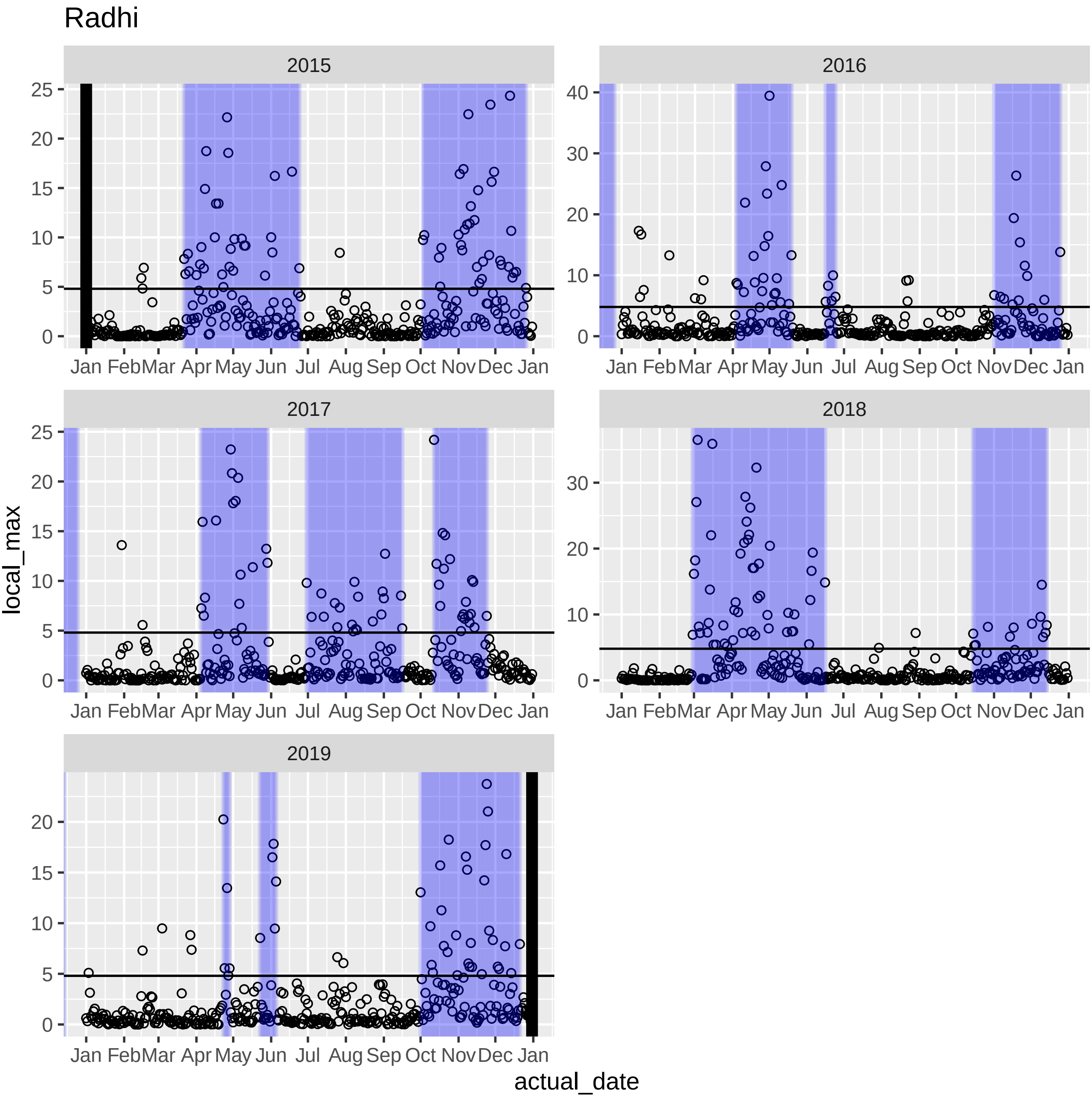
Radhi
2015
2016
2017
2018
2019
local_max
actual_date
0
5
10
15
20
25
30
40
Jan
Feb
Mar
Apr
May
Jun
Jul
Aug
Sep
Oct
Nov
Dec

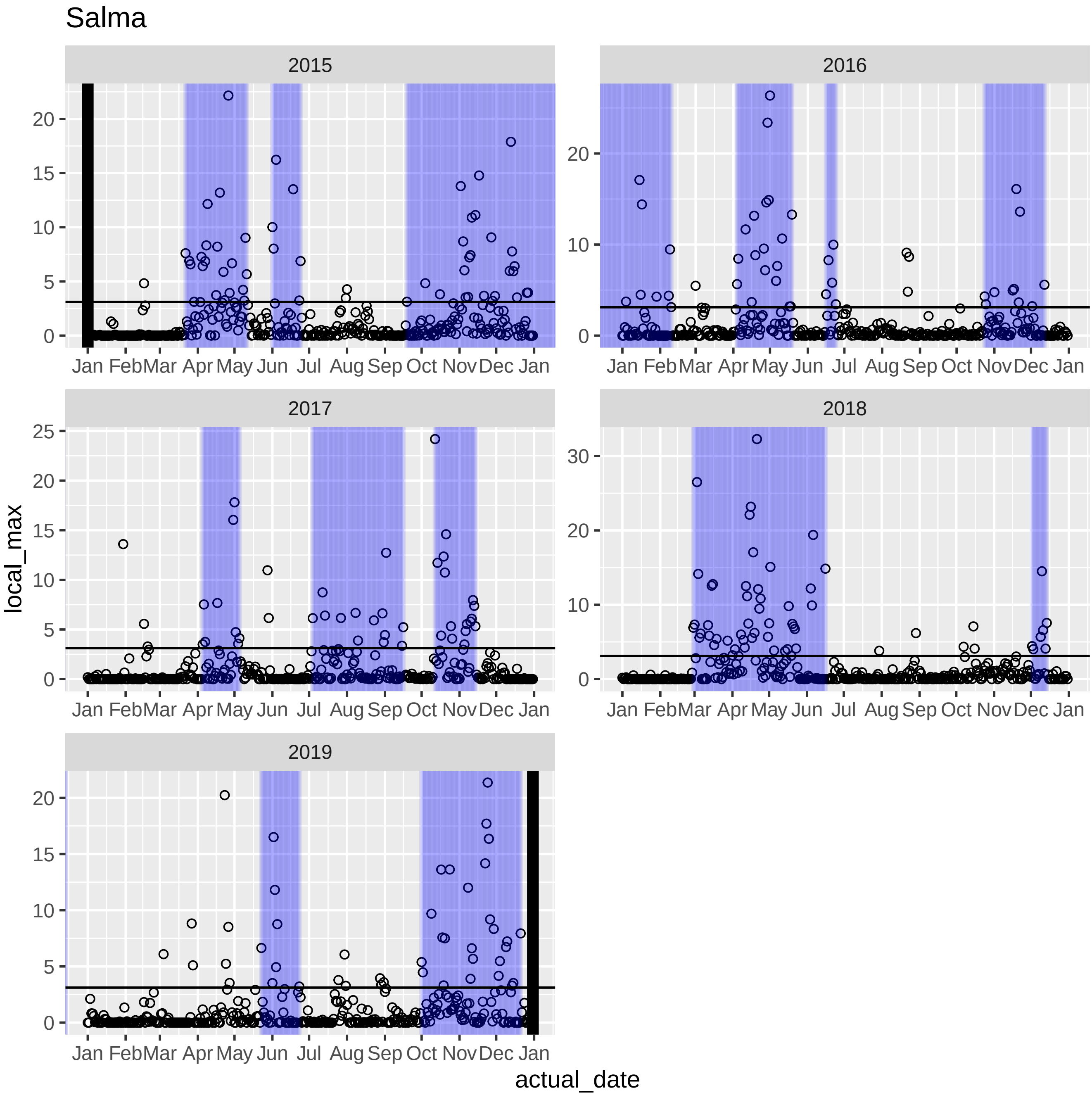
Salma
2015
2016
2017
2018
2019
local_max
actual_date
Jan Feb Mar Apr May Jun Jul Aug Sep Oct Nov Dec Jan
0
5
10
15
20
25
30

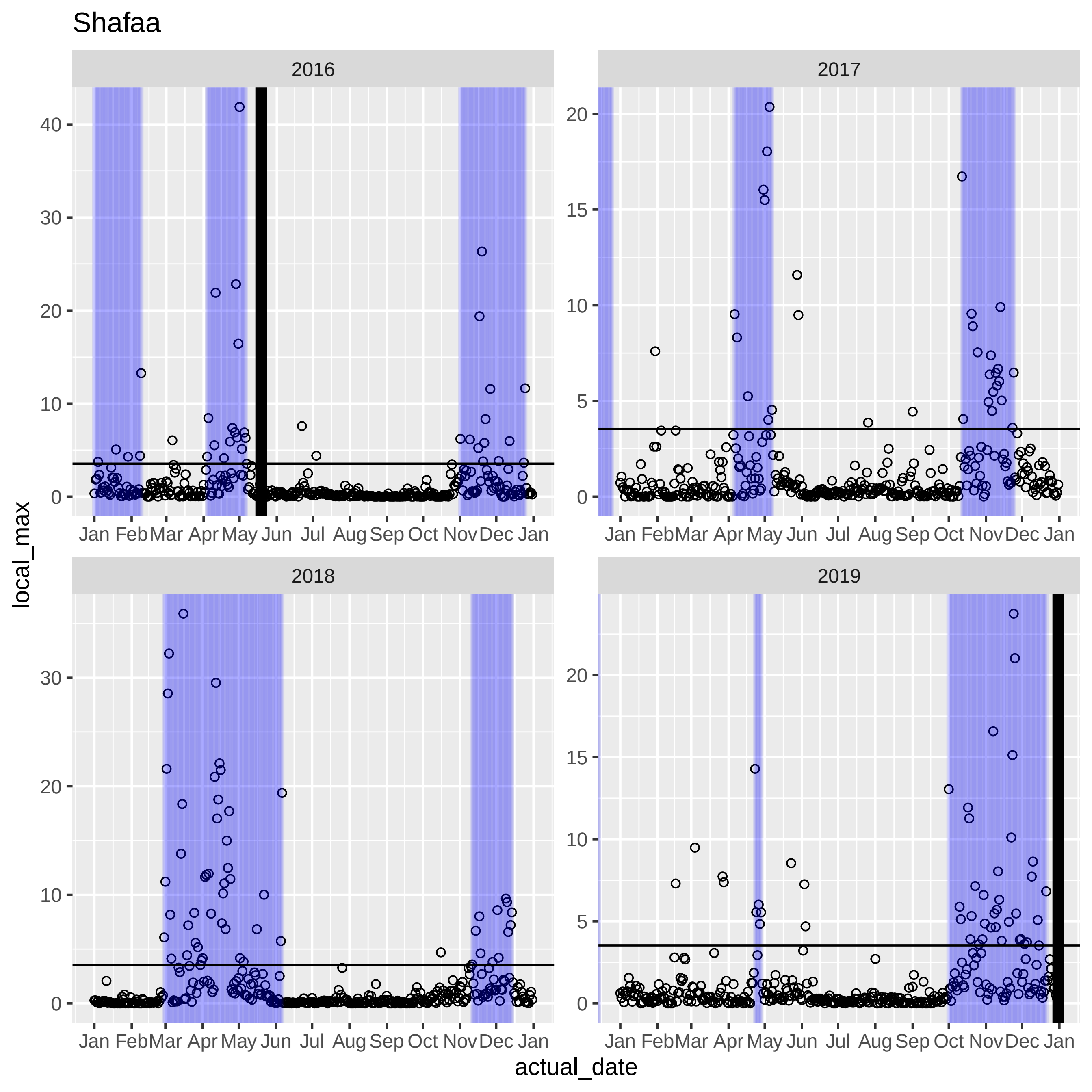

Shafaa
2016
2017
2018
2019
local_max
actual_date
Jan
Feb
Mar
Apr
May
Jun
Jul
Aug
Sep
Oct
Nov
Dec

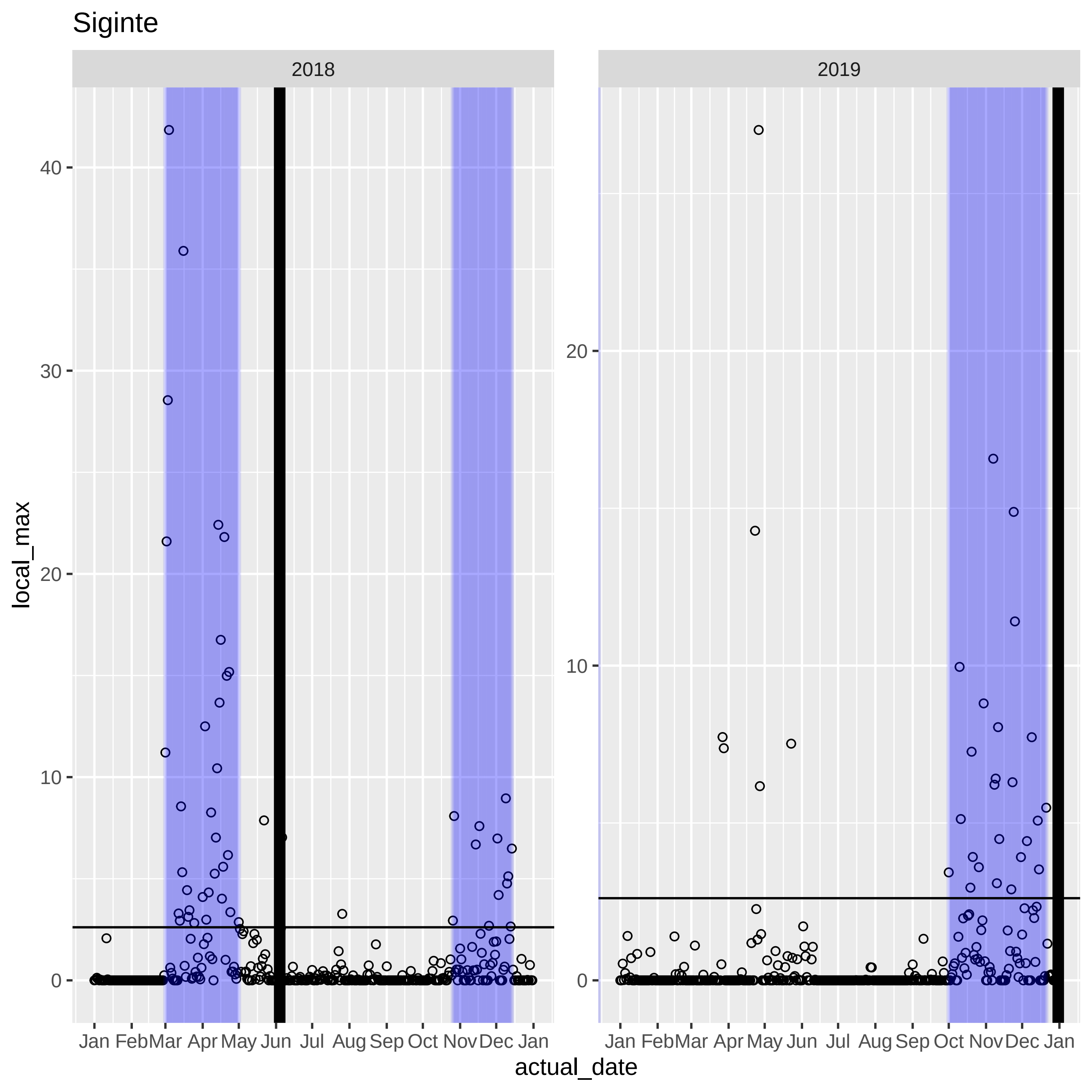
Siginte
2018
2019
local_max
actual_date
0
10
20
30
40
Jan Feb Mar Apr May Jun Jul Aug Sep Oct Nov Dec Jan

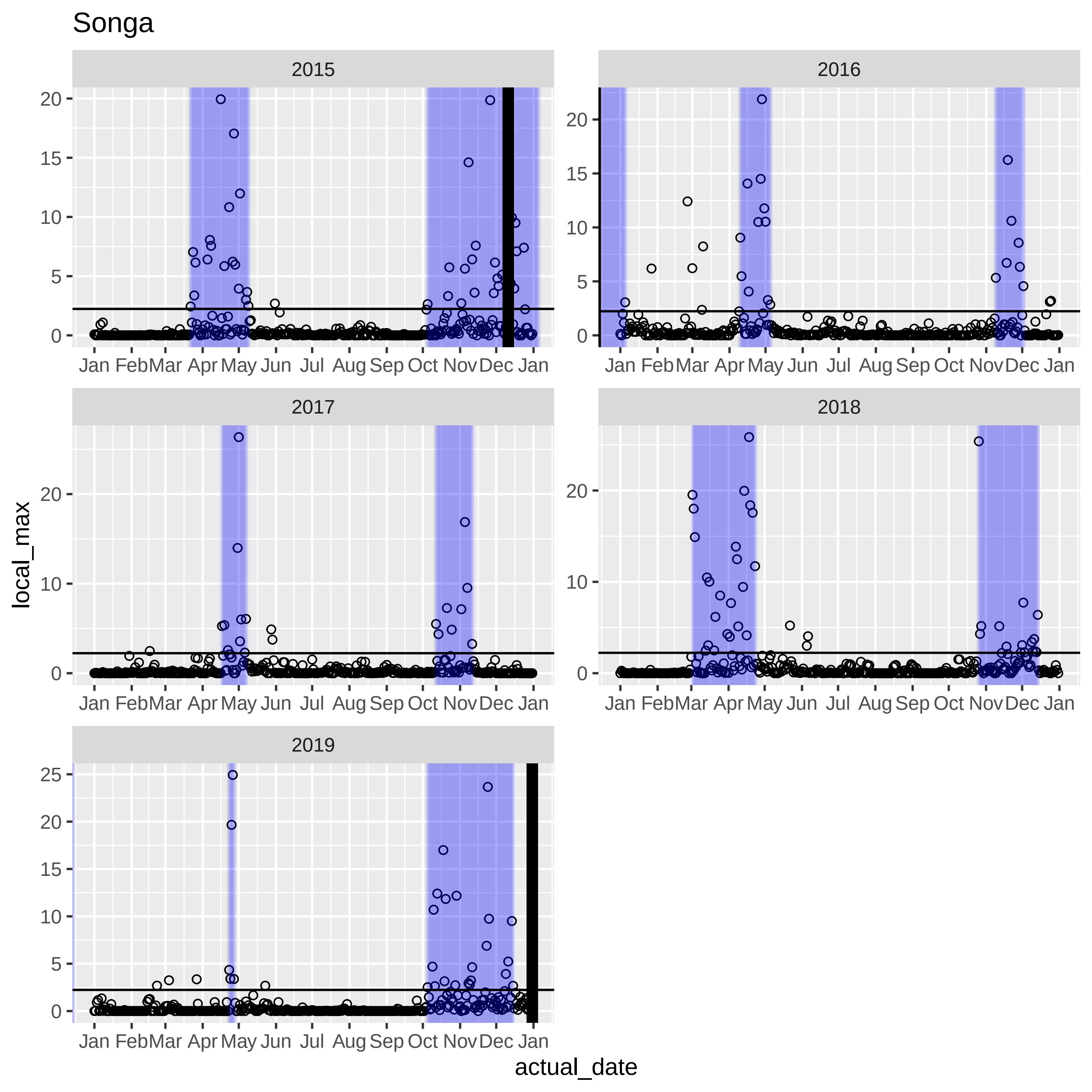

Songa
2015
2016
2017
2018
2019
local_max
actual_date
Jan Feb Mar Apr May Jun Jul Aug Sep Oct Nov Dec Jan
0
5
10
15
20
25

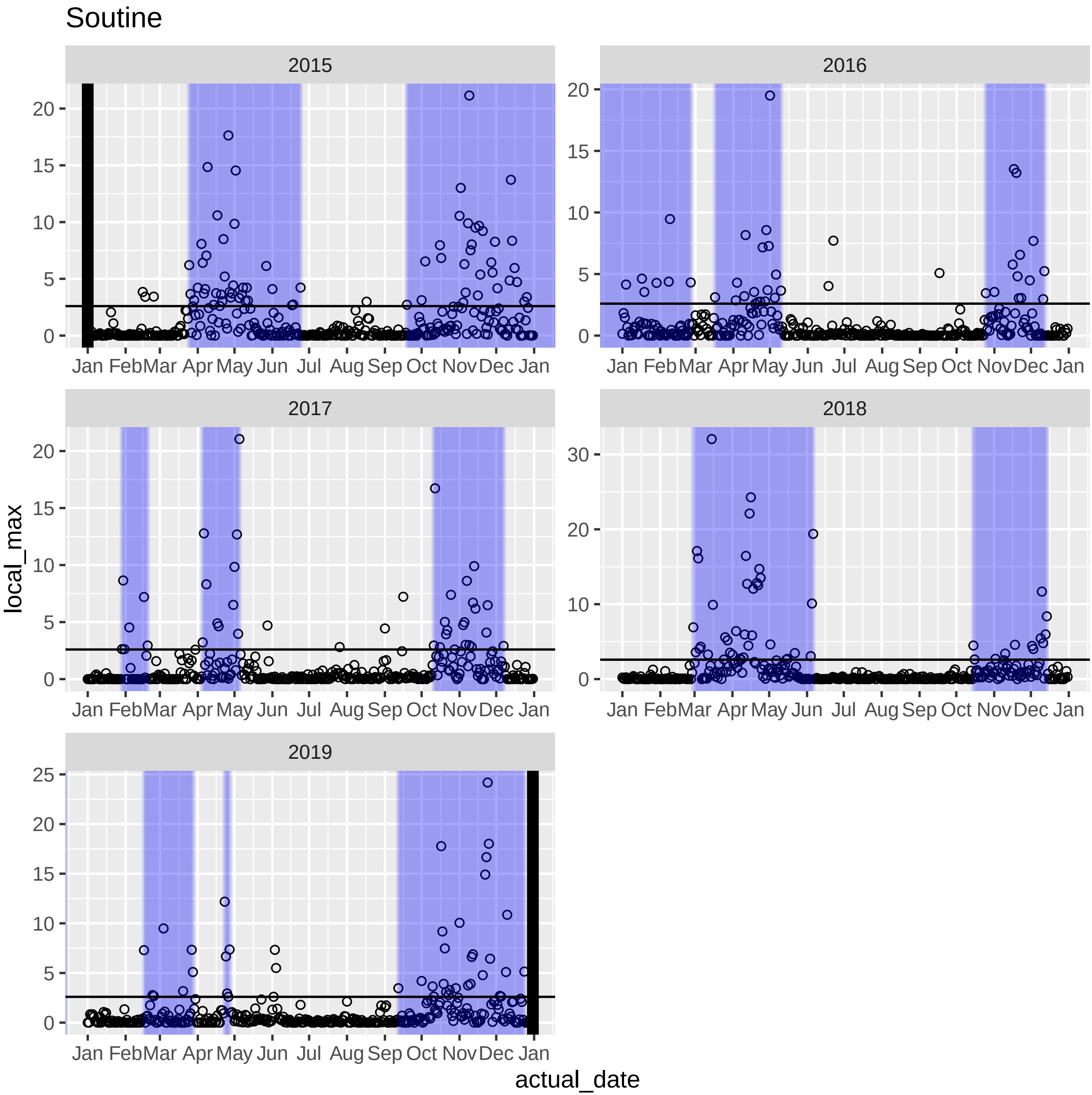

Soutine
2015
2016
2017
2018
2019
local_max
actual_date
Jan
Feb
Mar
Apr
May
Jun
Jul
Aug
Sep
Oct
Nov
Dec

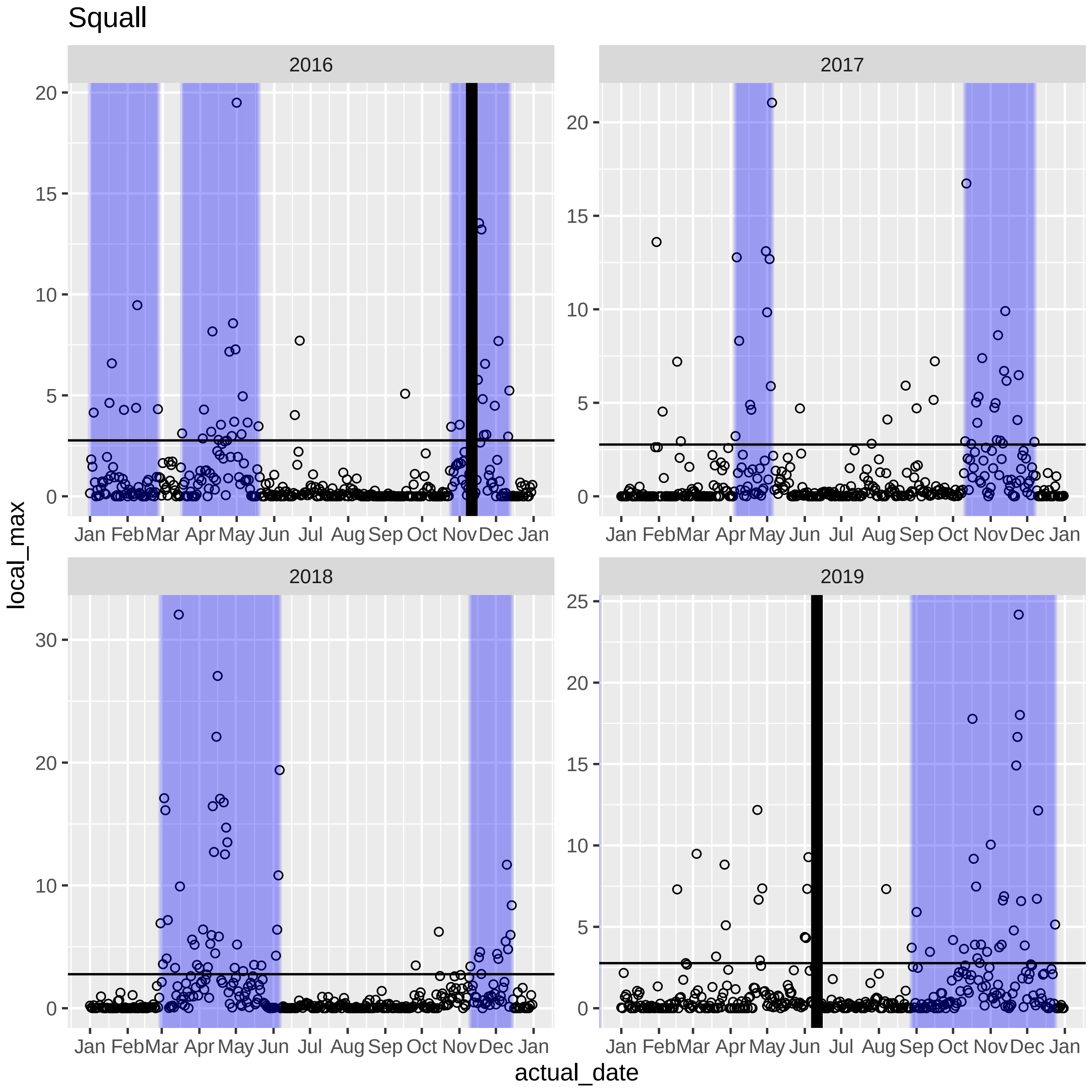

Squall
2016
2017
2018
2019
local_max
actual_date
Jan Feb Mar Apr May Jun Jul Aug Sep Oct Nov Dec Jan
0
5
10
15
20
25
30

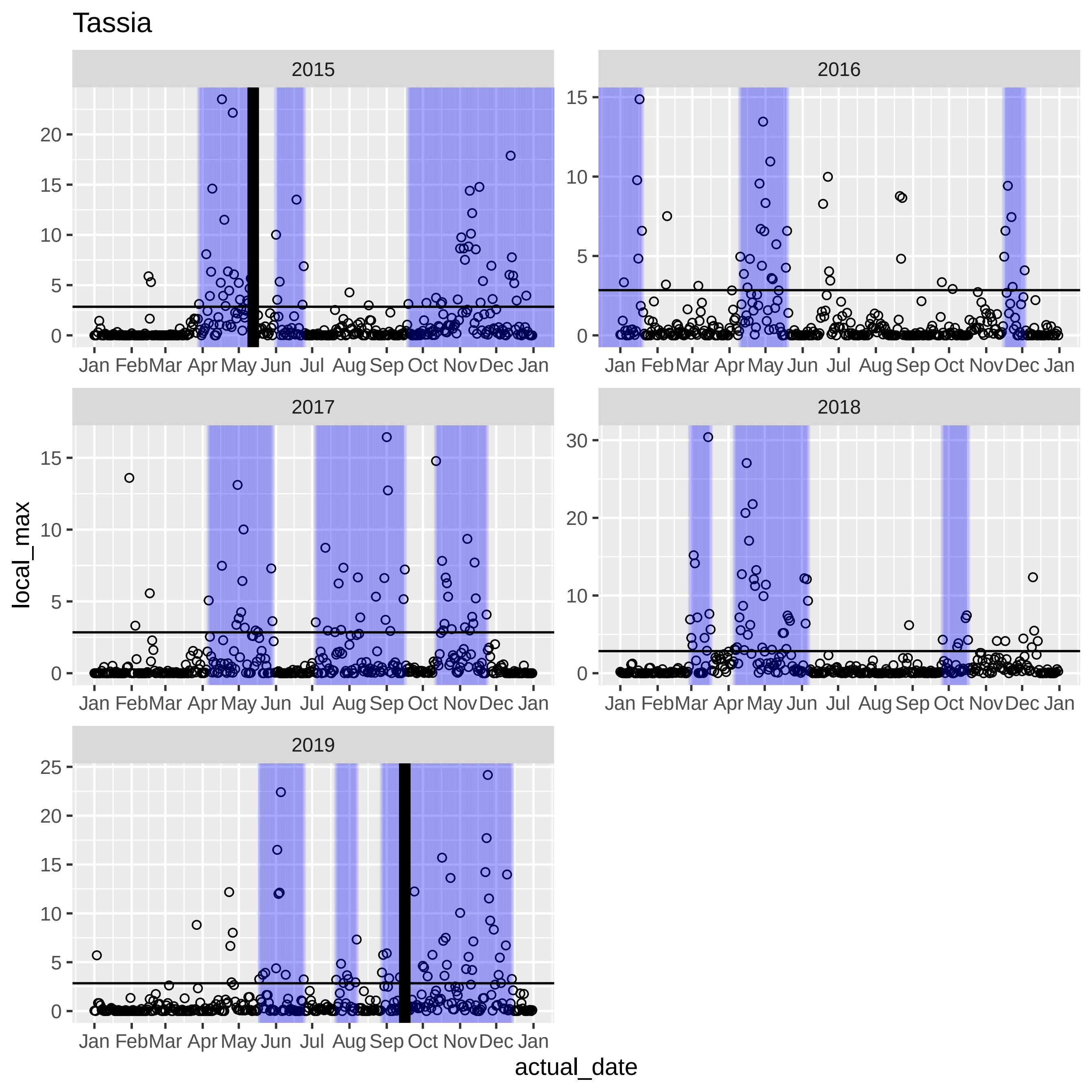

Tassia
2015
2016
2017
2018
2019
local_max
actual_date
Jan
Feb
Mar
Apr
May
Jun
Jul
Aug
Sep
Oct
Nov
Dec

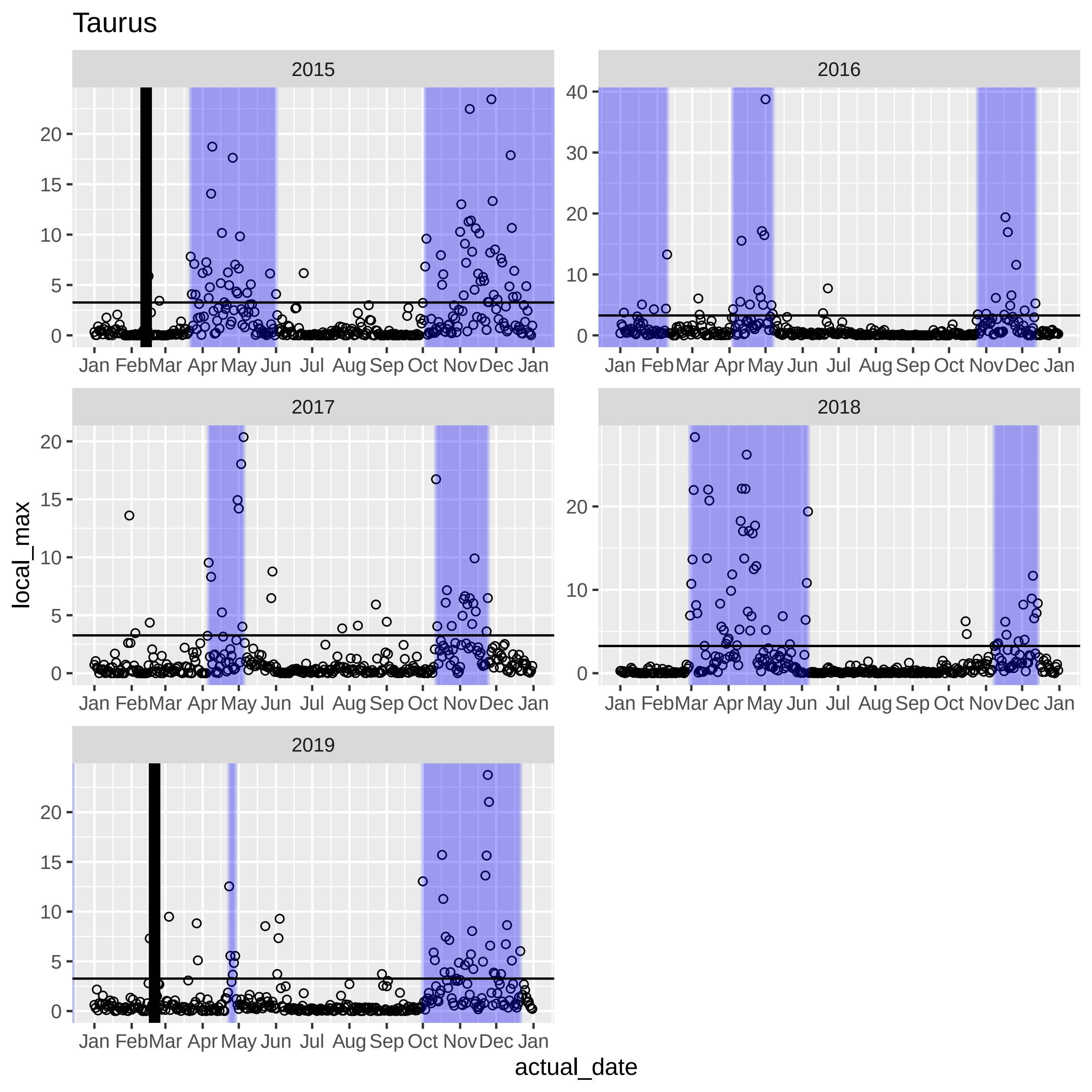

Taurus
2015
2016
2017
2018
2019
local_max
actual_date
Jan Feb Mar Apr May Jun Jul Aug Sep Oct Nov Dec Jan

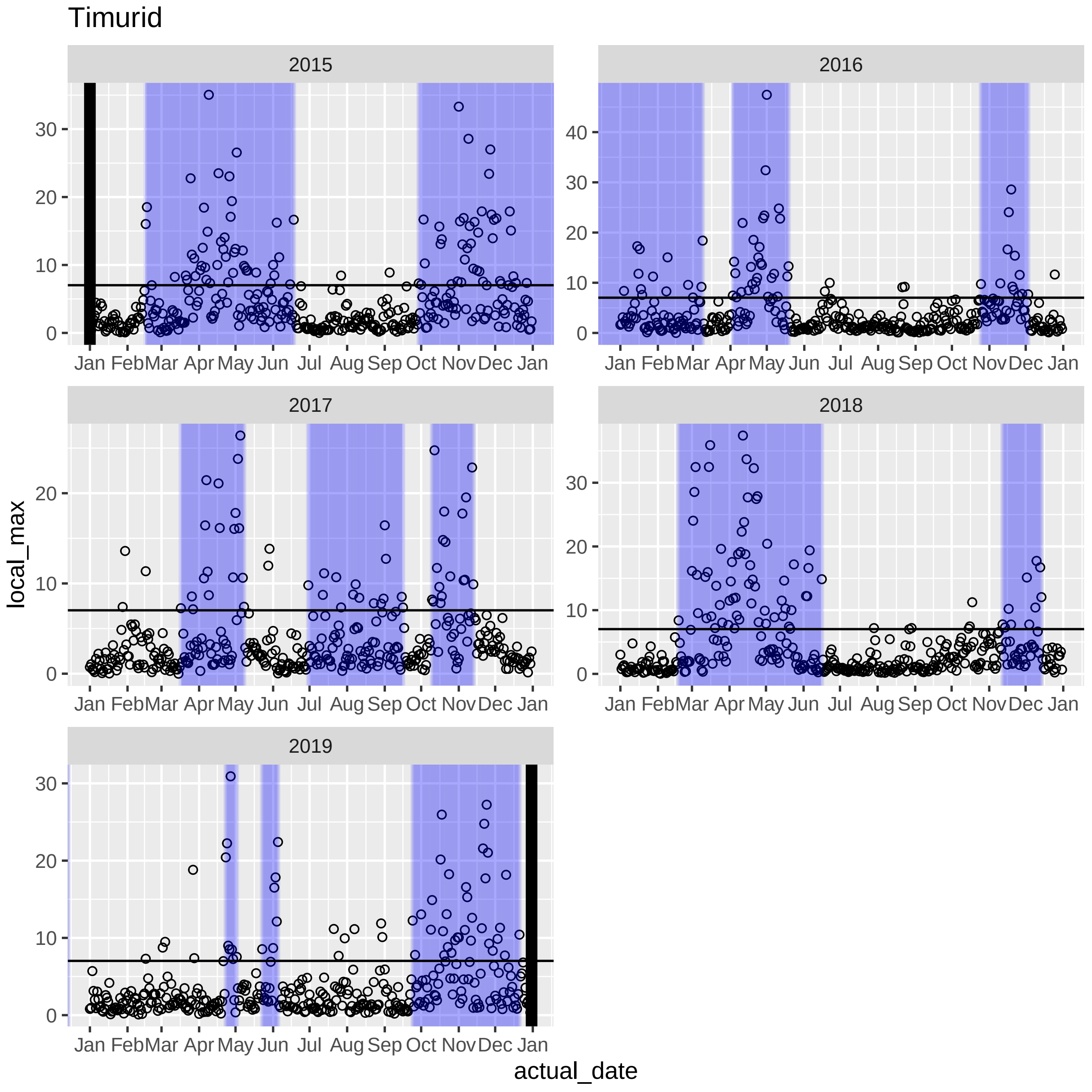
Timurid
2015
2016
2017
2018
2019
local_max
actual_date
0
10
20
30
40
Jan Feb Mar Apr May Jun Jul Aug Sep Oct Nov Dec Jan

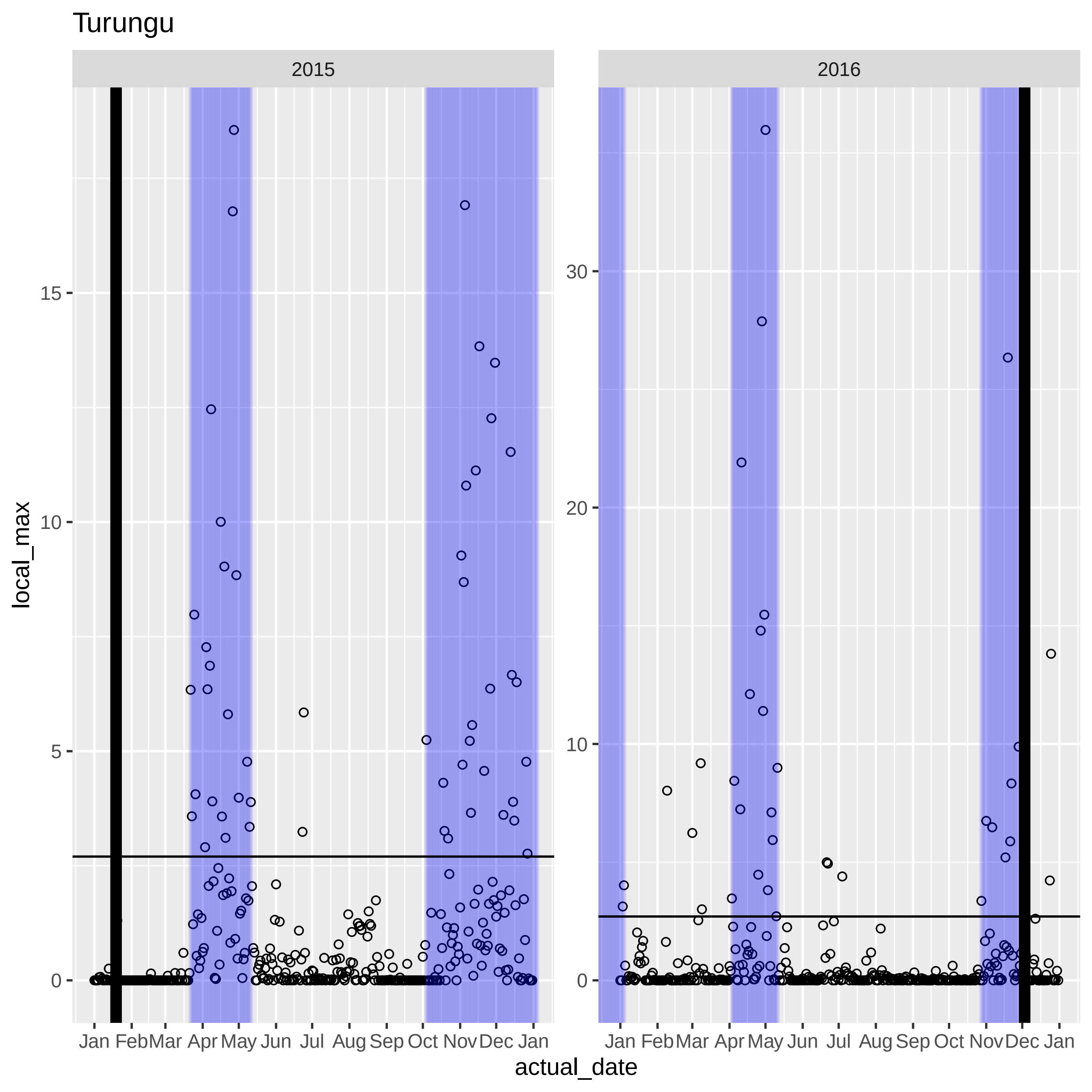

Turungu
2015
2016
local_max
actual_date
0
5
10
15
20
30
Jan Feb Mar Apr May Jun Jul Aug Sep Oct Nov Dec Jan

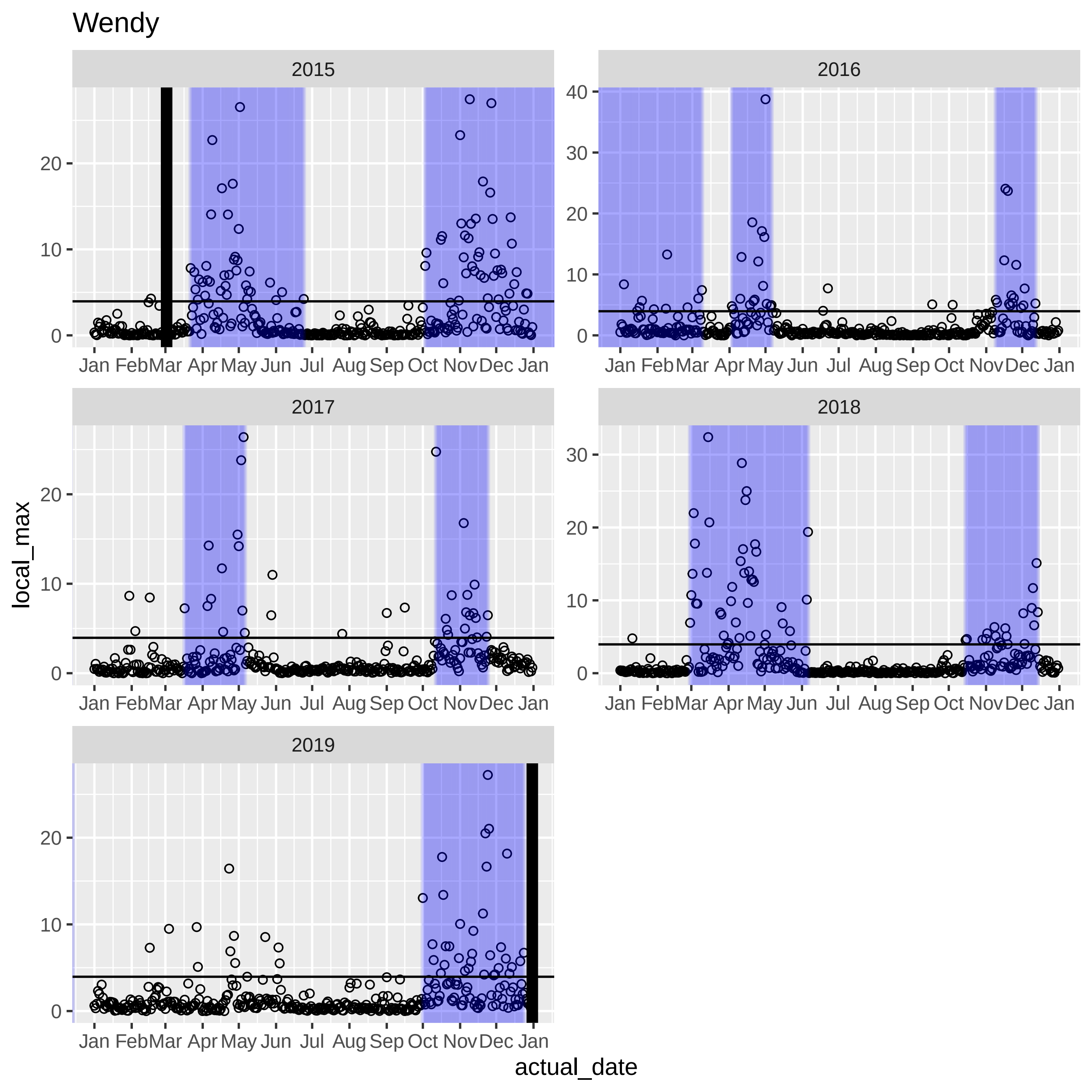

Wendy
2015
2016
2017
2018
2019
local_max
actual_date
Jan Feb Mar Apr May Jun Jul Aug Sep Oct Nov Dec Jan
0
10
20
30
40

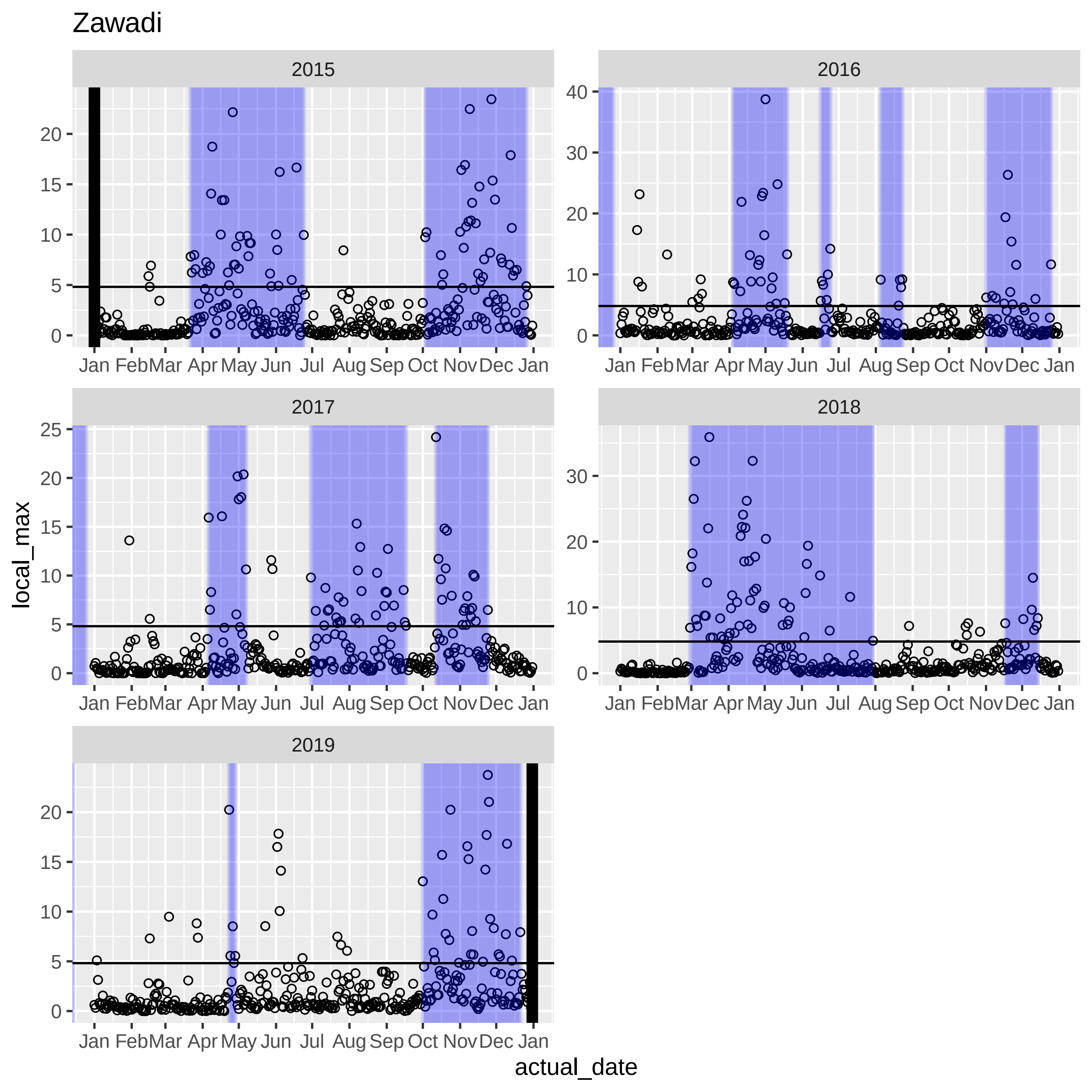
Zawadi
2015
2016
2017
2018
2019
local_max
actual_date
Jan Feb Mar Apr May Jun Jul Aug Sep Oct Nov Dec Jan
