# Supplementary material for: Local rainfall is more likely than distant thunderstorms to affect movement behaviour in Northern Kenyan elephants
Source: PLoS One. 2024 Dec 23;19(12):e0307520. doi: 10.1371/journal.pone.0307520 (PMC11666045; doi:10.1371/journal.pone.0307520)
Supplement: S3 File — State plot indicates per observation (i.e. day) if elephant was in the fast or slow state. Velocity plot indicates the associated average daytime velocity. State 1 = slow state, State 2 = fast state. Latitude-longitude plot indicates when where each elephant was in state 1 or 2. Residual and QQ plot indicates model fit. (PDF) [file pone.0307520.s003.pdf]

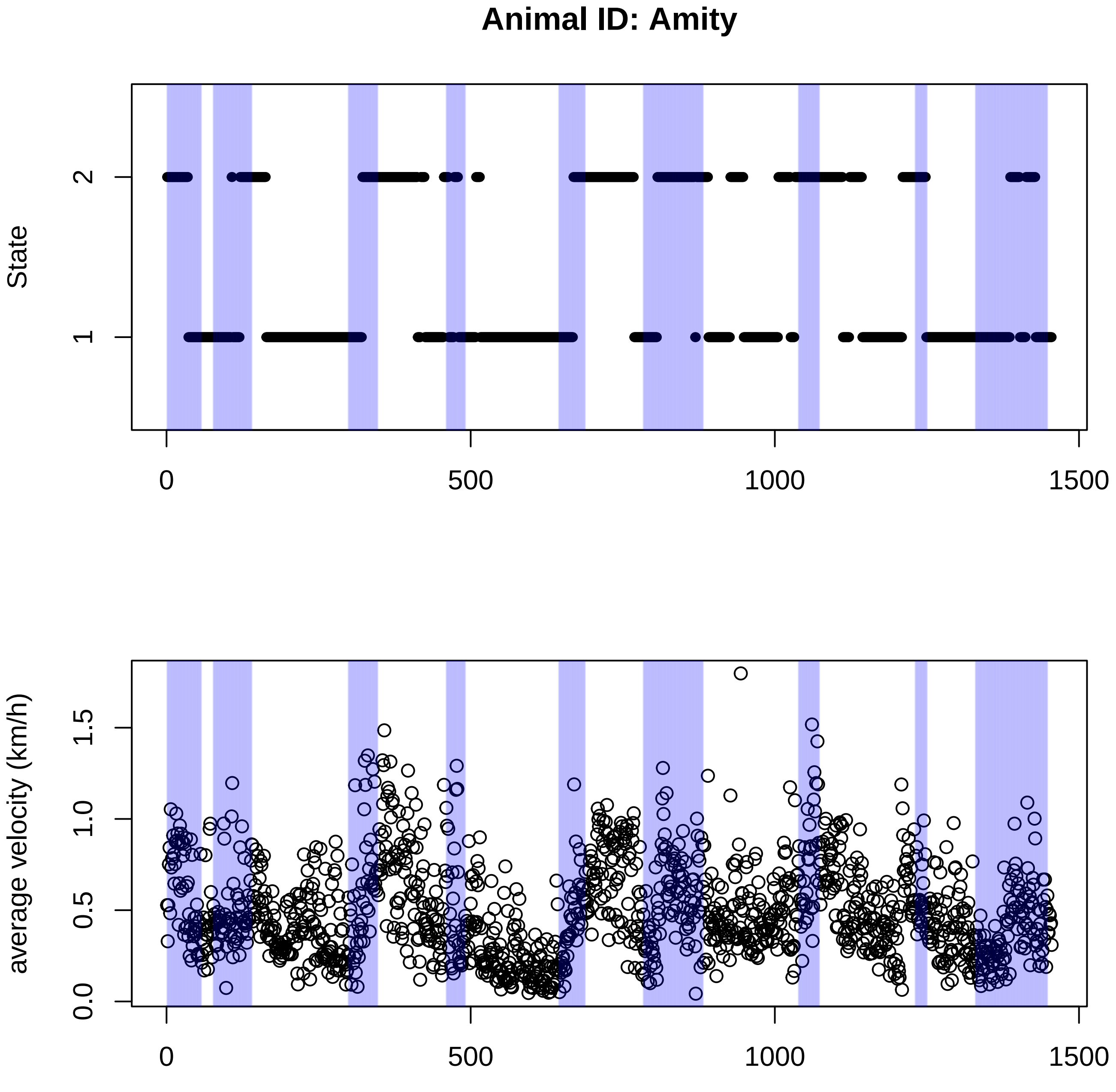

Animal ID: Amity
State
2
1
0
500
1000
1500
average velocity (km/h)
0.0
0.5
1.0
1.5

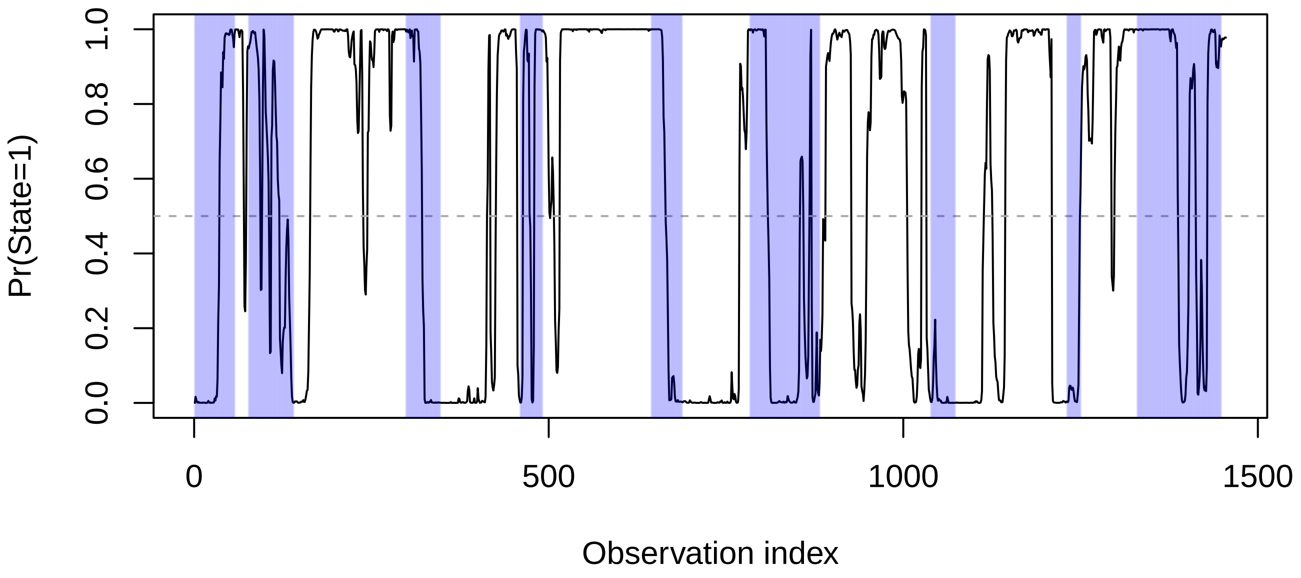

Pr(State=1)
0.0
0.2
0.4
0.6
0.8
1.0
0
500
1000
1500
Observation index

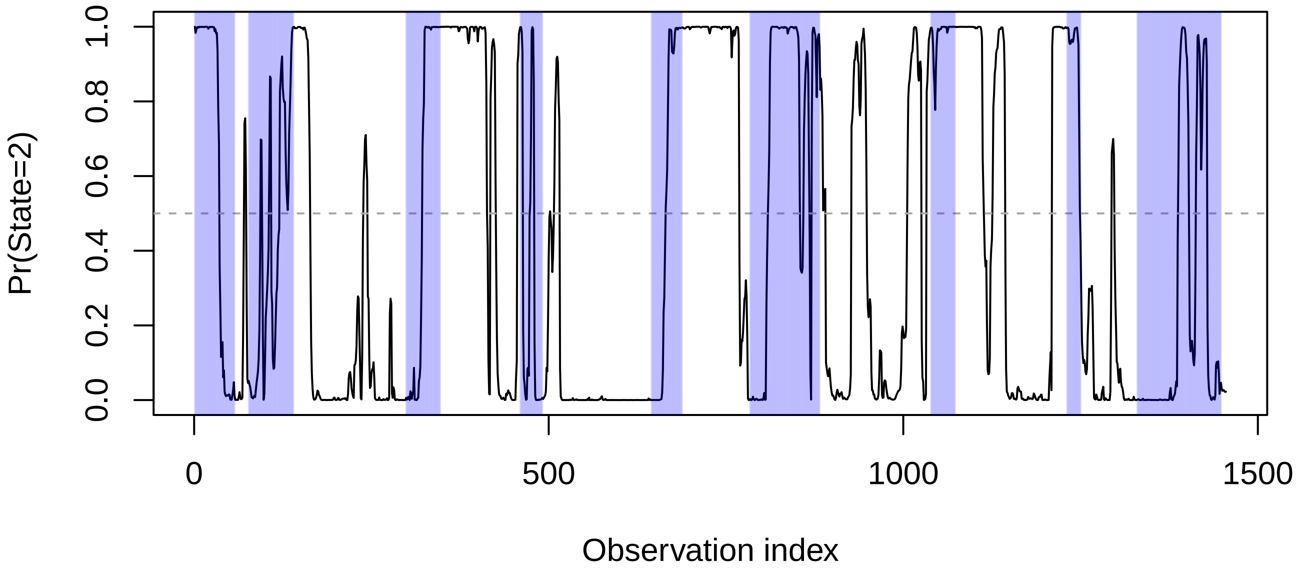

Pr(State=2)
0.0
0.2
0.4
0.6
0.8
1.0
0
500
1000
1500
Observation index

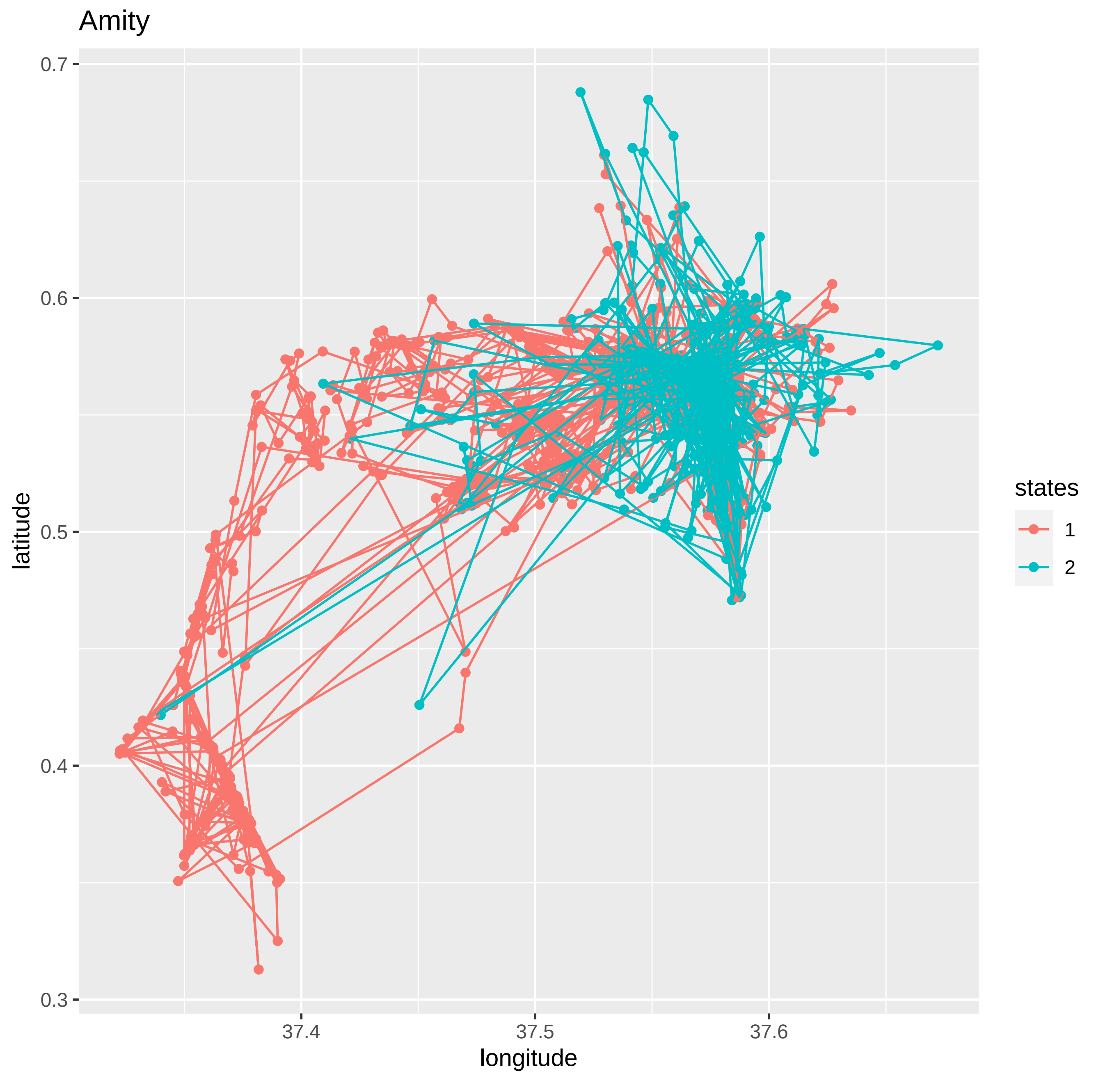

Amity
0.7
0.6
0.5
0.4
0.3
latitude
37.4
37.5
37.6
longitude
states
1
2

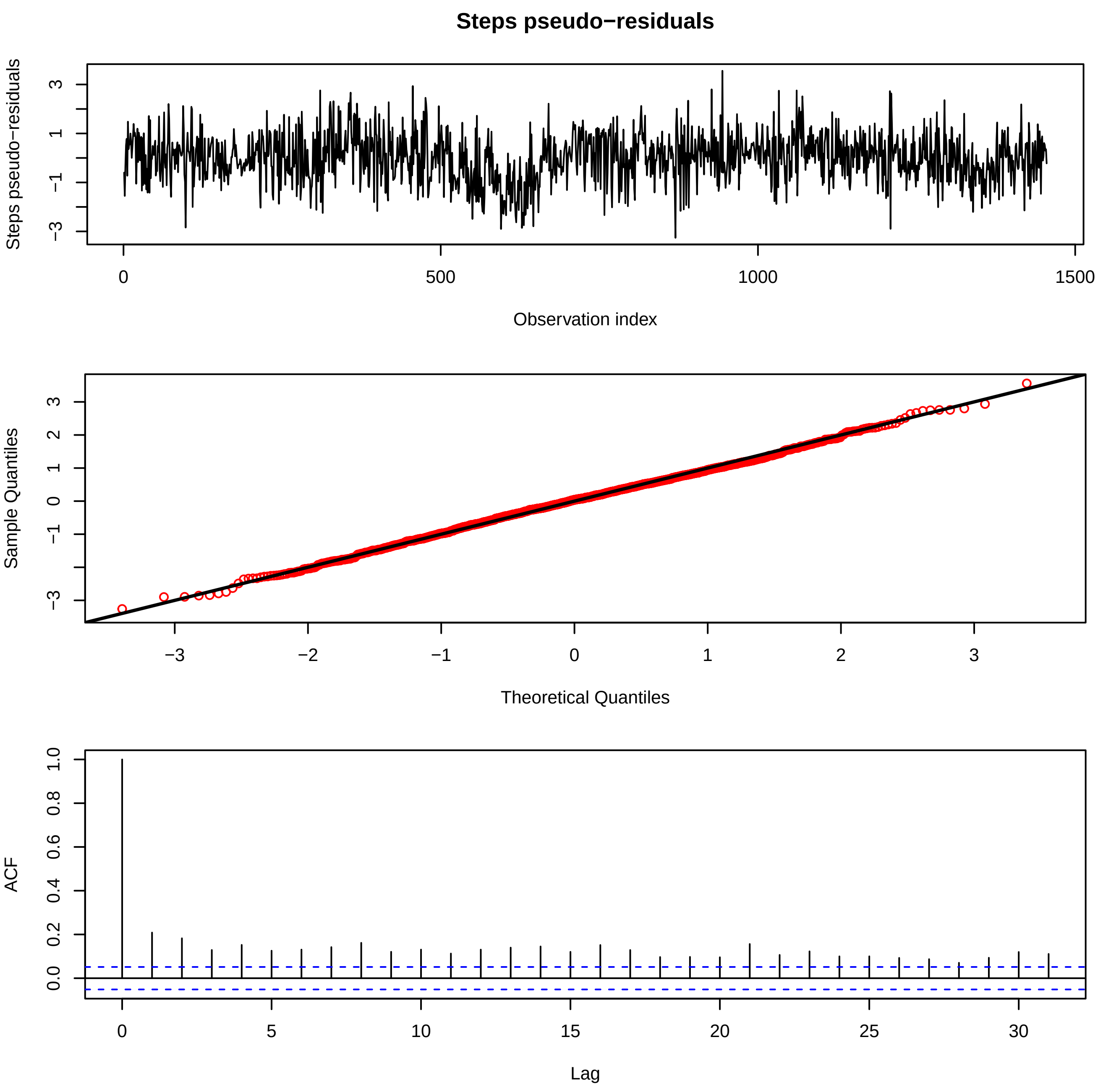

Steps pseudo−residuals
Steps pseudo−residuals
Observation index
Sample Quantiles
Theoretical Quantiles
ACF
Lag

Animal ID: Annabelle

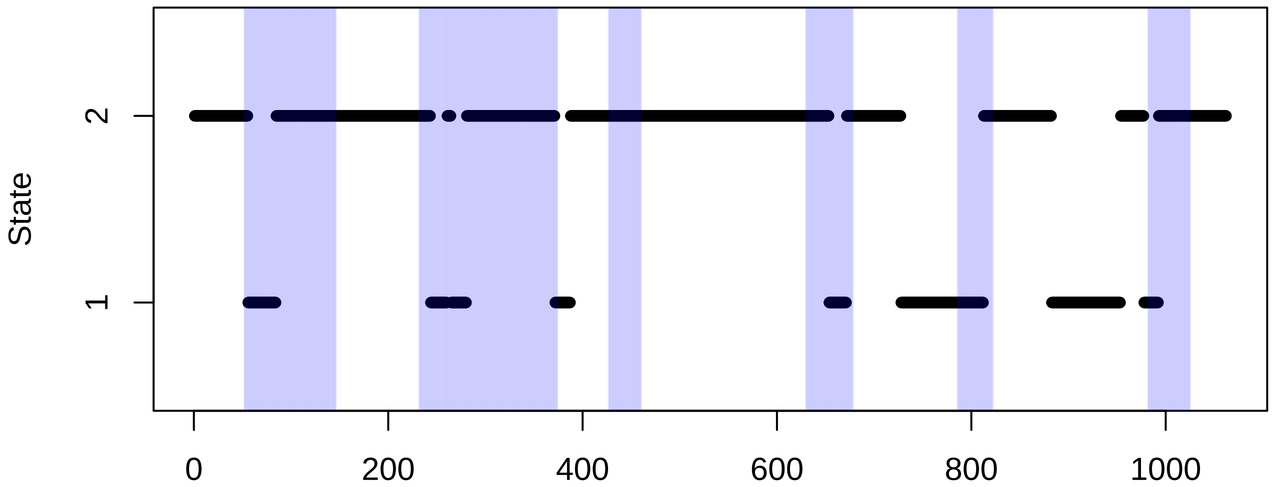

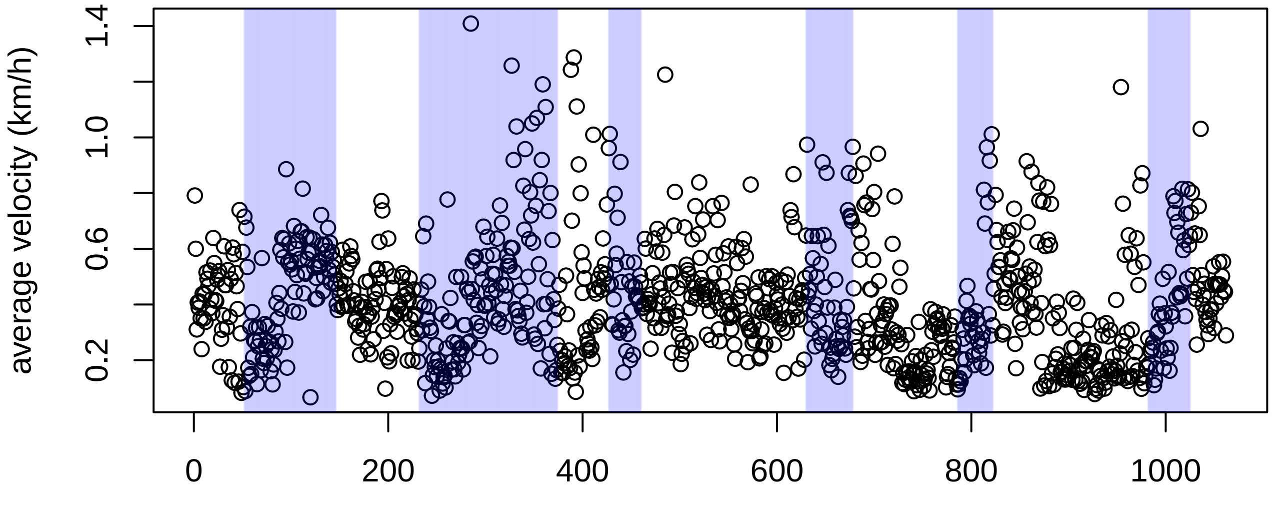

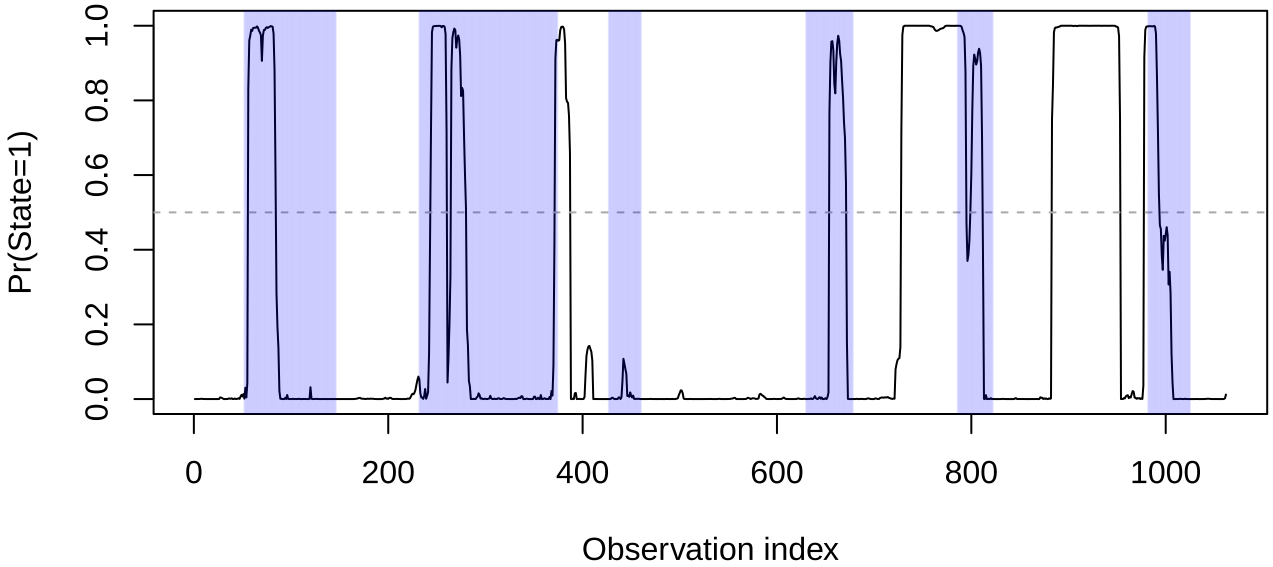
Pr(State=1)
0.0
0.2
0.4
0.6
0.8
1.0
0
200
400
600
800
1000
Observation index

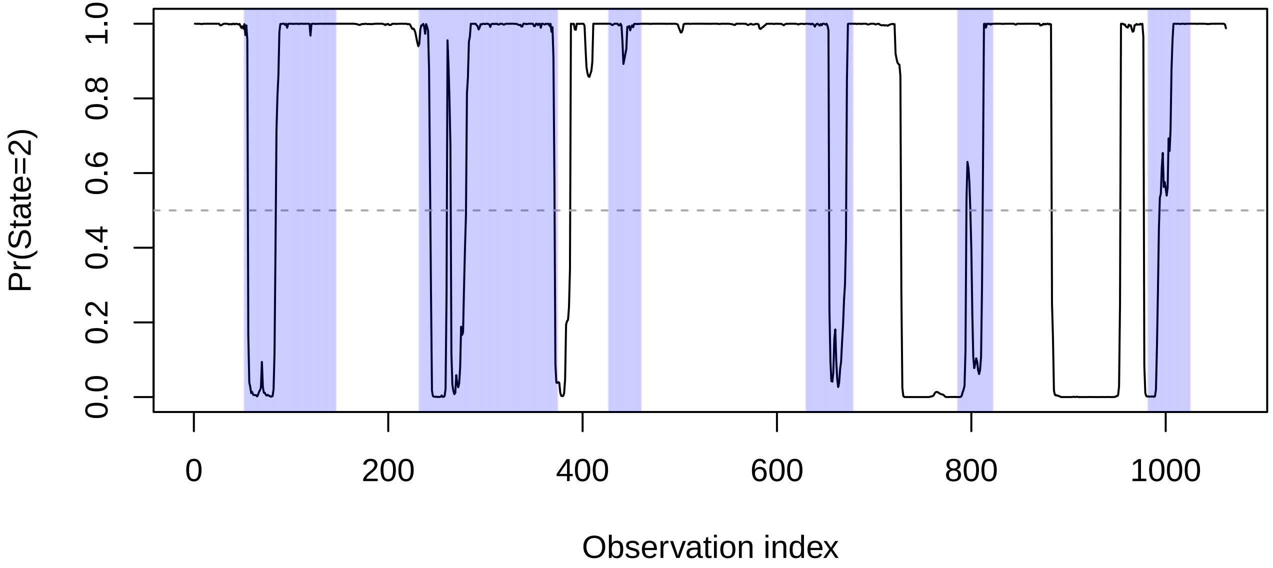
Pr(State=2)
0.0
0.2
0.4
0.6
0.8
1.0
0
200
400
600
800
1000
Observation index

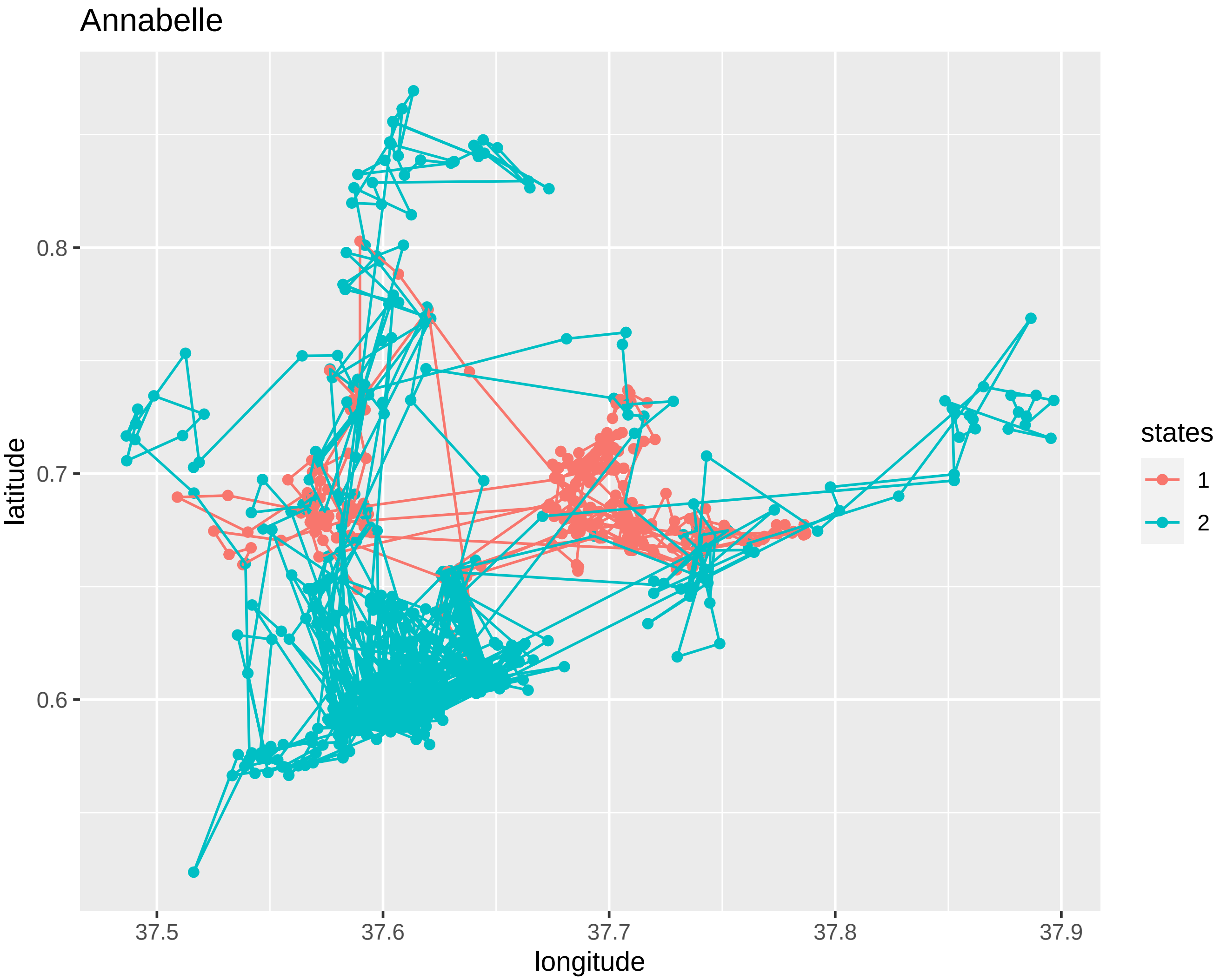

Annabelle
latitude
0.8
0.7
0.6
longitude
37.5
37.6
37.7
37.8
37.9
states
1
2

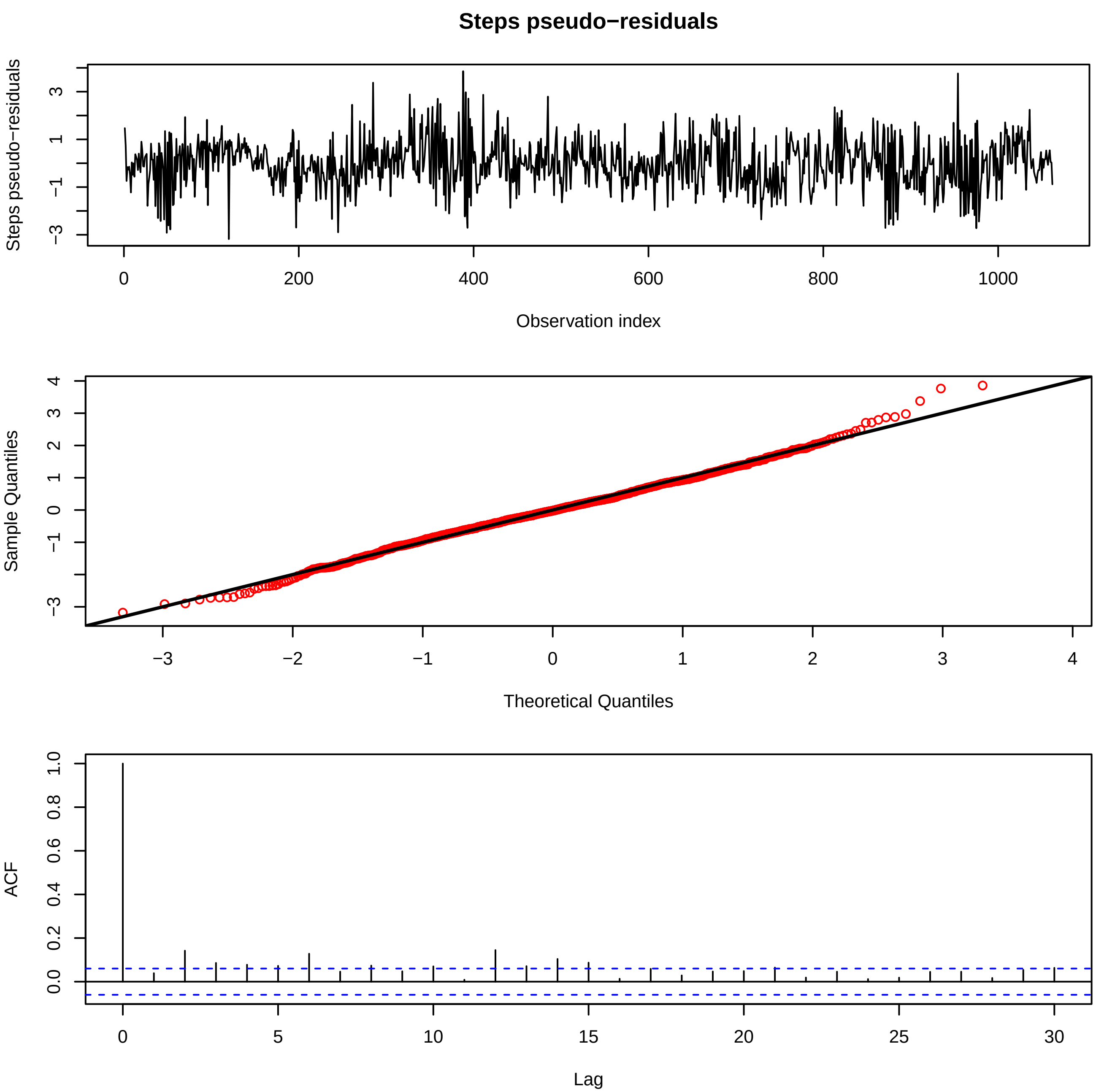

Steps pseudo−residuals
Steps pseudo−residuals
Observation index
Sample Quantiles
Theoretical Quantiles
ACF
Lag

Animal ID: Arden

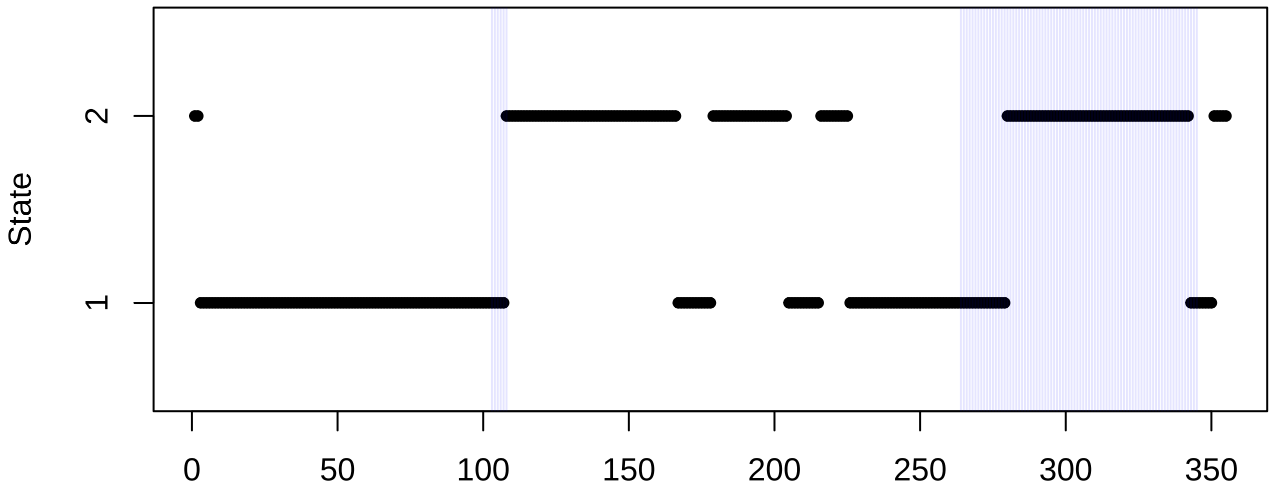

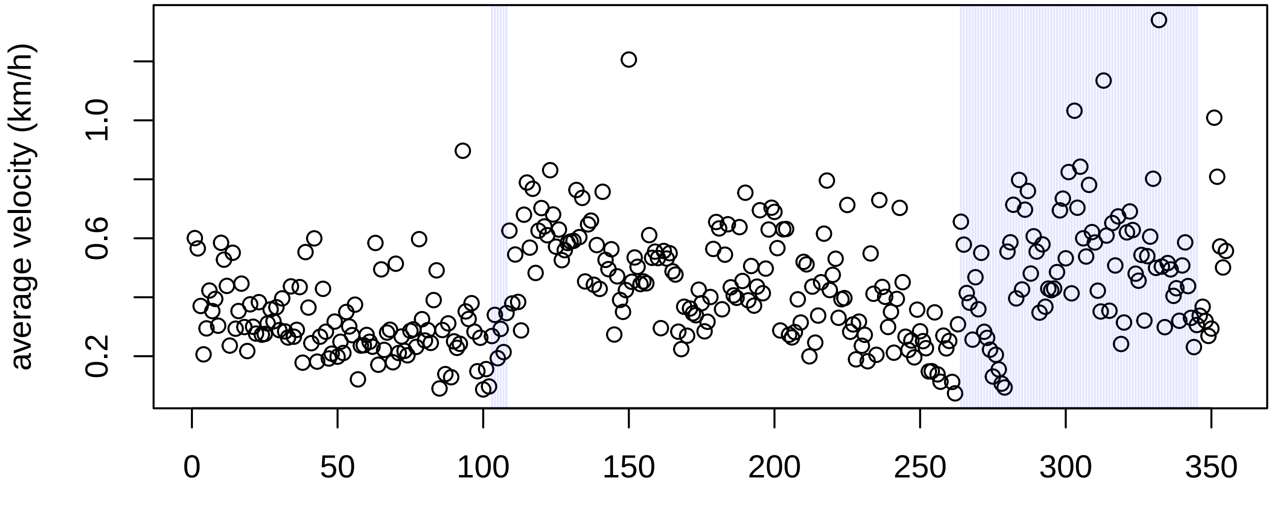

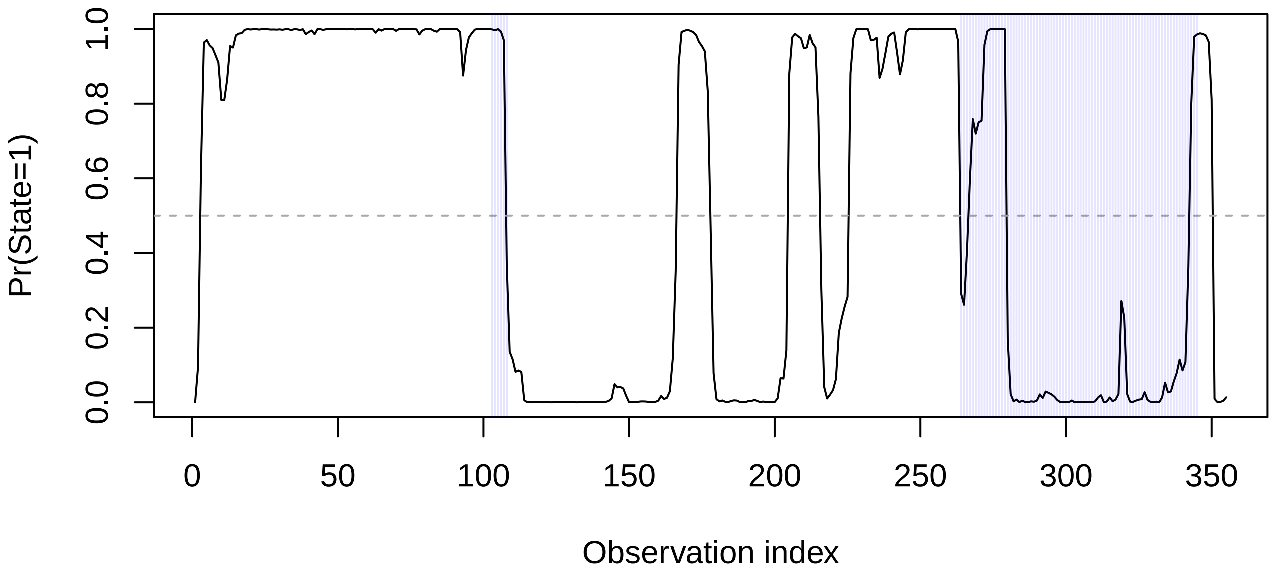

Pr(State=1)
0.0
0.2
0.4
0.6
0.8
1.0
0
50
100
150
200
250
300
350
Observation index

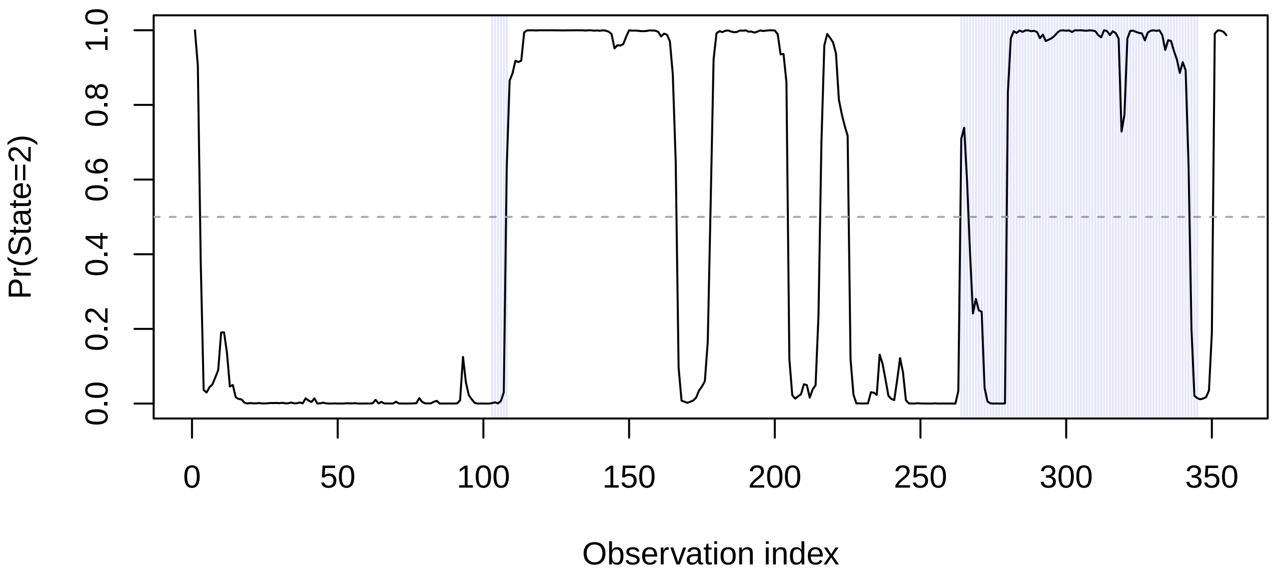

Pr(State=2)
0.0
0.2
0.4
0.6
0.8
1.0
0
50
100
150
200
250
300
350
Observation index

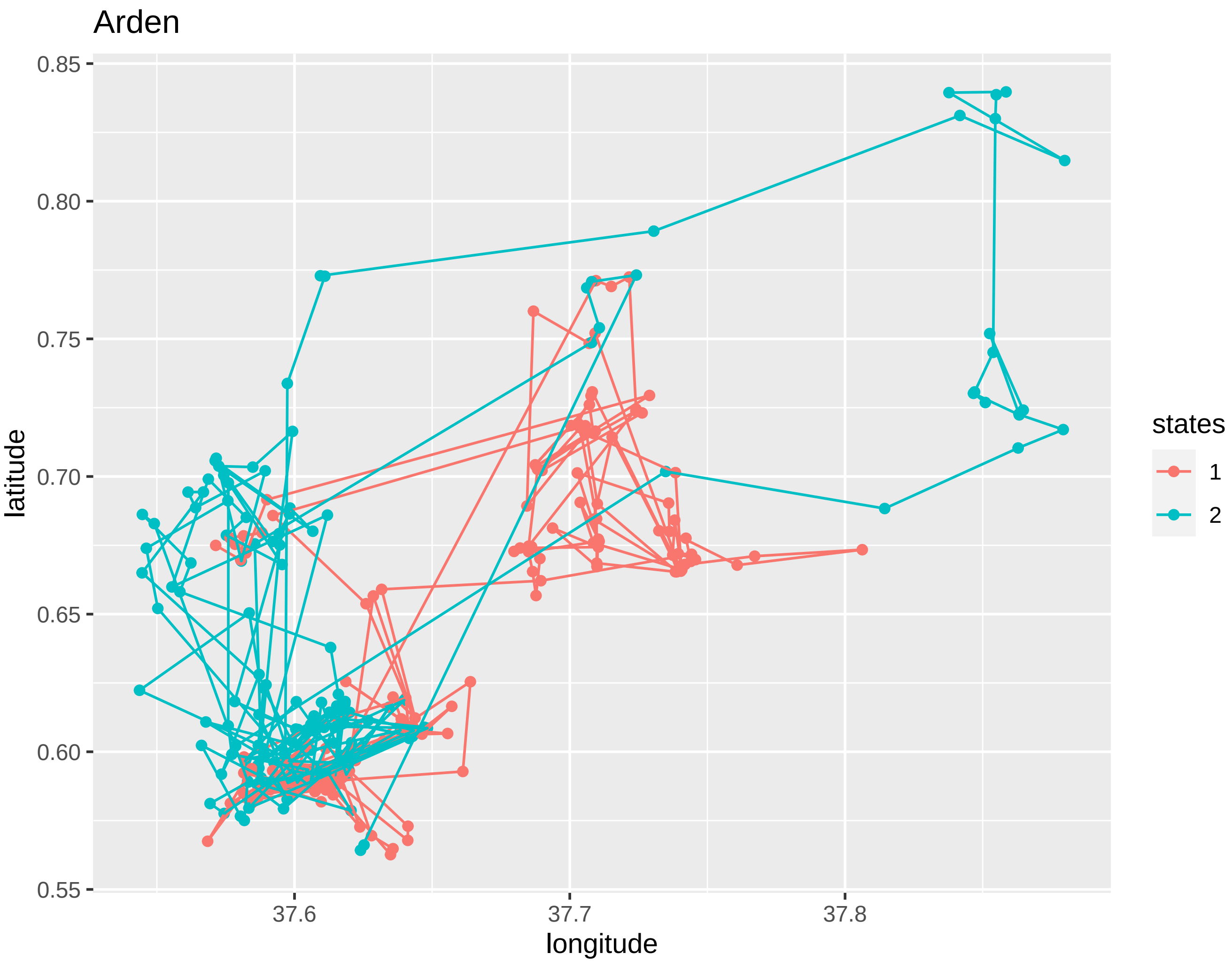
Arden
0.85
0.80
0.75
0.70
0.65
0.60
0.55
latitude
37.6
37.7
37.8
longitude
states
1
2

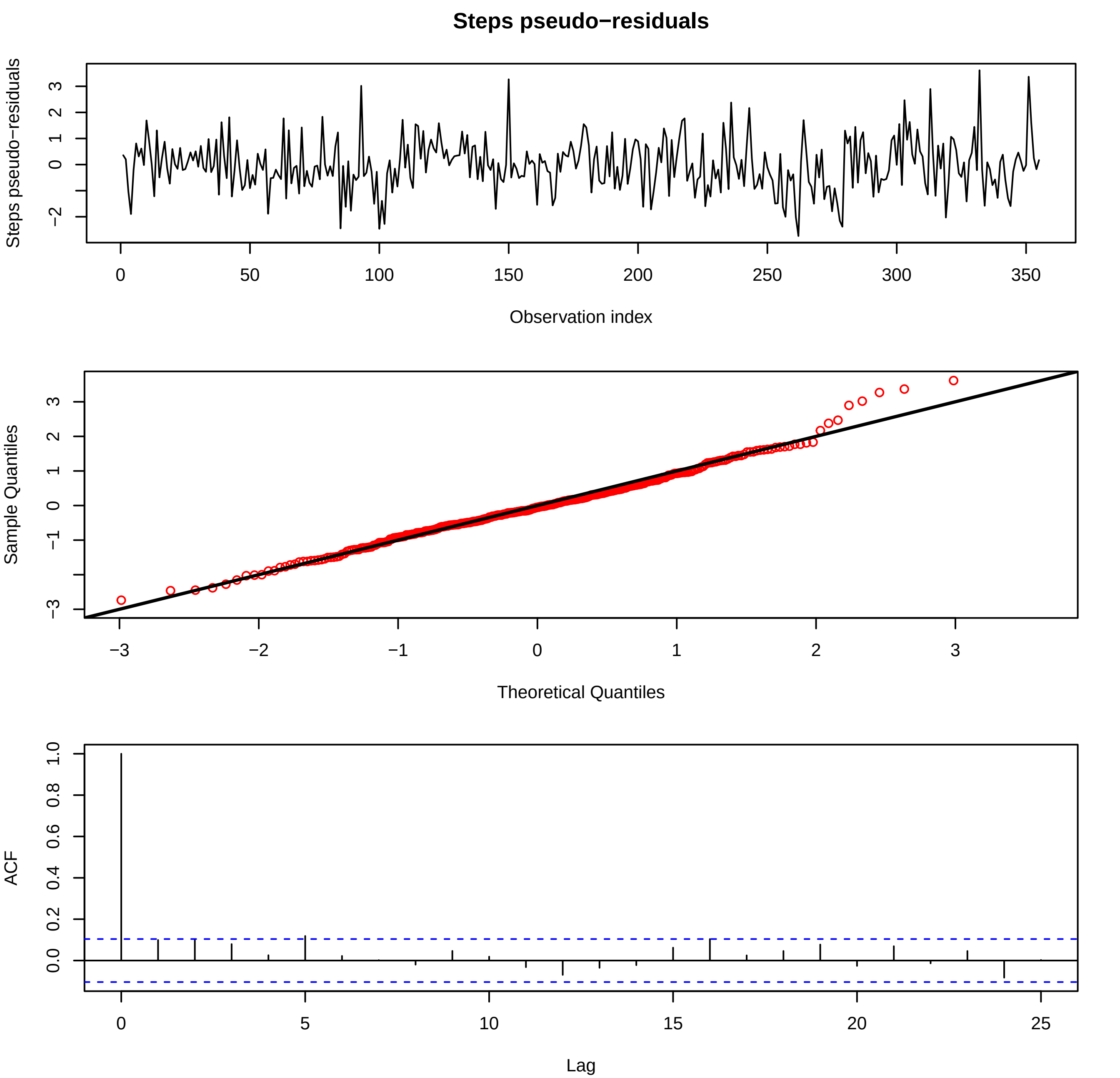
Steps pseudo−residuals
Steps pseudo−residuals
Observation index
Sample Quantiles
Theoretical Quantiles
ACF
Lag

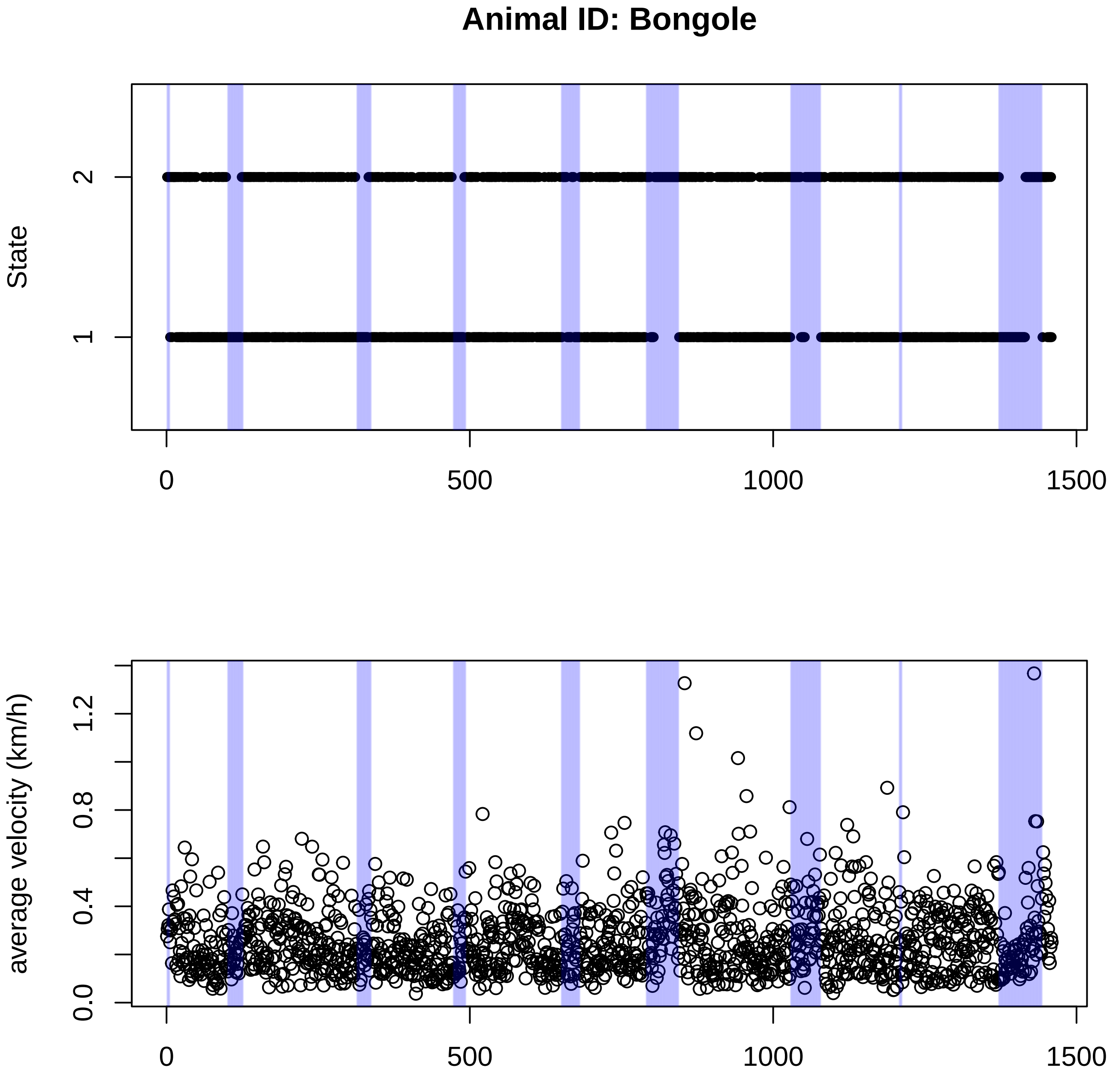
Animal ID: Bongole
State
2
1
0
500
1000
1500
average velocity (km/h)
0.0
0.4
0.8
1.2
0
500
1000
1500

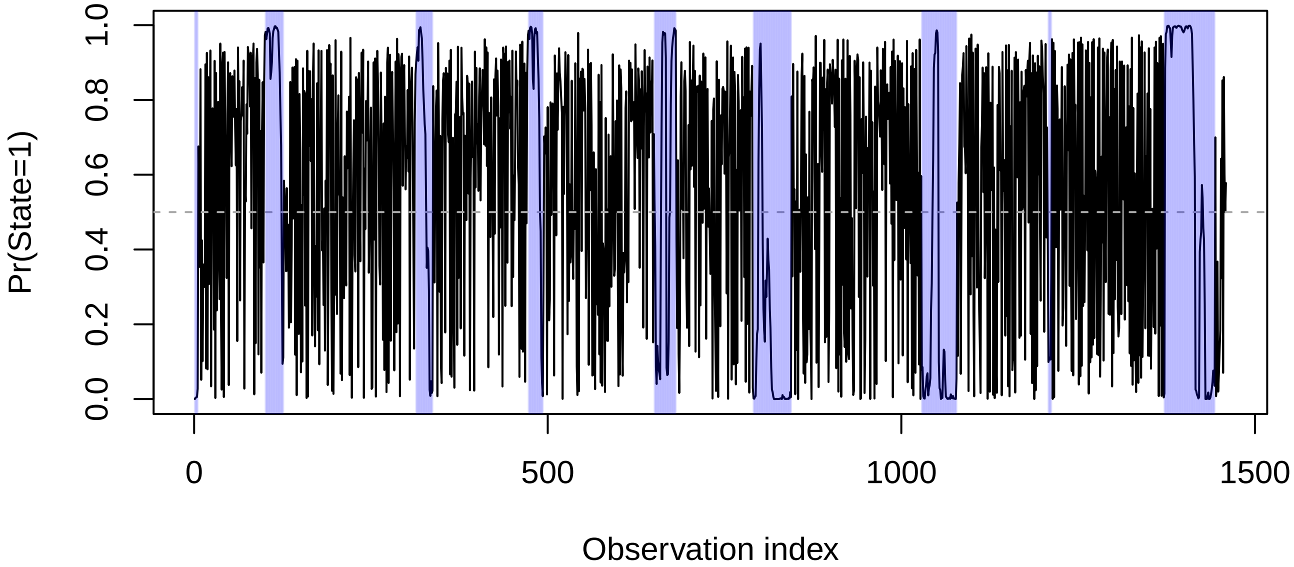
Pr(State=1)
0.0
0.2
0.4
0.6
0.8
1.0
0
500
1000
1500
Observation index

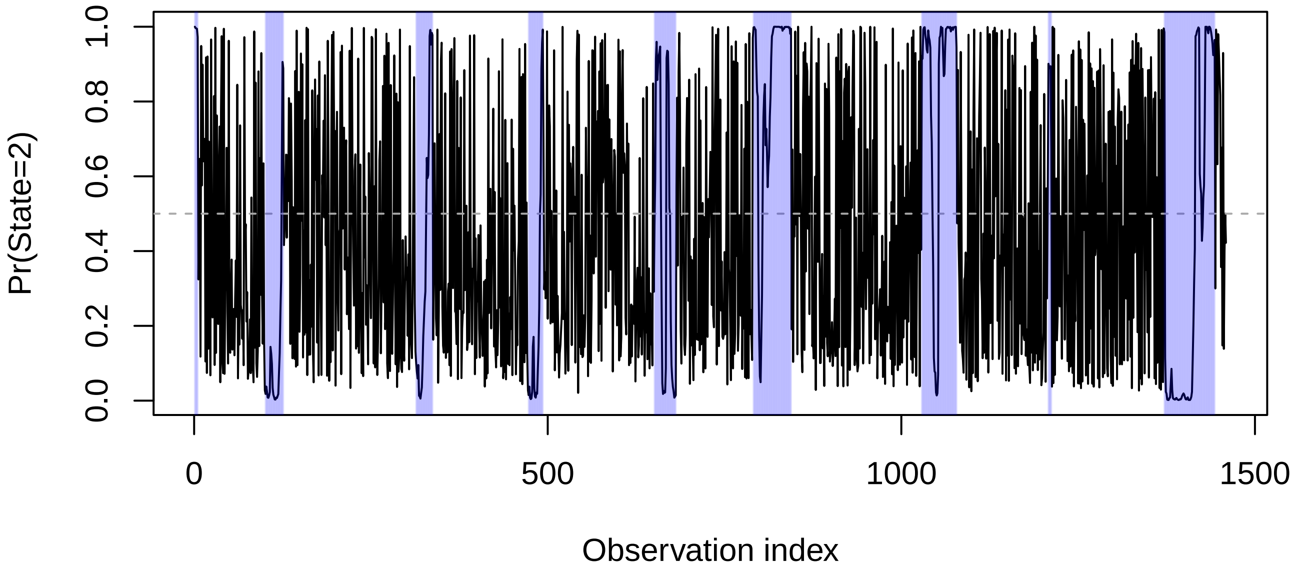
Pr(State=2)
0.0
0.2
0.4
0.6
0.8
1.0
0
500
1000
1500
Observation index

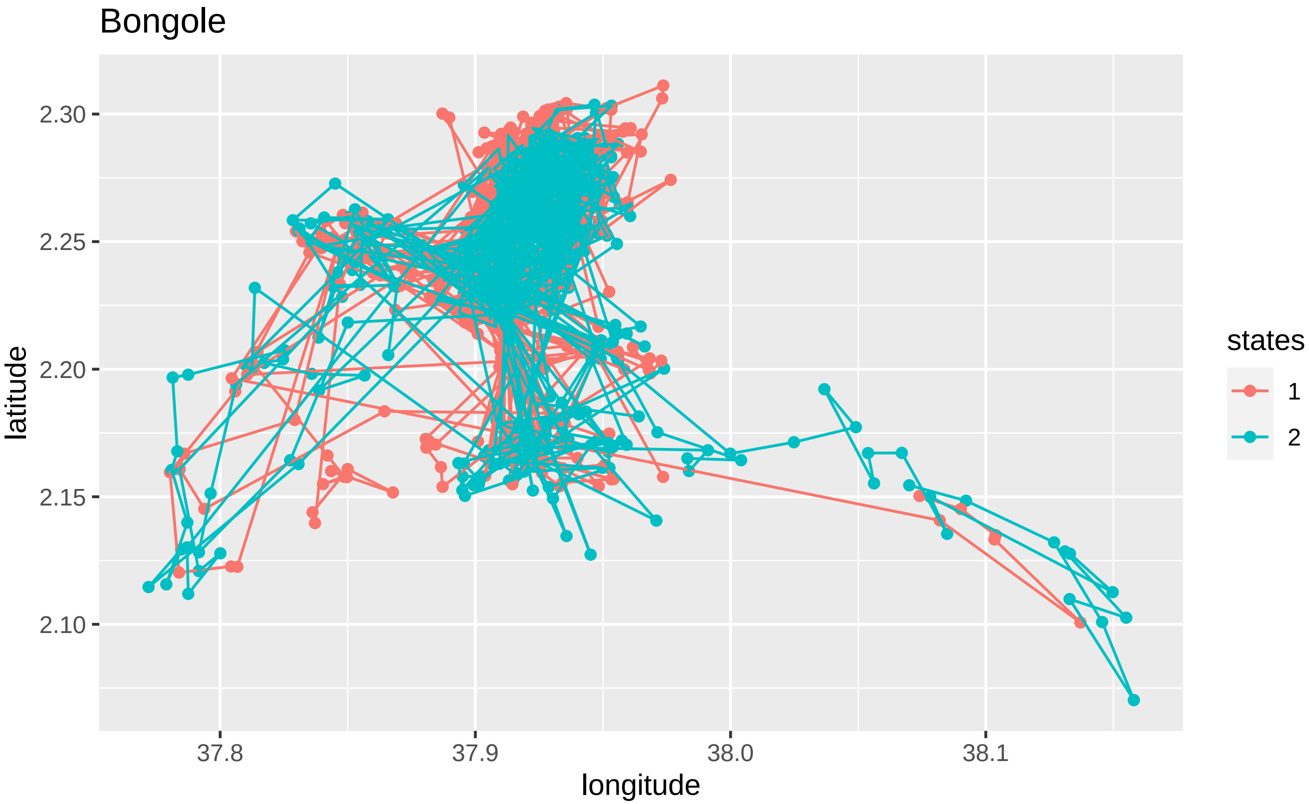

Bongole
latitude
2.30
2.25
2.20
2.15
2.10
longitude
37.8
37.9
38.0
38.1
states
1
2

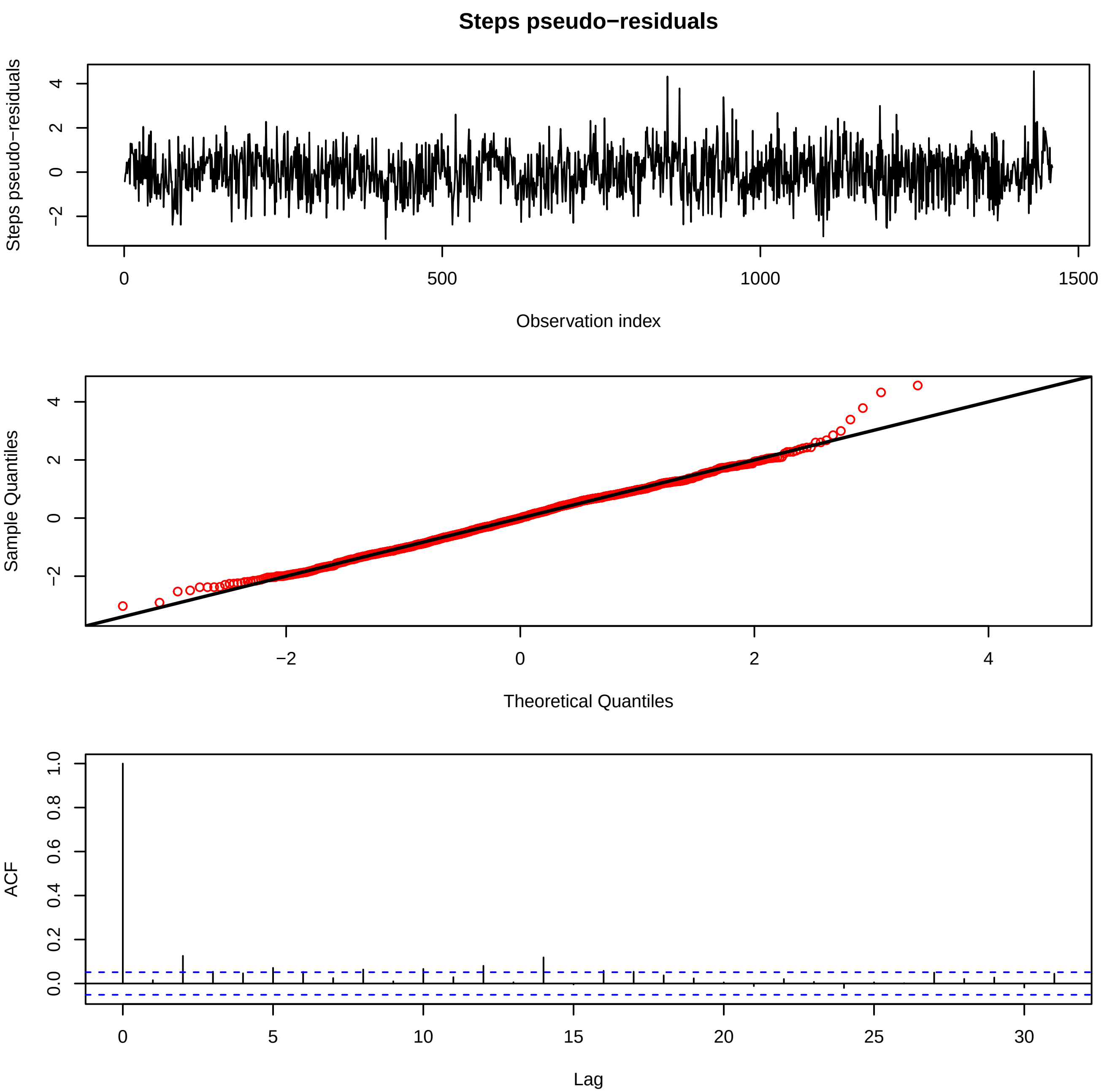

Steps pseudo−residuals
Steps pseudo−residuals
Observation index
Sample Quantiles
Theoretical Quantiles
ACF
Lag

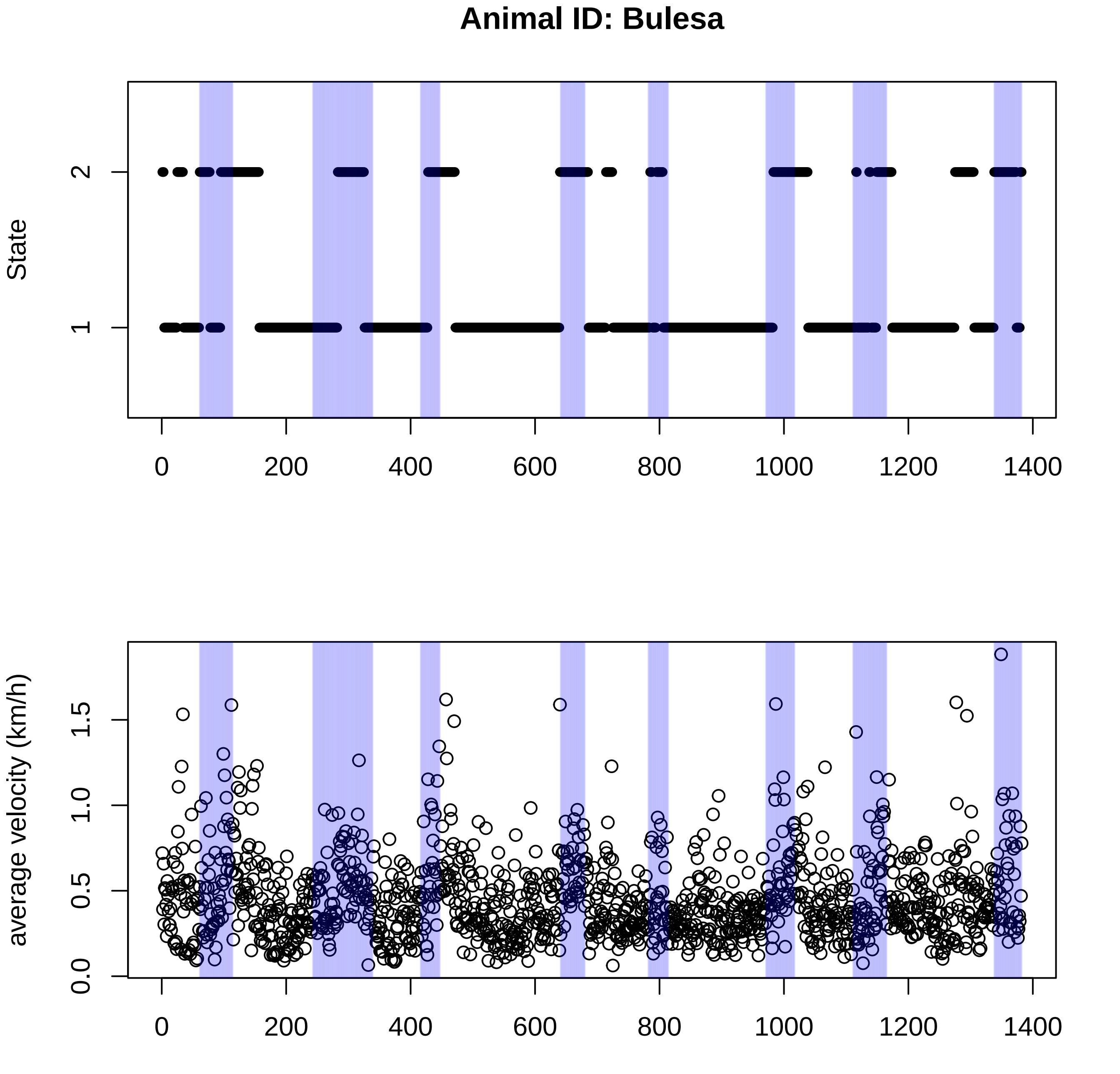
Animal ID: Bulesa
State
2
1
0
200
400
600
800
1000
1200
1400
average velocity (km/h)
0.0
0.5
1.0
1.5

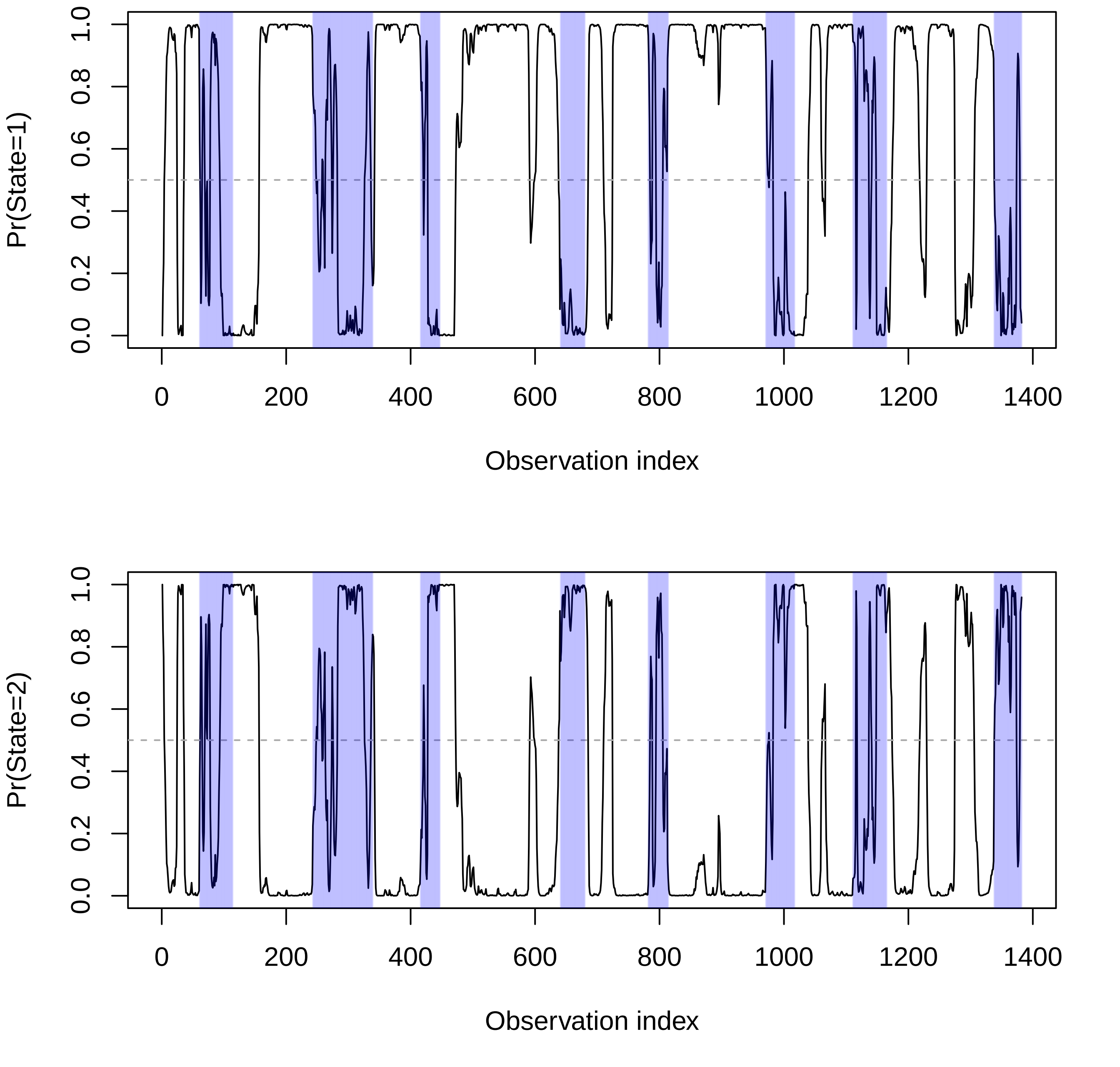

Pr(State=1)
1.0
0.8
0.6
0.4
0.2
0.0
0
200
400
600
800
1000
1200
1400
Observation index
Pr(State=2)
1.0
0.8
0.6
0.4
0.2
0.0
0
200
400
600
800
1000
1200
1400
Observation index

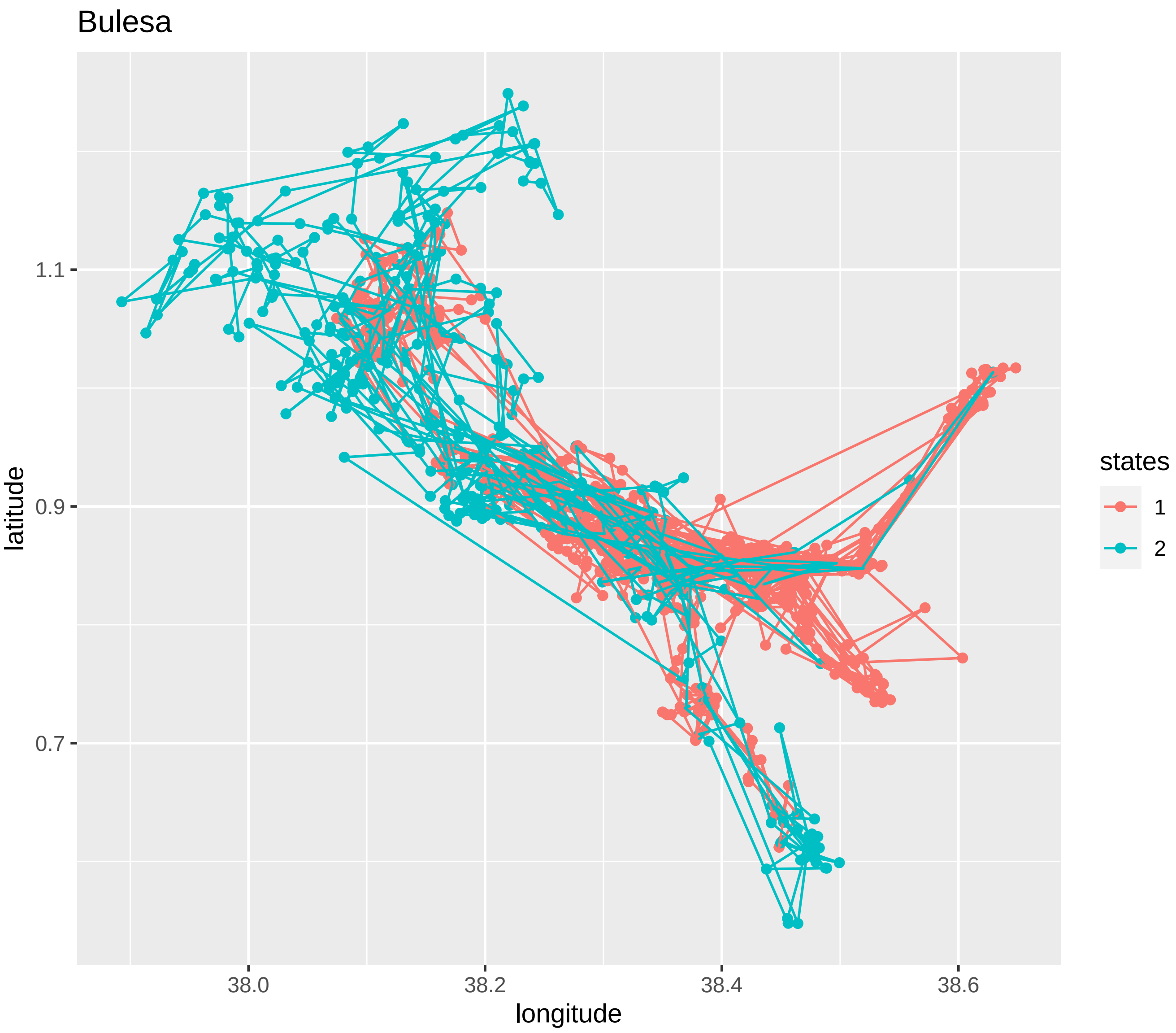

Bulesa
latitude
longitude
1.1
0.9
0.7
38.0
38.2
38.4
38.6
states
1
2

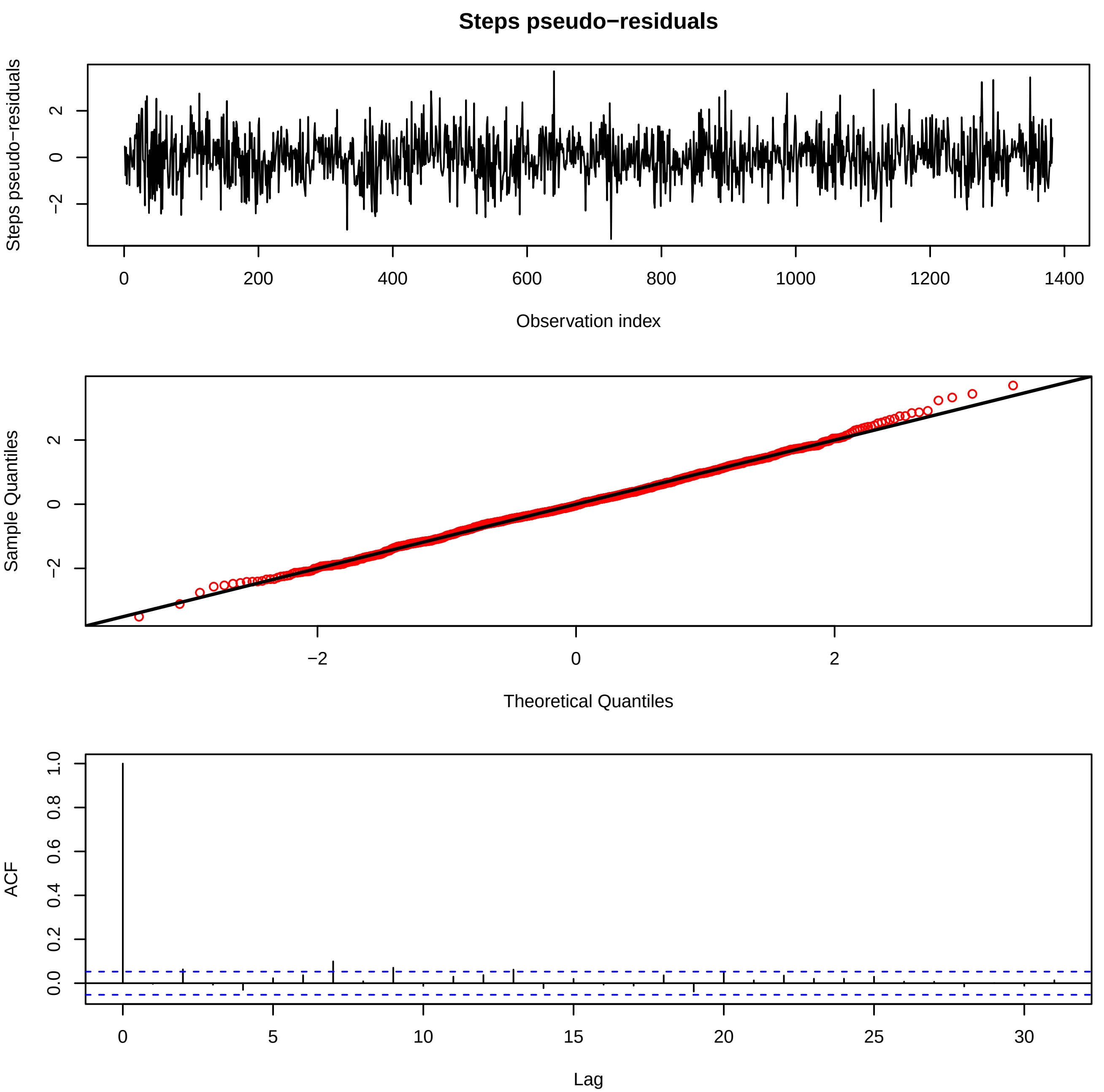
Steps pseudo−residuals
Steps pseudo−residuals
Observation index
Sample Quantiles
Theoretical Quantiles
ACF
Lag

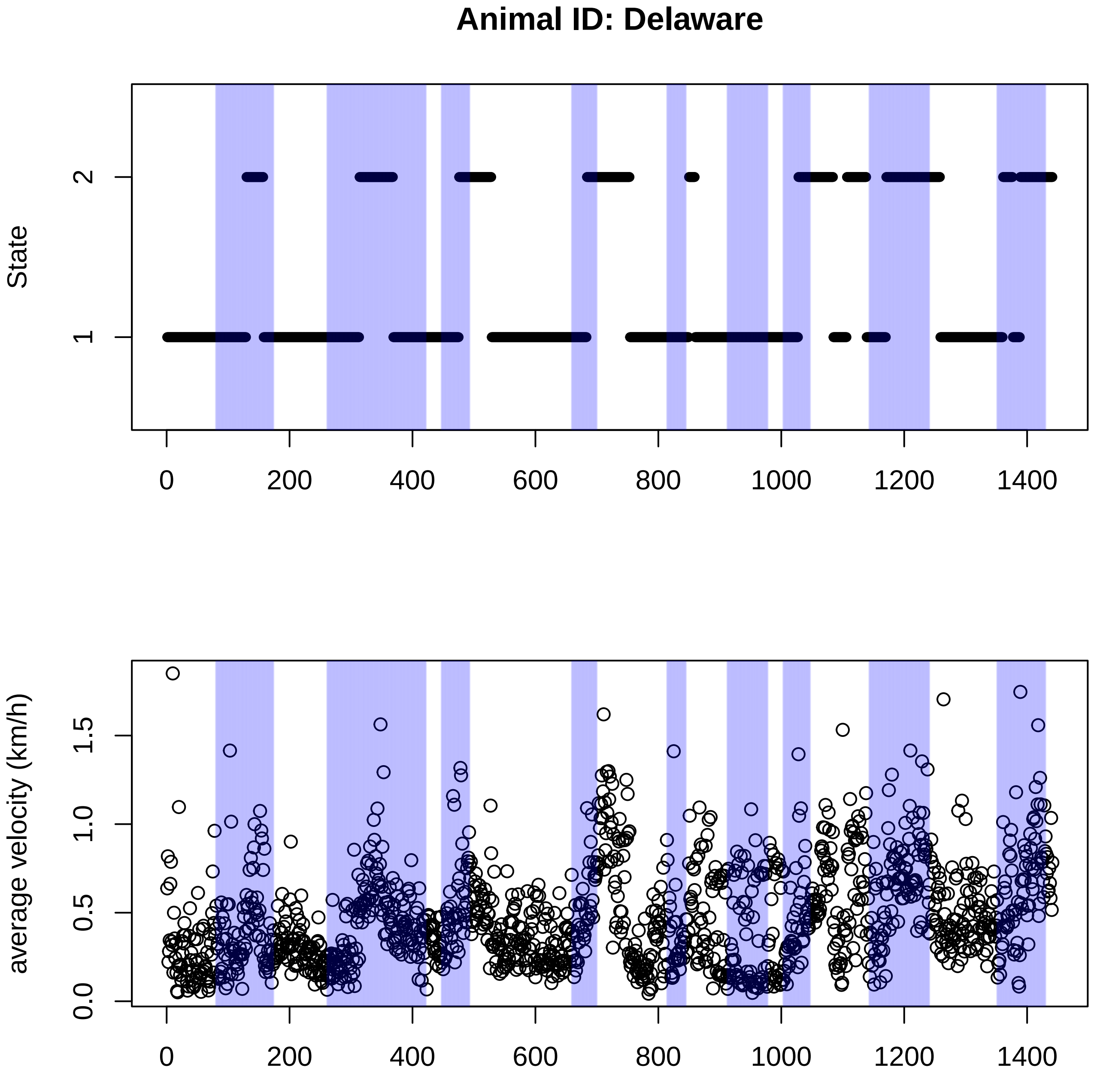

Animal ID: Delaware
State
2
1
0
200
400
600
800
1000
1200
1400
average velocity (km/h)
0.0
0.5
1.0
1.5

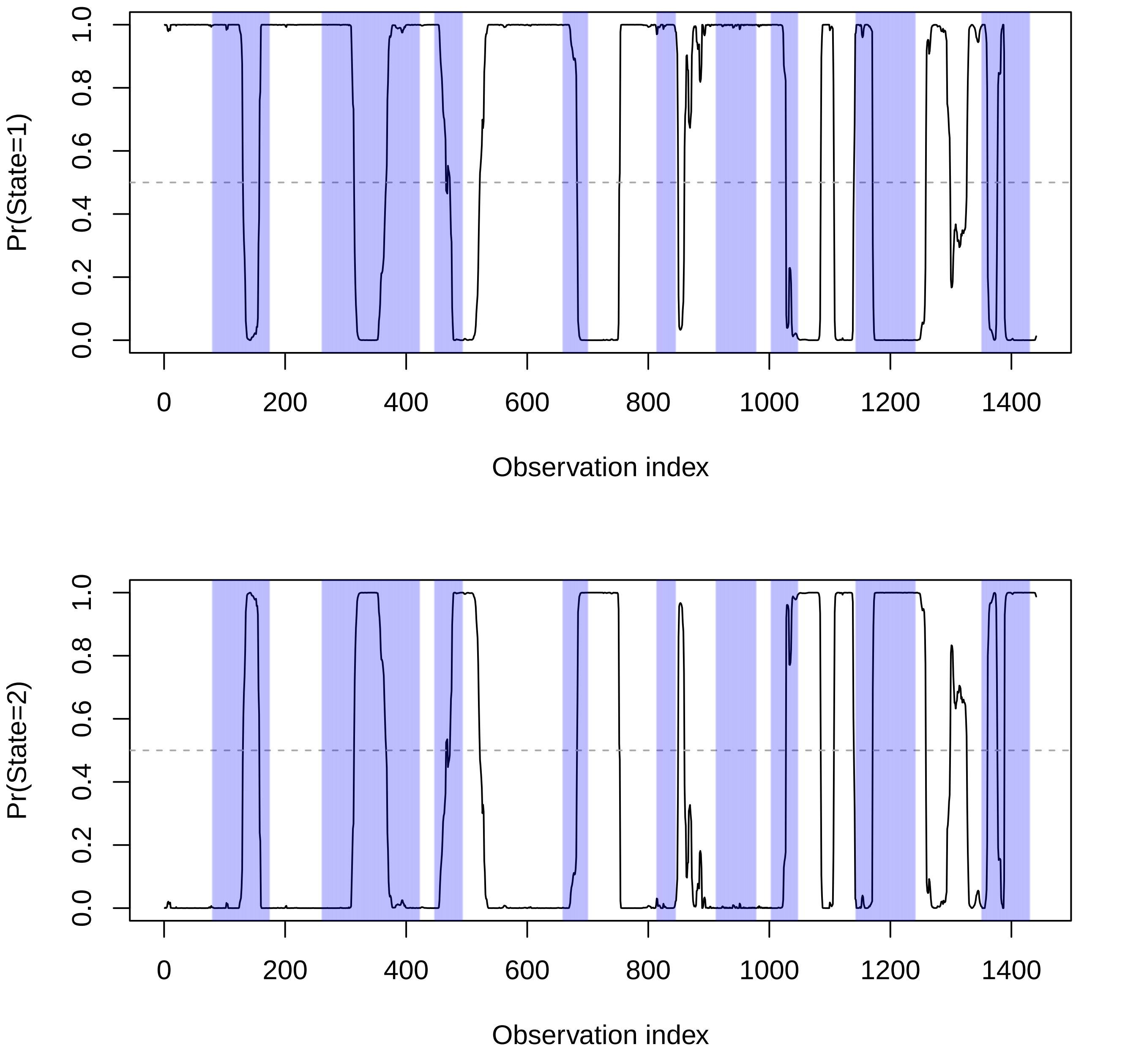

Pr(State=1)
1.0
0.8
0.6
0.4
0.2
0.0
0
200
400
600
800
1000
1200
1400
Observation index
Pr(State=2)
1.0
0.8
0.6
0.4
0.2
0.0
0
200
400
600
800
1000
1200
1400
Observation index

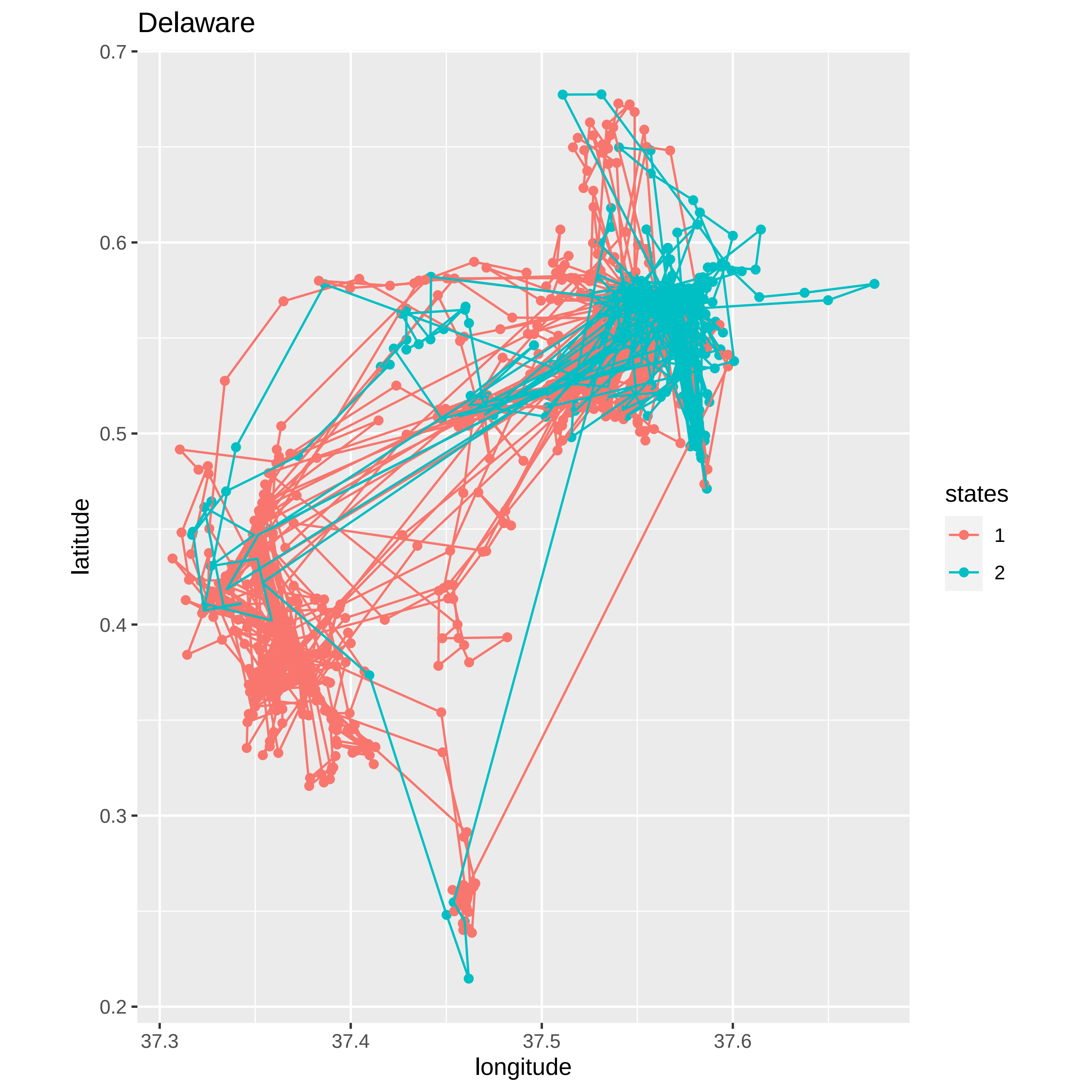

Delaware
latitude
longitude
0.7
0.6
0.5
0.4
0.3
0.2
37.3
37.4
37.5
37.6
states
1
2

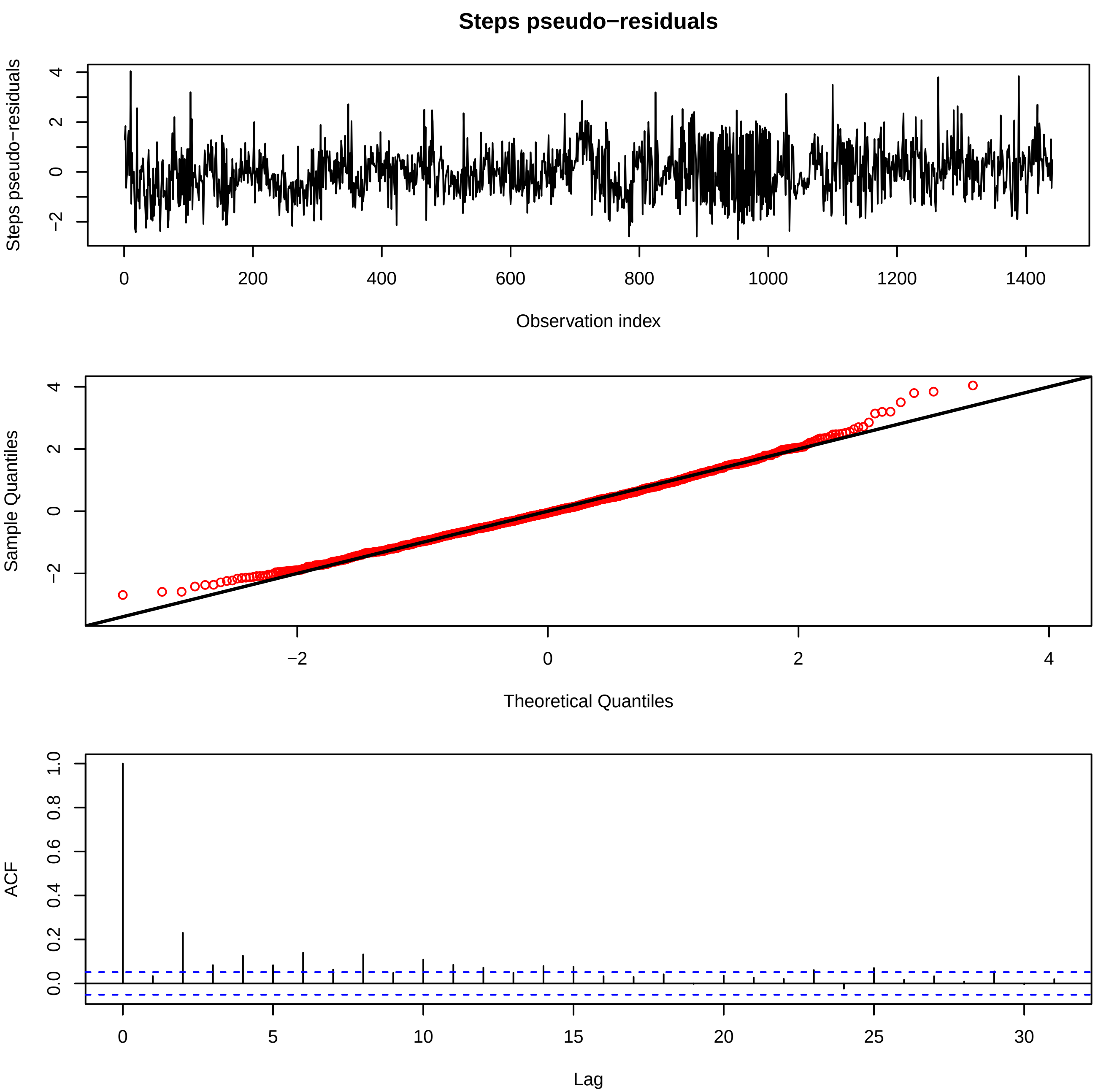

Steps pseudo−residuals
Steps pseudo−residuals
Observation index
Sample Quantiles
Theoretical Quantiles
ACF
Lag

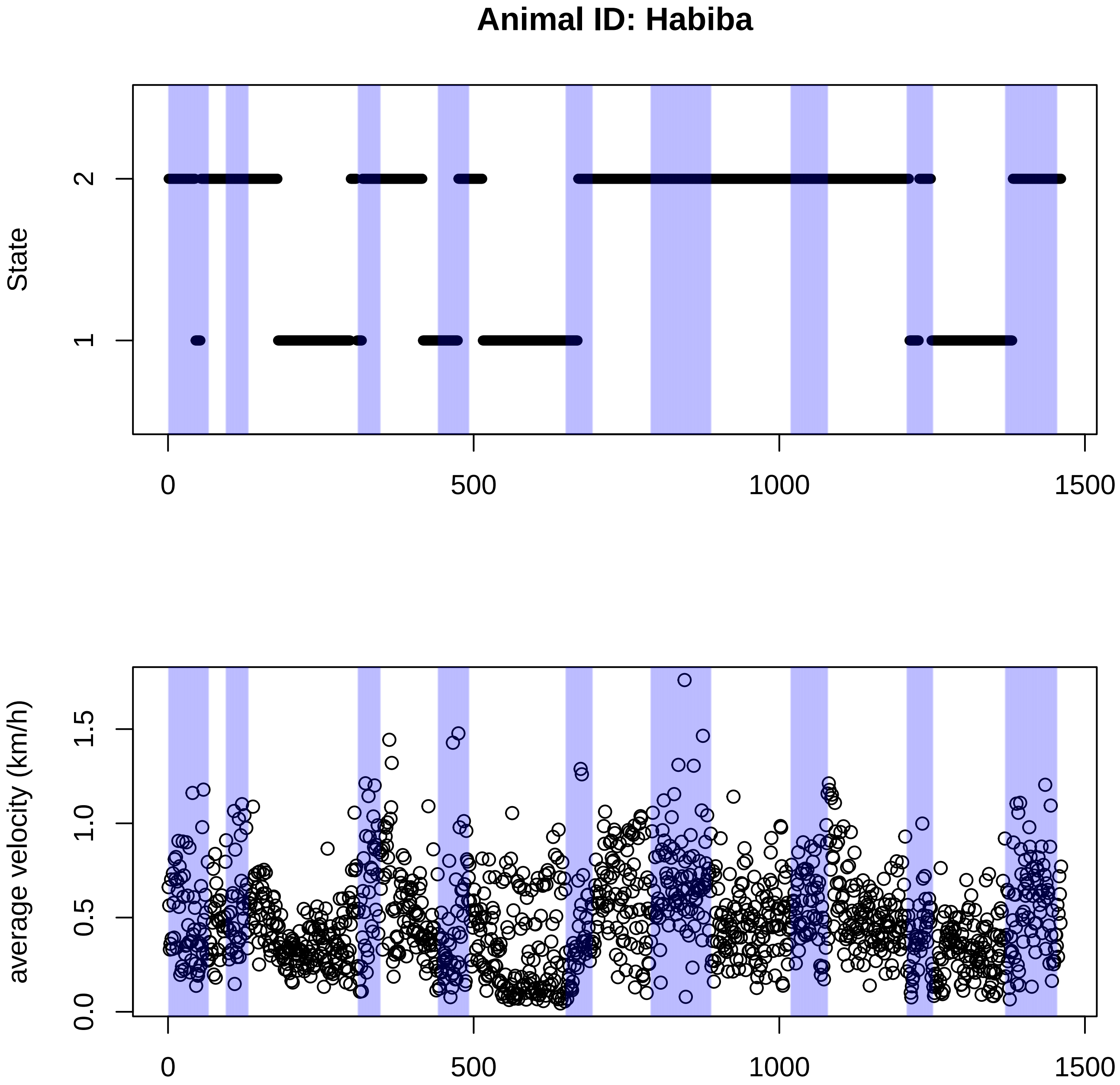

Animal ID: Habiba
State
2
1
0
500
1000
1500
average velocity (km/h)
0.0
0.5
1.0
1.5
0
500
1000
1500

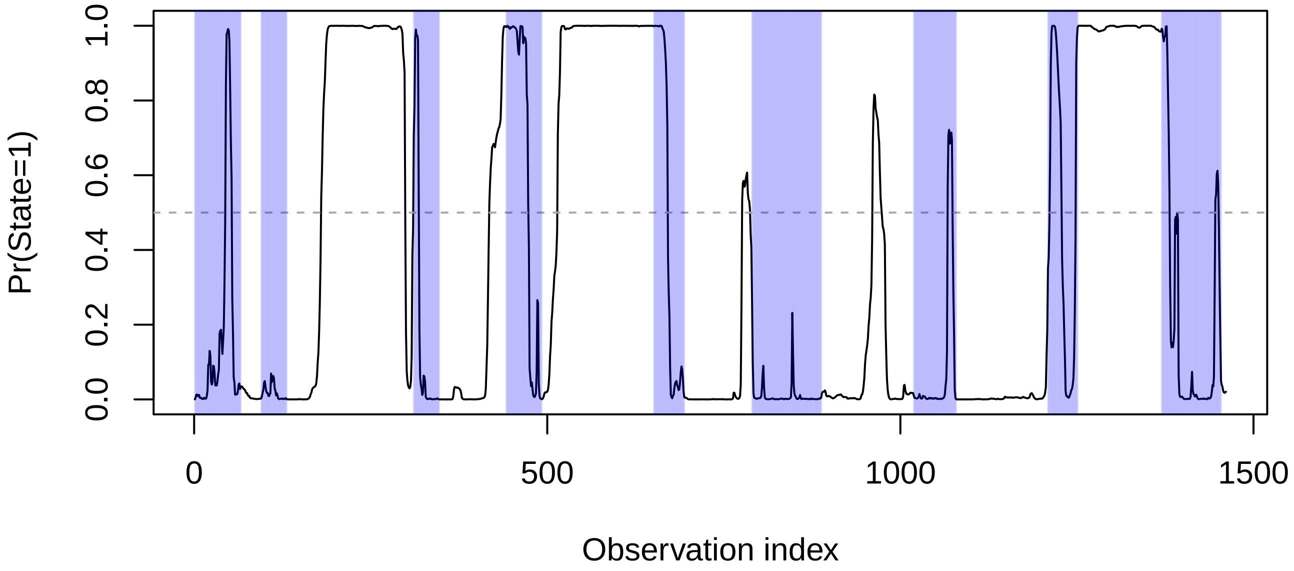
Pr(State=1)
1.0
0.8
0.6
0.4
0.2
0.0
0
500
1000
1500
Observation index

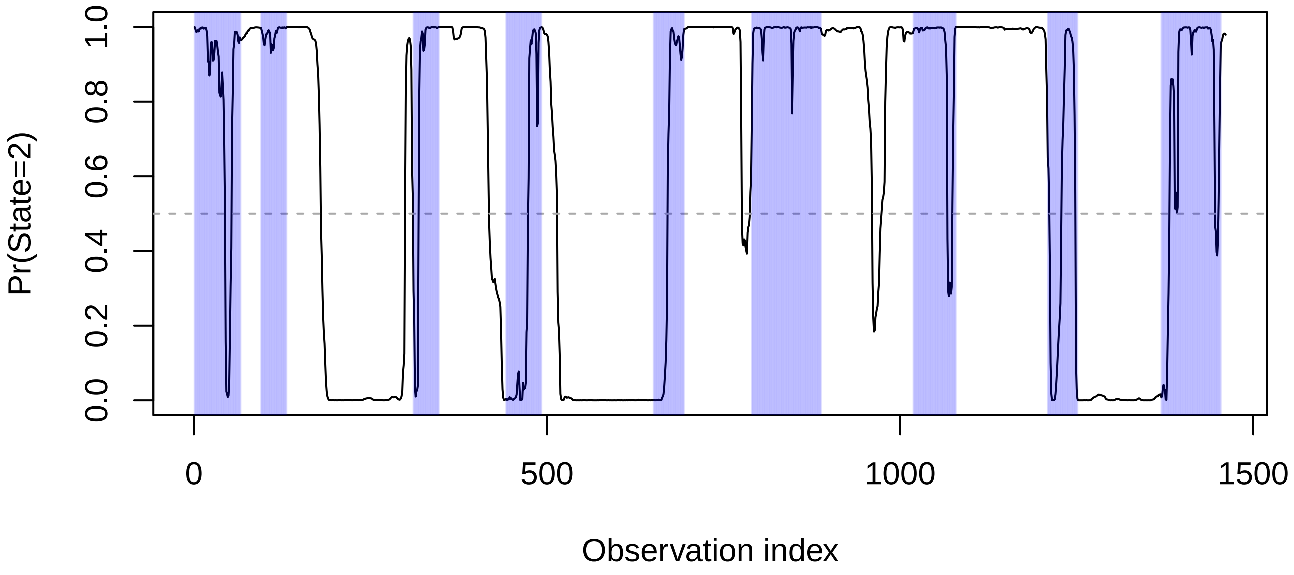
Pr(State=2)
1.0
0.8
0.6
0.4
0.2
0.0
0
500
1000
1500
Observation index

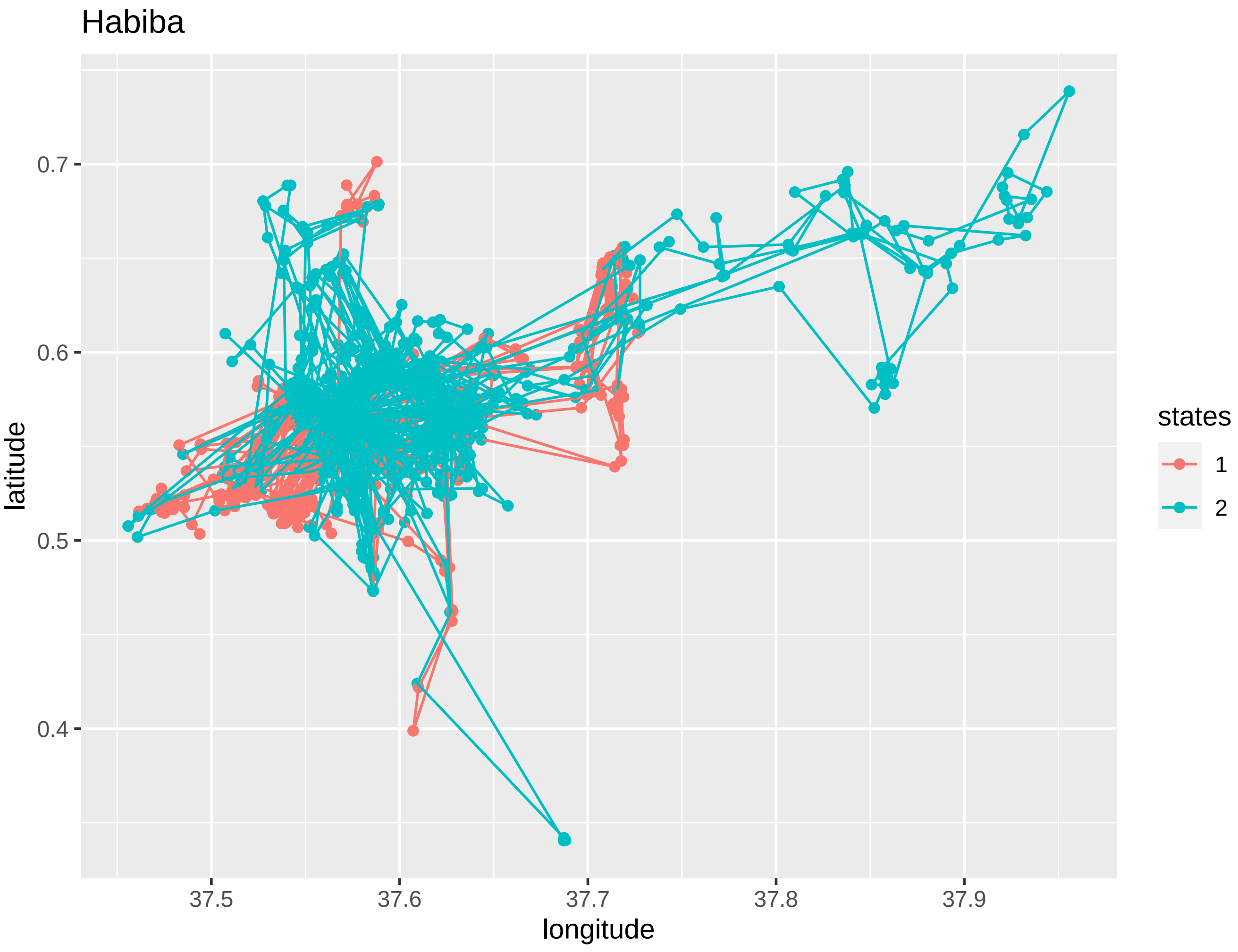

Habiba
0.7
0.6
0.5
0.4
latitude
37.5
37.6
37.7
37.8
37.9
longitude
states
1
2

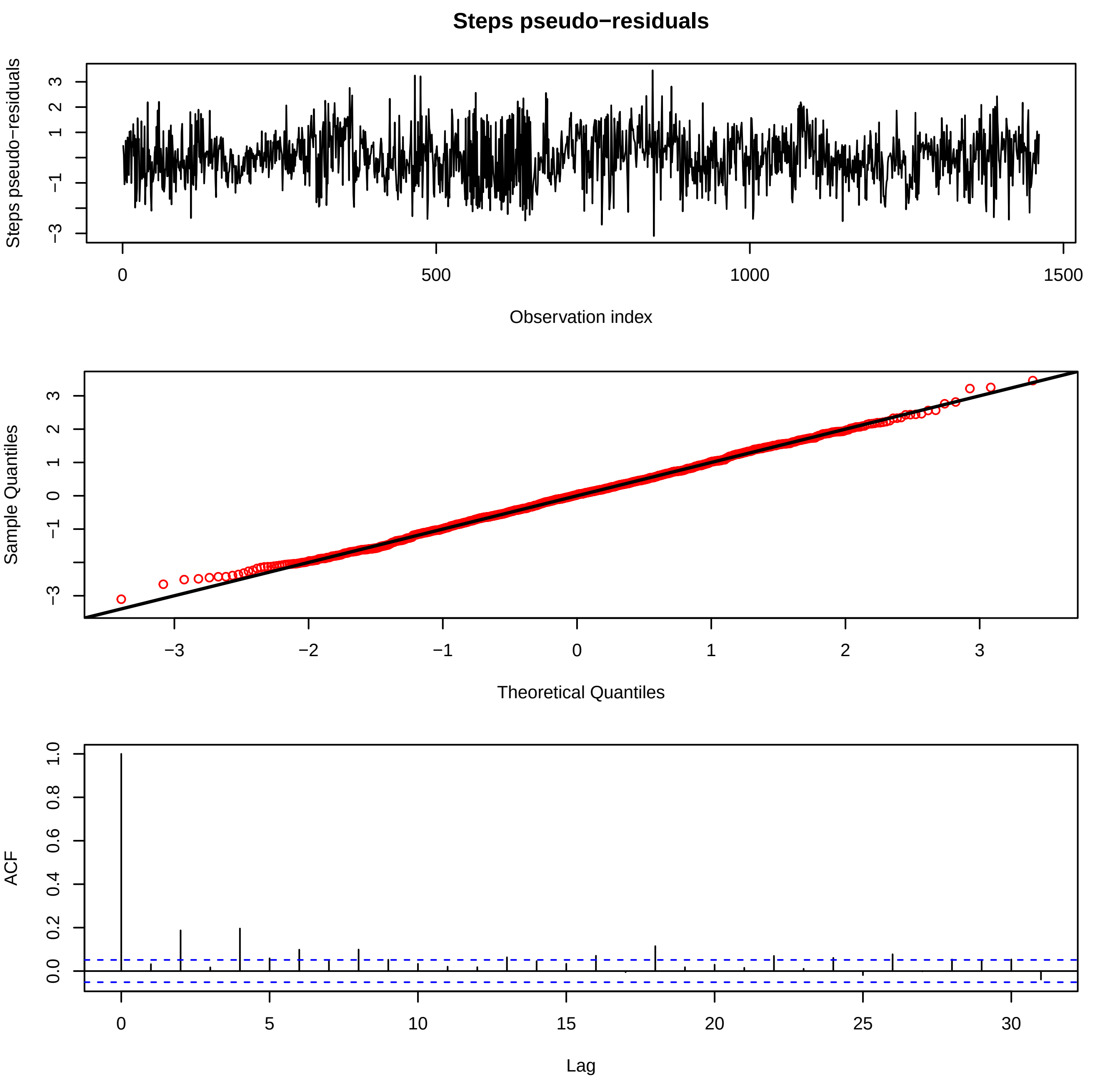
Steps pseudo−residuals
Steps pseudo−residuals
Observation index
Sample Quantiles
Theoretical Quantiles
ACF
Lag

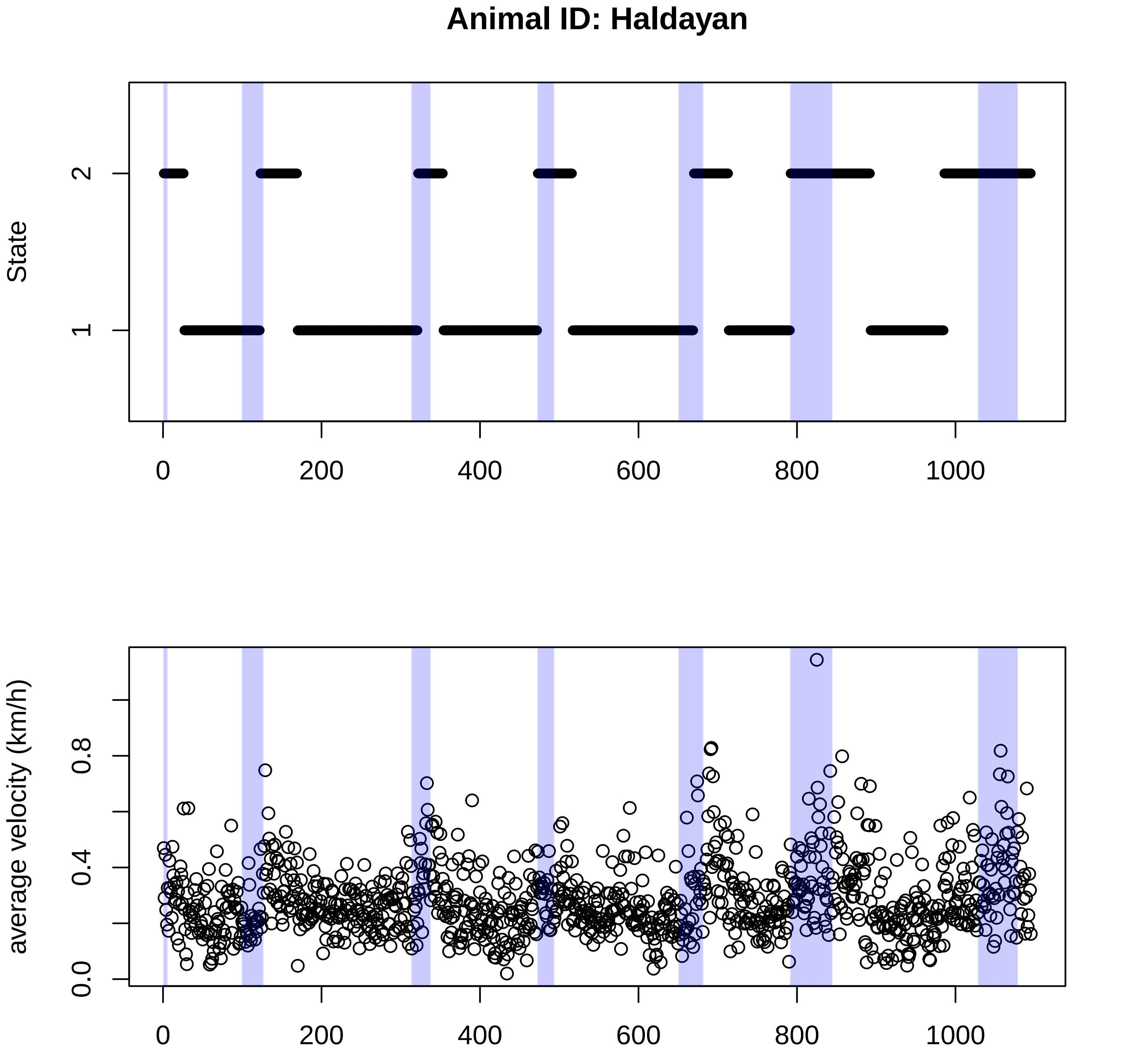

Animal ID: Haldayan
State
2
1
0
200
400
600
800
1000
average velocity (km/h)
0.0
0.4
0.8

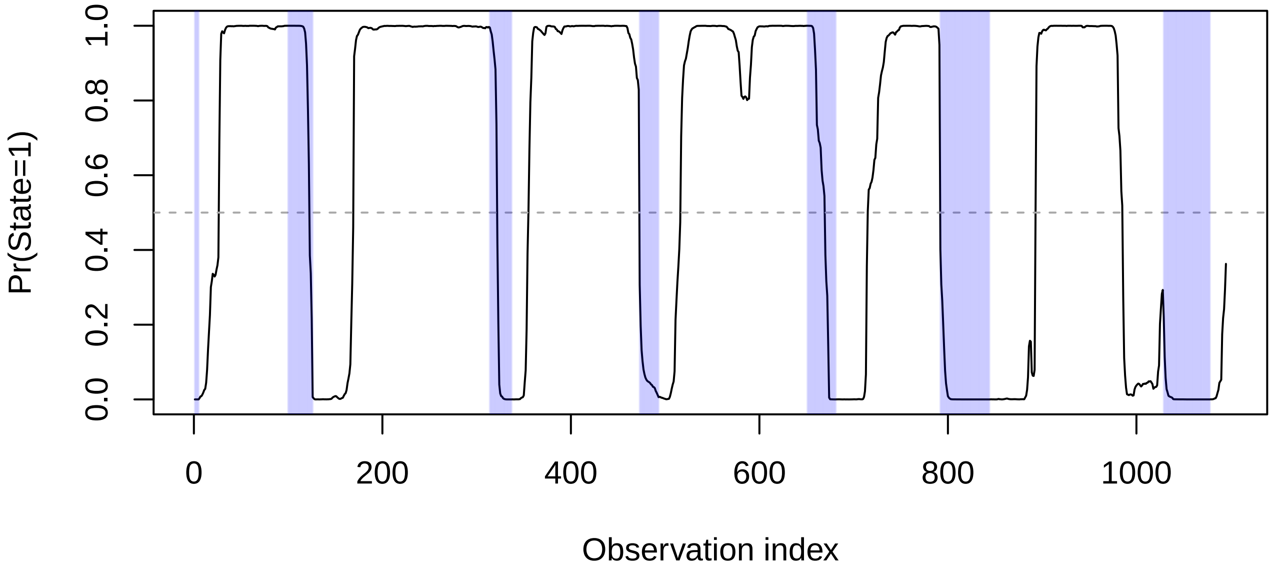

Pr(State=1)
0.0
0.2
0.4
0.6
0.8
1.0
0
200
400
600
800
1000
Observation index

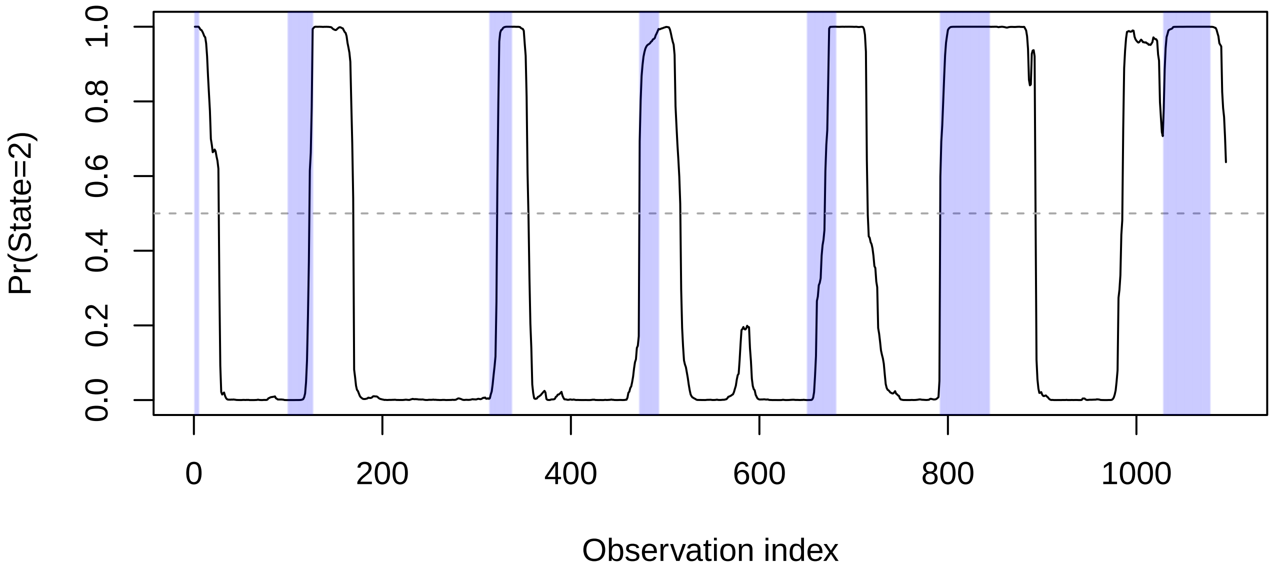

Pr(State=2)
0.0
0.2
0.4
0.6
0.8
1.0
0
200
400
600
800
1000
Observation index

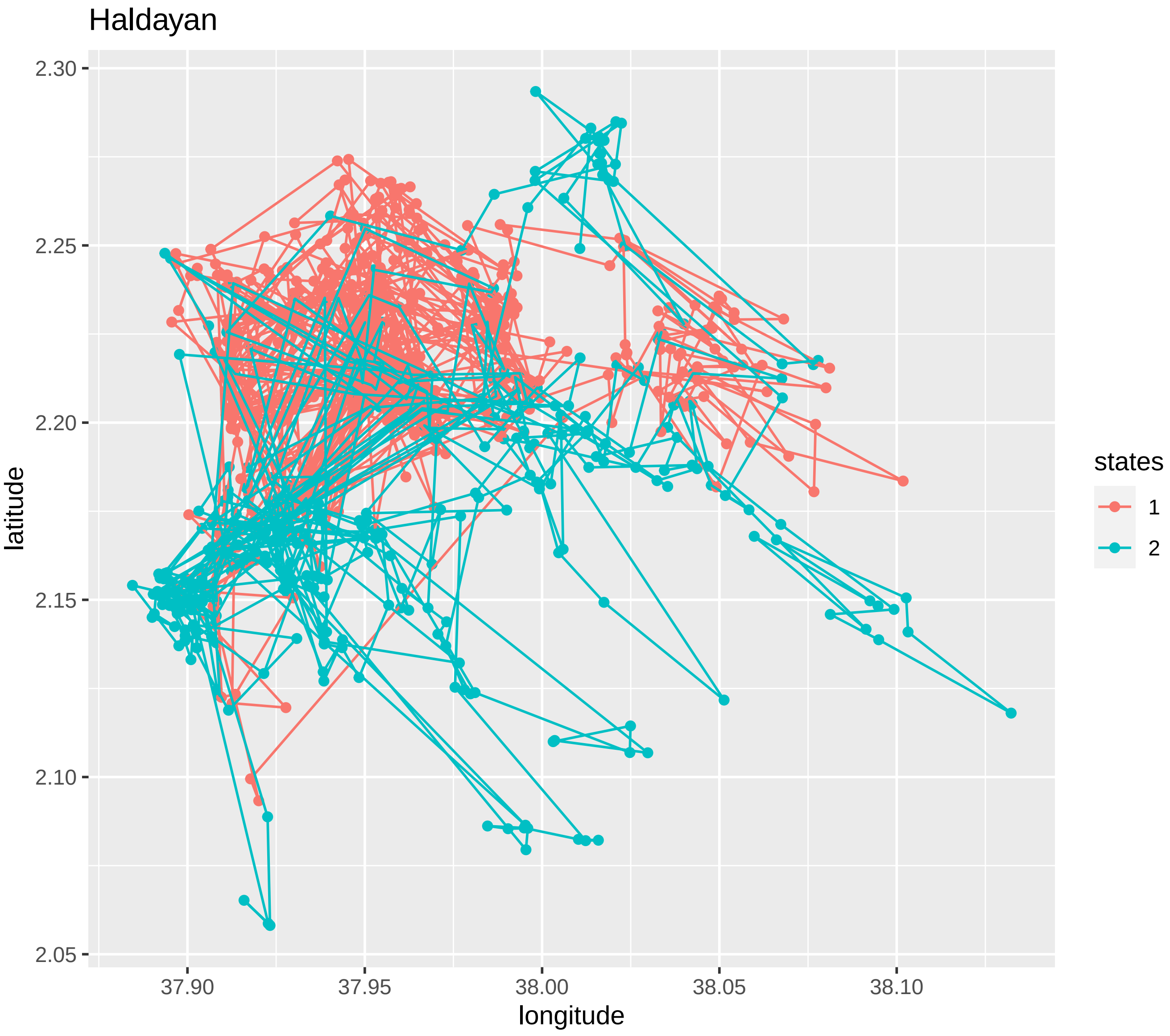

Haldayan
latitude
longitude
2.30
2.25
2.20
2.15
2.10
2.05
37.90
37.95
38.00
38.05
38.10
states
1
2

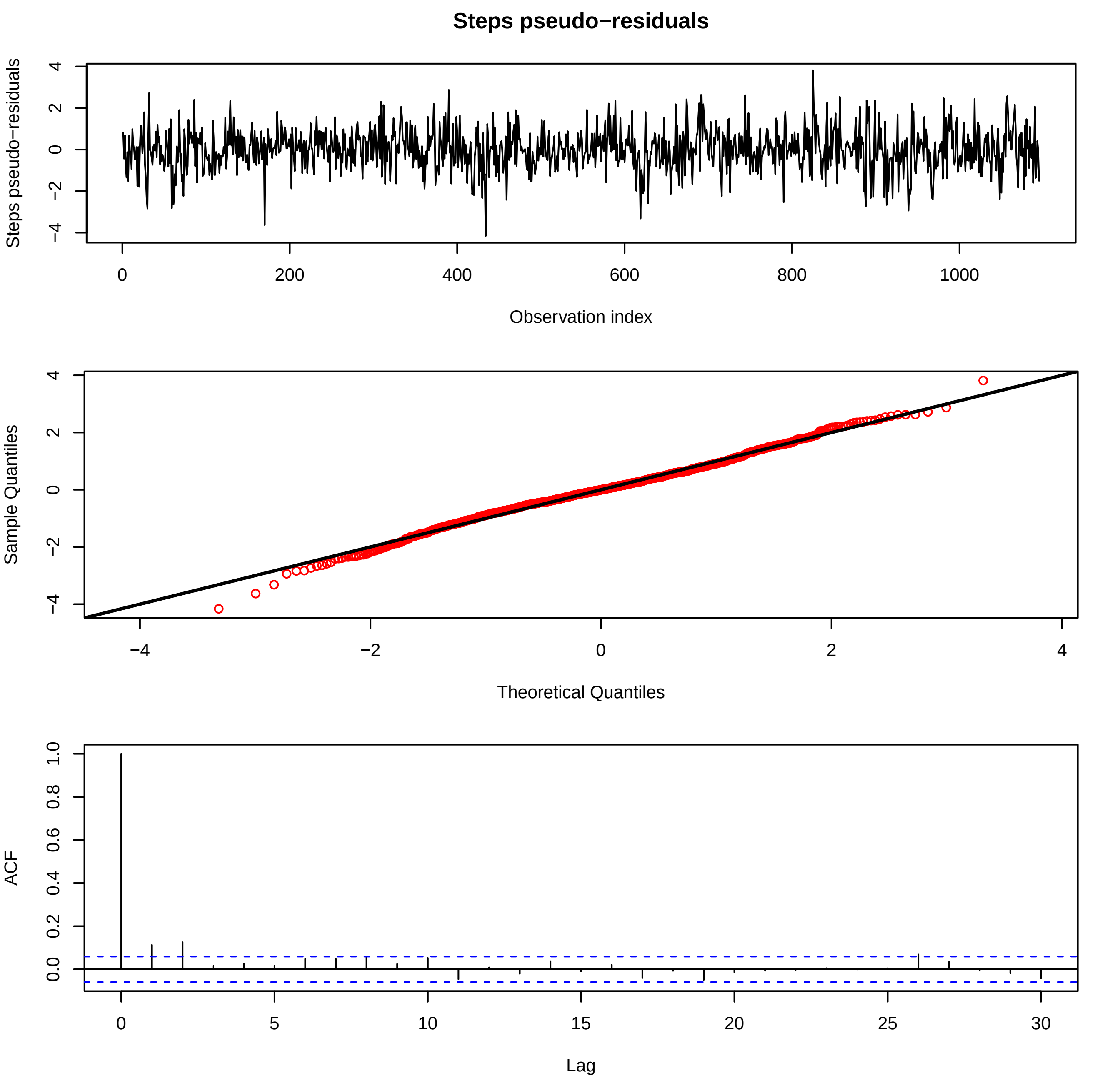

Steps pseudo−residuals
Steps pseudo−residuals
Observation index
Sample Quantiles
Theoretical Quantiles
ACF
Lag

Animal ID: Jessica_Samburu

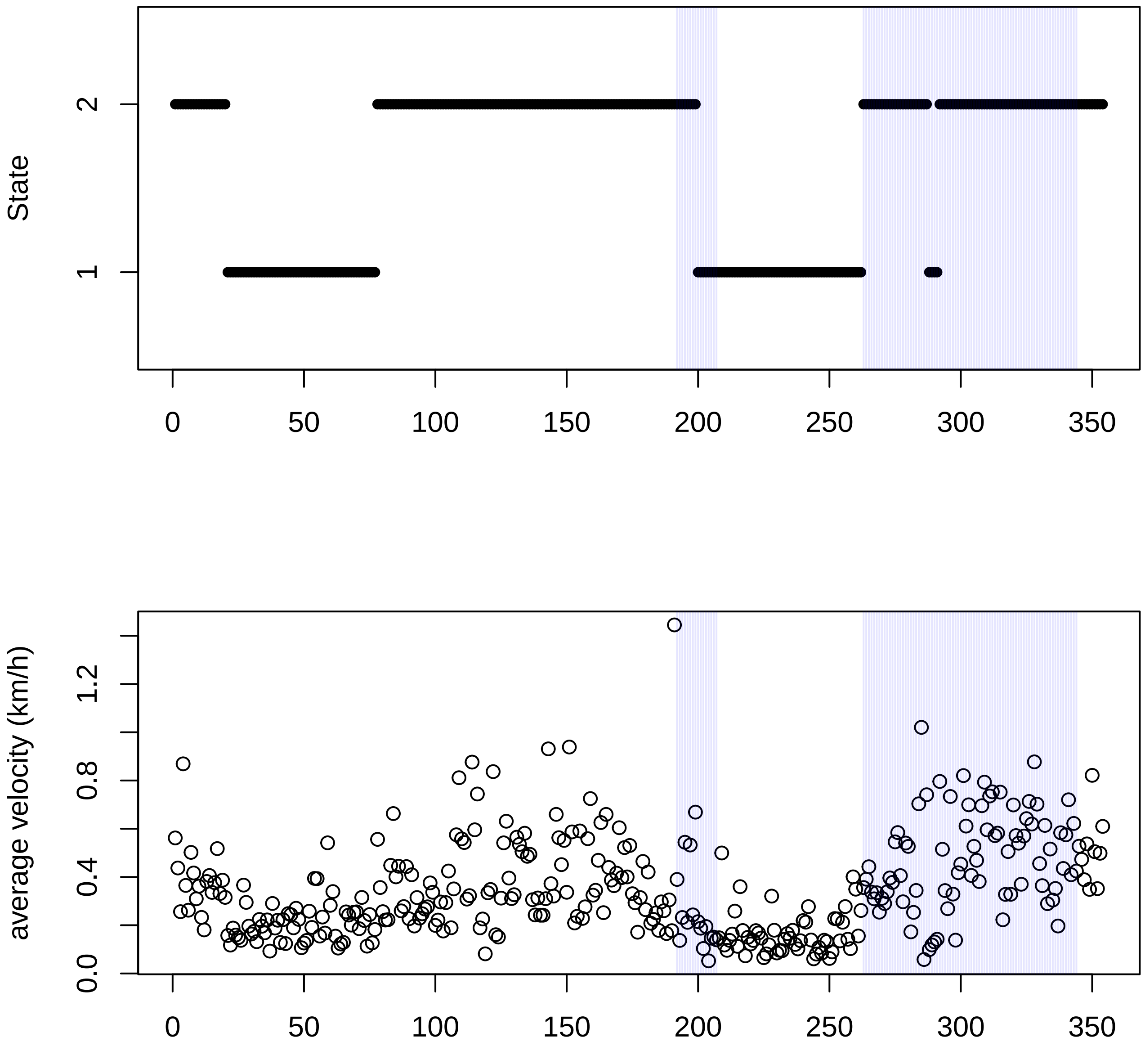

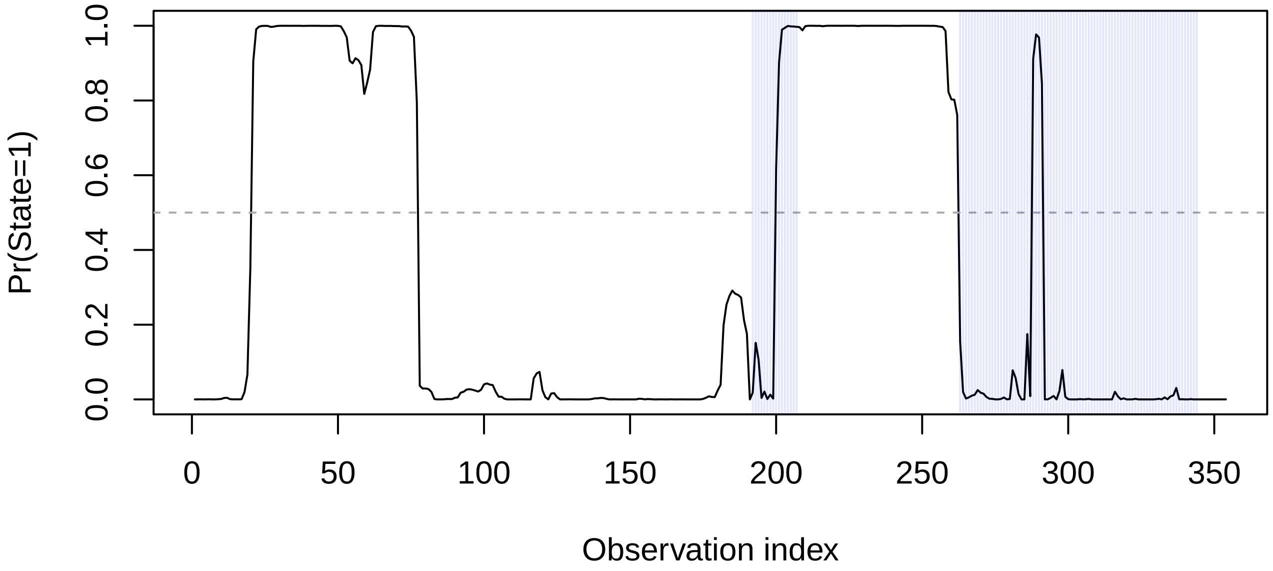

Pr(State=1)
1.0
0.8
0.6
0.4
0.2
0.0
0
50
100
150
200
250
300
350
Observation index

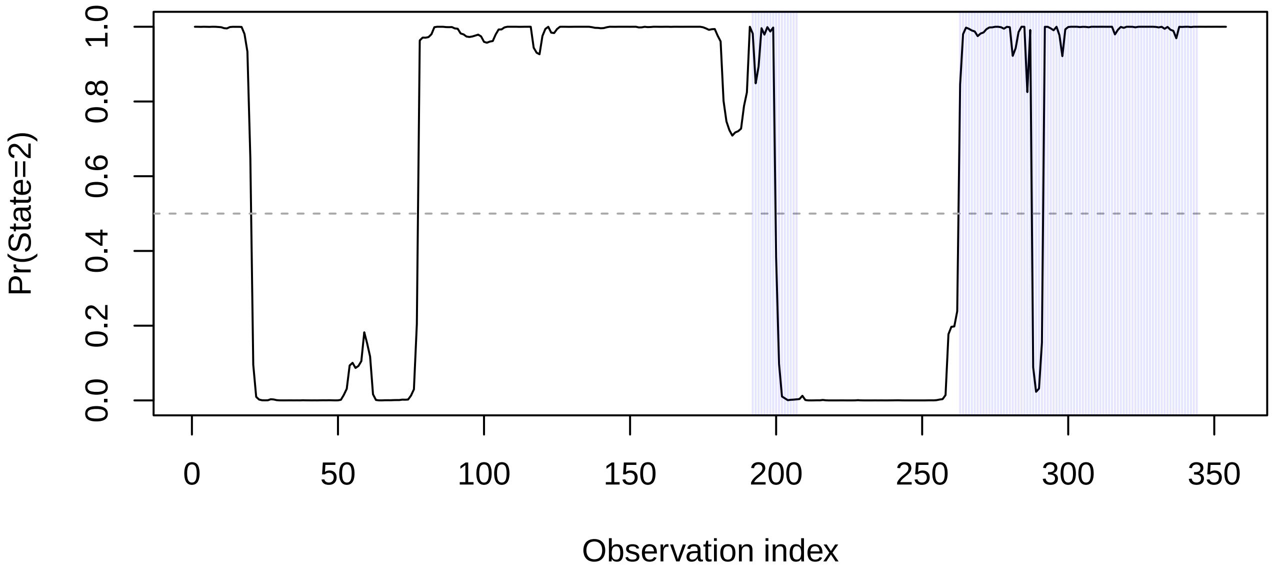

Pr(State=2)
1.0
0.8
0.6
0.4
0.2
0.0
0
50
100
150
200
250
300
350
Observation index

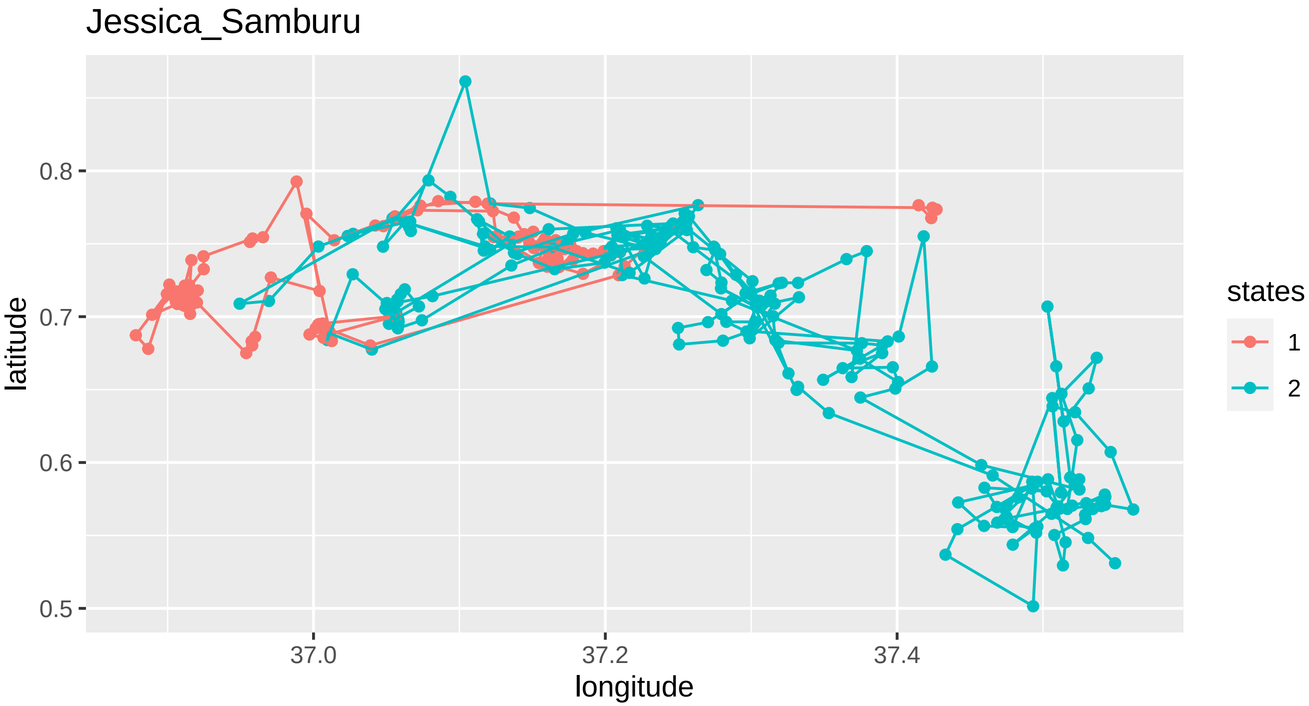

Jessica_Samburu
0.8
0.7
0.6
0.5
latitude
37.0
37.2
37.4
longitude
states
1
2

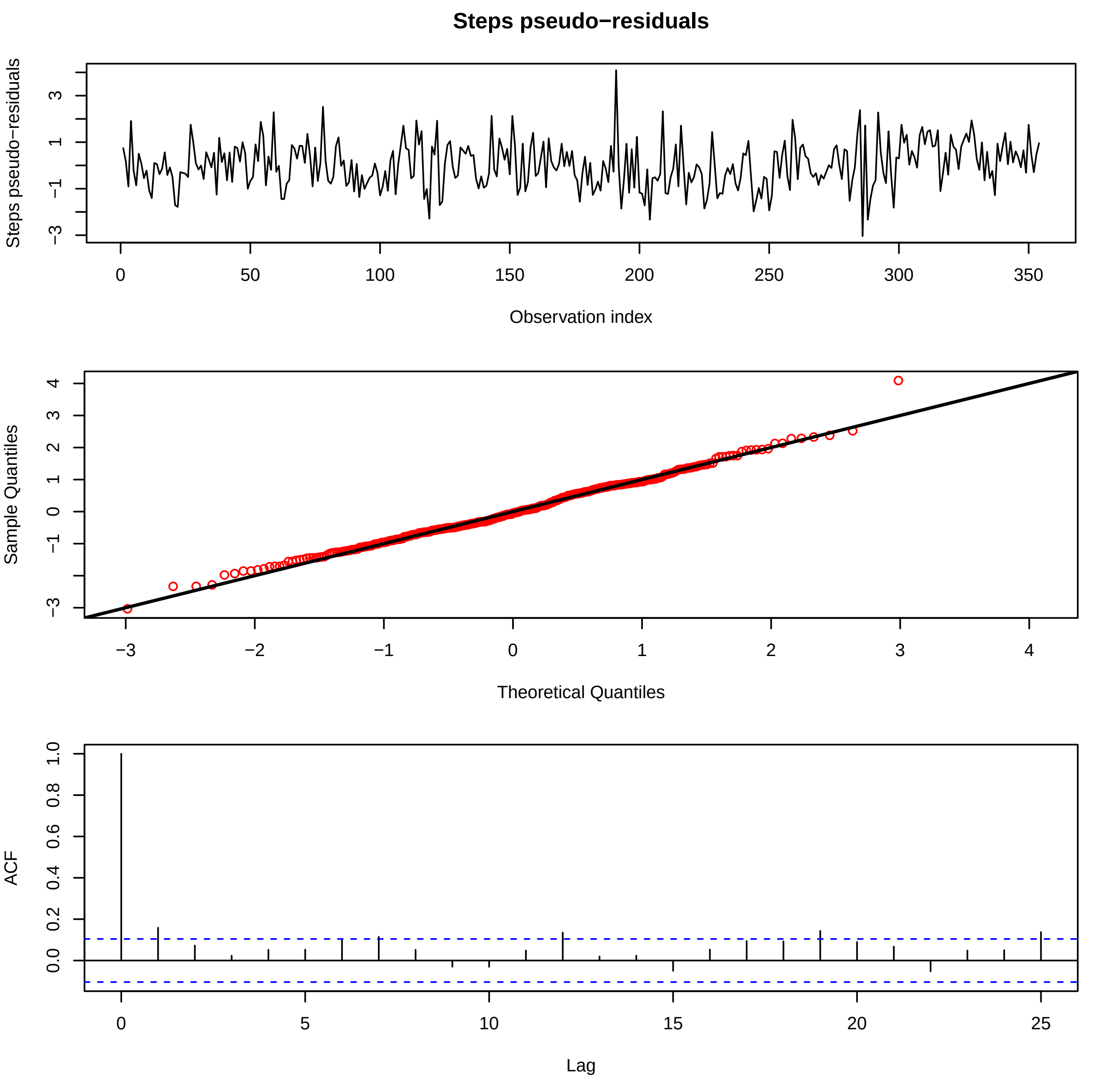
Steps pseudo−residuals
Steps pseudo−residuals
Observation index
Sample Quantiles
Theoretical Quantiles
ACF
Lag

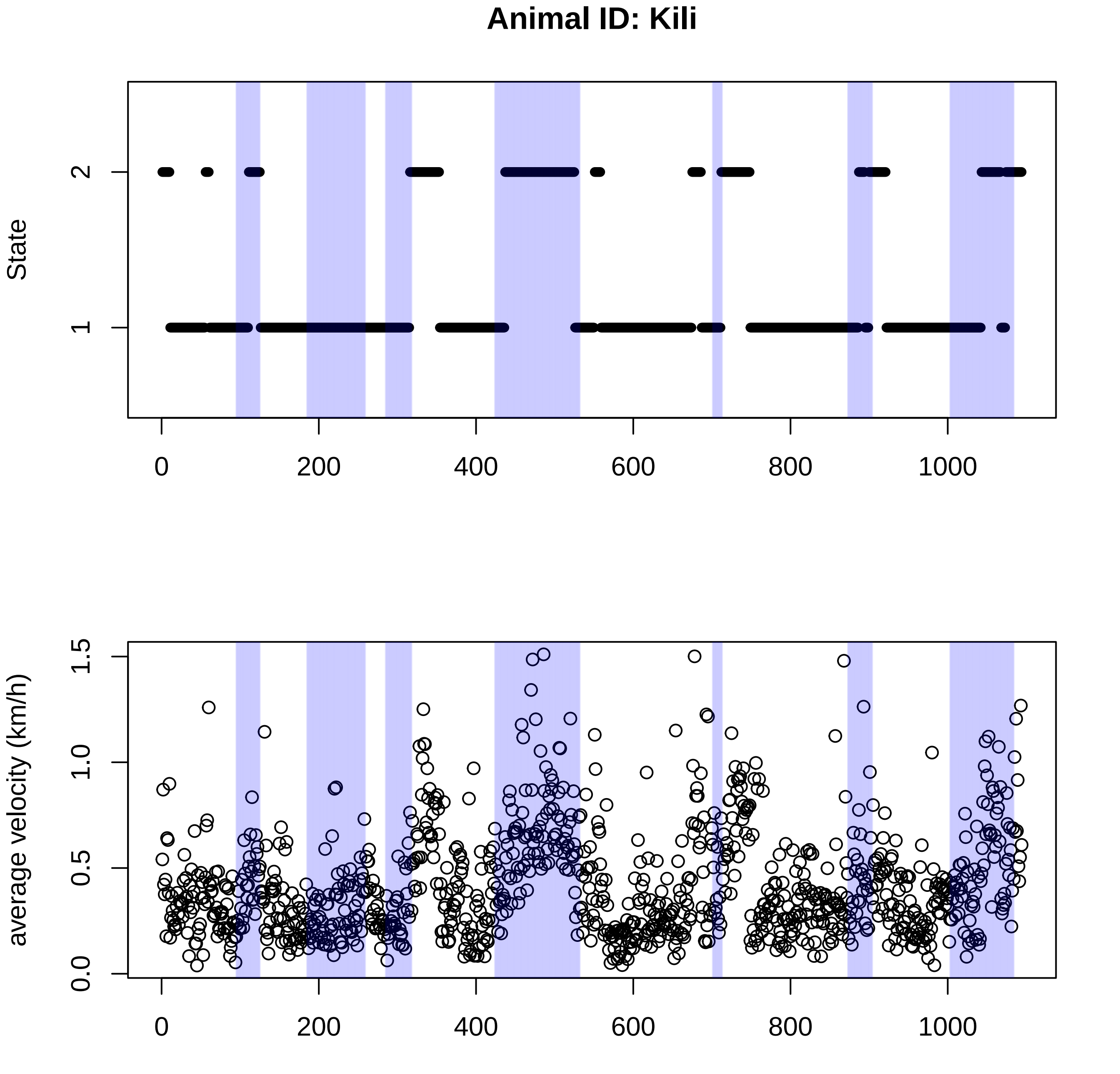

Animal ID: Kili
State
2
1
0
200
400
600
800
1000
average velocity (km/h)
1.5
1.0
0.5
0.0
0
200
400
600
800
1000

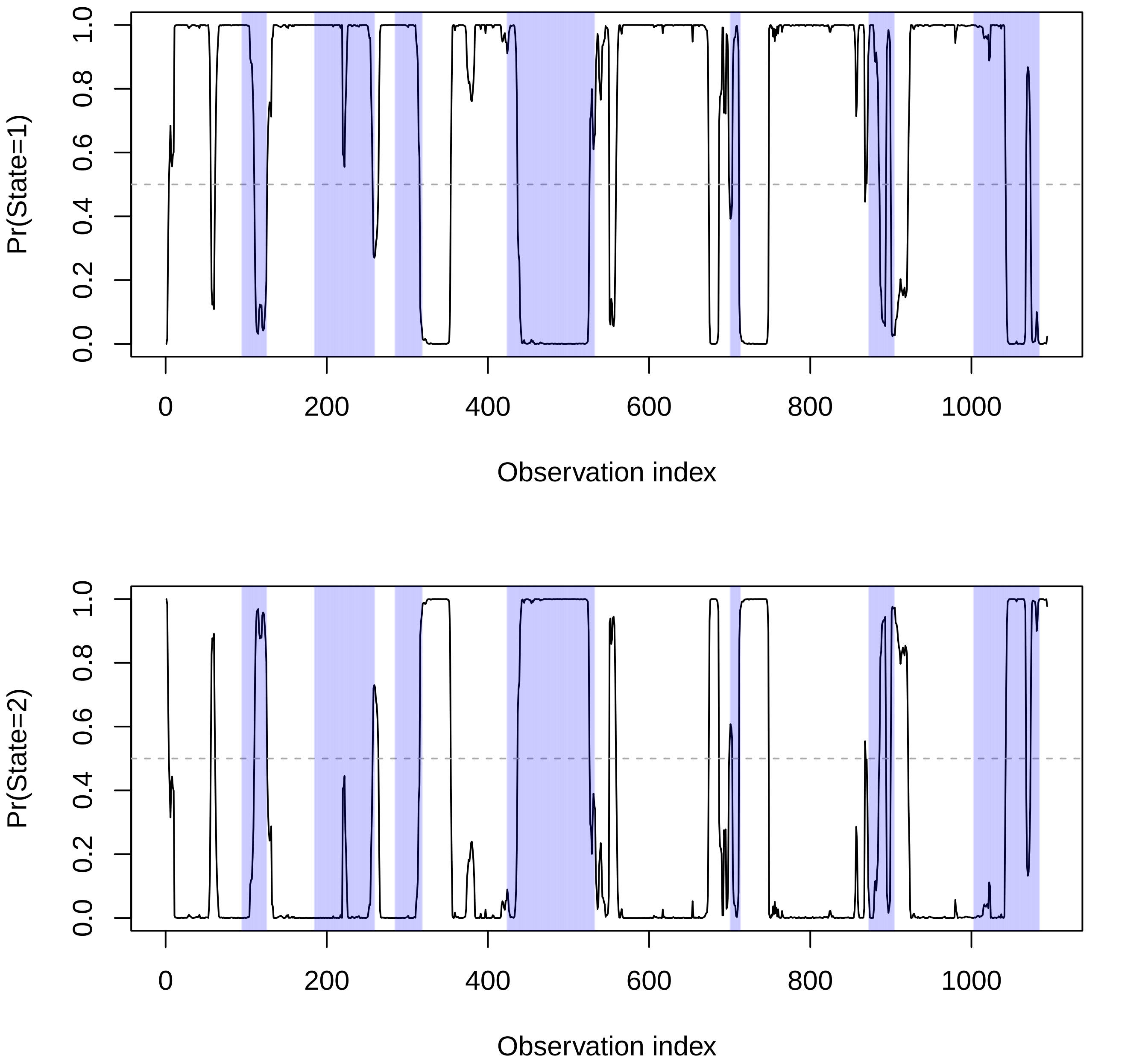
Pr(State=1)
1.0
0.8
0.6
0.4
0.2
0.0
0
200
400
600
800
1000
Observation index
Pr(State=2)
1.0
0.8
0.6
0.4
0.2
0.0
0
200
400
600
800
1000
Observation index

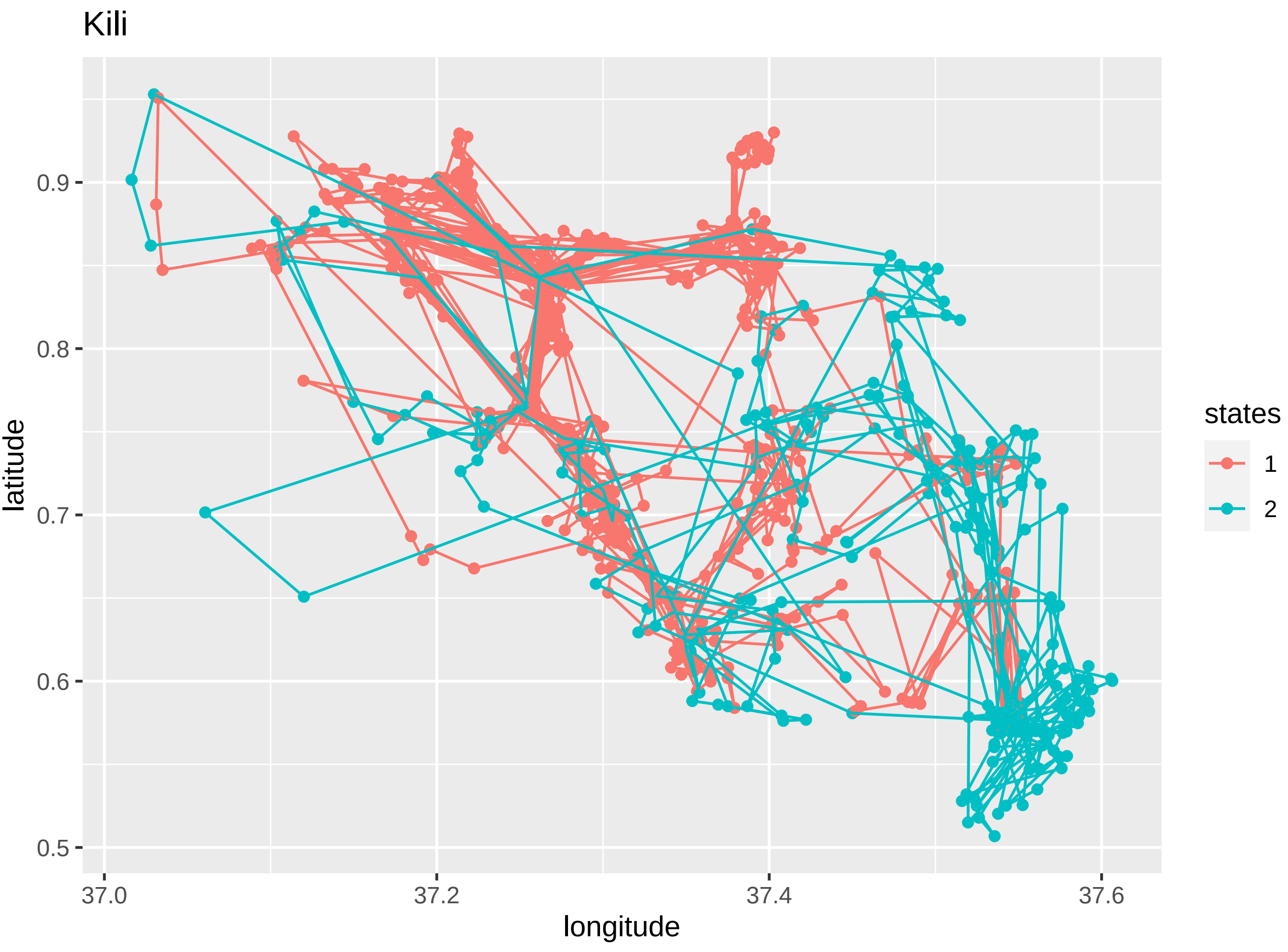

Kili
latitude
0.9
0.8
0.7
0.6
0.5
37.0
37.2
37.4
37.6
longitude
states
1
2

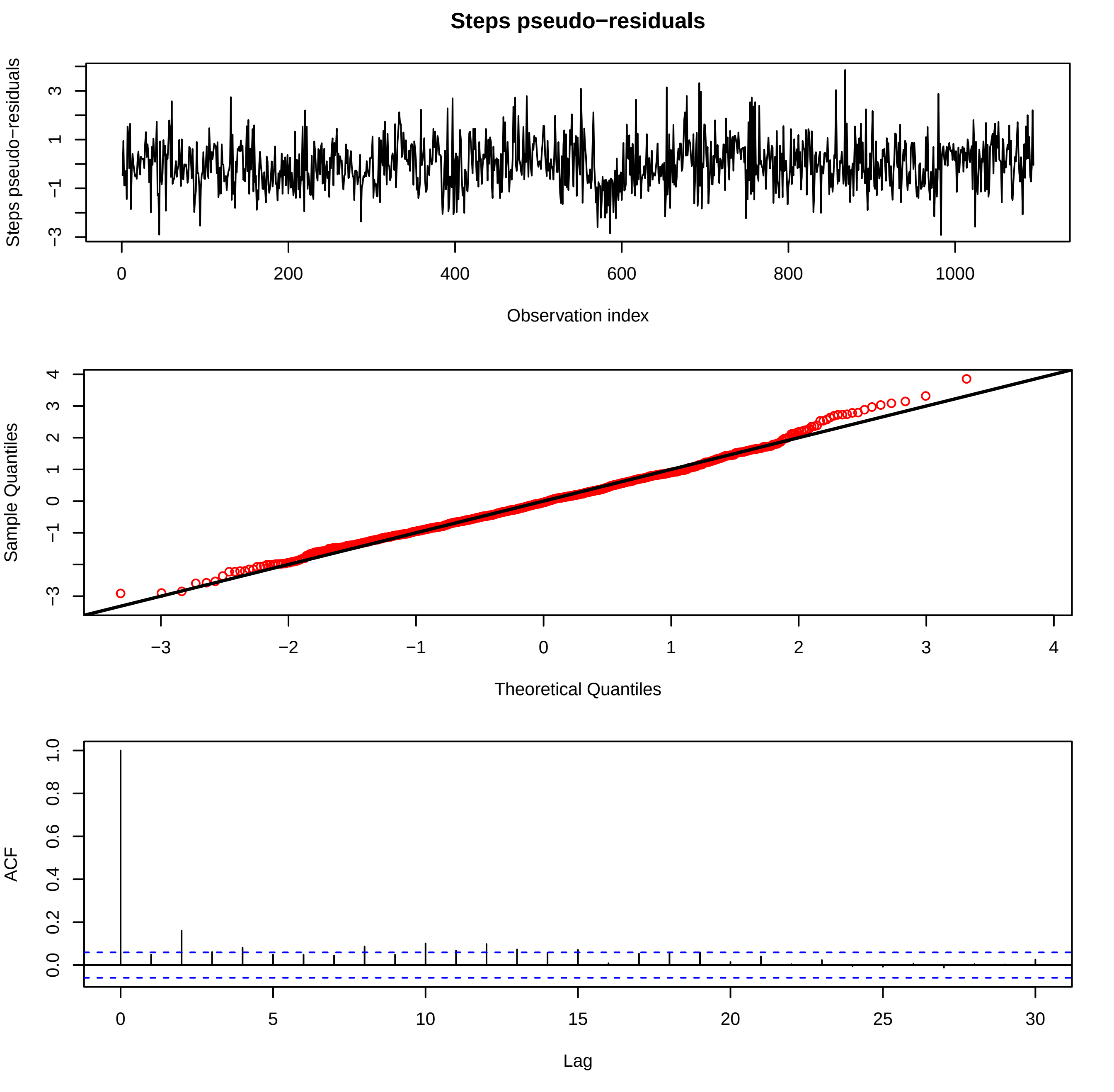
Steps pseudo−residuals
Steps pseudo−residuals
−3
−1
1
3
0
200
400
600
800
1000
Observation index
Sample Quantiles
−3
−1
0
1
2
3
4
−3
−2
−1
0
1
2
3
4
Theoretical Quantiles
ACF
0.0
0.2
0.4
0.6
0.8
1.0
0
5
10
15
20
25
30
Lag

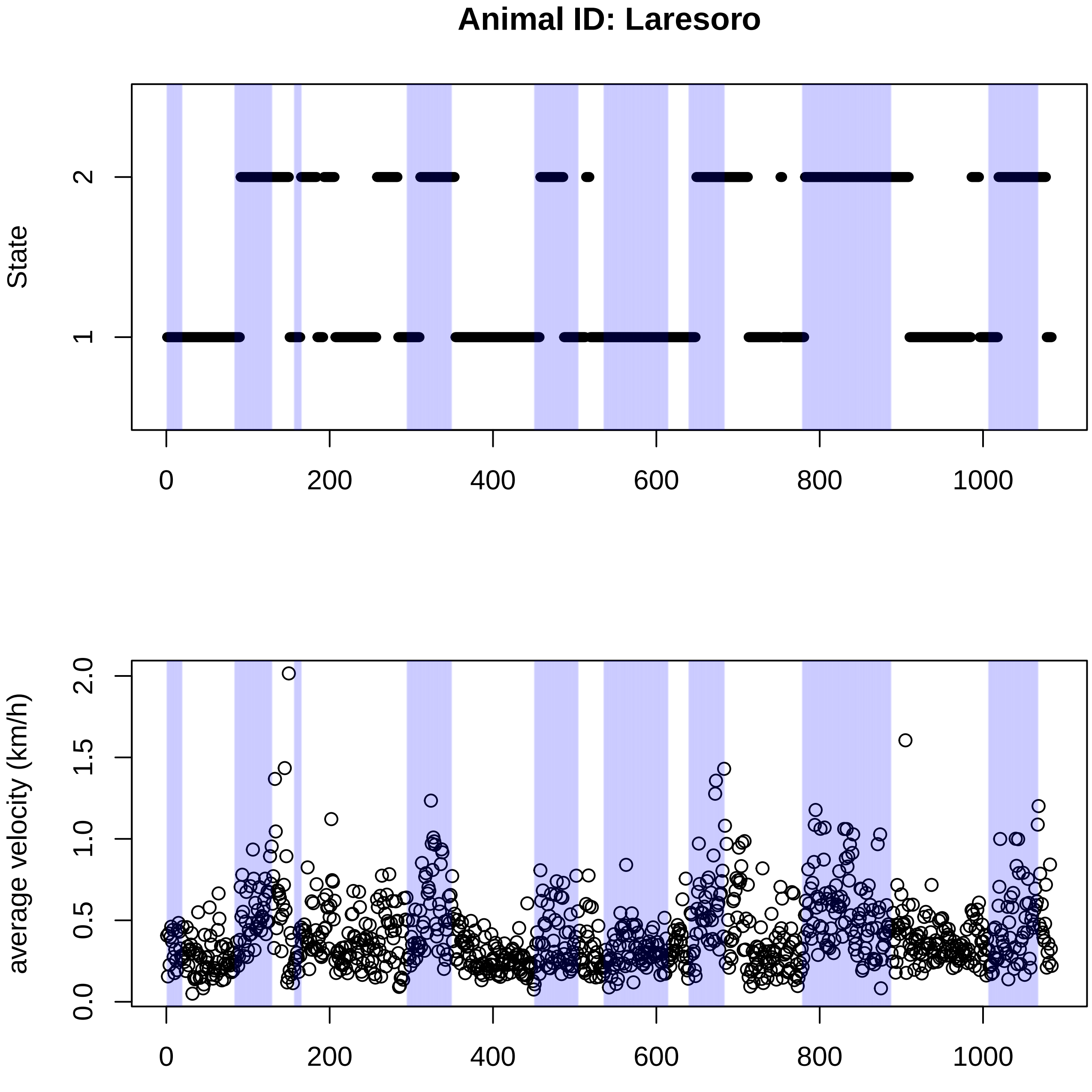

Animal ID: Laresoro
State
2
1
0
200
400
600
800
1000
average velocity (km/h)
0.0
0.5
1.0
1.5
2.0
0
200
400
600
800
1000

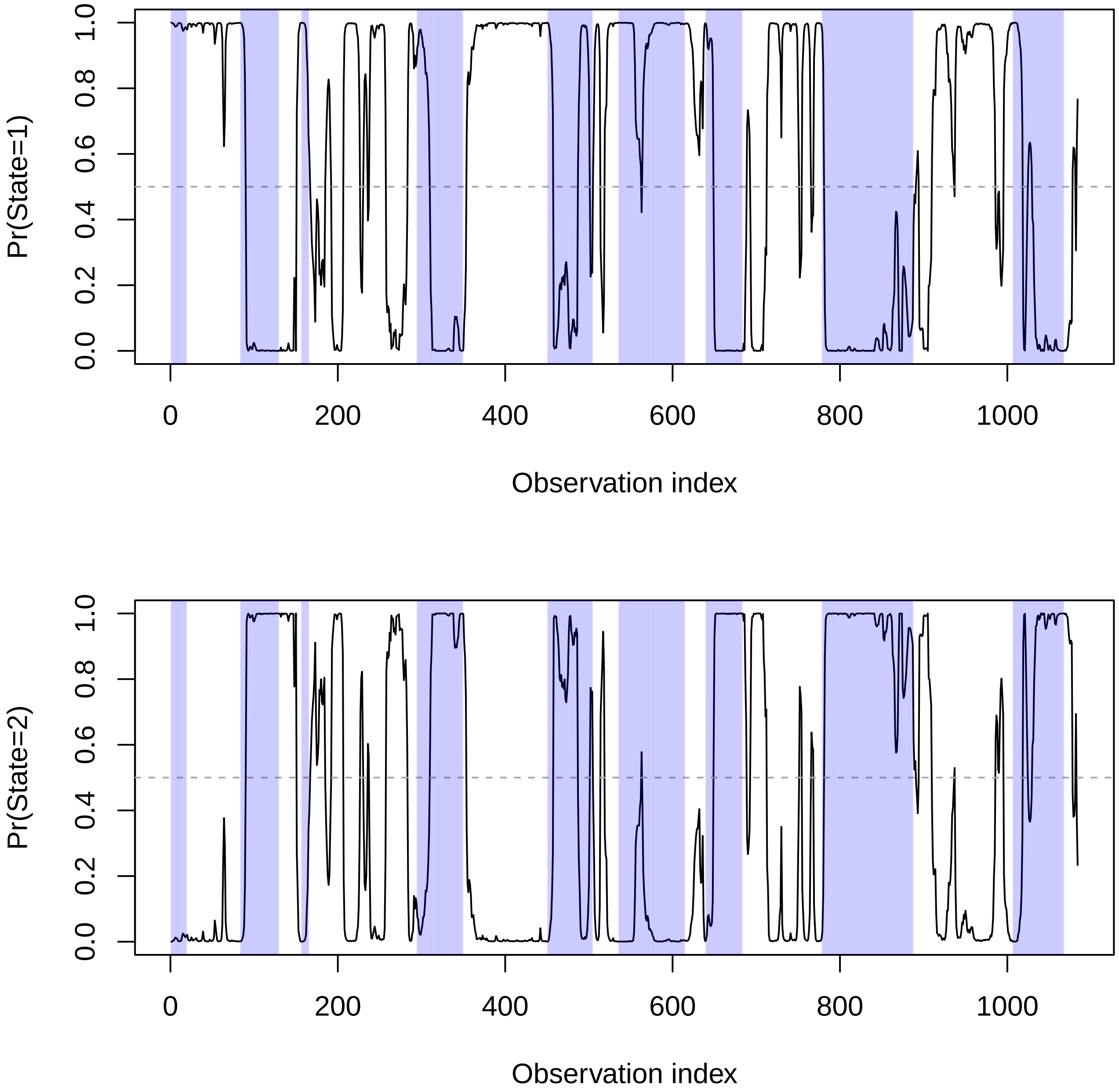
Pr(State=1)
0.0
0.2
0.4
0.6
0.8
1.0
0
200
400
600
800
1000
Observation index
Pr(State=2)
0.0
0.2
0.4
0.6
0.8
1.0
0
200
400
600
800
1000
Observation index

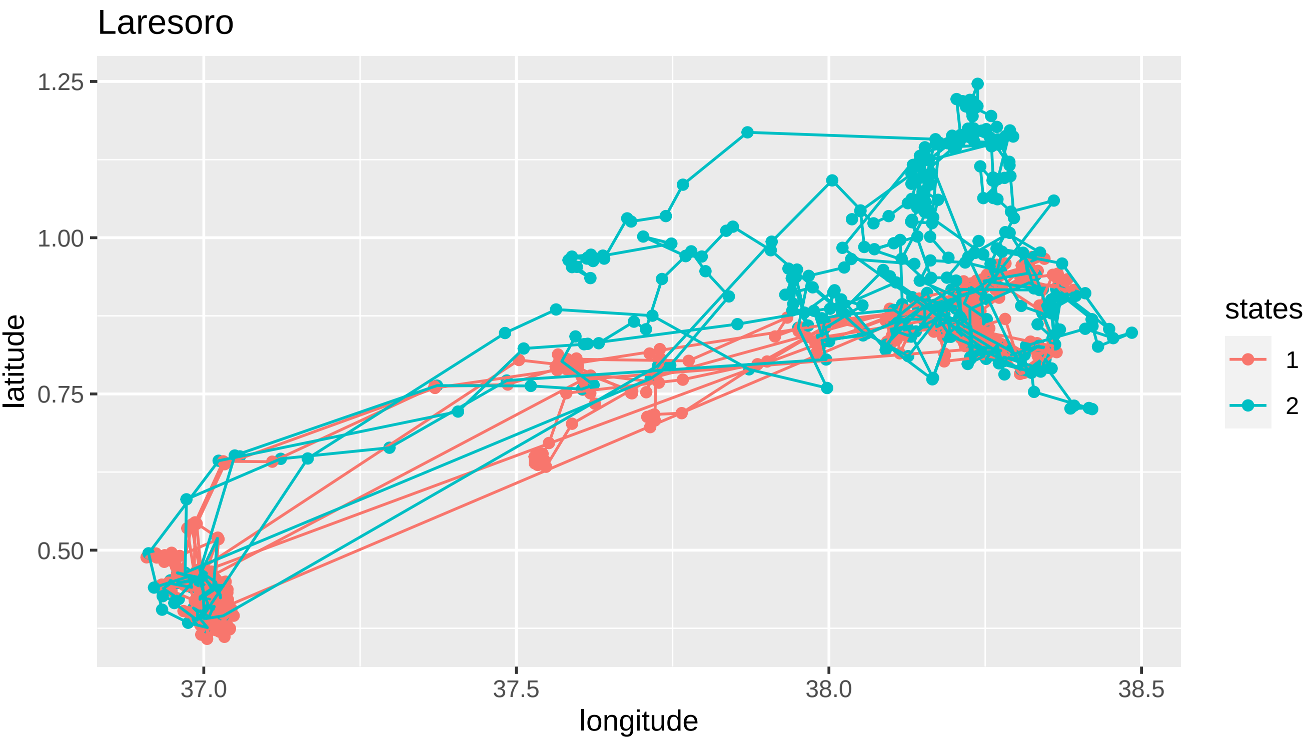

Laresoro
1.25
1.00
0.75
0.50
latitude
37.0
37.5
38.0
38.5
longitude
states
1
2

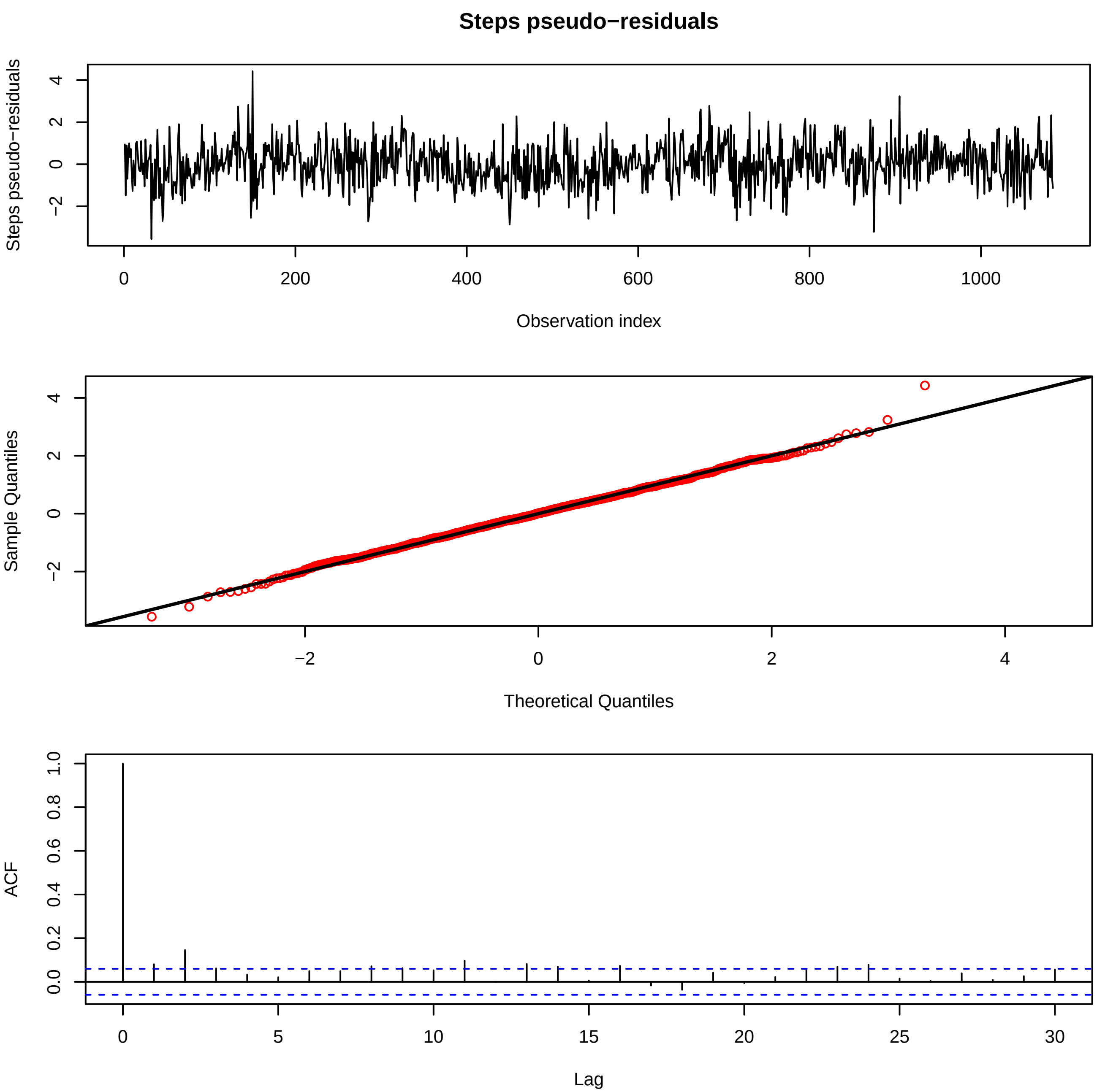
Steps pseudo−residuals
Steps pseudo−residuals
Observation index
Sample Quantiles
Theoretical Quantiles
ACF
Lag

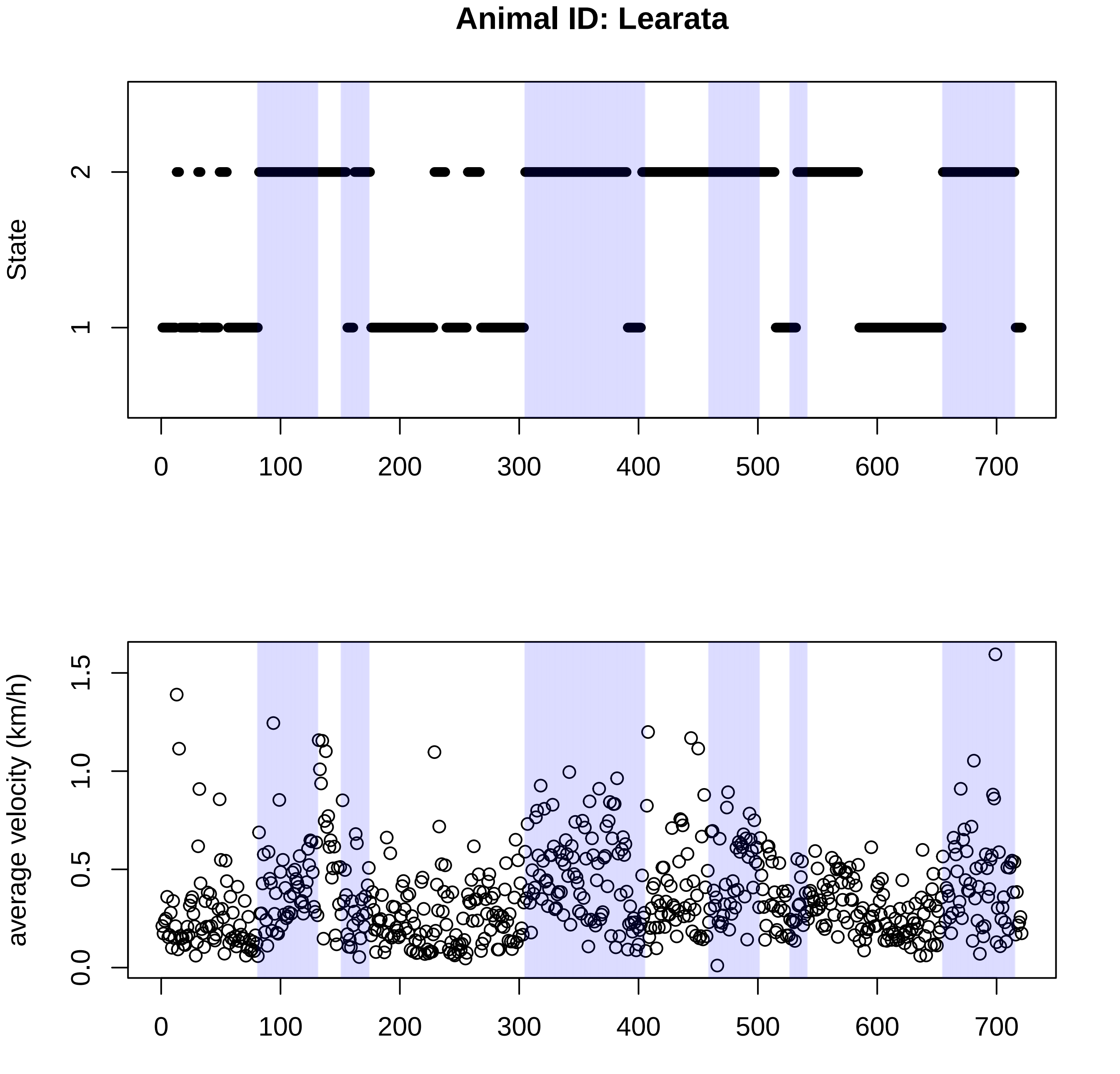

Animal ID: Learata
State
2
1
0
100
200
300
400
500
600
700
average velocity (km/h)
0.0
0.5
1.0
1.5

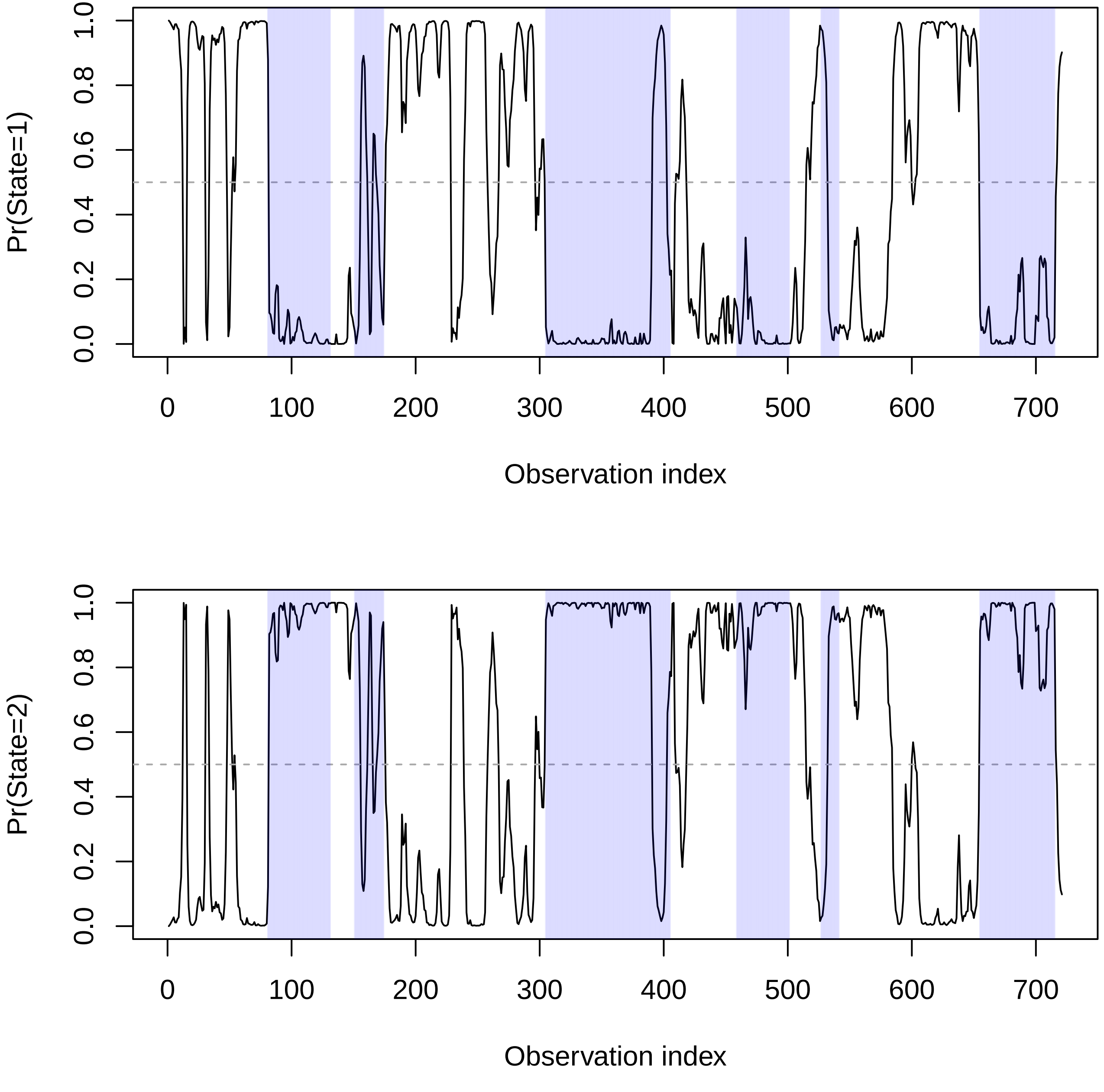

Pr(State=1)
0.0
0.2
0.4
0.6
0.8
1.0
0
100
200
300
400
500
600
700
Observation index
Pr(State=2)
0.0
0.2
0.4
0.6
0.8
1.0
0
100
200
300
400
500
600
700
Observation index

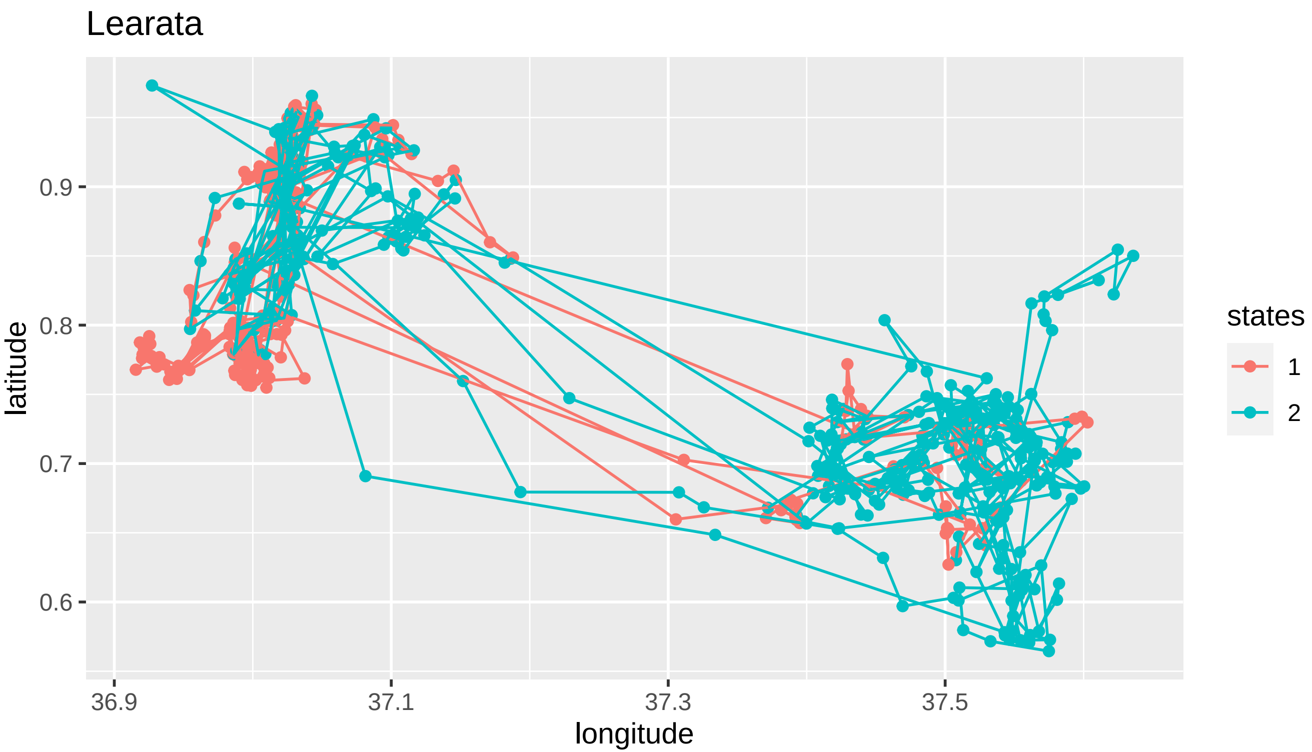

Learata
0.9
0.8
0.7
0.6
latitude
36.9
37.1
37.3
37.5
longitude
states
1
2

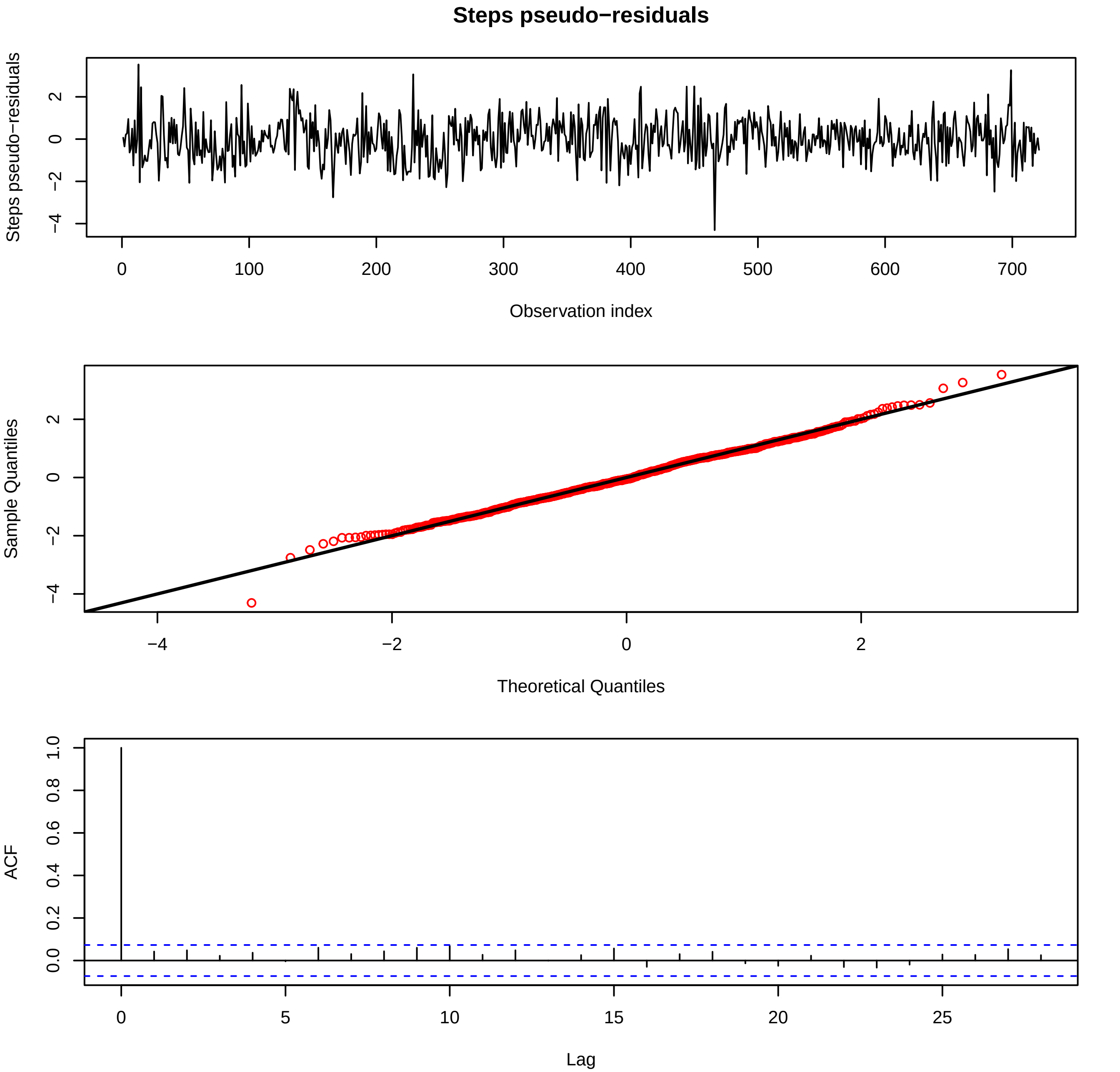

Steps pseudo−residuals
Steps pseudo−residuals
Observation index
Sample Quantiles
Theoretical Quantiles
ACF
Lag

Animal ID: Luna

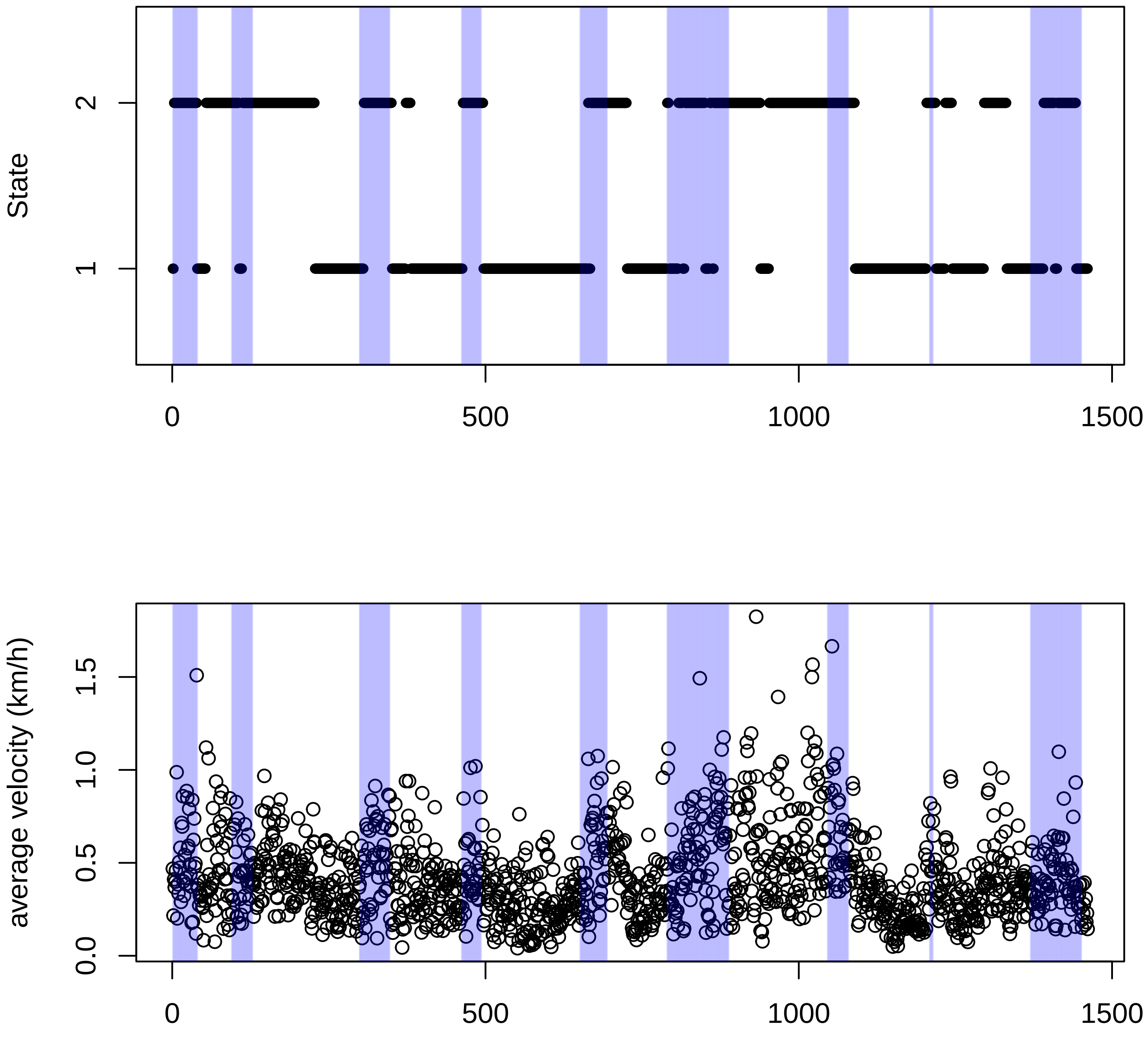

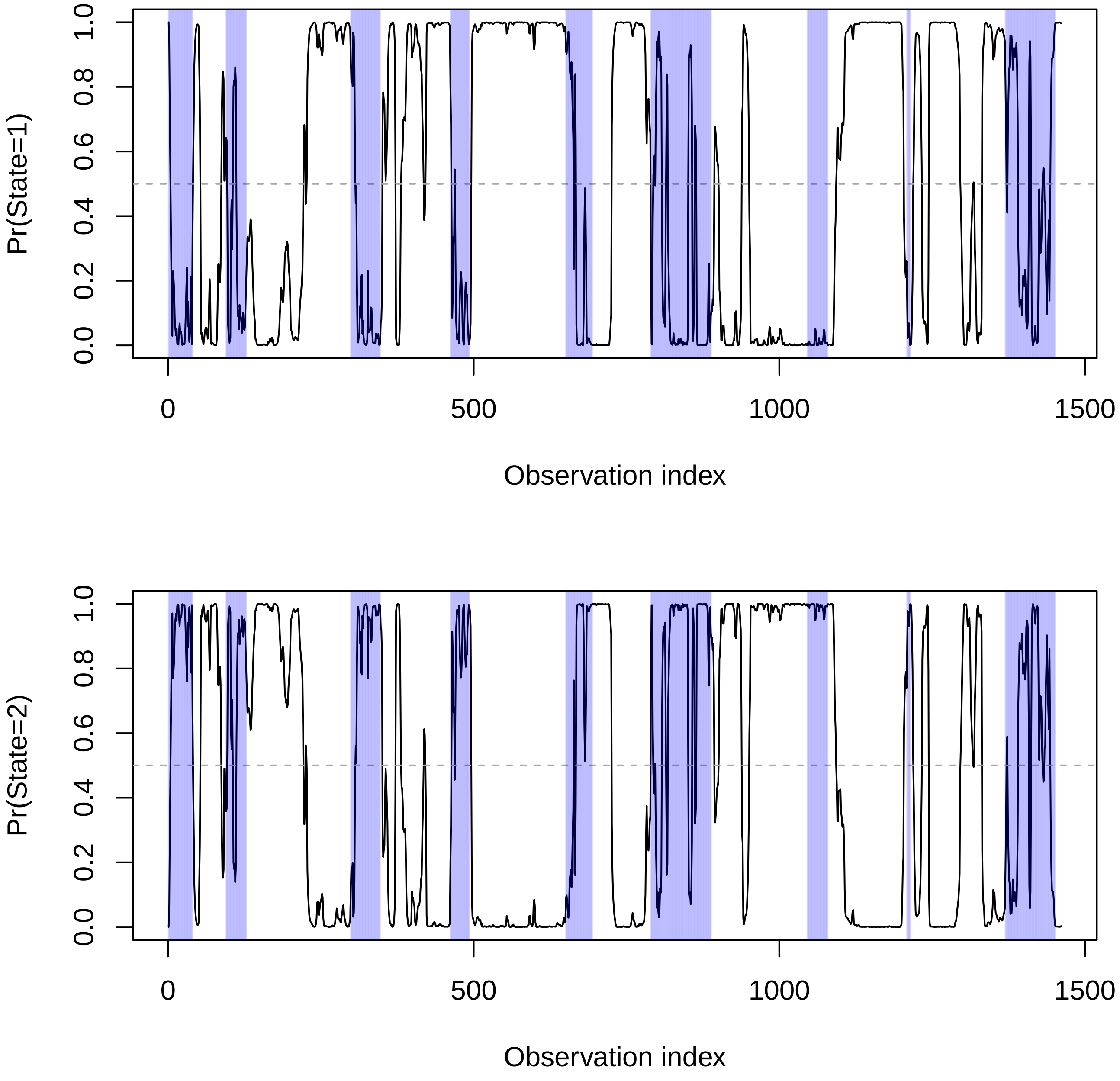

Pr(State=1)
0.0
0.2
0.4
0.6
0.8
1.0
0
500
1000
1500
Observation index
Pr(State=2)
0.0
0.2
0.4
0.6
0.8
1.0
0
500
1000
1500
Observation index

Luna

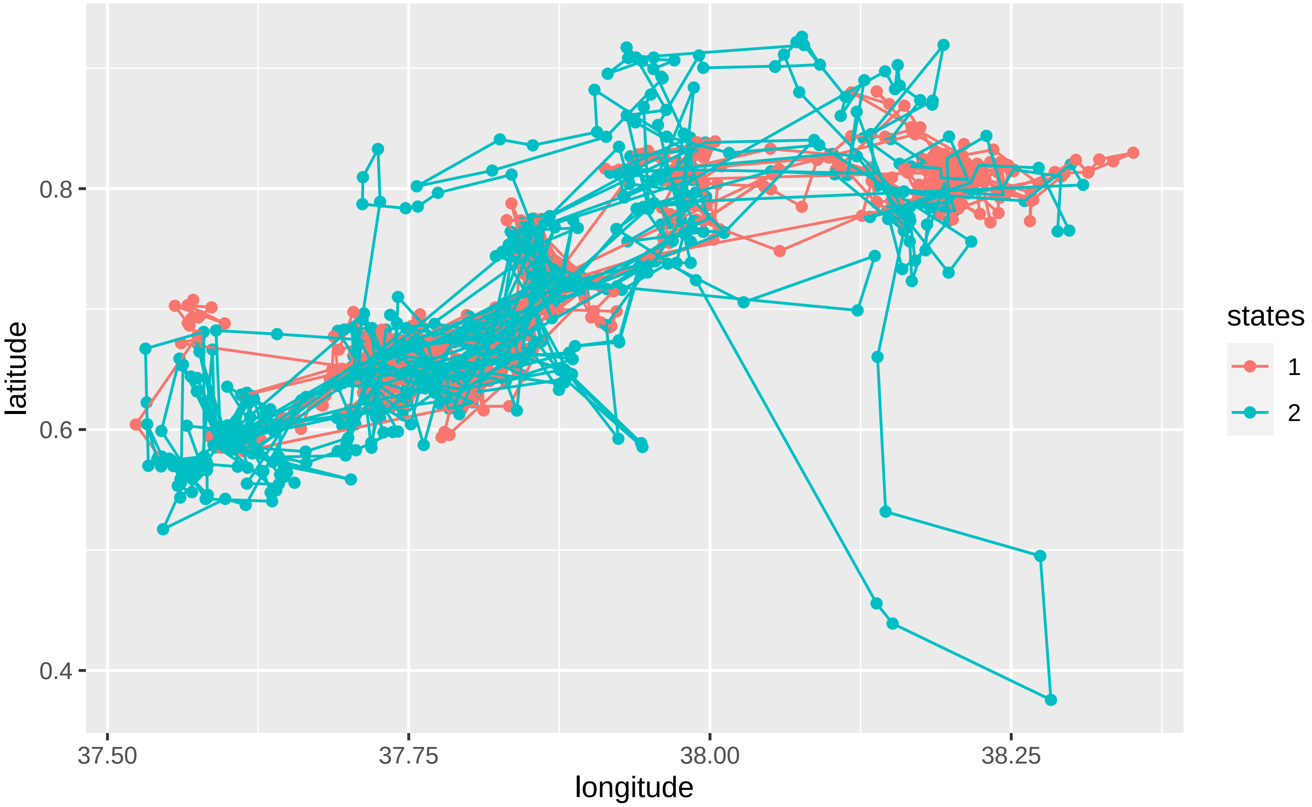

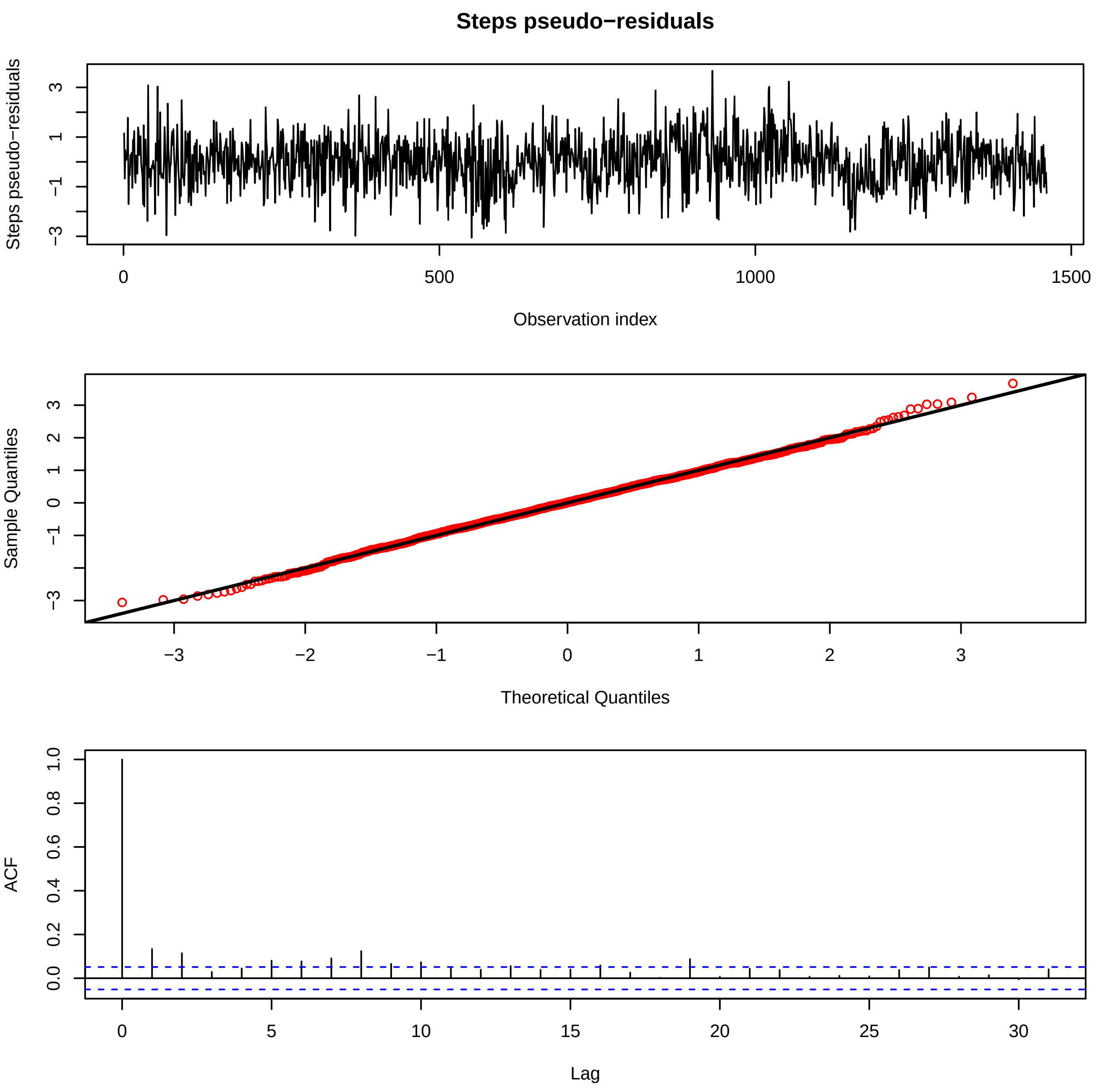
Steps pseudo−residuals
Steps pseudo−residuals
Observation index
Sample Quantiles
Theoretical Quantiles
ACF
Lag

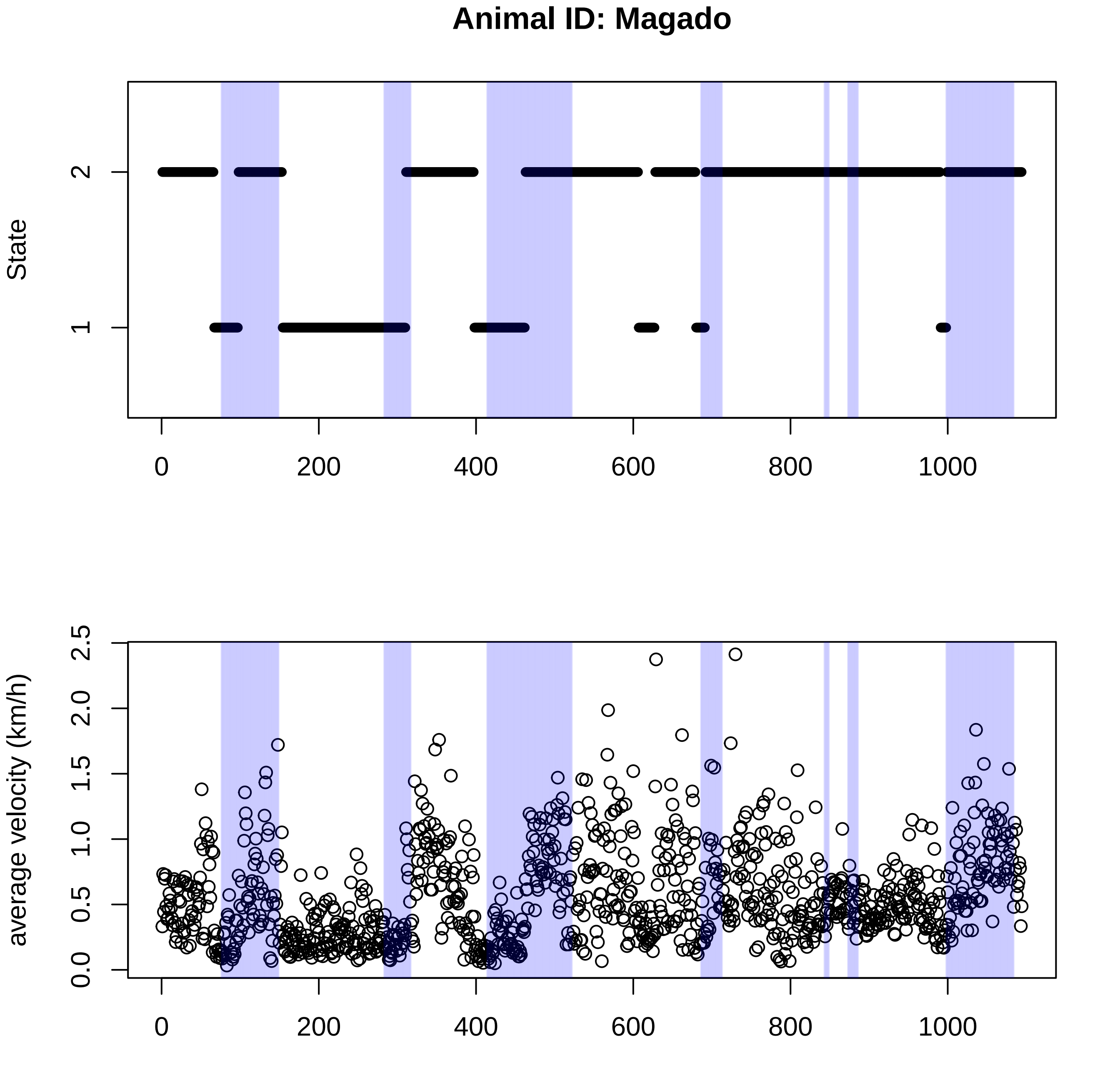

Animal ID: Magado
State
2
1
0
200
400
600
800
1000
average velocity (km/h)
0.0
0.5
1.0
1.5
2.0
2.5
0
200
400
600
800
1000

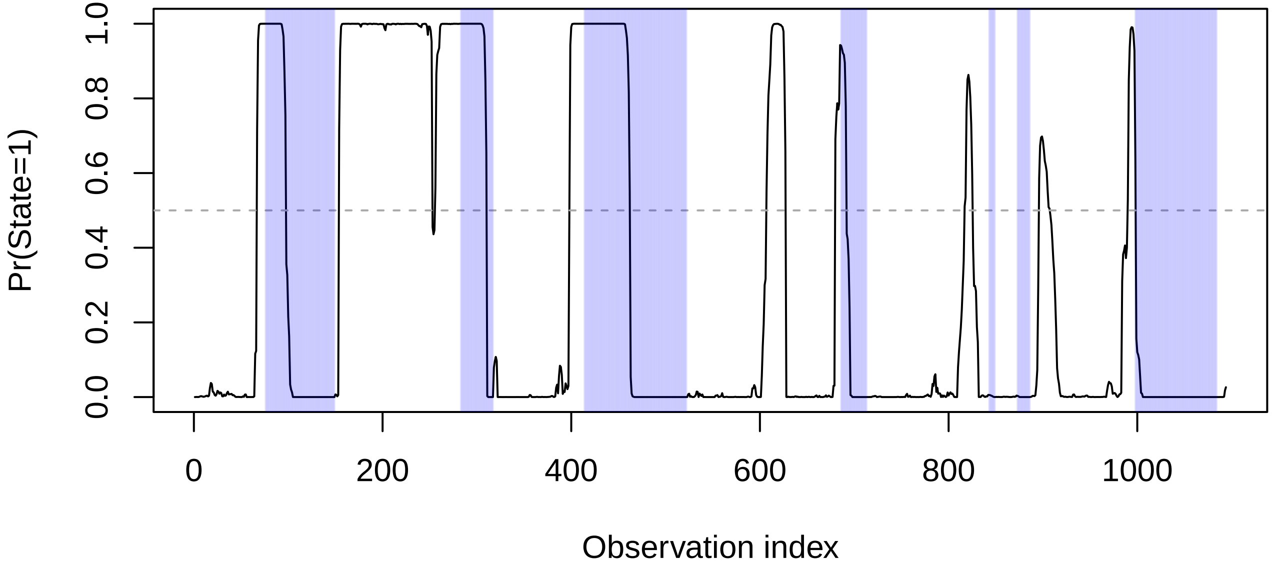

Pr(State=1)
0.0
0.2
0.4
0.6
0.8
1.0
0
200
400
600
800
1000
Observation index

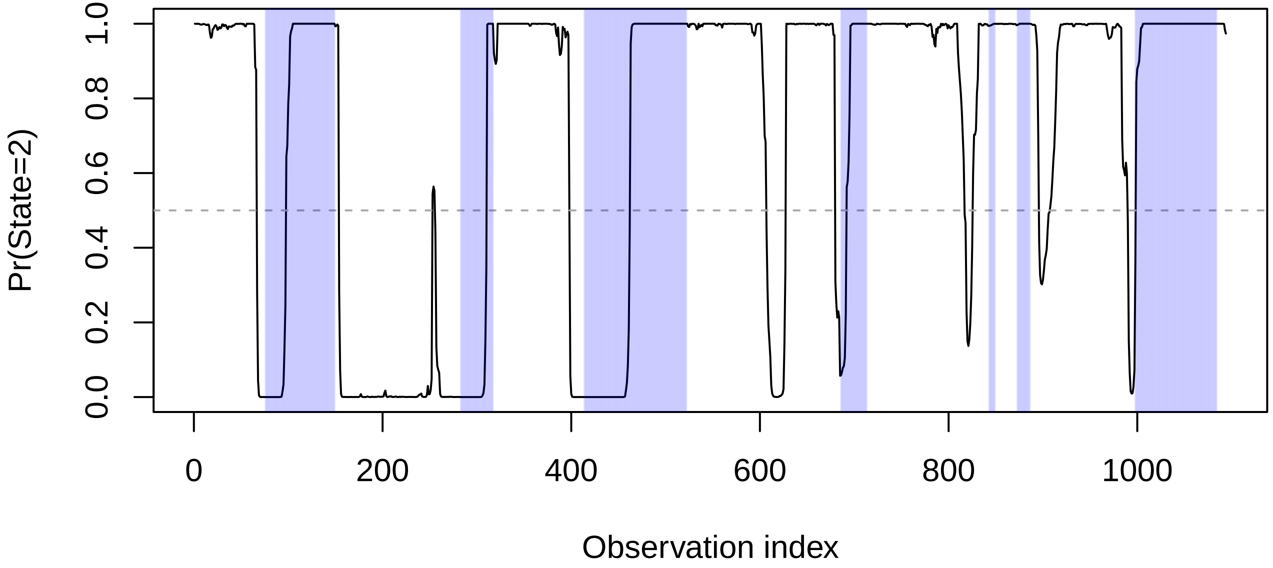

Pr(State=2)
0.0
0.2
0.4
0.6
0.8
1.0
0
200
400
600
800
1000
Observation index

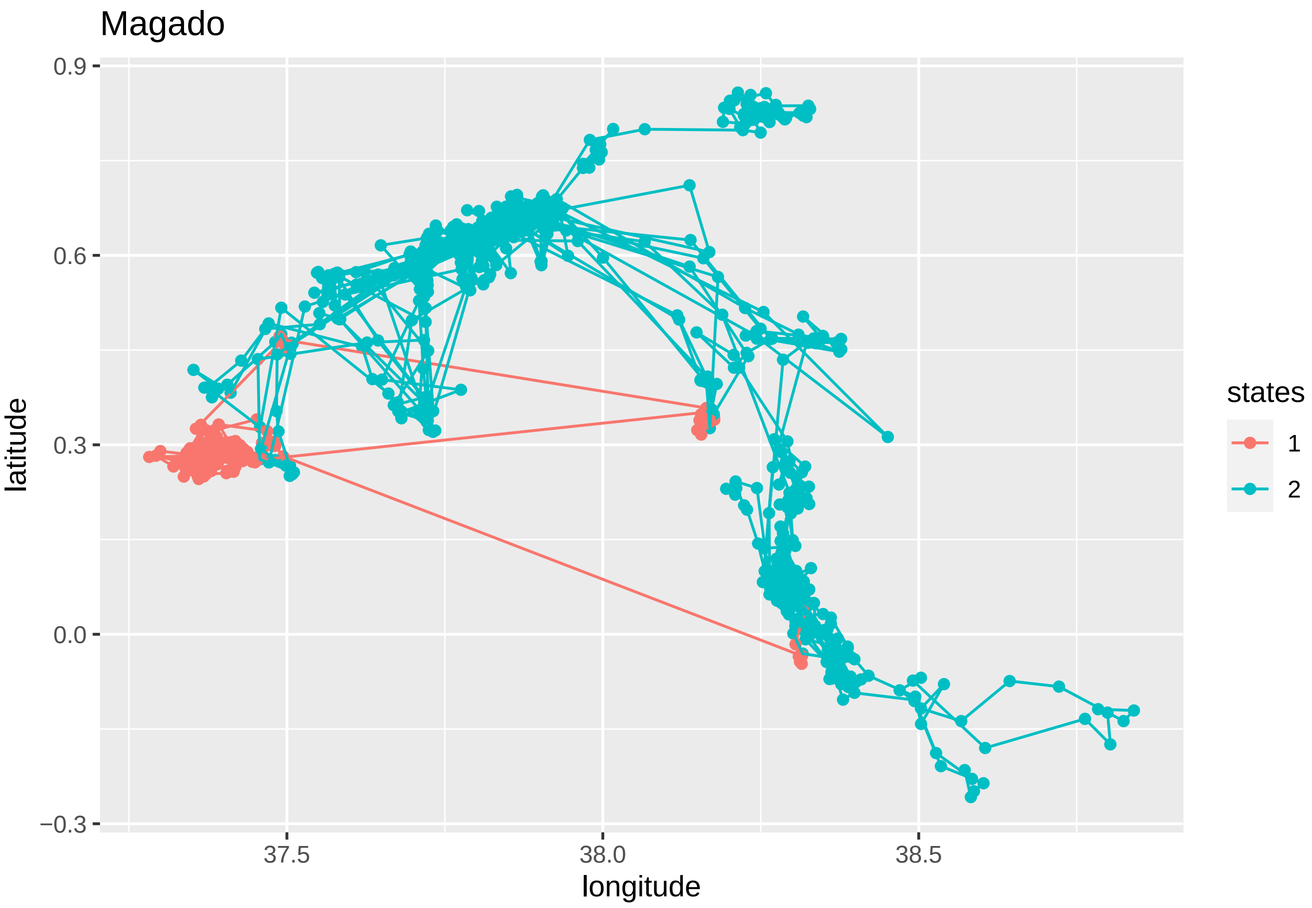
Magado
0.9
0.6
0.3
0.0
−0.3
latitude
37.5
38.0
38.5
longitude
states
1
2

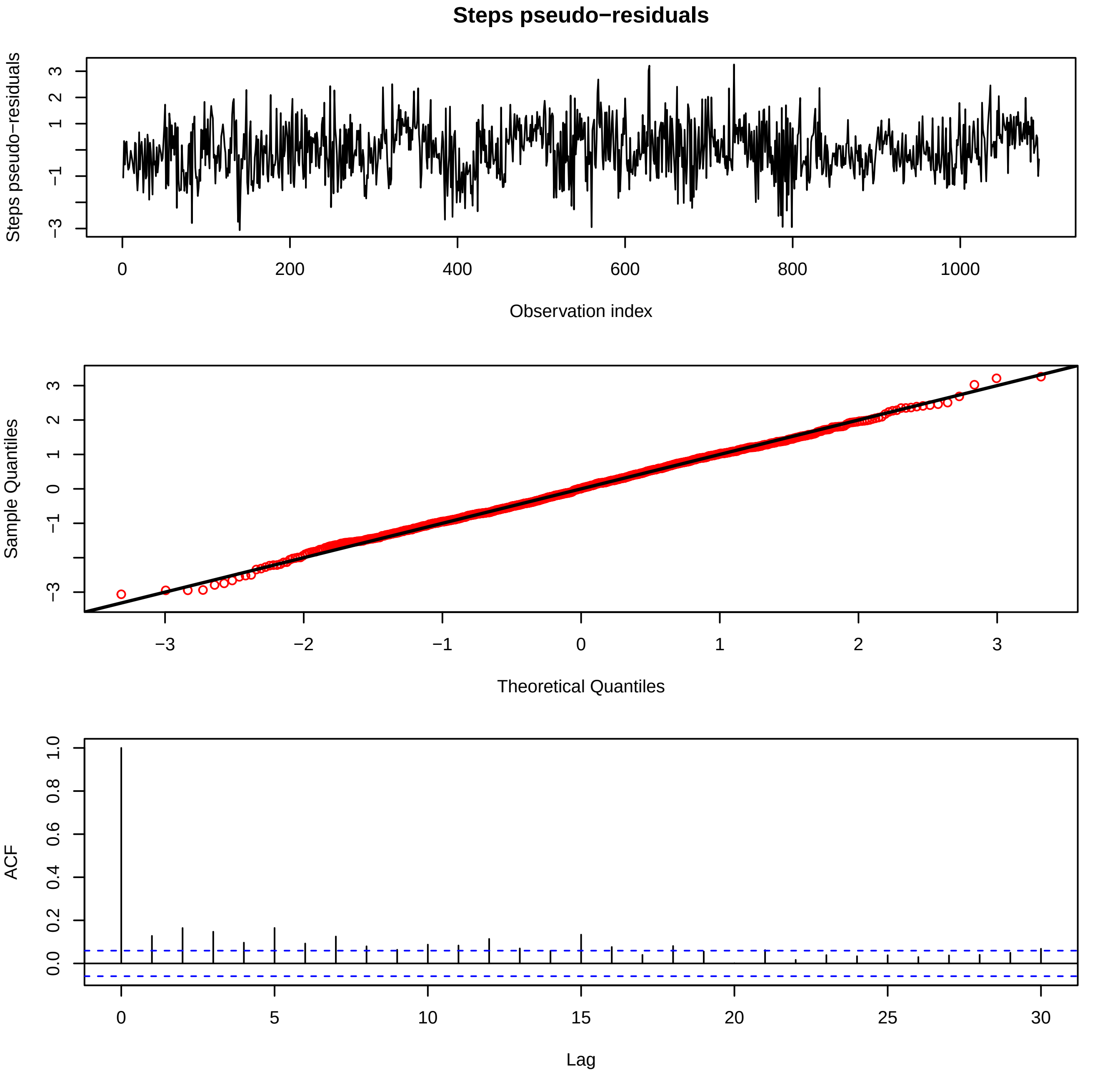

Steps pseudo−residuals
Steps pseudo−residuals
Observation index
Sample Quantiles
Theoretical Quantiles
ACF
Lag

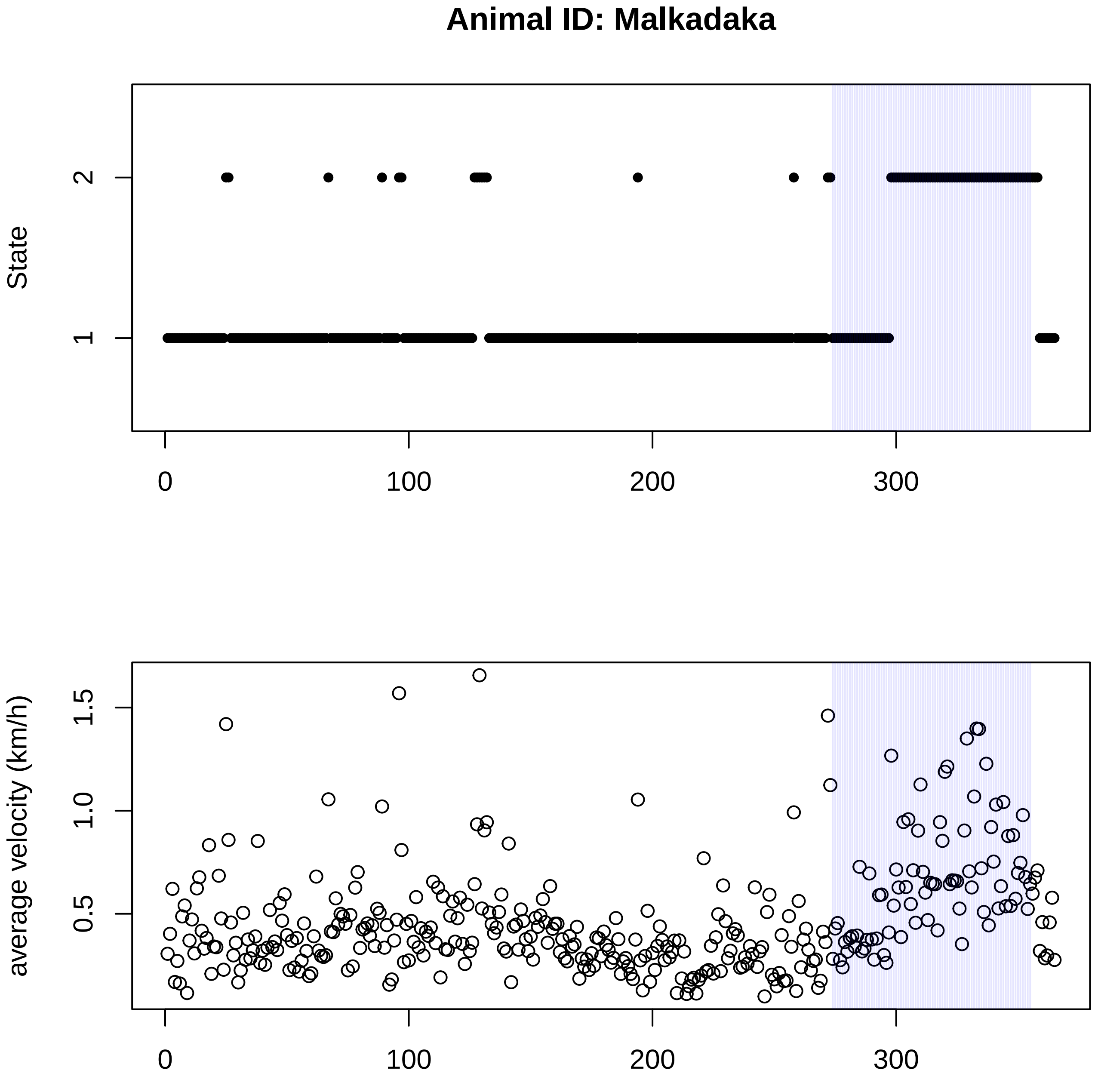

Animal ID: Malkadaka
State
1
2
0
100
200
300
average velocity (km/h)
0.5
1.0
1.5
0
100
200
300

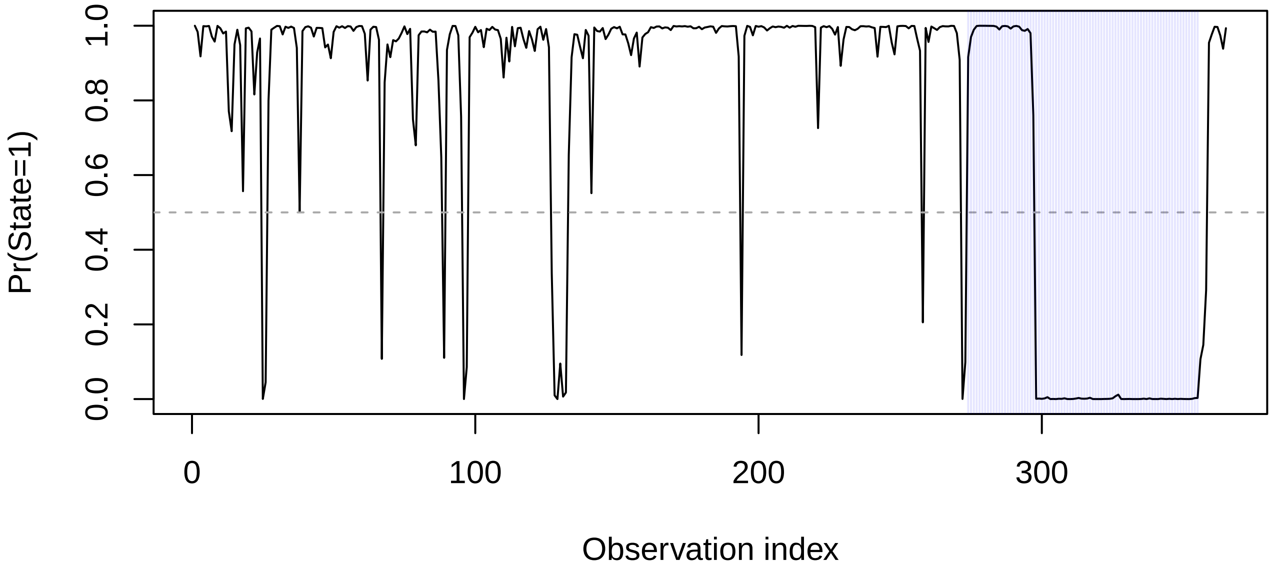

Pr(State=1)
0.0
0.2
0.4
0.6
0.8
1.0
0
100
200
300
Observation index

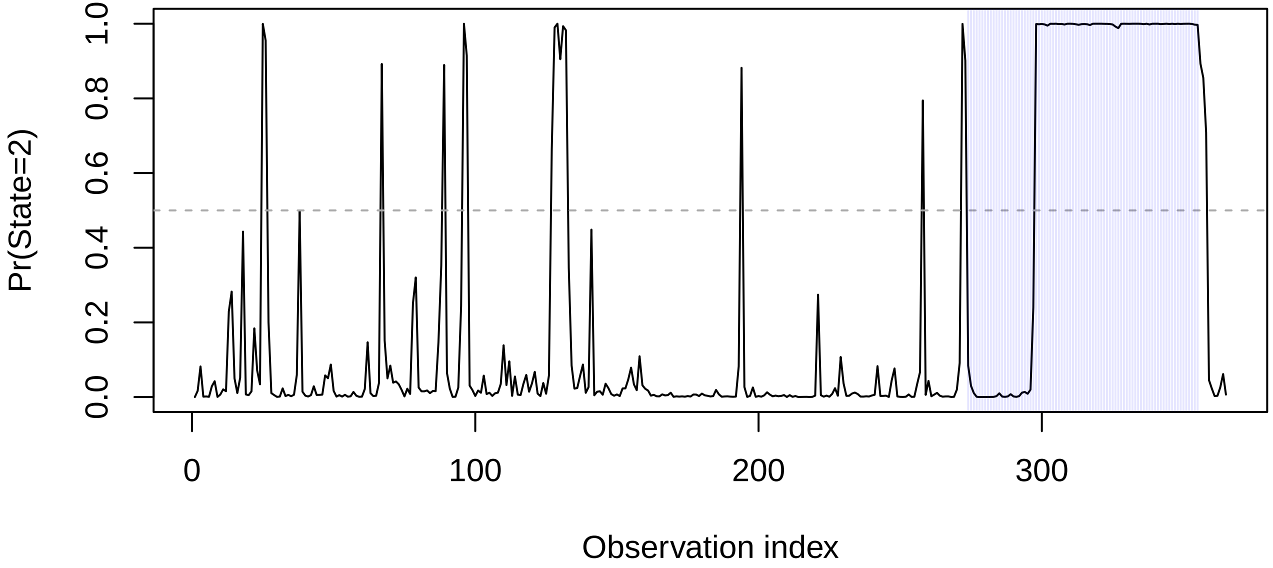

Pr(State=2)
0.0
0.2
0.4
0.6
0.8
1.0
0
100
200
300
Observation index

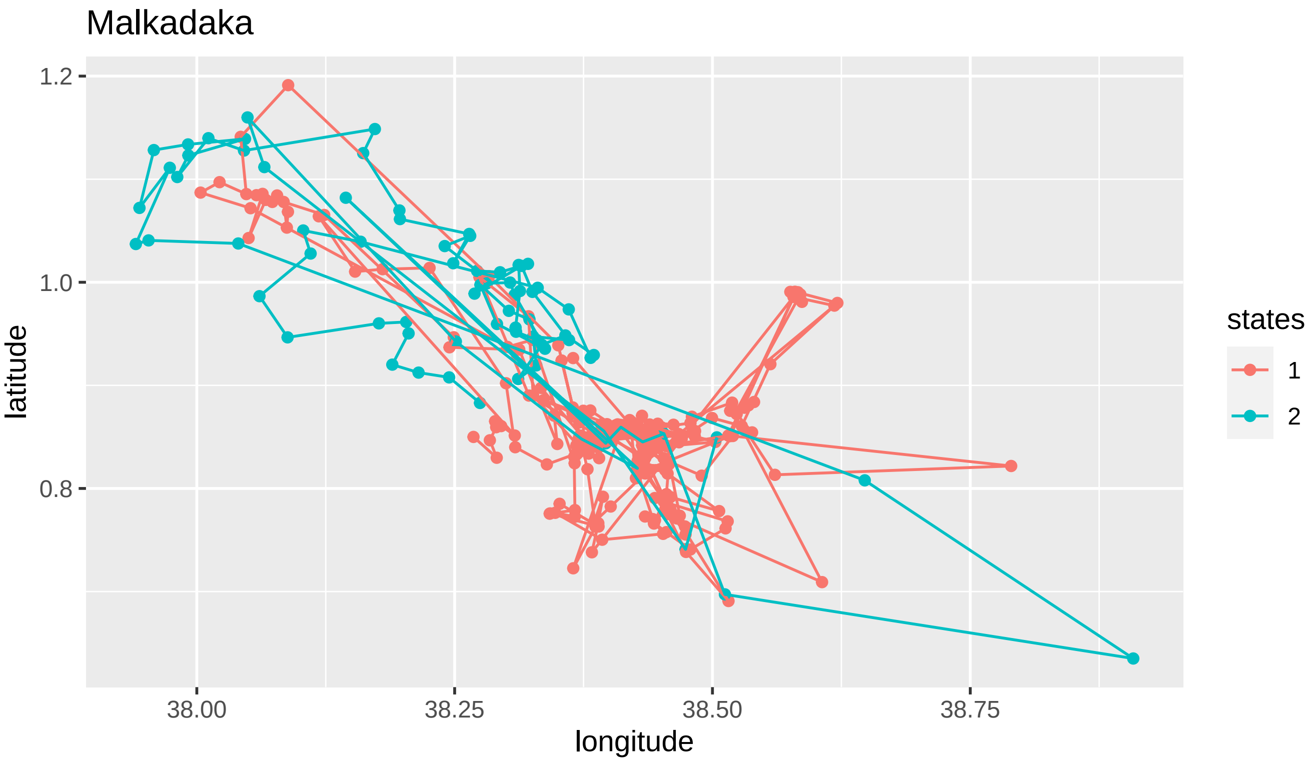
Malkadaka
latitude
longitude
1.2
1.0
0.8
38.00
38.25
38.50
38.75
states
1
2

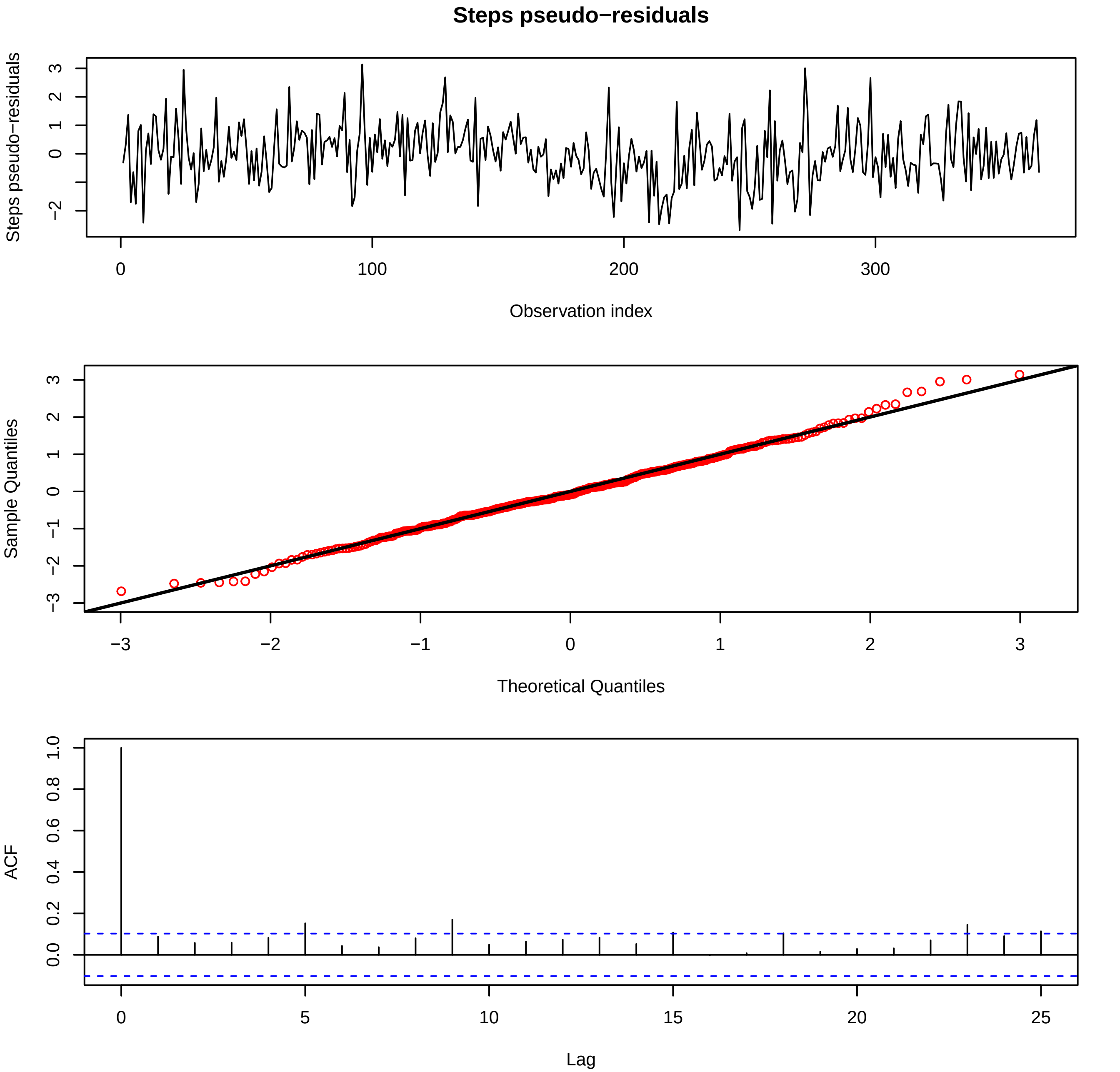

Steps pseudo−residuals
Steps pseudo−residuals
Observation index
0
100
200
300
−2
0
1
2
3
Sample Quantiles
Theoretical Quantiles
−3
−2
−1
0
1
2
3
ACF
Lag
0
5
10
15
20
25
0.0
0.2
0.4
0.6
0.8
1.0

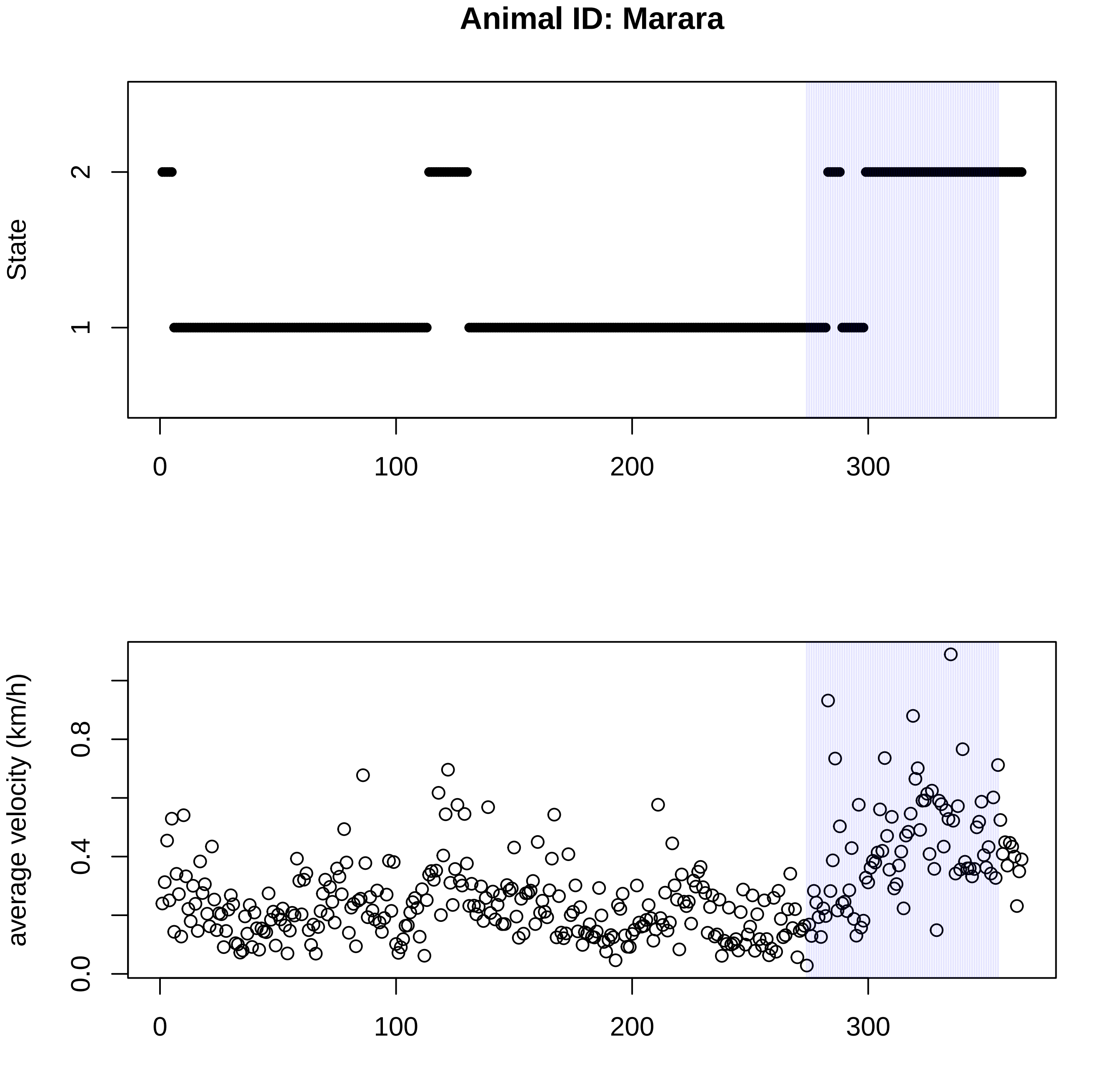

Animal ID: Marara
State
2
1
0
100
200
300
average velocity (km/h)
0.0
0.4
0.8

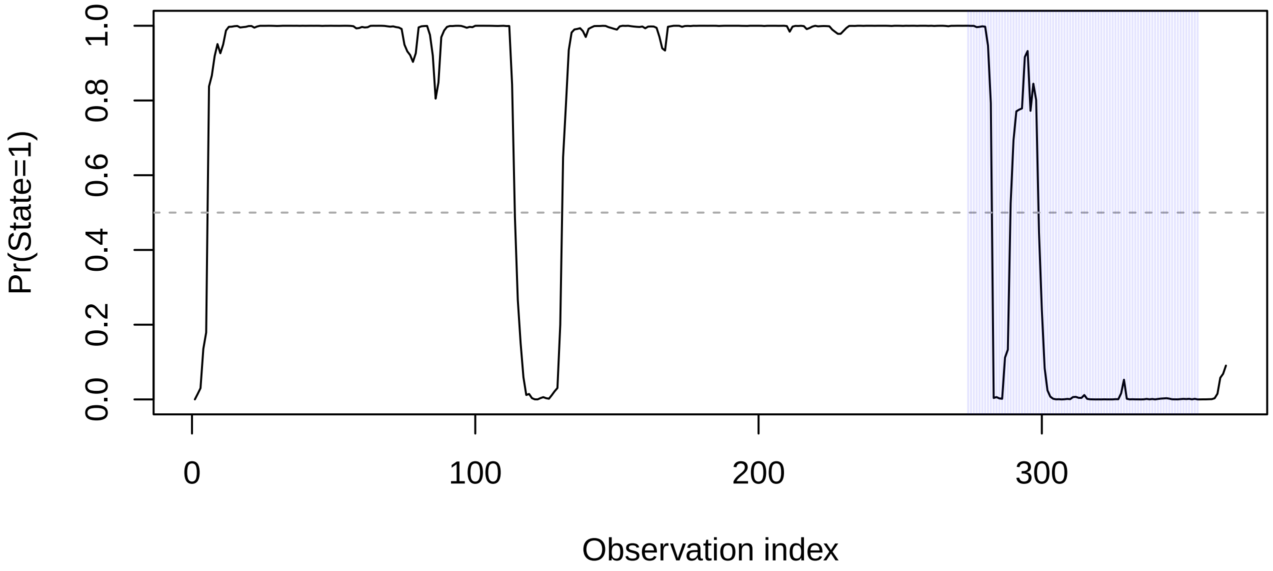

Pr(State=1)
1.0
0.8
0.6
0.4
0.2
0.0
0
100
200
300
Observation index

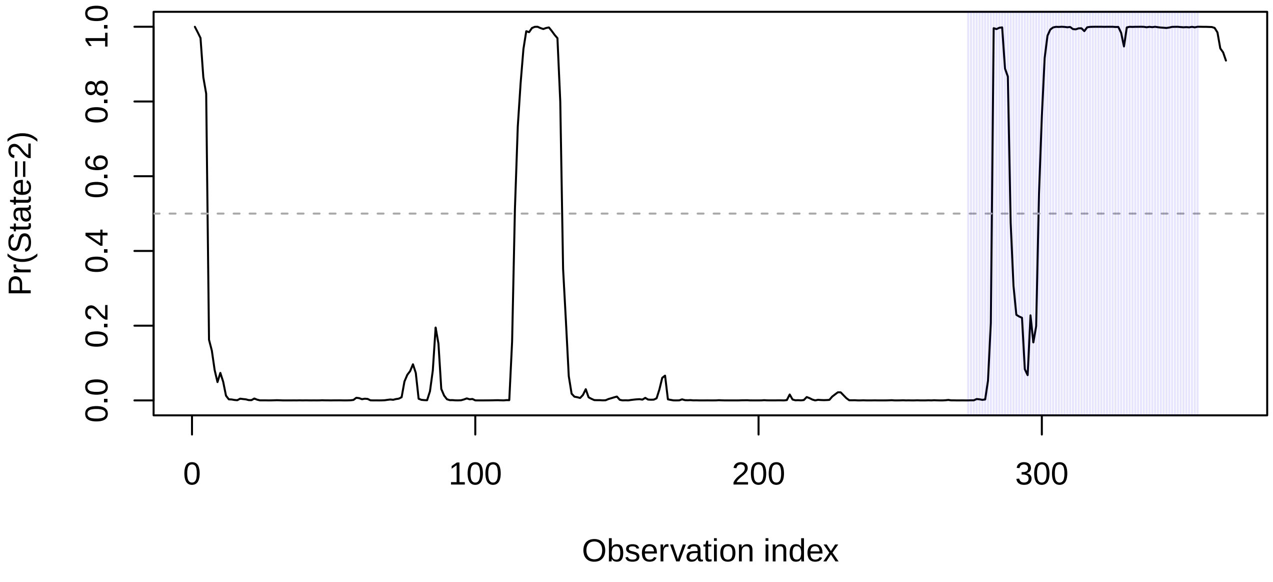

Pr(State=2)
1.0
0.8
0.6
0.4
0.2
0.0
0
100
200
300
Observation index

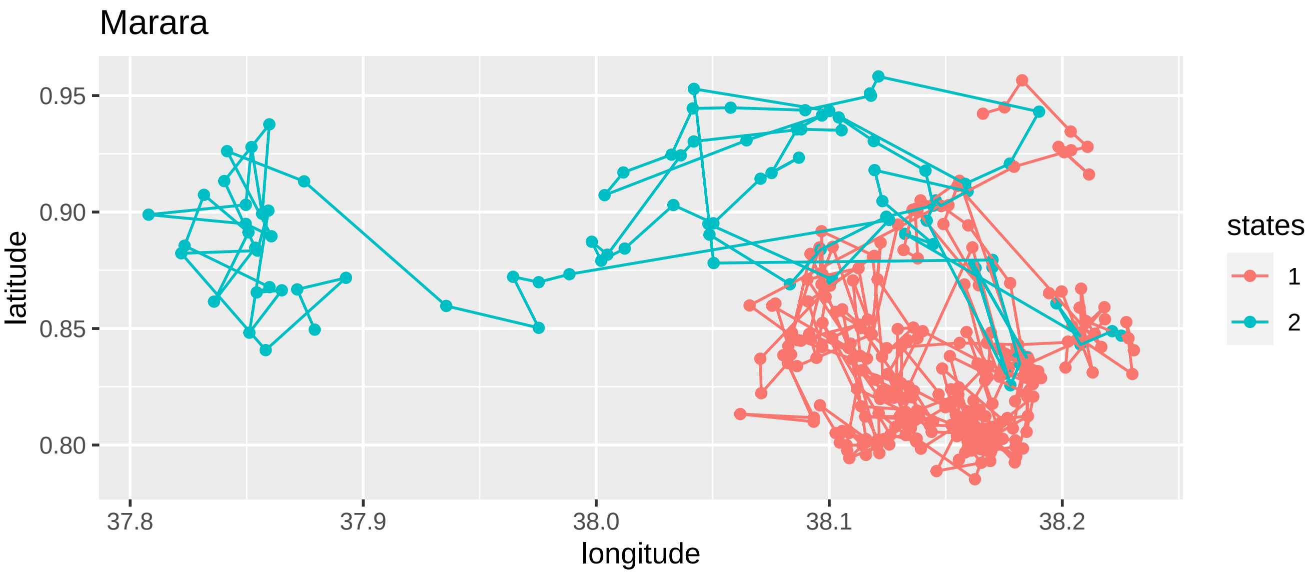

Marara
0.95
0.90
0.85
0.80
latitude
37.8
37.9
38.0
38.1
38.2
longitude
states
1
2

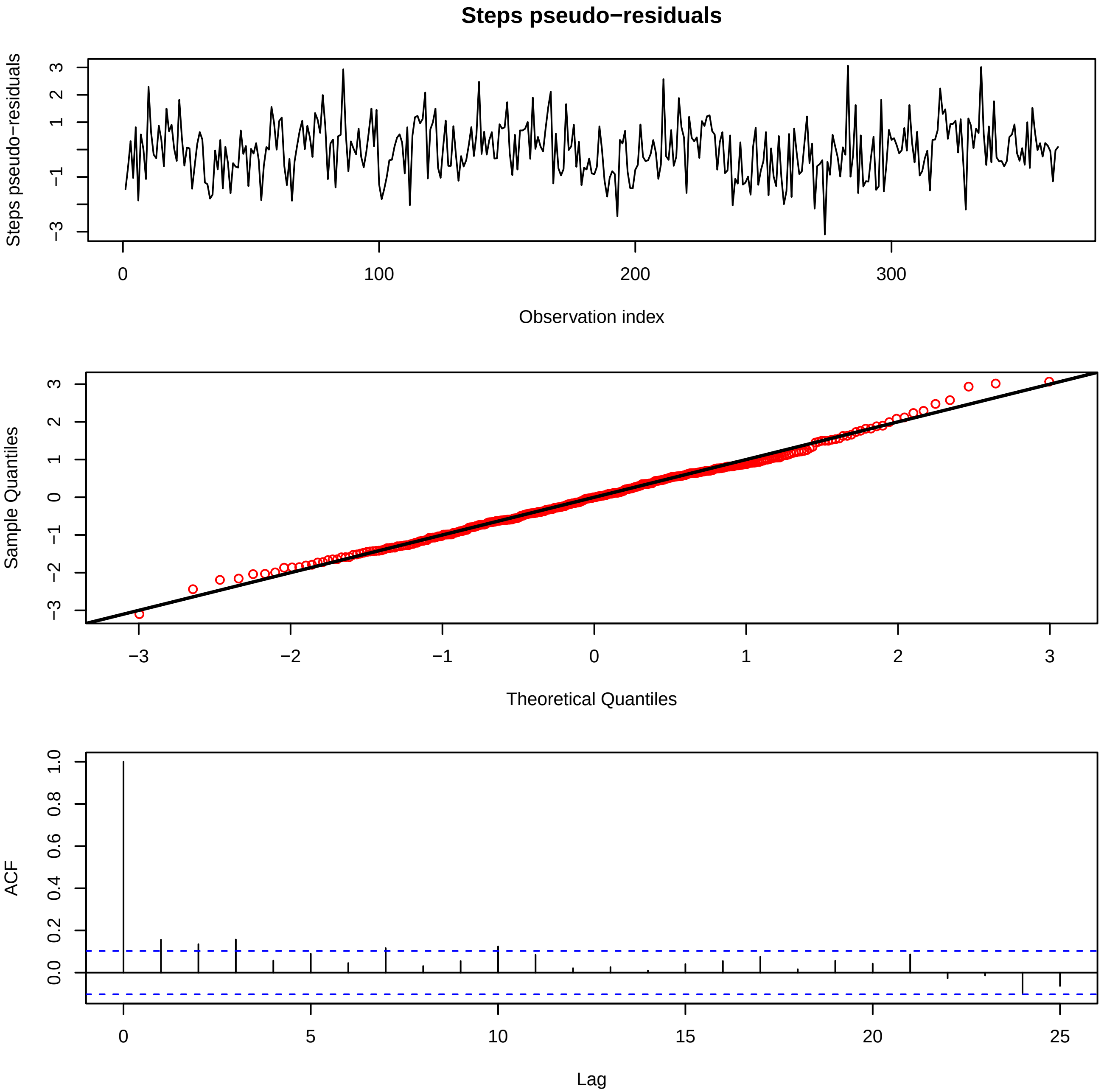

Steps pseudo−residuals
Steps pseudo−residuals
Observation index
Sample Quantiles
Theoretical Quantiles
ACF
Lag

Animal ID: Naisula

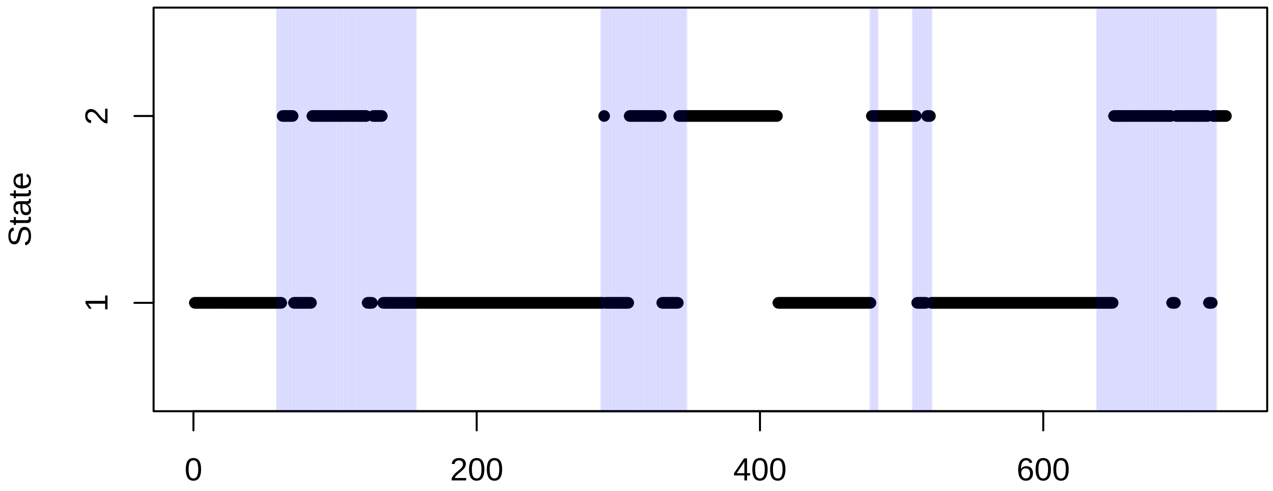

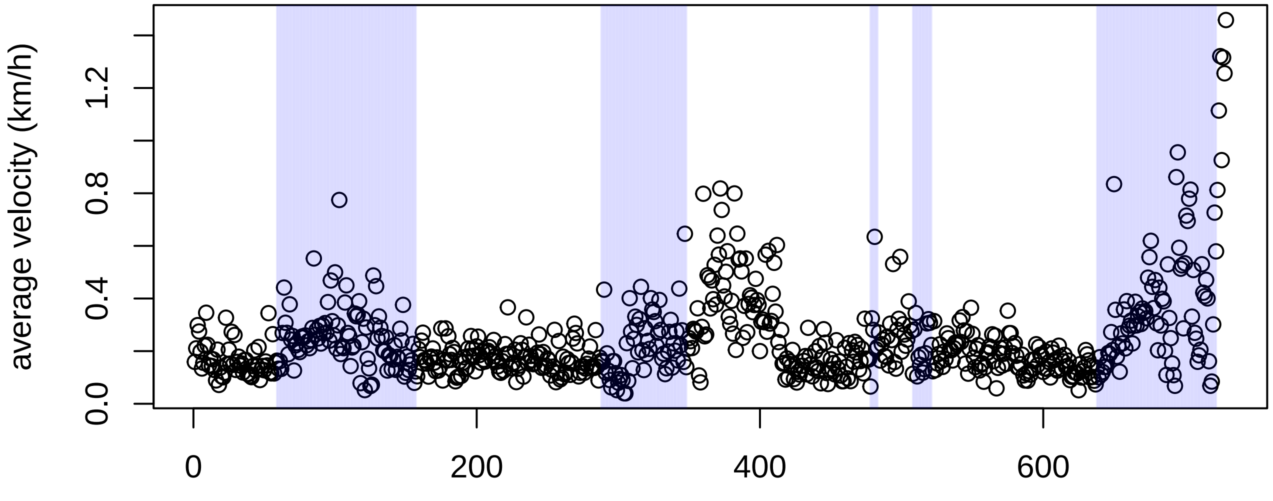

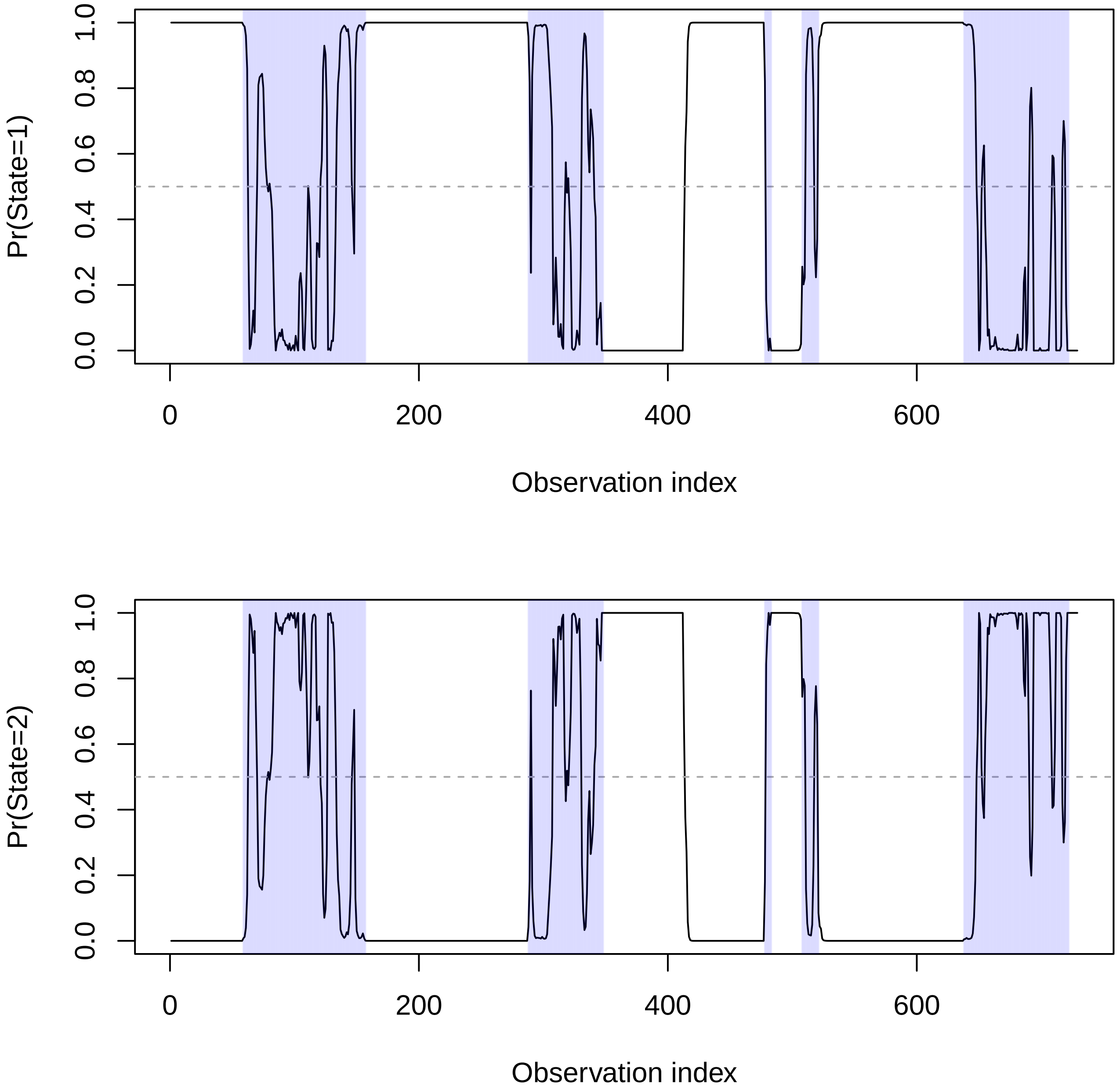

Pr(State=1)
0.0
0.2
0.4
0.6
0.8
1.0
0
200
400
600
Observation index
Pr(State=2)
0.0
0.2
0.4
0.6
0.8
1.0
0
200
400
600
Observation index

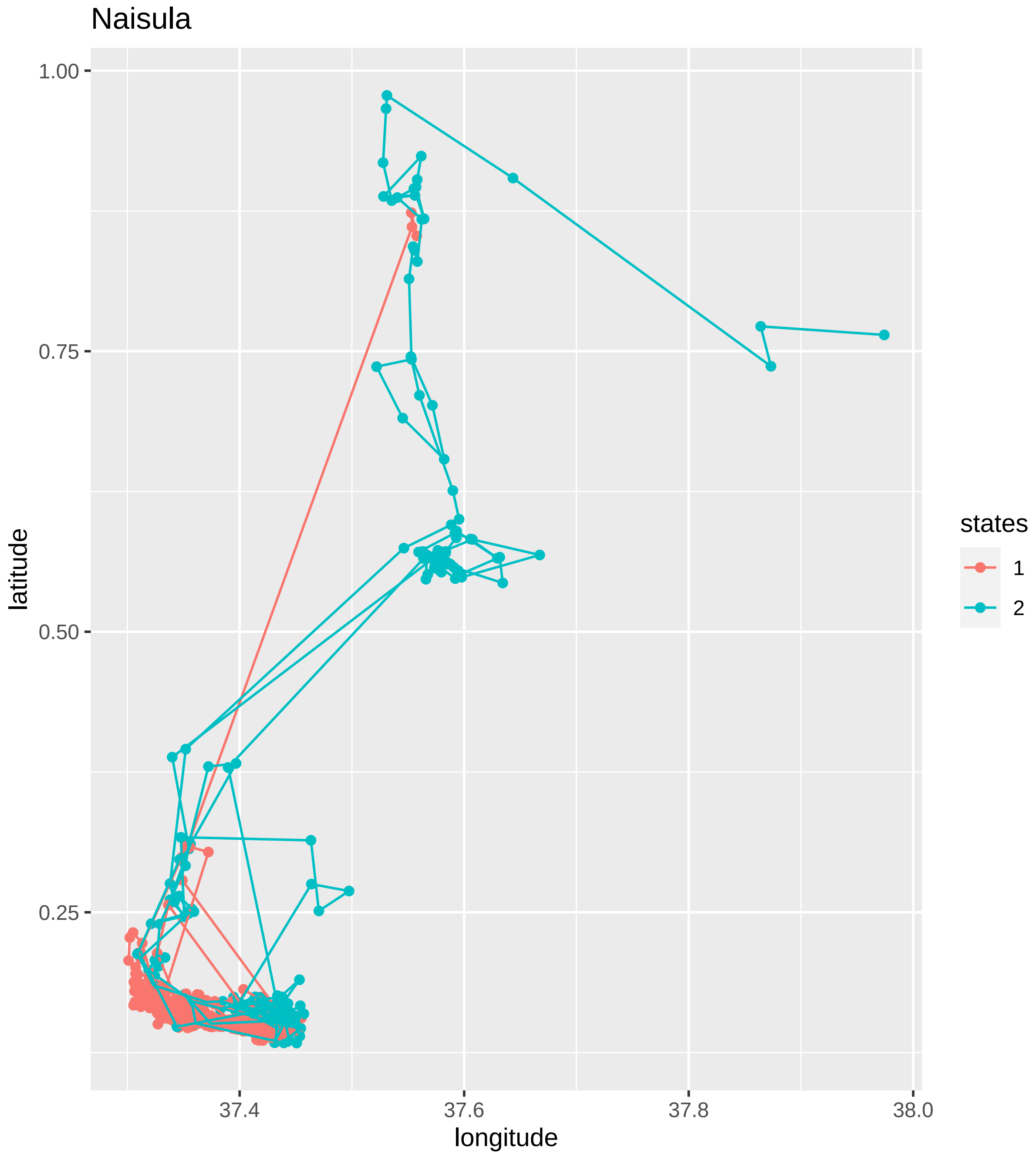

Naisula
latitude
1.00
0.75
0.50
0.25
longitude
37.4
37.6
37.8
38.0
states
1
2

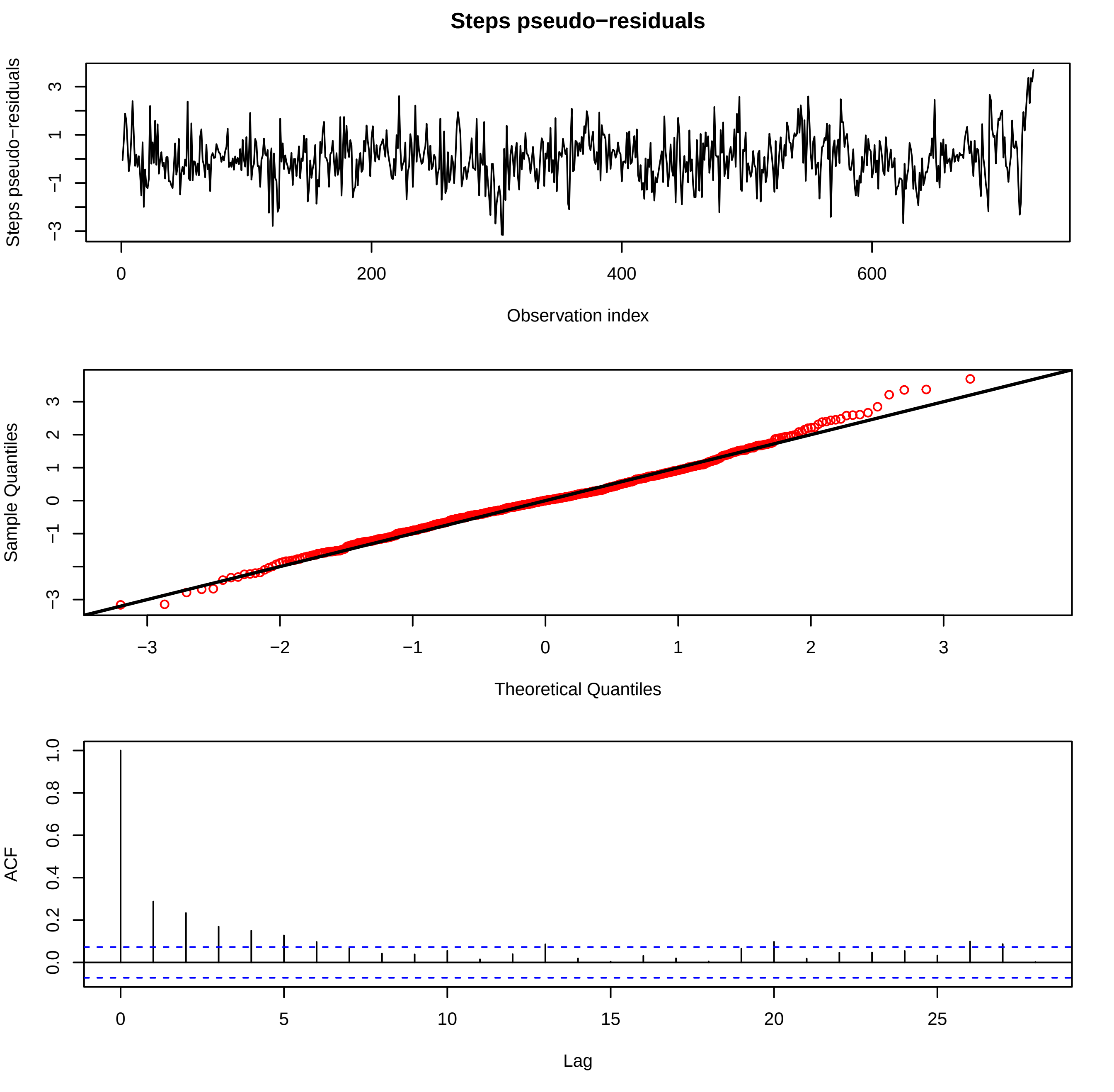
Steps pseudo−residuals
Steps pseudo−residuals
−3
−1
1
3
0
200
400
600
Observation index
Sample Quantiles
−3
−1
0
1
2
3
−3
−2
−1
0
1
2
3
Theoretical Quantiles
ACF
0.0
0.2
0.4
0.6
0.8
1.0
0
5
10
15
20
25
Lag

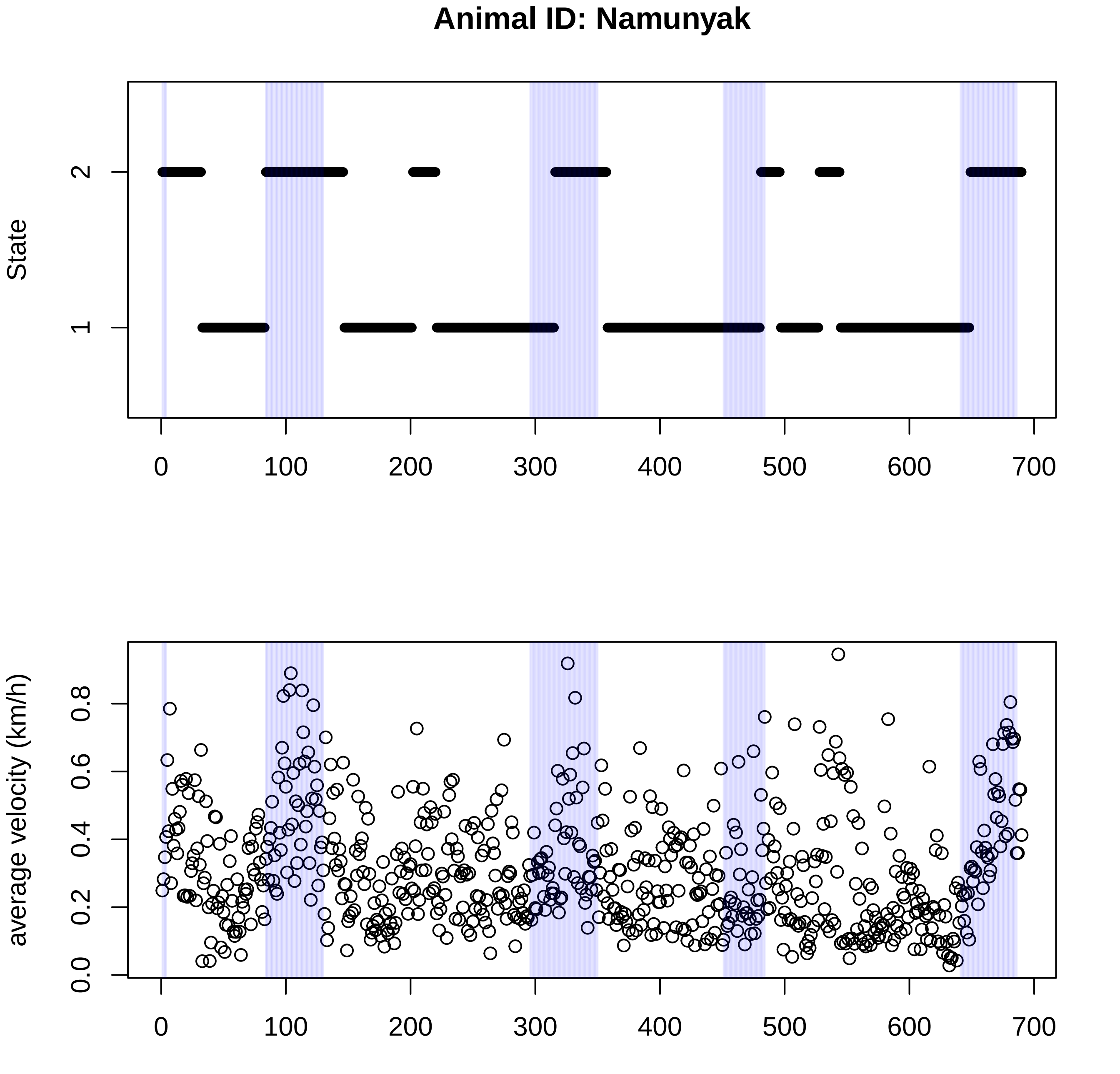

Animal ID: Namunyak
State
2
1
0
100
200
300
400
500
600
700
average velocity (km/h)
0.0
0.2
0.4
0.6
0.8

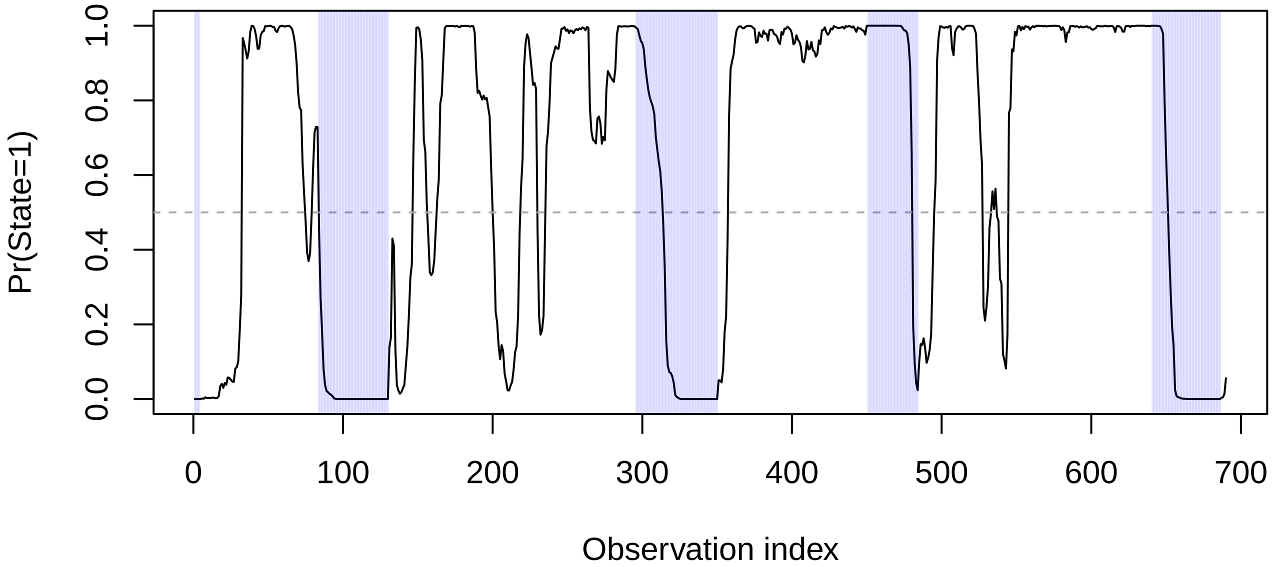

Pr(State=1)
0.0
0.2
0.4
0.6
0.8
1.0
0
100
200
300
400
500
600
700
Observation index

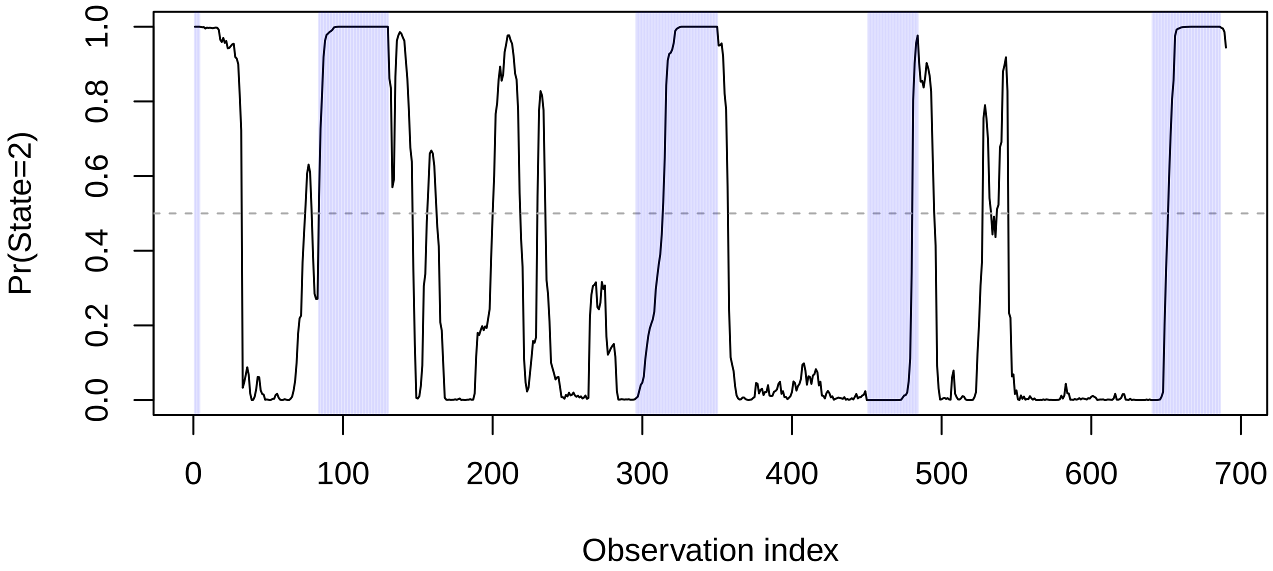

Pr(State=2)
0.0
0.2
0.4
0.6
0.8
1.0
0
100
200
300
400
500
600
700
Observation index

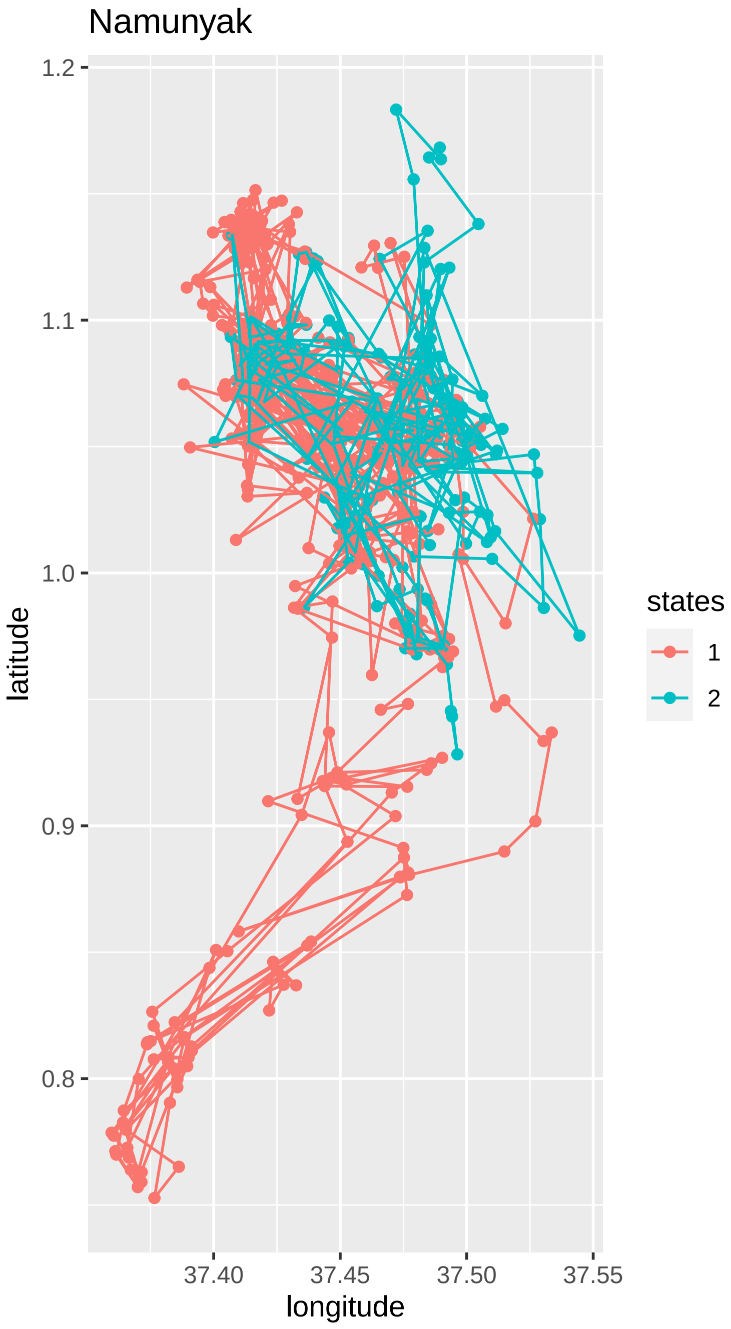

Namunyak
latitude
longitude
1.2
1.1
1.0
0.9
0.8
37.40
37.45
37.50
37.55
states
1
2

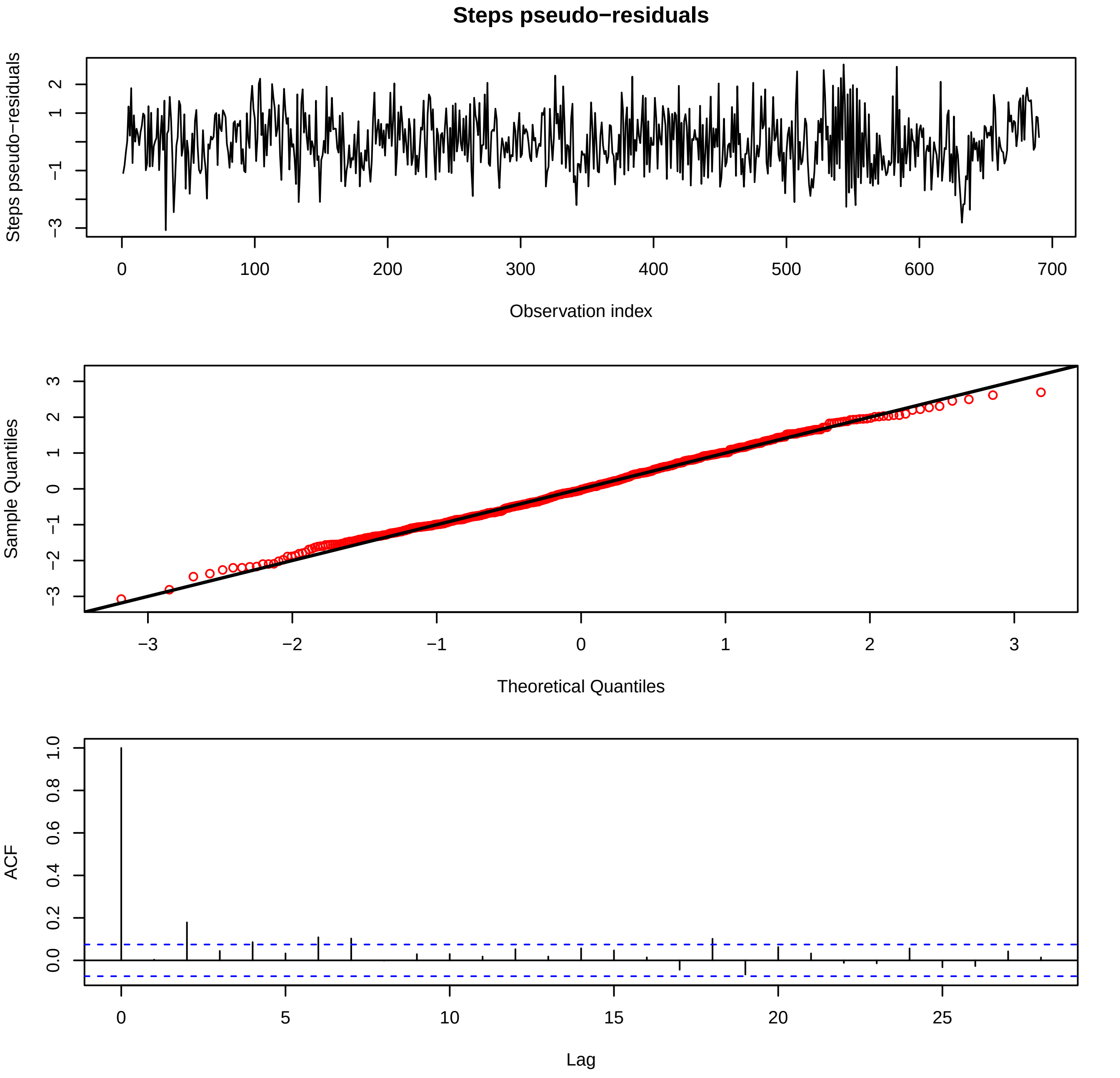

Steps pseudo−residuals
Steps pseudo−residuals
Observation index
Sample Quantiles
Theoretical Quantiles
ACF
Lag

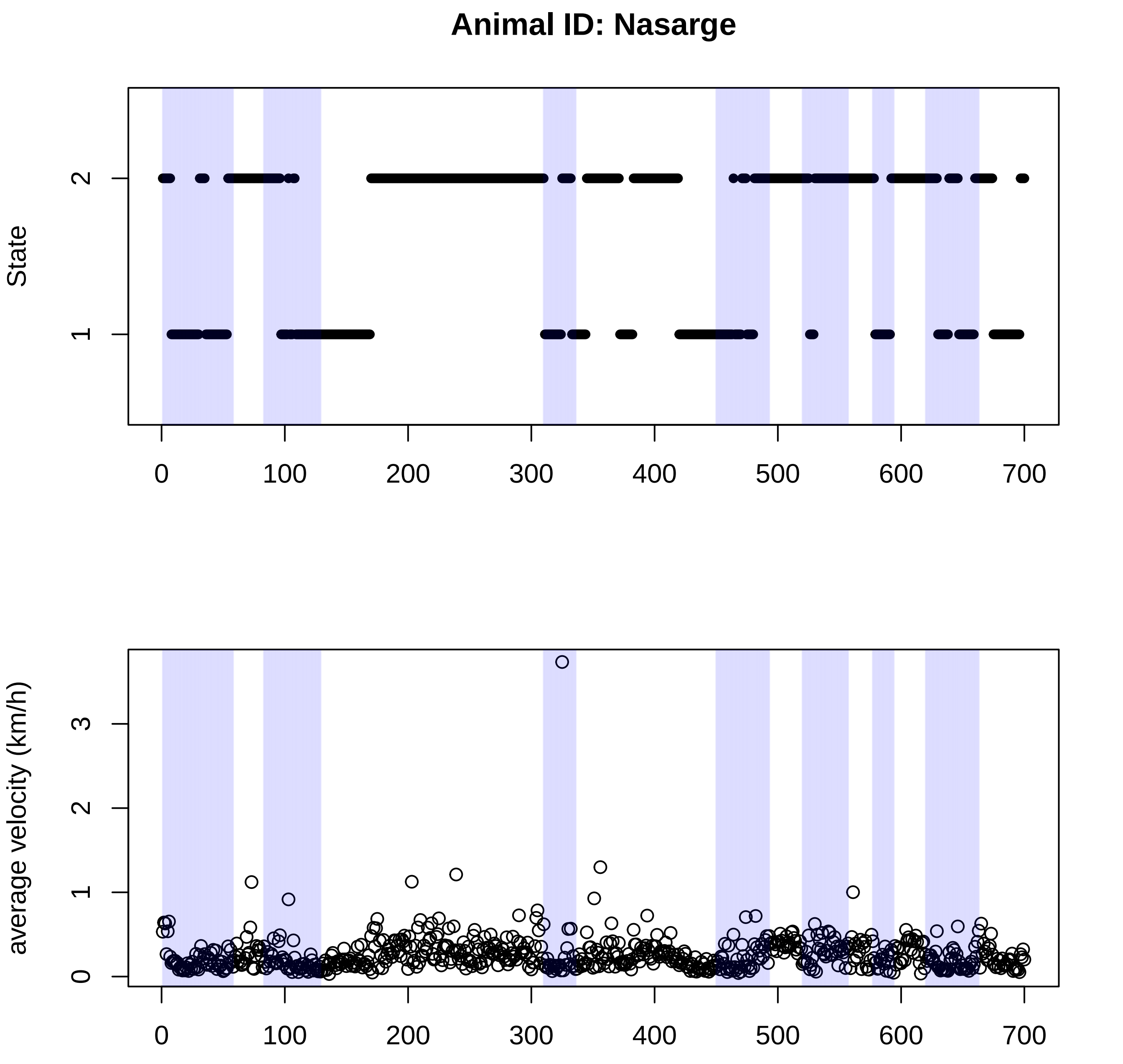

Animal ID: Nasarge
State
2
1
0
100
200
300
400
500
600
700
average velocity (km/h)
3
2
1
0
0
100
200
300
400
500
600
700

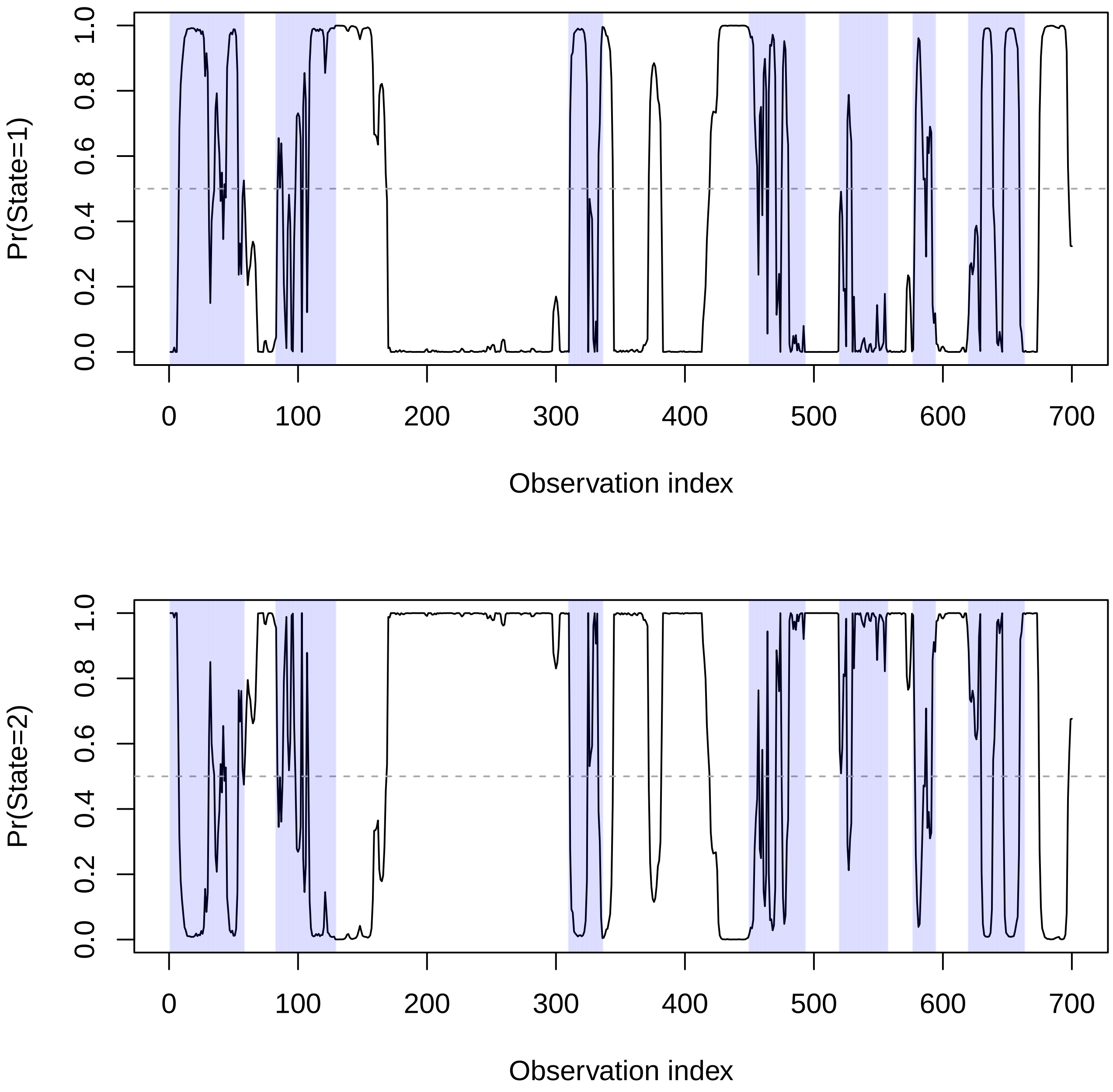
Pr(State=1)
1.0
0.8
0.6
0.4
0.2
0.0
0
100
200
300
400
500
600
700
Observation index
Pr(State=2)
1.0
0.8
0.6
0.4
0.2
0.0
0
100
200
300
400
500
600
700
Observation index

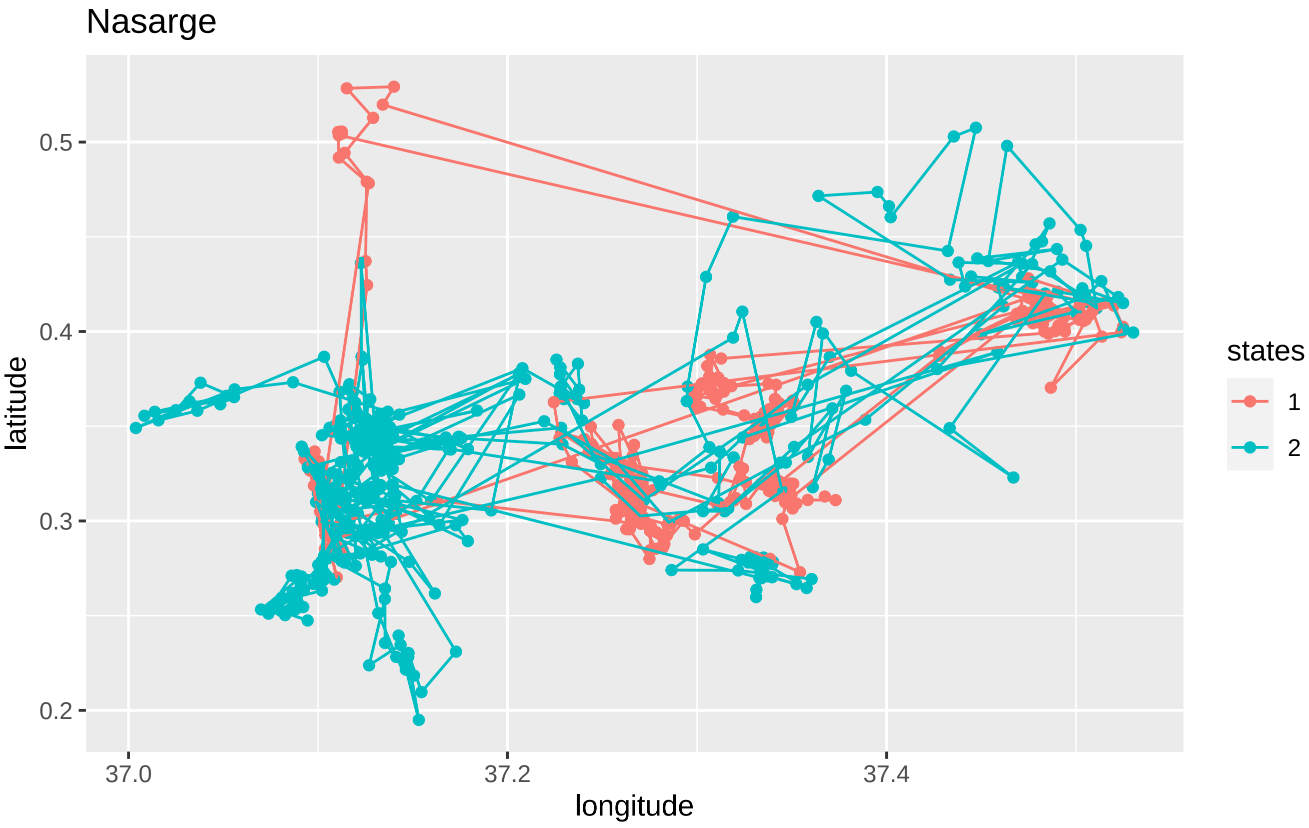
Nasarge
latitude
longitude
0.5
0.4
0.3
0.2
37.0
37.2
37.4
states
1
2

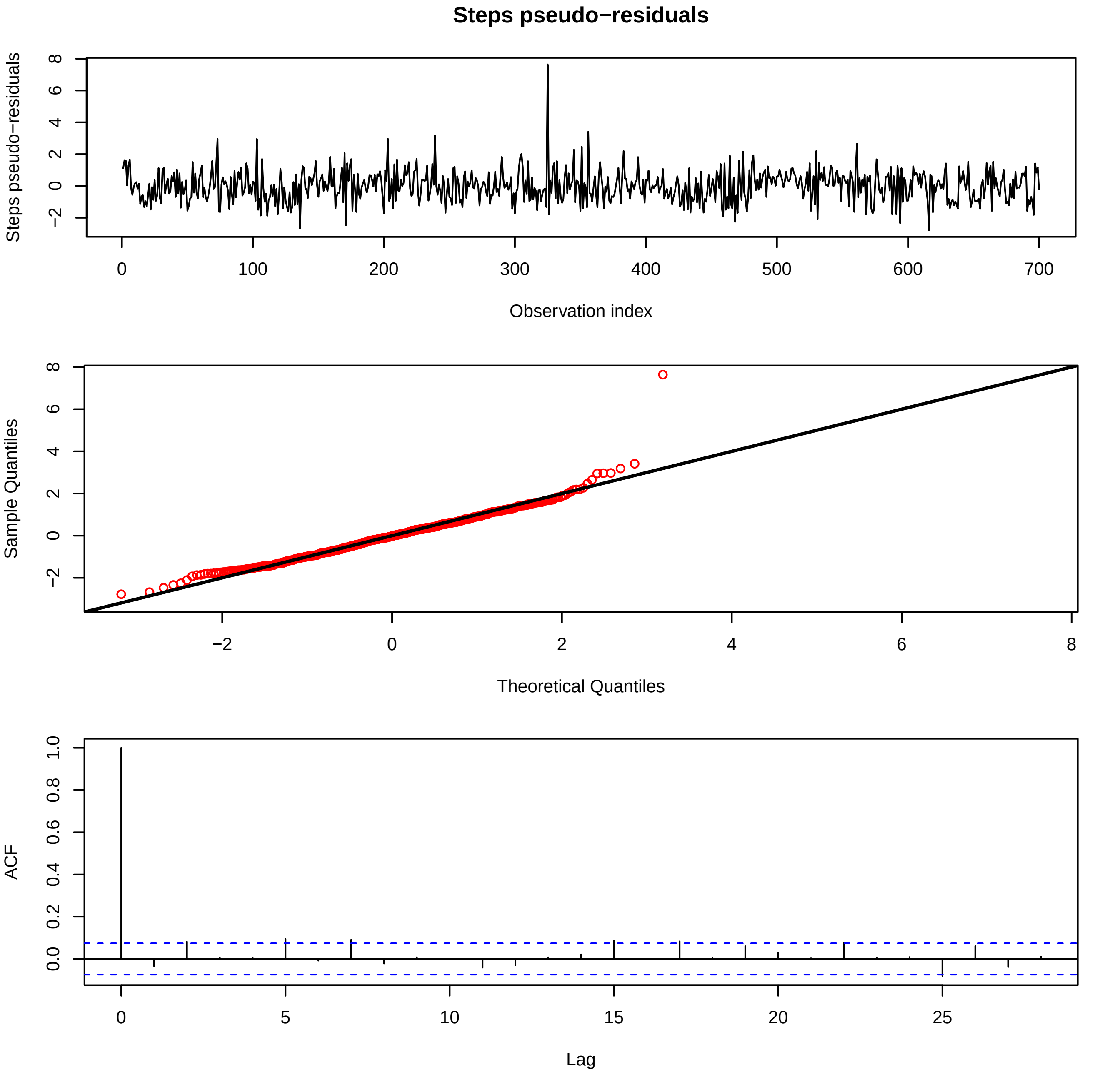

Steps pseudo−residuals
Steps pseudo−residuals
Observation index
Sample Quantiles
Theoretical Quantiles
ACF
Lag

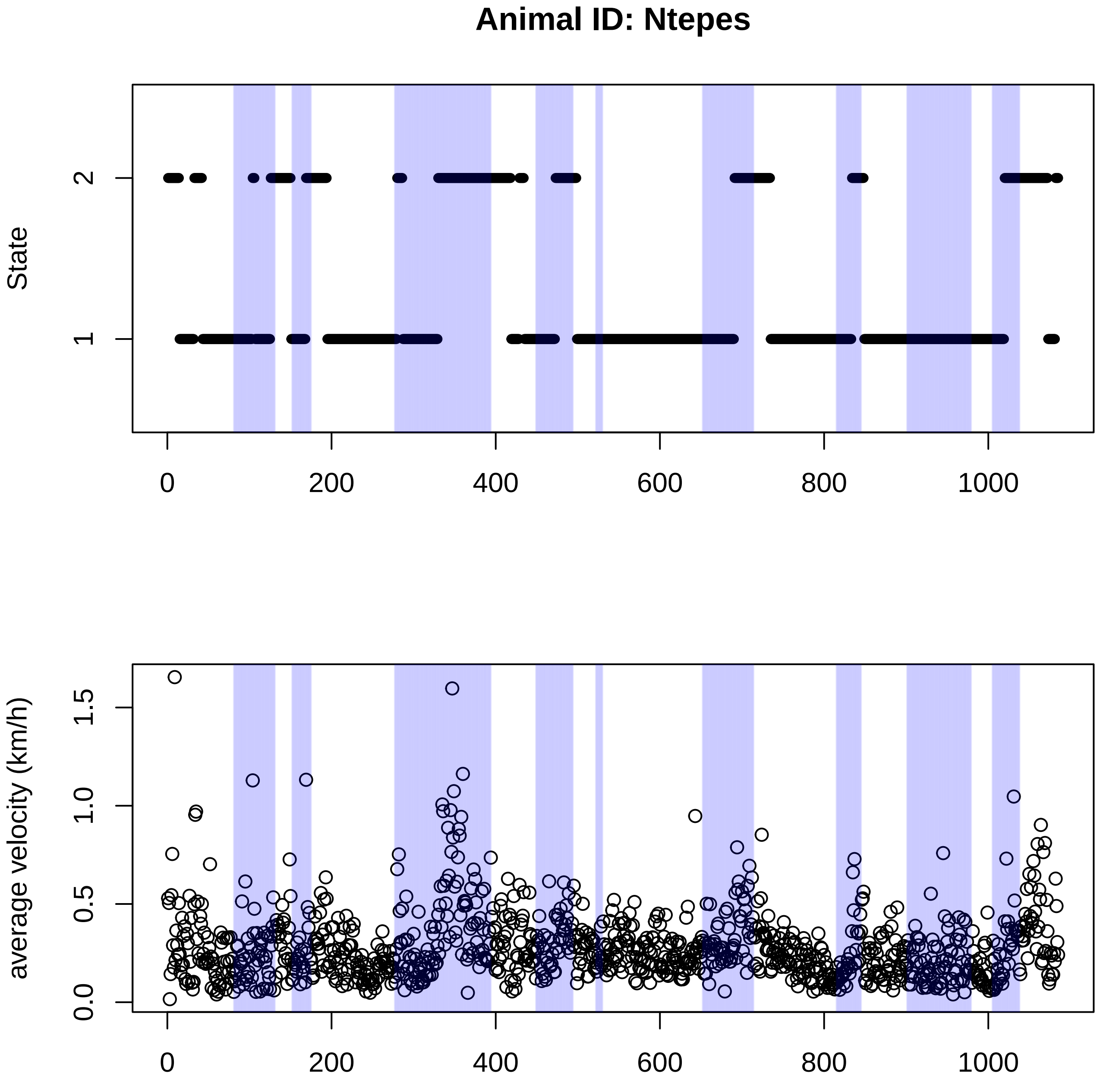
Animal ID: Ntepes
State
2
1
0
200
400
600
800
1000
average velocity (km/h)
1.5
1.0
0.5
0.0
0
200
400
600
800
1000

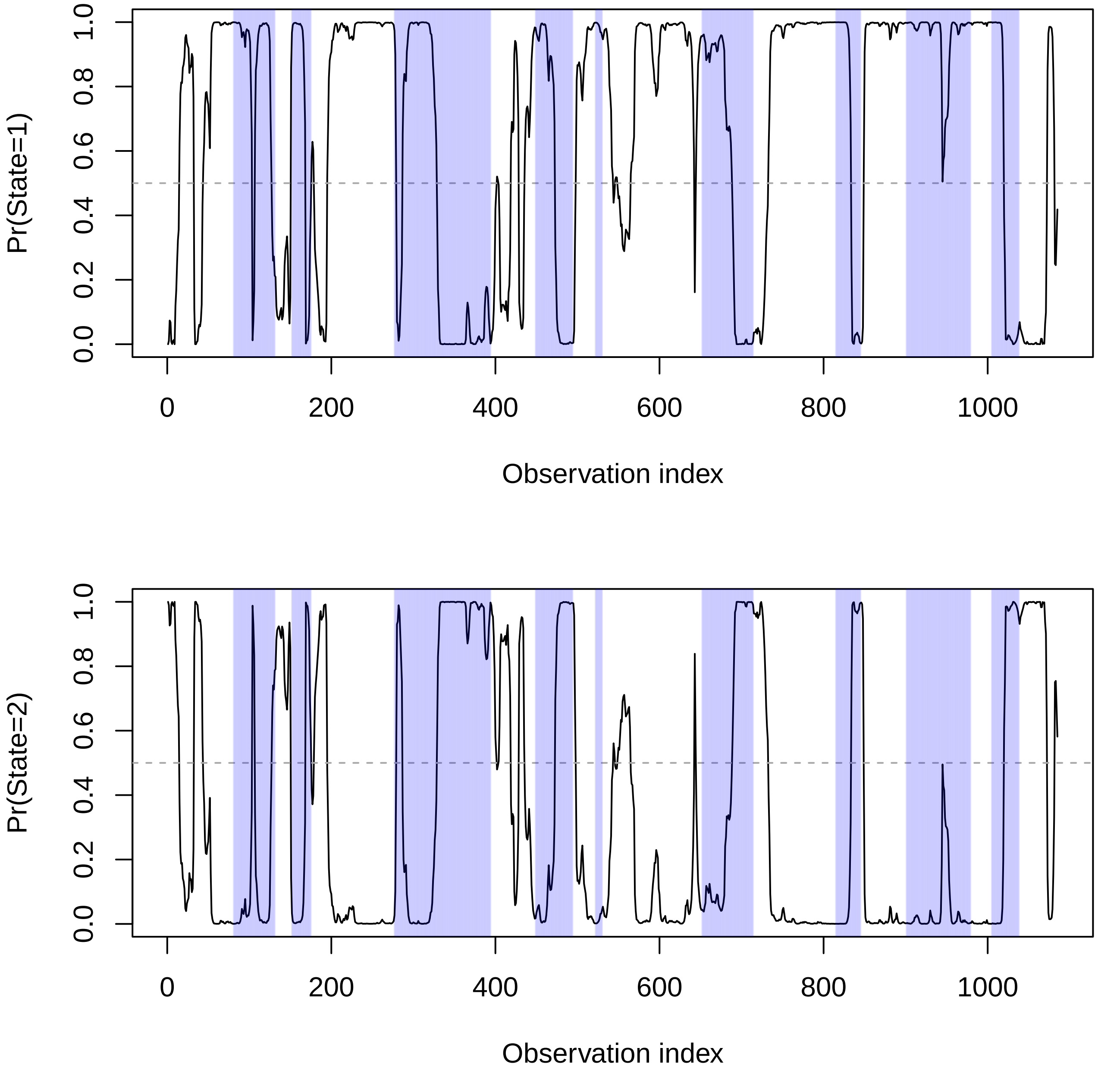

Pr(State=1)
0.0
0.2
0.4
0.6
0.8
1.0
0
200
400
600
800
1000
Observation index
Pr(State=2)
0.0
0.2
0.4
0.6
0.8
1.0
0
200
400
600
800
1000
Observation index

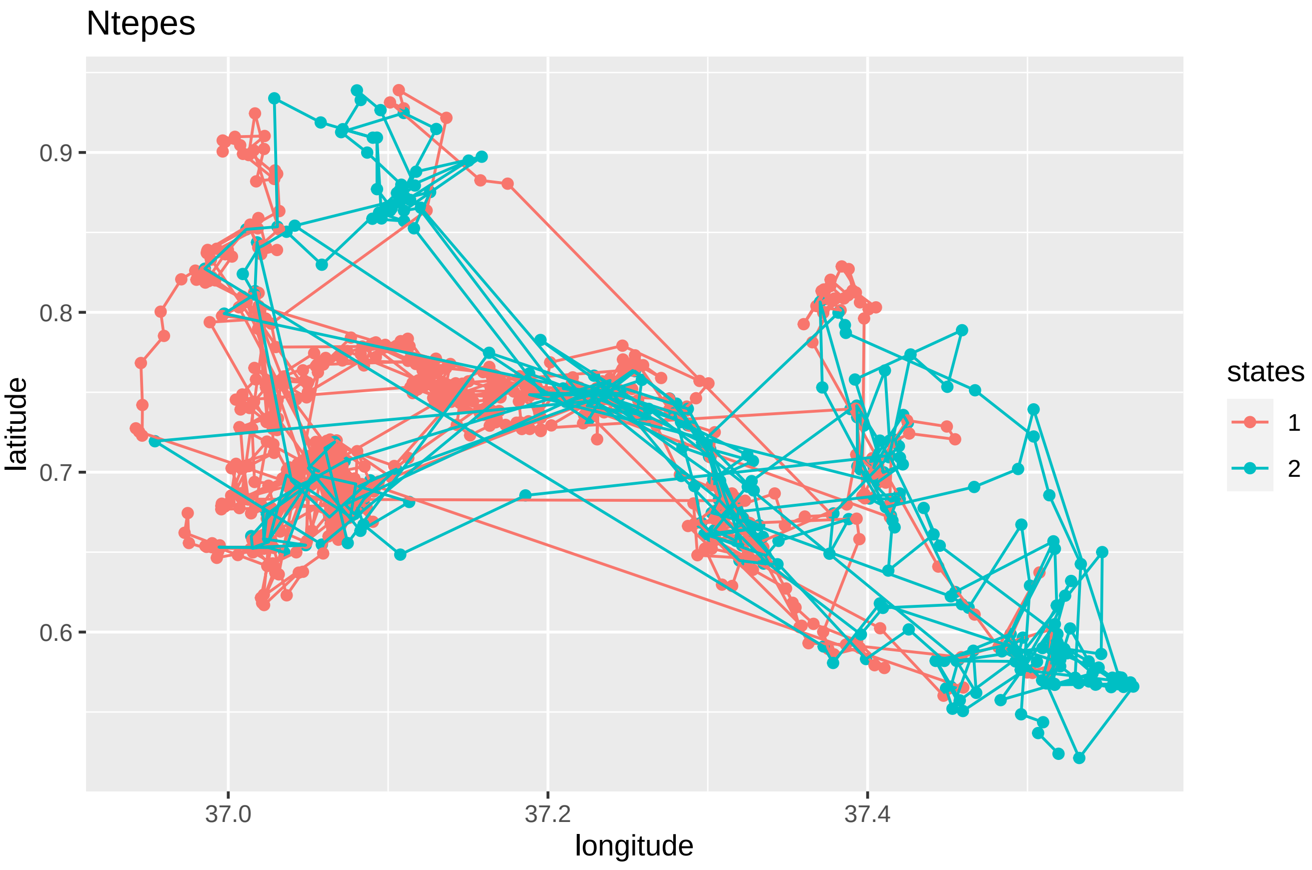

Ntepes
0.9
0.8
0.7
0.6
latitude
37.0
37.2
37.4
longitude
states
1
2

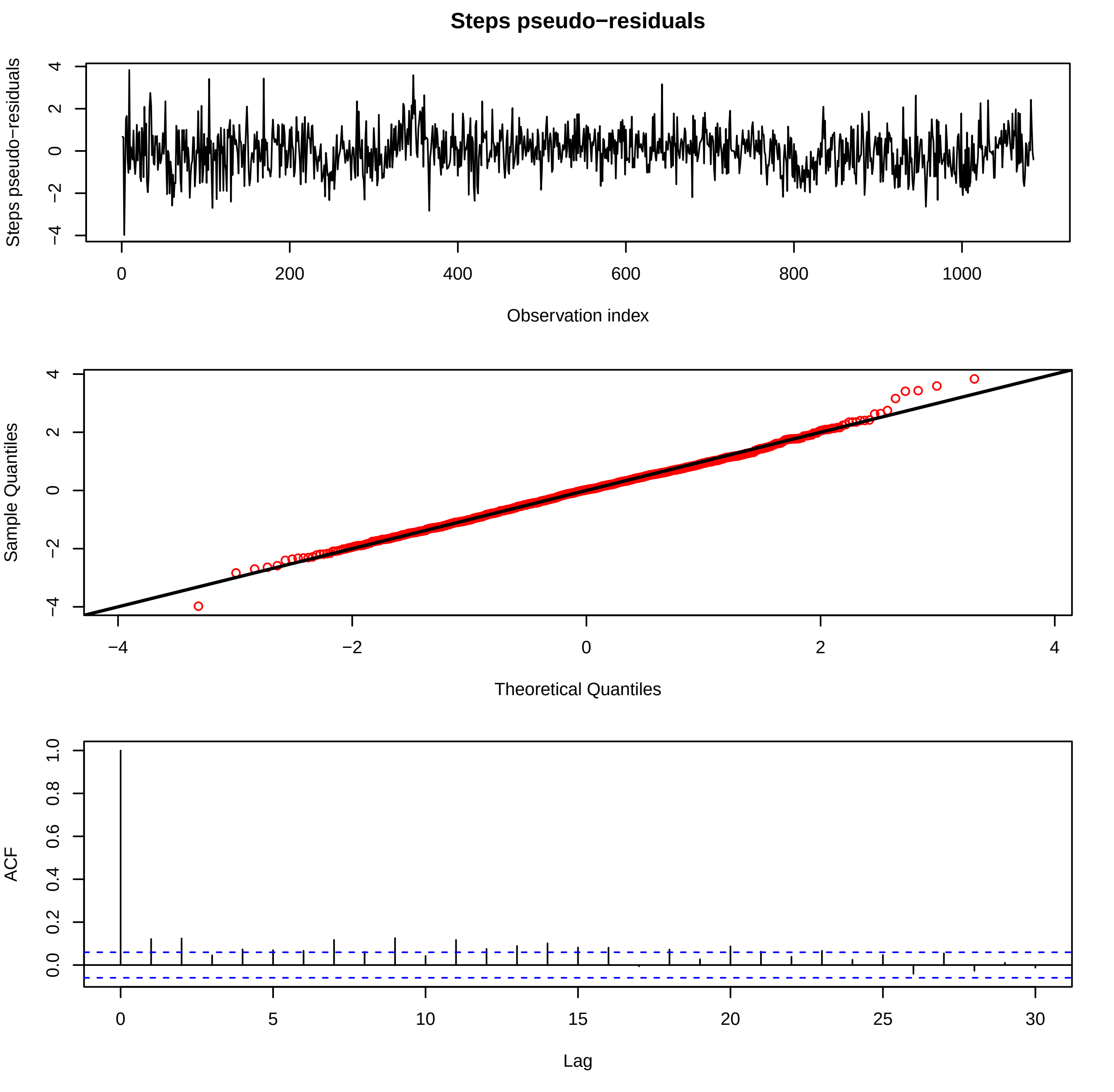

Steps pseudo−residuals
Steps pseudo−residuals
Observation index
Sample Quantiles
Theoretical Quantiles
ACF
Lag

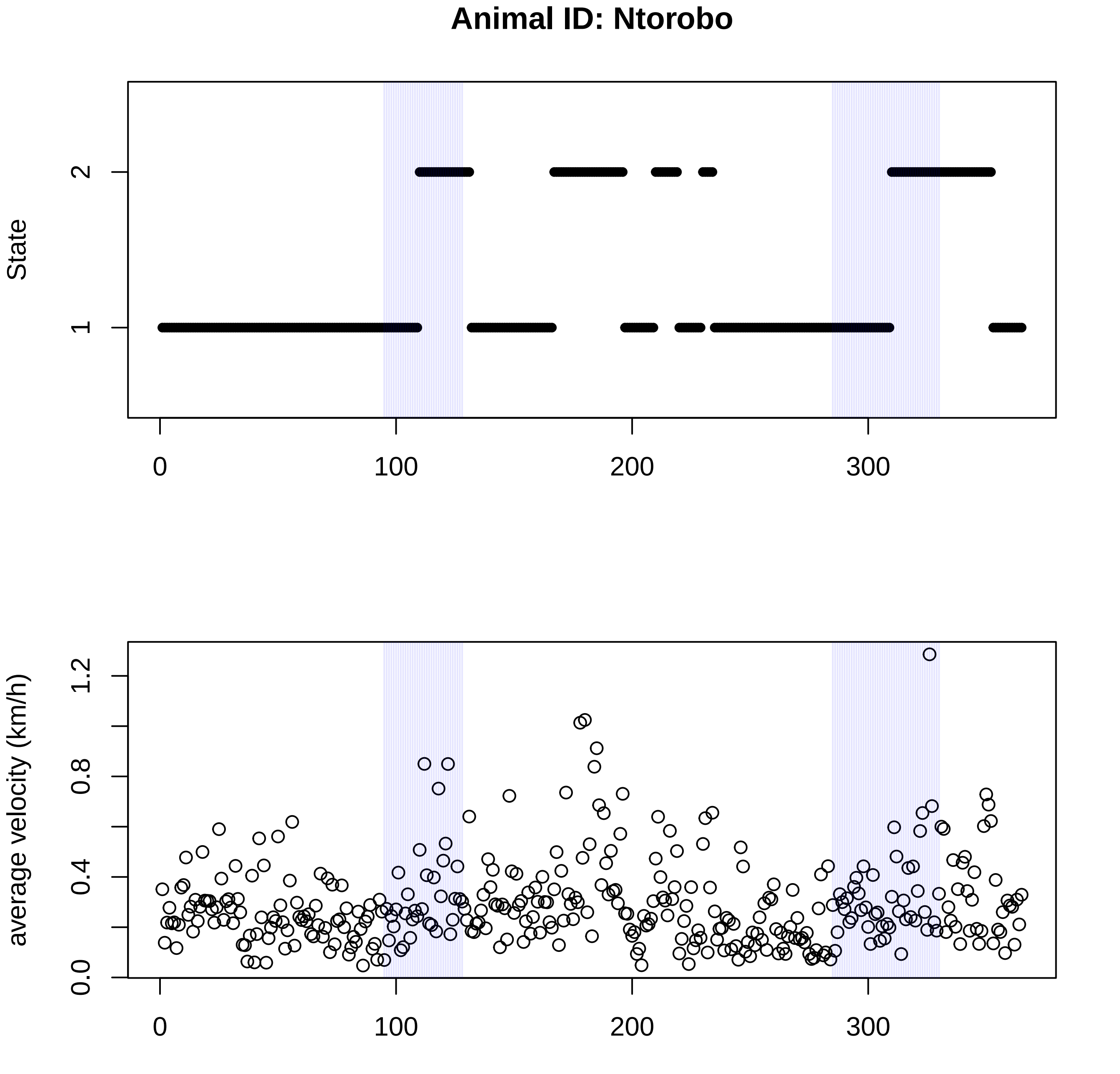
Animal ID: Ntorobo
State
1
2
0
100
200
300
average velocity (km/h)
0.0
0.4
0.8
1.2
0
100
200
300

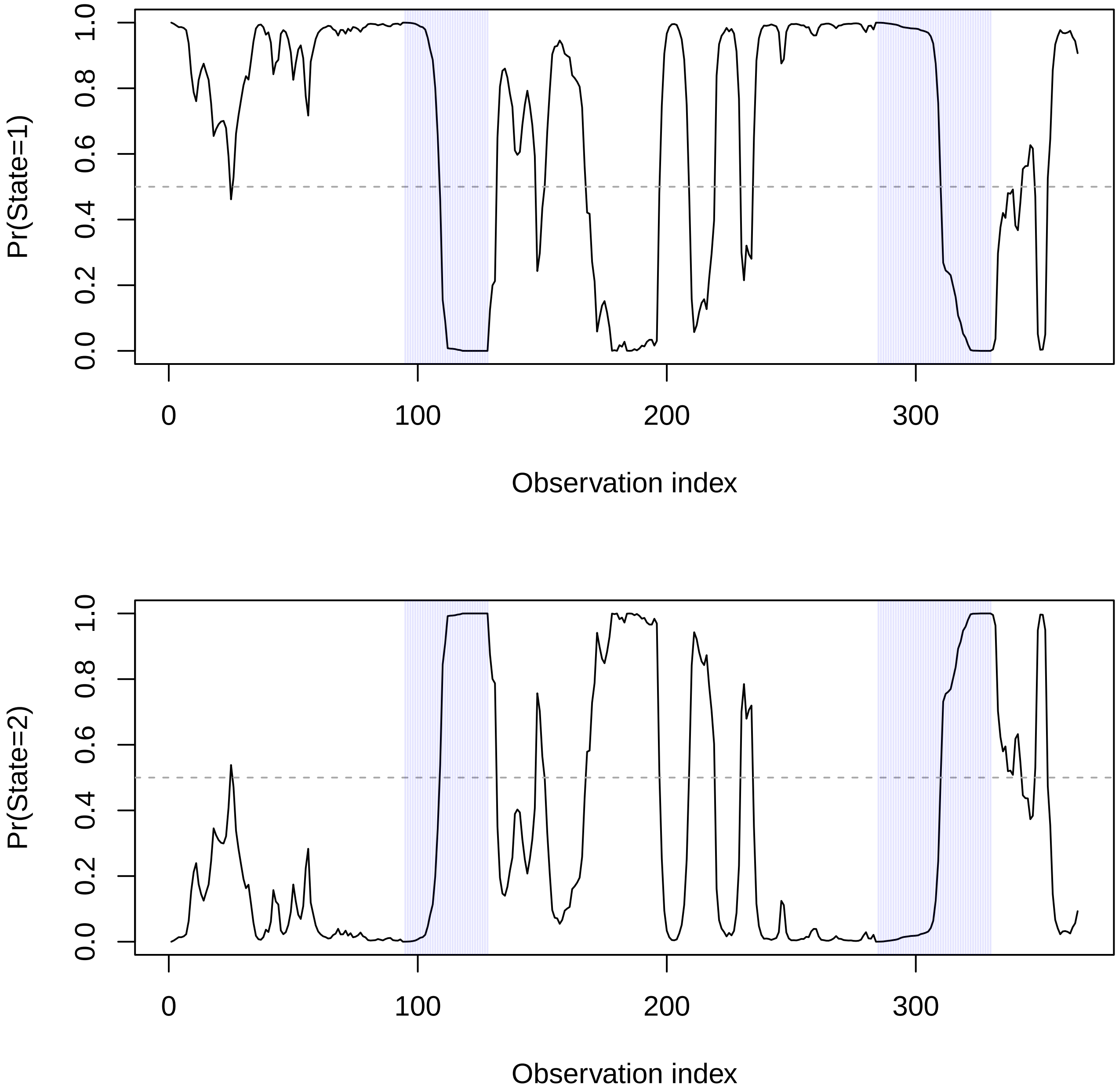

Pr(State=1)
0.0
0.2
0.4
0.6
0.8
1.0
0
100
200
300
Observation index
Pr(State=2)
0.0
0.2
0.4
0.6
0.8
1.0
0
100
200
300
Observation index

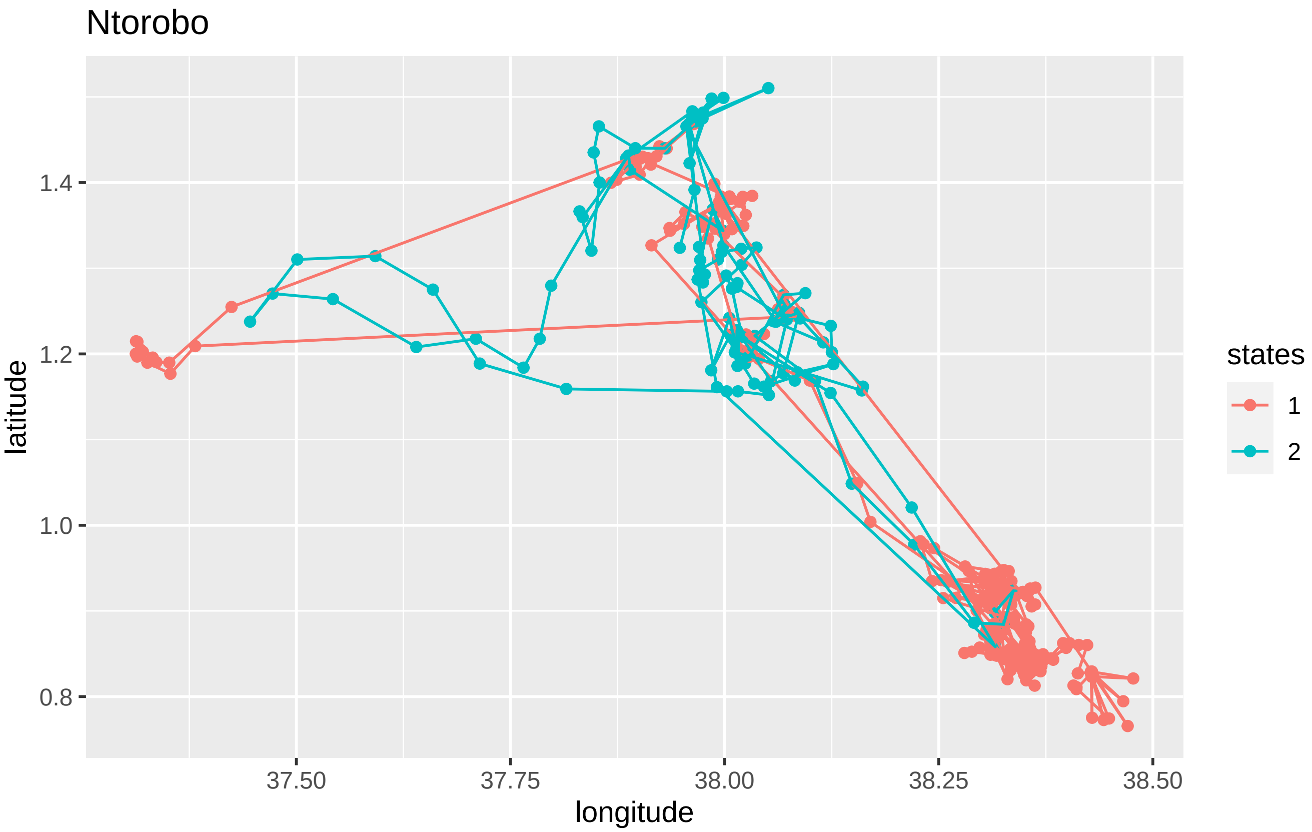

Ntorobo
latitude
longitude
1.4
1.2
1.0
0.8
37.50
37.75
38.00
38.25
38.50
states
1
2

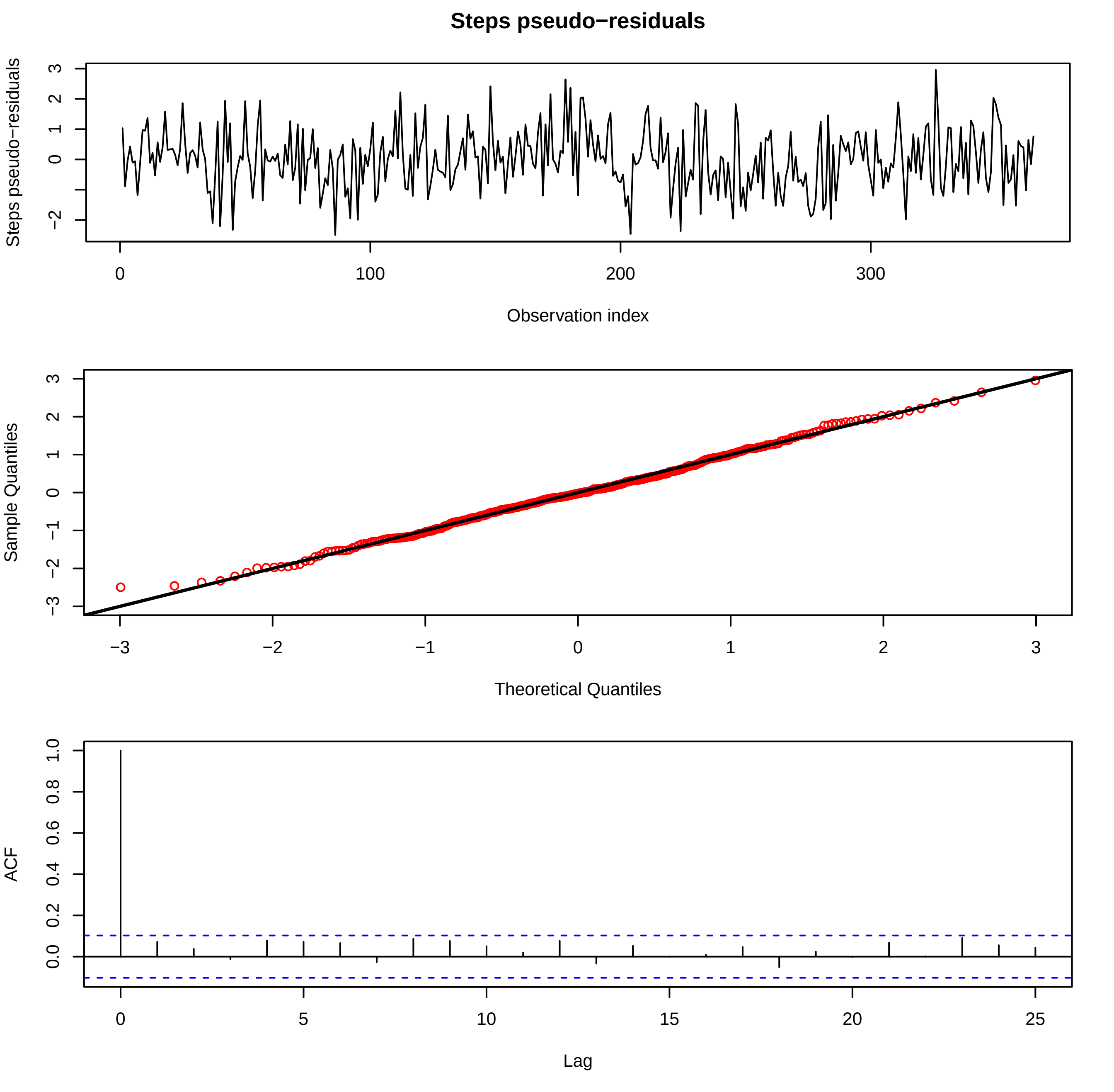

Steps pseudo−residuals
Steps pseudo−residuals
Observation index
0
100
200
300
−2
0
1
2
3
Sample Quantiles
Theoretical Quantiles
−3
−2
−1
0
1
2
3
ACF
Lag
0
5
10
15
20
25
0.0
0.2
0.4
0.6
0.8
1.0

Animal ID: Nutmeg

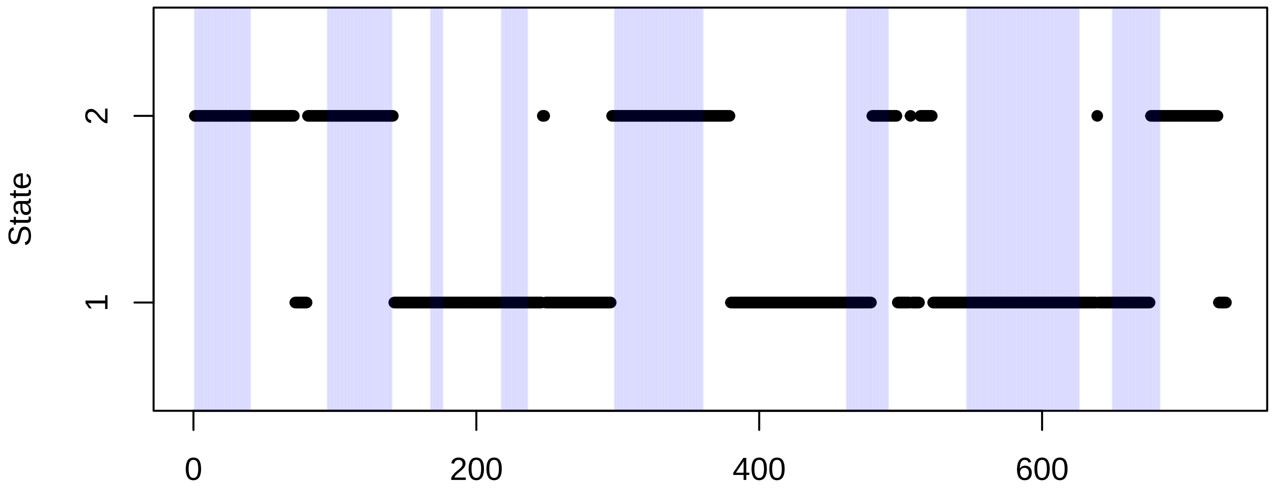

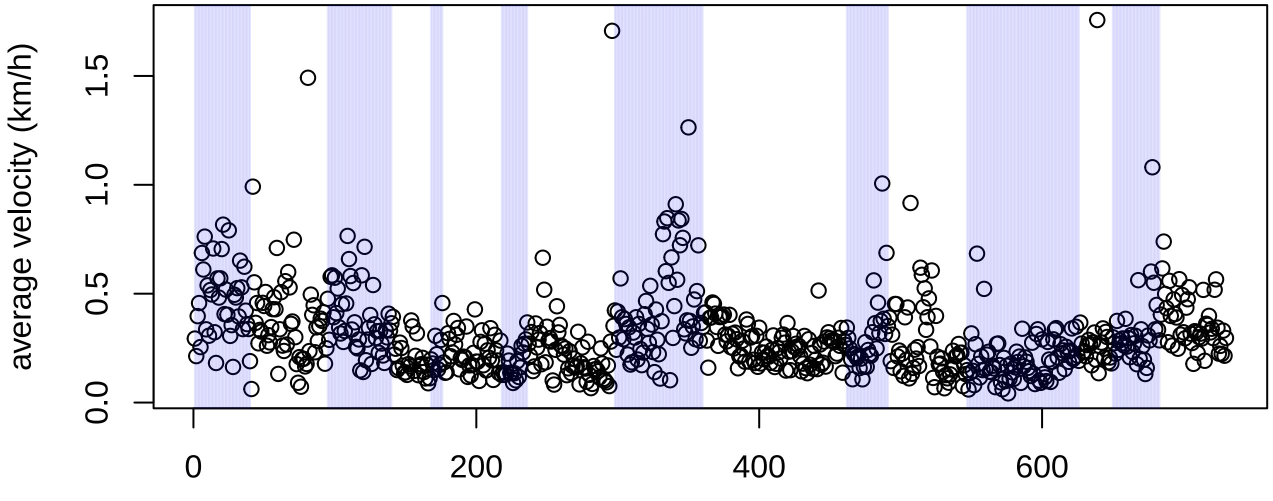

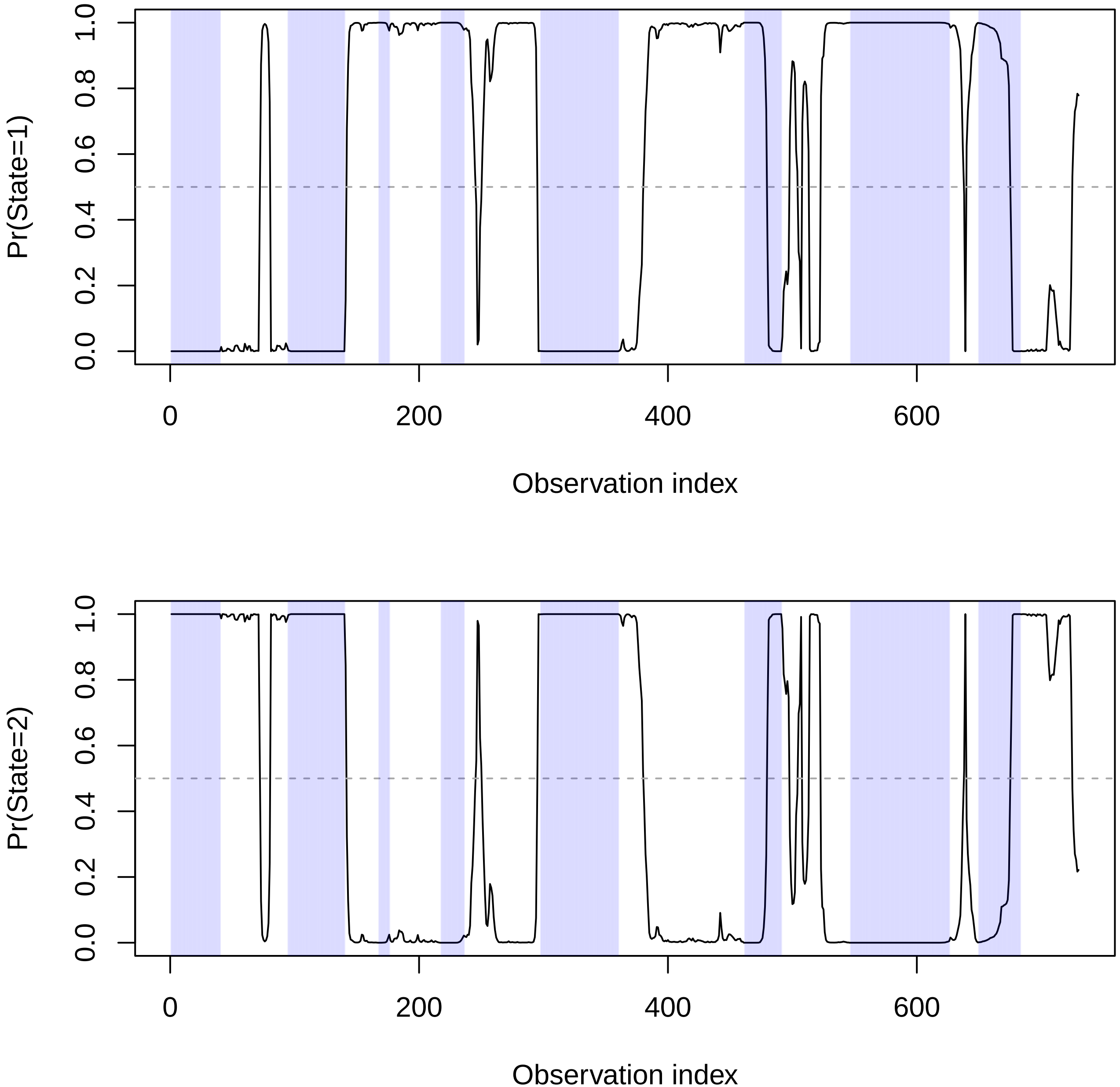

Pr(State=1)
0.0
0.2
0.4
0.6
0.8
1.0
0
200
400
600
Observation index
Pr(State=2)
0.0
0.2
0.4
0.6
0.8
1.0
0
200
400
600
Observation index

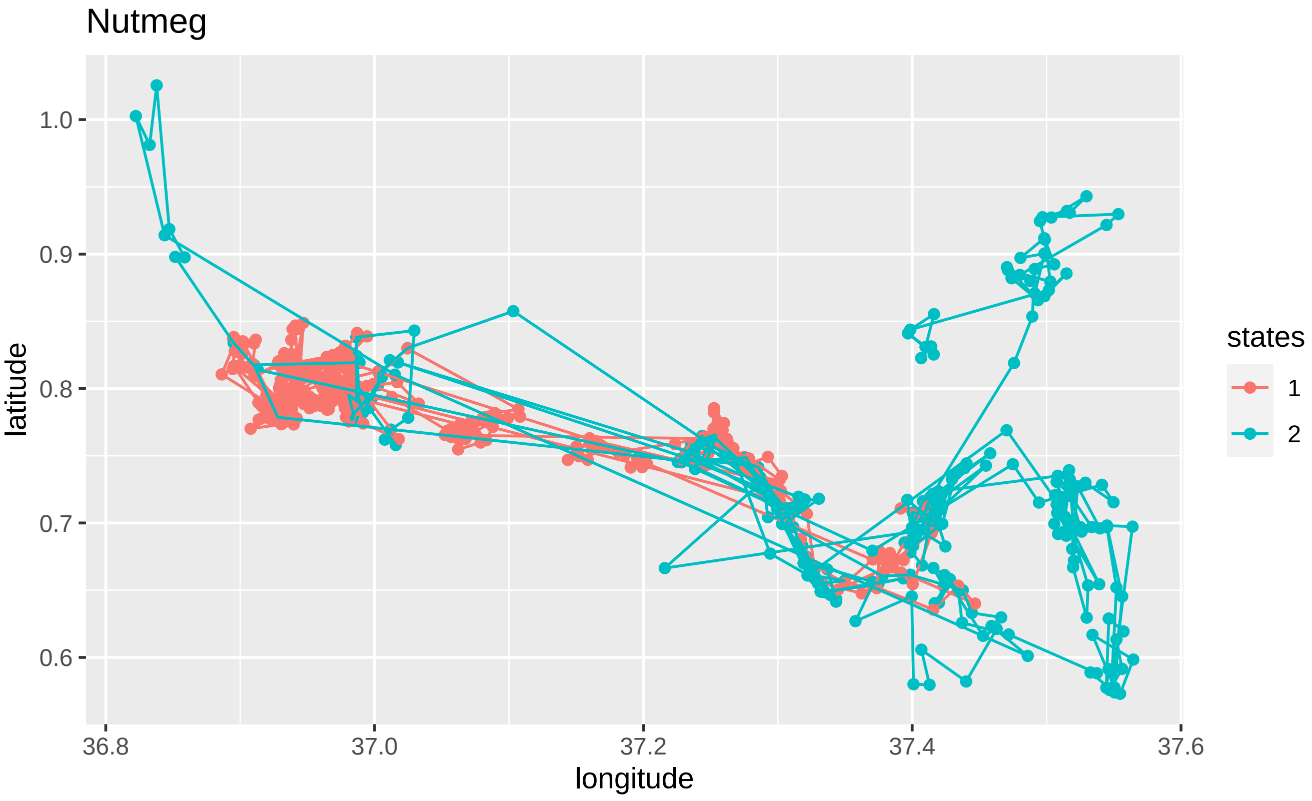

Nutmeg
1.0
0.9
0.8
0.7
0.6
latitude
36.8
37.0
37.2
37.4
37.6
longitude
states
1
2

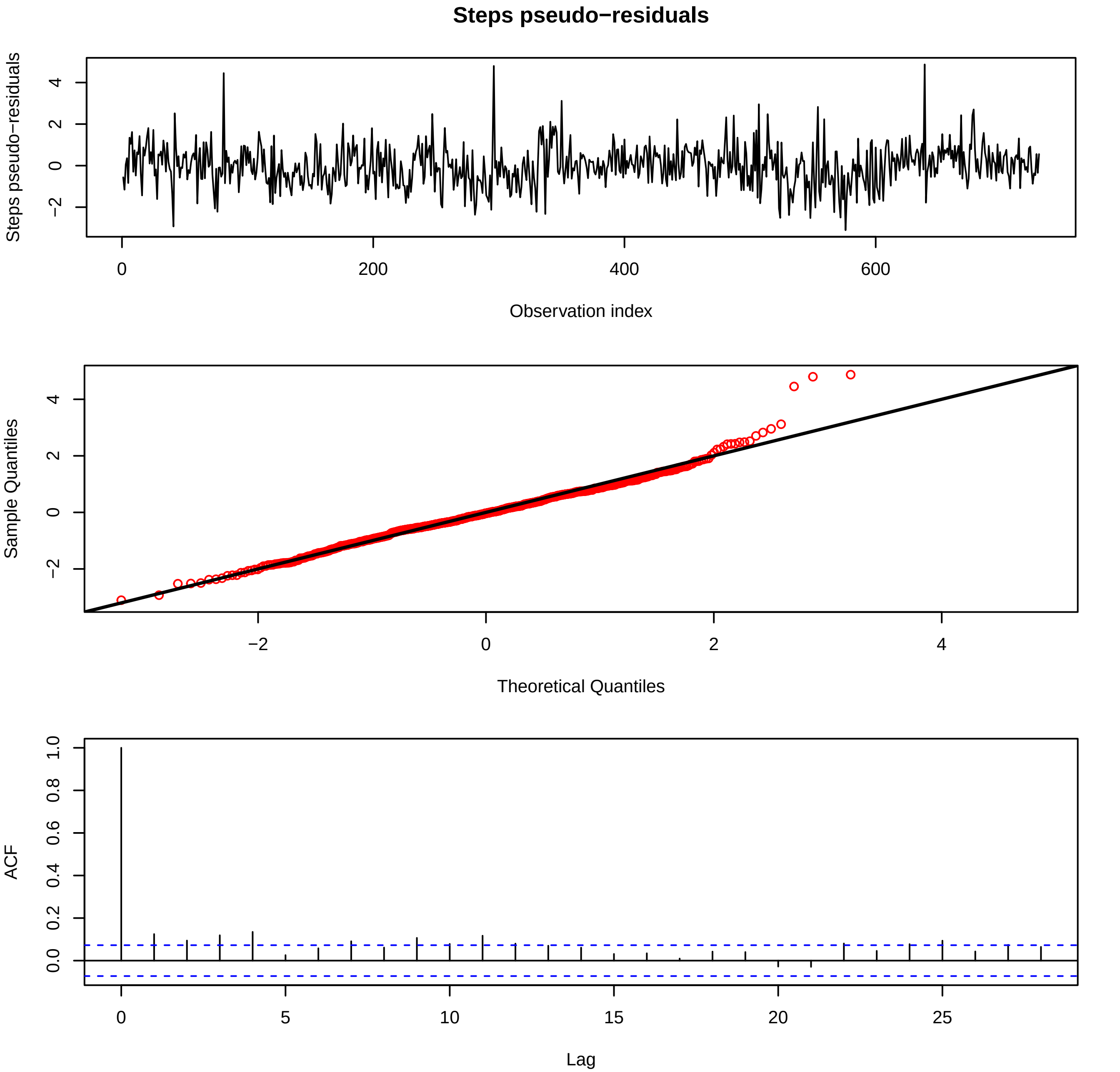

Steps pseudo−residuals
Steps pseudo−residuals
−2
0
2
4
0
200
400
600
Observation index
Sample Quantiles
−2
0
2
4
−2
0
2
4
Theoretical Quantiles
ACF
0.0
0.2
0.4
0.6
0.8
1.0
0
5
10
15
20
25
Lag

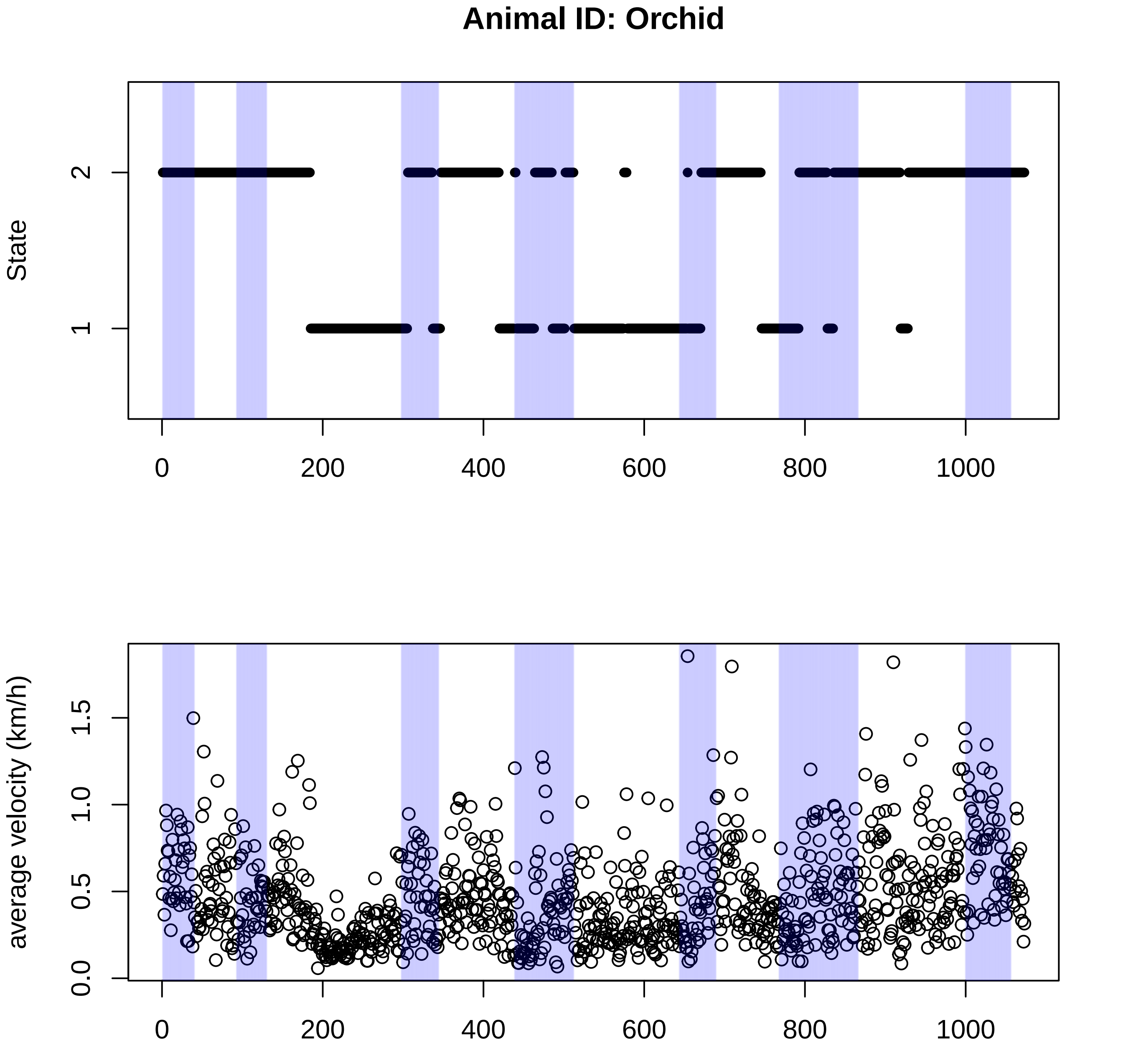
Animal ID: Orchid
State
2
1
0
200
400
600
800
1000
average velocity (km/h)
0.0
0.5
1.0
1.5
0
200
400
600
800
1000

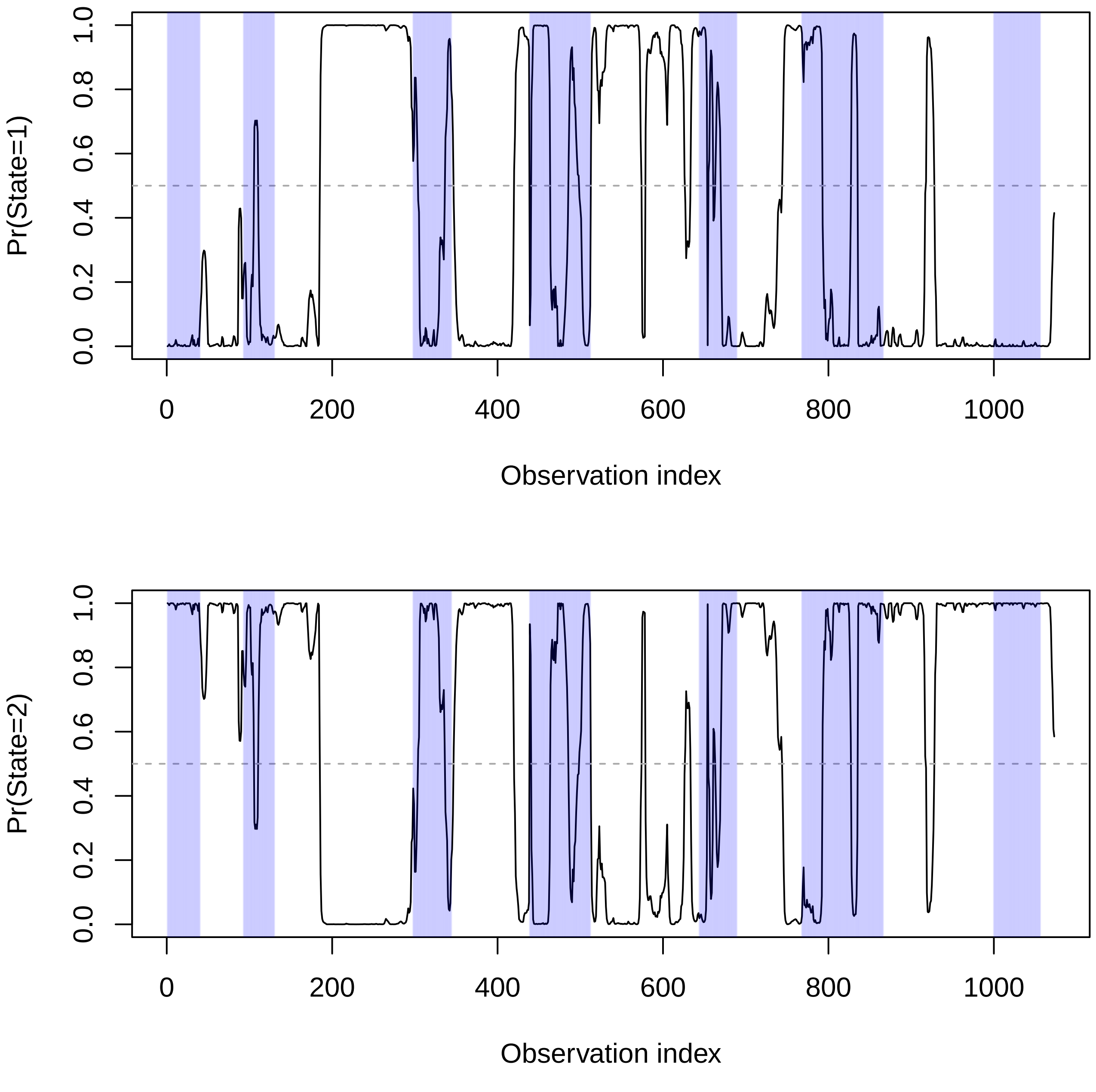

Pr(State=1)
1.0
0.8
0.6
0.4
0.2
0.0
0
200
400
600
800
1000
Observation index
Pr(State=2)
1.0
0.8
0.6
0.4
0.2
0.0
0
200
400
600
800
1000
Observation index

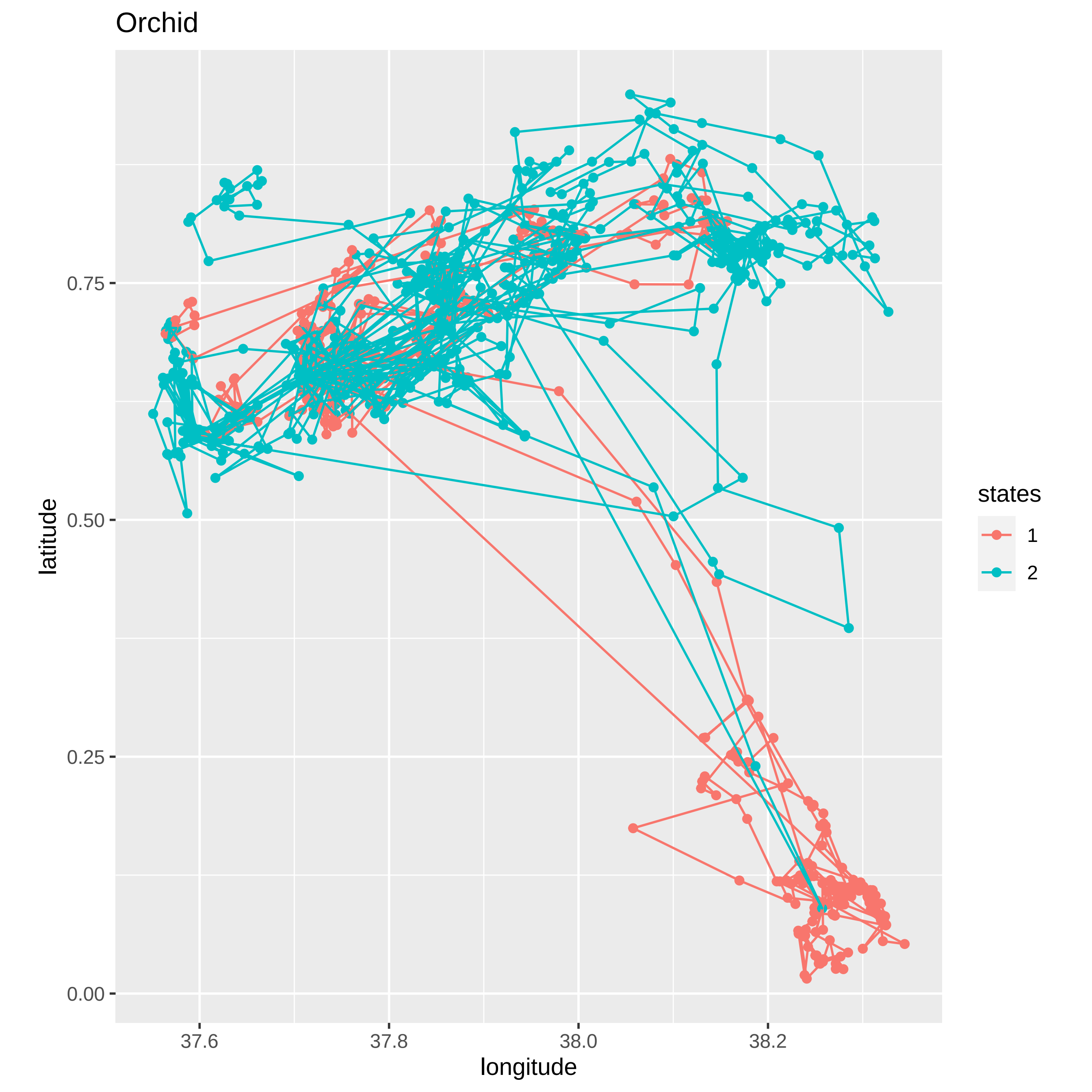

Orchid
latitude
longitude
0.00
0.25
0.50
0.75
37.6
37.8
38.0
38.2
states
1
2

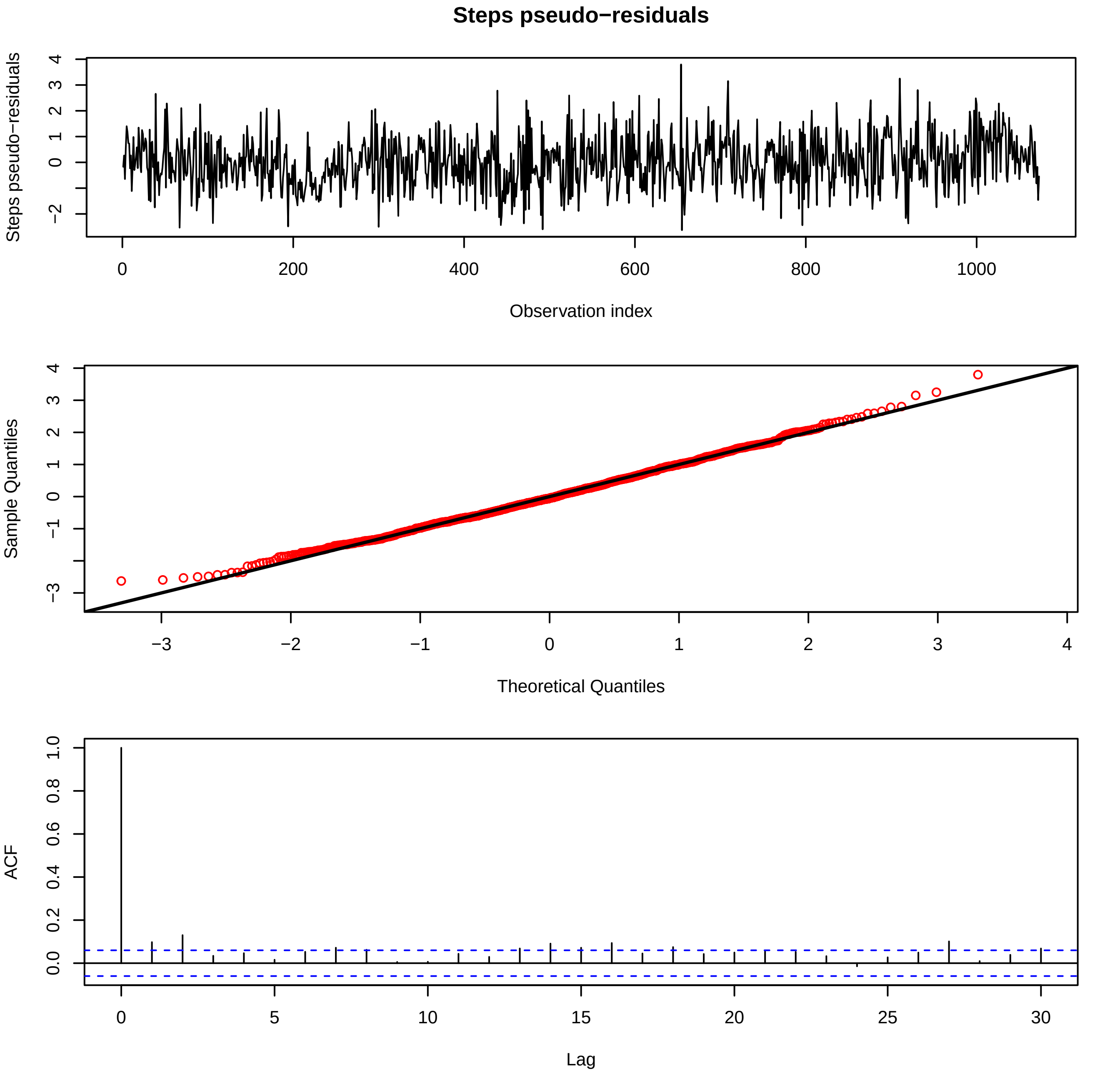

Steps pseudo−residuals
Steps pseudo−residuals
Observation index
Sample Quantiles
Theoretical Quantiles
ACF
Lag

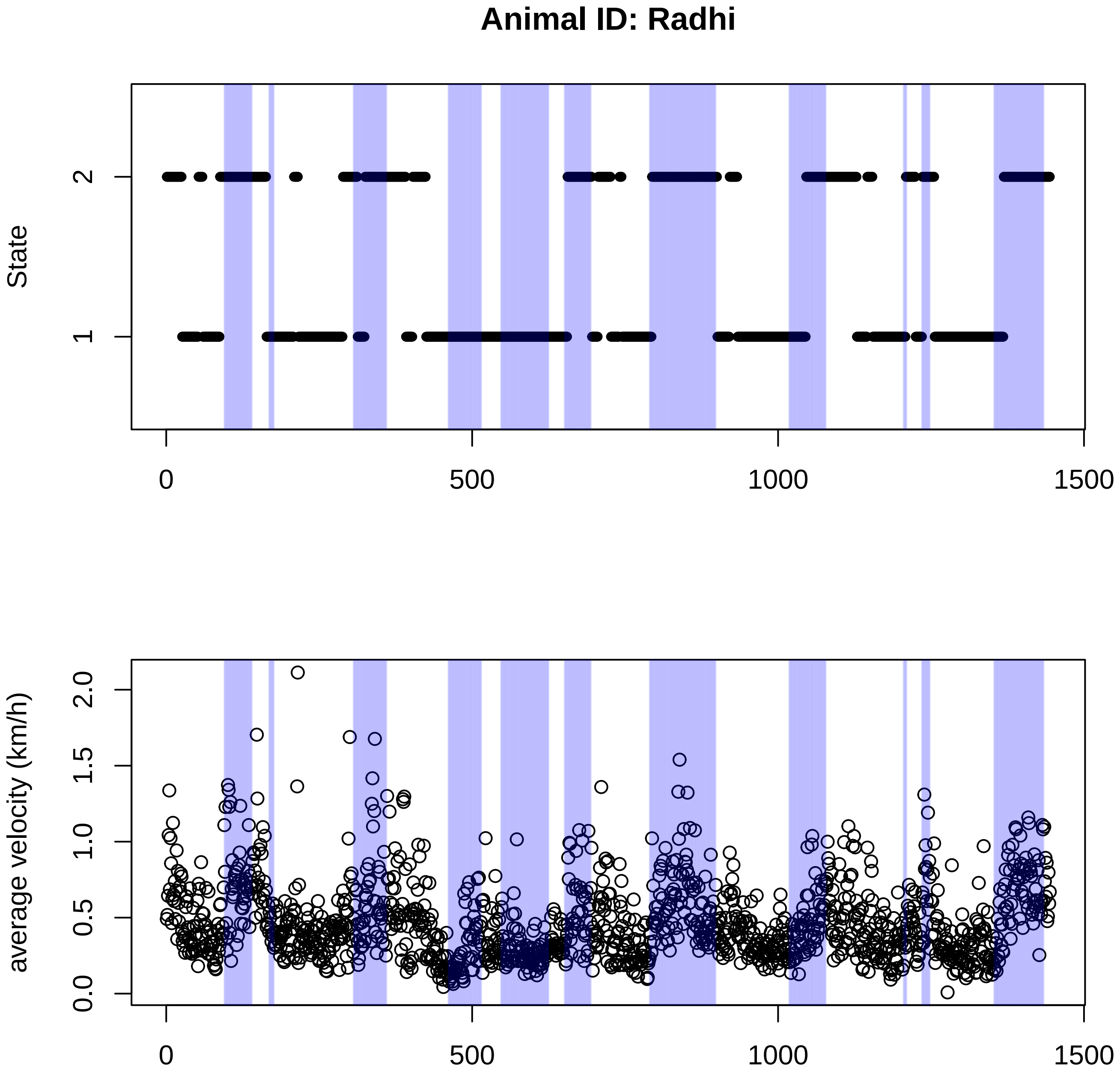

Animal ID: Radhi
State
2
1
0
500
1000
1500
average velocity (km/h)
0.0
0.5
1.0
1.5
2.0

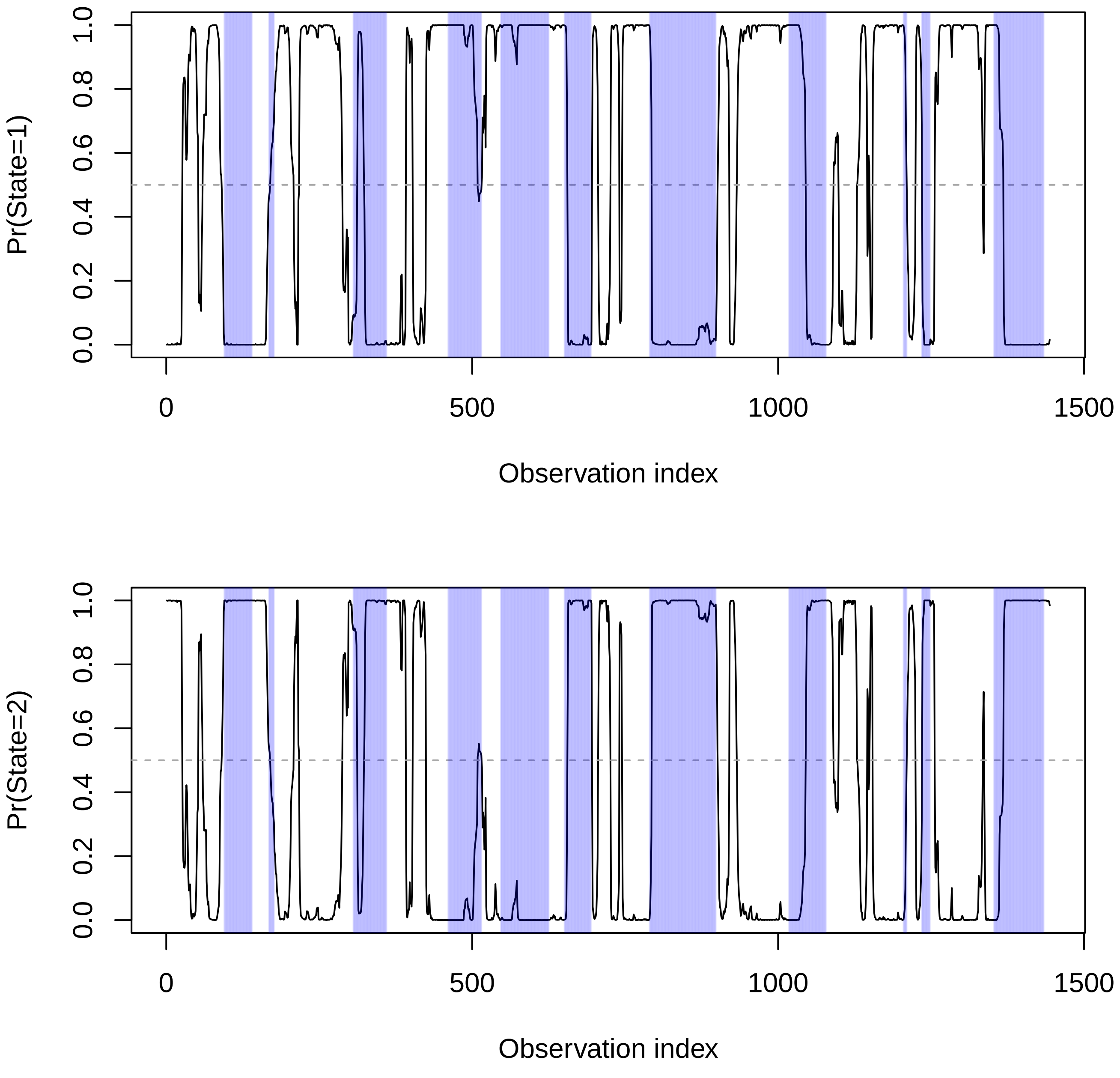
Pr(State=1)
0.0
0.2
0.4
0.6
0.8
1.0
0
500
1000
1500
Observation index
Pr(State=2)
0.0
0.2
0.4
0.6
0.8
1.0
0
500
1000
1500
Observation index

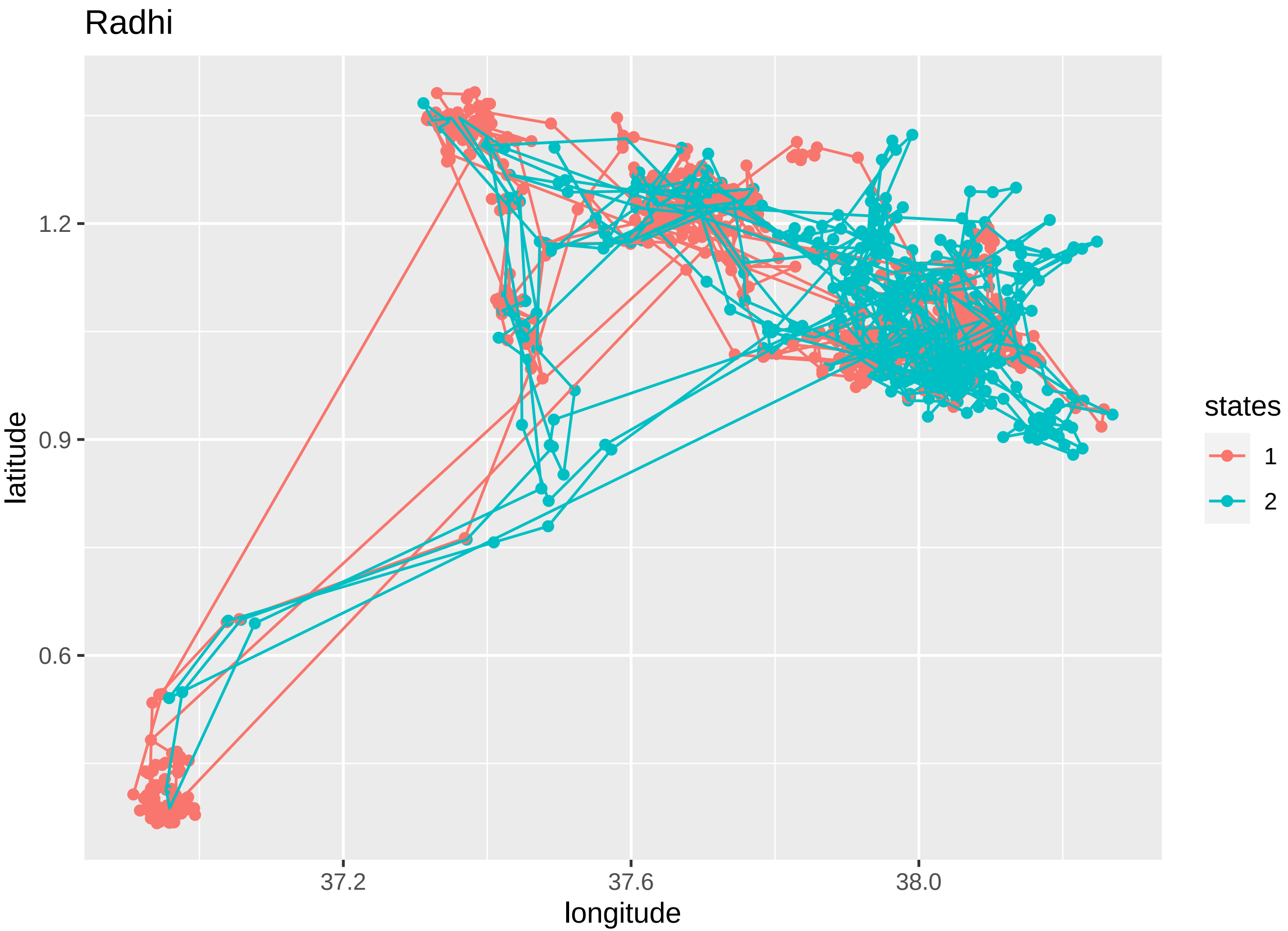

Radhi
latitude
1.2
0.9
0.6
37.2
37.6
38.0
longitude
states
1
2

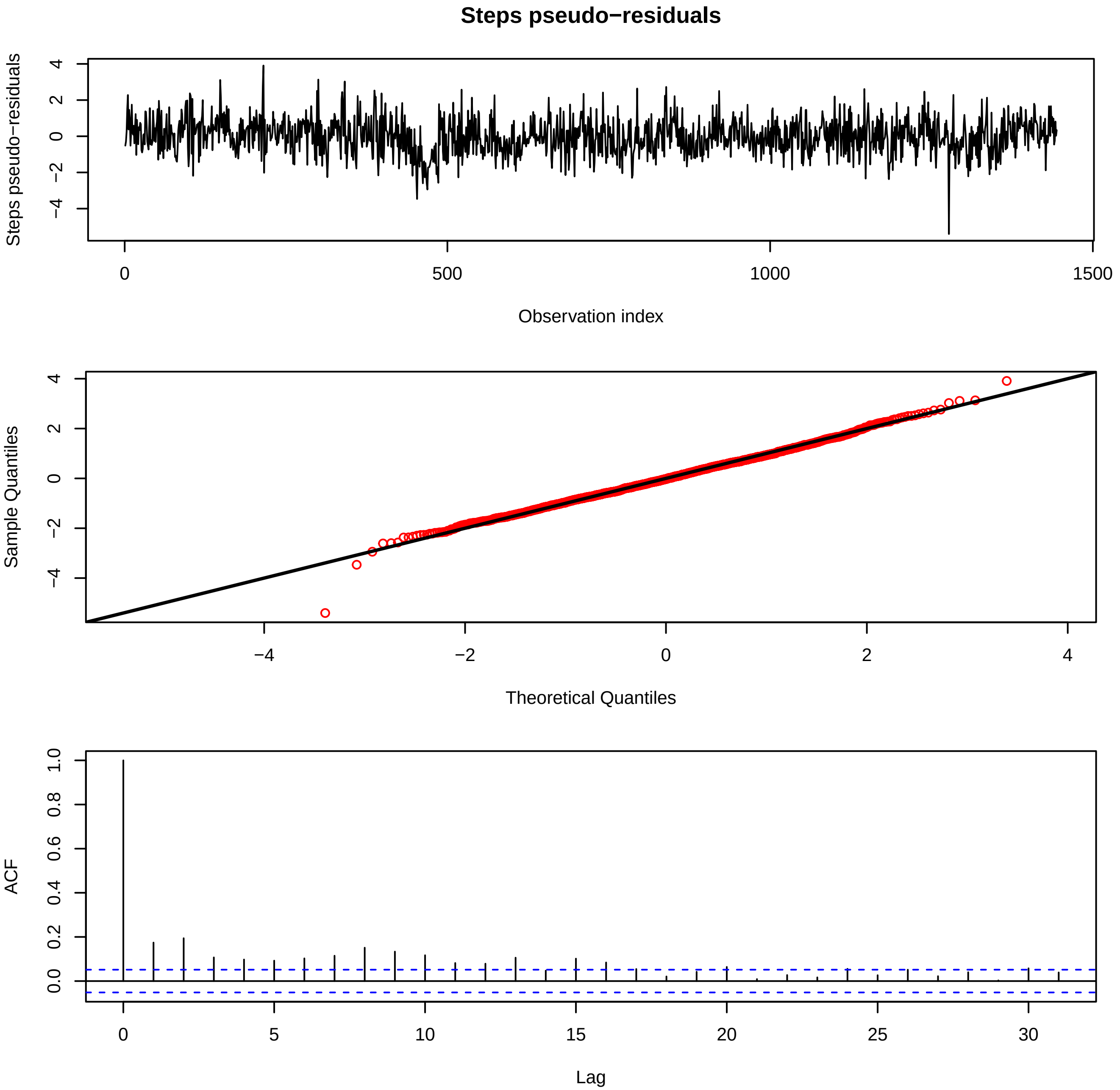

Steps pseudo−residuals
Steps pseudo−residuals
Observation index
Sample Quantiles
Theoretical Quantiles
ACF
Lag

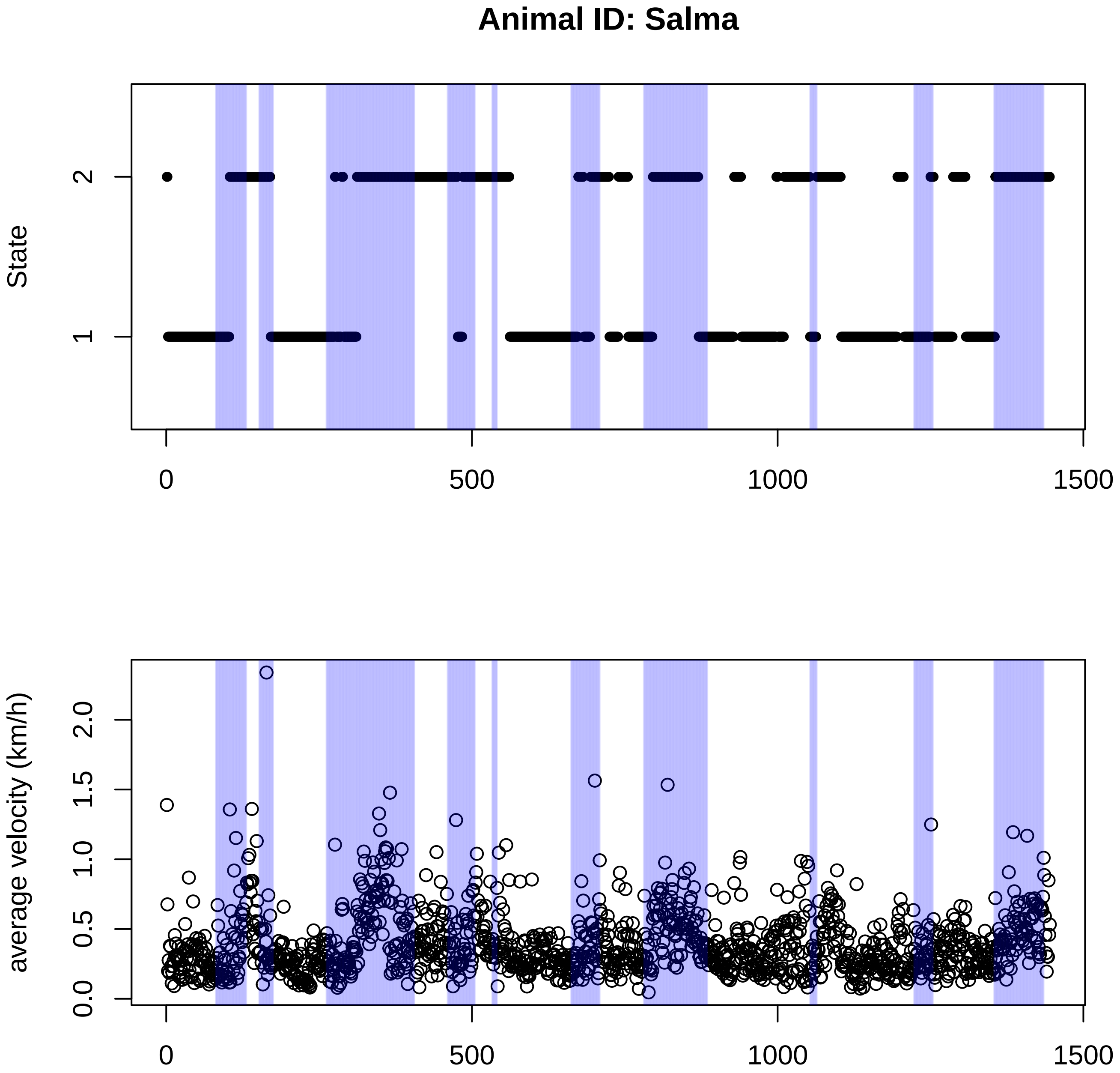

Animal ID: Salma
State
2
1
0
500
1000
1500
average velocity (km/h)
0.0
0.5
1.0
1.5
2.0

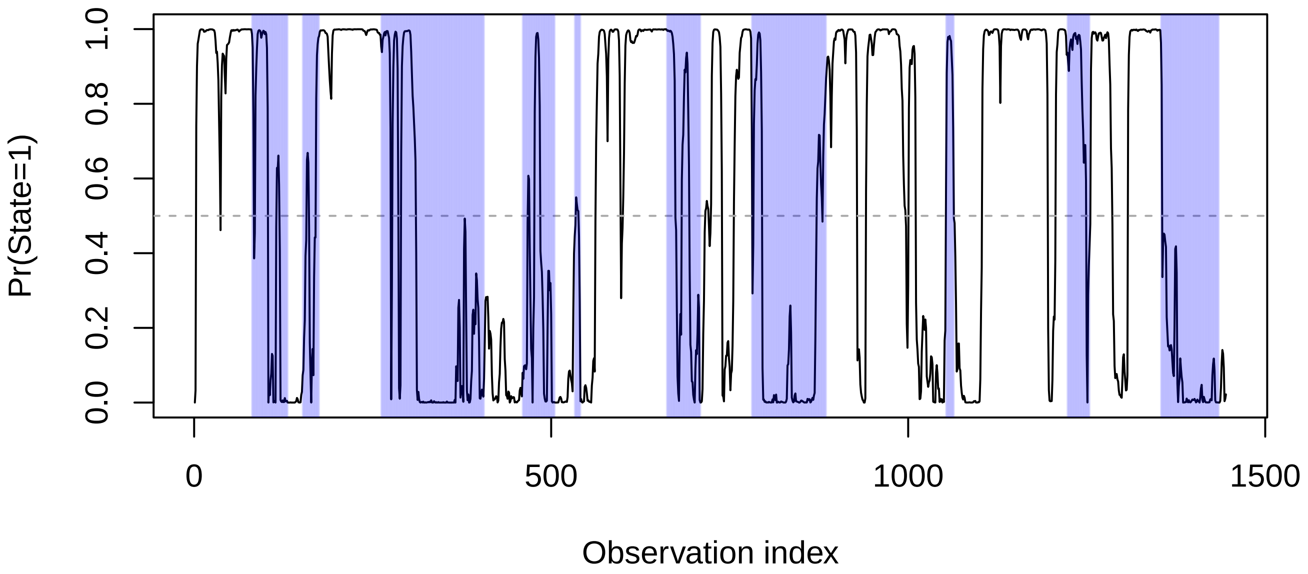

Pr(State=1)
0.0
0.2
0.4
0.6
0.8
1.0
0
500
1000
1500
Observation index

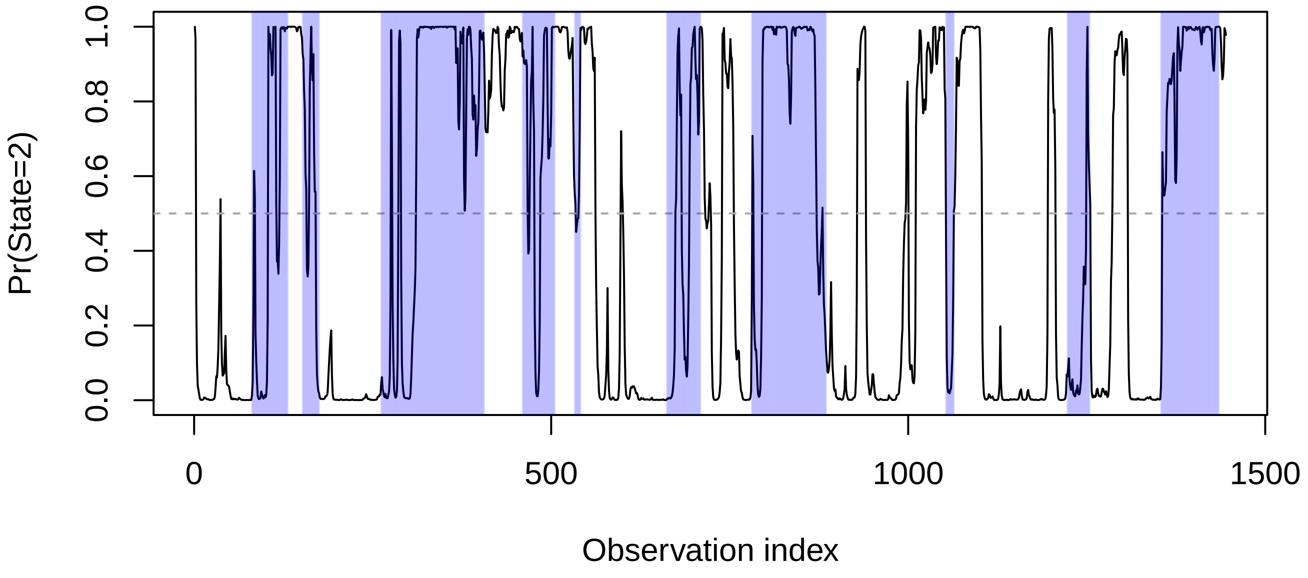

Pr(State=2)
0.0
0.2
0.4
0.6
0.8
1.0
0
500
1000
1500
Observation index

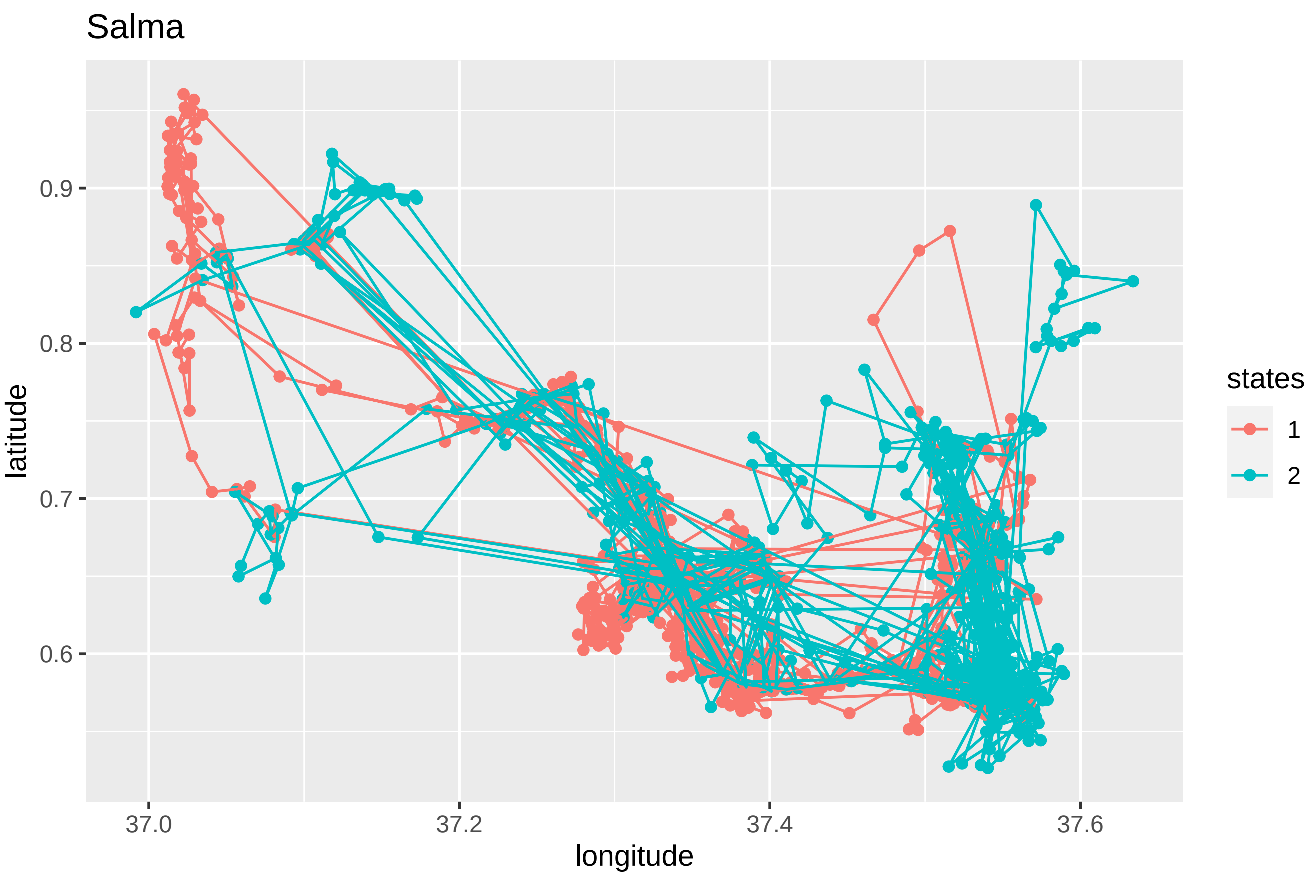

Salma
latitude
longitude
0.9
0.8
0.7
0.6
37.0
37.2
37.4
37.6
states
1
2

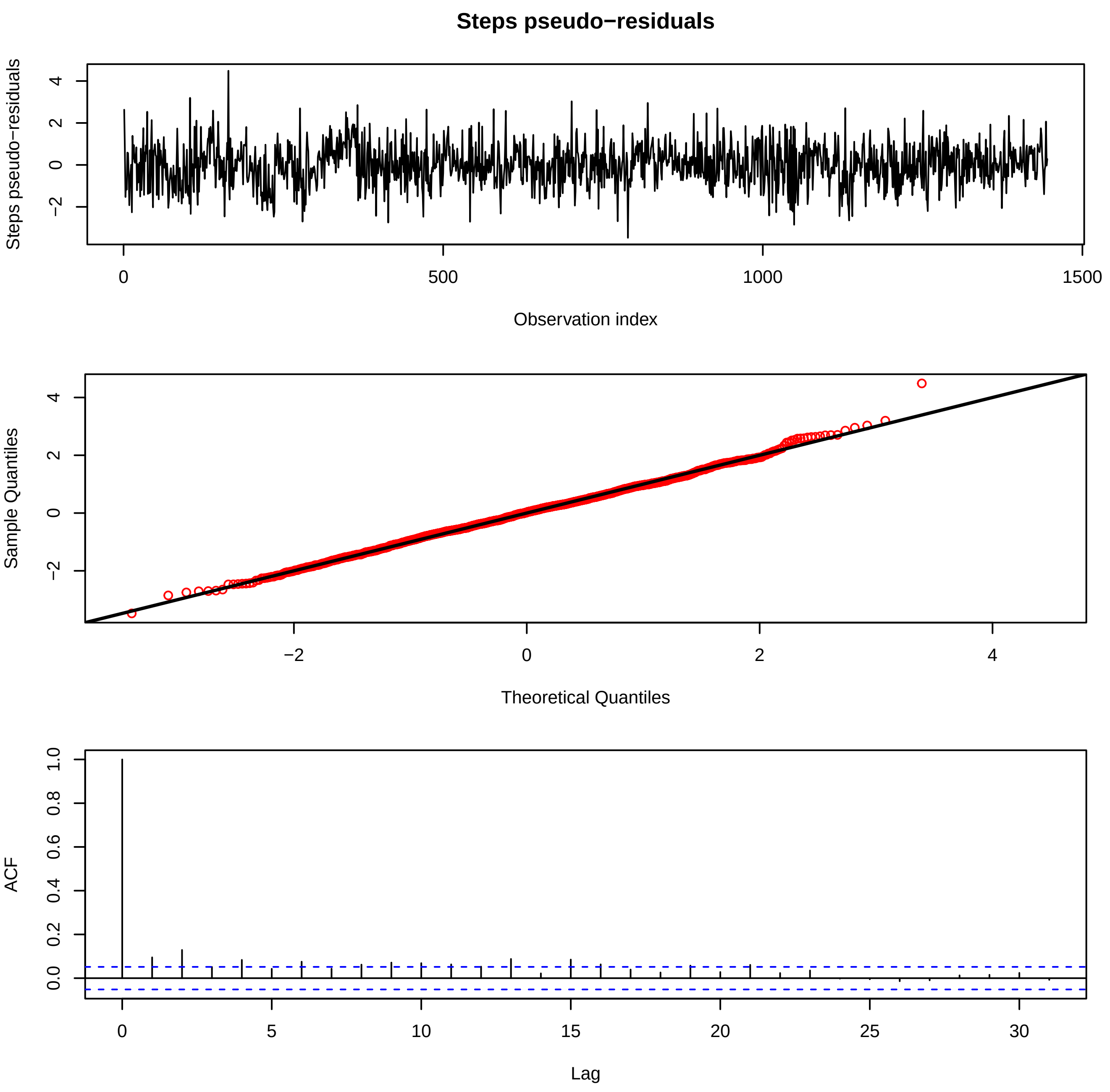

Steps pseudo−residuals
Steps pseudo−residuals
4
2
0
−2
0
500
1000
1500
Observation index
Sample Quantiles
4
2
0
−2
−2
0
2
4
Theoretical Quantiles
ACF
1.0
0.8
0.6
0.4
0.2
0.0
0
5
10
15
20
25
30
Lag

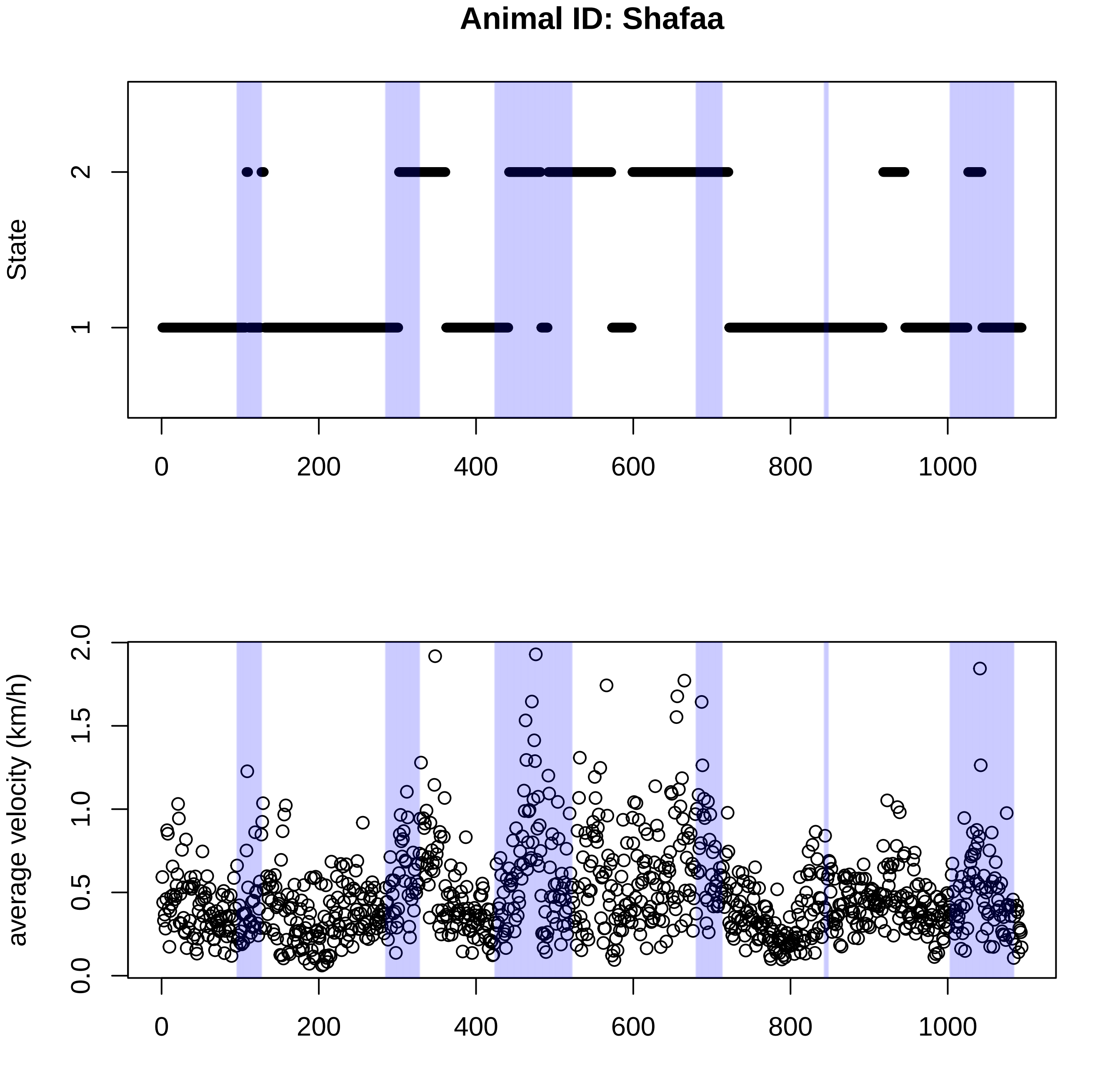

Animal ID: Shafaa
State
1
2
0
200
400
600
800
1000
average velocity (km/h)
0.0
0.5
1.0
1.5
2.0

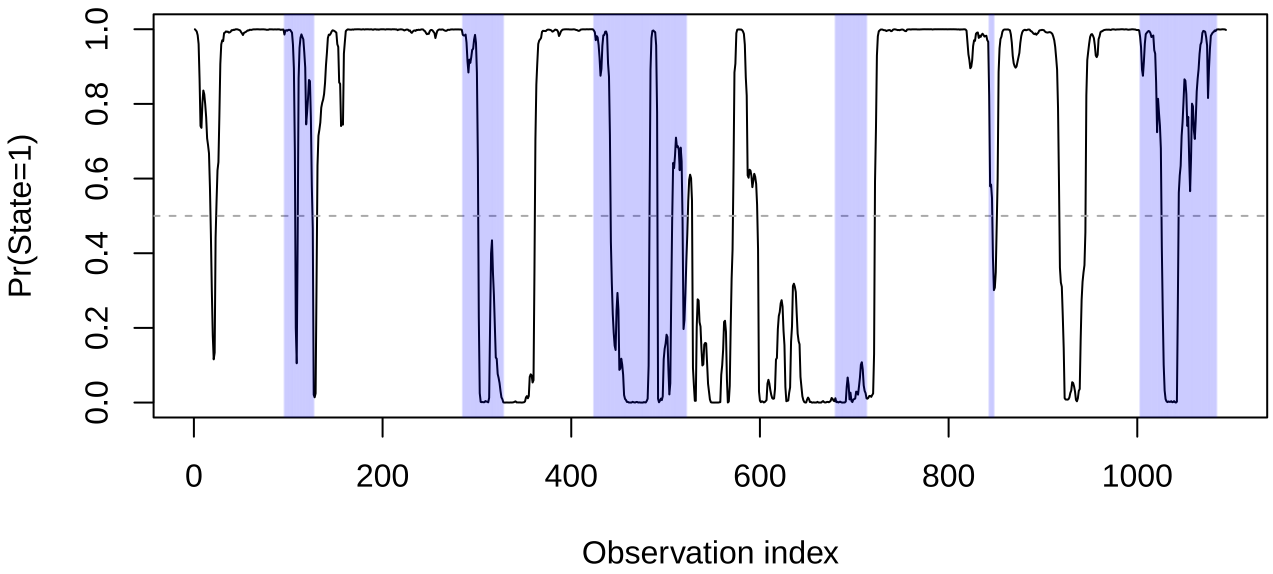

Pr(State=1)
1.0
0.8
0.6
0.4
0.2
0.0
0
200
400
600
800
1000
Observation index

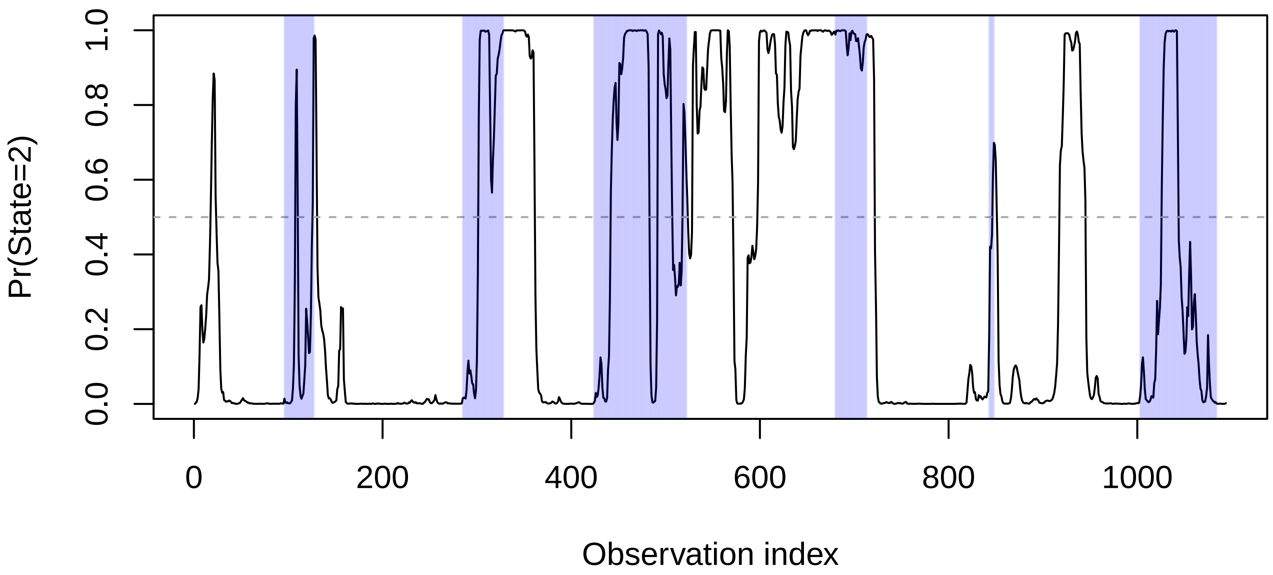

Pr(State=2)
1.0
0.8
0.6
0.4
0.2
0.0
0
200
400
600
800
1000
Observation index

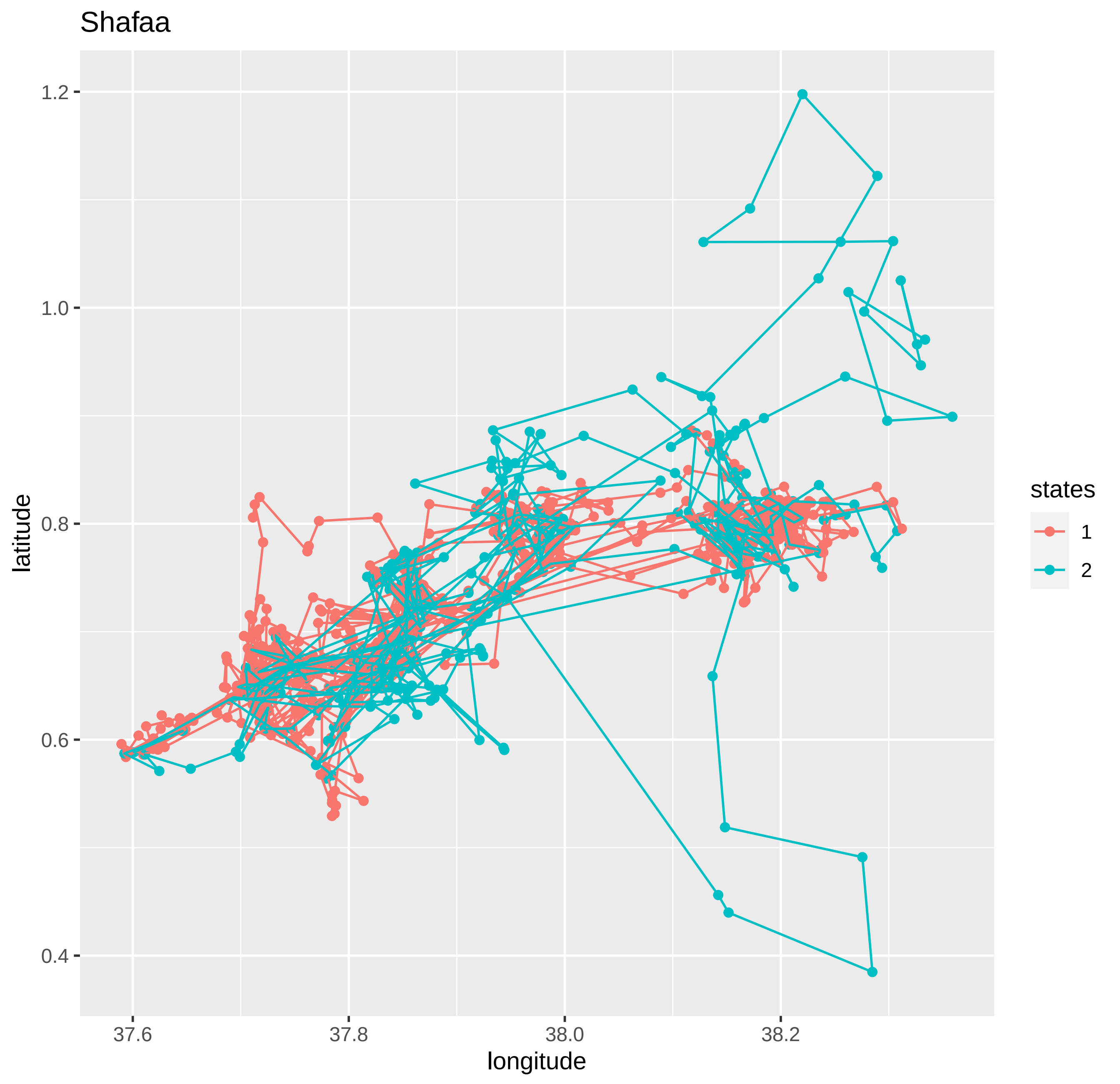

Shafaa
latitude
1.2
1.0
0.8
0.6
0.4
longitude
37.6
37.8
38.0
38.2
states
1
2

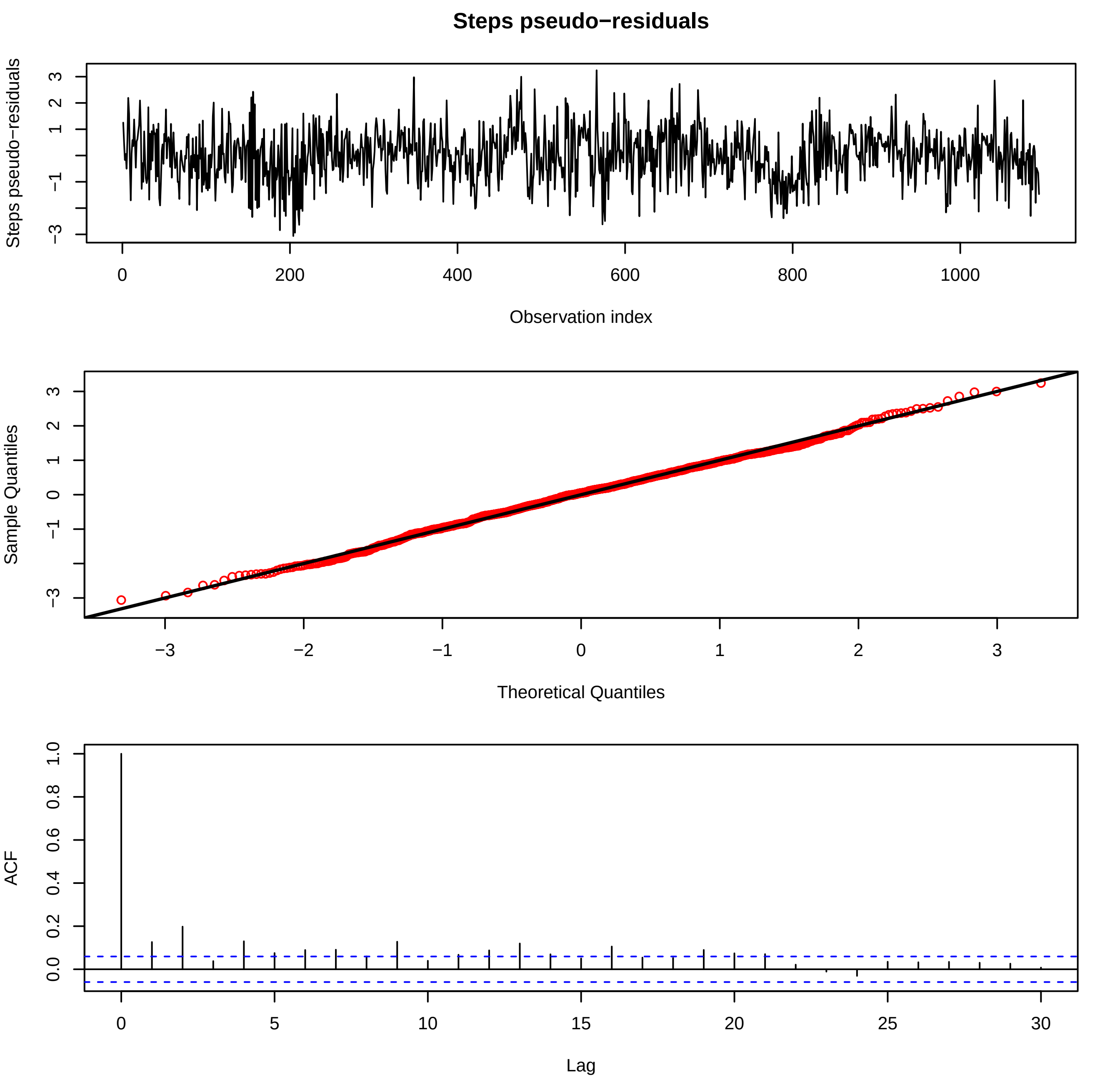

Steps pseudo−residuals
Steps pseudo−residuals
Observation index
0
200
400
600
800
1000
−3
−1
1
2
3
Sample Quantiles
Theoretical Quantiles
−3
−2
−1
0
1
2
3
ACF
Lag
0.0
0.2
0.4
0.6
0.8
1.0
0
5
10
15
20
25
30

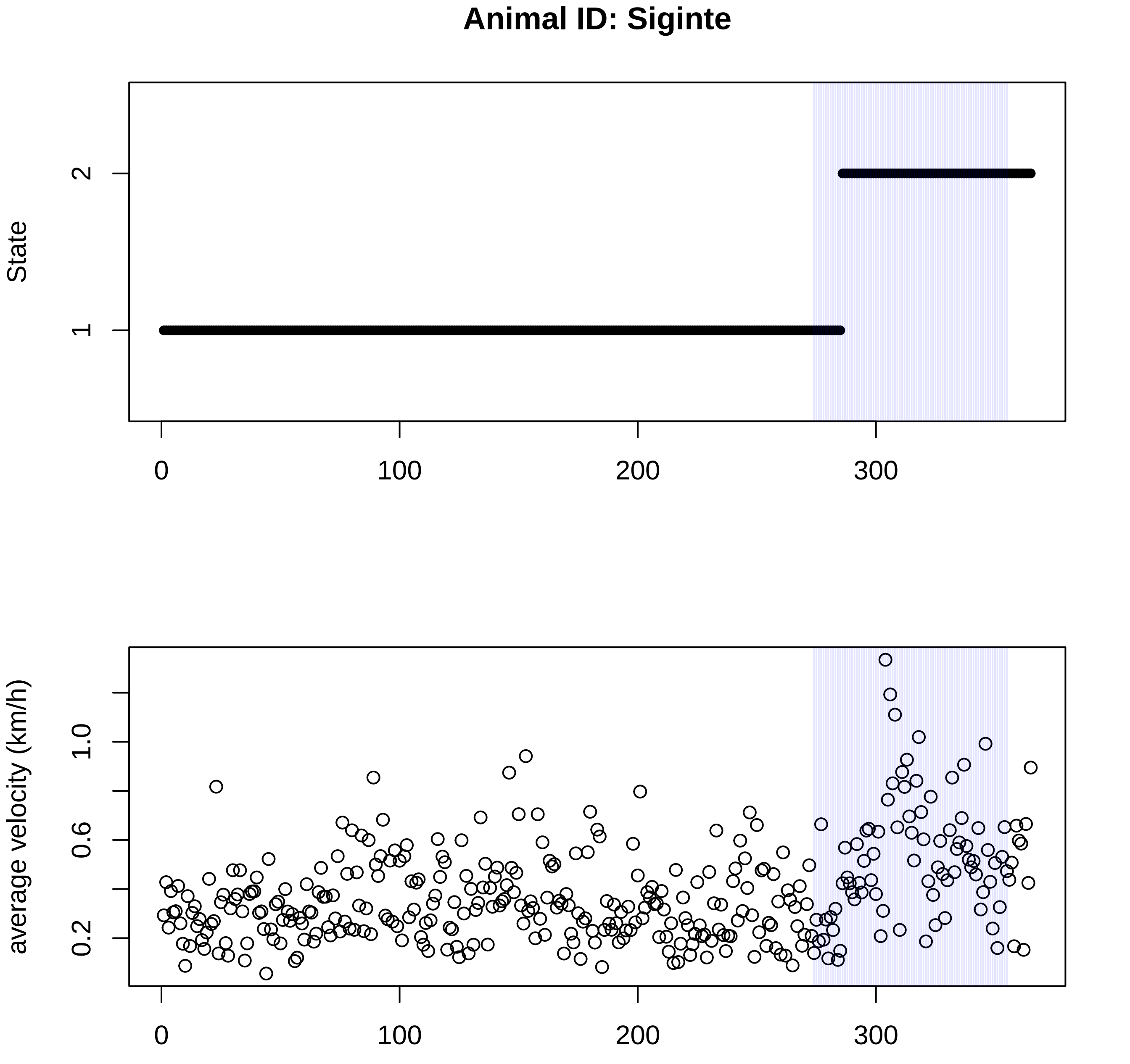
Animal ID: Siginte
State
1
2
0
100
200
300
average velocity (km/h)
0.2
0.6
1.0
0
100
200
300

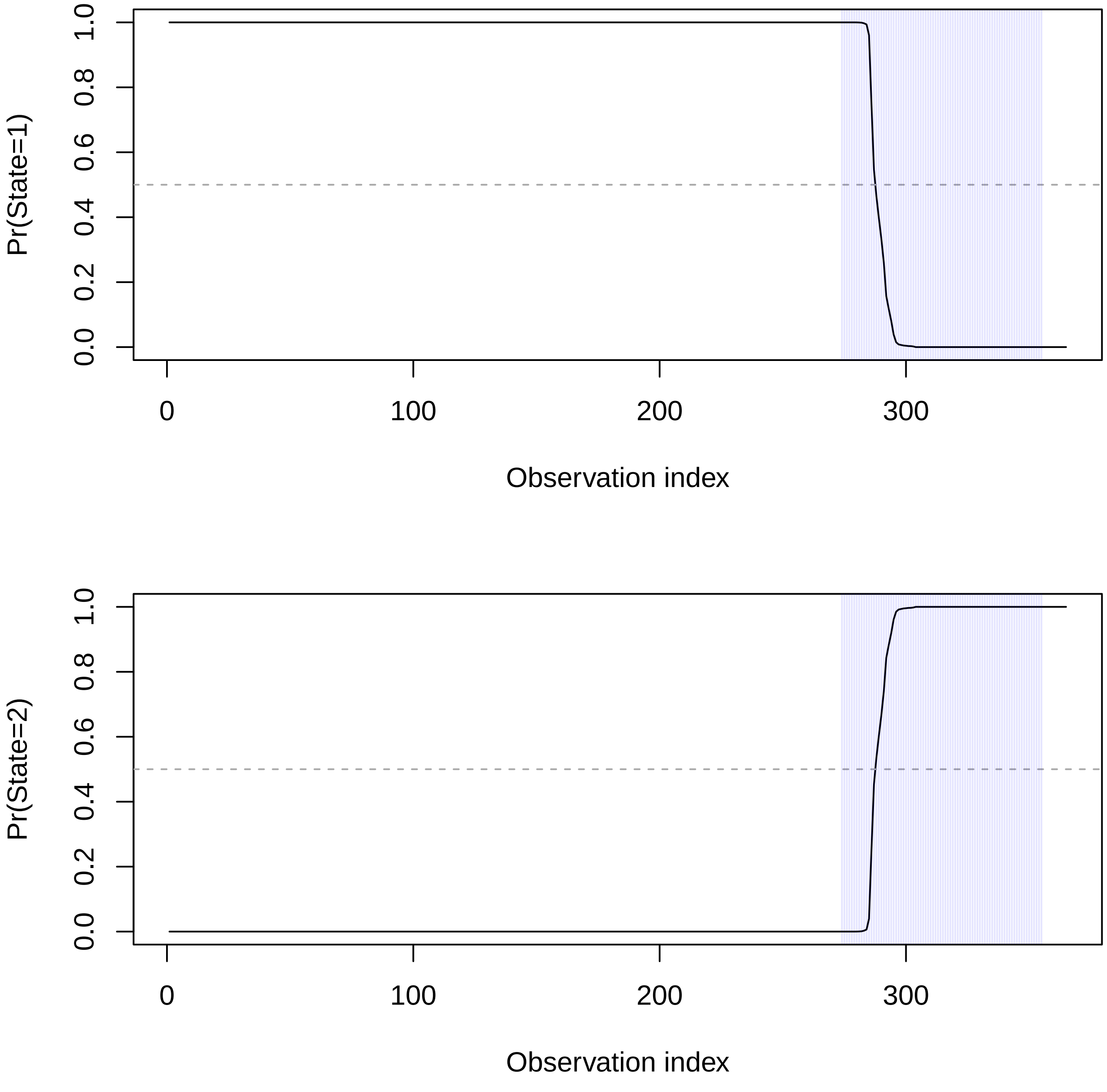

Pr(State=1)
1.0
0.8
0.6
0.4
0.2
0.0
0
100
200
300
Observation index
Pr(State=2)
1.0
0.8
0.6
0.4
0.2
0.0
0
100
200
300
Observation index

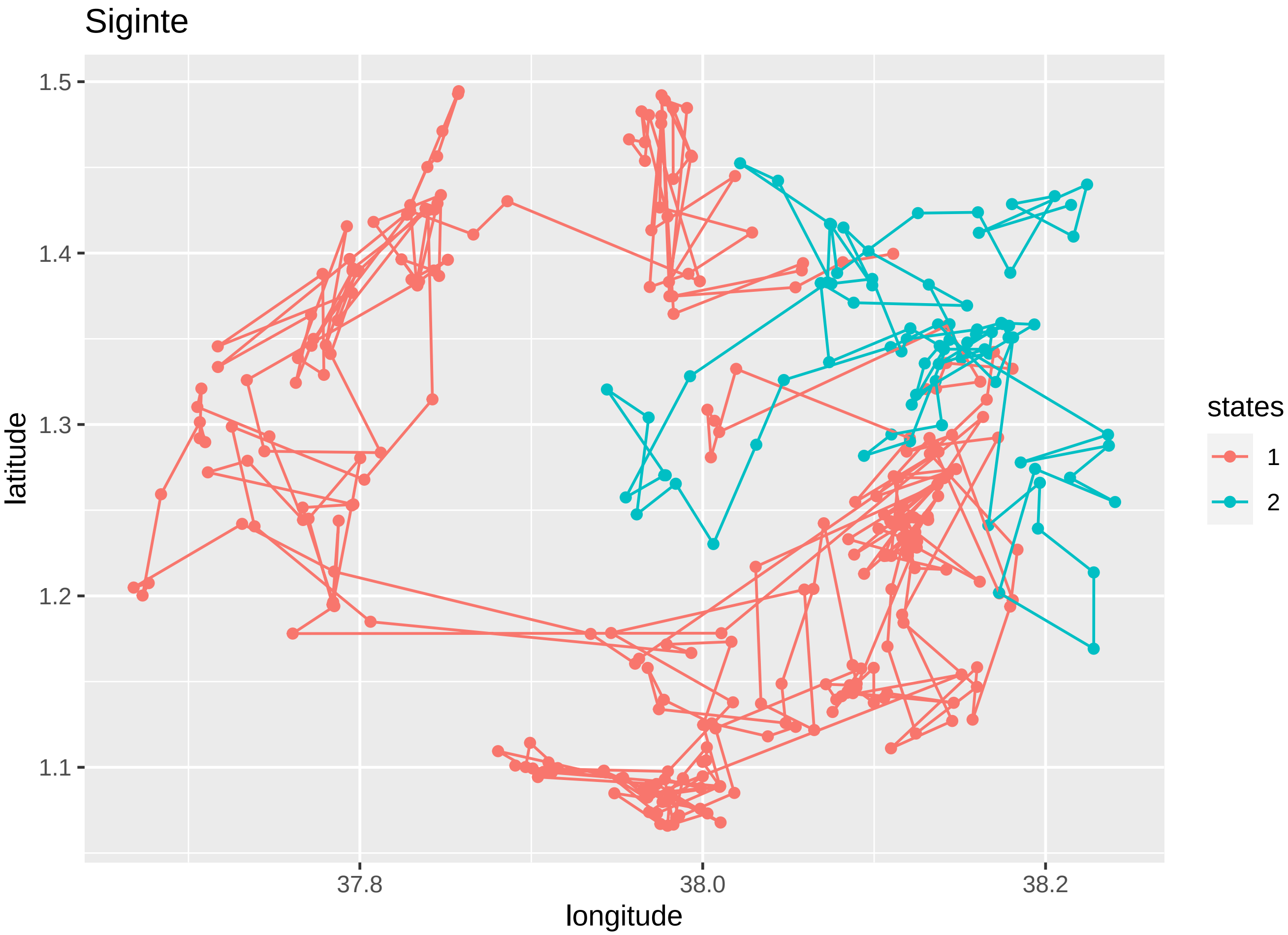

Siginte
1.5
1.4
1.3
1.2
1.1
latitude
37.8
38.0
38.2
longitude
states
1
2

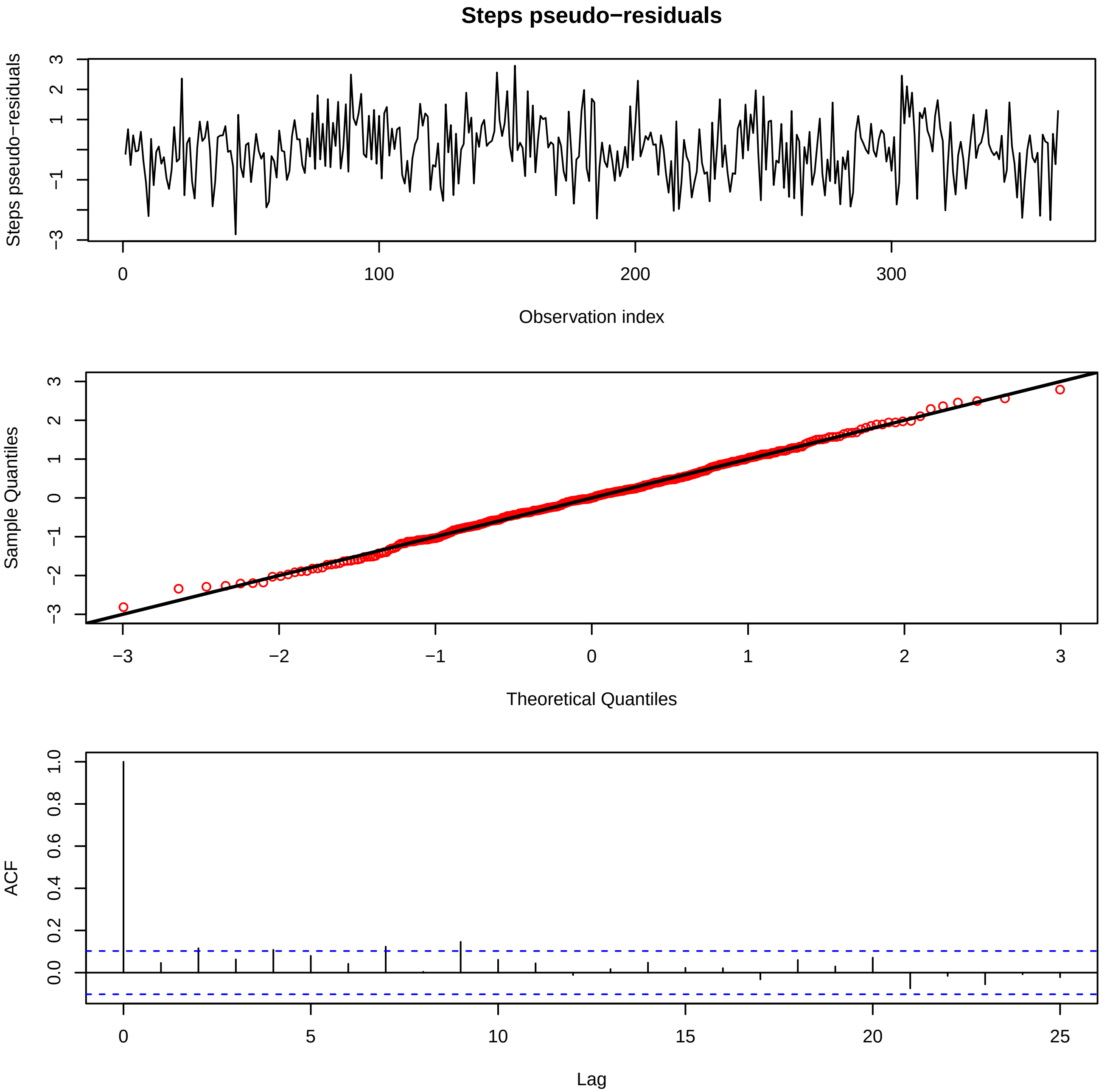

Steps pseudo−residuals
Steps pseudo−residuals
−3
−1
1
2
3
0
100
200
300
Observation index
Sample Quantiles
−3
−2
−1
0
1
2
3
−3
−2
−1
0
1
2
3
Theoretical Quantiles
ACF
0.0
0.2
0.4
0.6
0.8
1.0
0
5
10
15
20
25
Lag

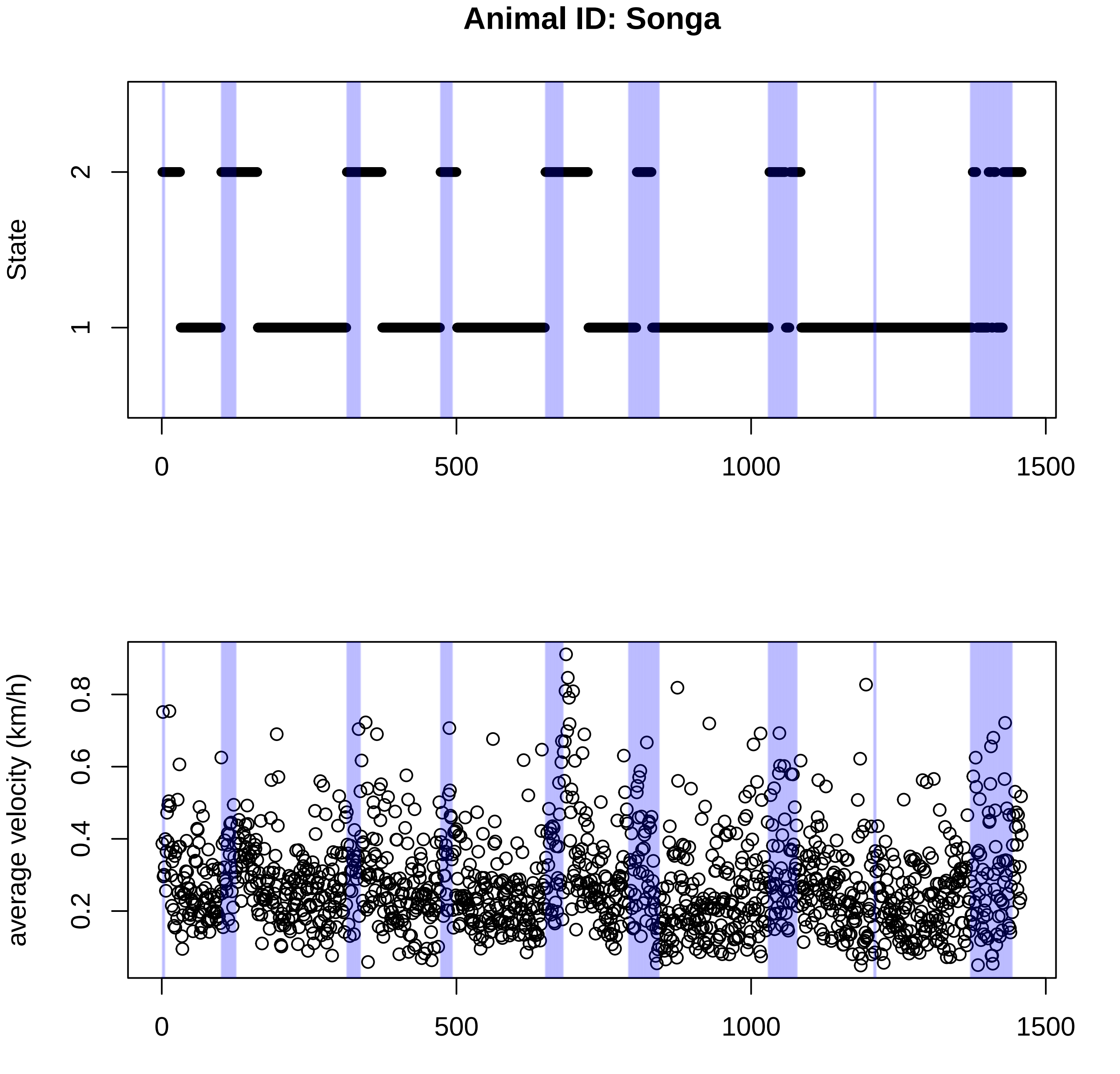
Animal ID: Songa
State
2
1
0
500
1000
1500
average velocity (km/h)
0.2
0.4
0.6
0.8
0
500
1000
1500

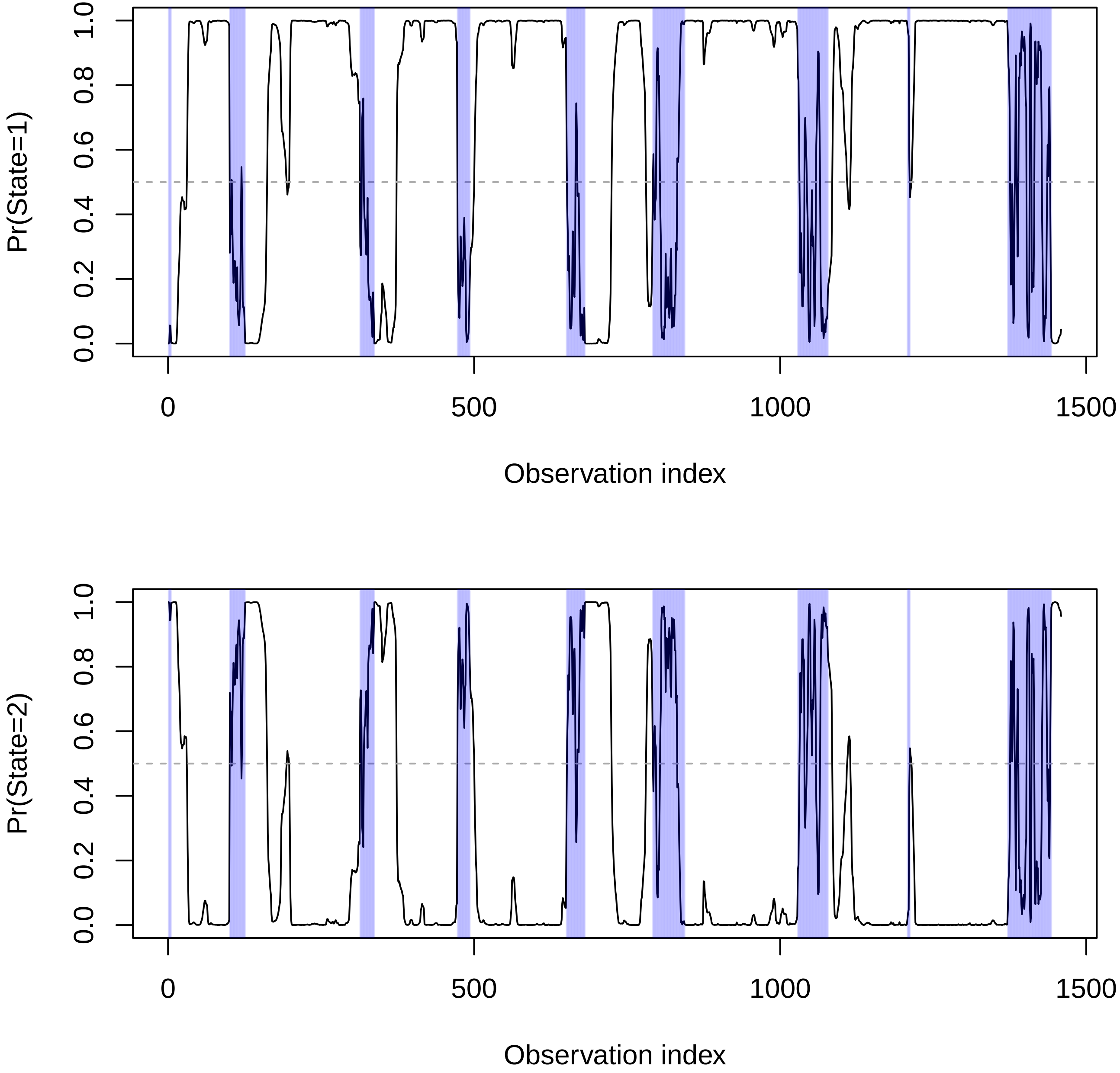
Pr(State=1)
0.0
0.2
0.4
0.6
0.8
1.0
0
500
1000
1500
Observation index
Pr(State=2)
0.0
0.2
0.4
0.6
0.8
1.0
0
500
1000
1500
Observation index

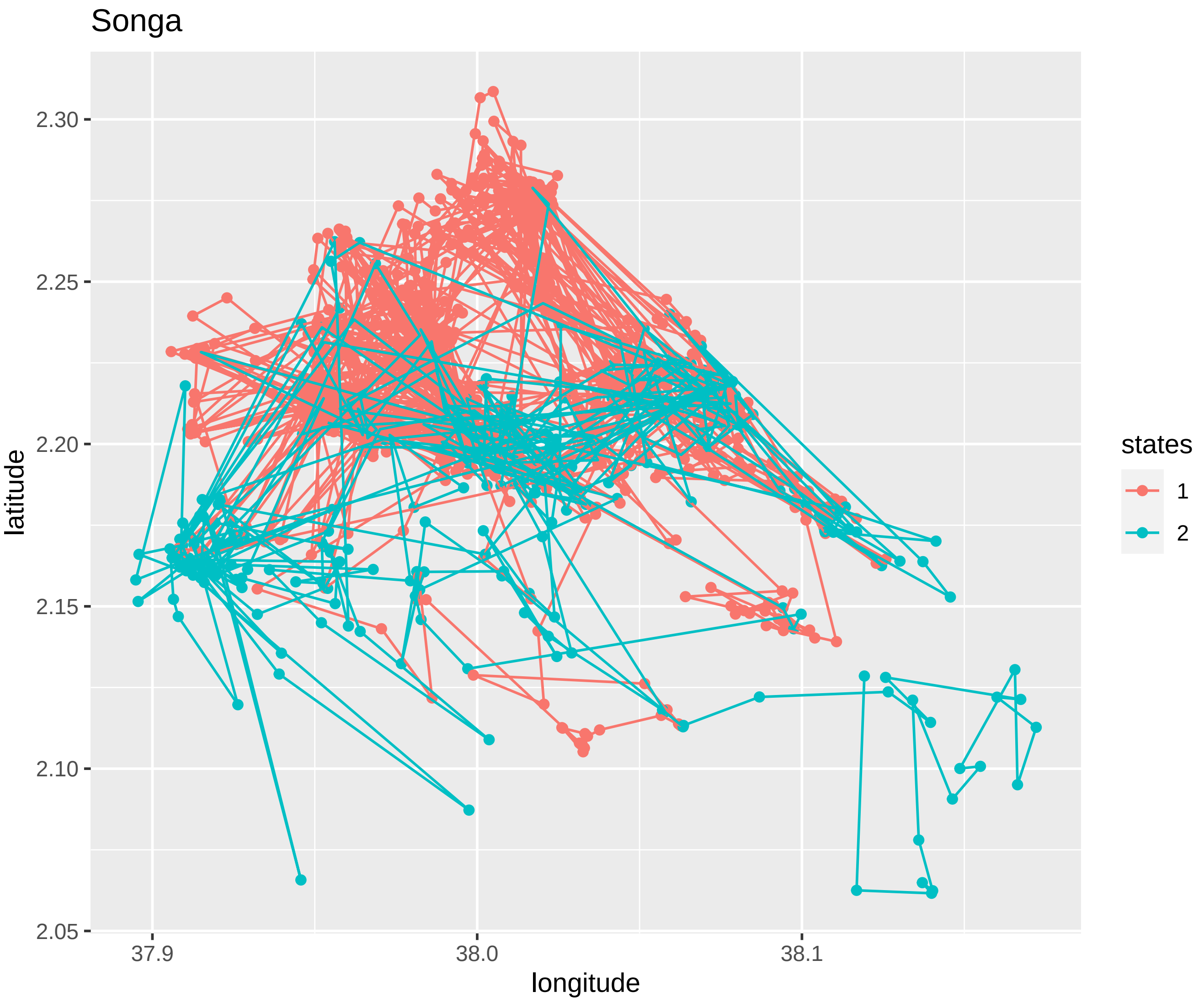
Songa
latitude
longitude
2.30
2.25
2.20
2.15
2.10
2.05
37.9
38.0
38.1
states
1
2

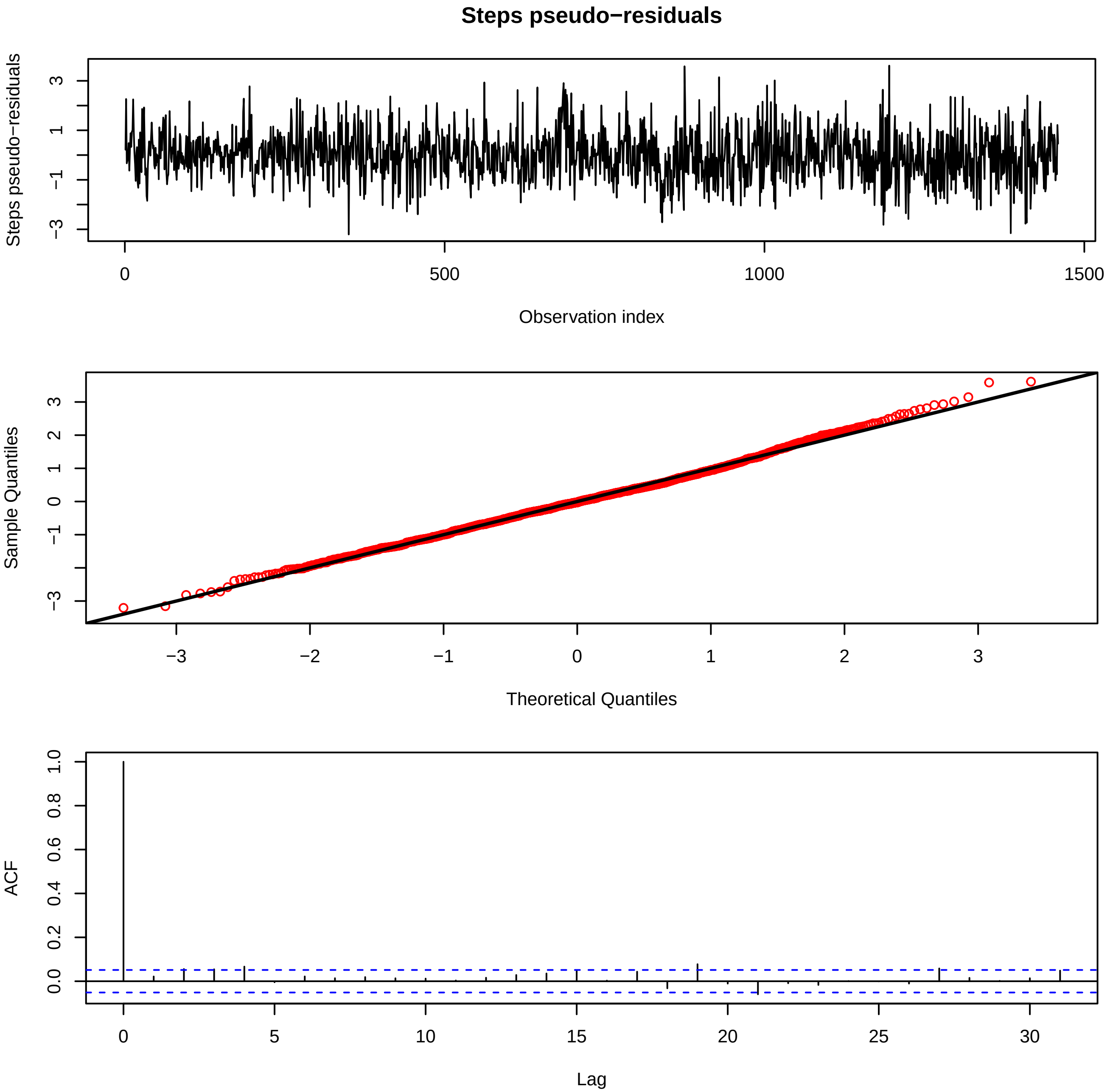
Steps pseudo−residuals
Steps pseudo−residuals
3
1
−1
−3
0
500
1000
1500
Observation index
Sample Quantiles
3
2
1
0
−1
−3
−3
−2
−1
0
1
2
3
Theoretical Quantiles
ACF
1.0
0.8
0.6
0.4
0.2
0.0
0
5
10
15
20
25
30
Lag

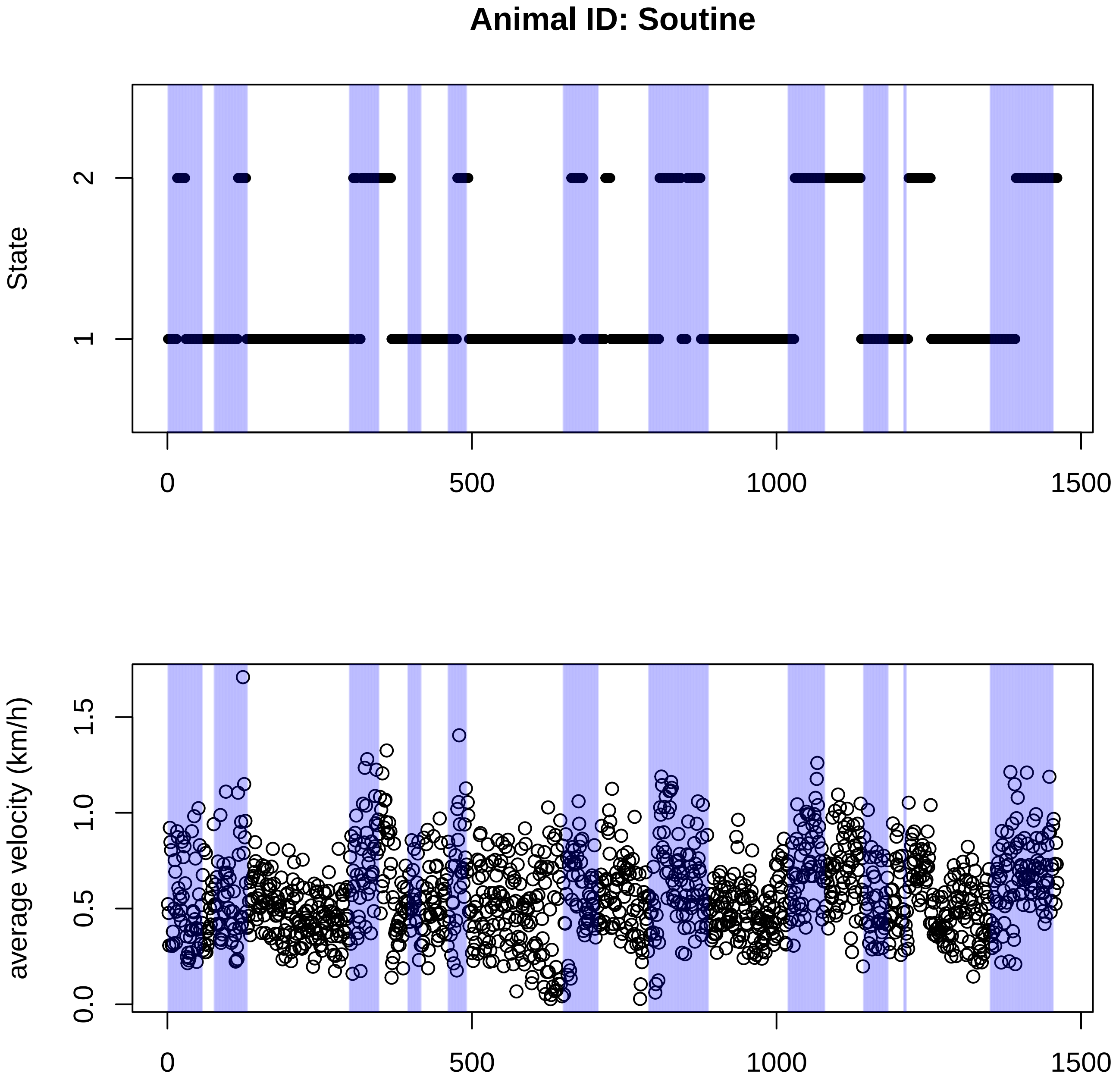
Animal ID: Soutine
State
2
1
0
500
1000
1500
average velocity (km/h)
0.0
0.5
1.0
1.5
0
500
1000
1500

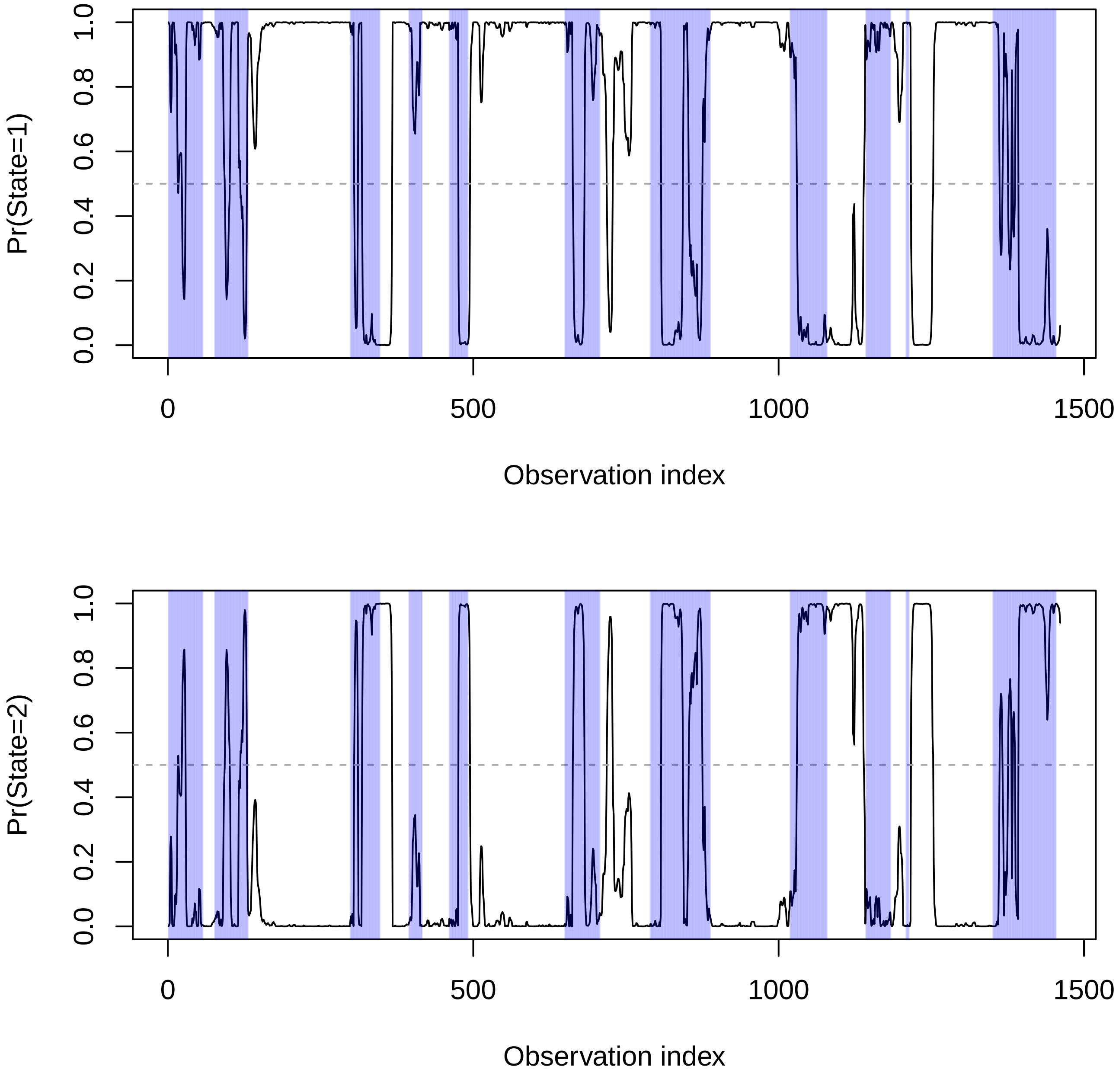
Pr(State=1)
1.0
0.8
0.6
0.4
0.2
0.0
0
500
1000
1500
Observation index
Pr(State=2)
1.0
0.8
0.6
0.4
0.2
0.0
0
500
1000
1500
Observation index

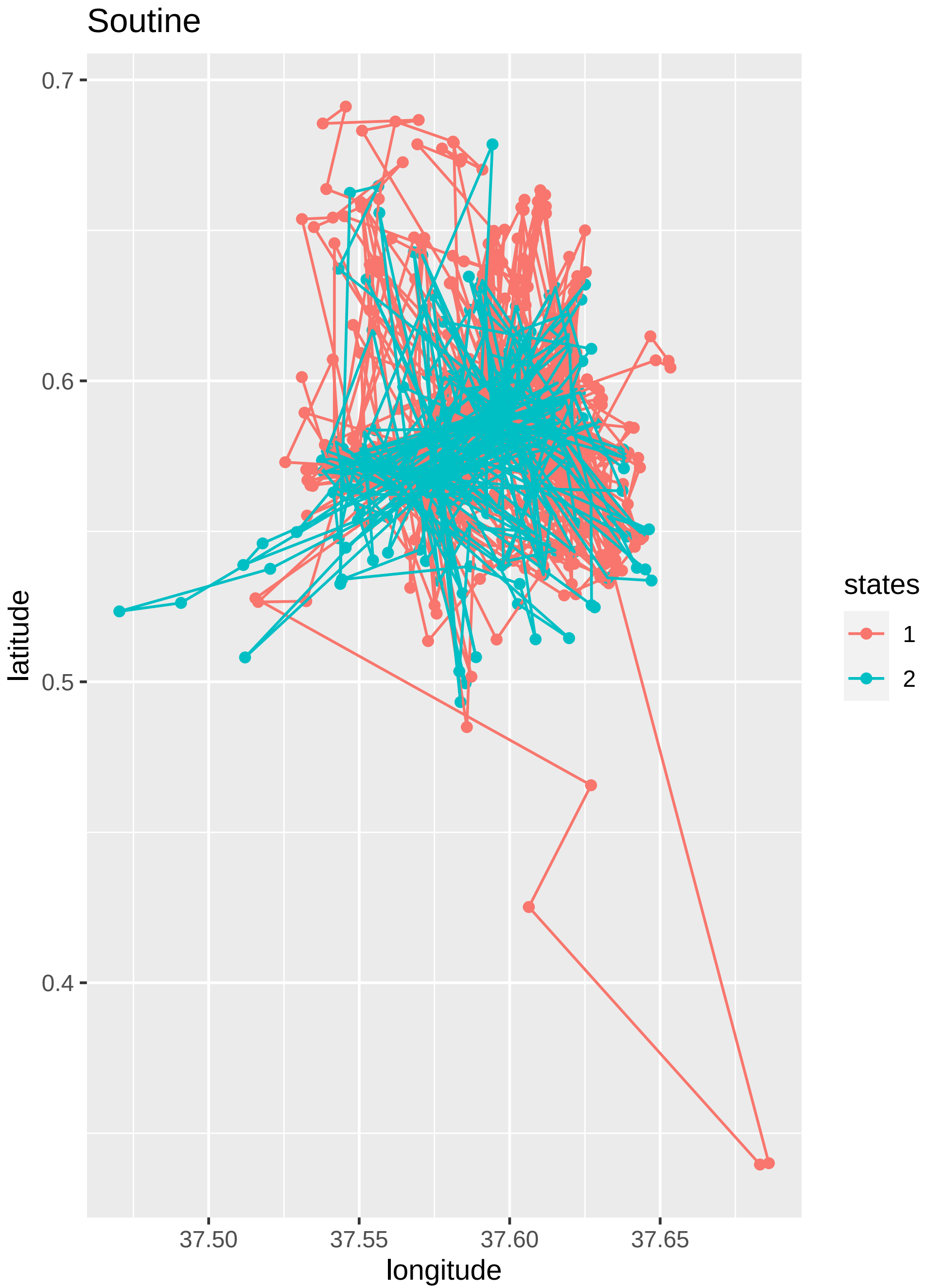
Soutine
0.7
0.6
0.5
0.4
37.50
37.55
37.60
37.65
longitude
latitude
states
1
2

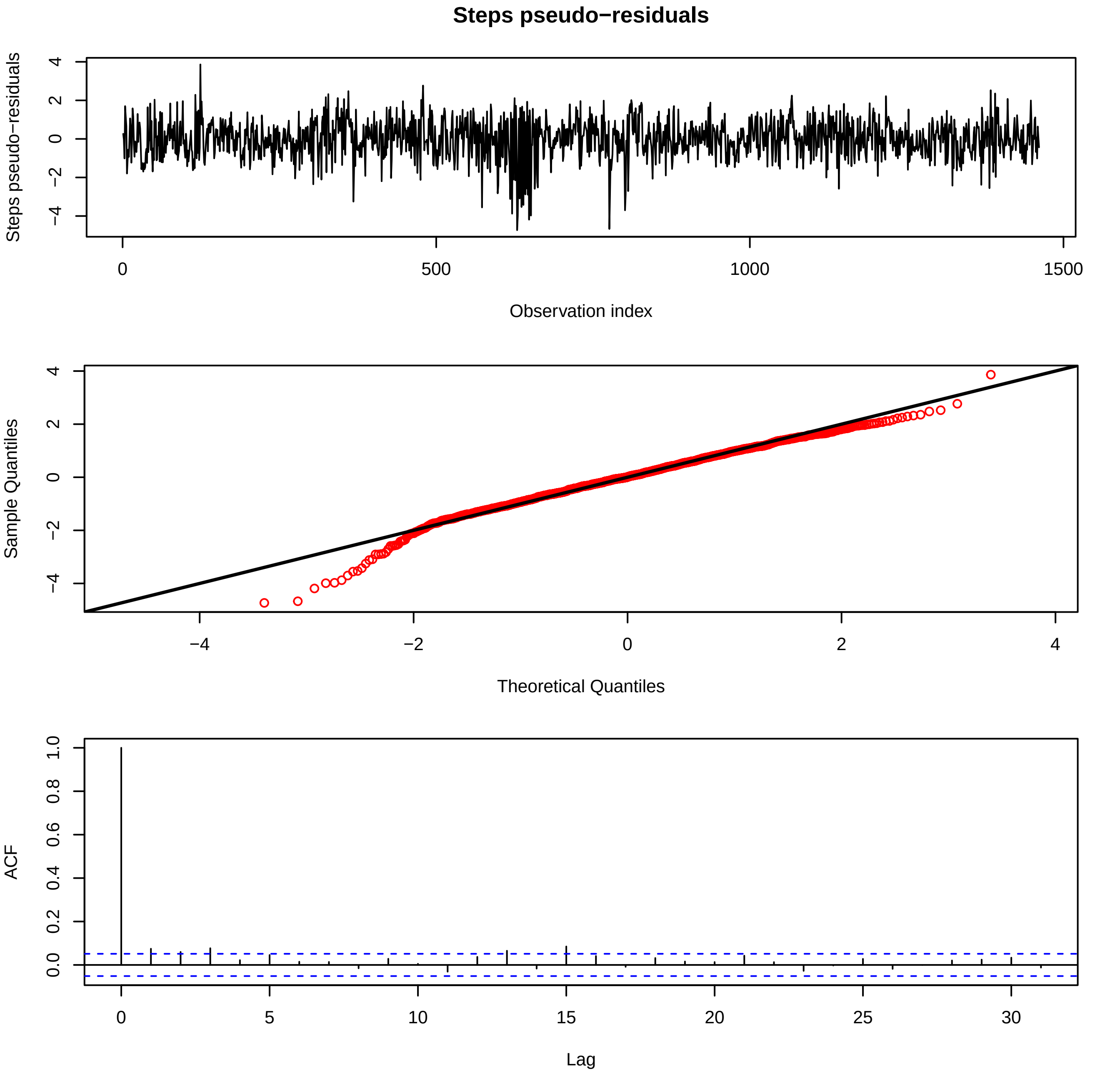

Steps pseudo−residuals
Steps pseudo−residuals
Observation index
Sample Quantiles
Theoretical Quantiles
ACF
Lag

Animal ID: Squall

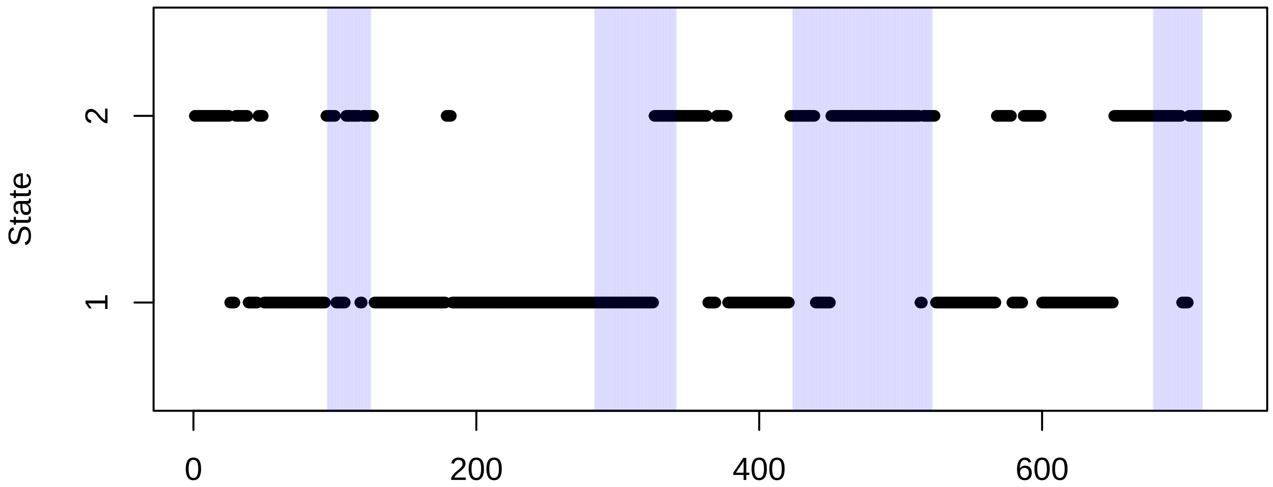

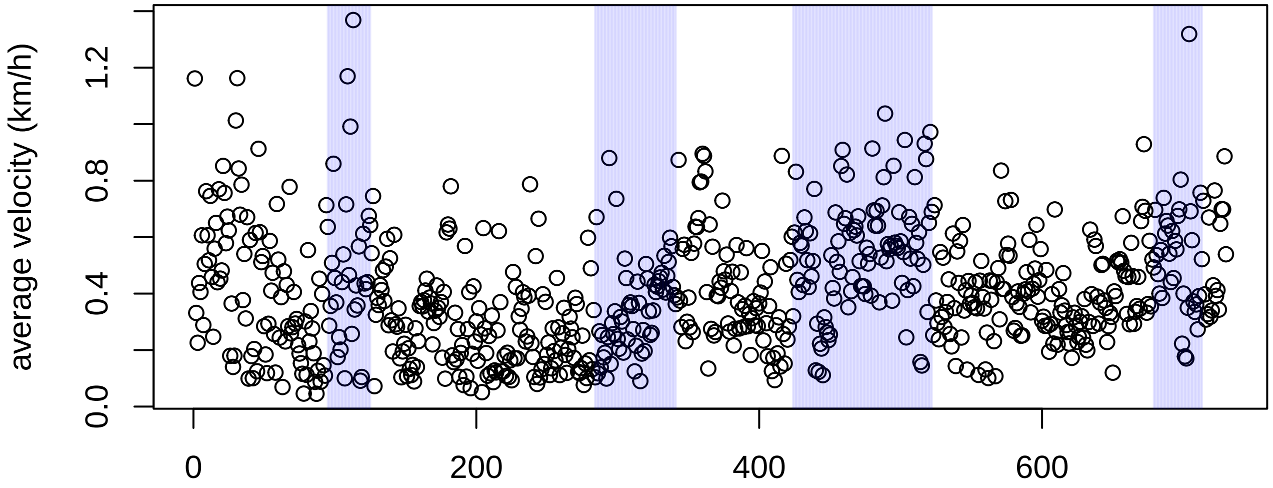

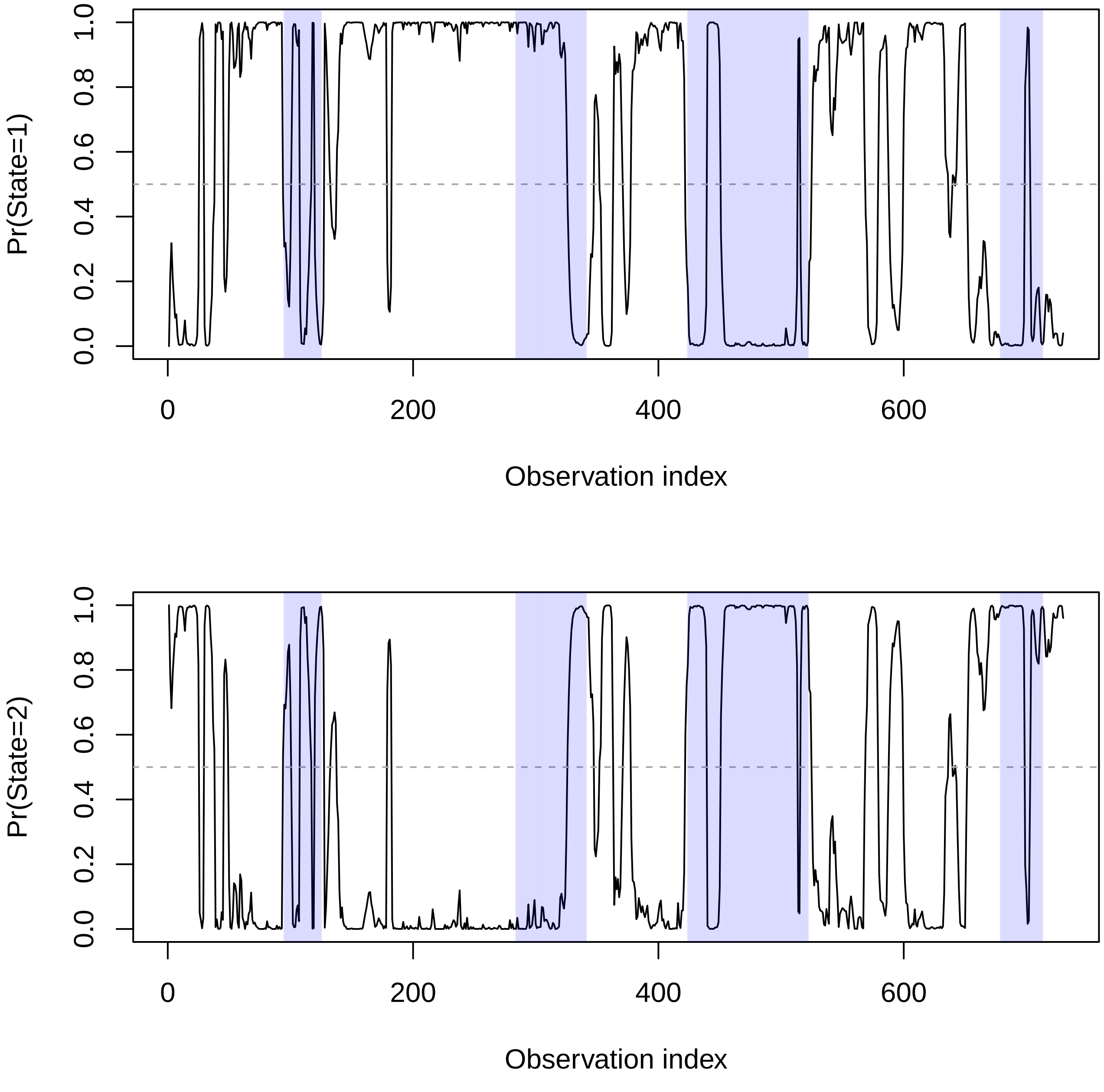

Pr(State=1)
0.0
0.2
0.4
0.6
0.8
1.0
0
200
400
600
Observation index
Pr(State=2)
0.0
0.2
0.4
0.6
0.8
1.0
0
200
400
600
Observation index

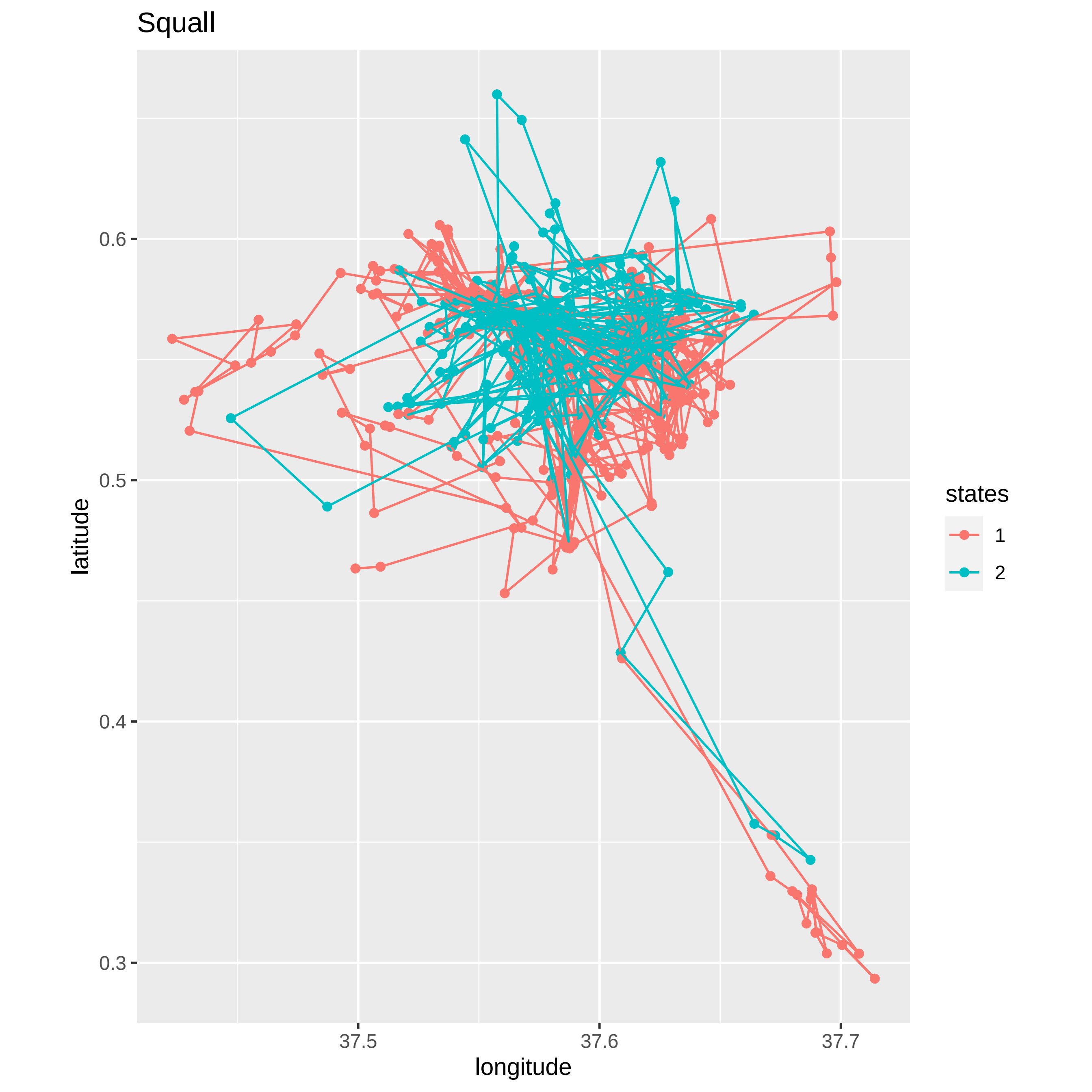
Squall
latitude
0.6
0.5
0.4
0.3
longitude
37.5
37.6
37.7
states
1
2

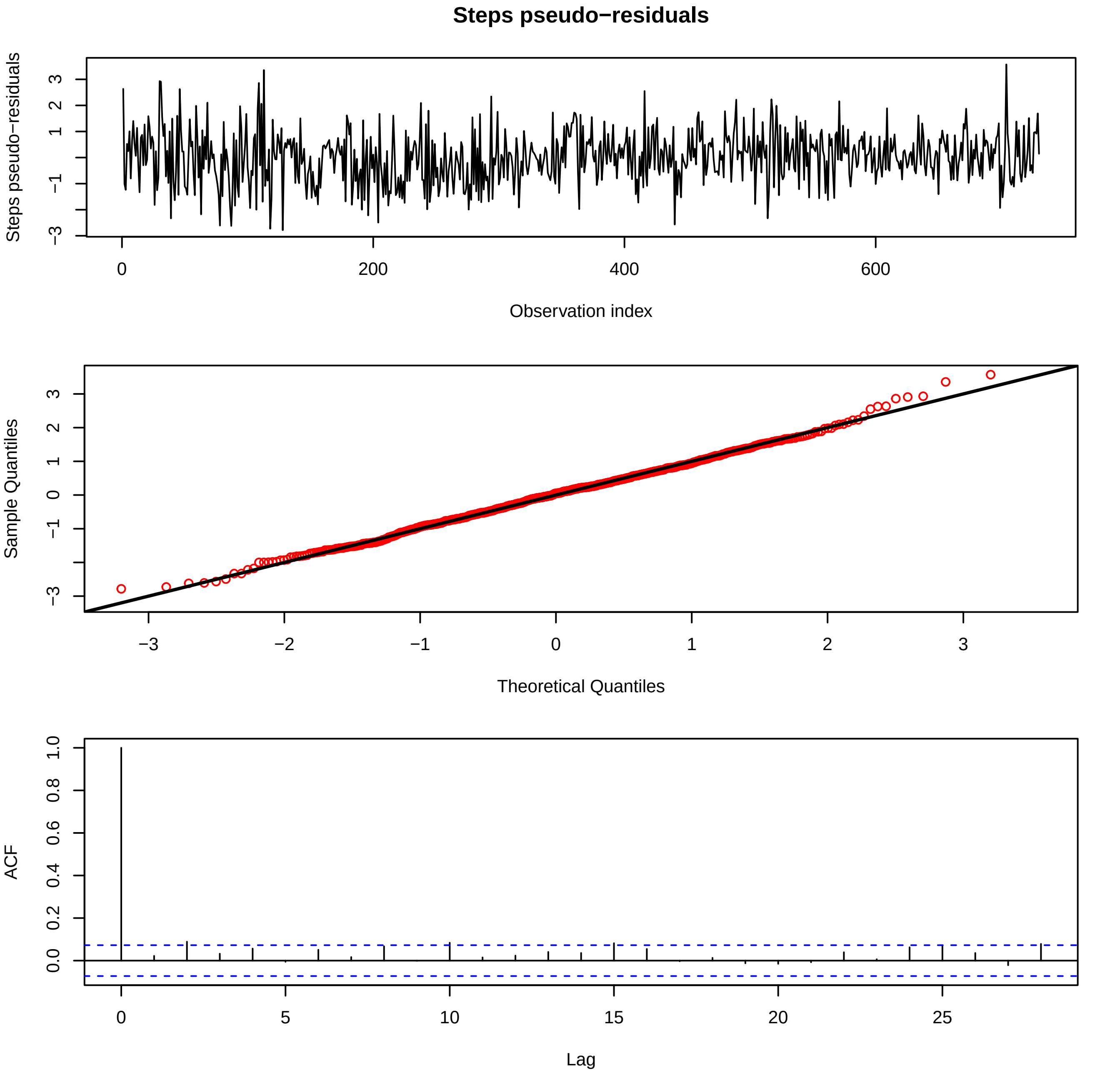

Steps pseudo−residuals
Steps pseudo−residuals
Observation index
Sample Quantiles
Theoretical Quantiles
ACF
Lag

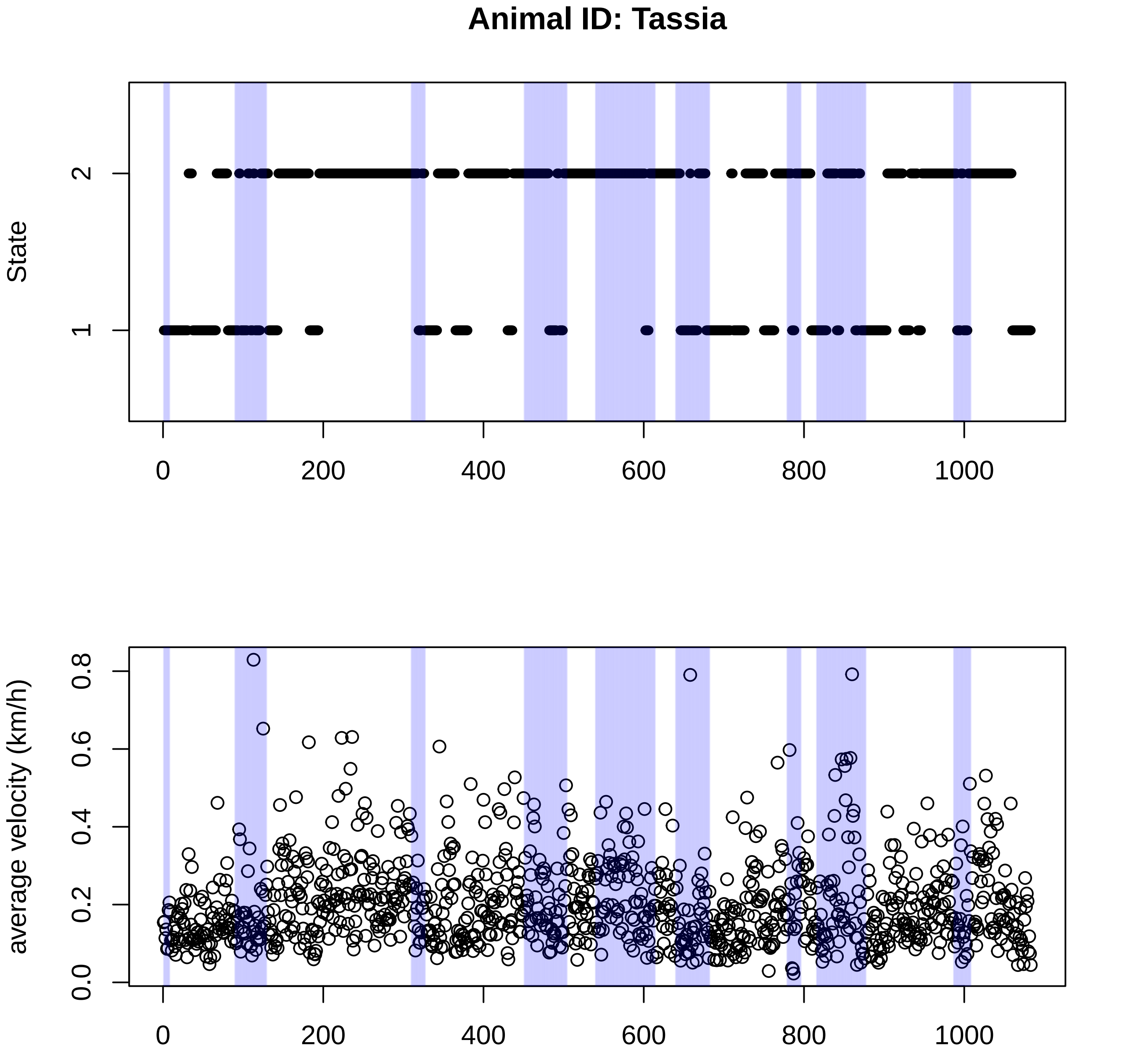

Animal ID: Tassia
State
2
1
0
200
400
600
800
1000
average velocity (km/h)
0.0
0.2
0.4
0.6
0.8
0
200
400
600
800
1000

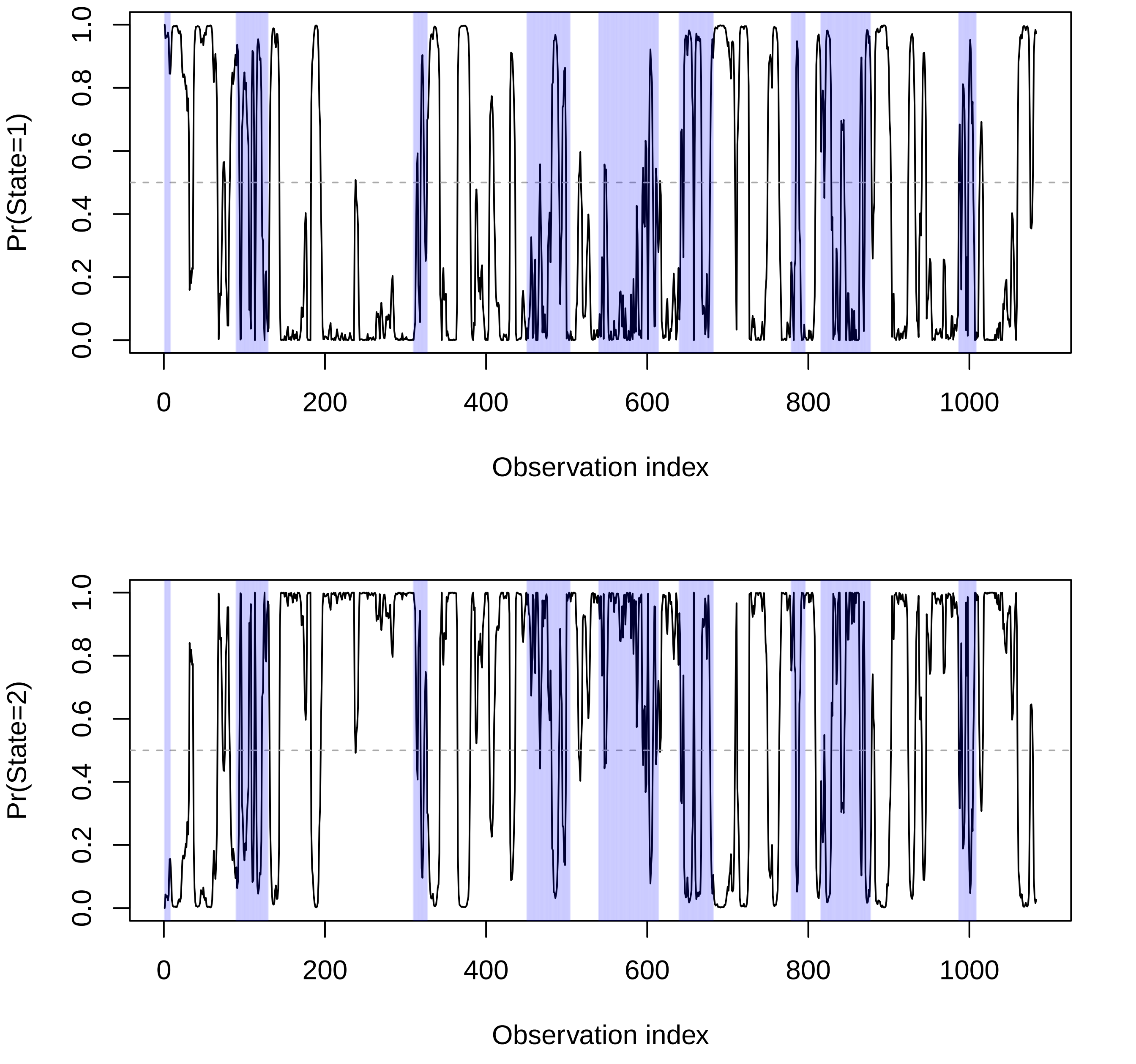

Pr(State=1)
Pr(State=2)
Observation index
0.0
0.2
0.4
0.6
0.8
1.0
0
200
400
600
800
1000

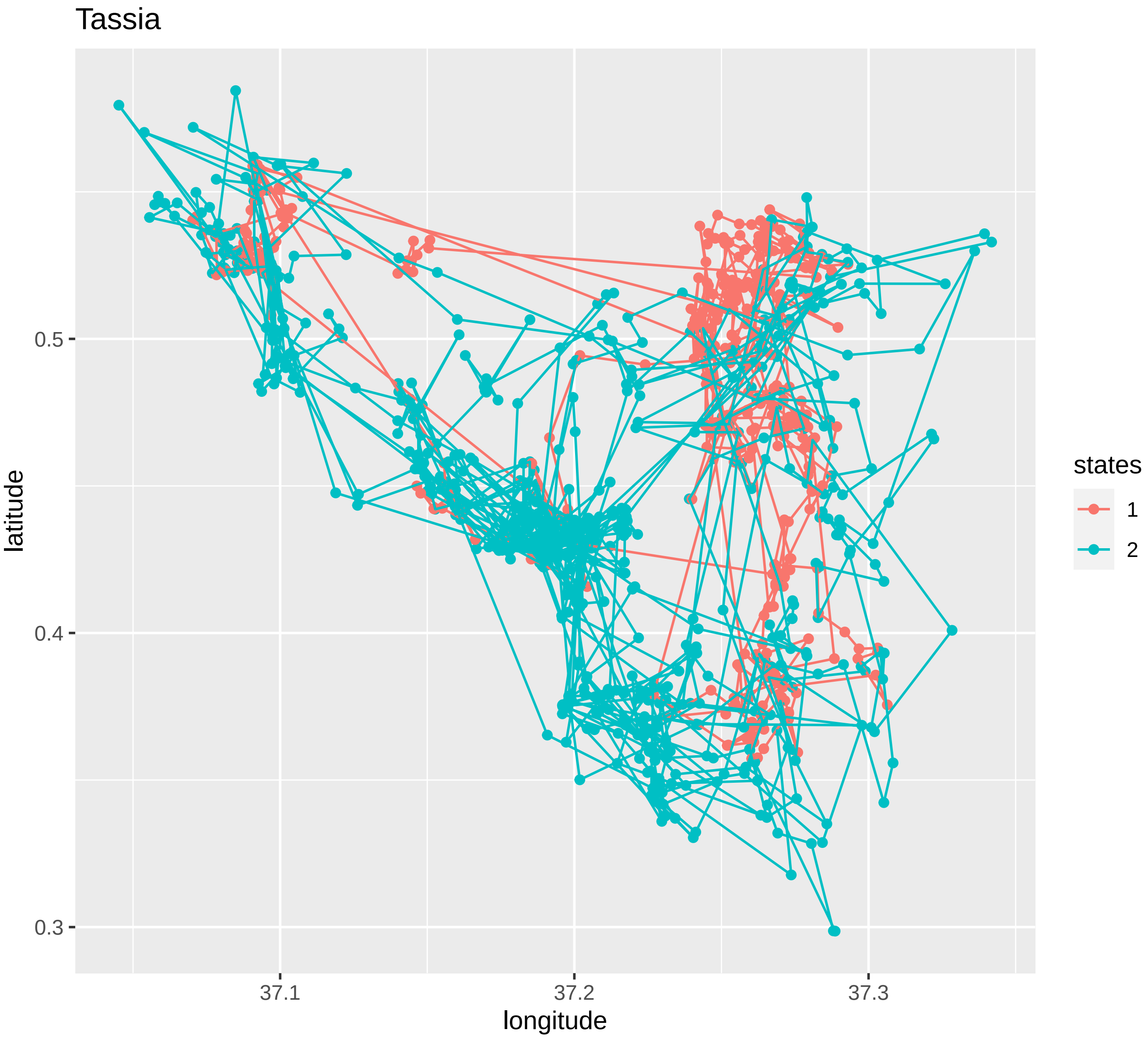

Tassia
latitude
longitude
0.5
0.4
0.3
37.1
37.2
37.3
states
1
2

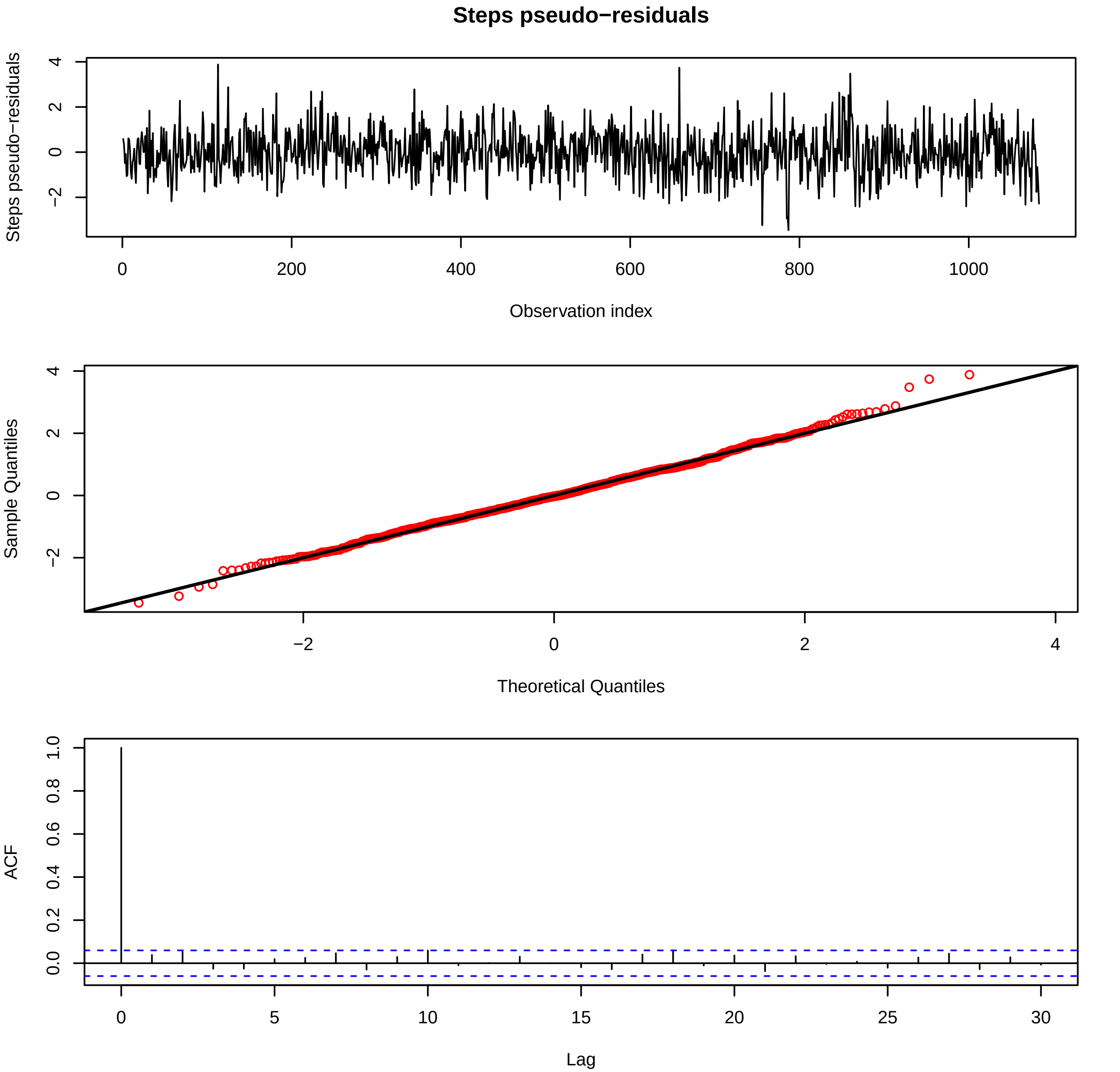
Steps pseudo−residuals
Steps pseudo−residuals
Observation index
0
200
400
600
800
1000
4
2
0
−2
Sample Quantiles
Theoretical Quantiles
−2
0
2
4
ACF
Lag
1.0
0.8
0.6
0.4
0.2
0.0
0
5
10
15
20
25
30

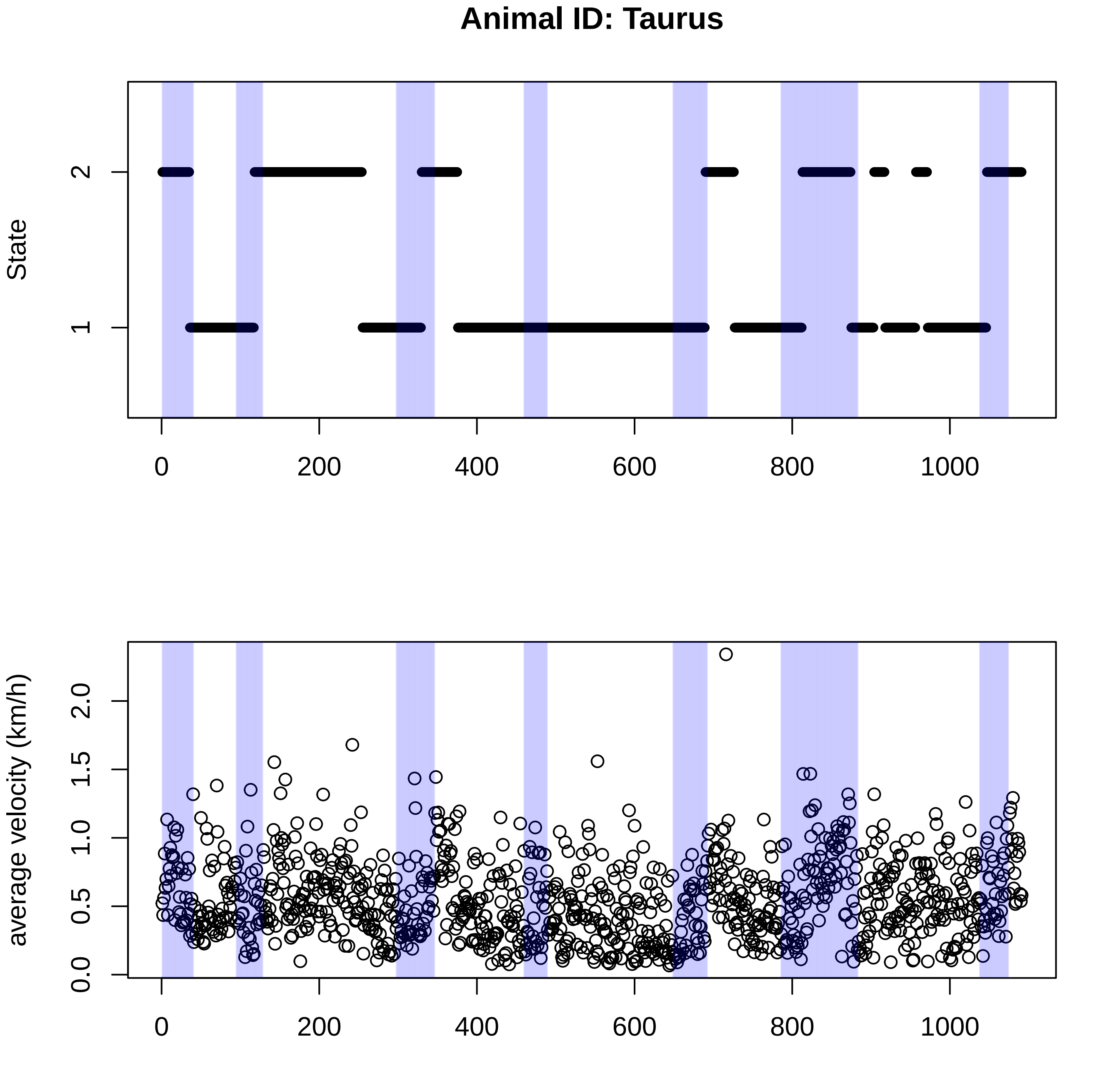

Animal ID: Taurus
State
2
1
0
200
400
600
800
1000
average velocity (km/h)
0.0
0.5
1.0
1.5
2.0

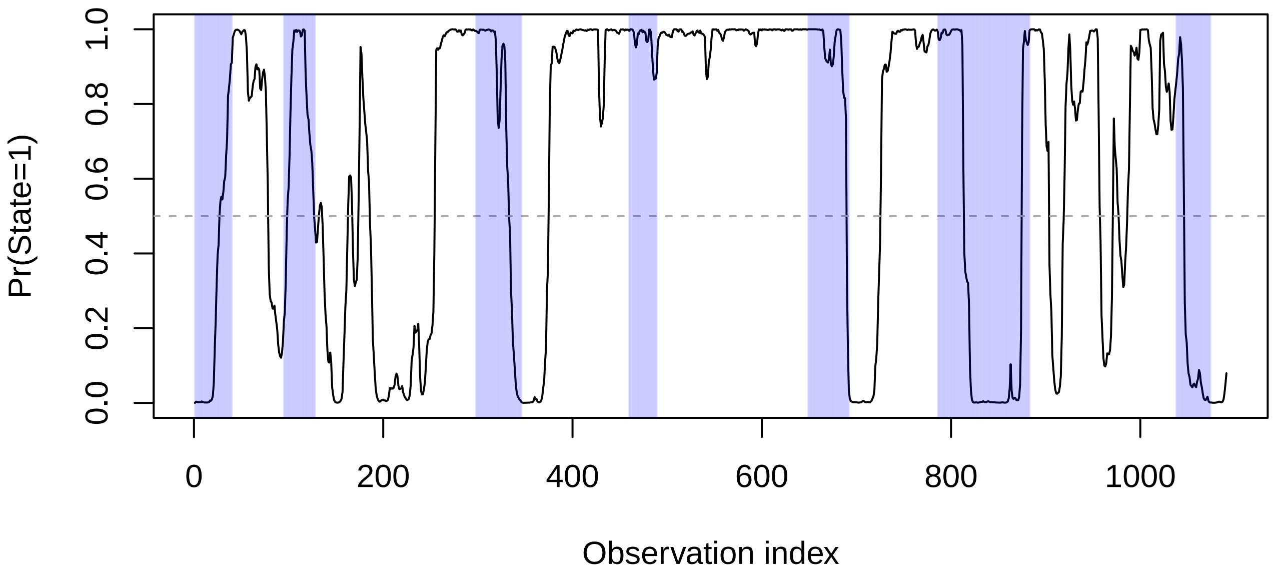

Pr(State=1)
0.0
0.2
0.4
0.6
0.8
1.0
0
200
400
600
800
1000
Observation index

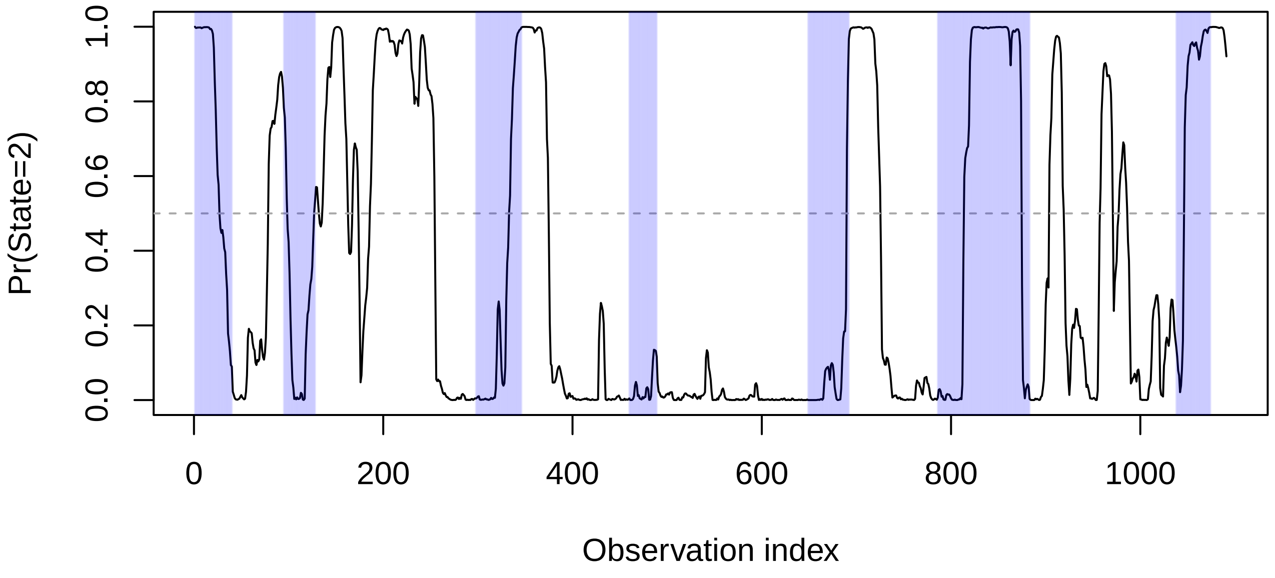

Pr(State=2)
0.0
0.2
0.4
0.6
0.8
1.0
0
200
400
600
800
1000
Observation index

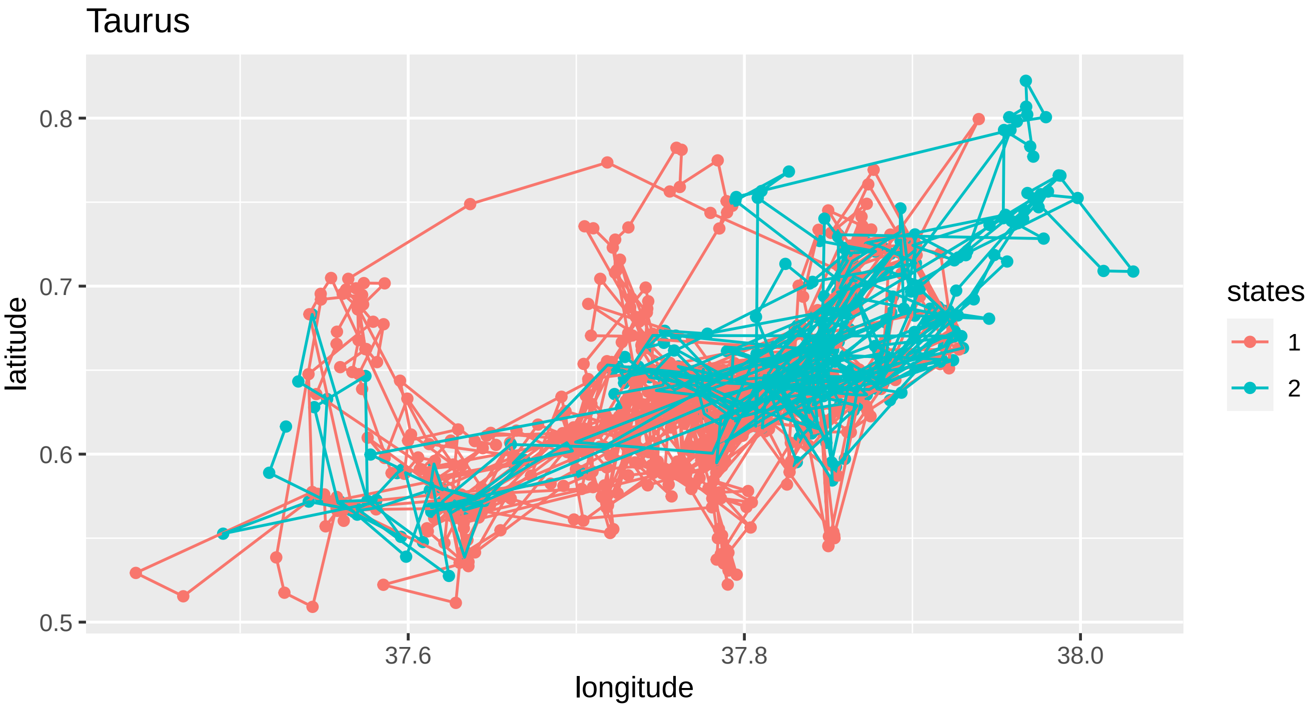

Taurus
0.8
0.7
0.6
0.5
latitude
37.6
37.8
38.0
longitude
states
1
2

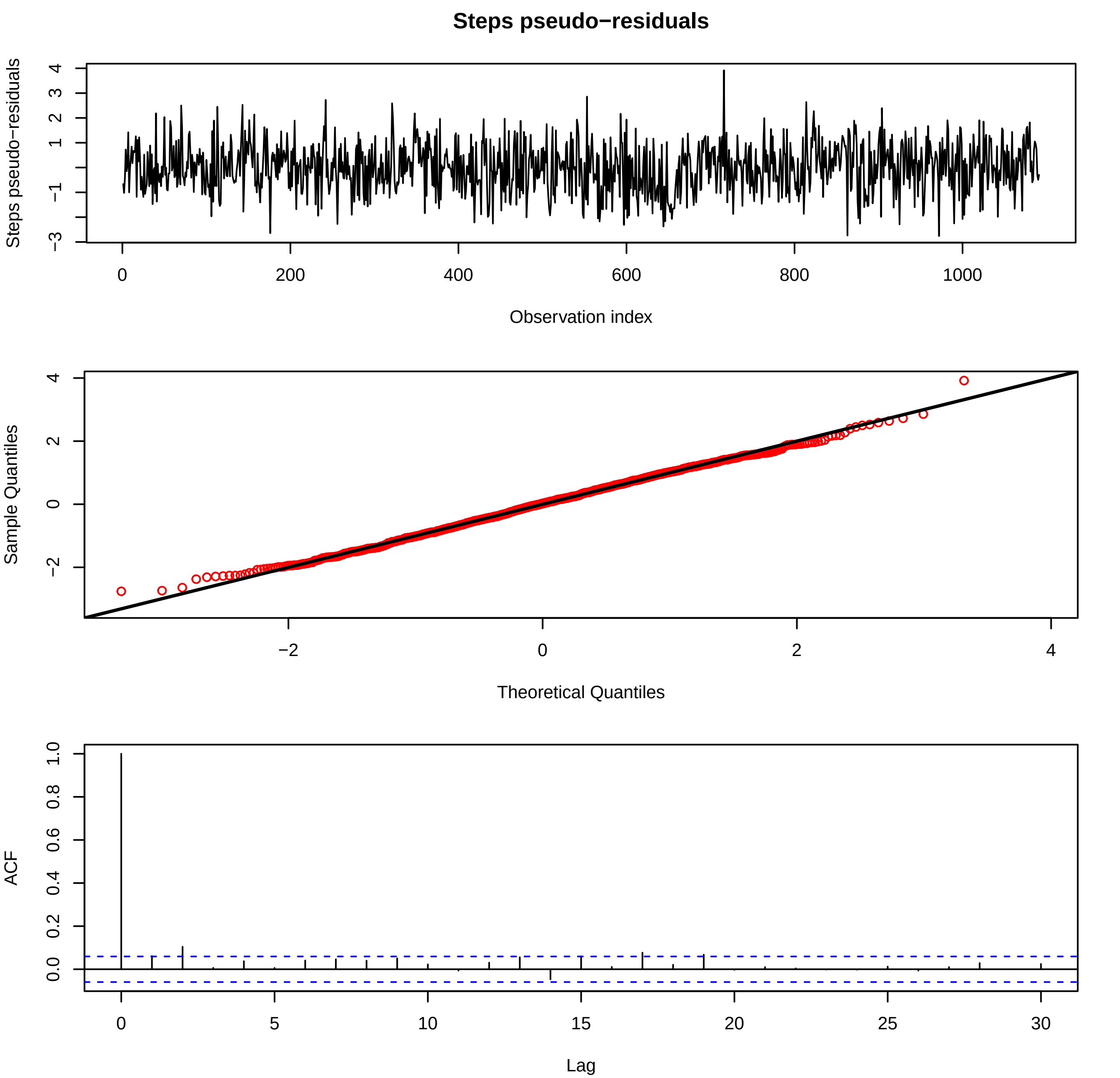
Steps pseudo−residuals
Steps pseudo−residuals
−3
−1
1
2
3
4
0
200
400
600
800
1000
Observation index
Sample Quantiles
−2
0
2
4
Theoretical Quantiles
ACF
0.0
0.2
0.4
0.6
0.8
1.0
0
5
10
15
20
25
30
Lag

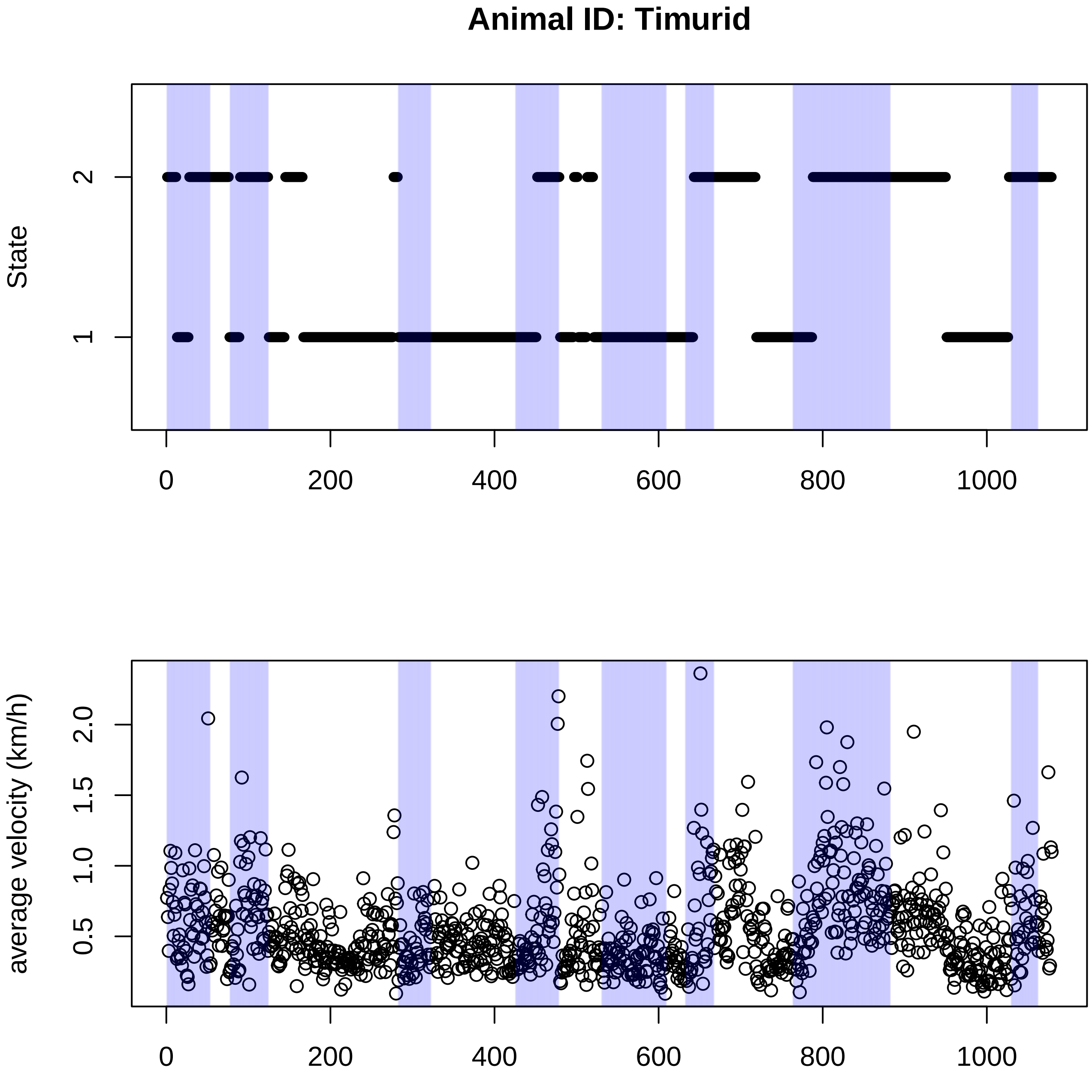

Animal ID: Timurid
State
2
1
0
200
400
600
800
1000
average velocity (km/h)
0.5
1.0
1.5
2.0

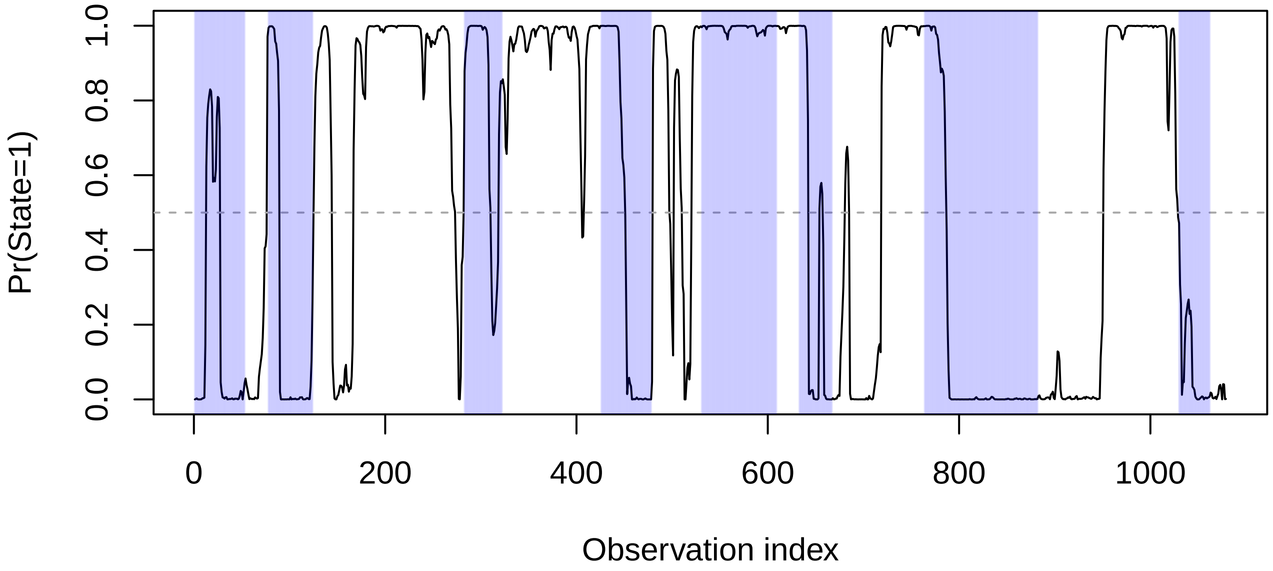

Pr(State=1)
0.0
0.2
0.4
0.6
0.8
1.0
0
200
400
600
800
1000
Observation index

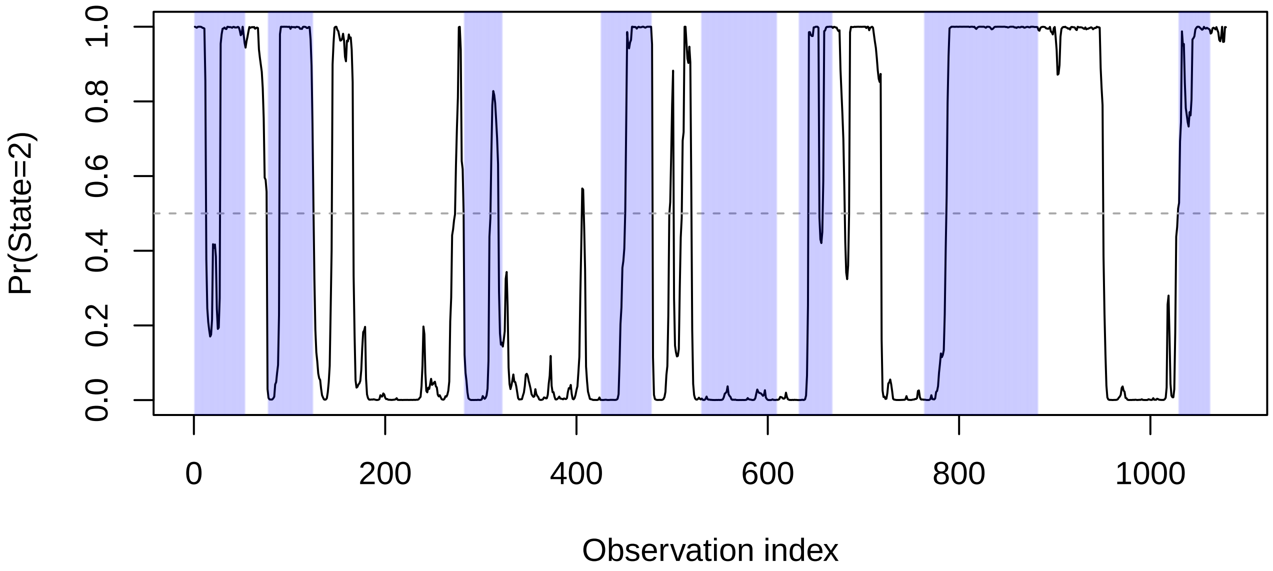

Pr(State=2)
0.0
0.2
0.4
0.6
0.8
1.0
0
200
400
600
800
1000
Observation index

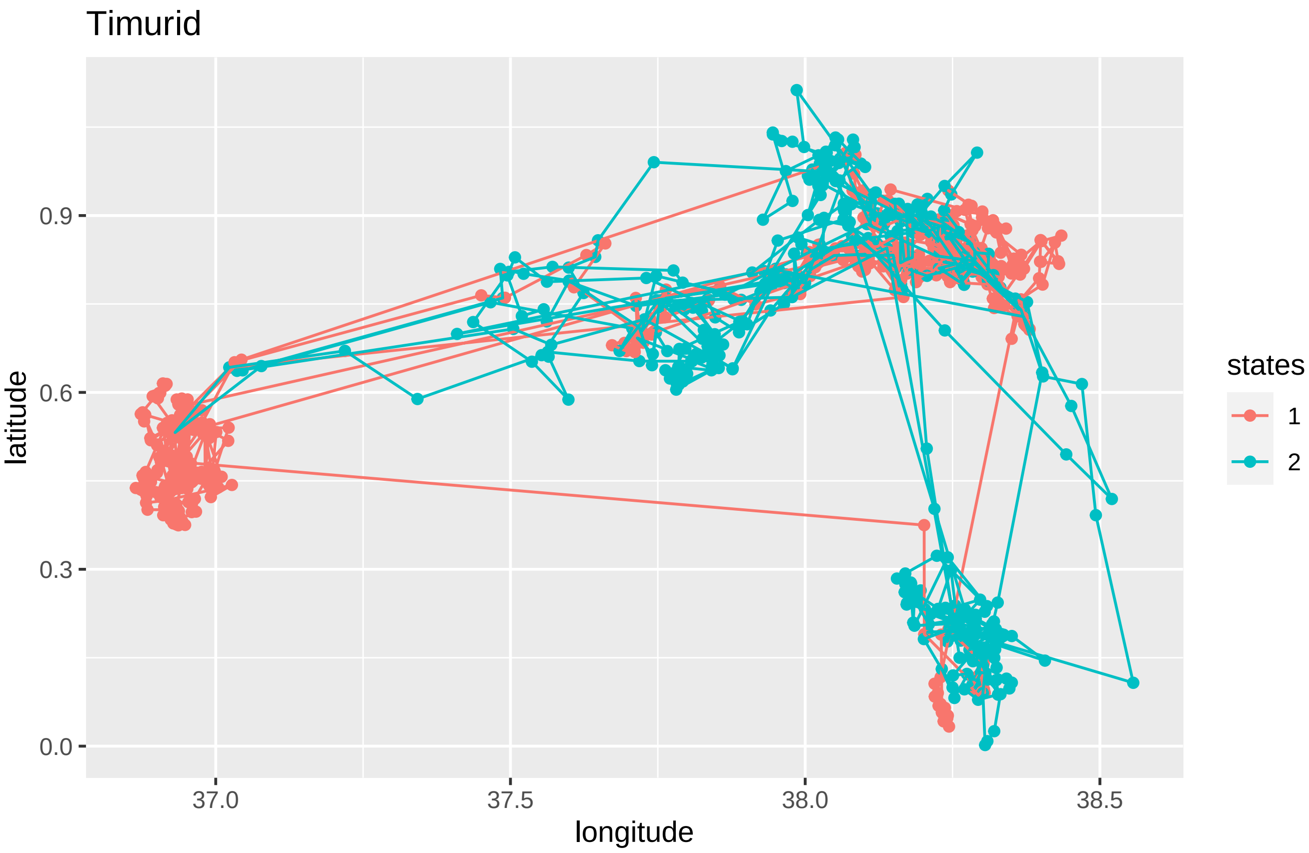
Timurid
latitude
longitude
0.0
0.3
0.6
0.9
37.0
37.5
38.0
38.5
states
1
2

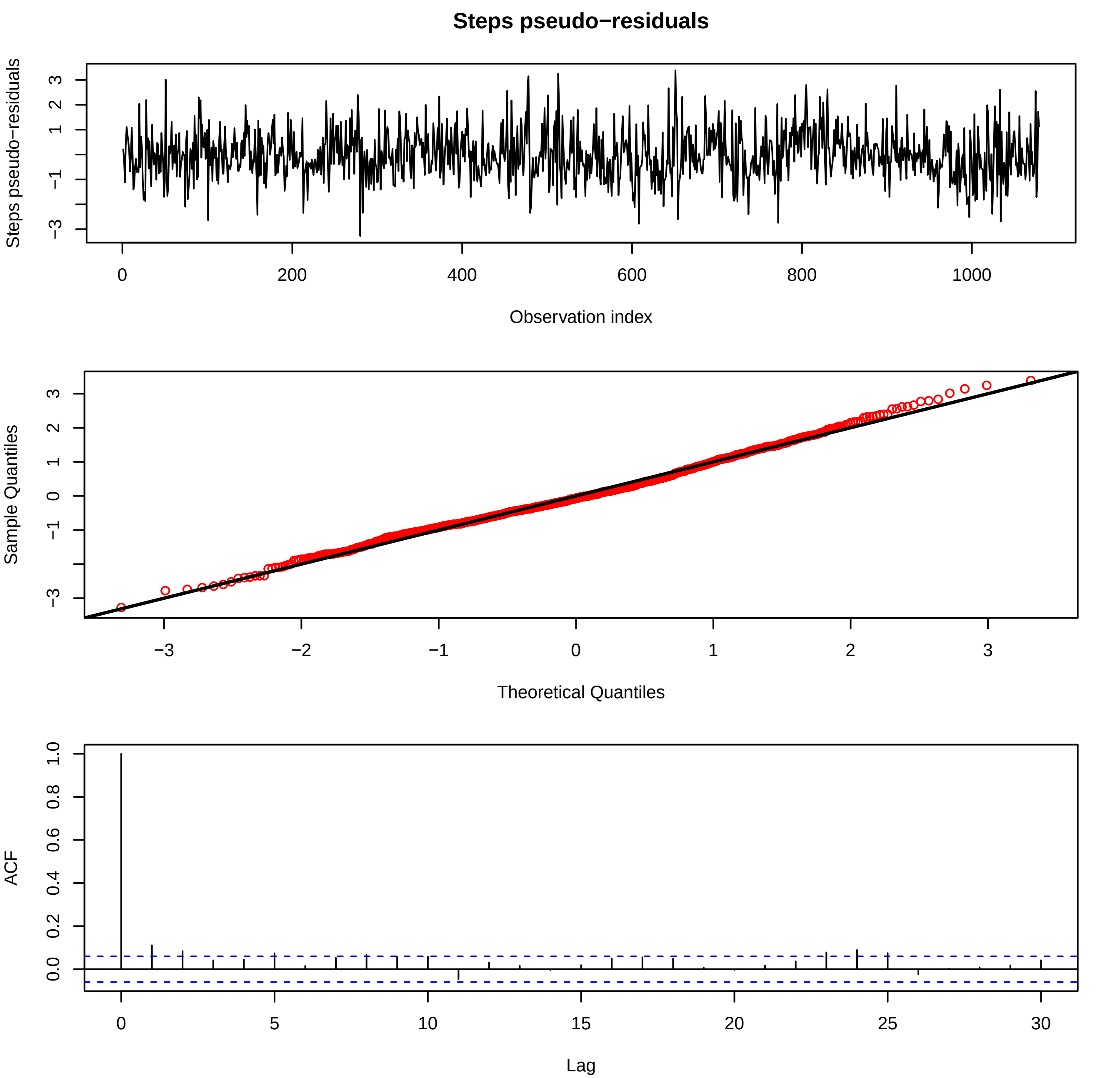

Steps pseudo−residuals
Steps pseudo−residuals
Observation index
Sample Quantiles
Theoretical Quantiles
ACF
Lag

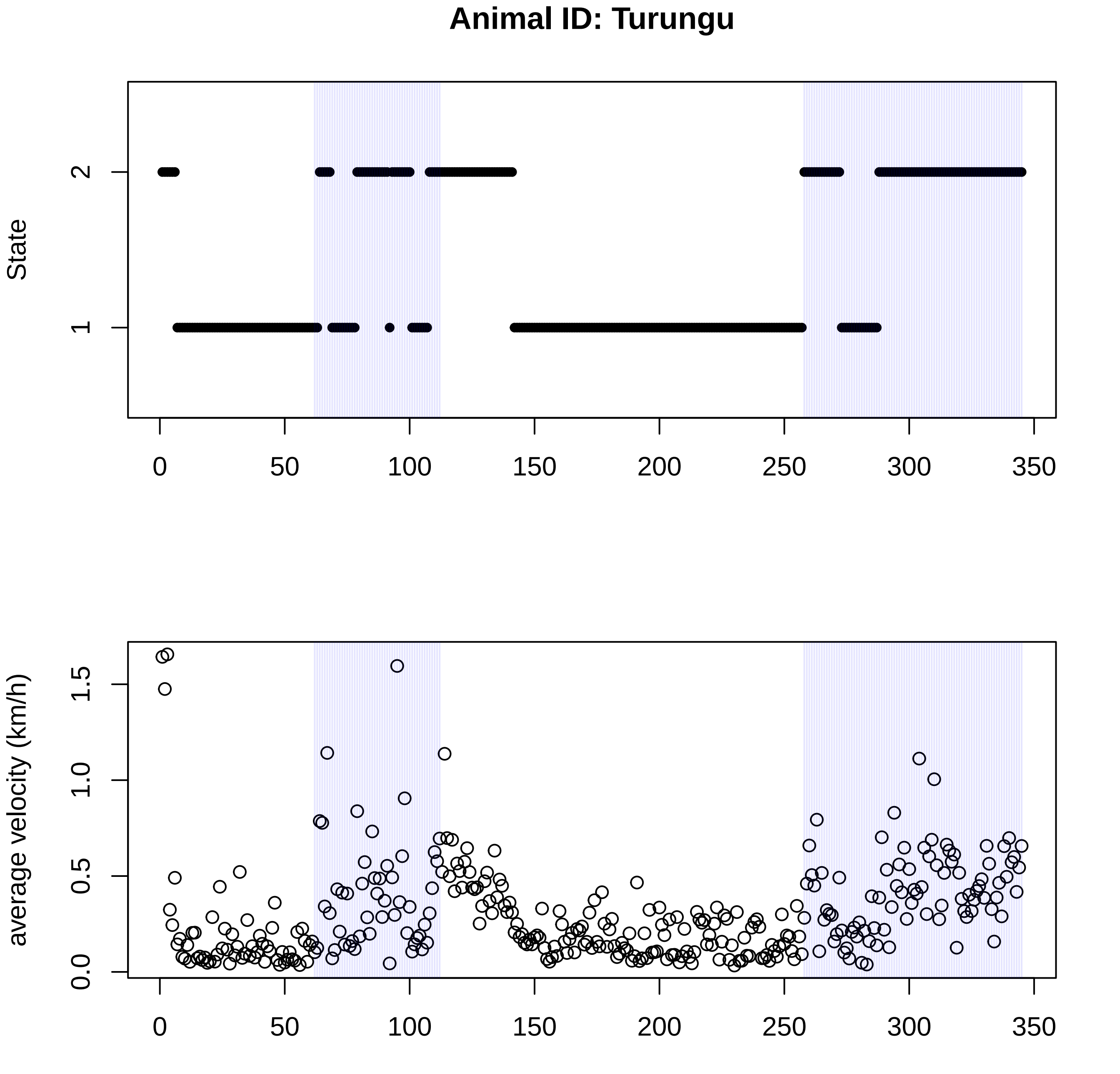

Animal ID: Turungu
State
2
1
0
50
100
150
200
250
300
350
average velocity (km/h)
0.0
0.5
1.0
1.5

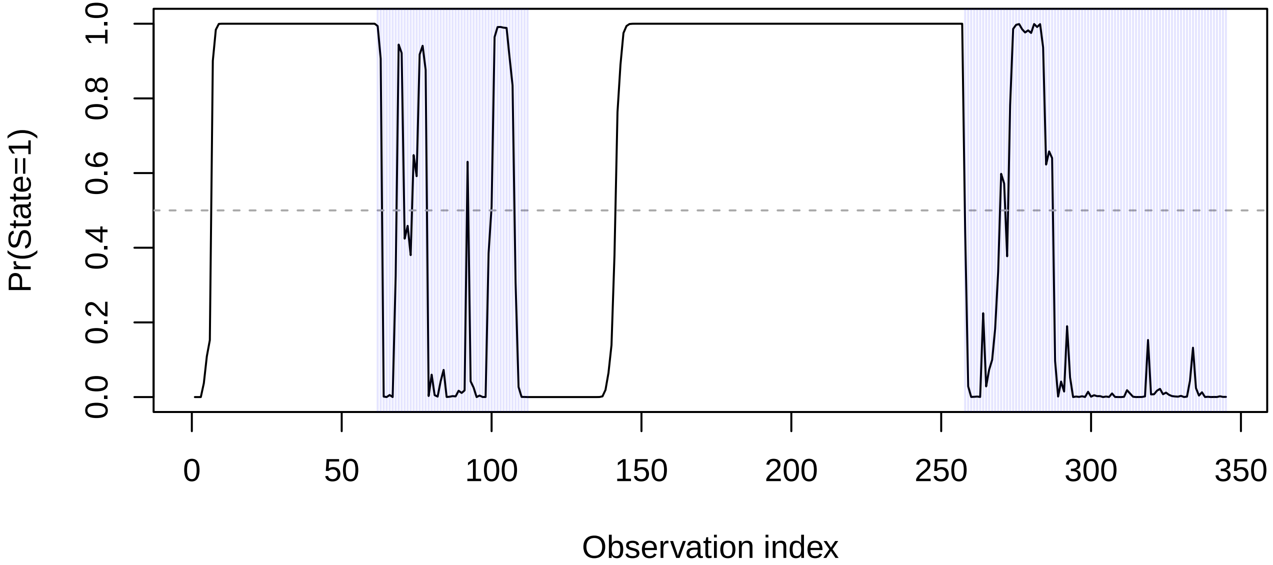
Pr(State=1)
1.0
0.8
0.6
0.4
0.2
0.0
0
50
100
150
200
250
300
350
Observation index

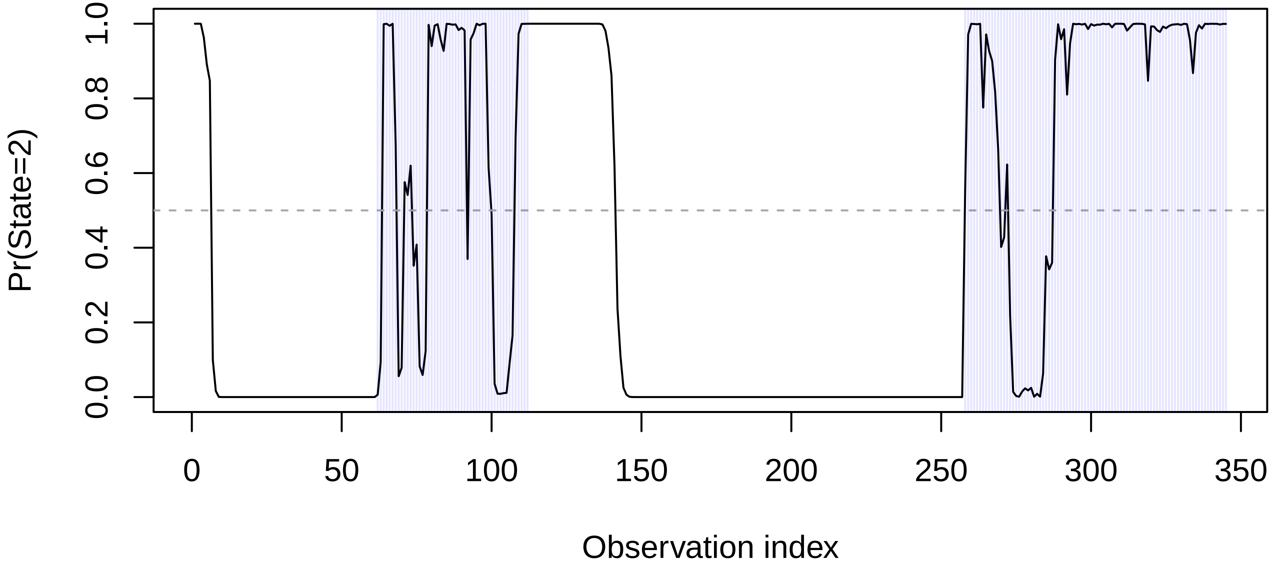
Pr(State=2)
1.0
0.8
0.6
0.4
0.2
0.0
0
50
100
150
200
250
300
350
Observation index

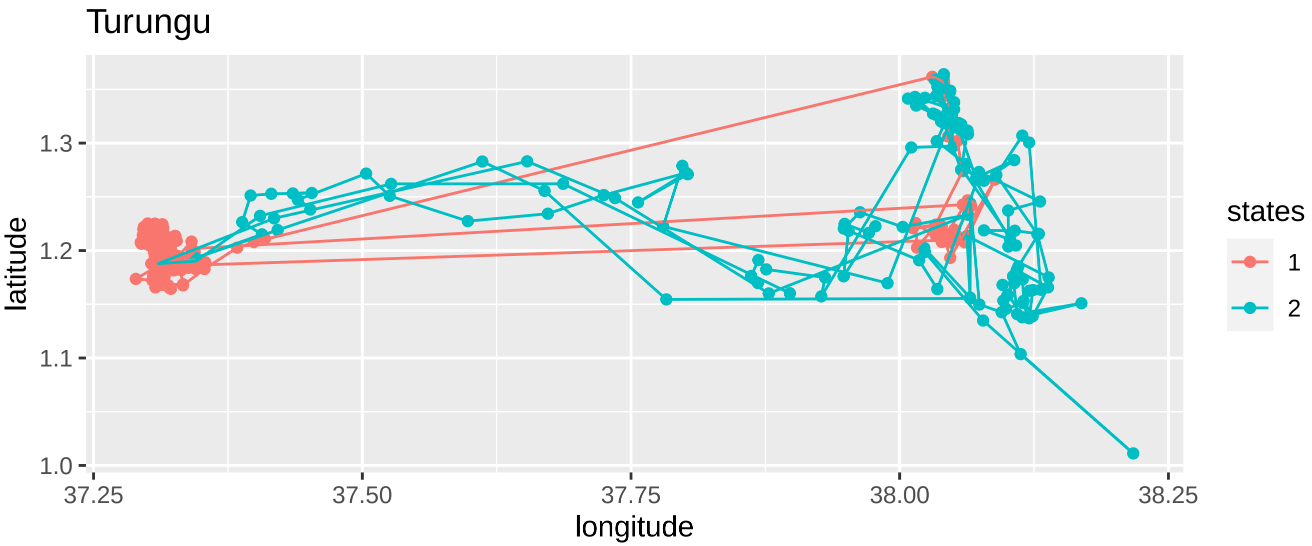

Turungu
latitude
1.3
1.2
1.1
1.0
37.25
37.50
37.75
38.00
38.25
longitude
states
1
2

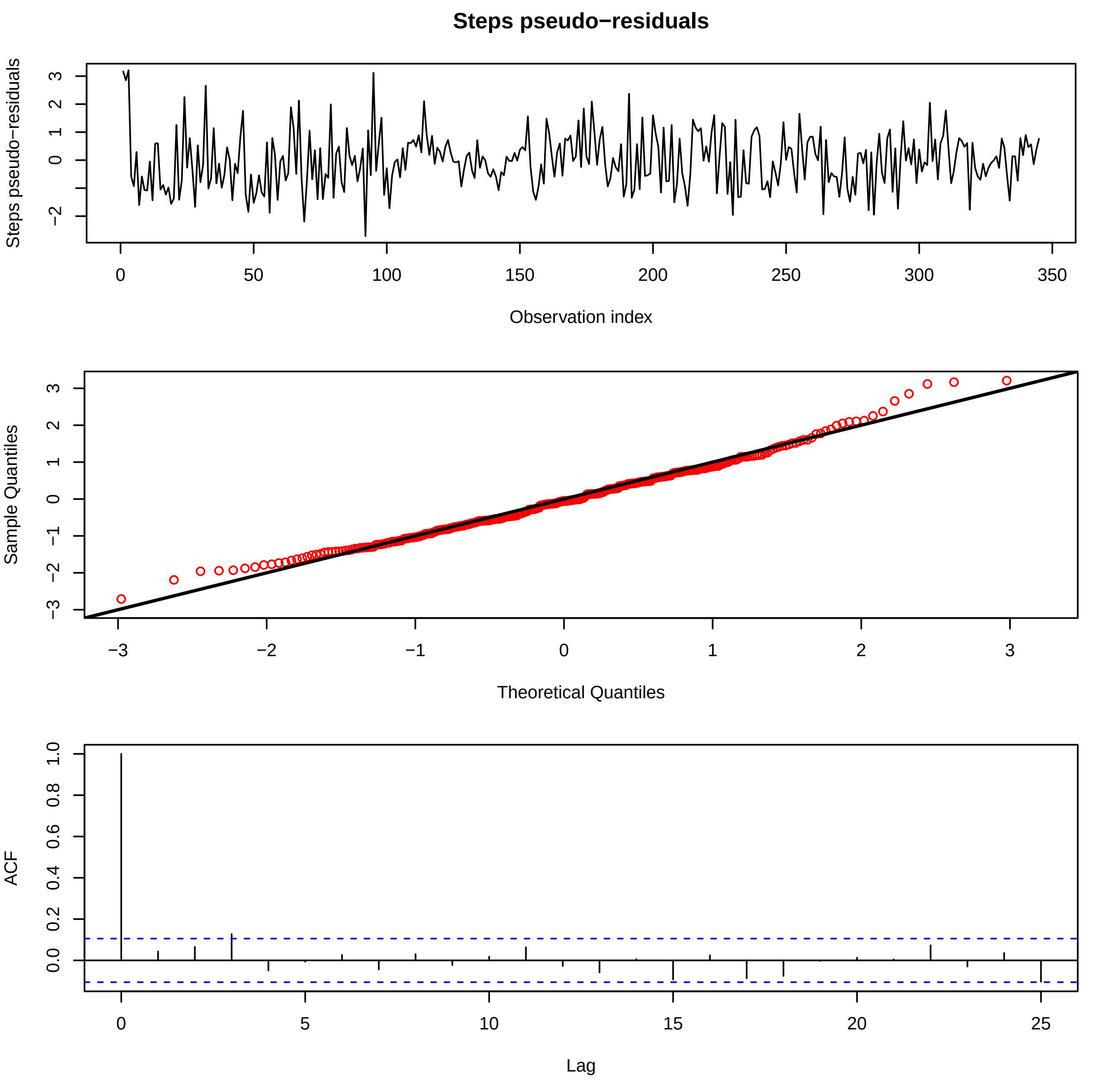
Steps pseudo−residuals
Steps pseudo−residuals
Observation index
Sample Quantiles
Theoretical Quantiles
ACF
Lag

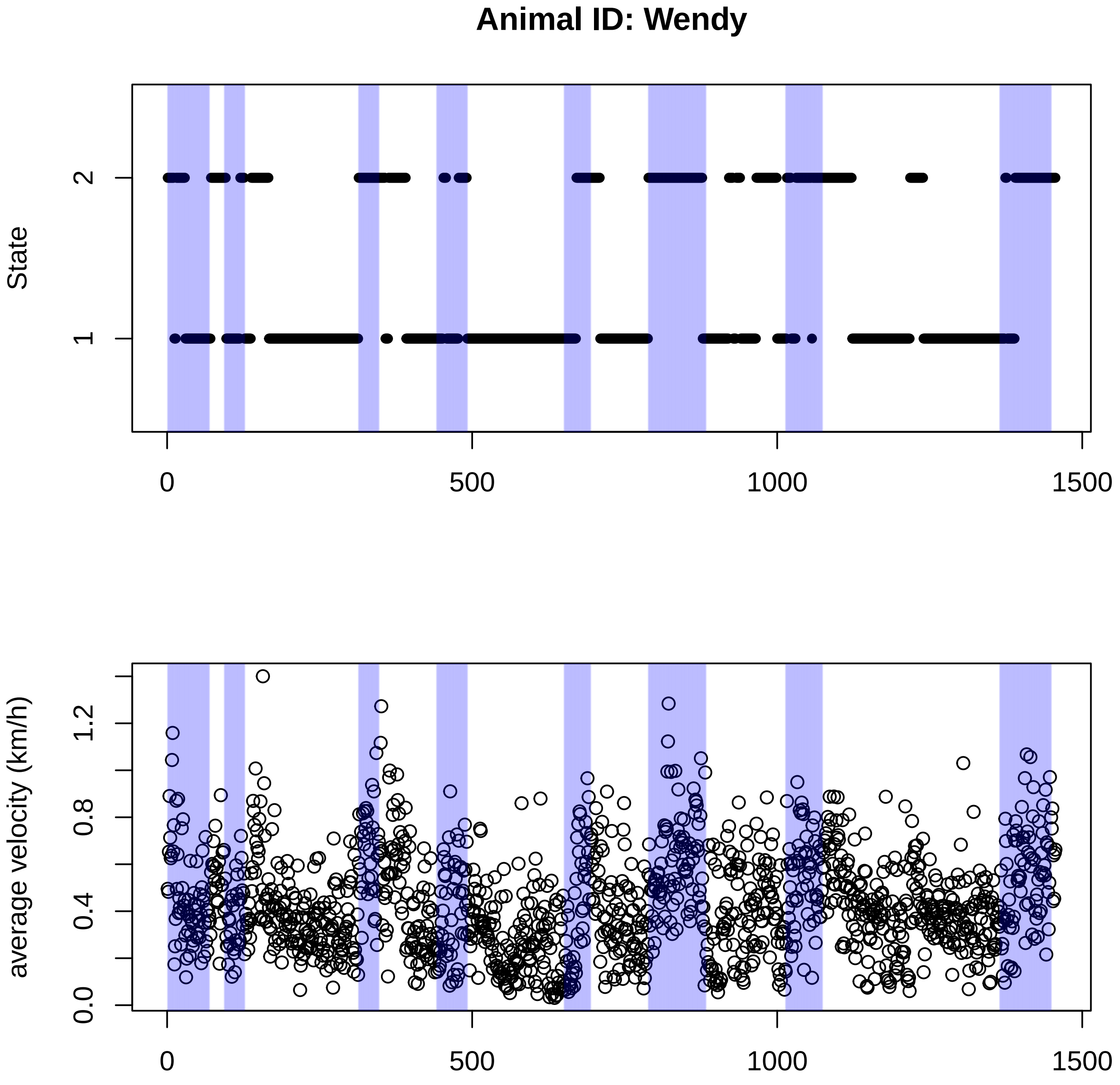
Animal ID: Wendy
State
2
1
0
500
1000
1500
average velocity (km/h)
0.0
0.4
0.8
1.2
0
500
1000
1500

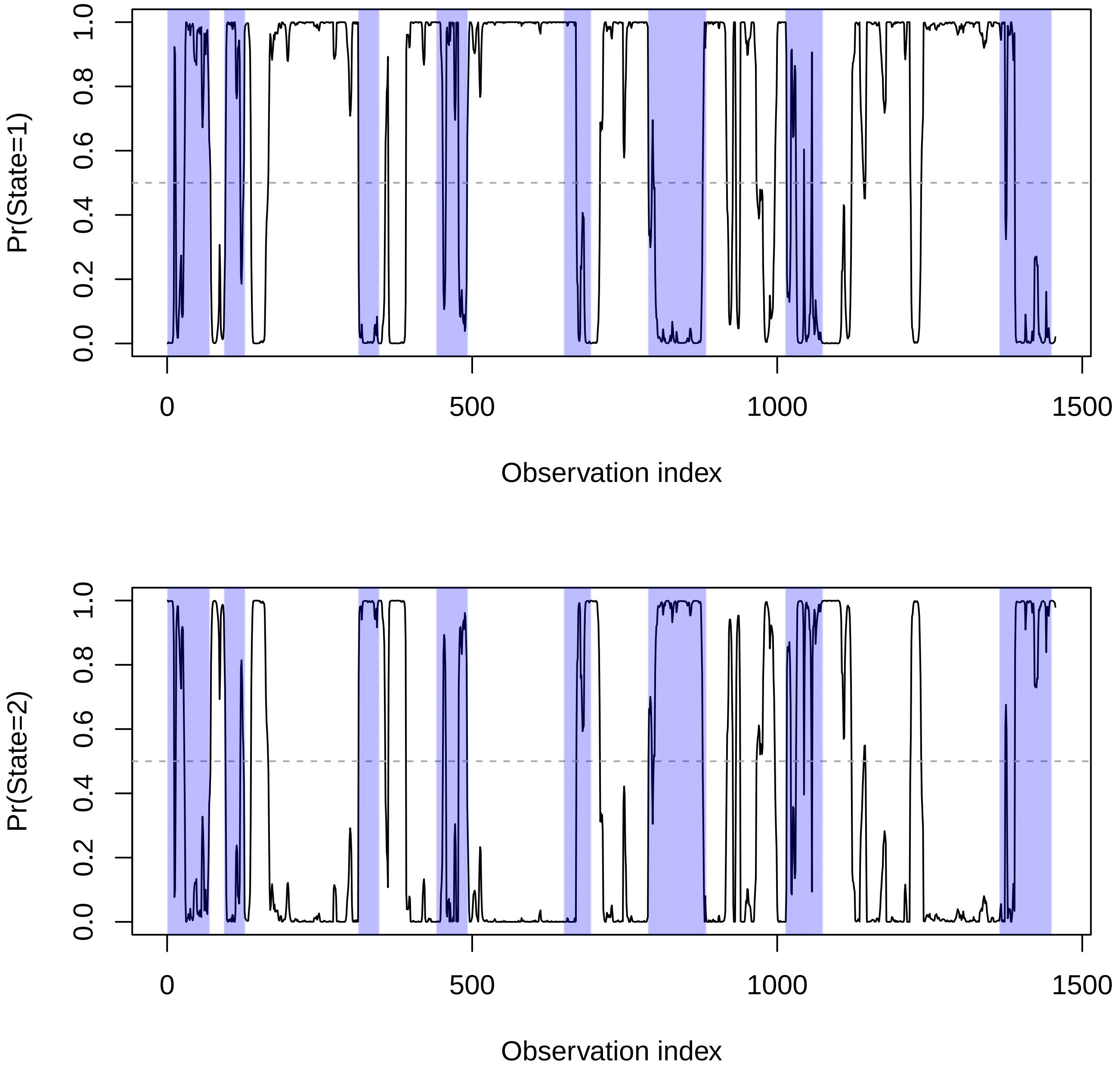

Pr(State=1)
0.0
0.2
0.4
0.6
0.8
1.0
0
500
1000
1500
Observation index
Pr(State=2)
0.0
0.2
0.4
0.6
0.8
1.0
0
500
1000
1500
Observation index

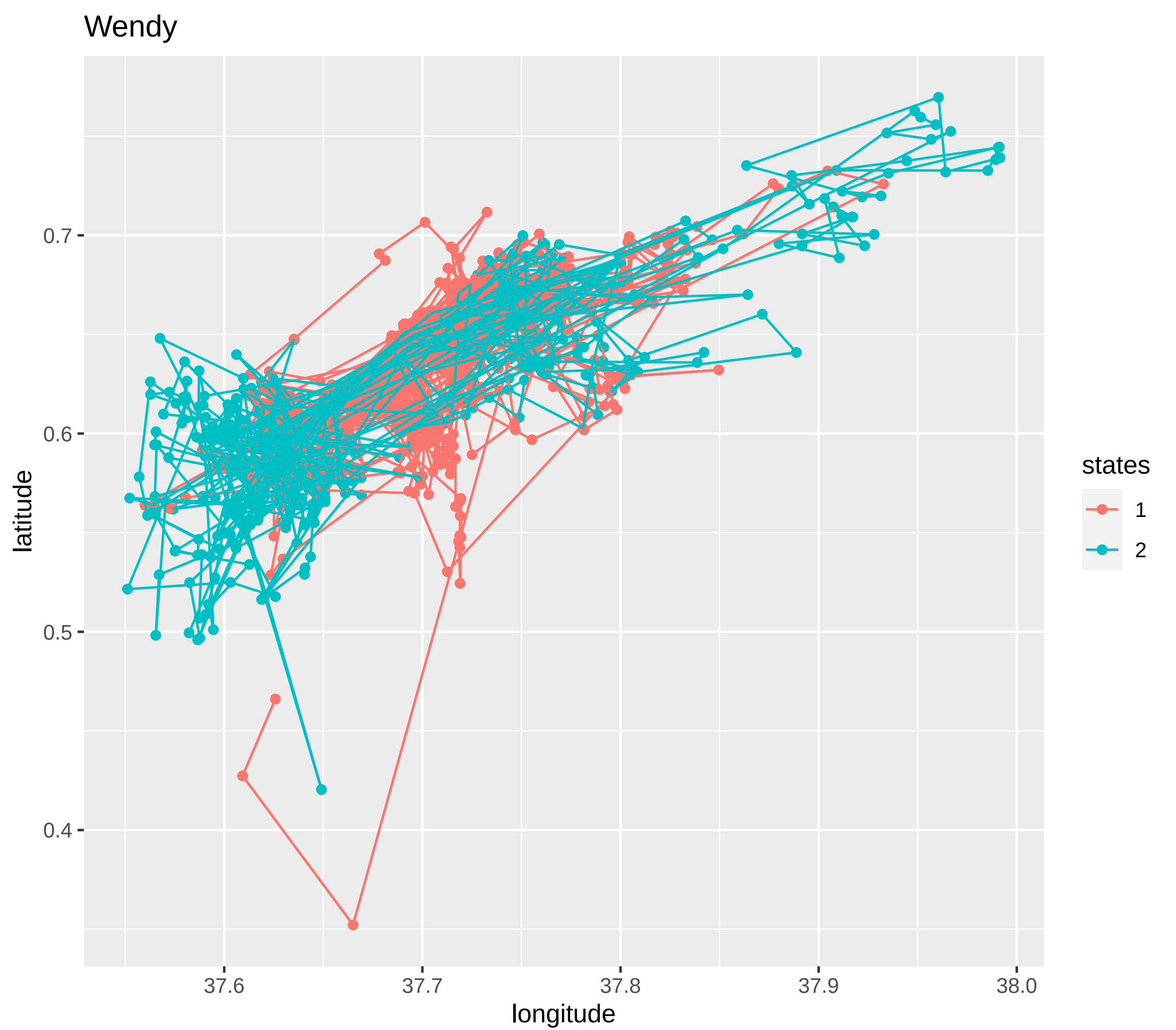

Wendy
latitude
0.7
0.6
0.5
0.4
longitude
37.6
37.7
37.8
37.9
38.0
states
1
2

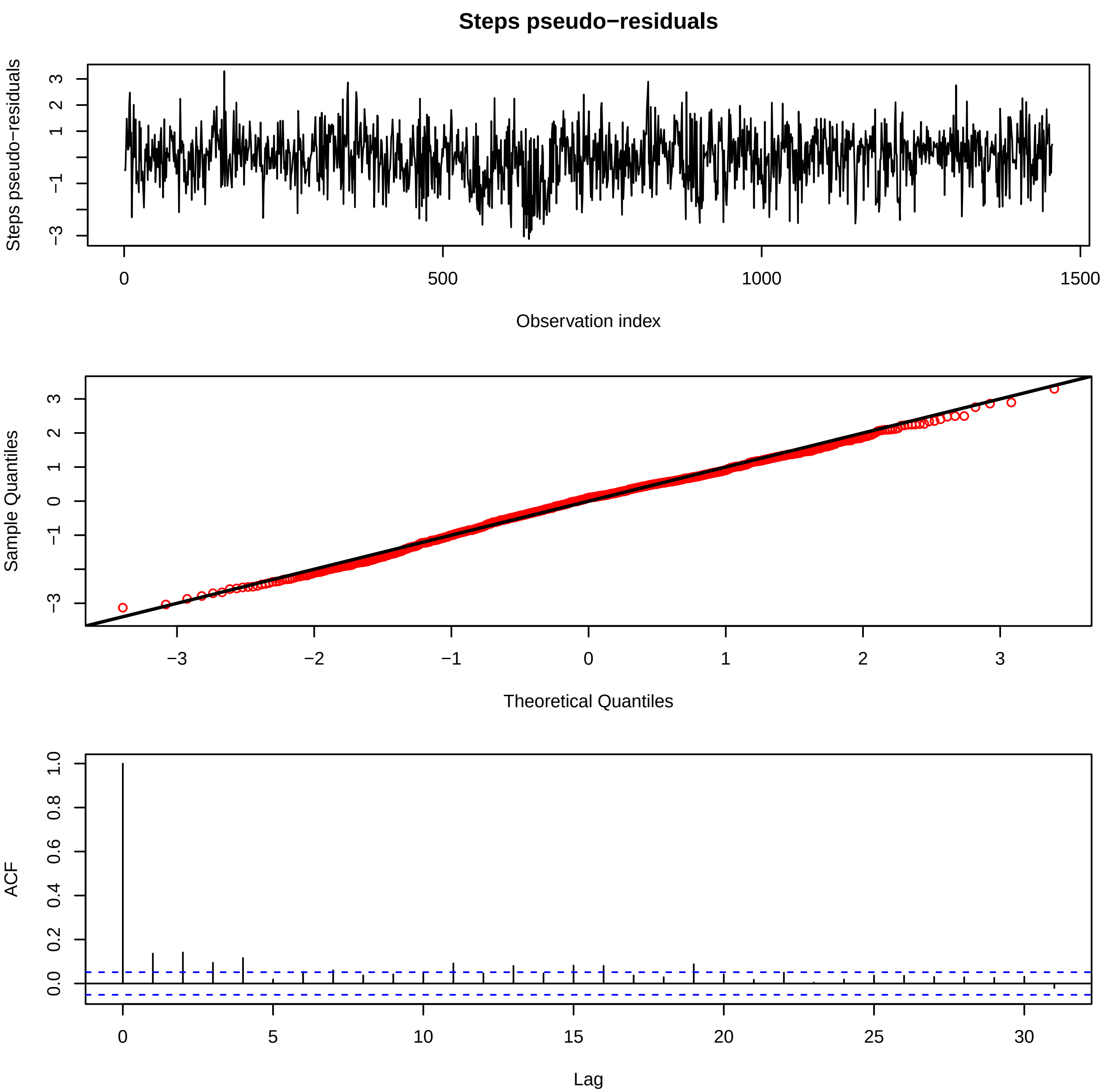

Steps pseudo−residuals
Steps pseudo−residuals
Observation index
Sample Quantiles
Theoretical Quantiles
ACF
Lag

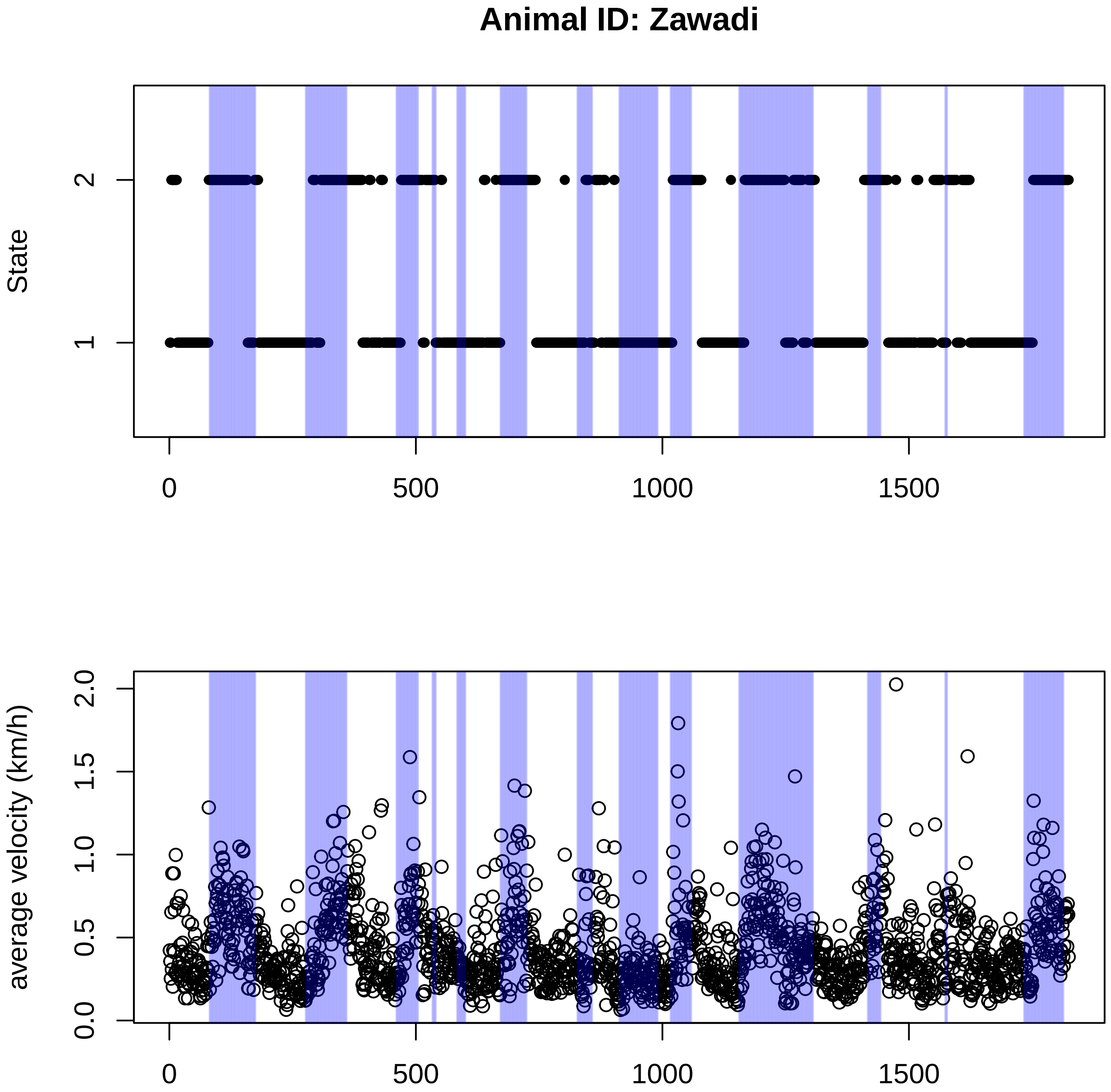

Animal ID: Zawadi
State
2
1
0
500
1000
1500
average velocity (km/h)
0.0
0.5
1.0
1.5
2.0

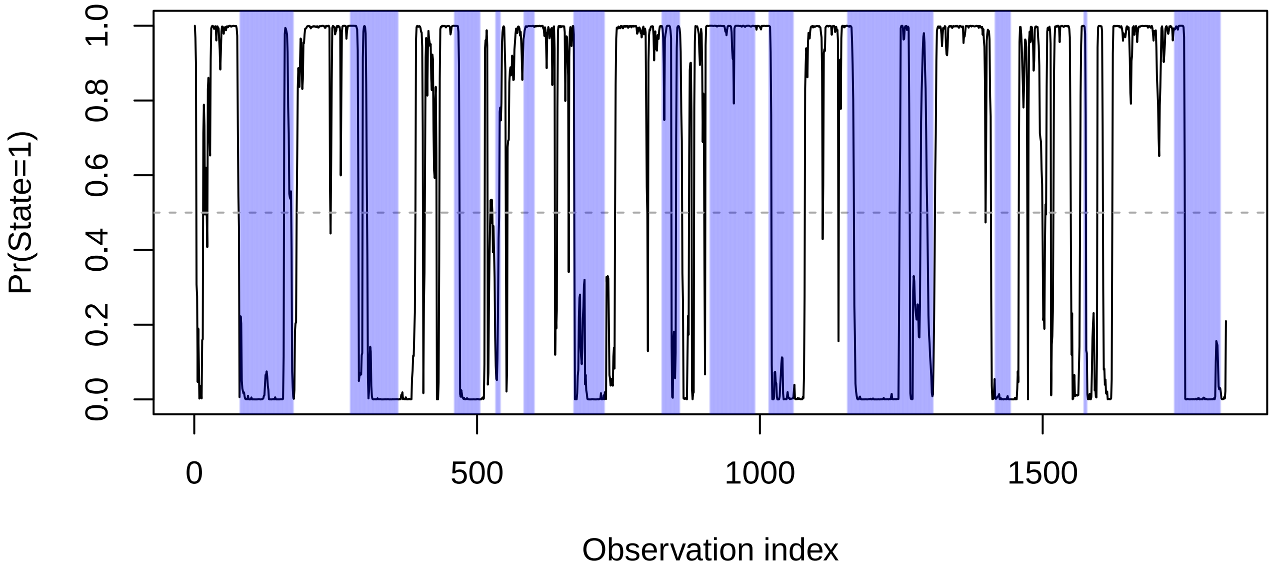

Pr(State=1)
0.0
0.2
0.4
0.6
0.8
1.0
0
500
1000
1500
Observation index

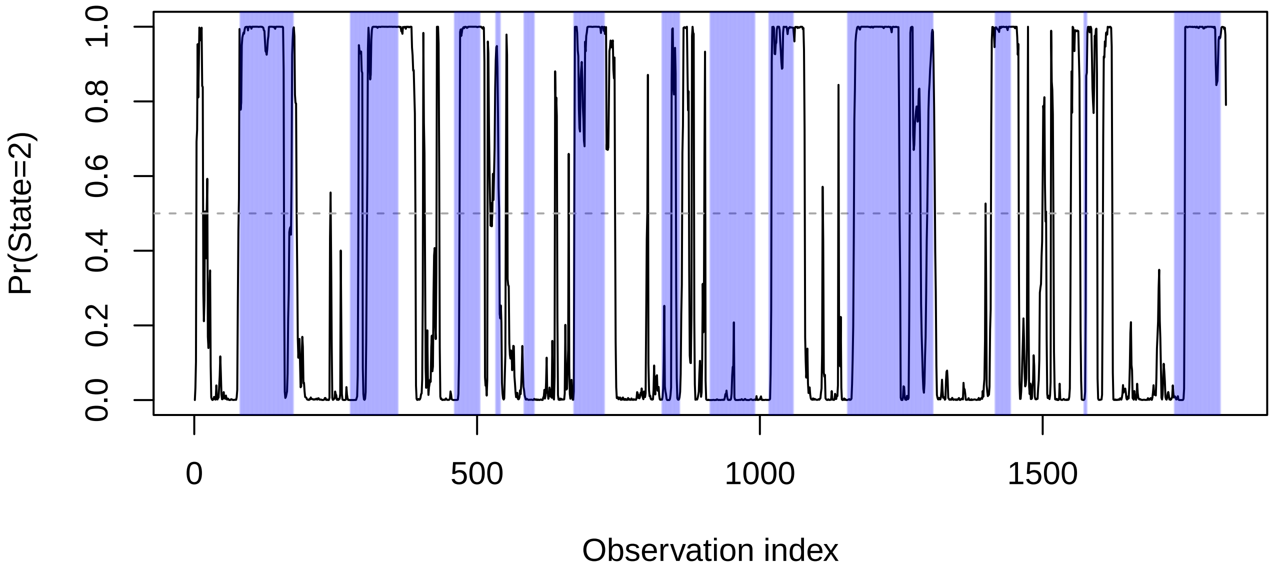

Pr(State=2)
0.0
0.2
0.4
0.6
0.8
1.0
0
500
1000
1500
Observation index

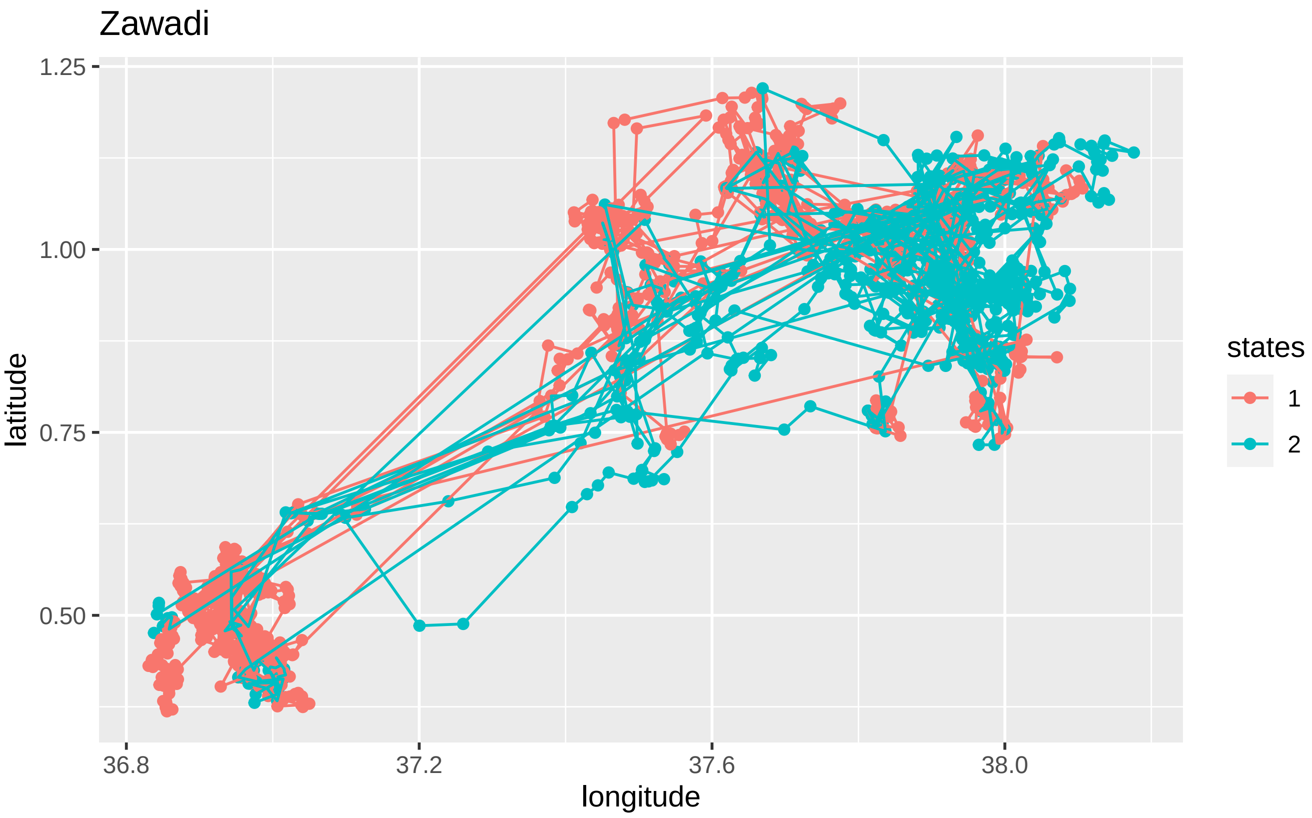
Zawadi
1.25
1.00
0.75
0.50
latitude
36.8
37.2
37.6
38.0
longitude
states
1
2

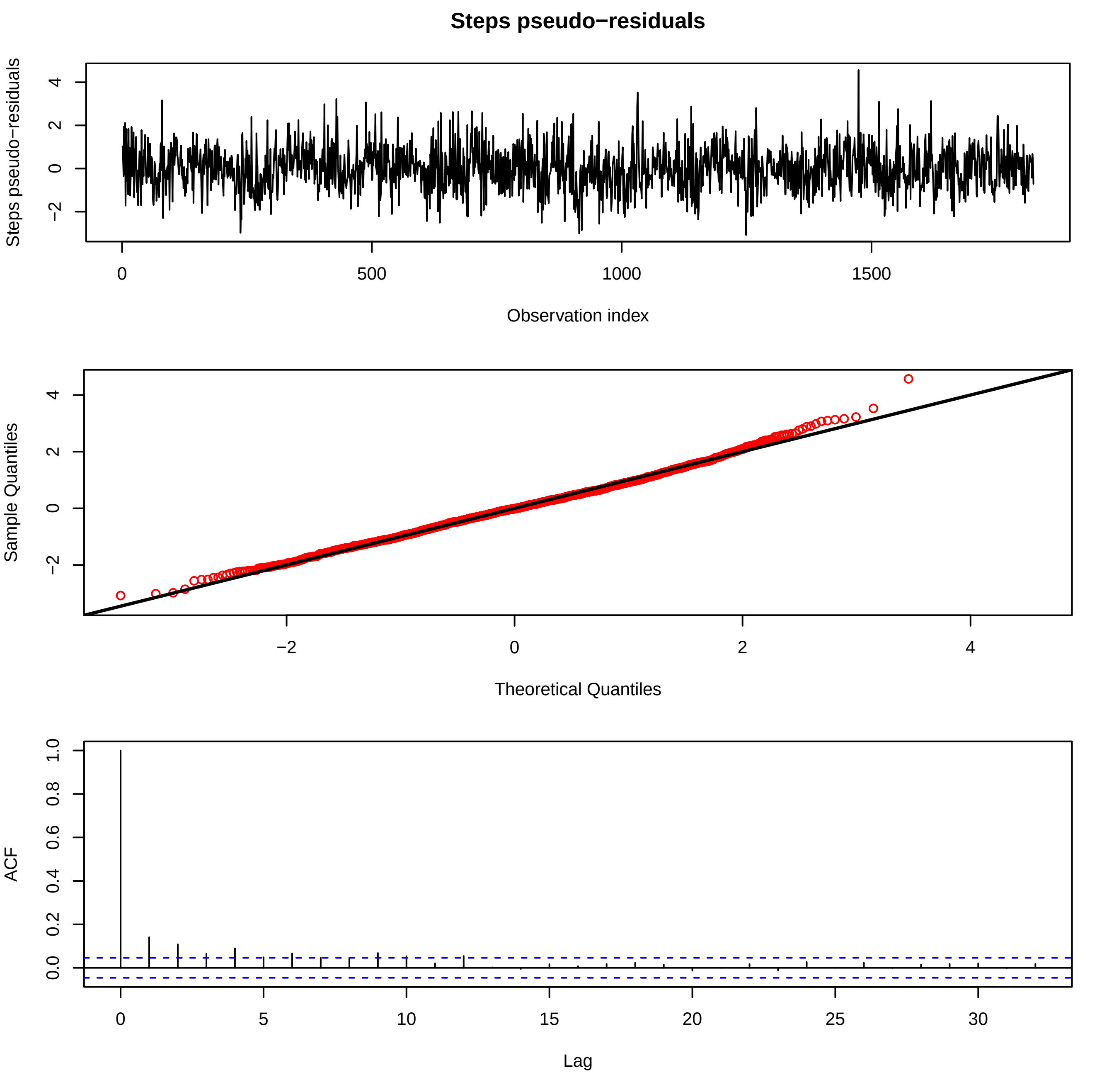

Steps pseudo−residuals
Steps pseudo−residuals
4
2
0
−2
0
500
1000
1500
Observation index
Sample Quantiles
4
2
0
−2
−2
0
2
4
Theoretical Quantiles
ACF
1.0
0.8
0.6
0.4
0.2
0.0
0
5
10
15
20
25
30
Lag
