# Supplementary material for: Local rainfall is more likely than distant thunderstorms to affect movement behaviour in Northern Kenyan elephants
Source: PLoS One. 2024 Dec 23;19(12):e0307520. doi: 10.1371/journal.pone.0307520 (PMC11666045; doi:10.1371/journal.pone.0307520)
Supplement: S4 File — For each elephant and each year the Q-Q plot, residual plot, and persistence velocity BCPA output plots are provided. BCPA output plots indicate abrupt annual changes in persistence velocity marked by vertical purple lines. Colours of points in the BCPA output plots indicate autocorrelation value. (PDF) [file pone.0307520.s004.pdf]

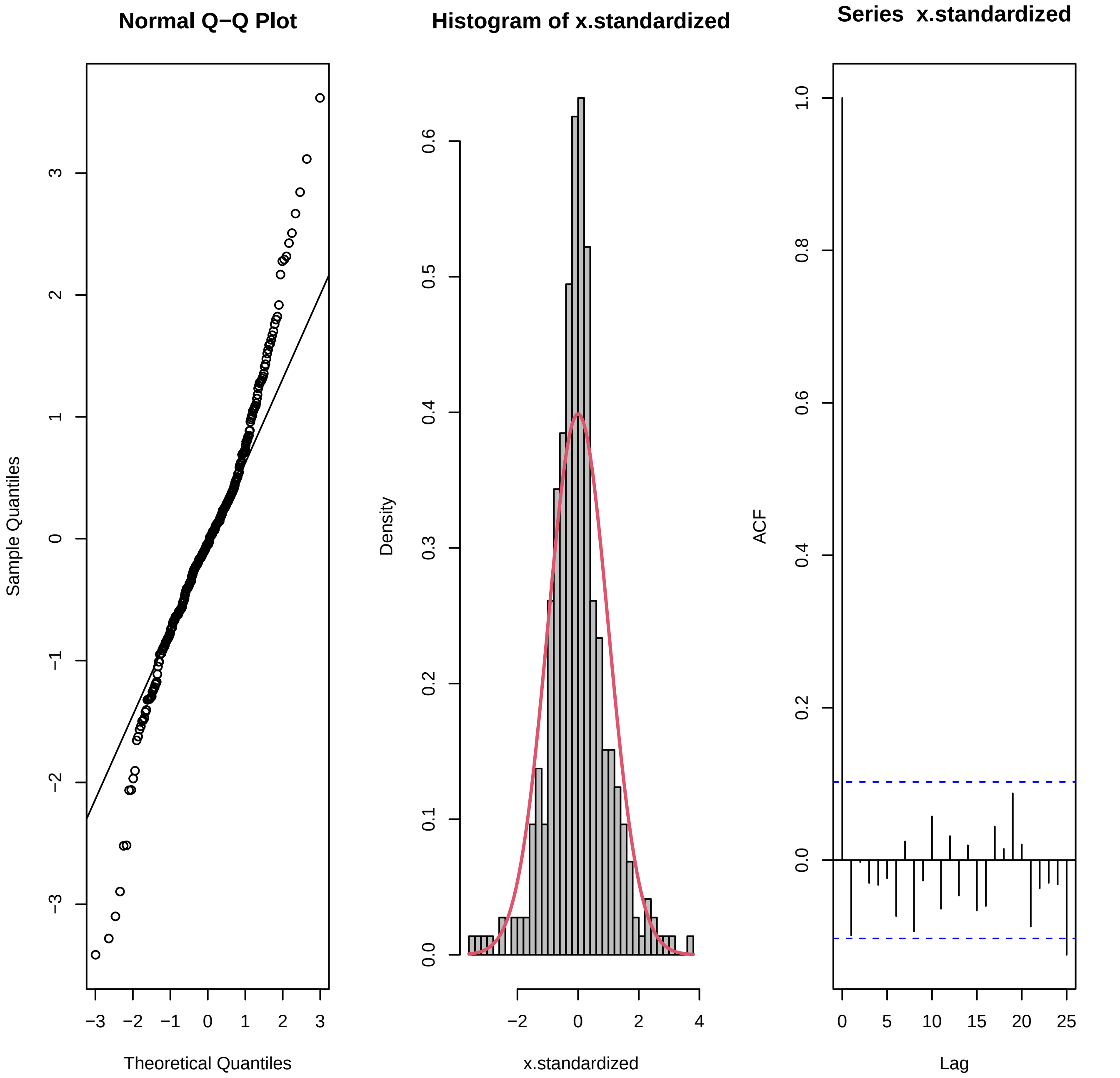
Normal Q−Q Plot
Sample Quantiles
Theoretical Quantiles
−3 −2 −1 0 1 2 3
Histogram of x.standardized
Density
x.standardized
0.0 0.1 0.2 0.3 0.4 0.5 0.6
−2 0 2 4
Series x.standardized
ACF
Lag
0.0 0.2 0.4 0.6 0.8 1.0
0 5 10 15 20 25

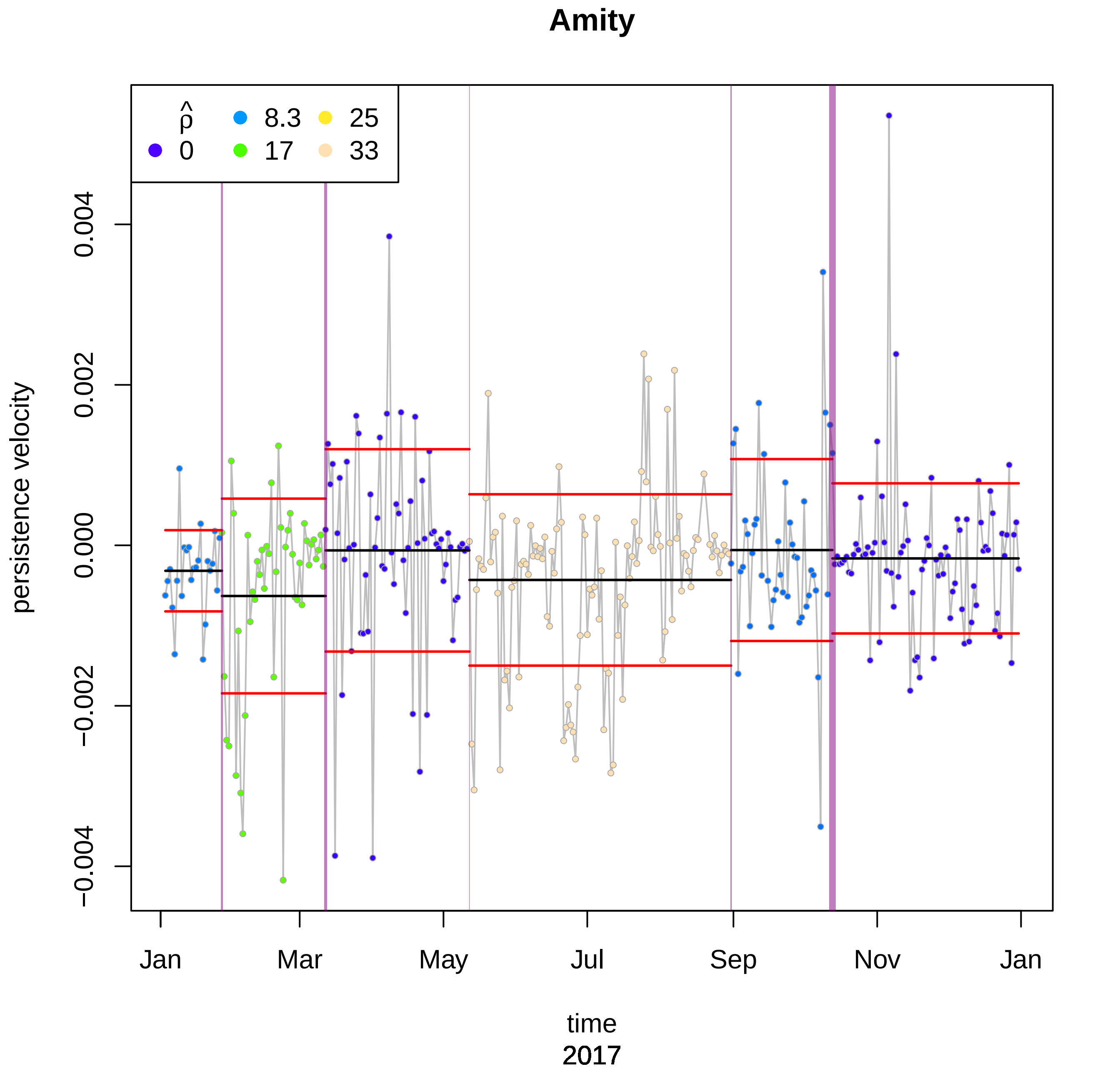

Amity
ρ̂
0
8.3
17
25
33
0.004
0.002
0.000
−0.002
−0.004
persistence velocity
Jan
Mar
May
Jul
Sep
Nov
Jan
time
2017

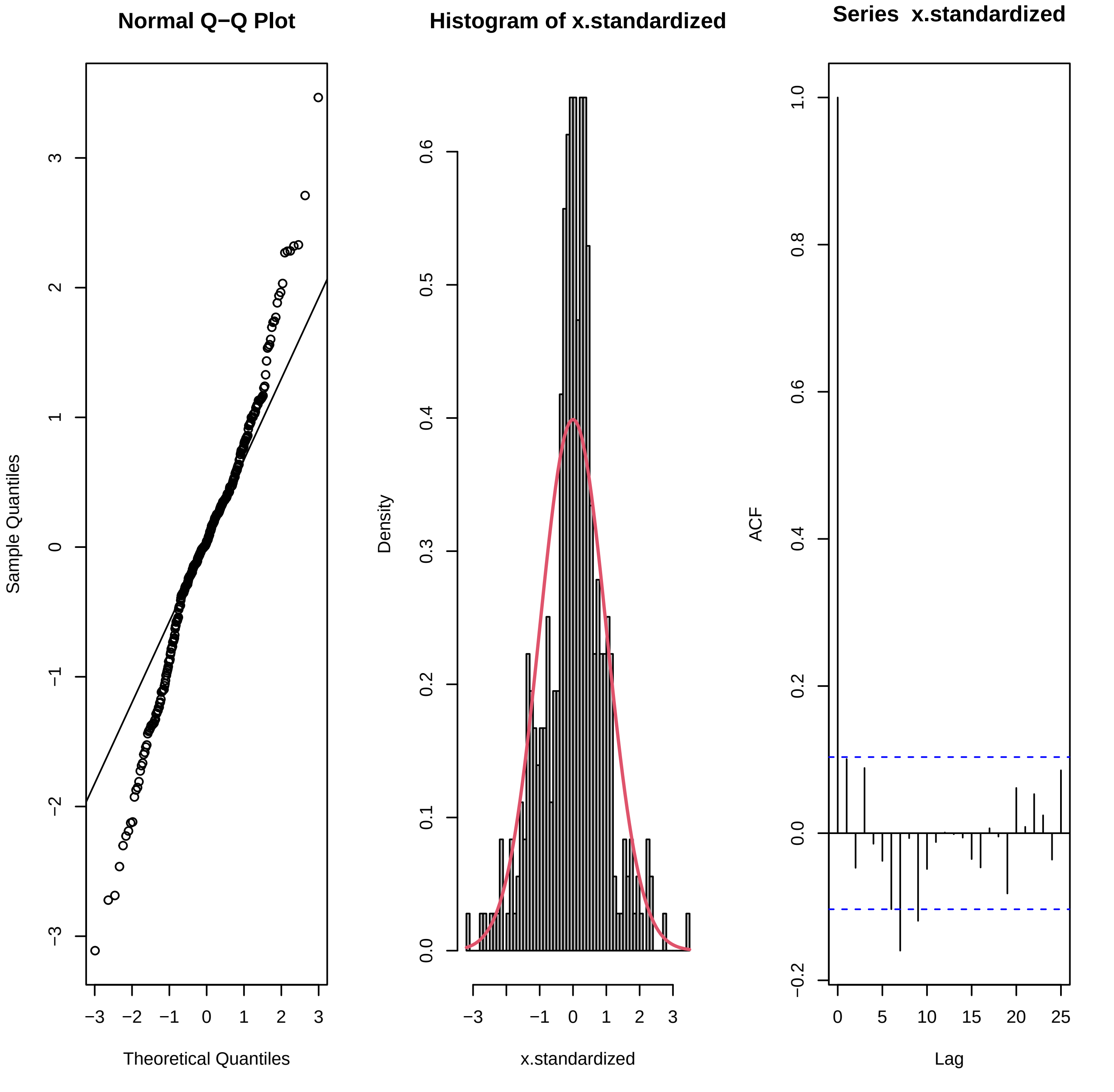

Normal Q−Q Plot
Sample Quantiles
Theoretical Quantiles
−3
−2
−1
0
1
2
3
Histogram of x.standardized
Density
x.standardized
0.0
0.1
0.2
0.3
0.4
0.5
0.6
Series x.standardized
ACF
Lag
−0.2
0.0
0.2
0.4
0.6
0.8
1.0
0
5
10
15
20
25

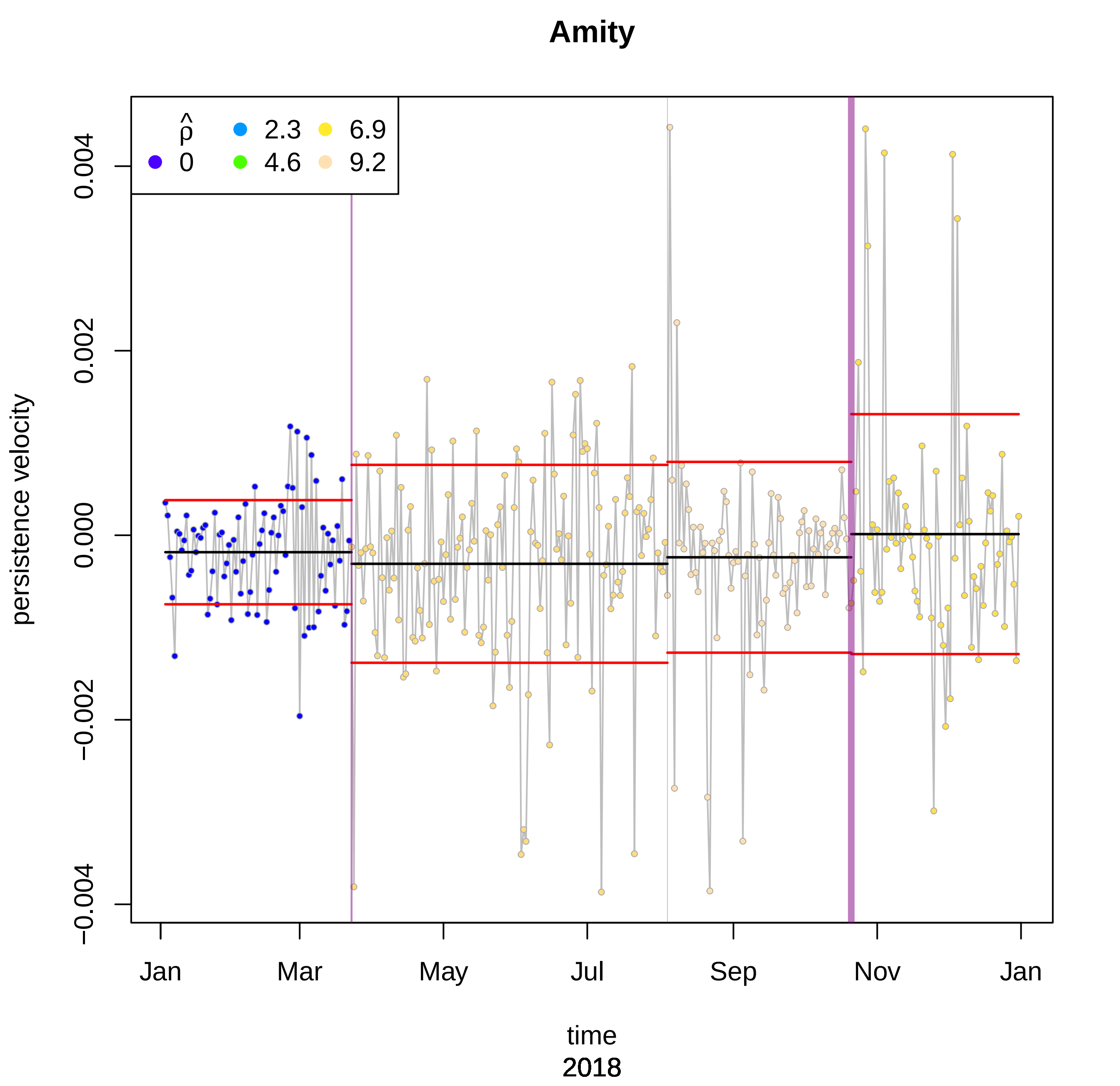

Amity
ρ̂
0
2.3
4.6
6.9
9.2
persistence velocity
0.004
0.002
0.000
−0.002
−0.004
Jan
Mar
May
Jul
Sep
Nov
Jan
time
2018

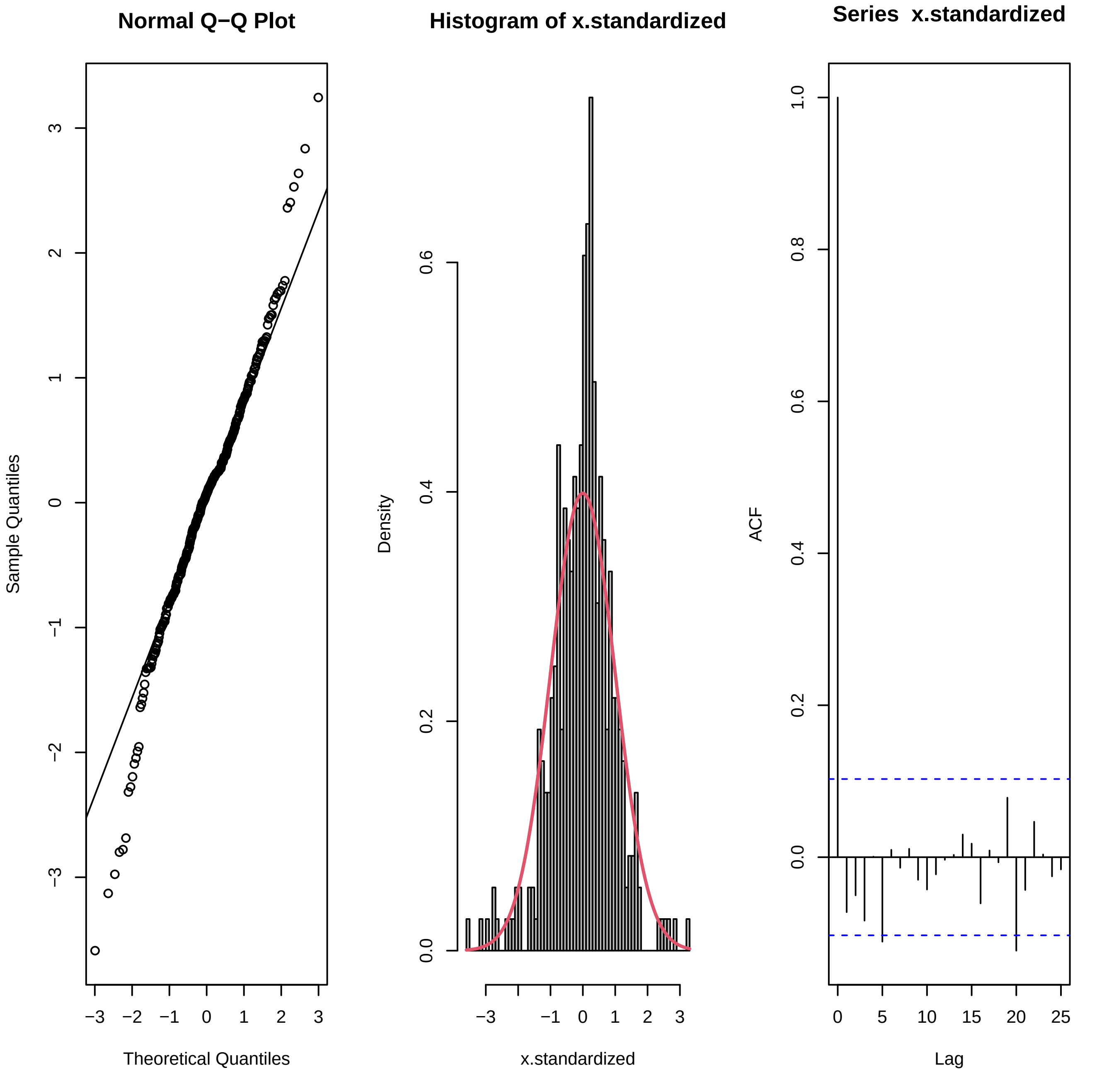

Normal Q−Q Plot
Sample Quantiles
Theoretical Quantiles
Histogram of x.standardized
Density
x.standardized
Series x.standardized
ACF
Lag

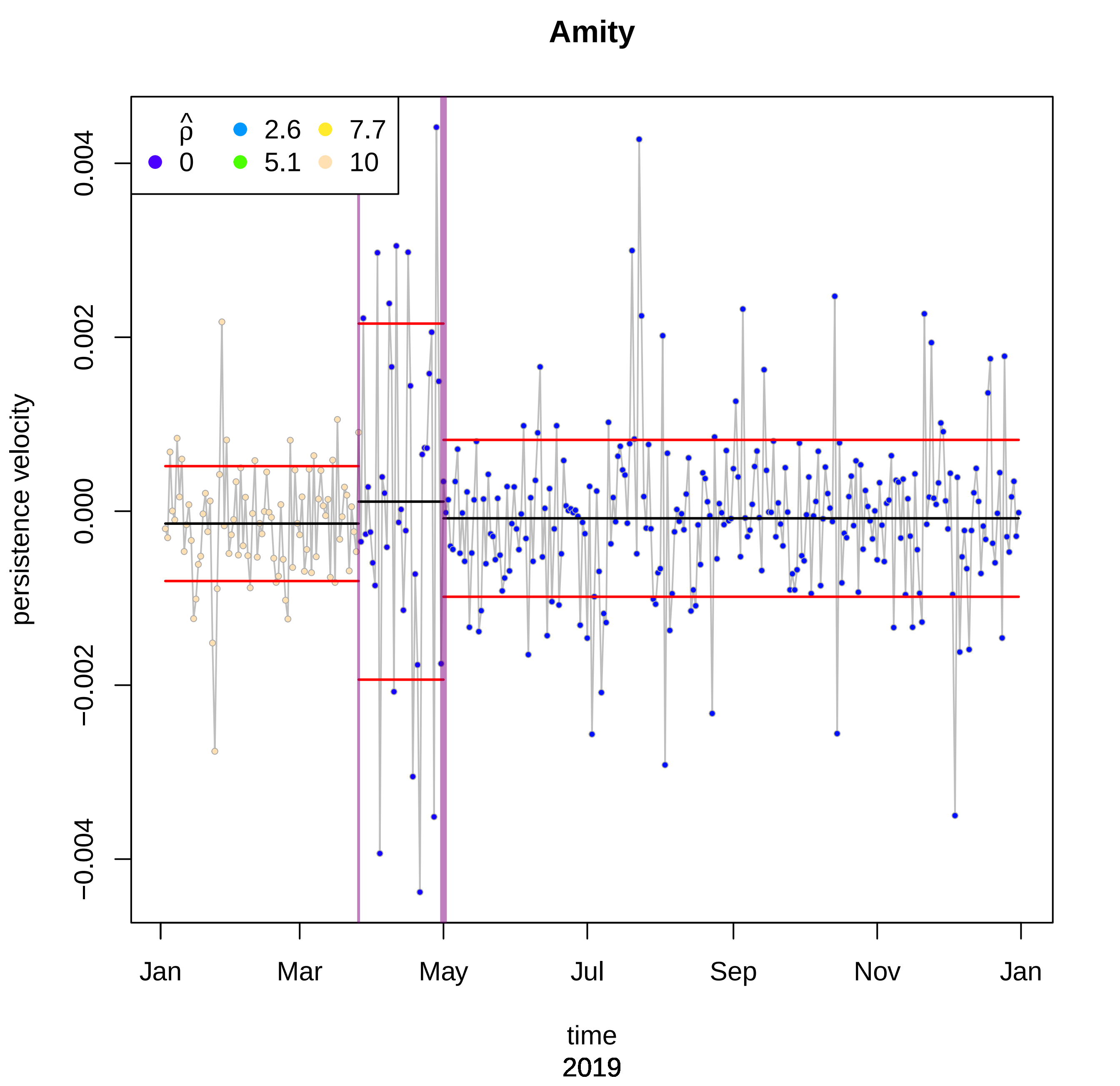

Amity
ρ̂
0
2.6
5.1
7.7
10
0.004
0.002
0.000
−0.002
−0.004
persistence velocity
Jan
Mar
May
Jul
Sep
Nov
Jan
time
2019

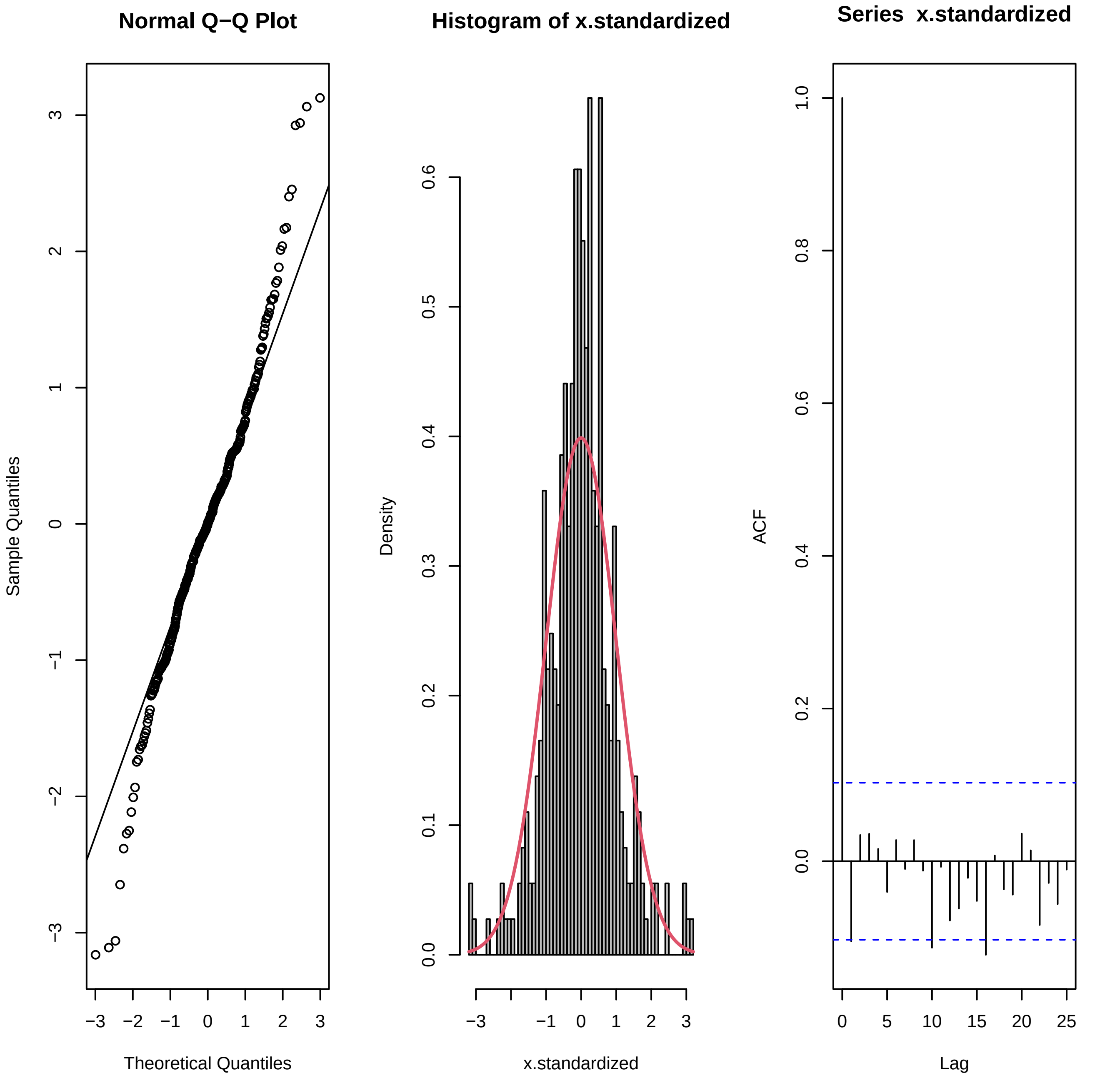

Normal Q−Q Plot
Sample Quantiles
Theoretical Quantiles
−3
−2
−1
0
1
2
3
Histogram of x.standardized
Density
x.standardized
0.0
0.1
0.2
0.3
0.4
0.5
0.6
Series x.standardized
ACF
Lag
0.0
0.2
0.4
0.6
0.8
1.0
0
5
10
15
20
25

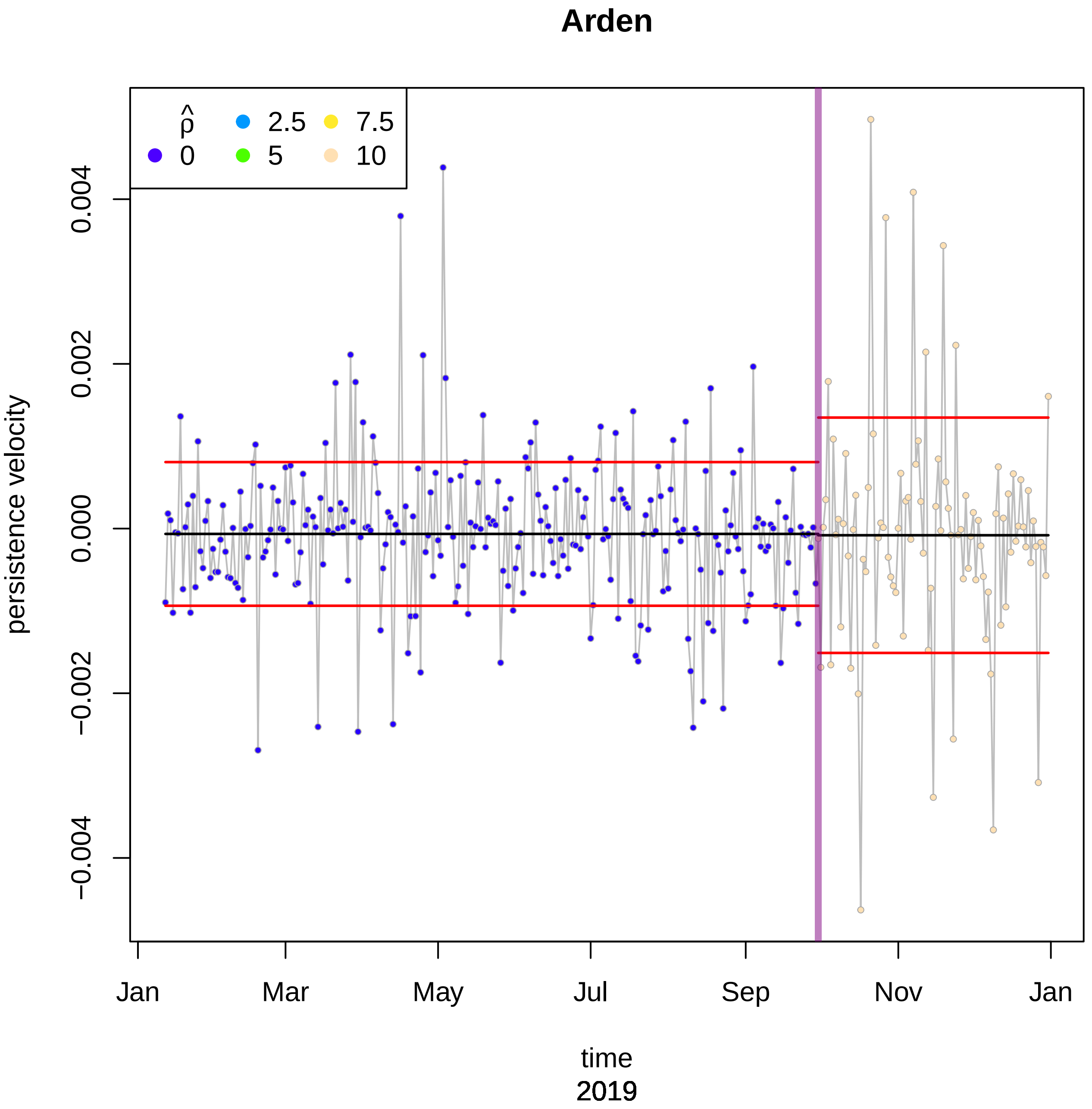

Arden
ρ̂
0
2.5
5
7.5
10
0.004
0.002
0.000
−0.002
−0.004
persistence velocity
Jan
Mar
May
Jul
Sep
Nov
Jan
time
2019

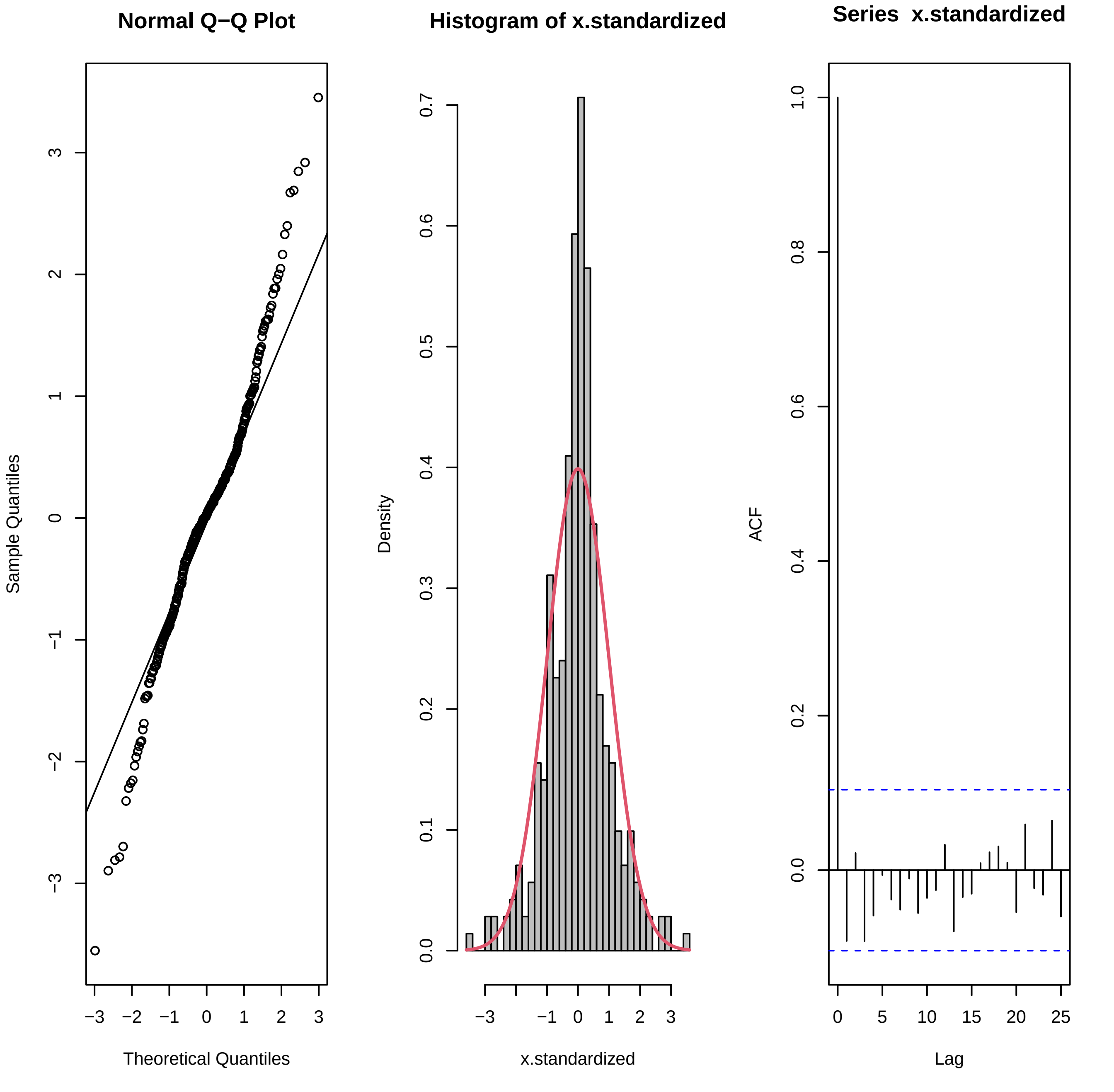

Normal Q−Q Plot
Sample Quantiles
Theoretical Quantiles
−3 −2 −1 0 1 2 3
Histogram of x.standardized
Density
x.standardized
0.0 0.1 0.2 0.3 0.4 0.5 0.6 0.7
−3 −1 0 1 2 3
Series x.standardized
ACF
Lag
0.0 0.2 0.4 0.6 0.8 1.0
0 5 10 15 20 25

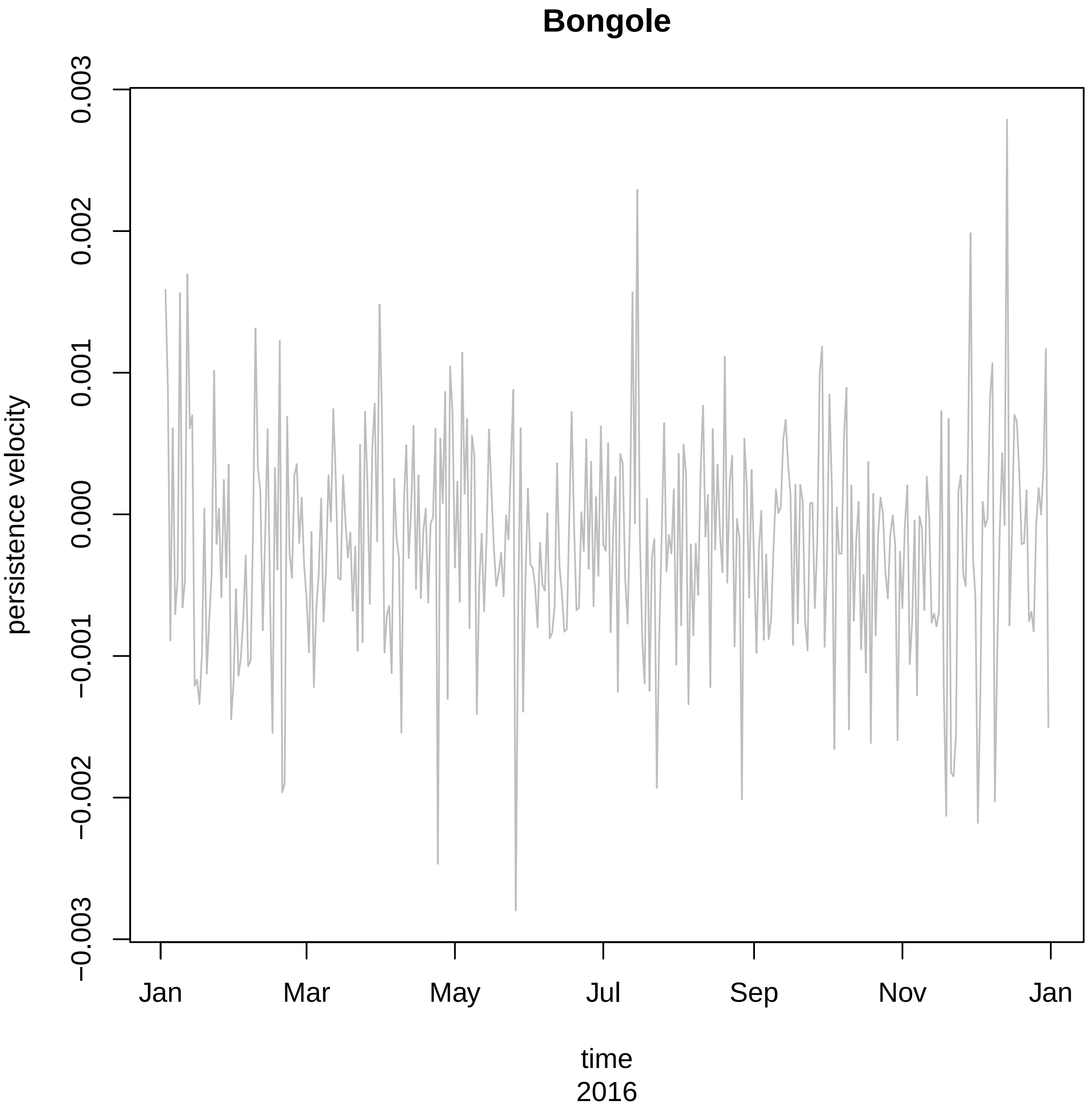
Bongole
persistence velocity
0.003
0.002
0.001
0.000
−0.001
−0.002
−0.003
Jan
Mar
May
Jul
Sep
Nov
Jan
time
2016

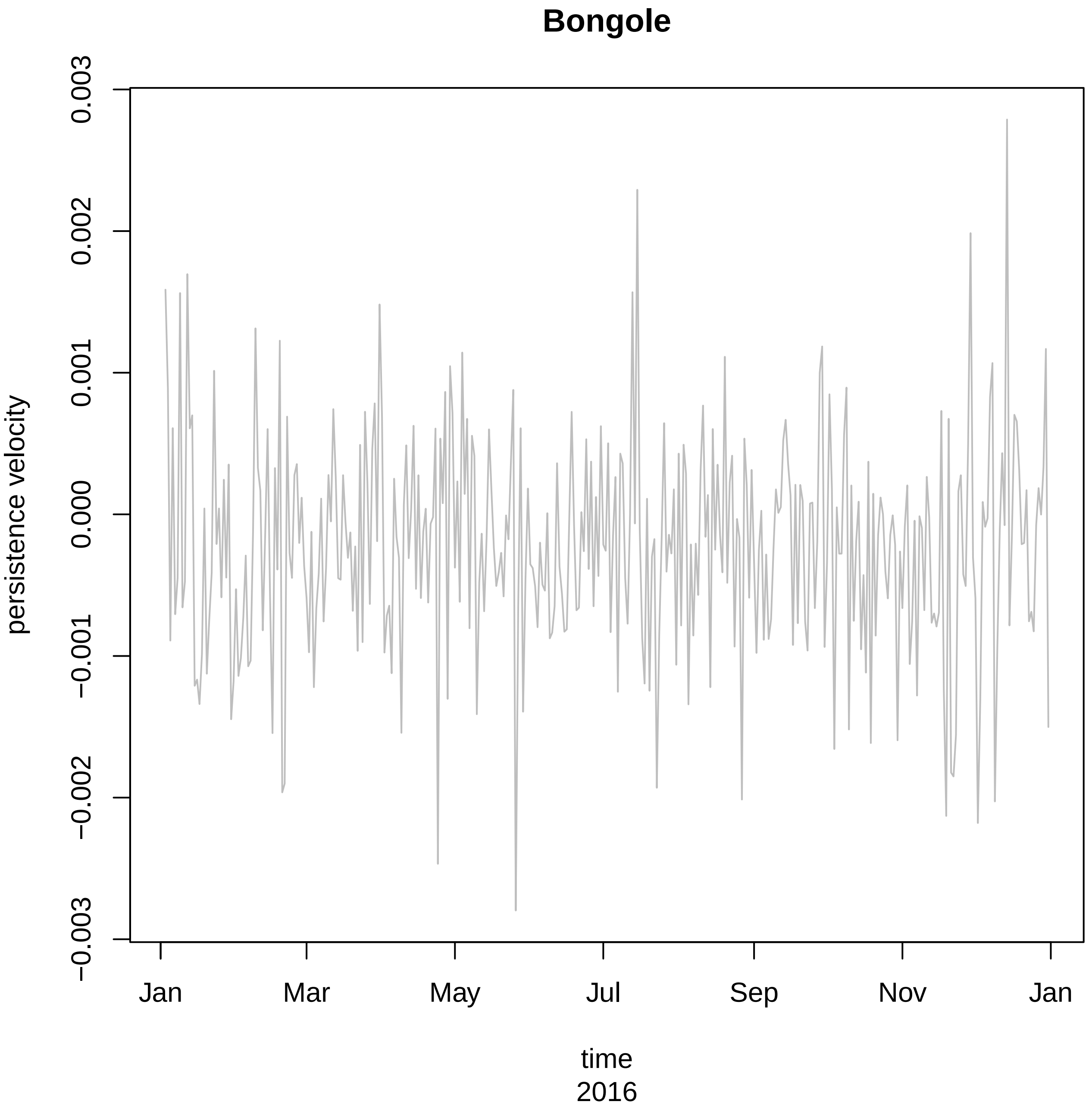
Bongole
persistence velocity
0.003
0.002
0.001
0.000
−0.001
−0.002
−0.003
Jan
Mar
May
Jul
Sep
Nov
Jan
time
2016

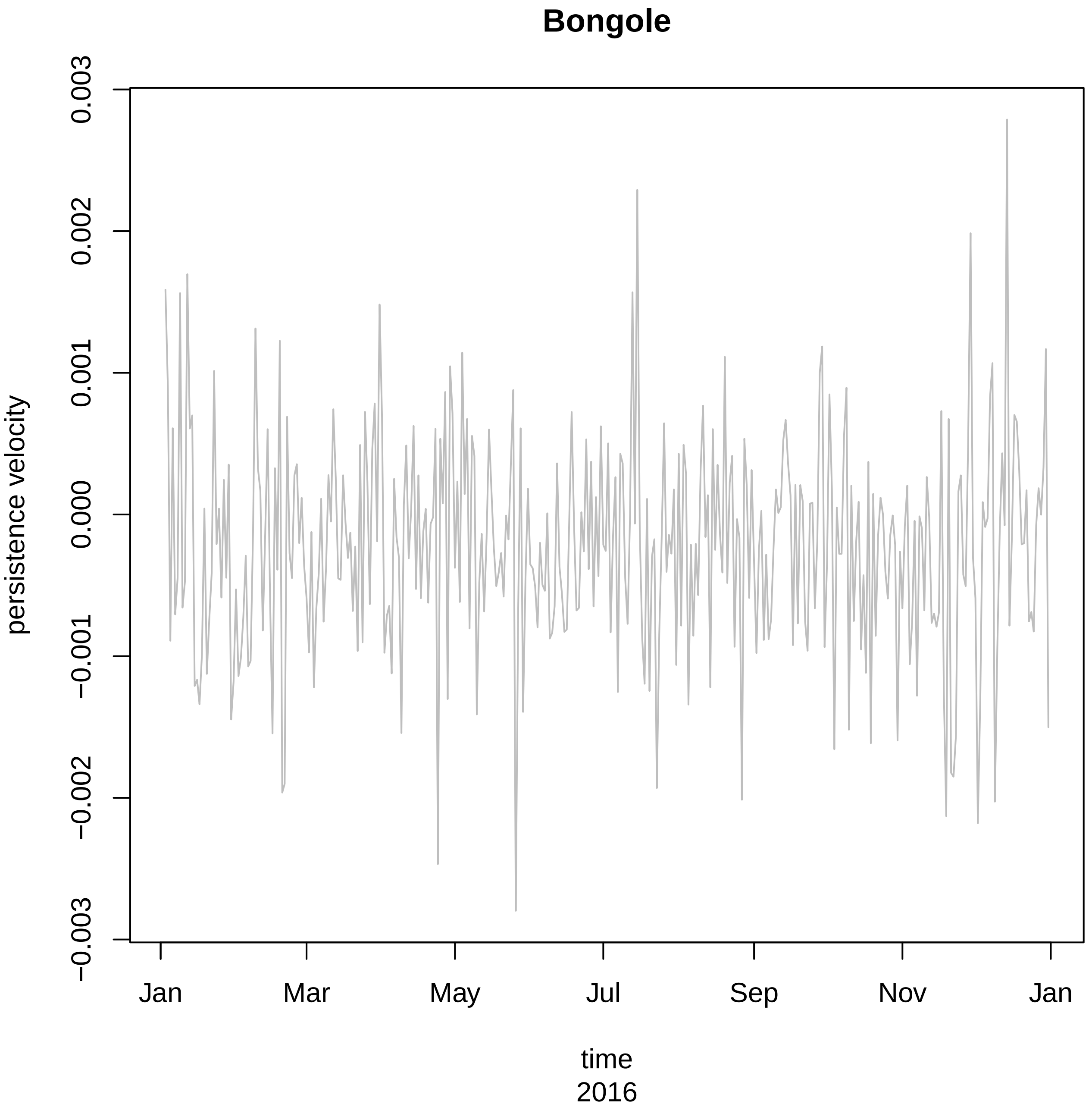

Bongole
persistence velocity
0.003
0.002
0.001
0.000
−0.001
−0.002
−0.003
Jan
Mar
May
Jul
Sep
Nov
Jan
time
2016

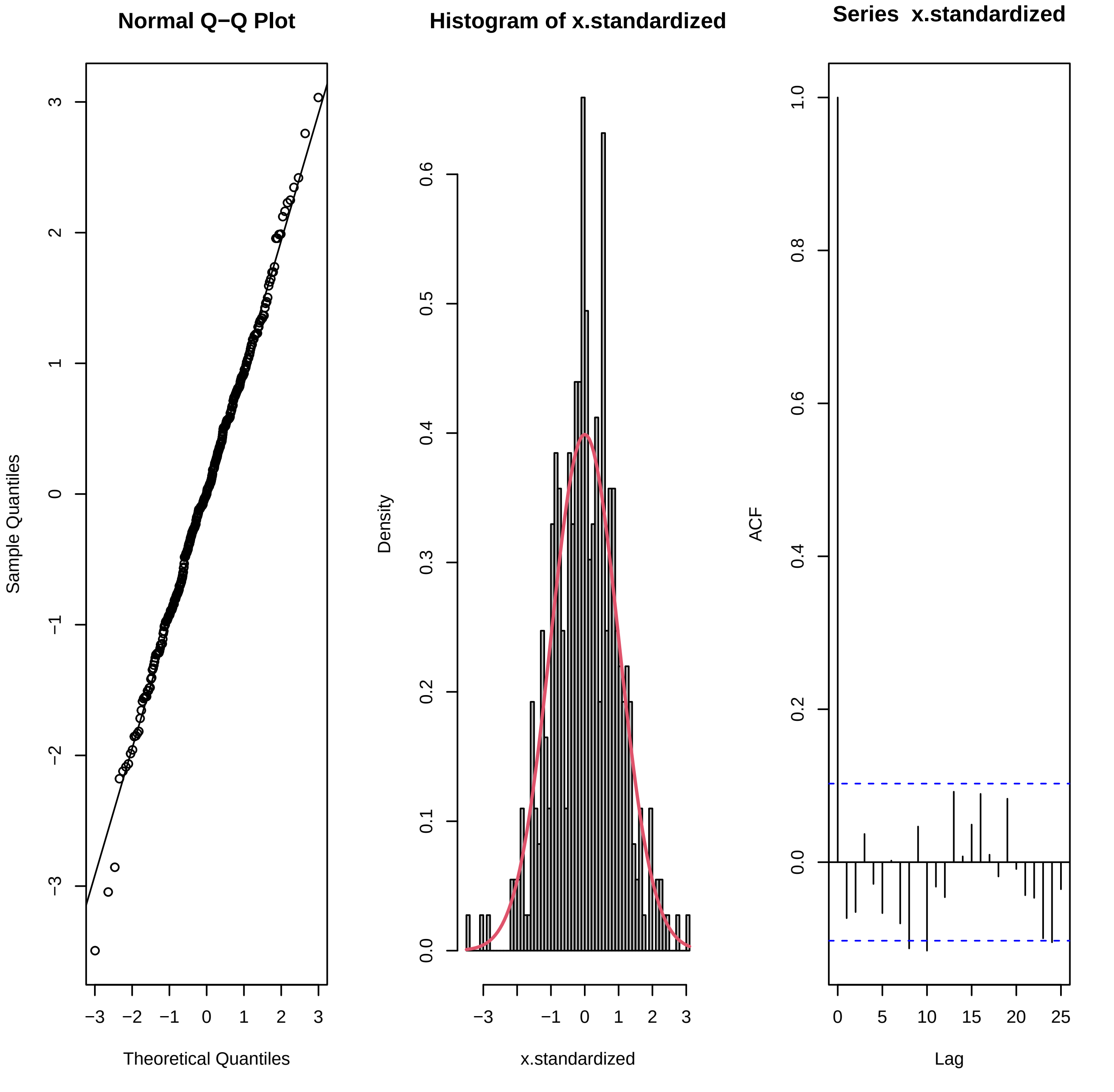

Normal Q−Q Plot
Sample Quantiles
Theoretical Quantiles
−3 −2 −1 0 1 2 3
Histogram of x.standardized
Density
0.0 0.1 0.2 0.3 0.4 0.5 0.6
x.standardized
−3 −1 0 1 2 3
Series x.standardized
ACF
0.0 0.2 0.4 0.6 0.8 1.0
Lag
0 5 10 15 20 25

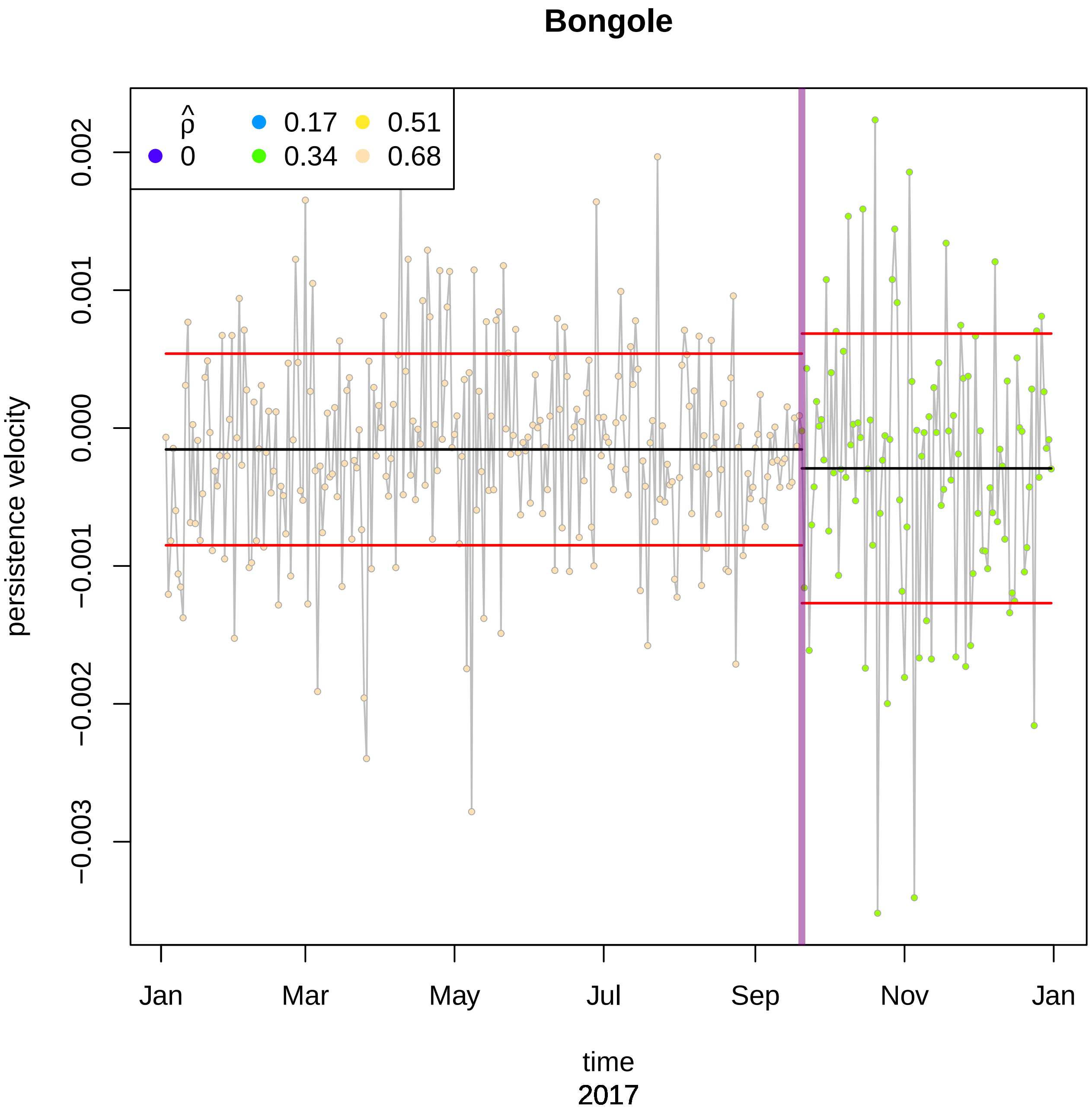

Bongole
ρ̂
0
0.17
0.34
0.51
0.68
persistence velocity
0.002
0.001
0.000
−0.001
−0.002
−0.003
Jan
Mar
May
Jul
Sep
Nov
Jan
time
2017

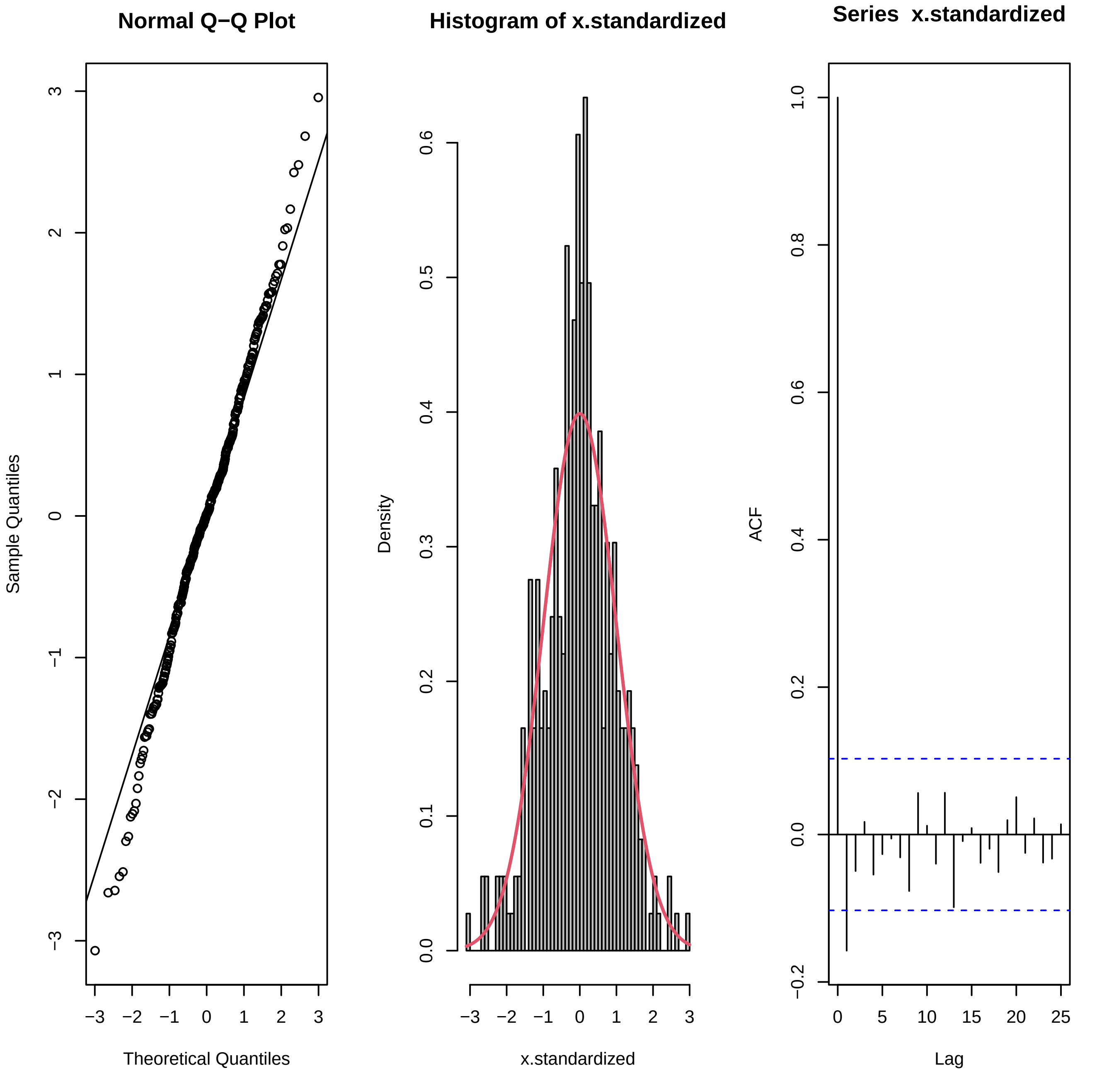
Normal Q−Q Plot
Sample Quantiles
Theoretical Quantiles
−3
−2
−1
0
1
2
3
Histogram of x.standardized
Density
x.standardized
0.0
0.1
0.2
0.3
0.4
0.5
0.6
Series x.standardized
ACF
Lag
−0.2
0.0
0.2
0.4
0.6
0.8
1.0
0
5
10
15
20
25

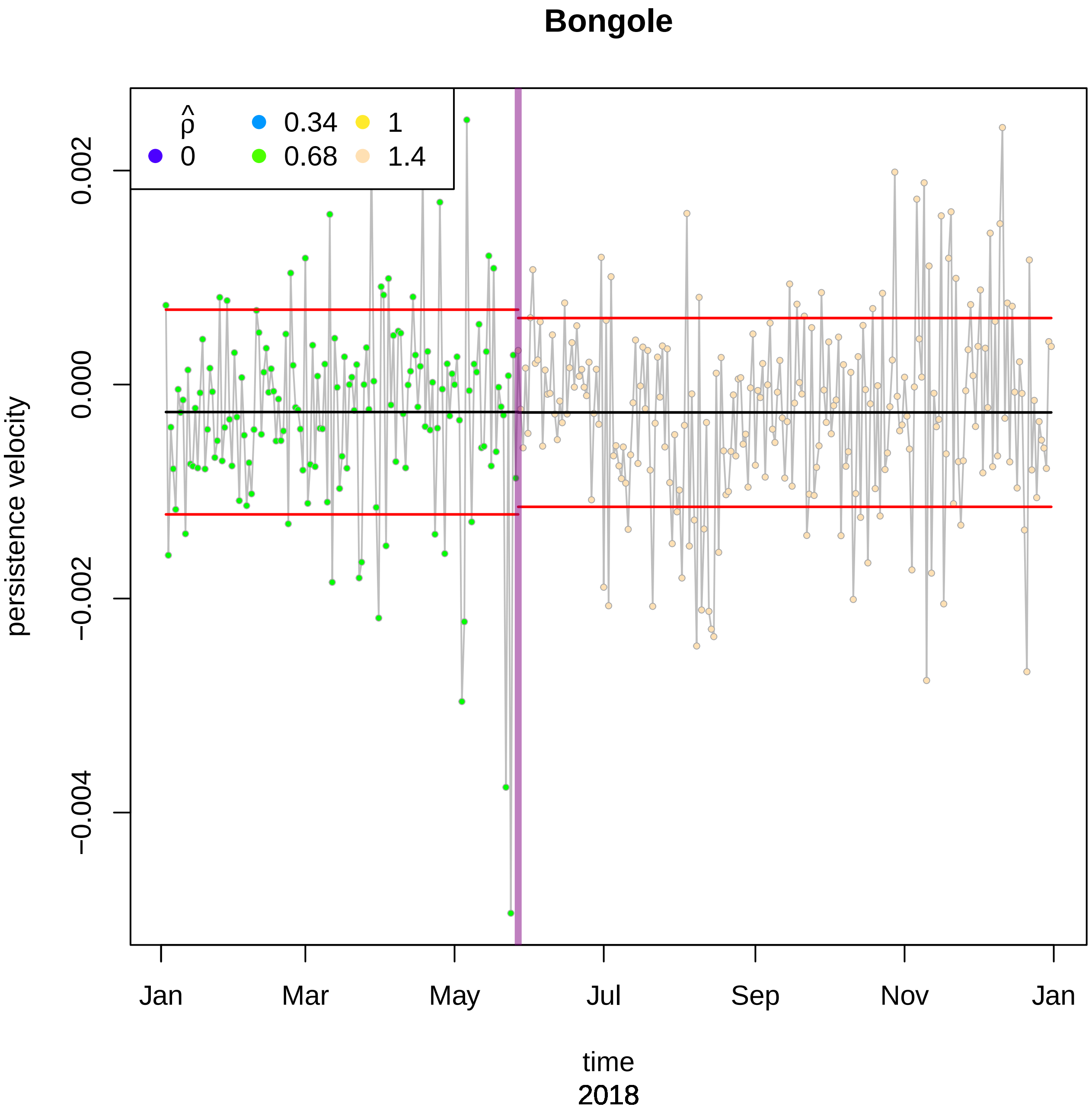

Bongole
ρ̂
0
0.34
0.68
1
1.4
0.002
0.000
−0.002
−0.004
persistence velocity
Jan
Mar
May
Jul
Sep
Nov
Jan
time
2018

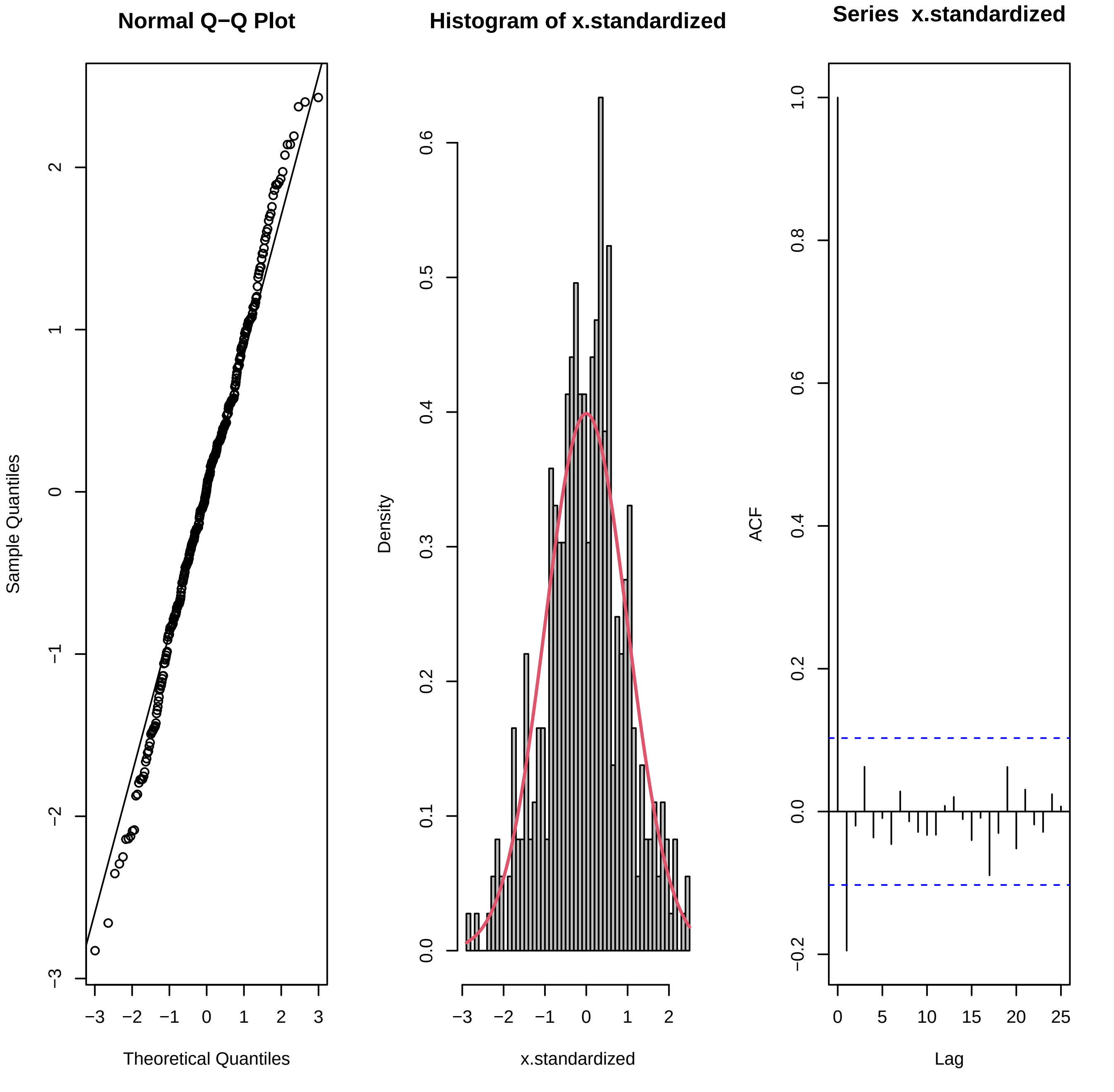

Normal Q−Q Plot
Sample Quantiles
Theoretical Quantiles
−3
−2
−1
0
1
2
3
Histogram of x.standardized
Density
x.standardized
0.0
0.1
0.2
0.3
0.4
0.5
0.6
Series x.standardized
ACF
Lag
−0.2
0.0
0.2
0.4
0.6
0.8
1.0
0
5
10
15
20
25

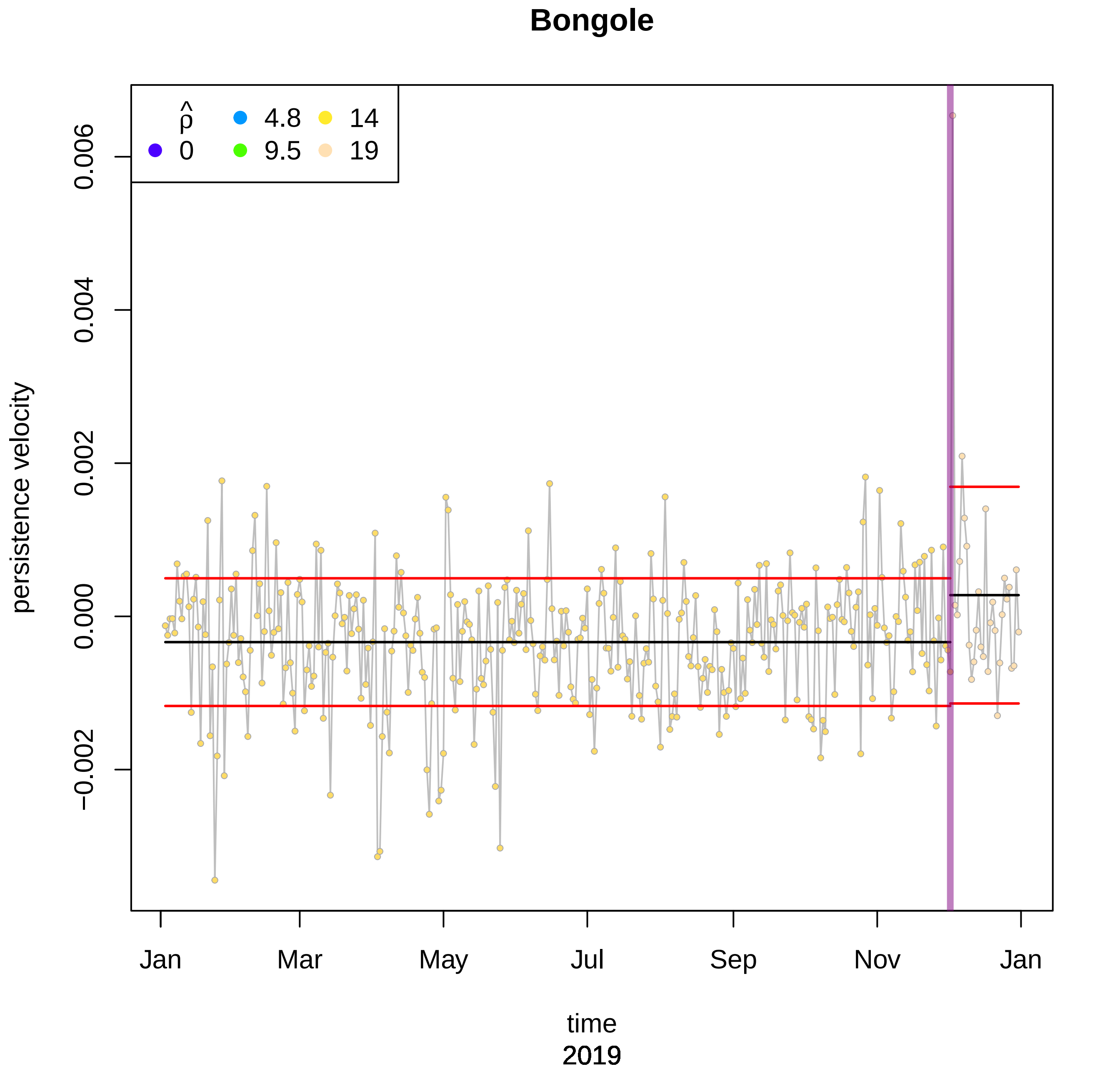

Bongole
ρ̂
0
4.8
9.5
14
19
persistence velocity
0.006
0.004
0.002
0.000
−0.002
Jan
Mar
May
Jul
Sep
Nov
Jan
time
2019

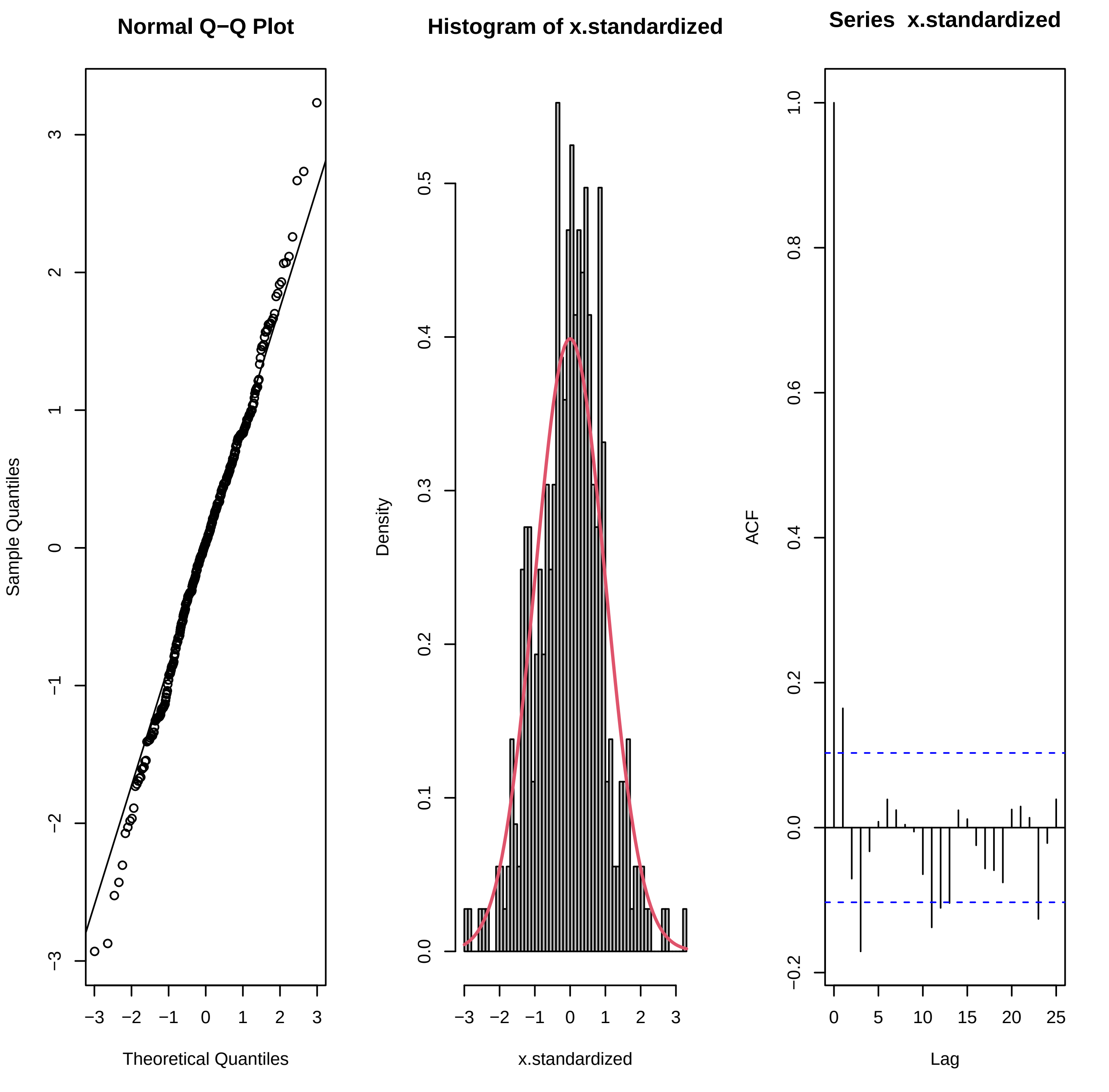

Normal Q−Q Plot
Sample Quantiles
Theoretical Quantiles
−3
−2
−1
0
1
2
3
Histogram of x.standardized
Density
x.standardized
0.0
0.1
0.2
0.3
0.4
0.5
Series x.standardized
ACF
Lag
−0.2
0.0
0.2
0.4
0.6
0.8
1.0
0
5
10
15
20
25

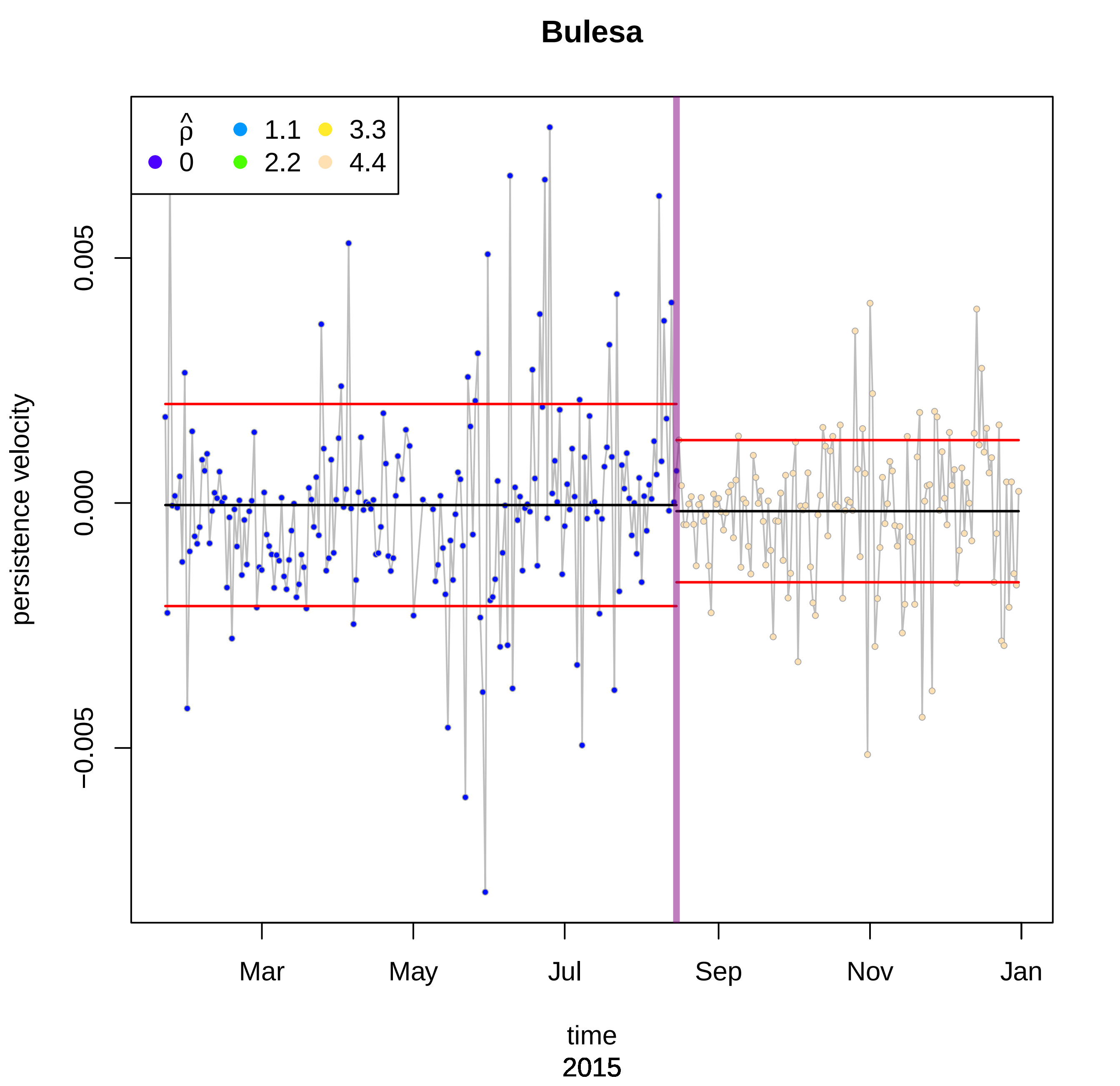

Bulesa
ρ̂
0
1.1
2.2
3.3
4.4
0.005
0.000
−0.005
persistence velocity
Mar
May
Jul
Sep
Nov
Jan
time
2015

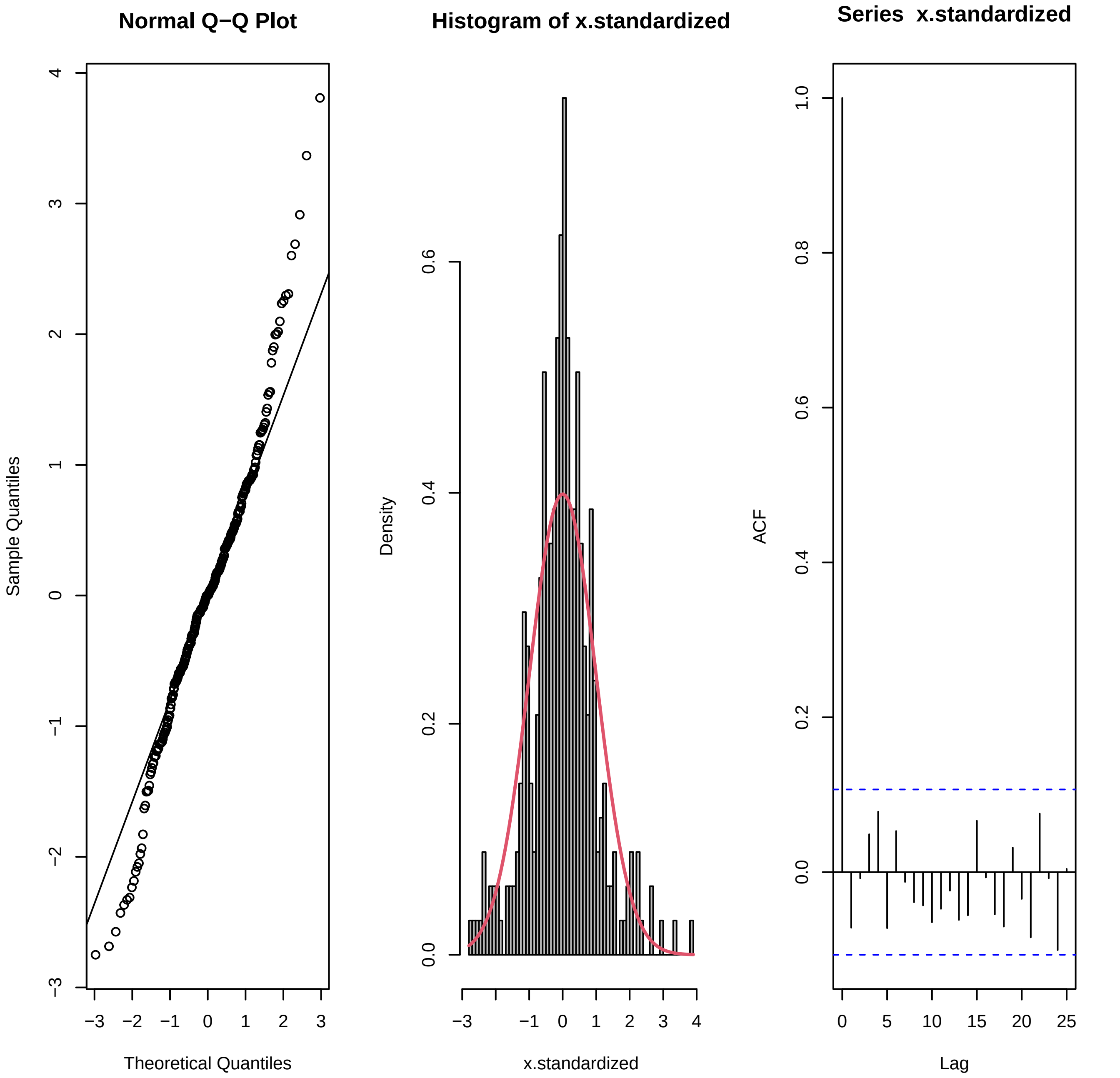
Normal Q−Q Plot
Sample Quantiles
Theoretical Quantiles
Histogram of x.standardized
Density
x.standardized
Series x.standardized
ACF
Lag

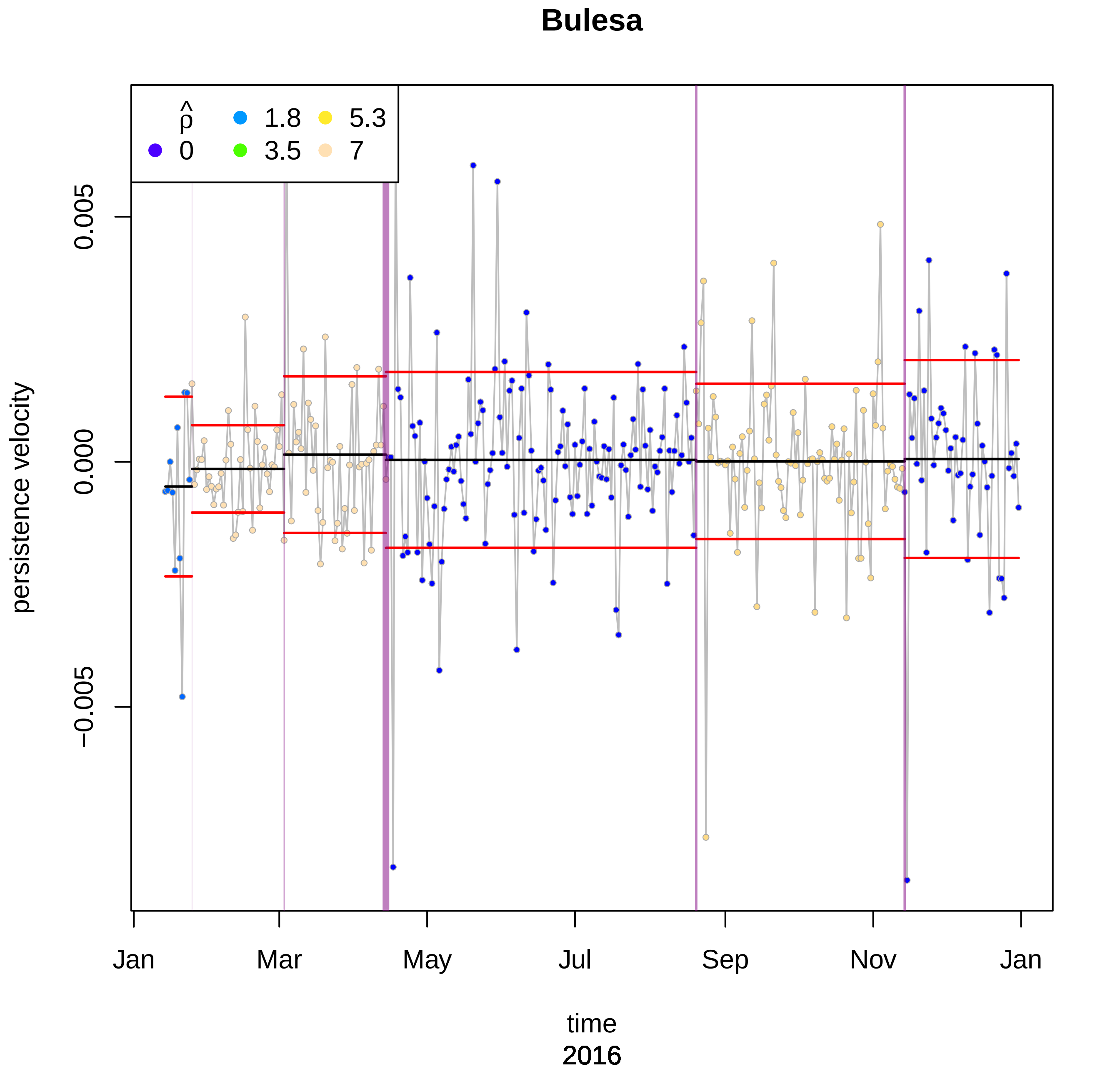

Bulesa
ρ̂
0
1.8
3.5
5.3
7
0.005
0.000
−0.005
persistence velocity
Jan
Mar
May
Jul
Sep
Nov
Jan
time
2016

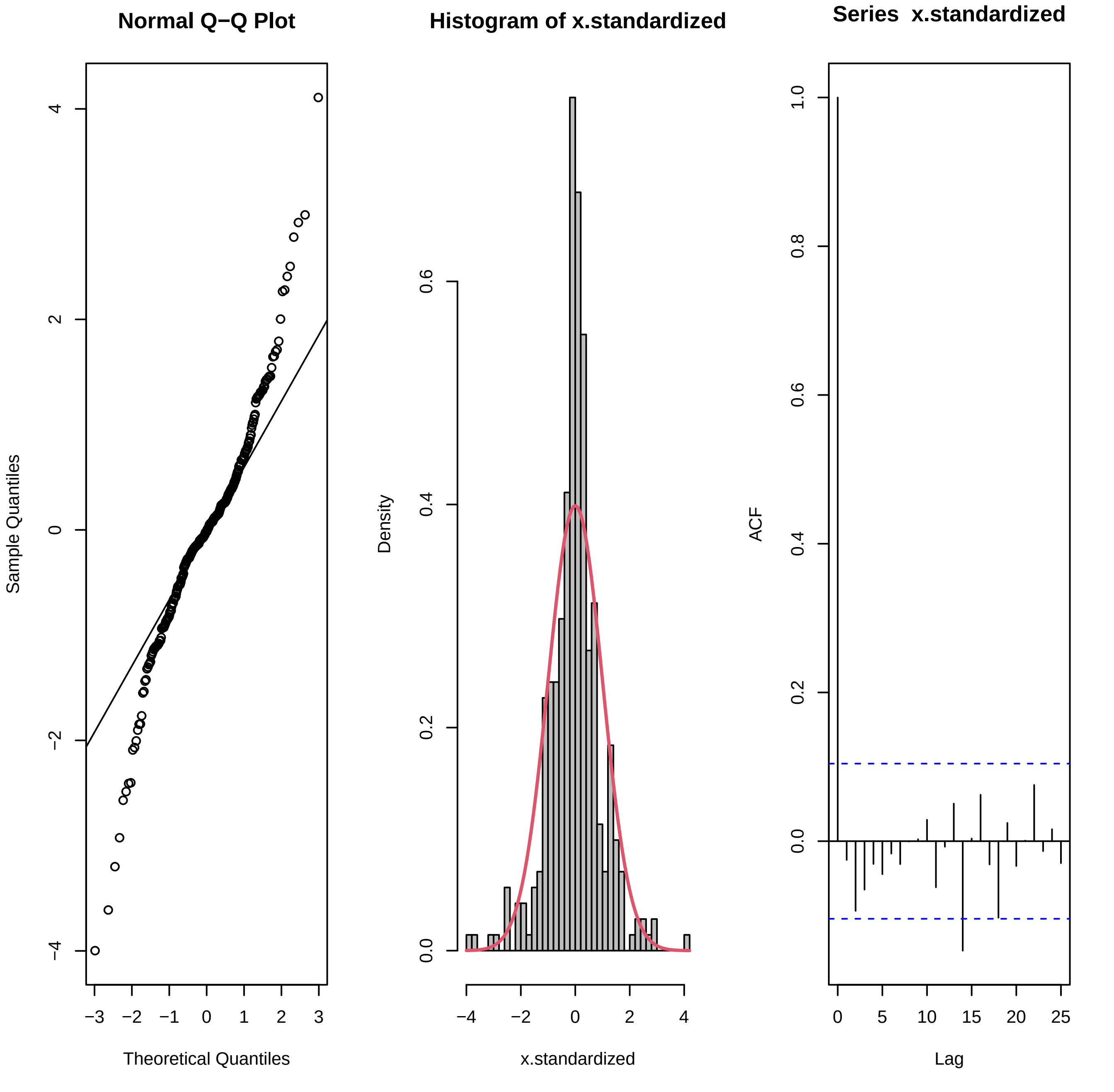

Normal Q−Q Plot
Sample Quantiles
Theoretical Quantiles
−4
−2
0
2
4
−3
−2
−1
0
1
2
3
Histogram of x.standardized
Density
x.standardized
0.0
0.2
0.4
0.6
−4
−2
0
2
4
Series x.standardized
ACF
Lag
0.0
0.2
0.4
0.6
0.8
1.0
0
5
10
15
20
25

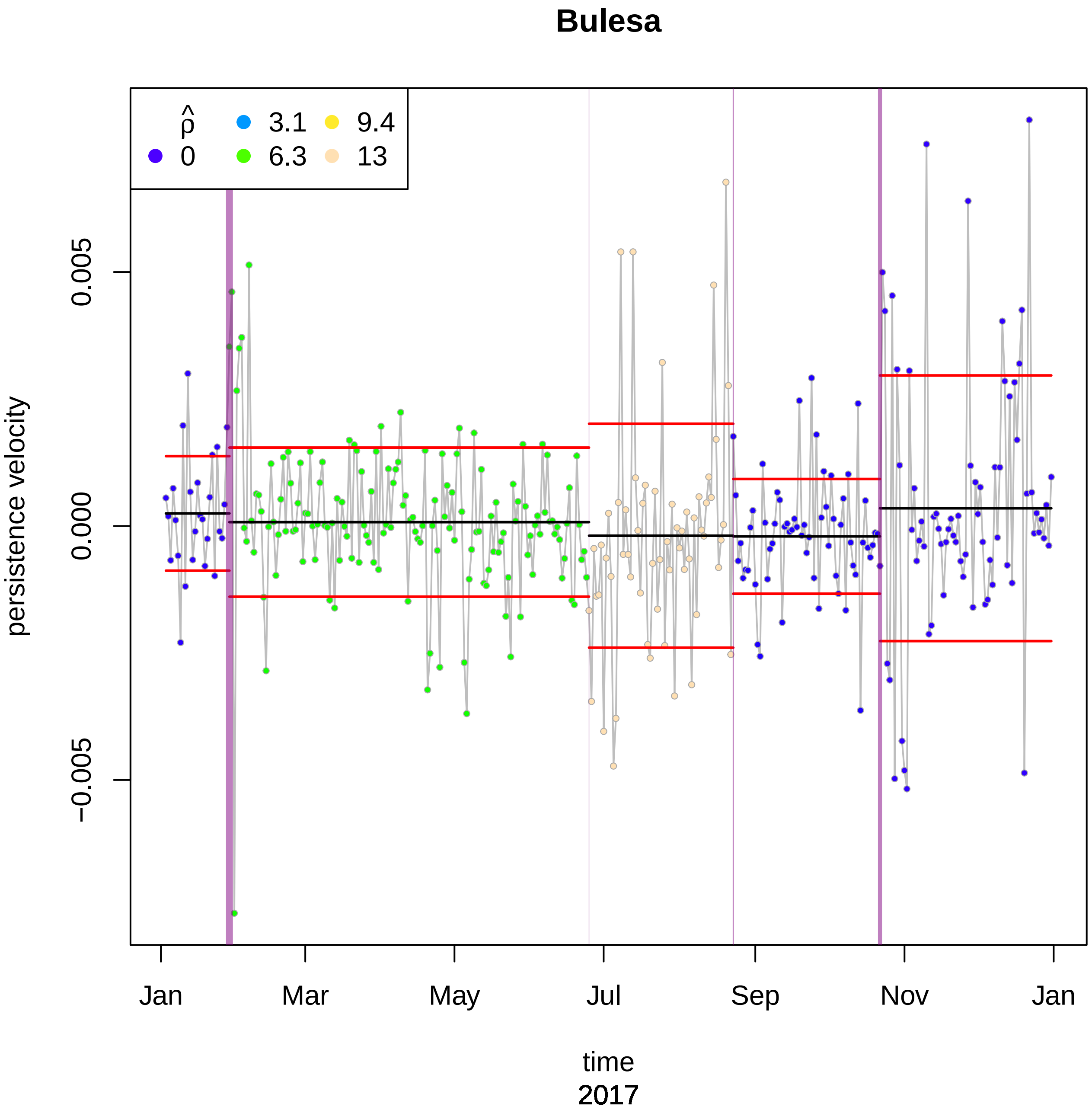
Bulesa
ρ̂
0
3.1
6.3
9.4
13
0.005
0.000
−0.005
persistence velocity
Jan
Mar
May
Jul
Sep
Nov
Jan
time
2017

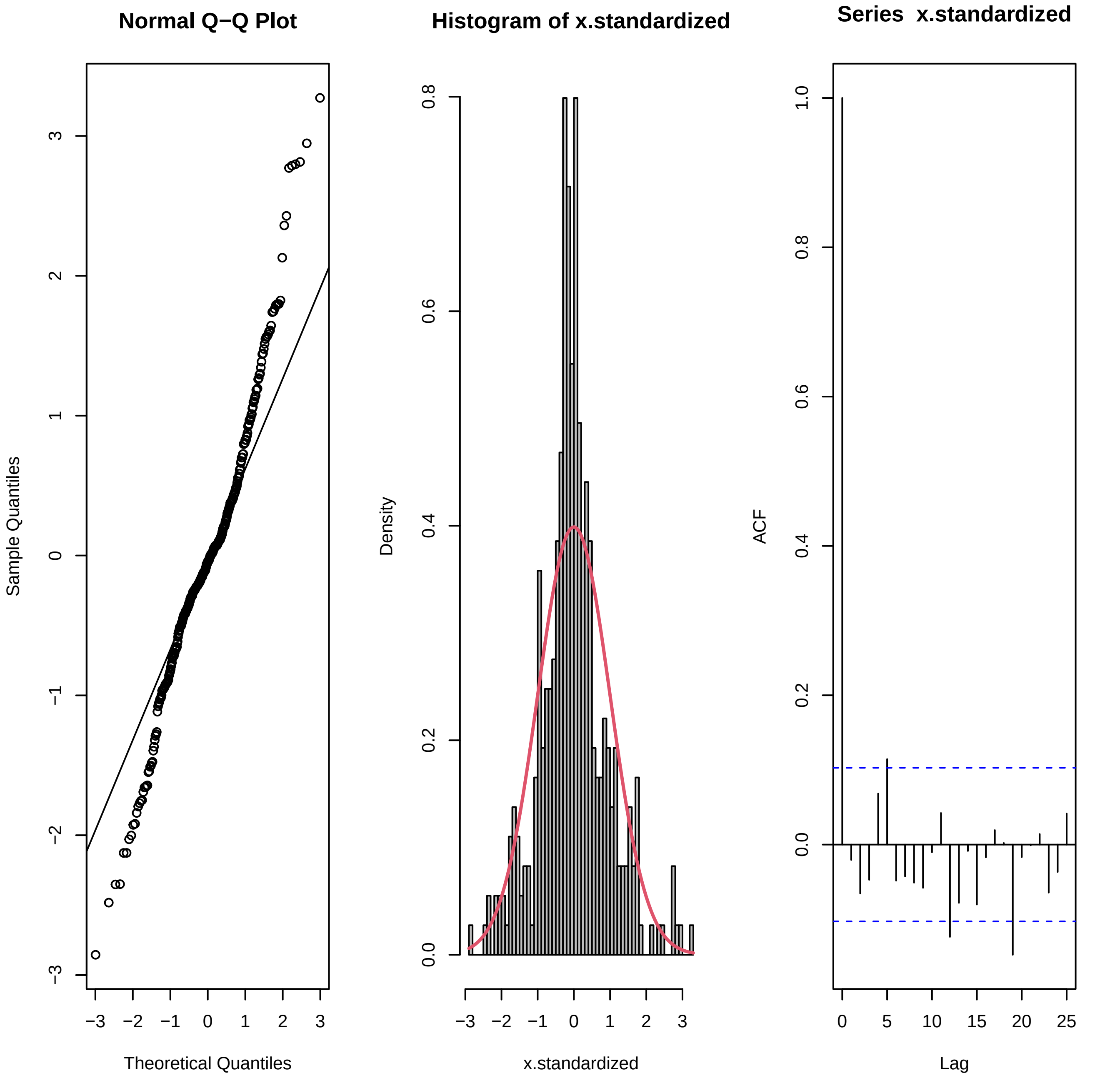
Normal Q−Q Plot
Histogram of x.standardized
Series x.standardized
Sample Quantiles
Theoretical Quantiles
Density
x.standardized
ACF
Lag

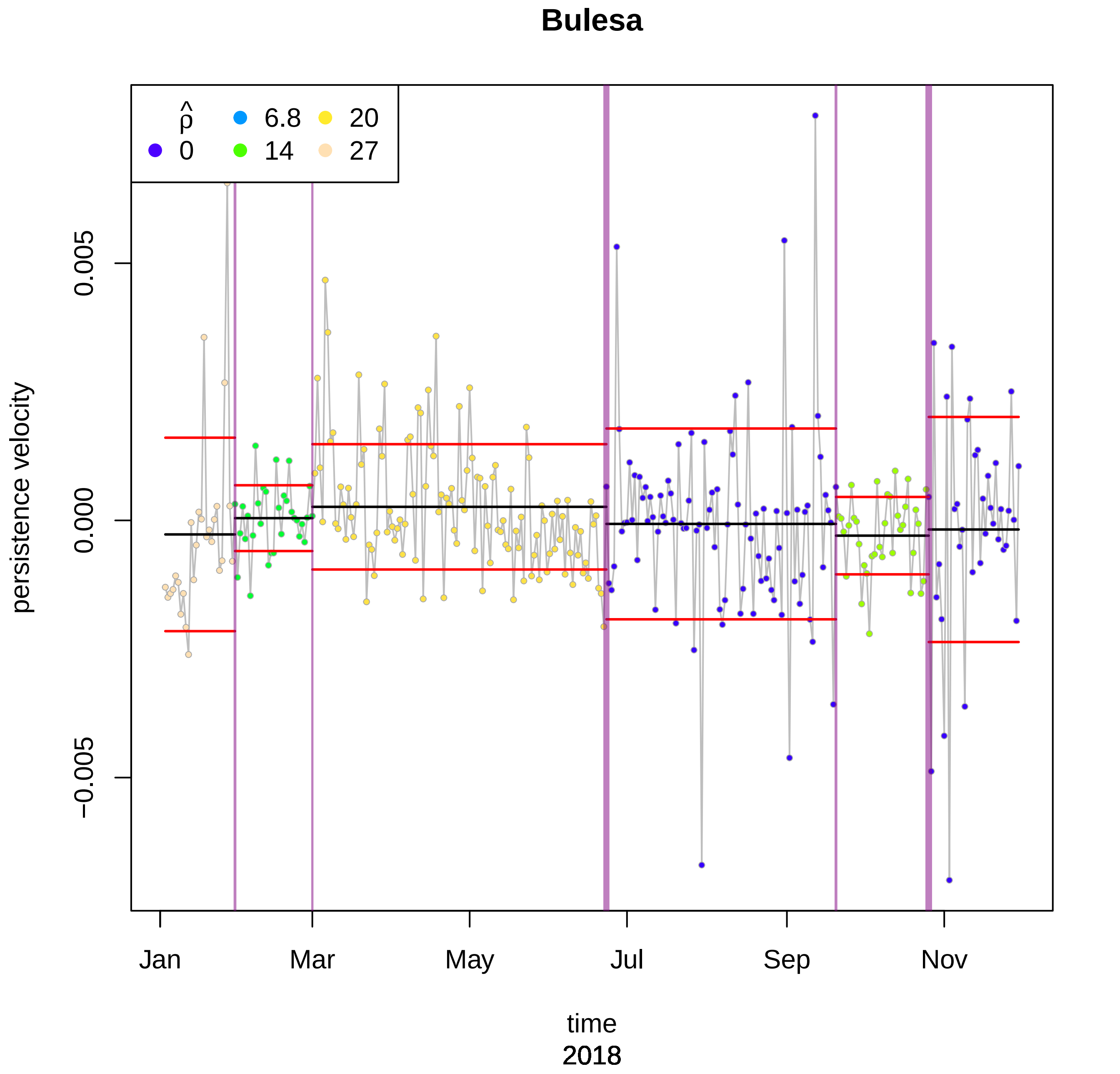

Bulesa
ρ̂
0
6.8
14
20
27
persistence velocity
0.005
0.000
−0.005
Jan
Mar
May
Jul
Sep
Nov
time
2018

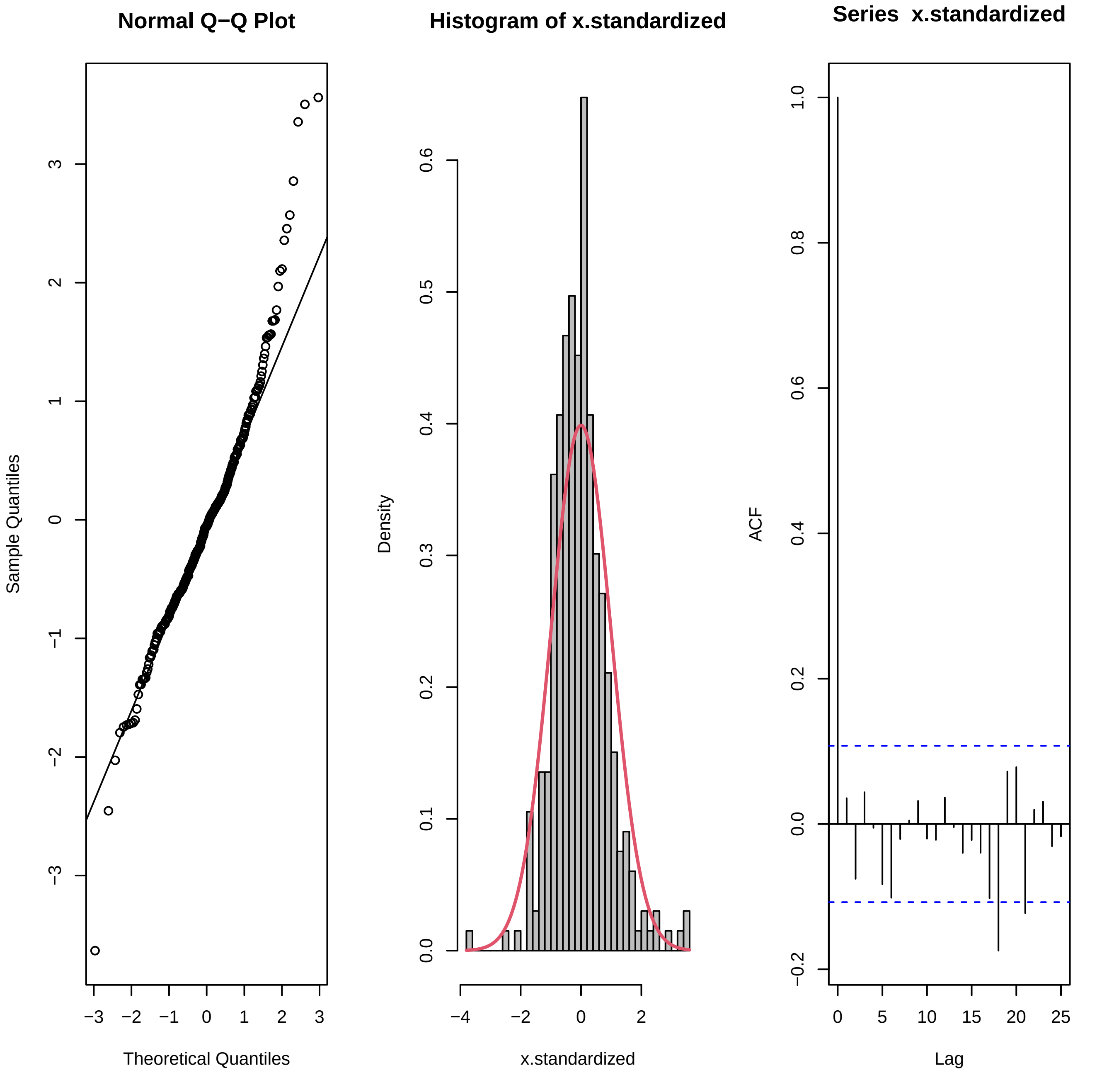
Normal Q−Q Plot
Sample Quantiles
Theoretical Quantiles
−3
−2
−1
0
1
2
3
Histogram of x.standardized
Density
x.standardized
0.0
0.1
0.2
0.3
0.4
0.5
0.6
−4
−2
0
2
Series x.standardized
ACF
Lag
−0.2
0.0
0.2
0.4
0.6
0.8
1.0
0
5
10
15
20
25

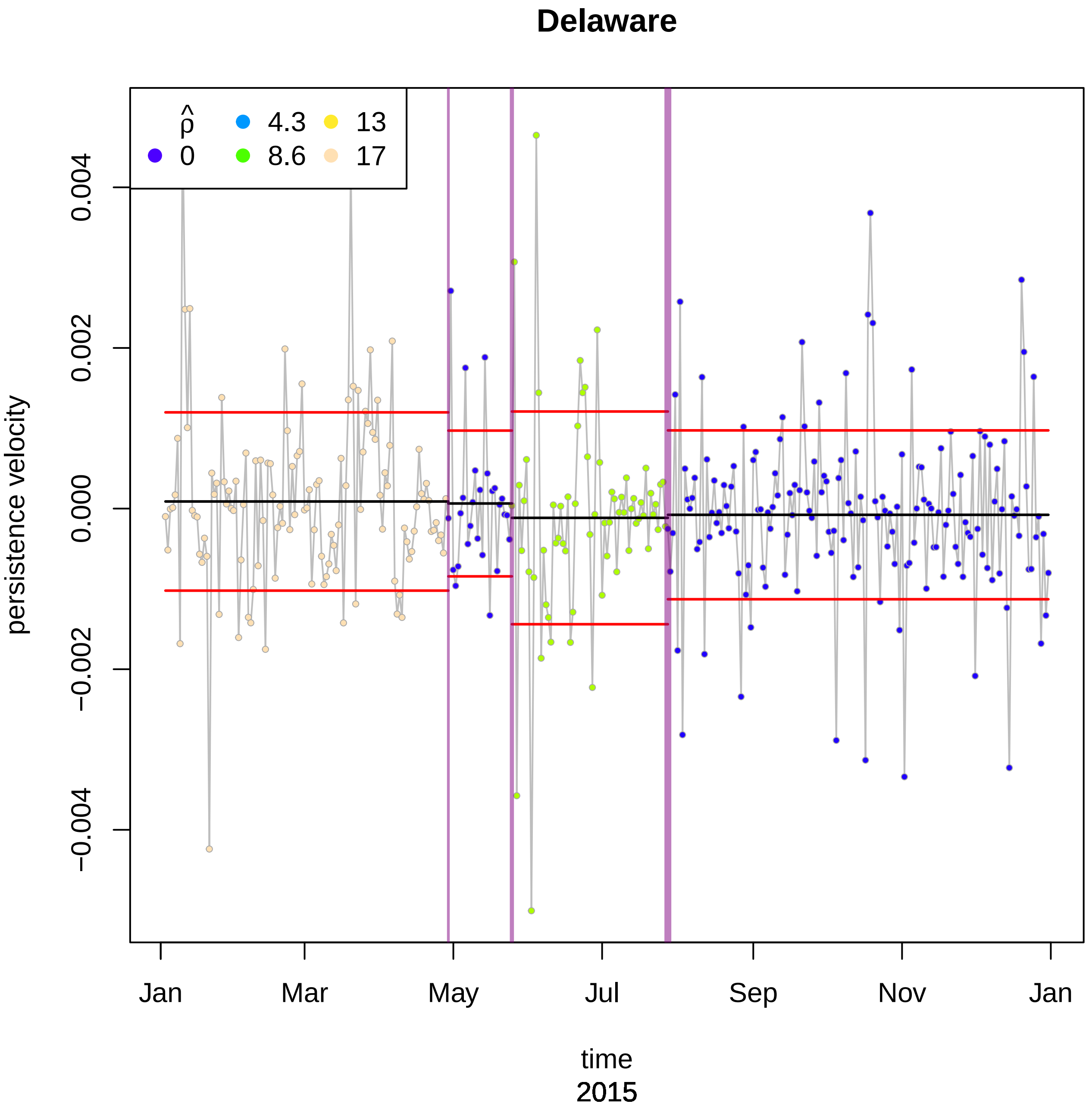

Delaware
ρ̂
0
4.3
8.6
13
17
persistence velocity
0.004
0.002
0.000
−0.002
−0.004
Jan
Mar
May
Jul
Sep
Nov
Jan
time
2015

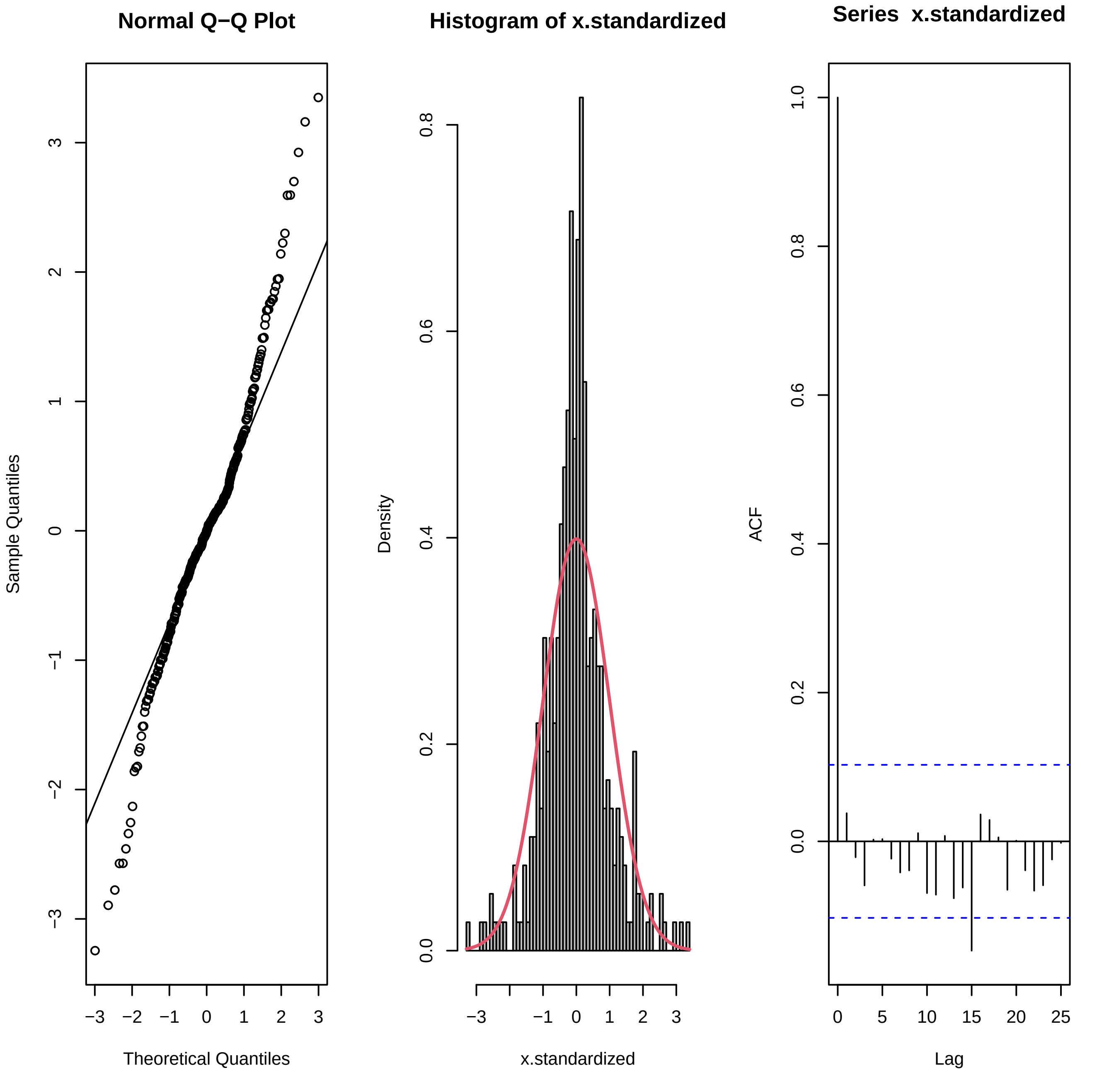

Normal Q−Q Plot
Sample Quantiles
Theoretical Quantiles
Histogram of x.standardized
Density
x.standardized
Series x.standardized
ACF
Lag

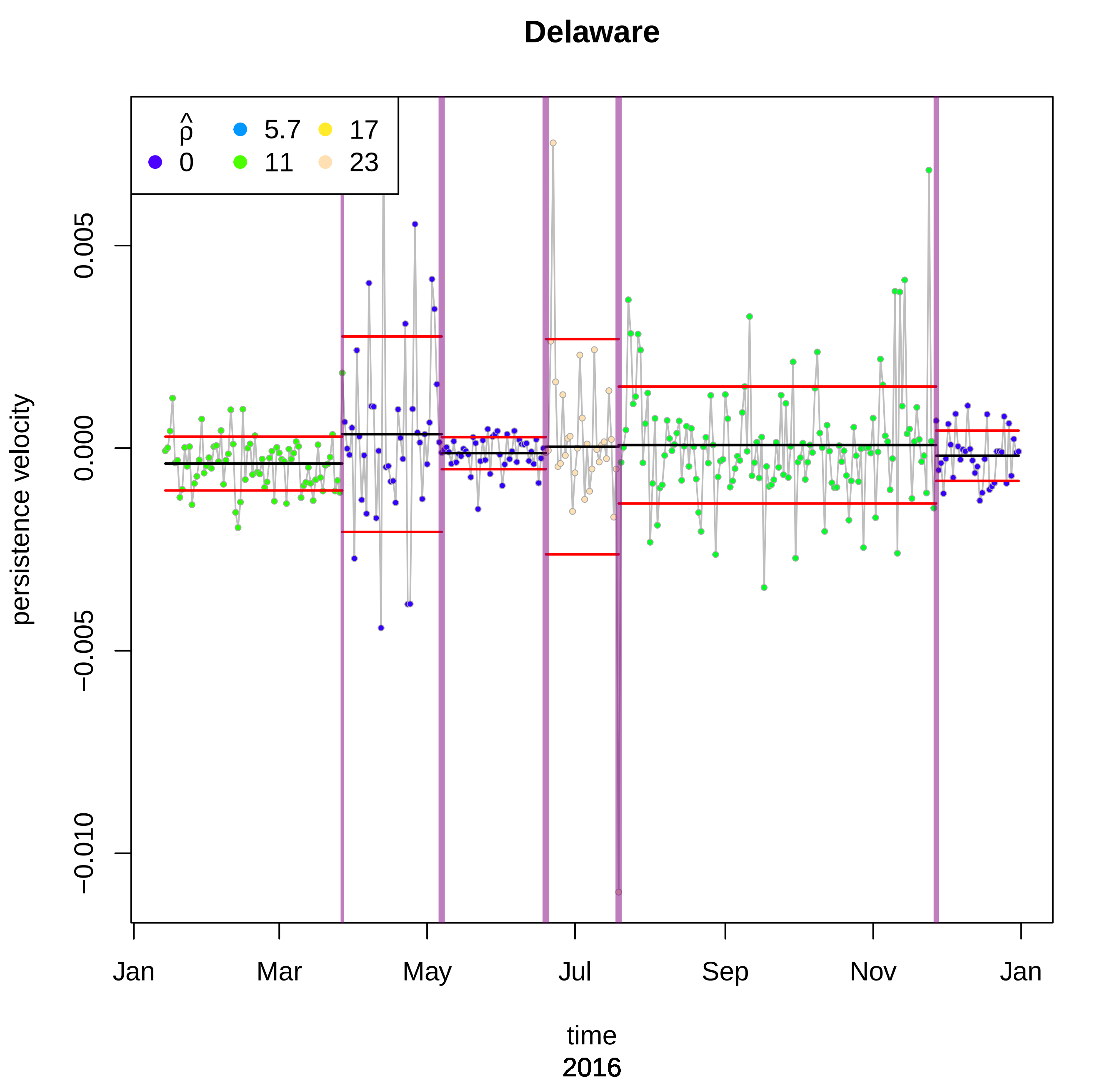

Delaware
ρ̂
0
5.7
11
17
23
persistence velocity
0.005
0.000
−0.005
−0.010
Jan
Mar
May
Jul
Sep
Nov
Jan
time
2016

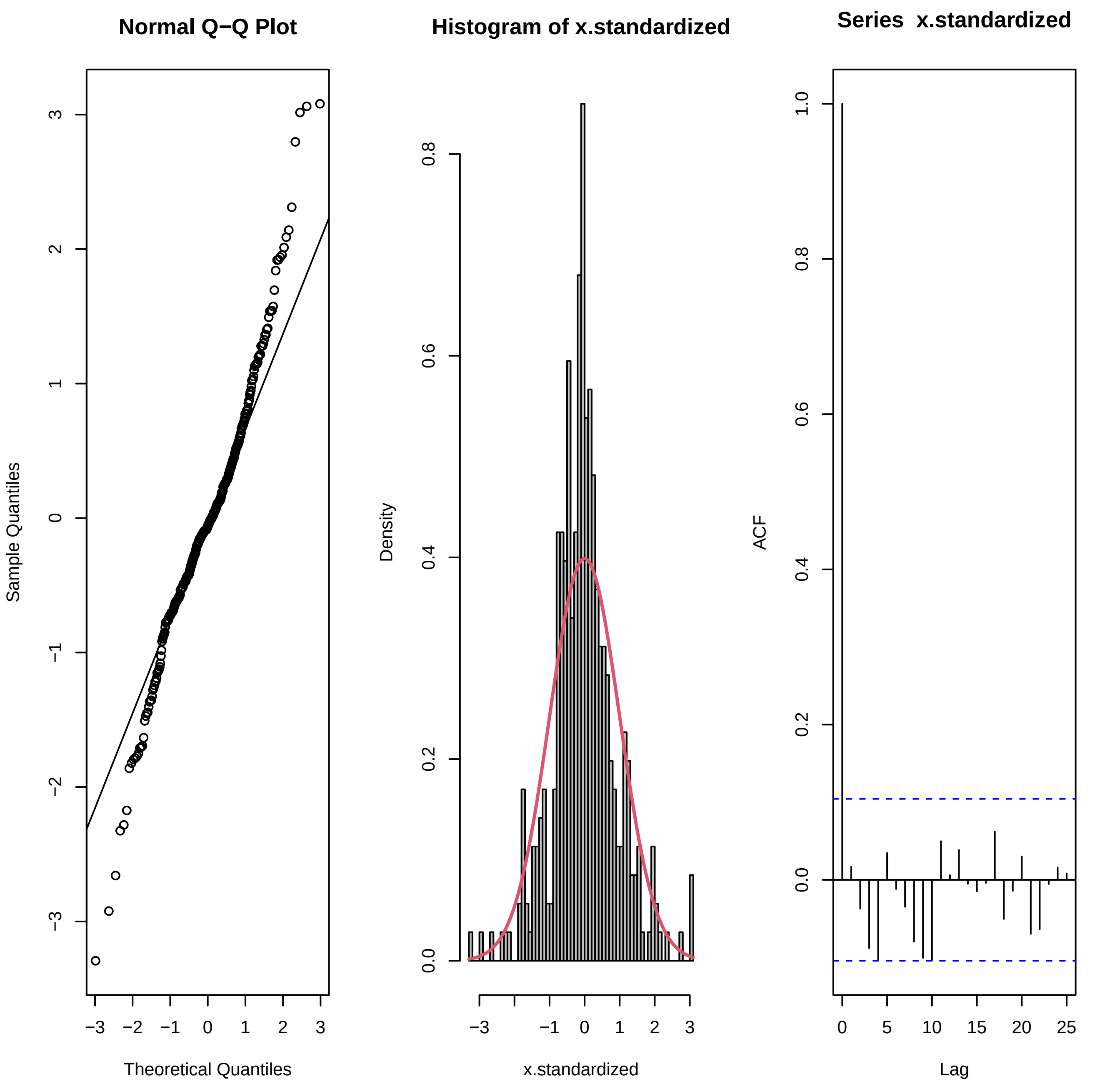

Normal Q−Q Plot
Sample Quantiles
Theoretical Quantiles
−3
−2
−1
0
1
2
3
Histogram of x.standardized
Density
x.standardized
0.0
0.2
0.4
0.6
0.8
Series x.standardized
ACF
Lag
0.0
0.2
0.4
0.6
0.8
1.0
0
5
10
15
20
25

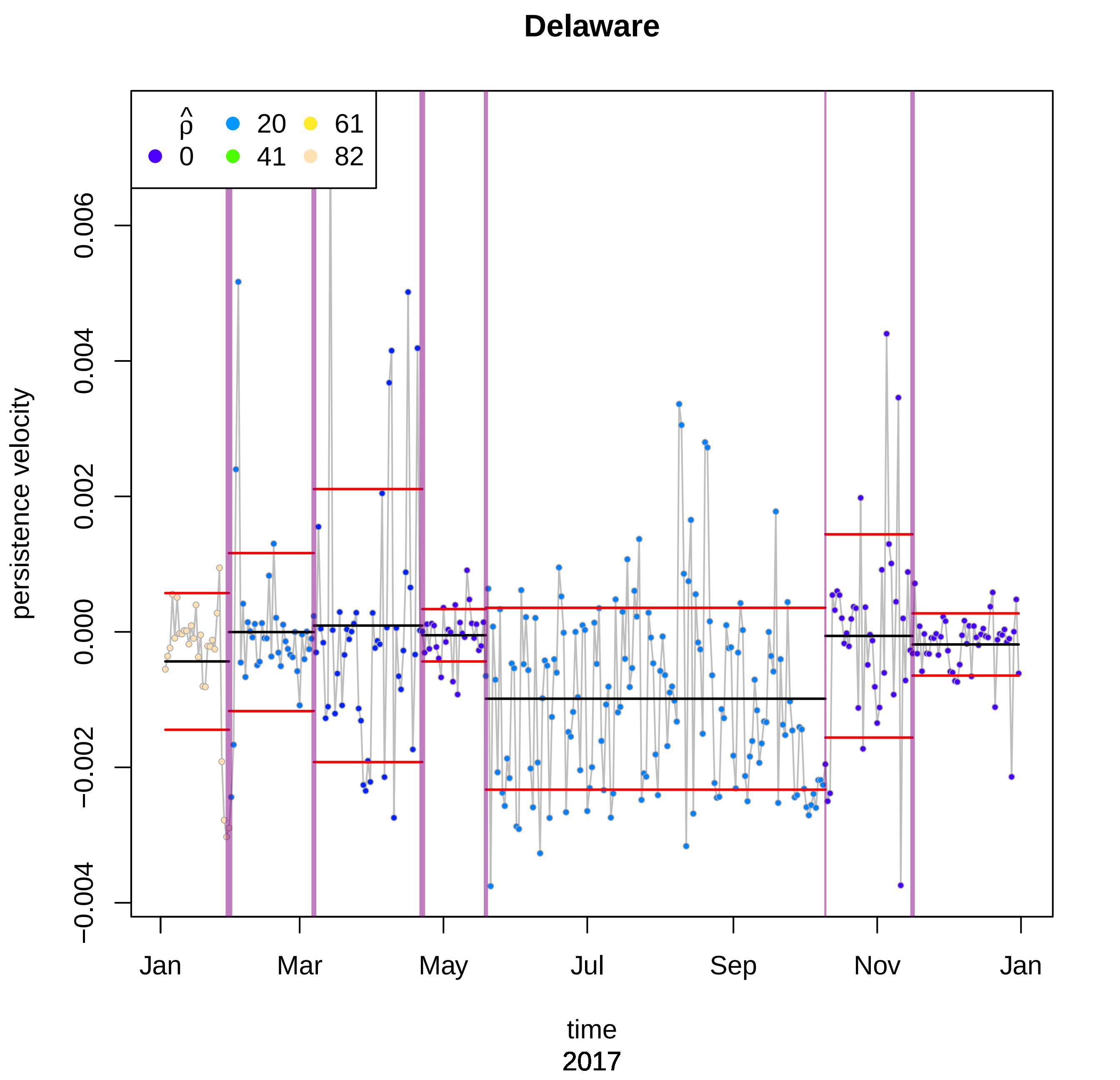

Delaware
ρ̂
0
20
41
61
82
persistence velocity
0.006
0.004
0.002
0.000
−0.002
−0.004
Jan
Mar
May
Jul
Sep
Nov
Jan
time
2017

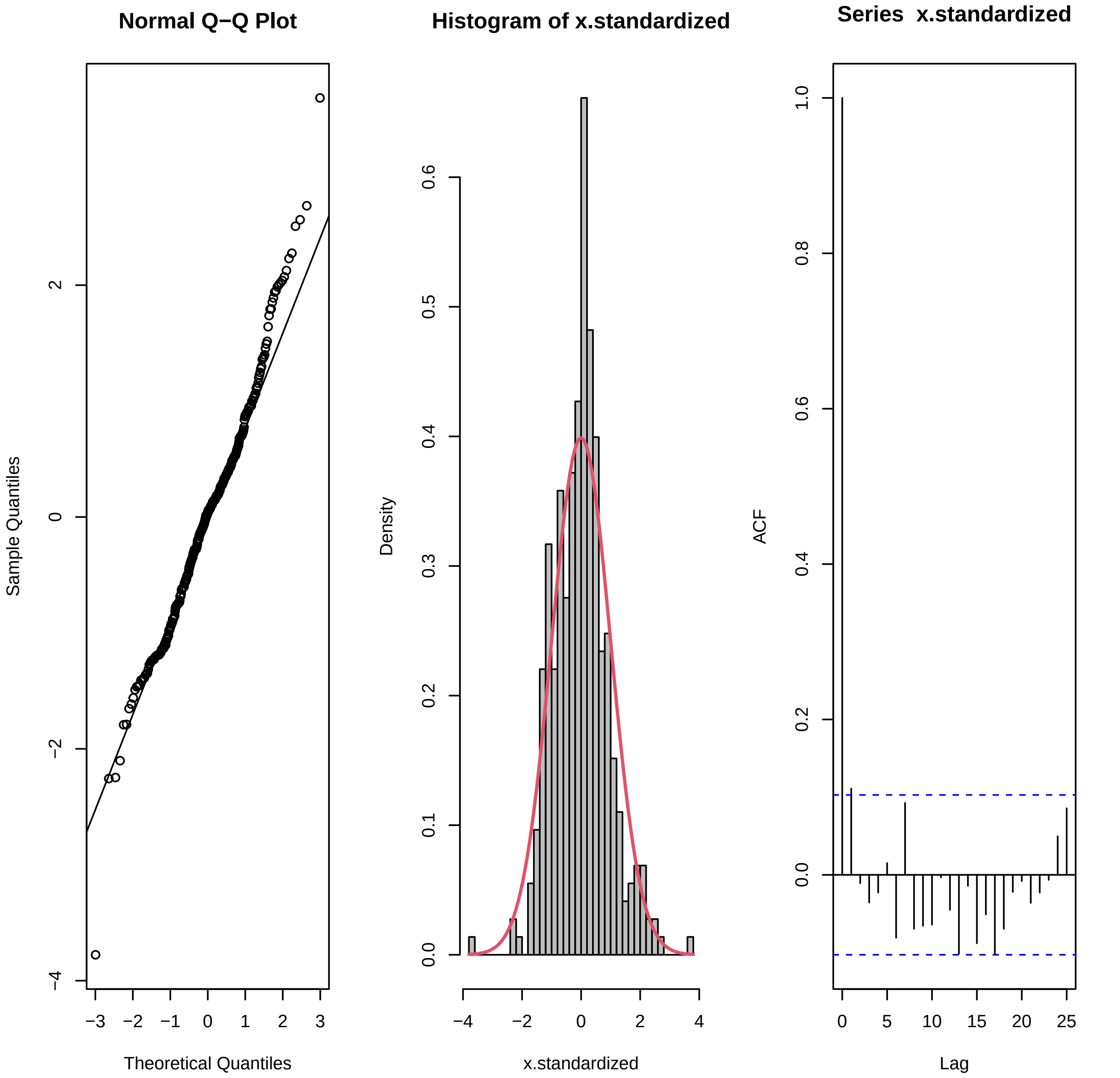

Normal Q−Q Plot
Sample Quantiles
Theoretical Quantiles
−4
−2
0
2
−3 −2 −1 0 1 2 3
Histogram of x.standardized
Density
x.standardized
0.0 0.1 0.2 0.3 0.4 0.5 0.6
−4 −2 0 2 4
Series x.standardized
ACF
Lag
0.0 0.2 0.4 0.6 0.8 1.0
0 5 10 15 20 25

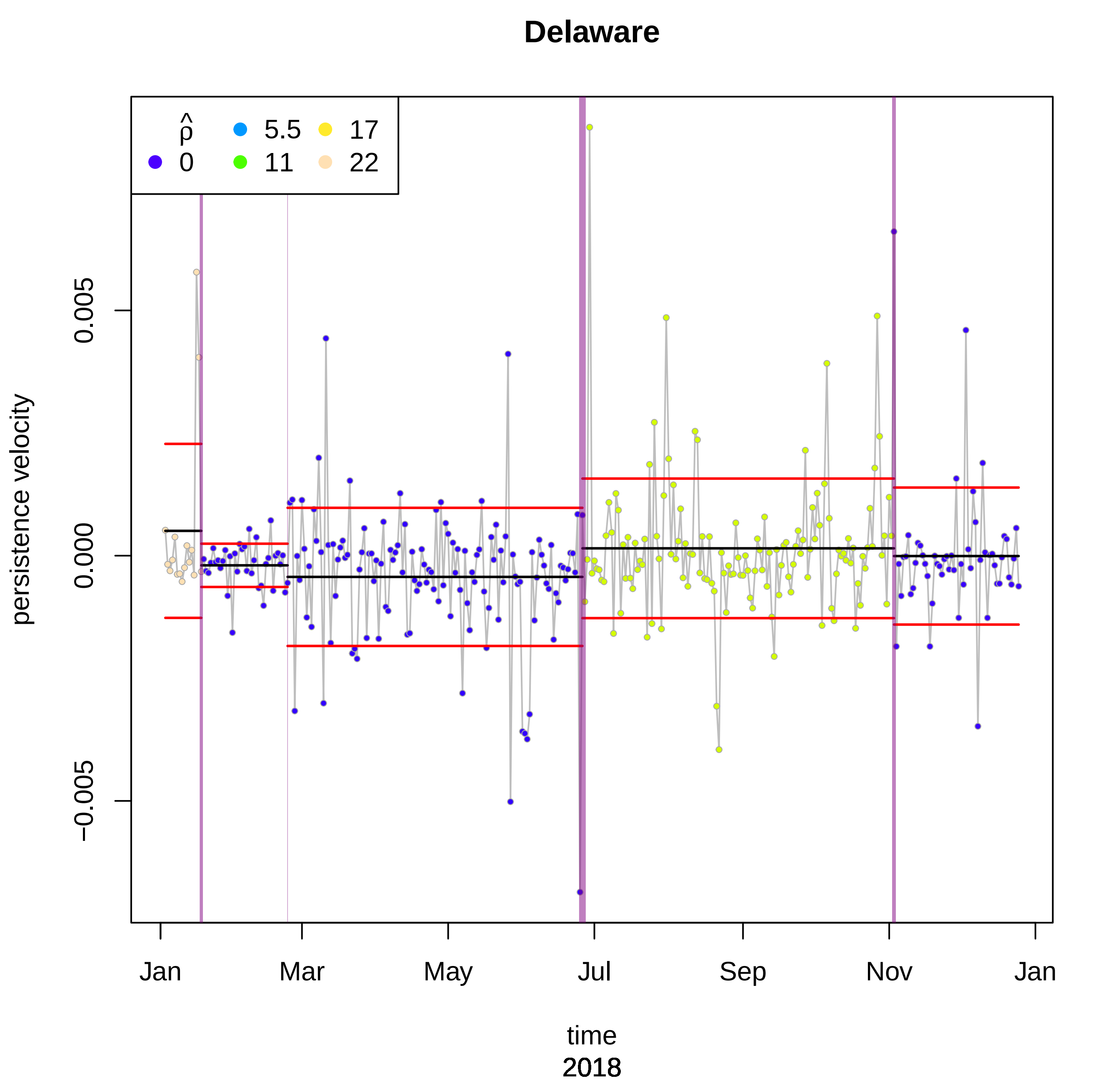

Delaware
ρ̂
0
5.5
11
17
22
persistence velocity
0.005
0.000
−0.005
Jan
Mar
May
Jul
Sep
Nov
Jan
time
2018

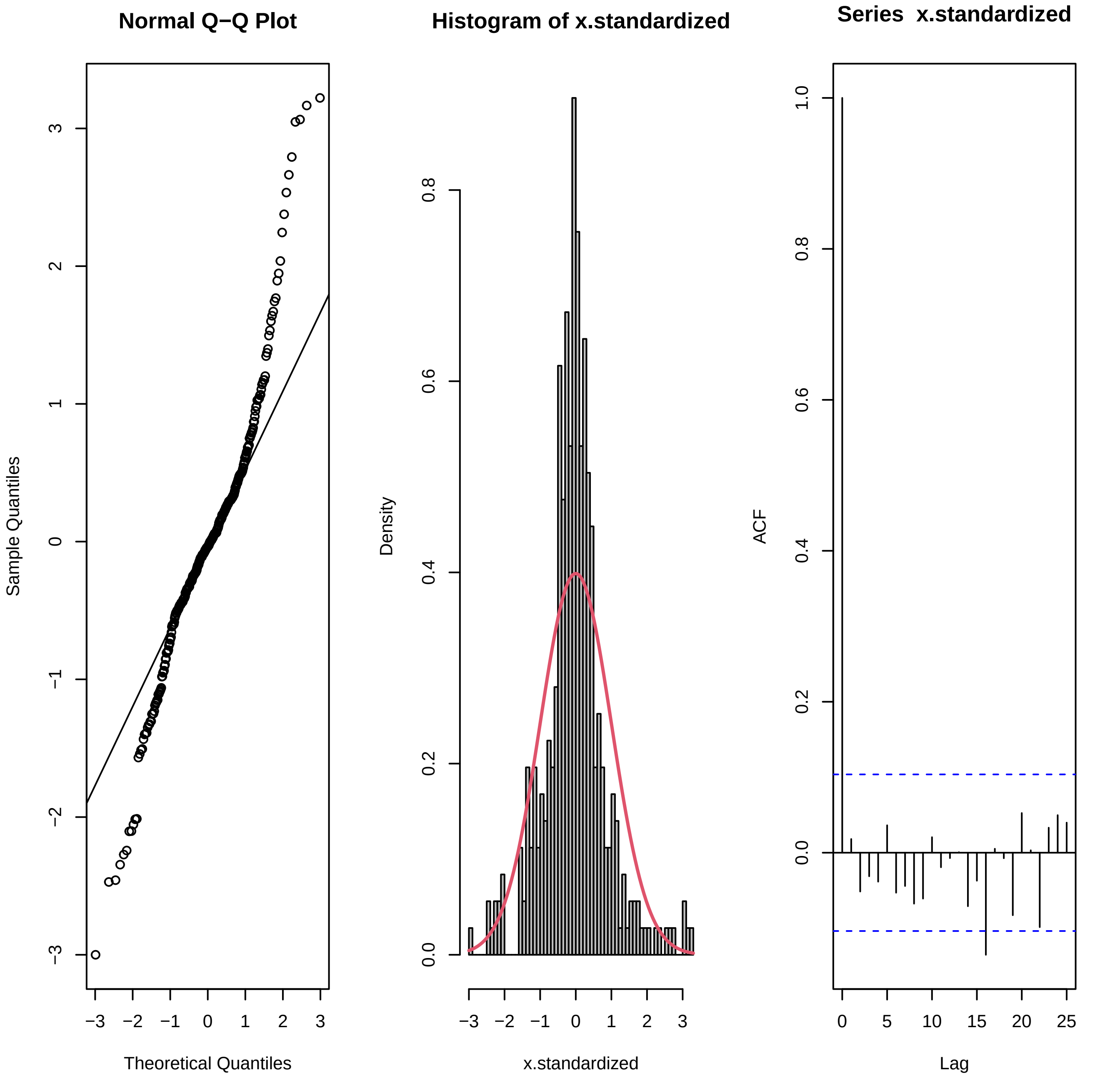

Normal Q−Q Plot
Sample Quantiles
Theoretical Quantiles
Histogram of x.standardized
Density
x.standardized
Series x.standardized
ACF
Lag

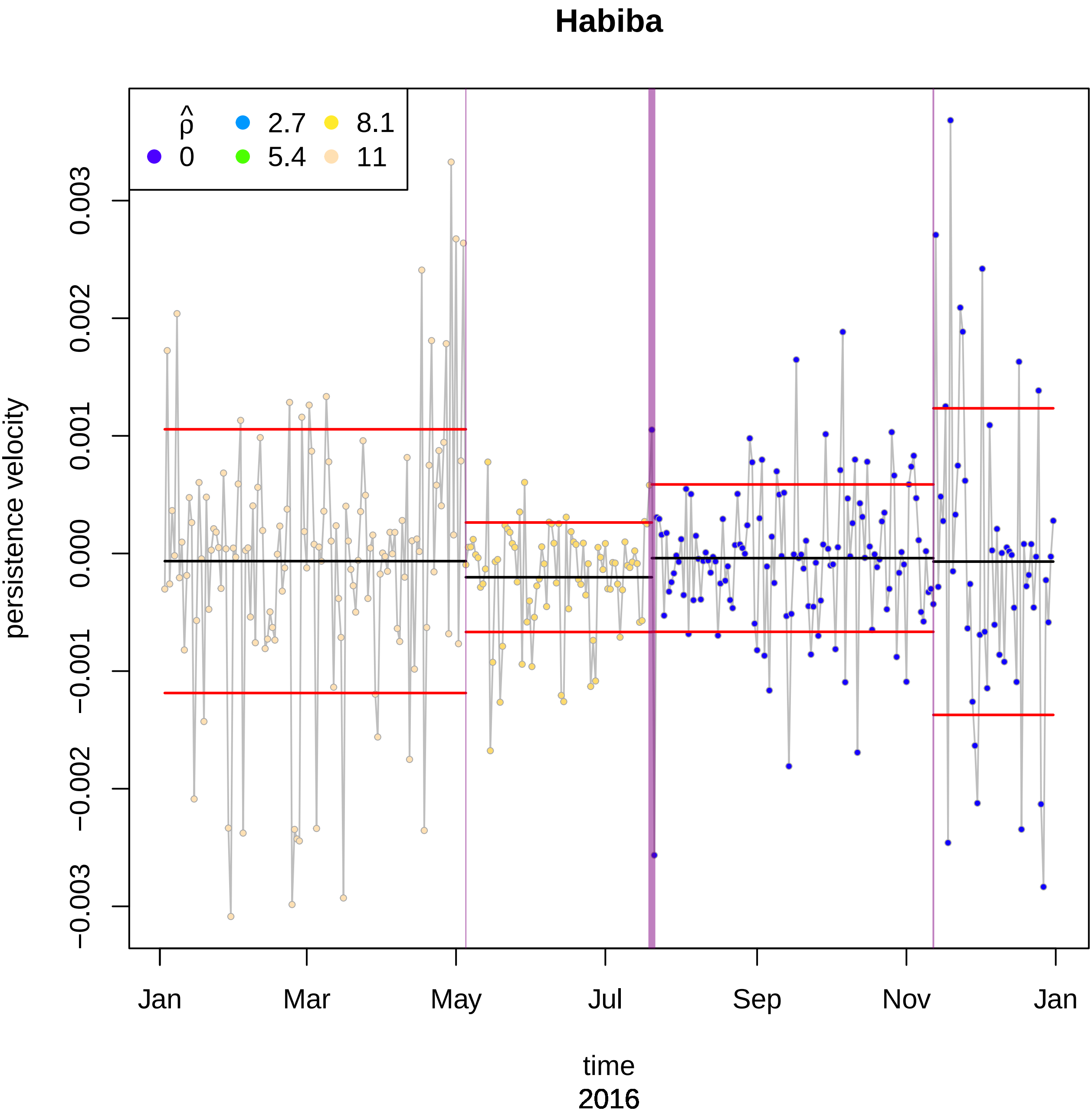
Habiba
ρ̂
0
2.7
5.4
8.1
11
persistence velocity
0.003
0.002
0.001
0.000
−0.001
−0.002
−0.003
Jan
Mar
May
Jul
Sep
Nov
Jan
time
2016

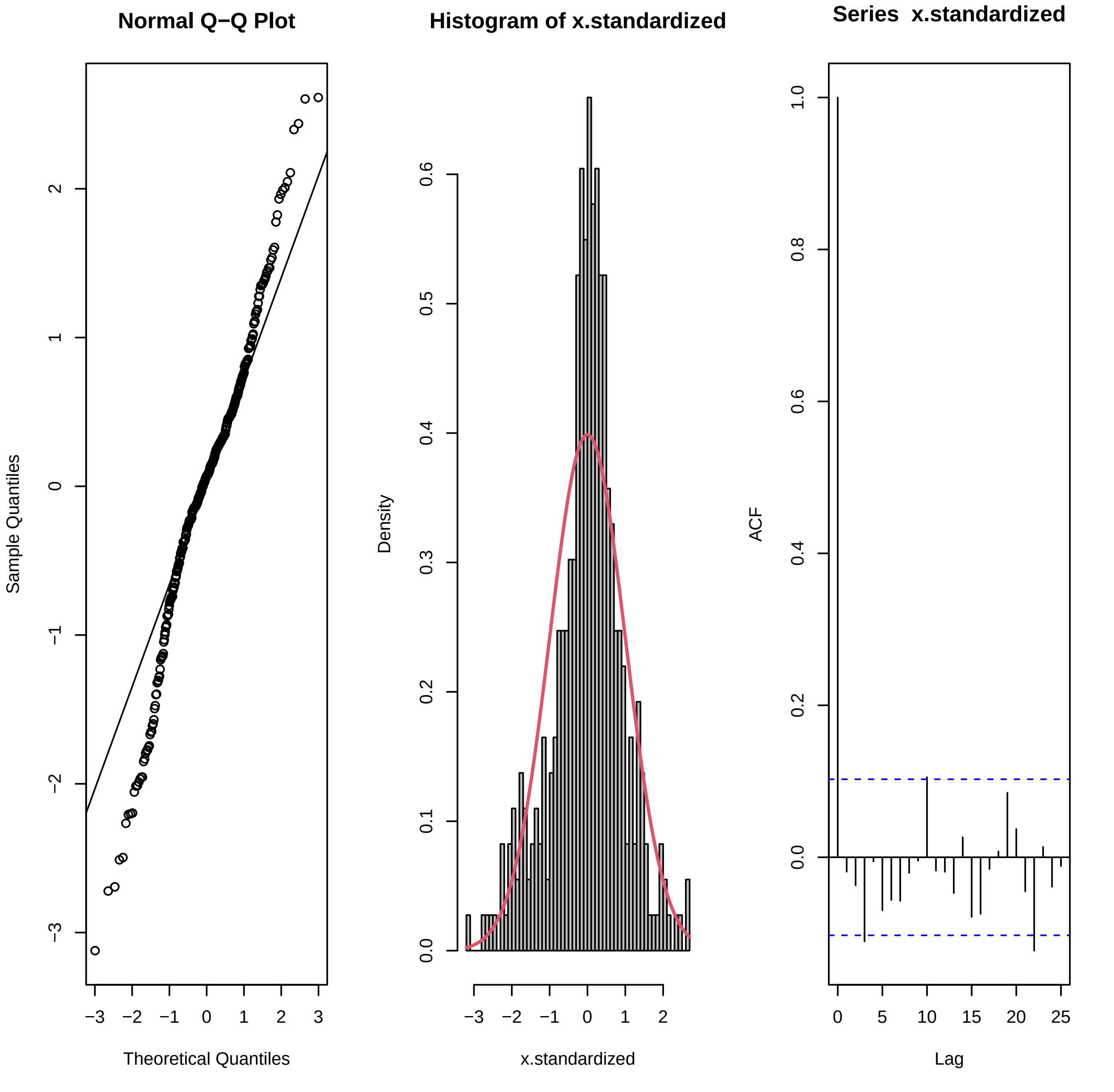

Normal Q−Q Plot
Sample Quantiles
Theoretical Quantiles
−3
−2
−1
0
1
2
3
Histogram of x.standardized
Density
x.standardized
0.0
0.1
0.2
0.3
0.4
0.5
0.6
Series x.standardized
ACF
Lag
0.0
0.2
0.4
0.6
0.8
1.0
0
5
10
15
20
25

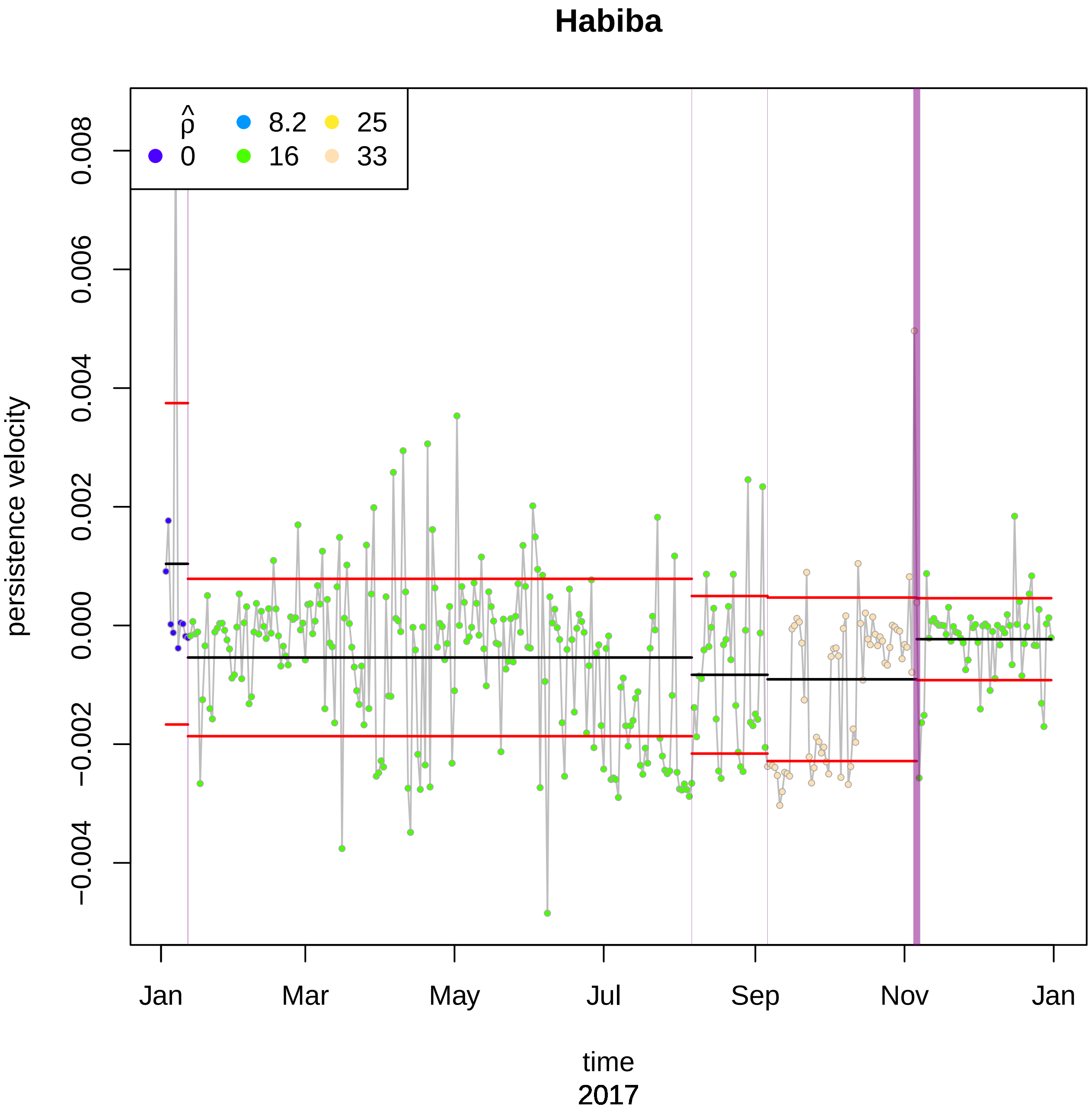

Habiba
ρ̂
0
8.2
16
25
33
persistence velocity
0.008
0.006
0.004
0.002
0.000
−0.002
−0.004
Jan
Mar
May
Jul
Sep
Nov
Jan
time
2017

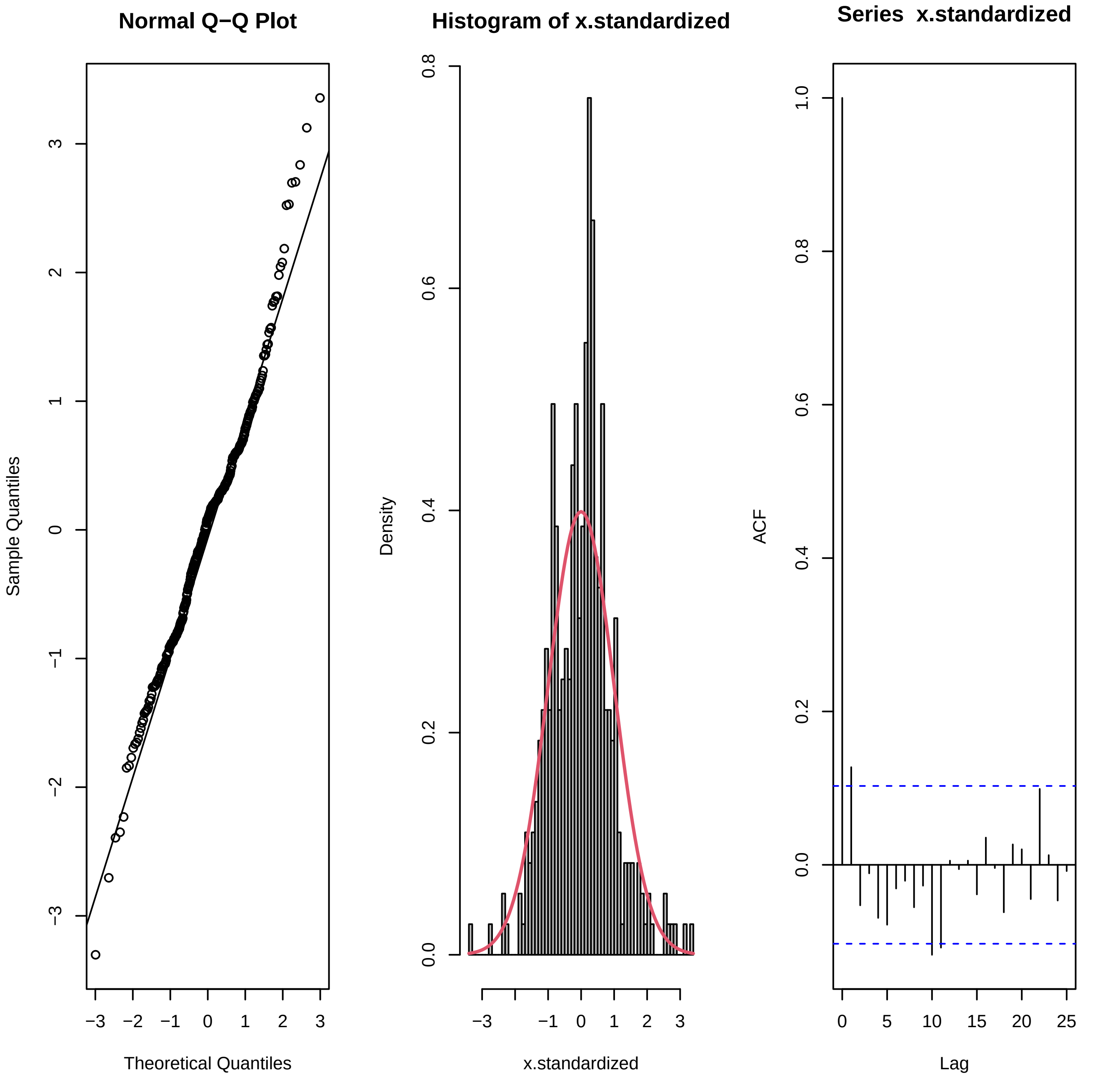

Normal Q−Q Plot
Sample Quantiles
Theoretical Quantiles
Histogram of x.standardized
Density
x.standardized
Series x.standardized
ACF
Lag

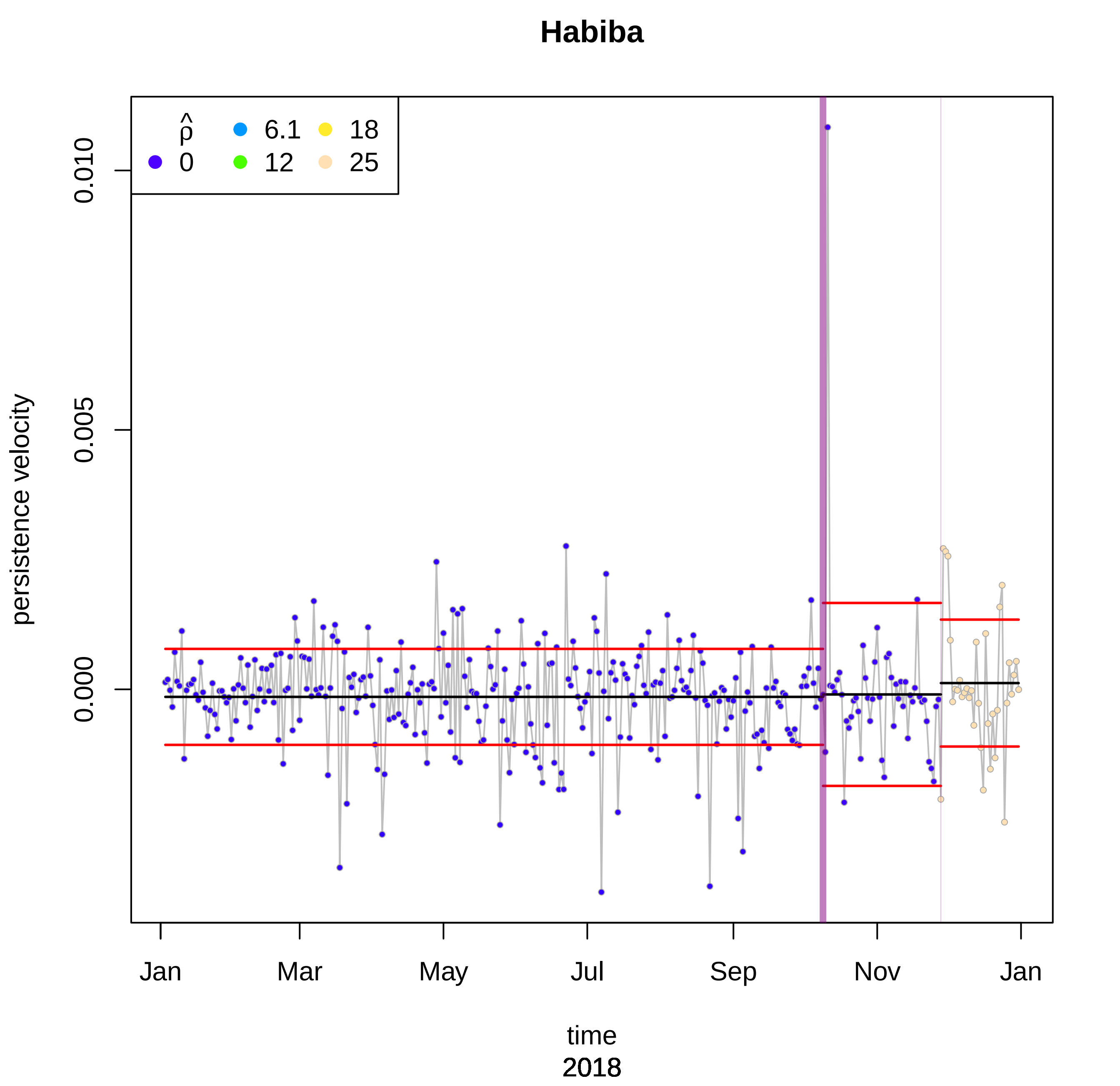
Habiba
ρ̂
0
6.1
12
18
25
persistence velocity
0.000
0.005
0.010
Jan
Mar
May
Jul
Sep
Nov
Jan
time
2018

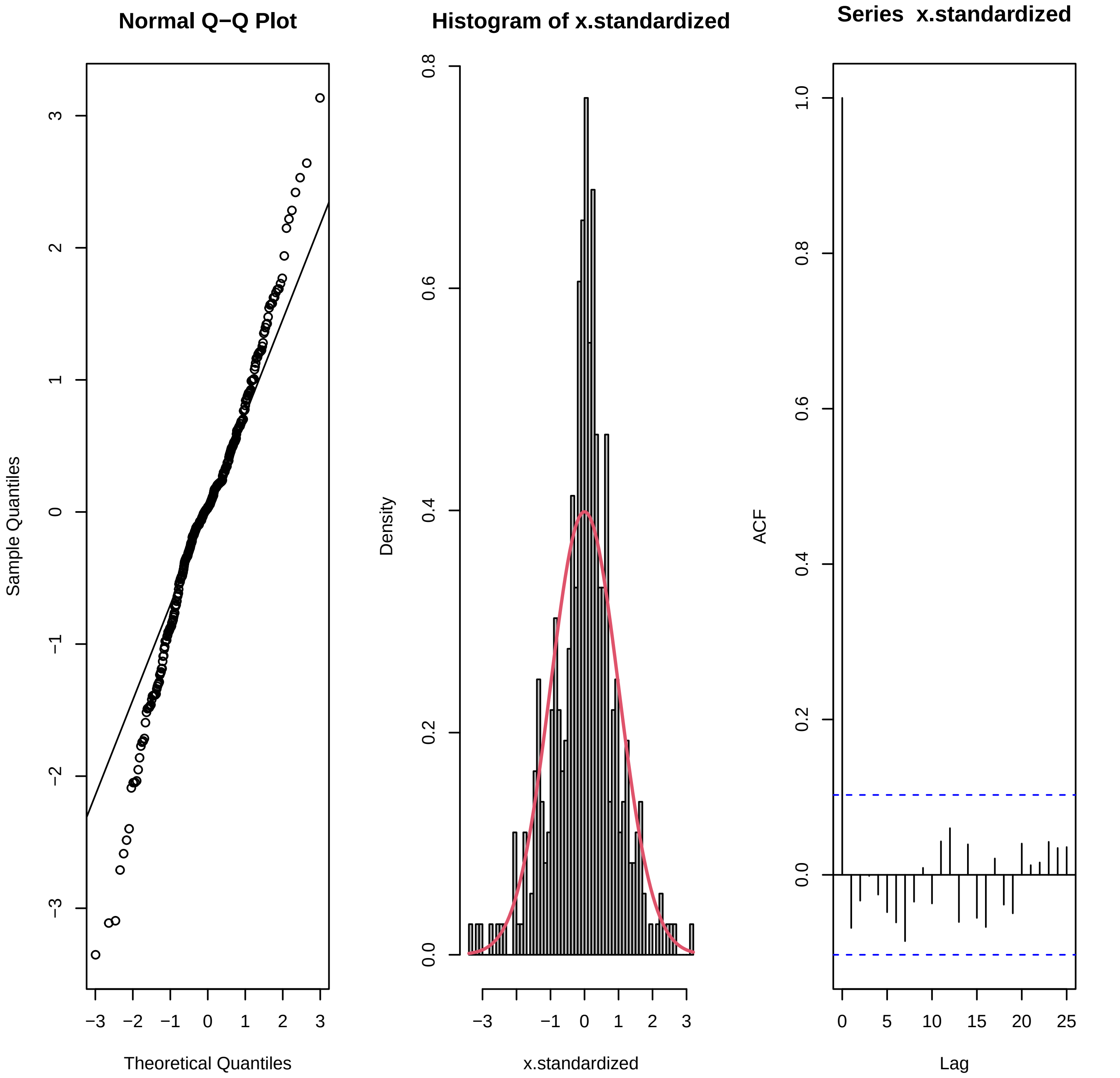
Normal Q−Q Plot
Sample Quantiles
Theoretical Quantiles
Histogram of x.standardized
Density
x.standardized
Series x.standardized
ACF
Lag

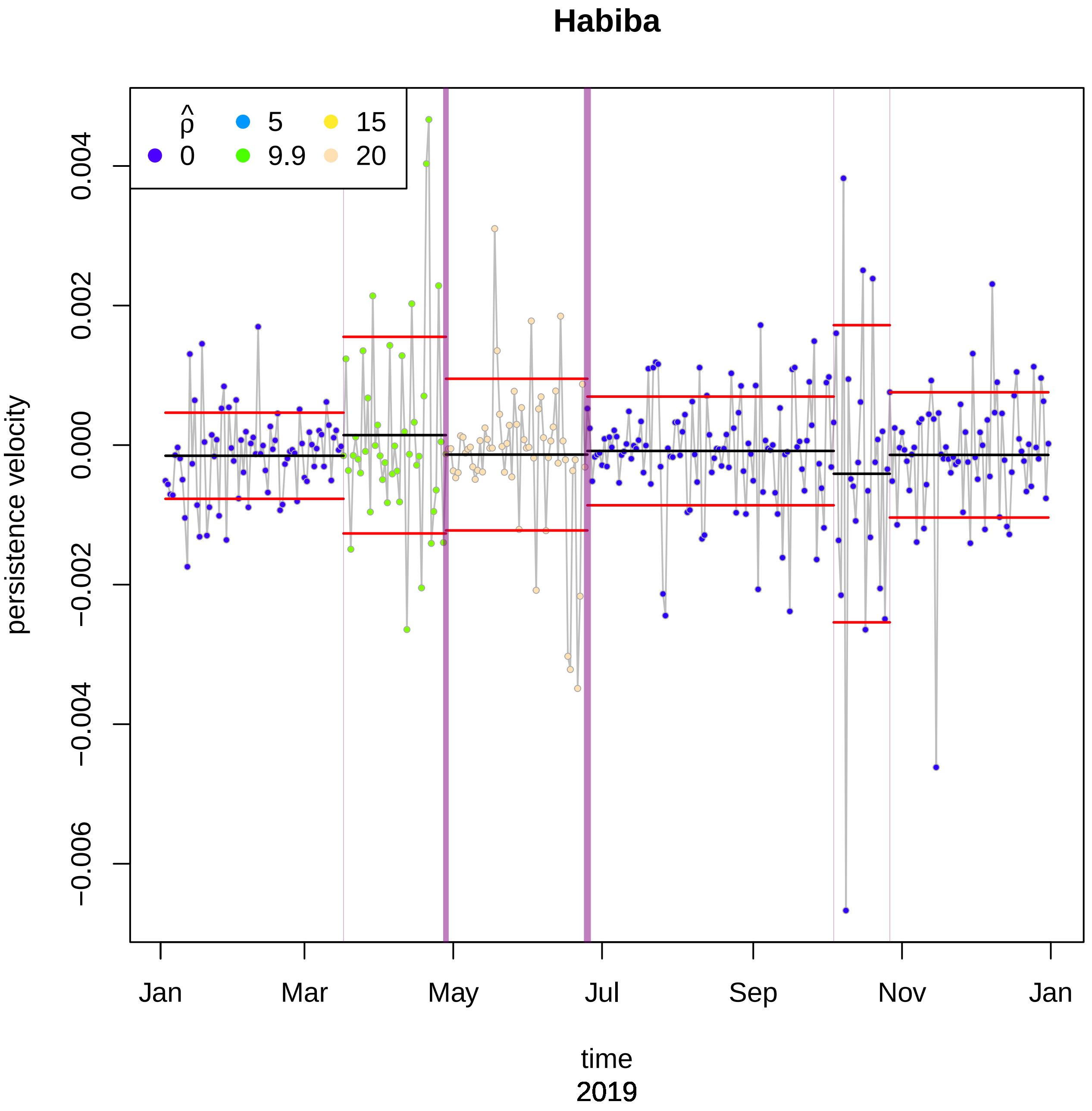

Habiba
ρ̂
0
5
9.9
15
20
0.004
0.002
0.000
−0.002
−0.004
−0.006
persistence velocity
Jan
Mar
May
Jul
Sep
Nov
Jan
time
2019

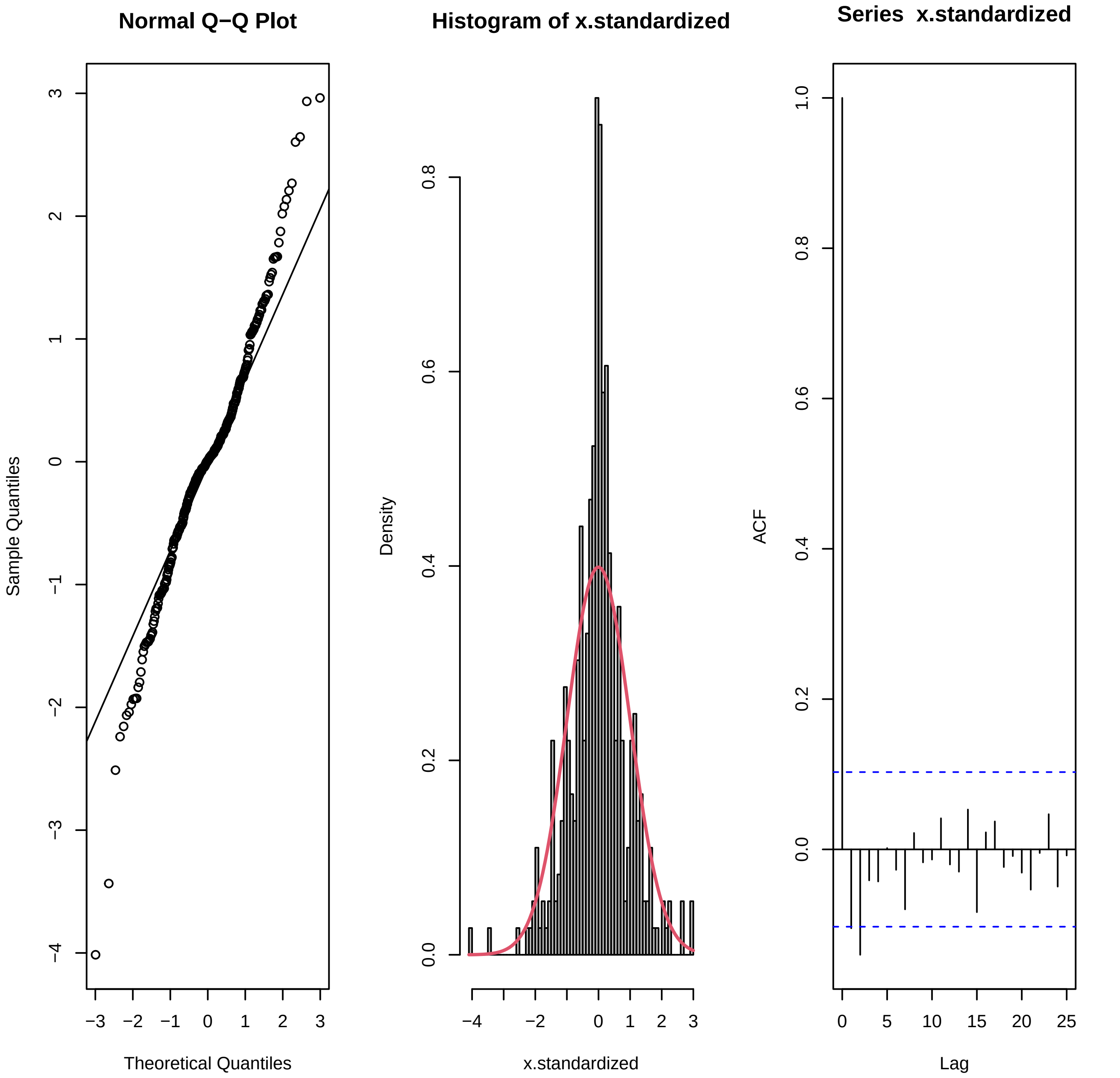

Normal Q−Q Plot
Sample Quantiles
Theoretical Quantiles
Histogram of x.standardized
Density
x.standardized
Series x.standardized
ACF
Lag

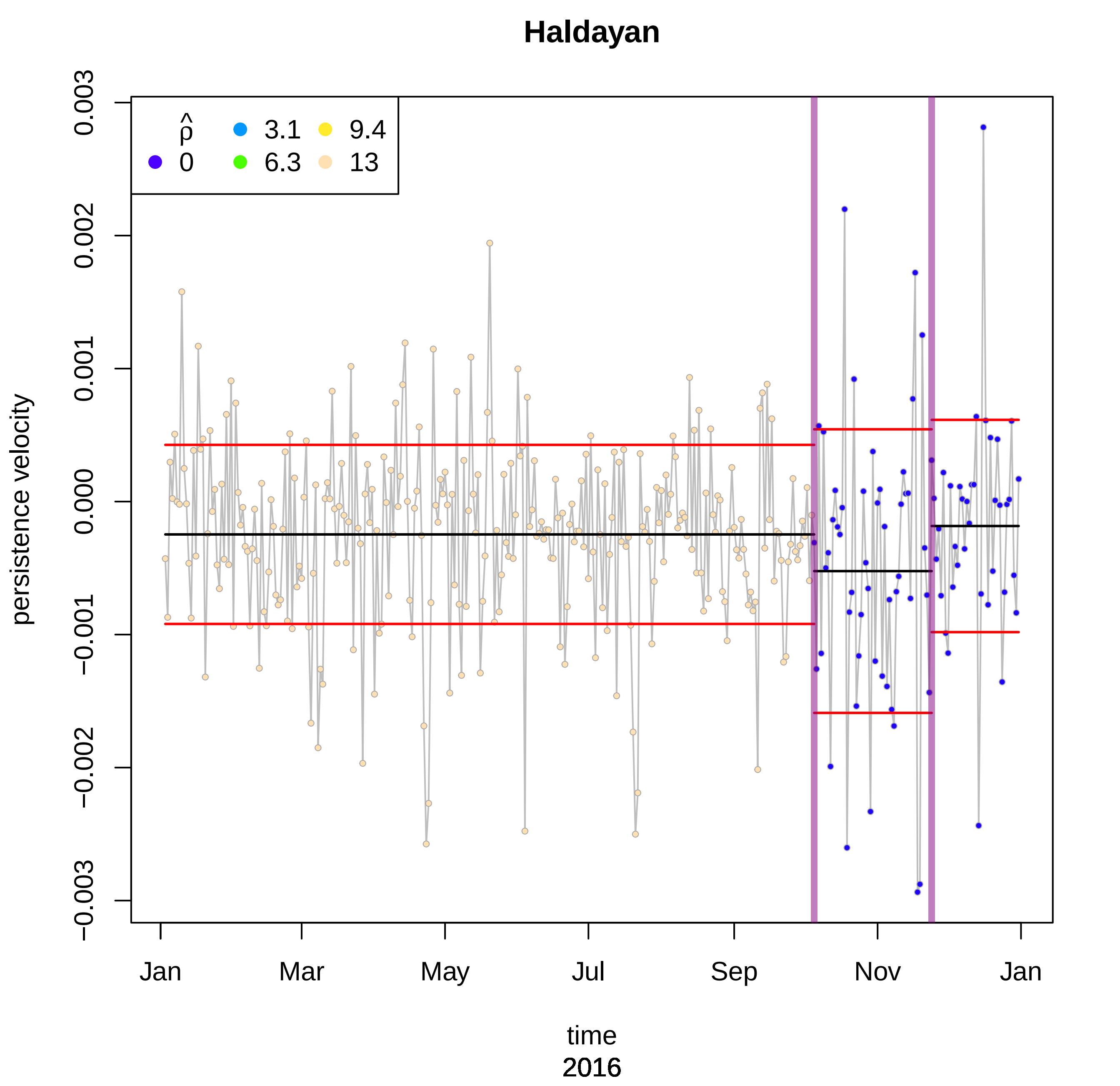

Haldayan
ρ̂
0
3.1
6.3
9.4
13
persistence velocity
0.003
0.002
0.001
0.000
−0.001
−0.002
−0.003
Jan
Mar
May
Jul
Sep
Nov
Jan
time
2016

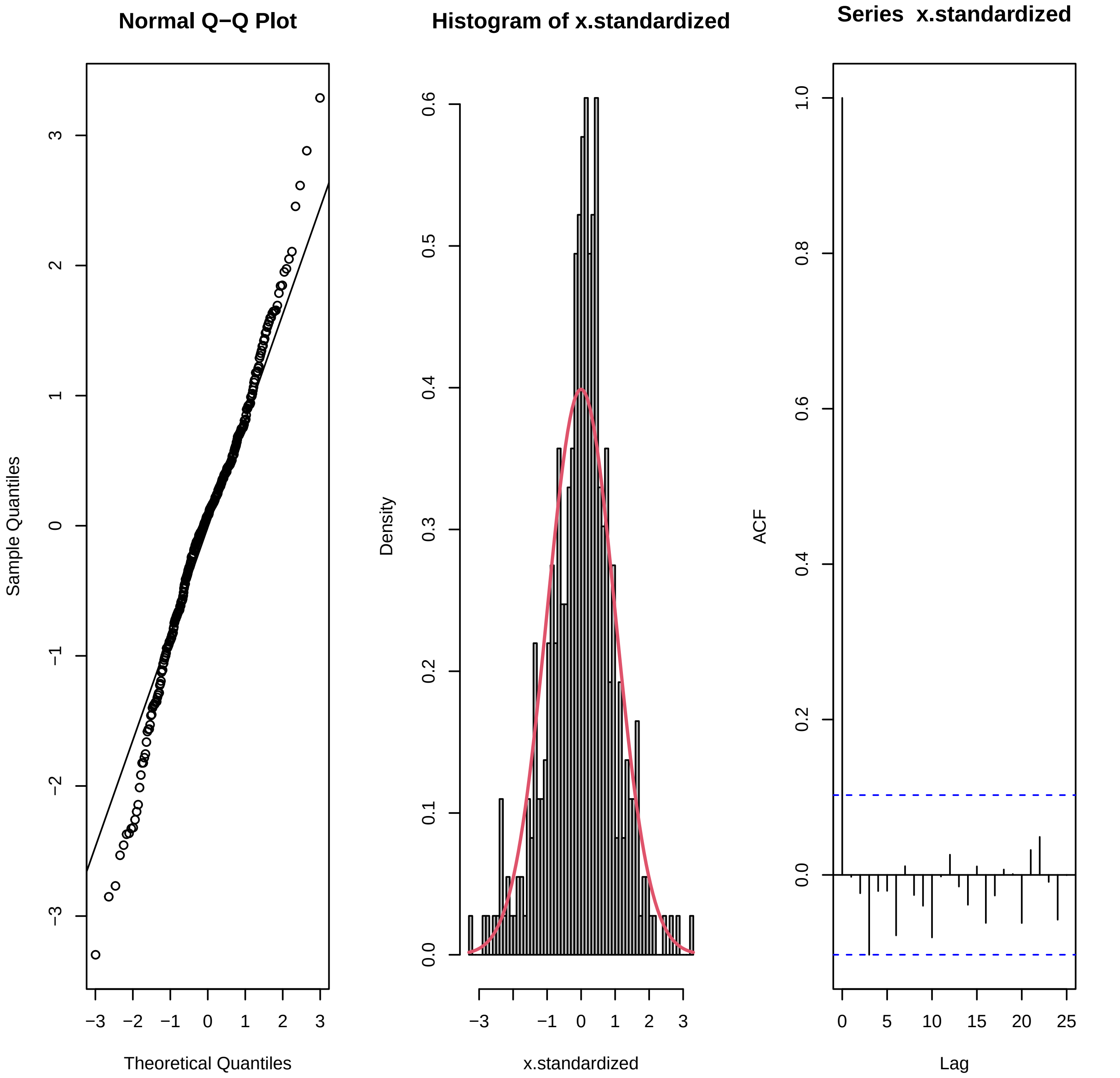

Normal Q−Q Plot
Sample Quantiles
Theoretical Quantiles
−3
−2
−1
0
1
2
3
Histogram of x.standardized
Density
x.standardized
0.0
0.1
0.2
0.3
0.4
0.5
0.6
Series x.standardized
ACF
Lag
0
5
10
15
20
25
1.0
0.8
0.6
0.4
0.2

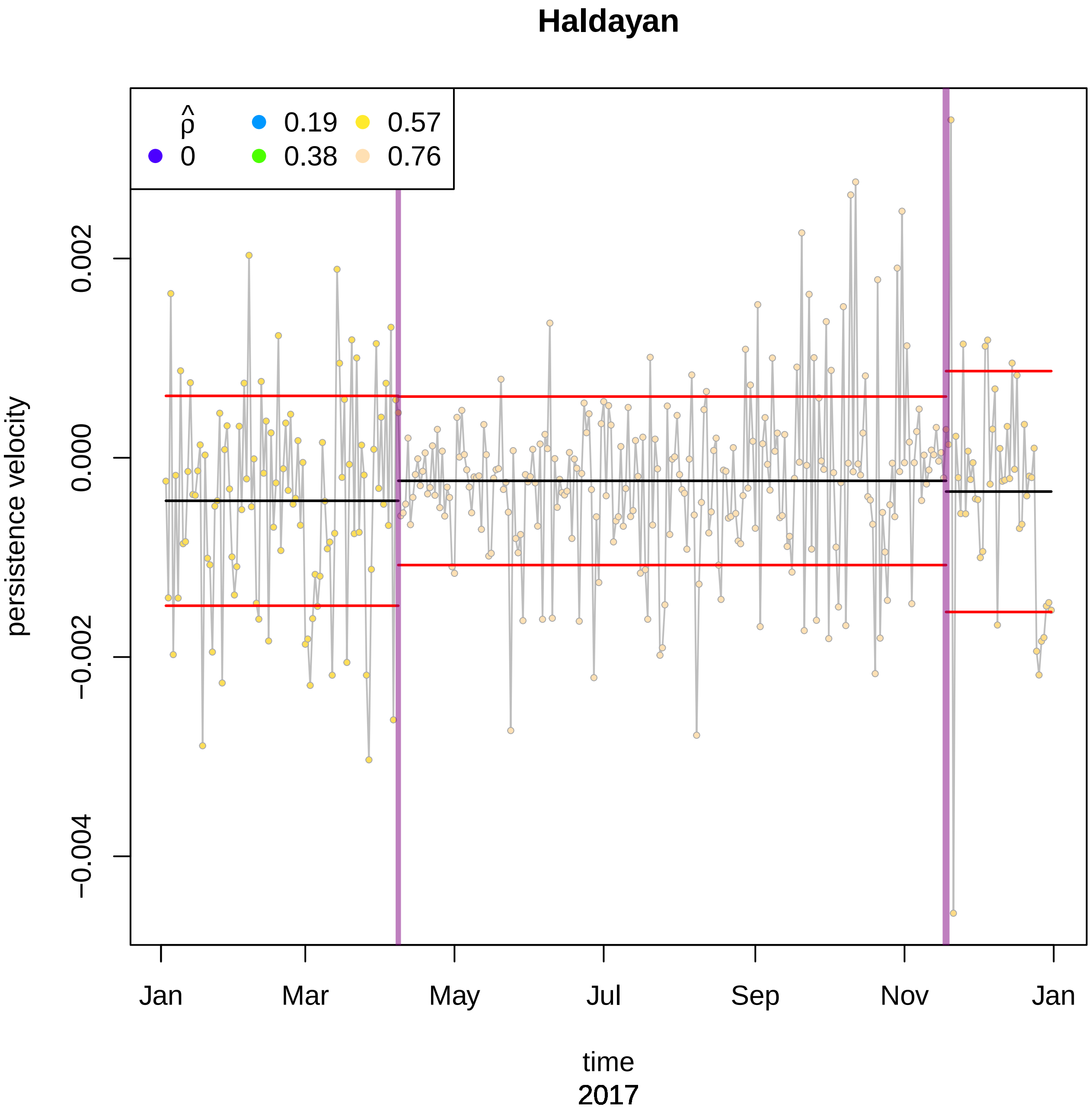
Haldayan
ρ̂
0
0.19
0.38
0.57
0.76
0.002
0.000
−0.002
−0.004
persistence velocity
Jan
Mar
May
Jul
Sep
Nov
Jan
time
2017

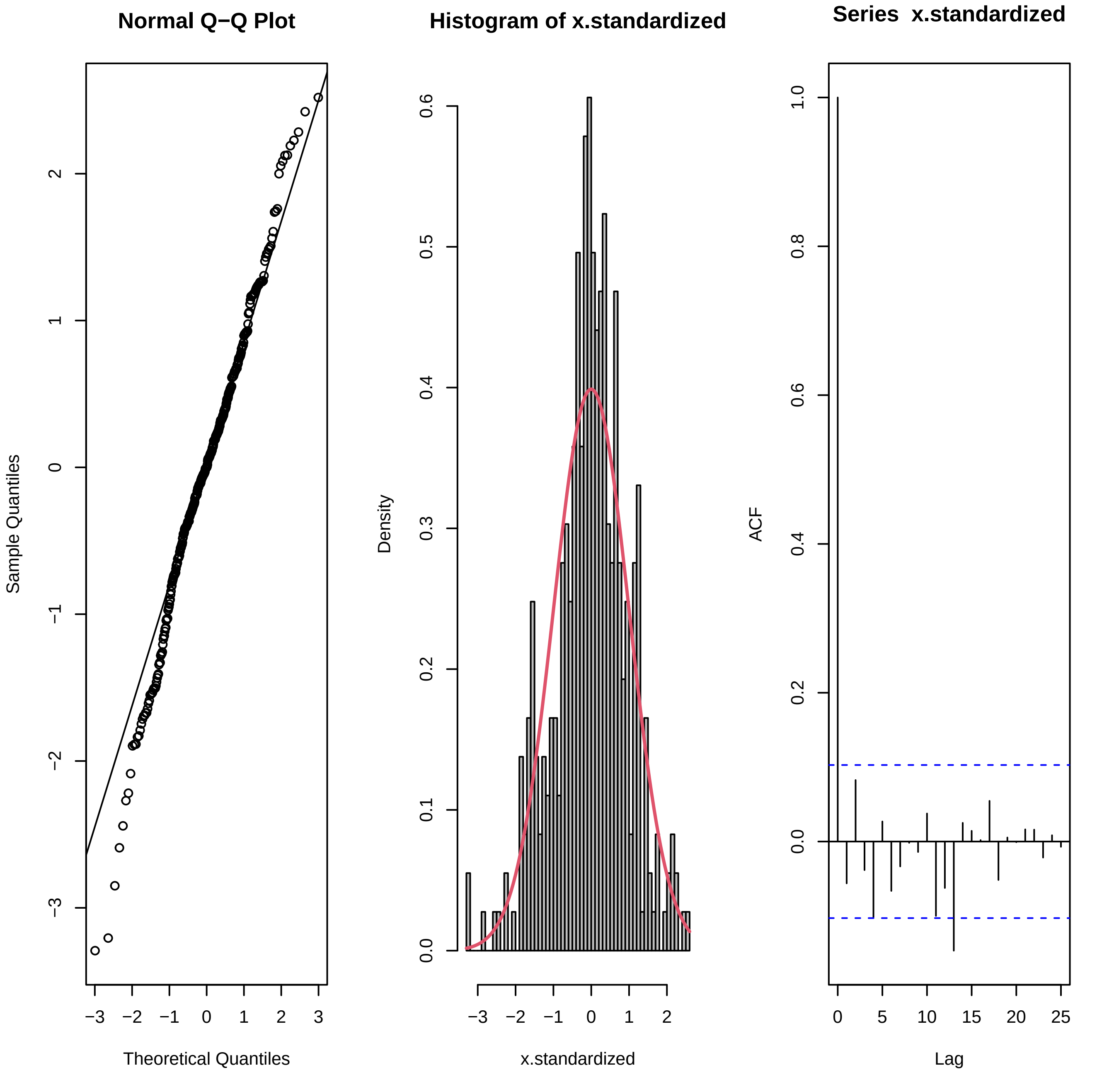
Normal Q−Q Plot
Sample Quantiles
Theoretical Quantiles
Histogram of x.standardized
Density
x.standardized
Series x.standardized
ACF
Lag

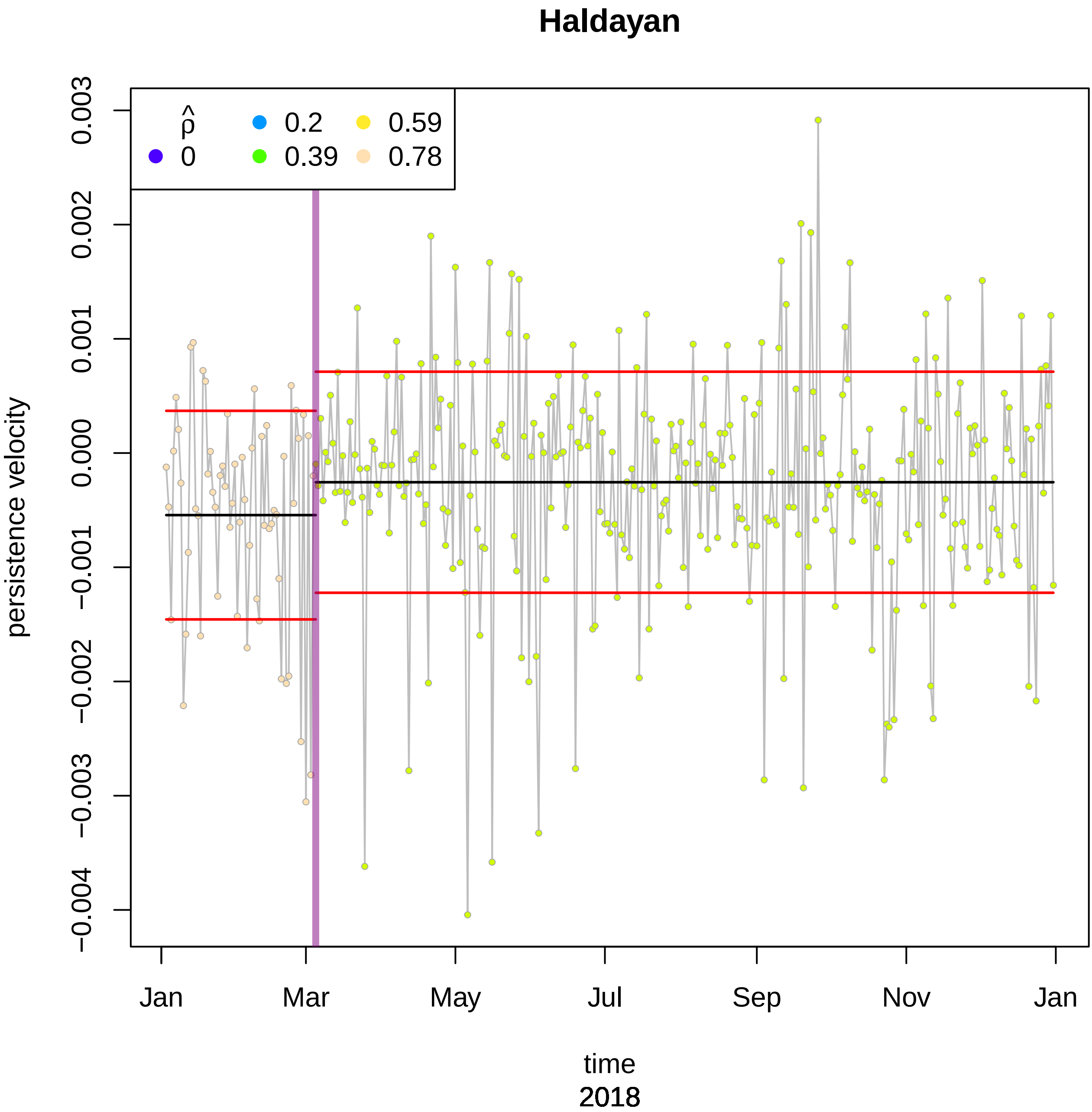

Haldayan
ρ̂
0
0.2
0.39
0.59
0.78
0.003
0.002
0.001
0.000
−0.001
−0.002
−0.003
−0.004
persistence velocity
Jan
Mar
May
Jul
Sep
Nov
Jan
time
2018

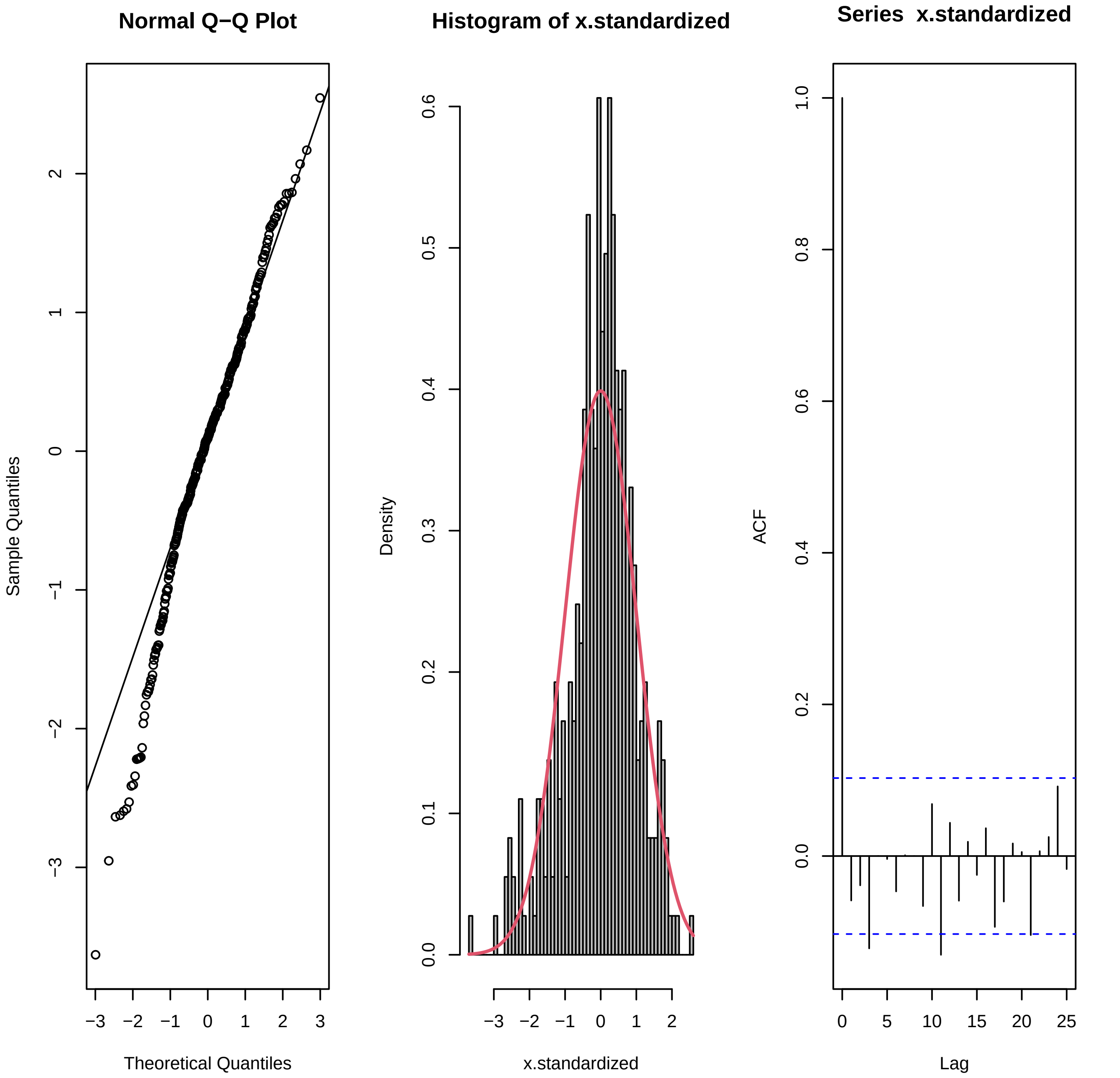

Normal Q−Q Plot
Sample Quantiles
Theoretical Quantiles
−3 −2 −1 0 1 2 3
Histogram of x.standardized
Density
x.standardized
0.0 0.1 0.2 0.3 0.4 0.5 0.6
Series x.standardized
ACF
Lag
0 5 10 15 20 25
0.0 0.2 0.4 0.6 0.8 1.0

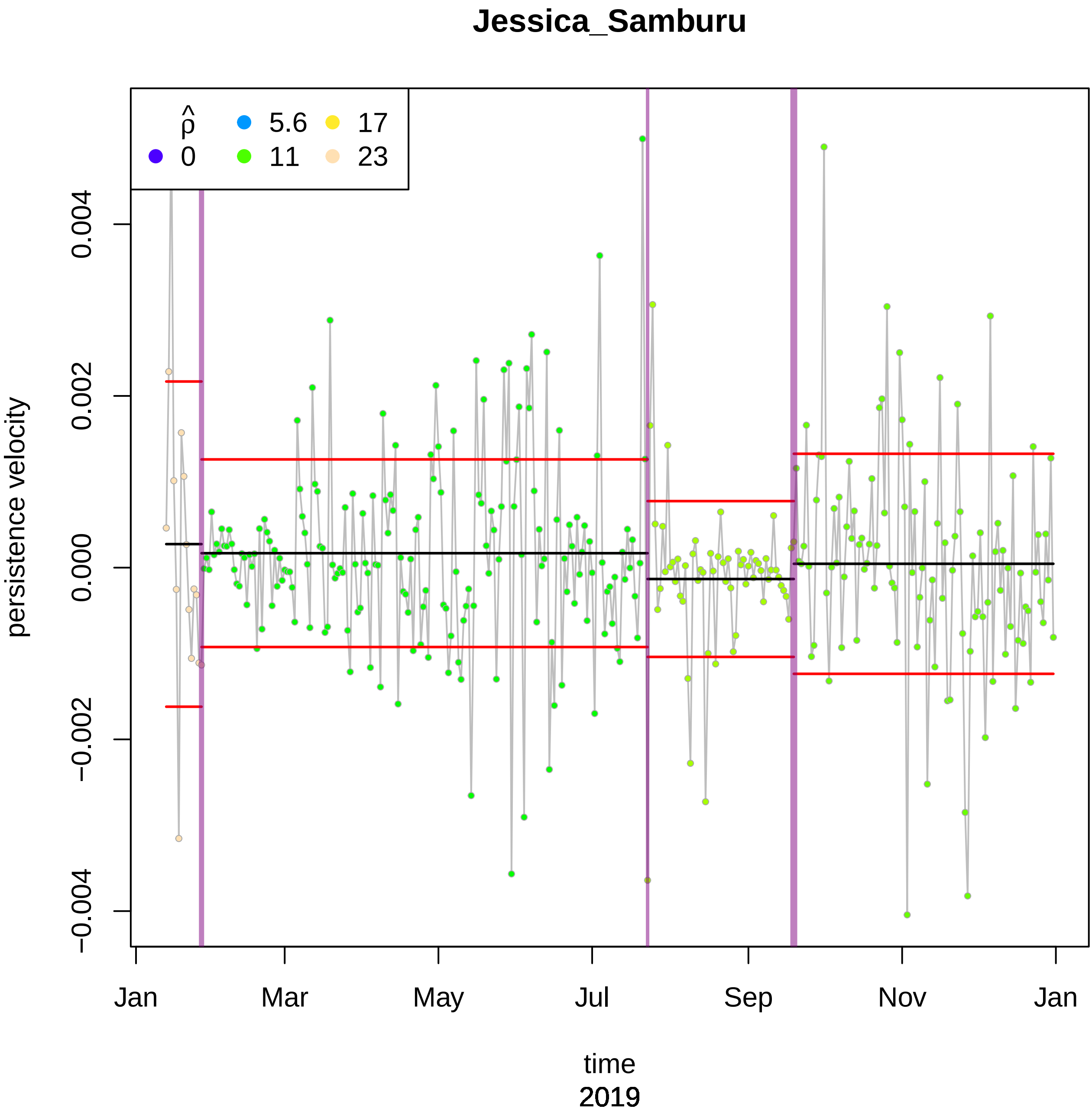

Jessica_Samburu
ρ̂
0
5.6
11
17
23
persistence velocity
0.004
0.002
0.000
−0.002
−0.004
Jan
Mar
May
Jul
Sep
Nov
Jan
time
2019

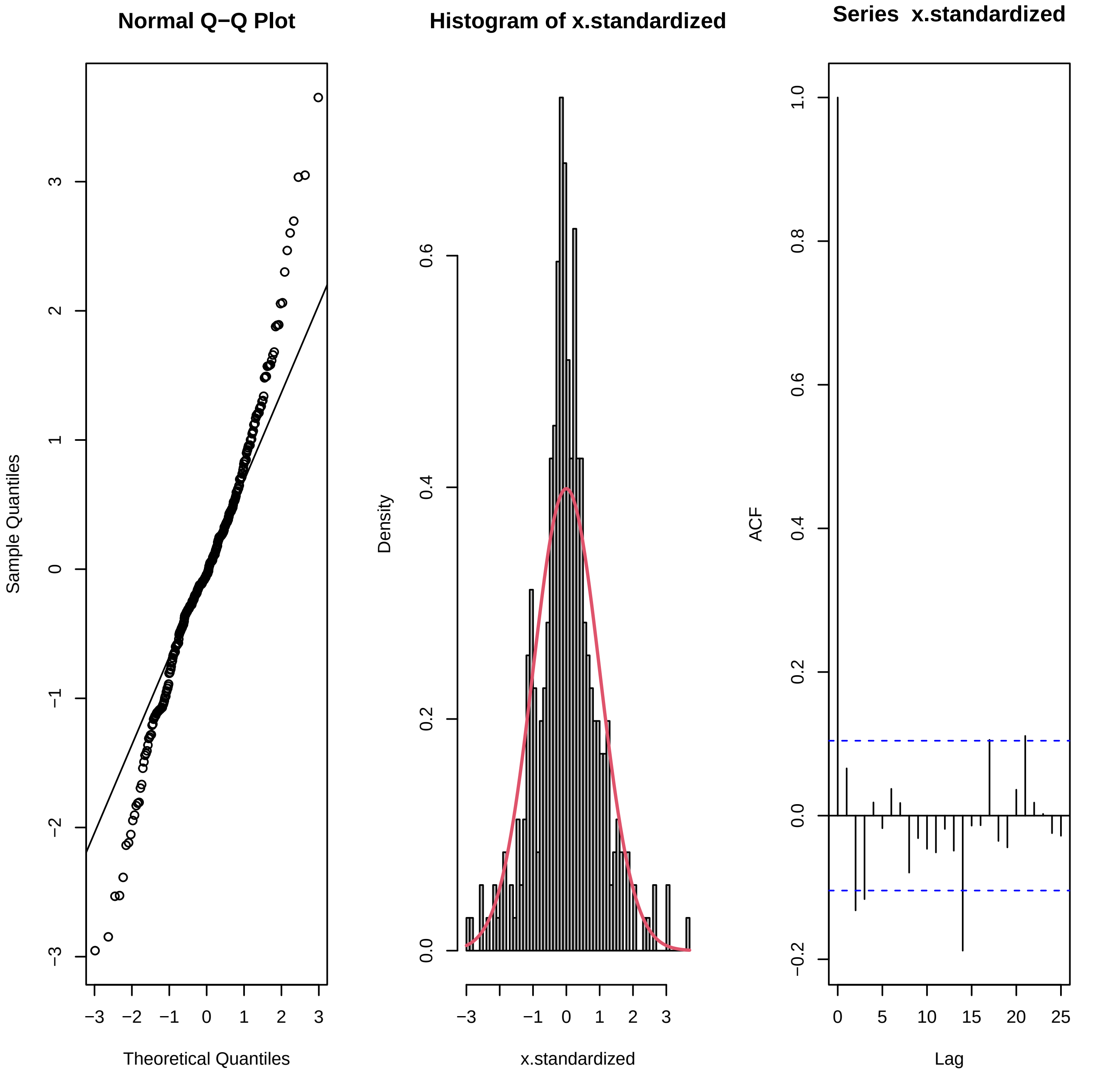
Normal Q−Q Plot
Sample Quantiles
Theoretical Quantiles
Histogram of x.standardized
Density
x.standardized
Series x.standardized
ACF
Lag

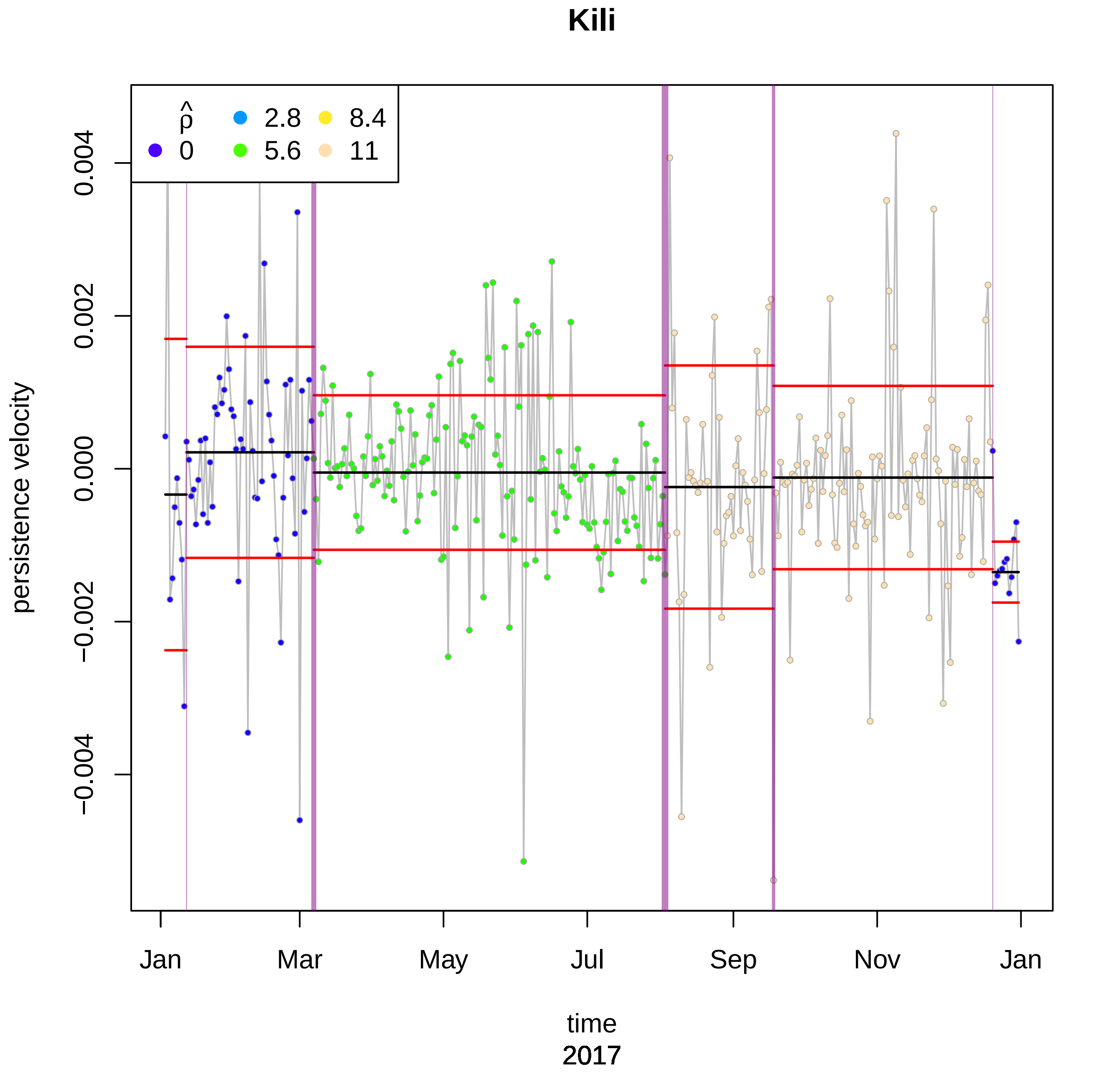
Kili
ρ̂
0
2.8
5.6
8.4
11
persistence velocity
0.004
0.002
0.000
−0.002
−0.004
Jan
Mar
May
Jul
Sep
Nov
Jan
time
2017

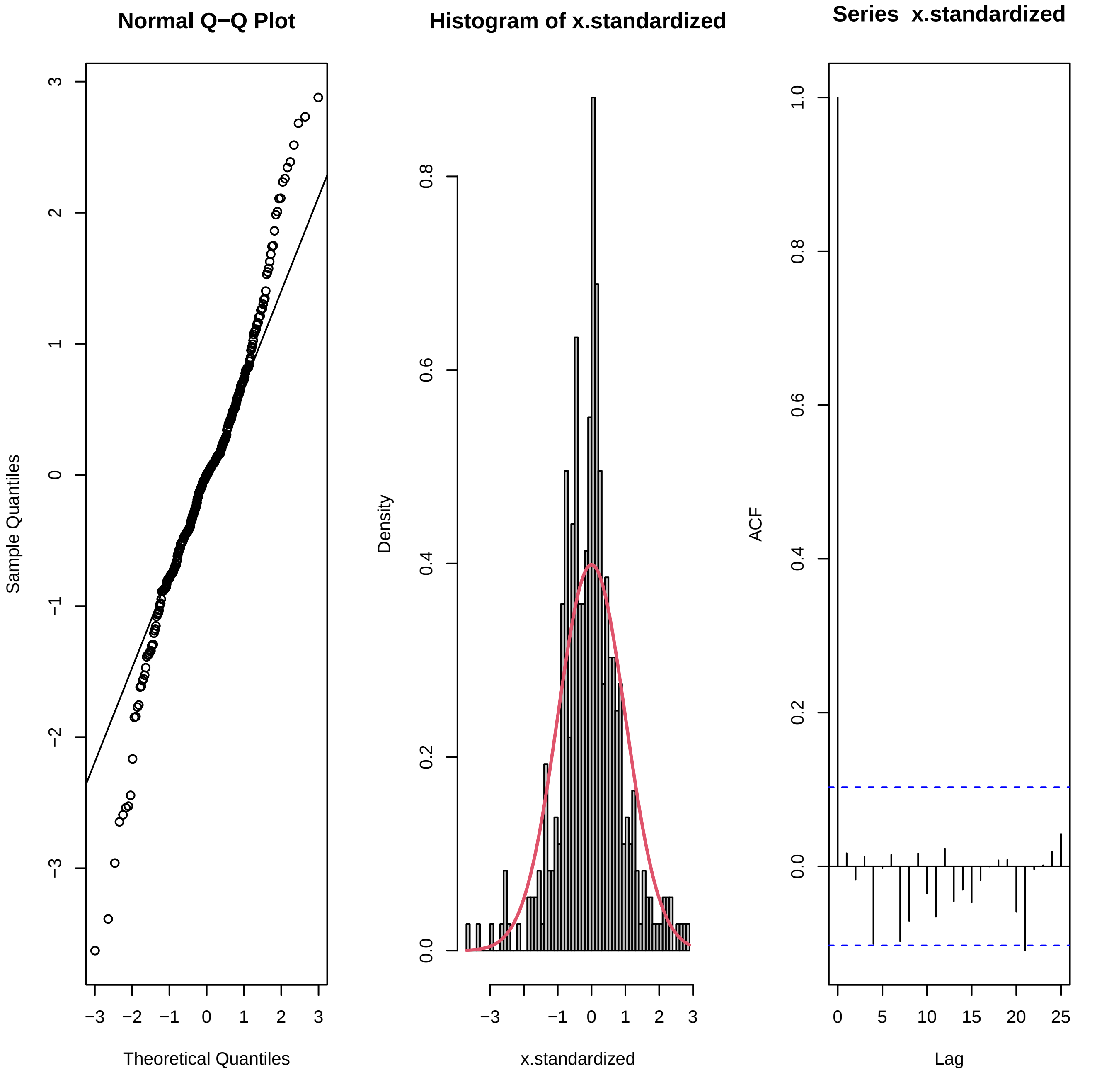

Normal Q−Q Plot
Sample Quantiles
Theoretical Quantiles
Histogram of x.standardized
Density
x.standardized
Series x.standardized
ACF
Lag

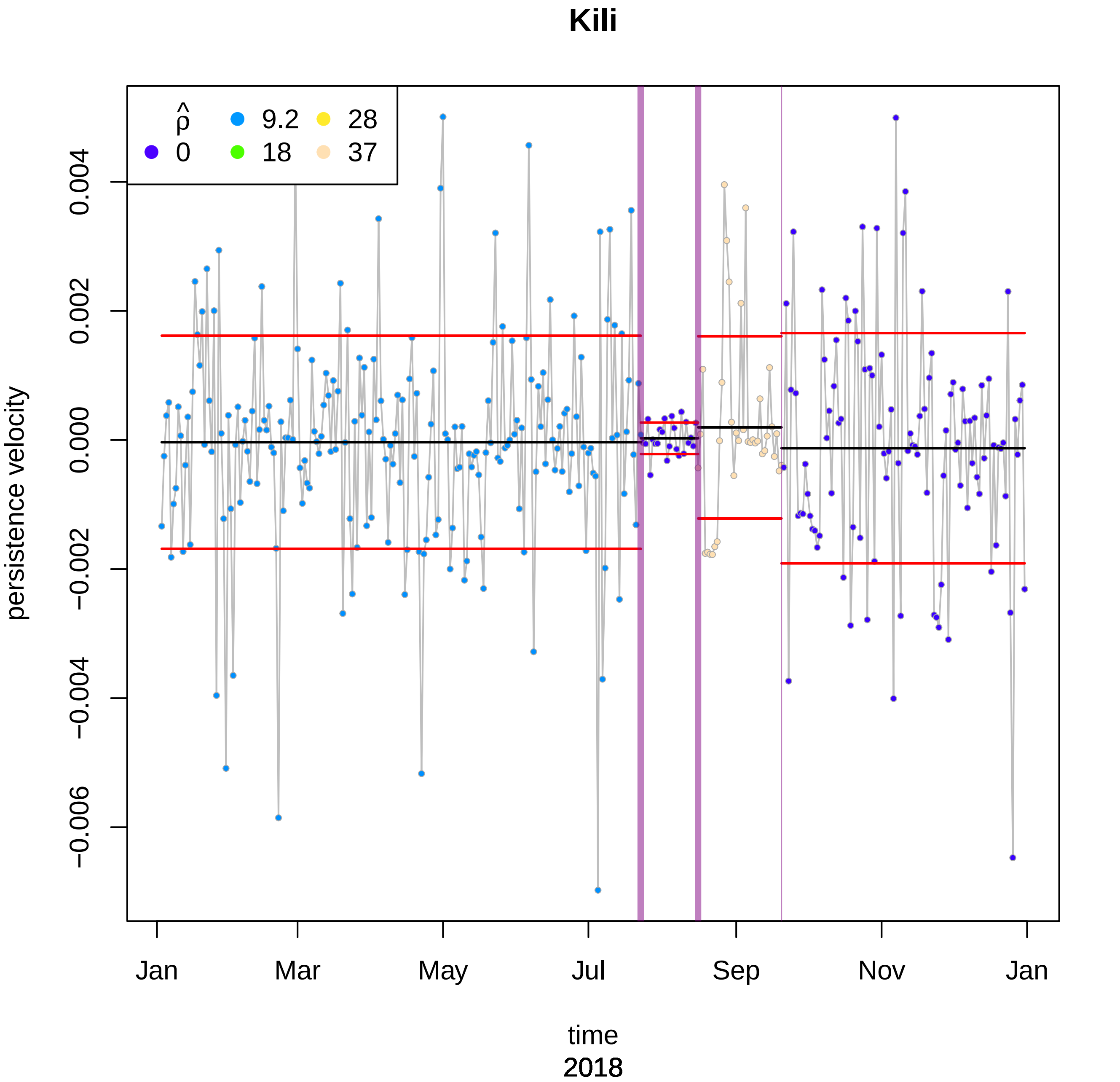

Kili
ρ̂
0
9.2
18
28
37
persistence velocity
0.004
0.002
0.000
−0.002
−0.004
−0.006
Jan
Mar
May
Jul
Sep
Nov
Jan
time
2018

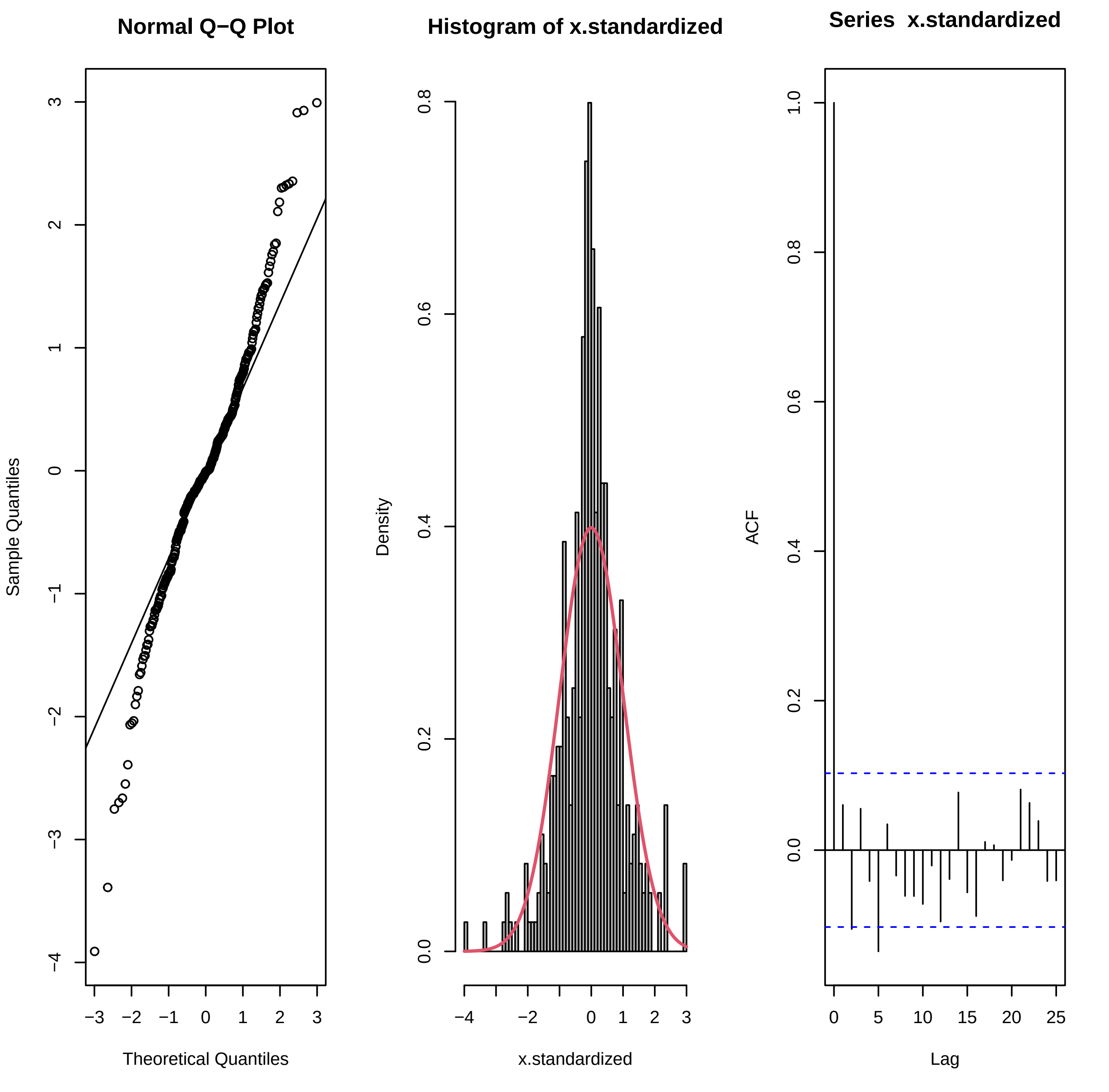

Normal Q−Q Plot
Sample Quantiles
Theoretical Quantiles
Histogram of x.standardized
Density
x.standardized
Series x.standardized
ACF
Lag

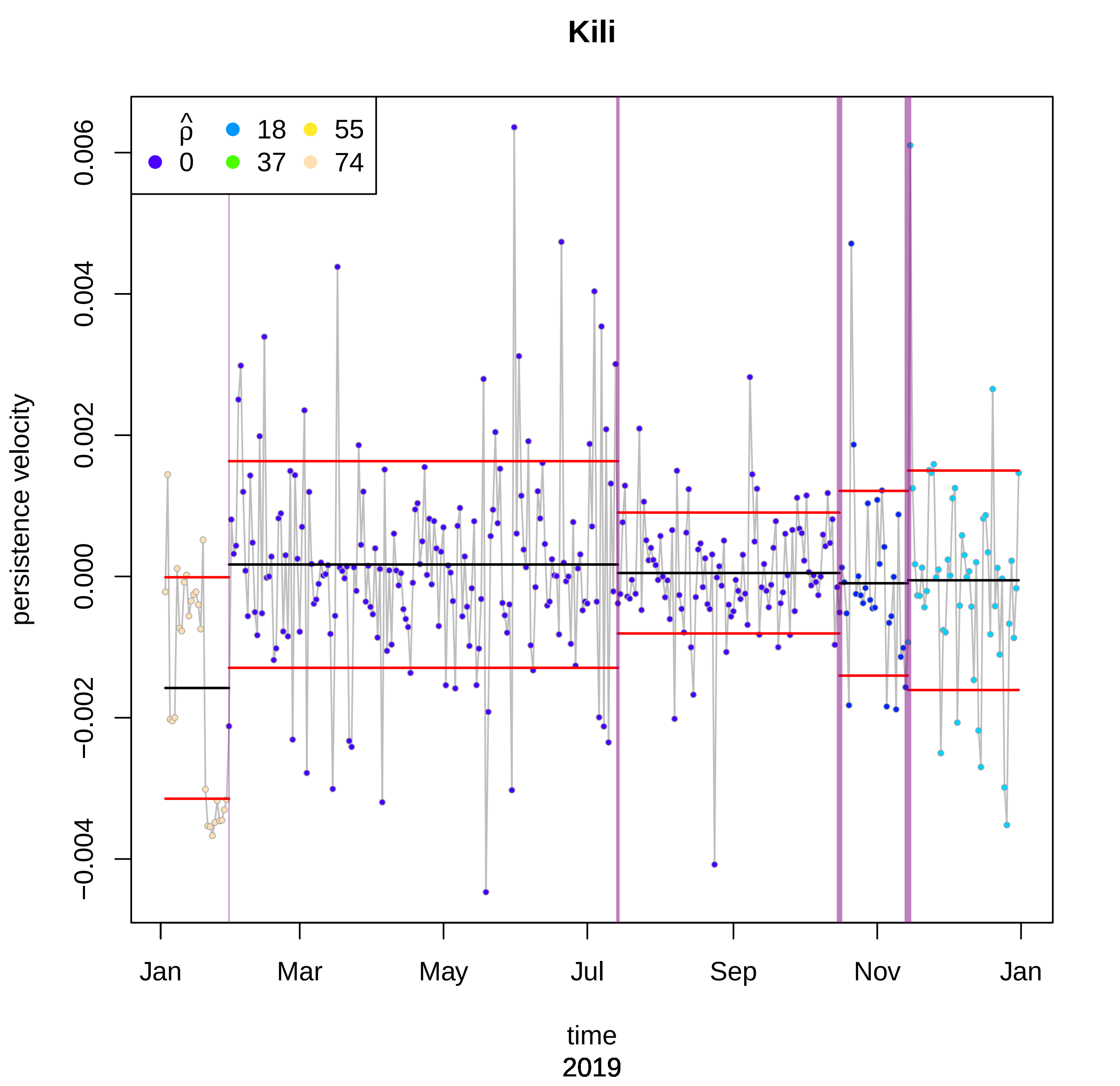

Kili
ρ̂
0
18
37
55
74
0.006
0.004
0.002
0.000
−0.002
−0.004
persistence velocity
Jan
Mar
May
Jul
Sep
Nov
Jan
time
2019

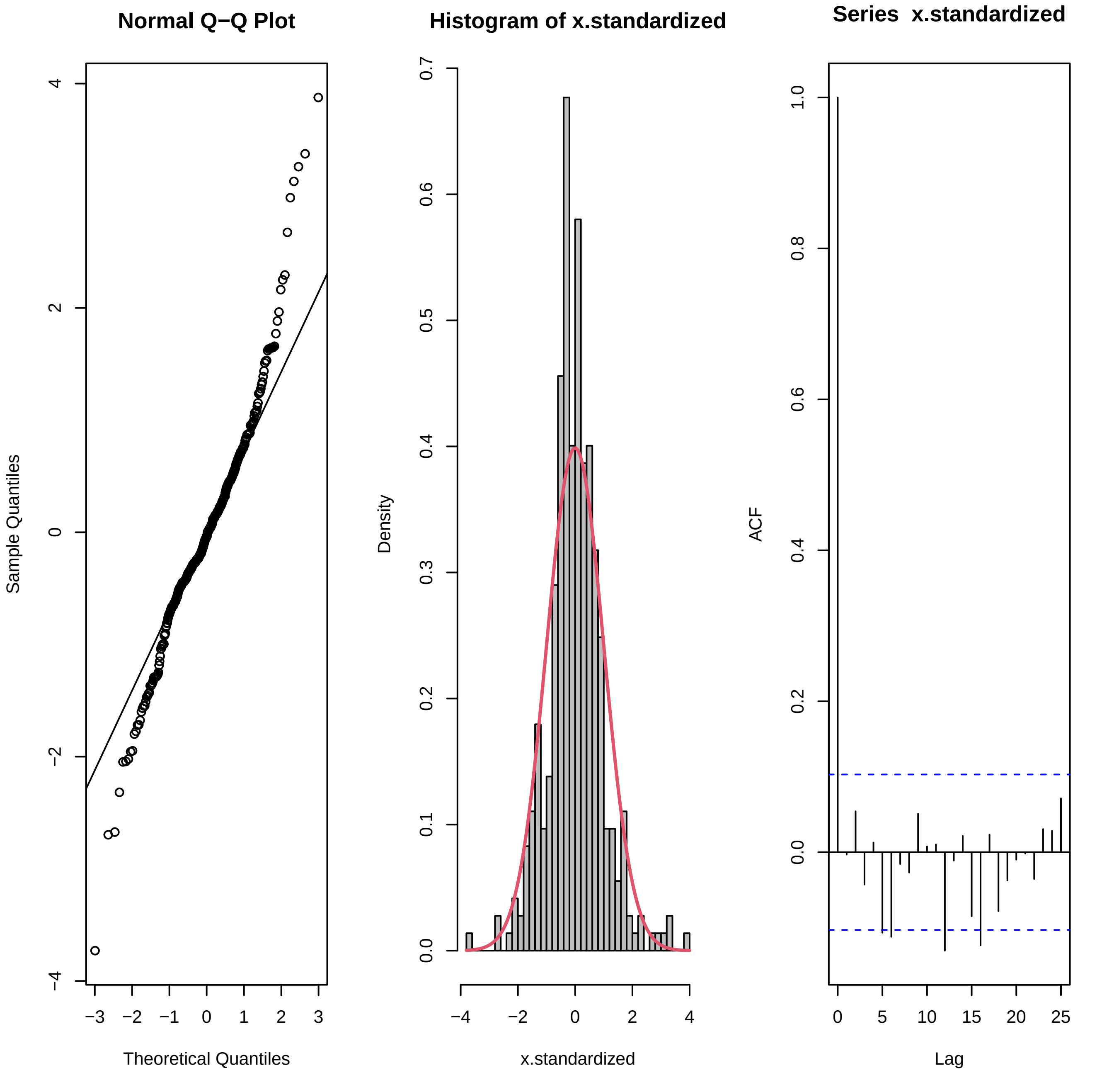
Normal Q−Q Plot
Theoretical Quantiles
Sample Quantiles
Histogram of x.standardized
x.standardized
Density
Series x.standardized
Lag
ACF

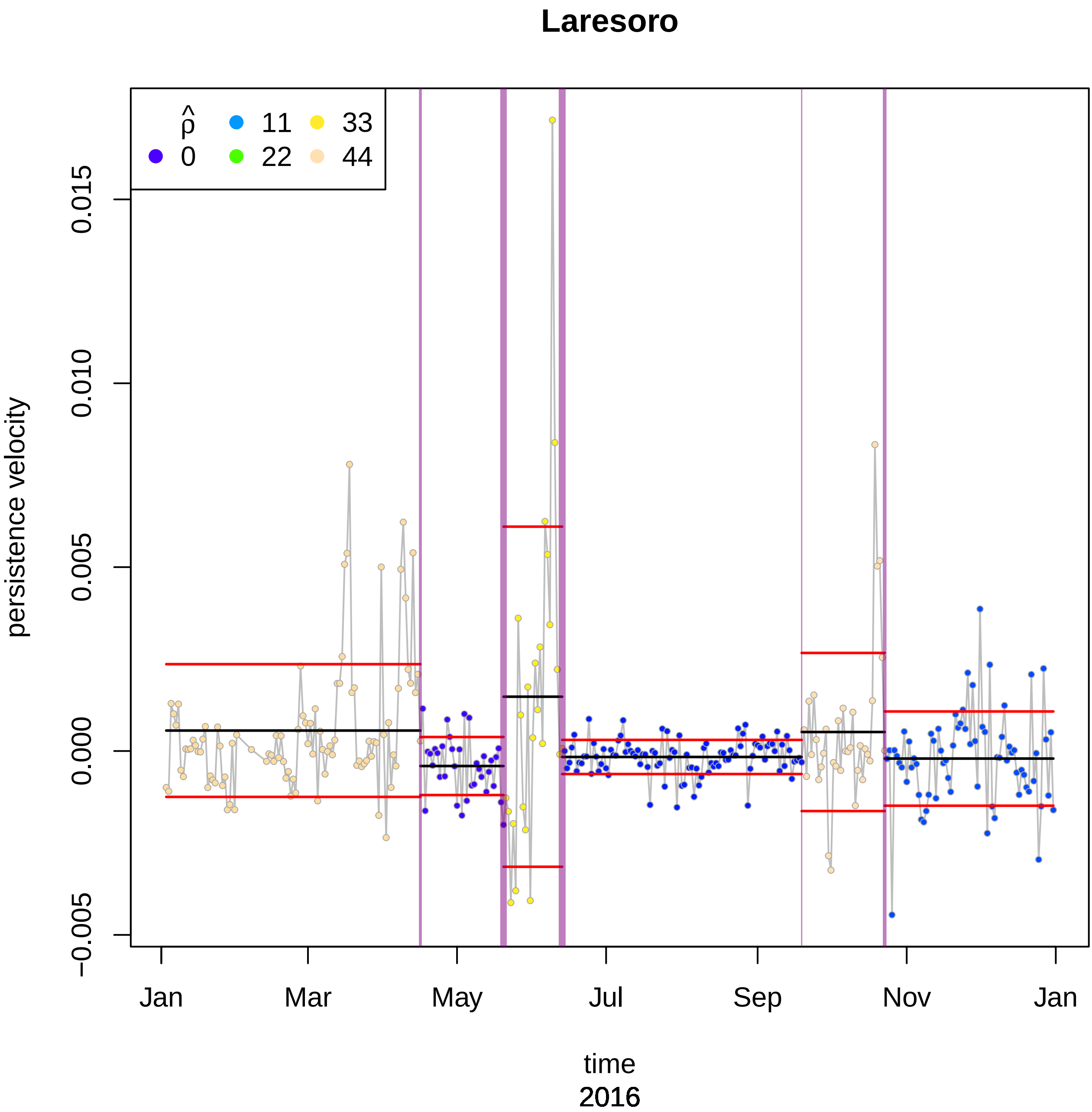

Laresoro
$\hat{\rho}$
0
11
22
33
44
0.015
0.010
0.005
0.000
−0.005
persistence velocity
Jan
Mar
May
Jul
Sep
Nov
Jan
time
2016

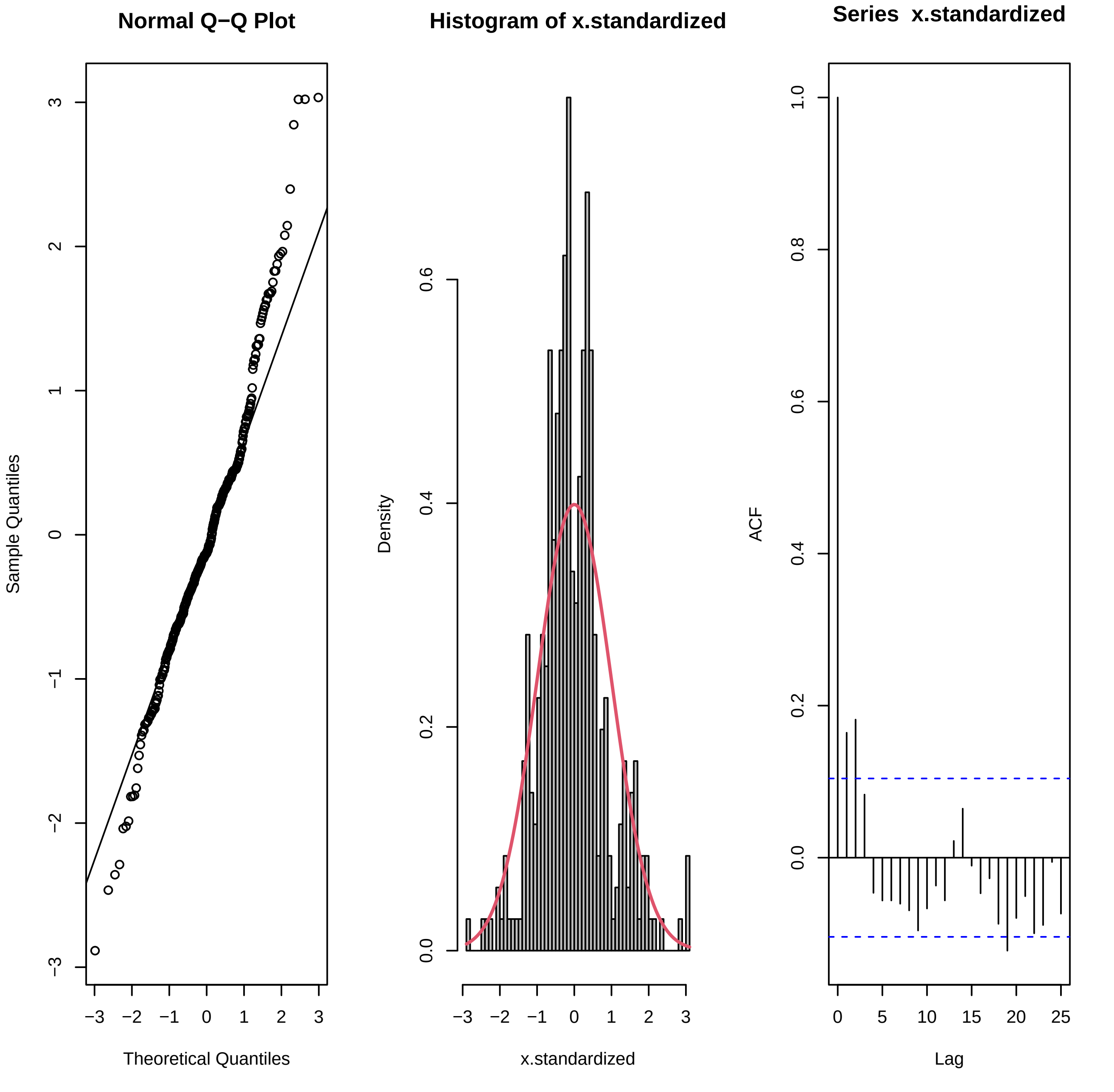

Normal Q−Q Plot
Sample Quantiles
Theoretical Quantiles
Histogram of x.standardized
Density
x.standardized
Series x.standardized
ACF
Lag

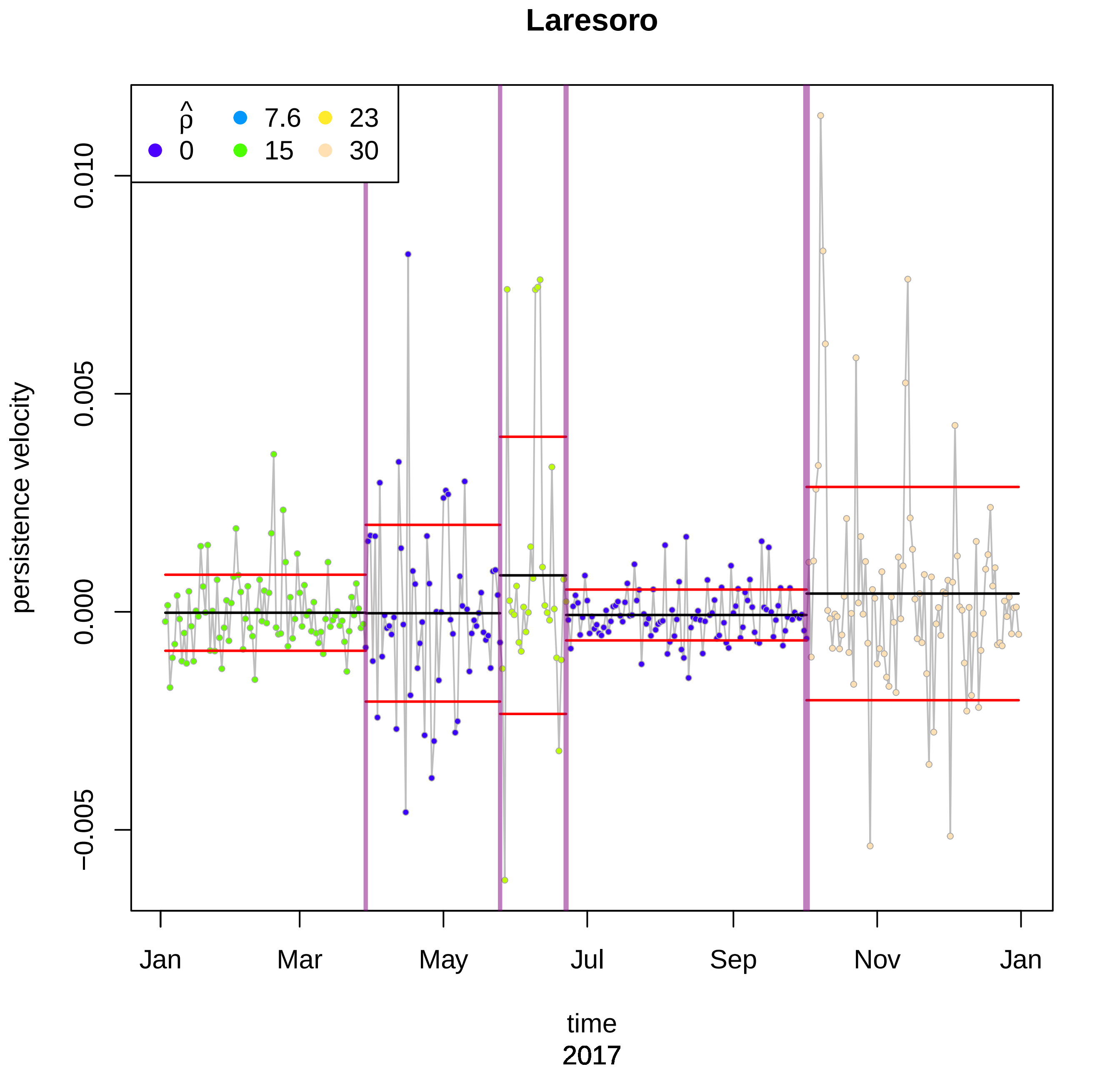
Laresoro
ρ̂
0
7.6
15
23
30
persistence velocity
0.010
0.005
0.000
−0.005
Jan
Mar
May
Jul
Sep
Nov
Jan
time
2017

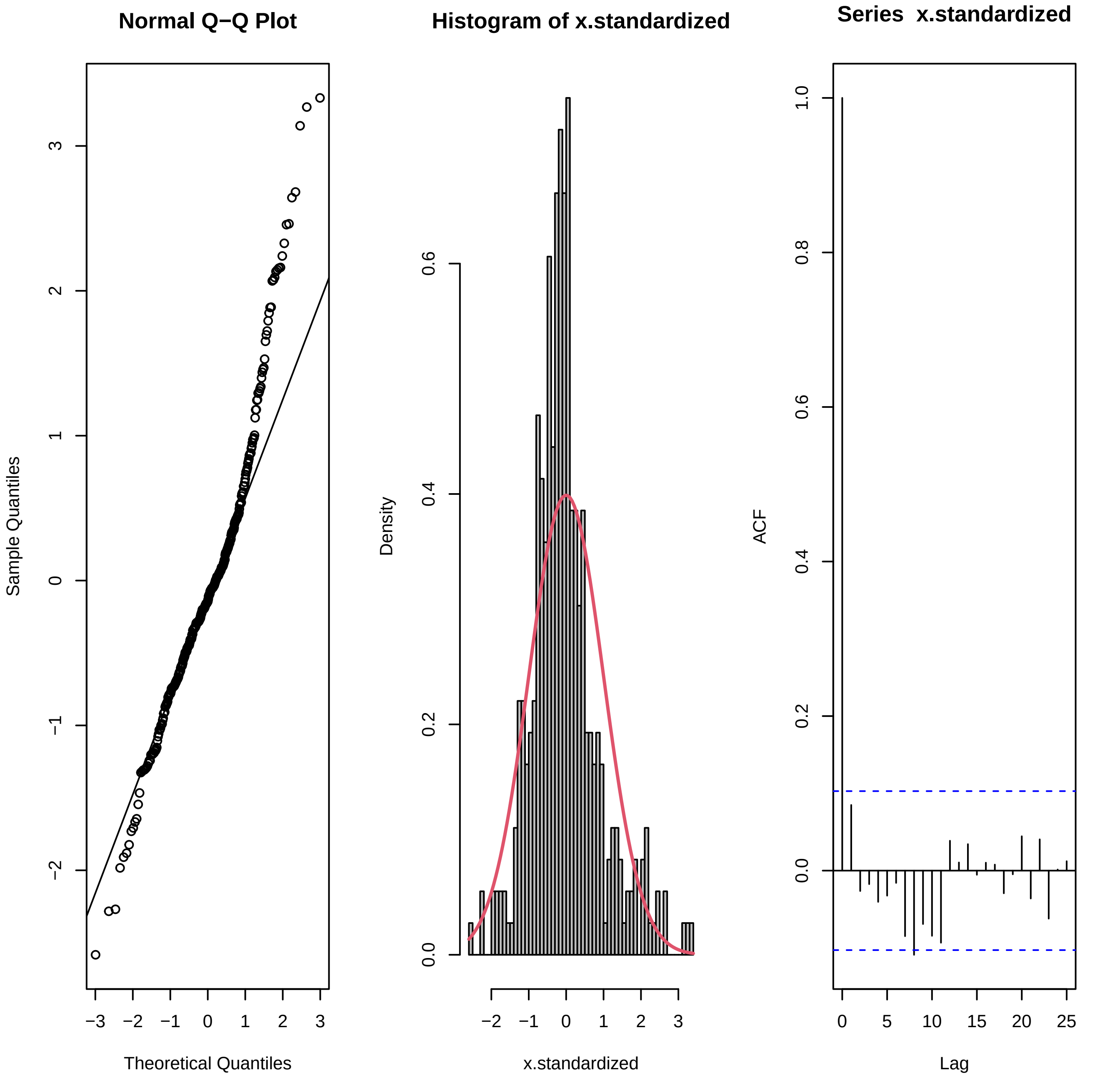

Normal Q−Q Plot
Sample Quantiles
Theoretical Quantiles
−3
−2
−1
0
1
2
3
Histogram of x.standardized
Density
x.standardized
0.0
0.2
0.4
0.6
Series x.standardized
ACF
Lag
0.0
0.2
0.4
0.6
0.8
1.0
0
5
10
15
20
25

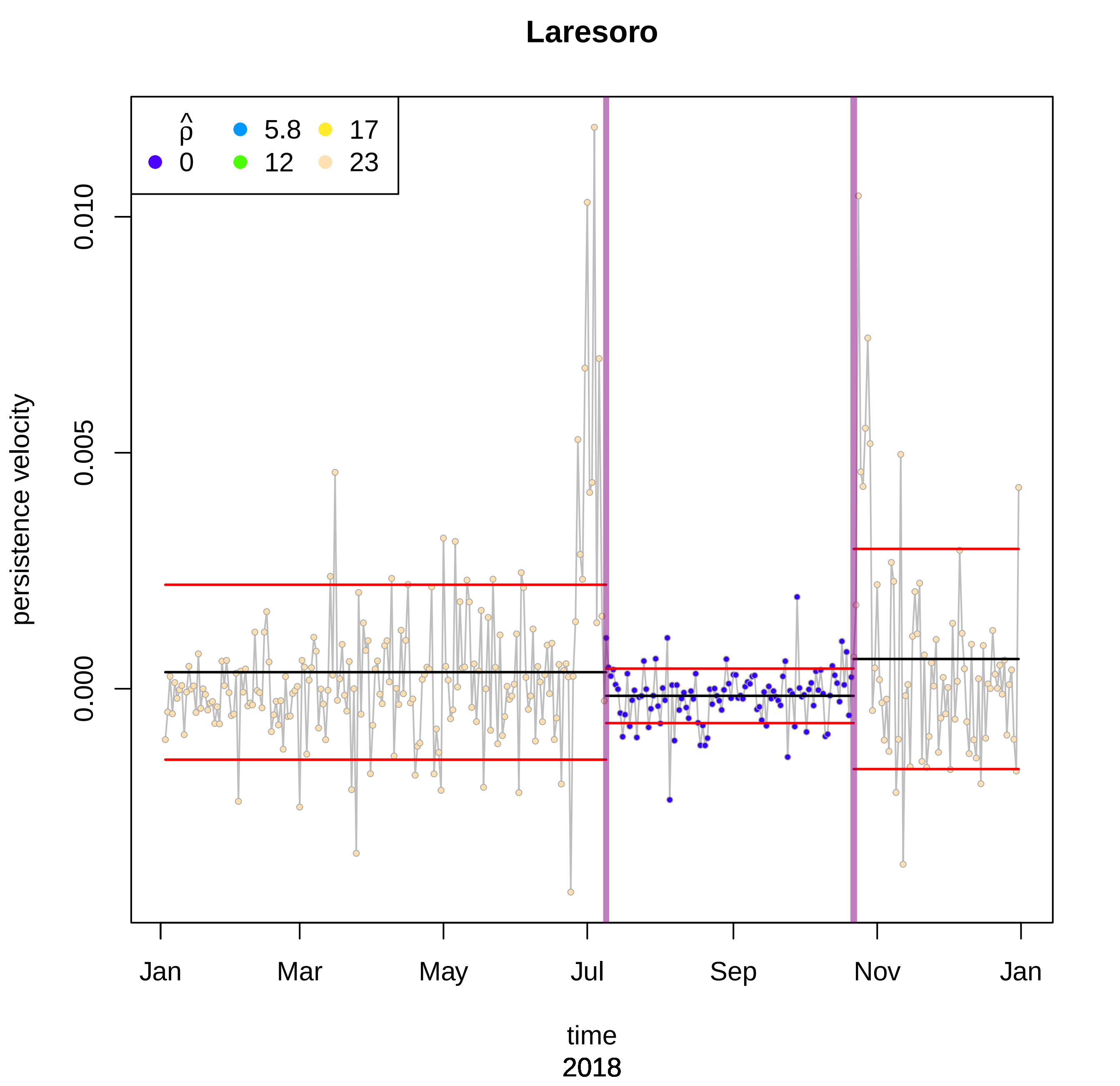
Laresoro
ρ̂
0
5.8
12
17
23
0.010
0.005
0.000
persistence velocity
Jan
Mar
May
Jul
Sep
Nov
Jan
time
2018

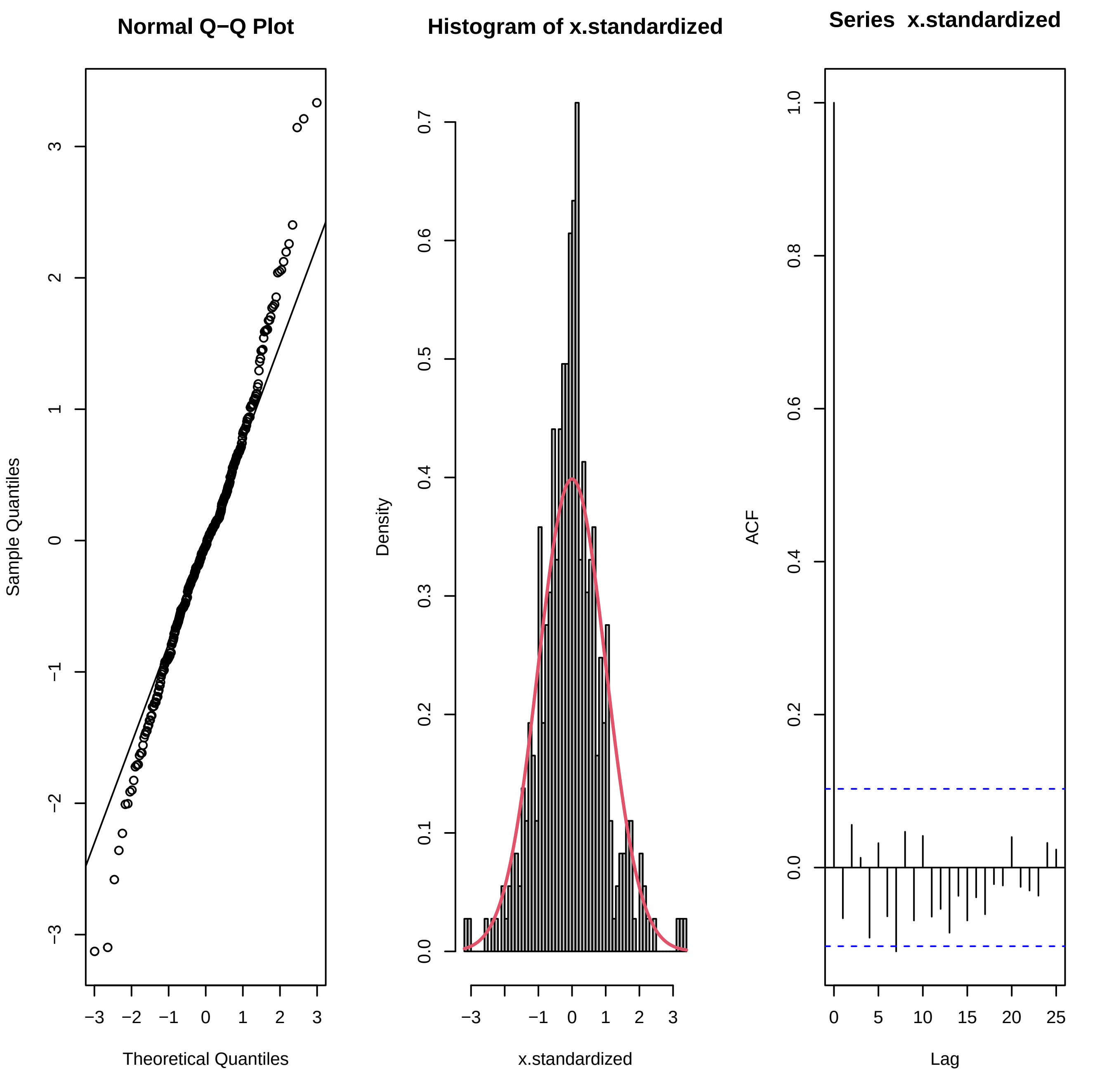

Normal Q−Q Plot
Sample Quantiles
Theoretical Quantiles
−3
−2
−1
0
1
2
3
Histogram of x.standardized
Density
x.standardized
0.0
0.1
0.2
0.3
0.4
0.5
0.6
0.7
Series x.standardized
ACF
Lag
0.0
0.2
0.4
0.6
0.8
1.0
0
5
10
15
20
25

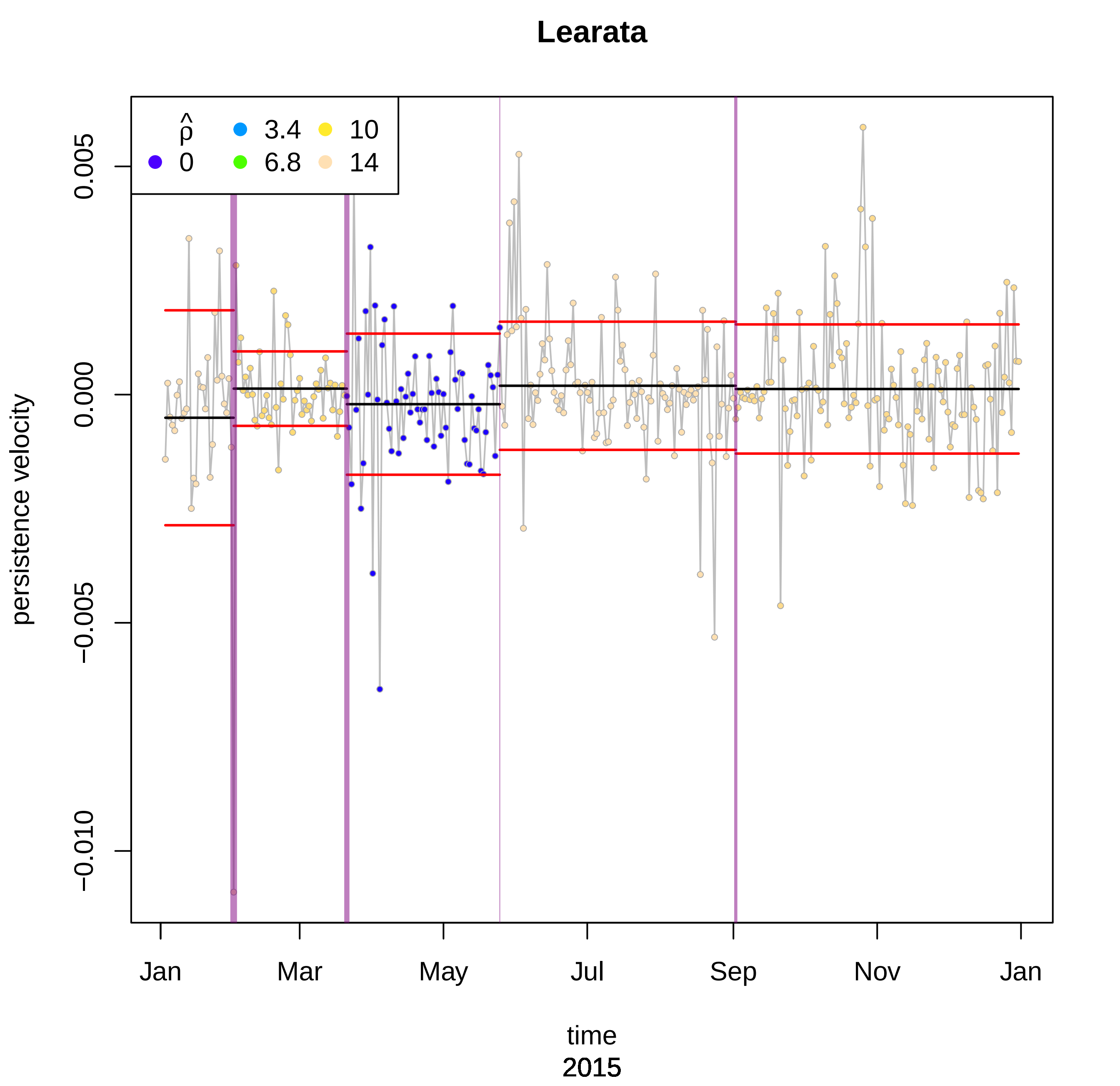

Learata
ρ̂
0
3.4
6.8
10
14
0.005
0.000
−0.005
−0.010
persistence velocity
Jan
Mar
May
Jul
Sep
Nov
Jan
time
2015

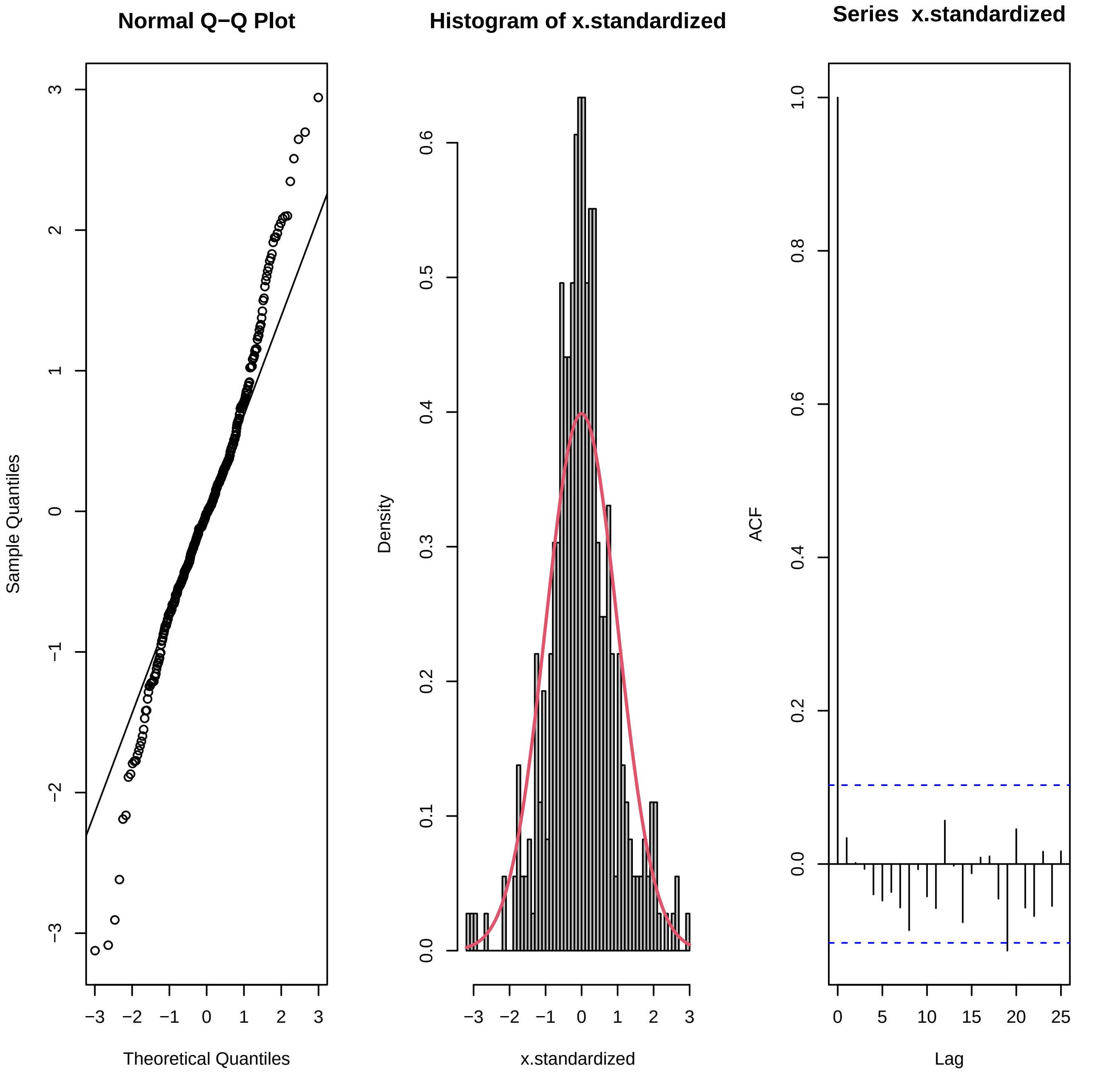
Normal Q−Q Plot
Sample Quantiles
Theoretical Quantiles
Histogram of x.standardized
Density
x.standardized
Series x.standardized
ACF
Lag

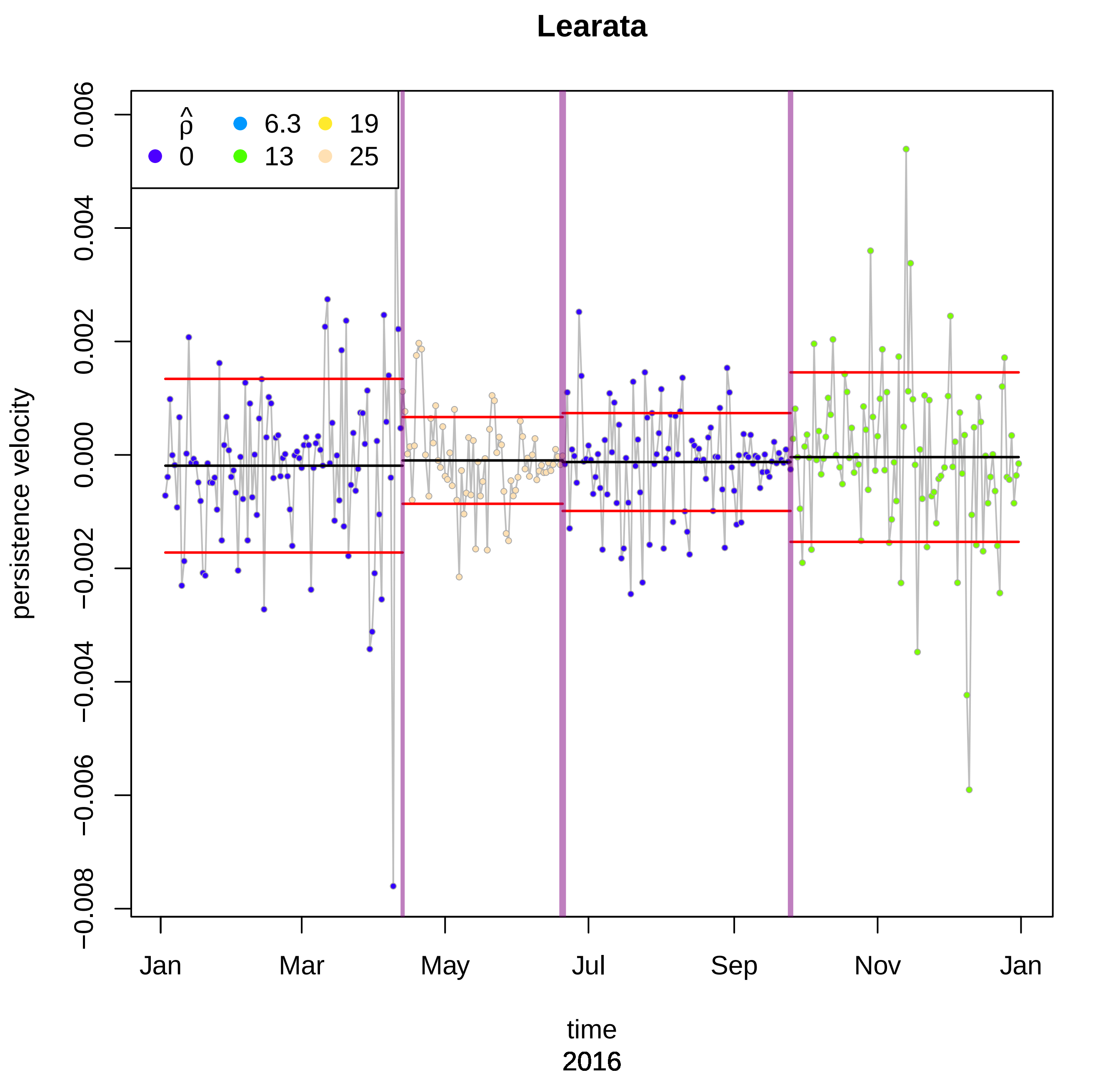

Learata
ρ̂
0
6.3
13
19
25
persistence velocity
0.006
0.004
0.002
0.000
−0.002
−0.004
−0.006
−0.008
Jan
Mar
May
Jul
Sep
Nov
Jan
time
2016

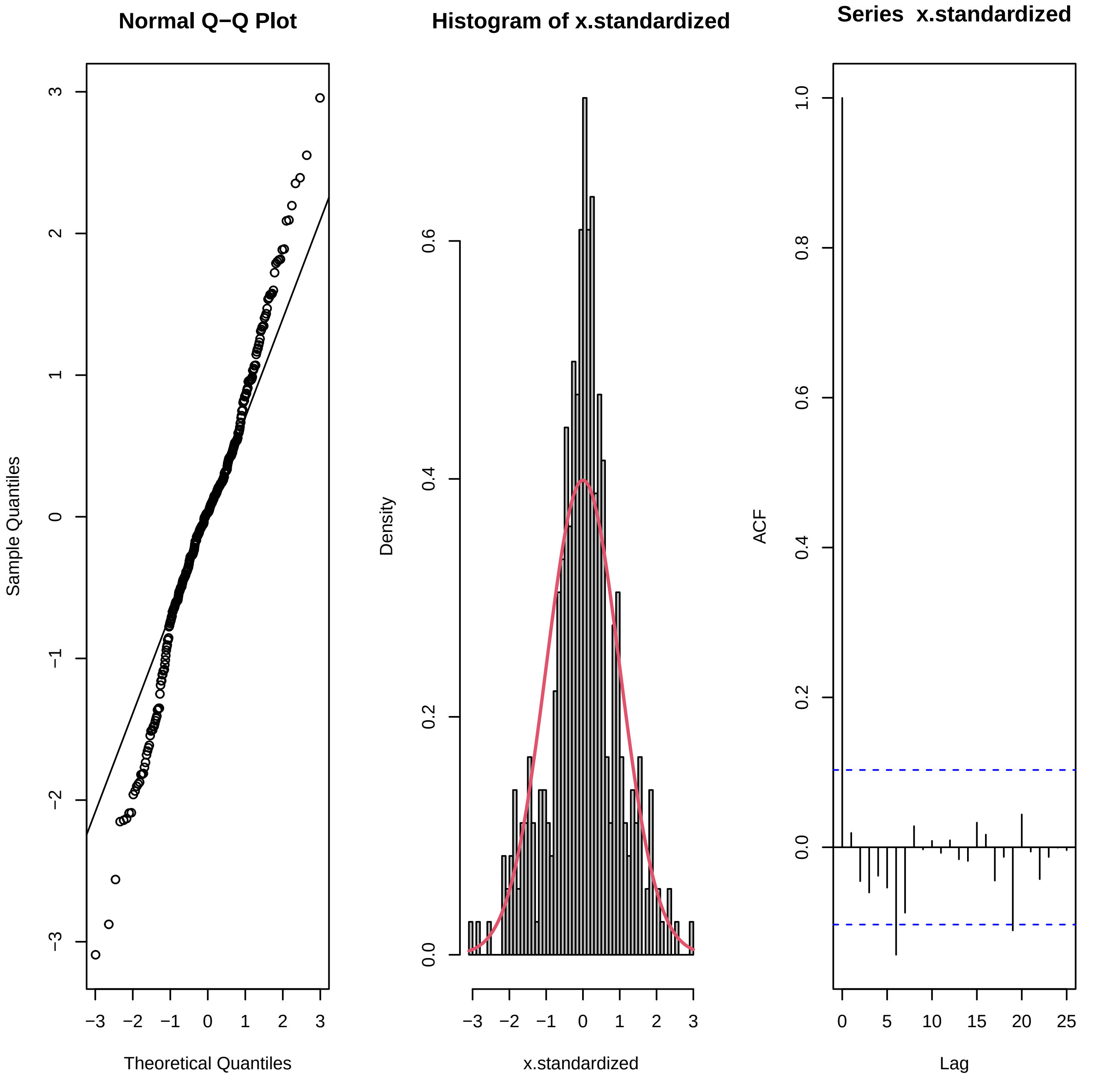
Normal Q−Q Plot
Sample Quantiles
Theoretical Quantiles
Histogram of x.standardized
Density
x.standardized
Series x.standardized
ACF
Lag

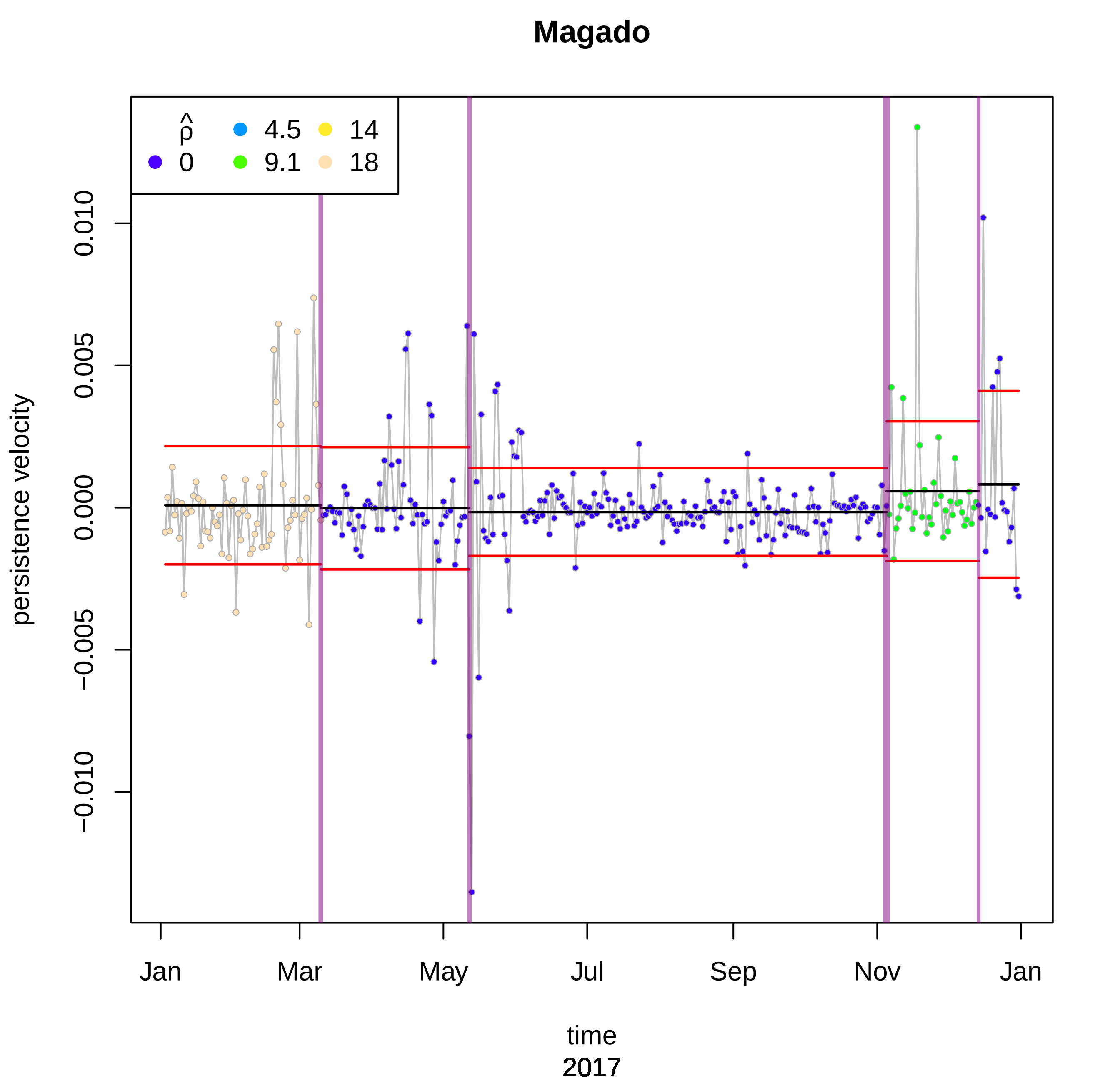

Magado
ρ̂
0
4.5
9.1
14
18
0.010
0.005
0.000
−0.005
−0.010
persistence velocity
Jan
Mar
May
Jul
Sep
Nov
Jan
time
2017

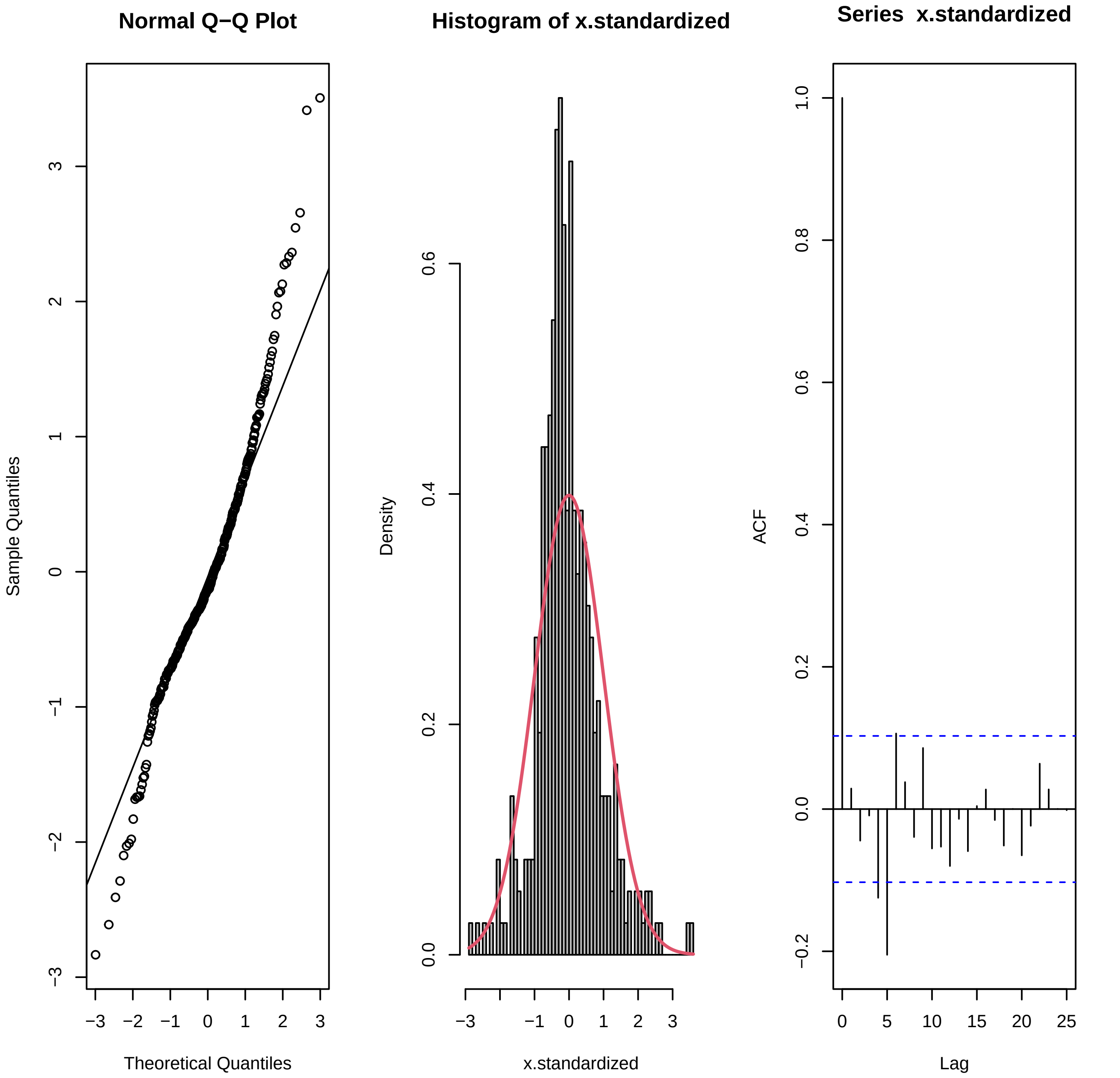

Normal Q−Q Plot
Sample Quantiles
Theoretical Quantiles
−3
−2
−1
0
1
2
3
Histogram of x.standardized
Density
x.standardized
0.0
0.2
0.4
0.6
Series x.standardized
ACF
Lag
−0.2
0.0
0.2
0.4
0.6
0.8
1.0
0
5
10
15
20
25

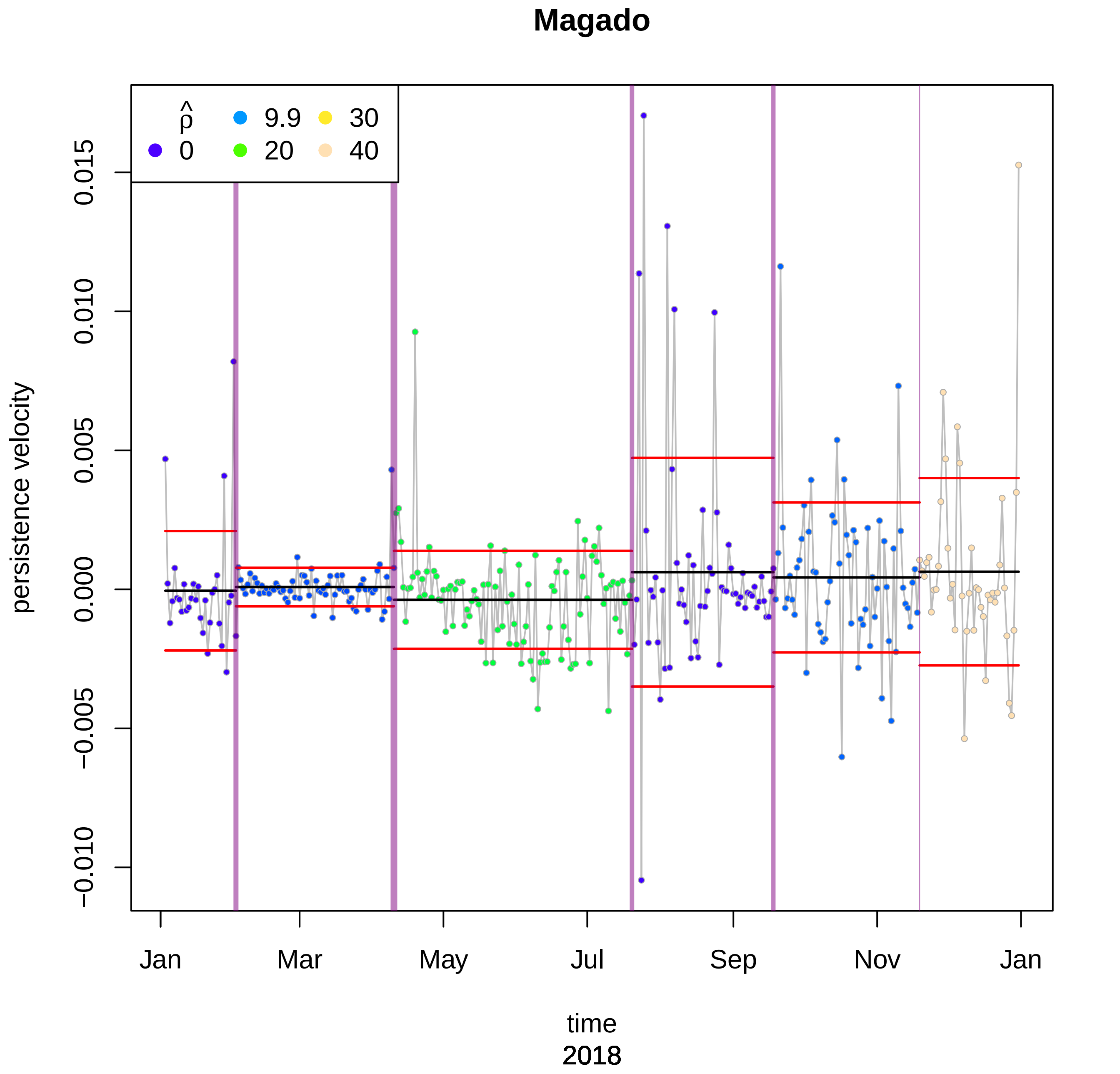

Magado
ρ̂
0
9.9
20
30
40
0.015
0.010
0.005
0.000
−0.005
−0.010
persistence velocity
Jan
Mar
May
Jul
Sep
Nov
Jan
time
2018

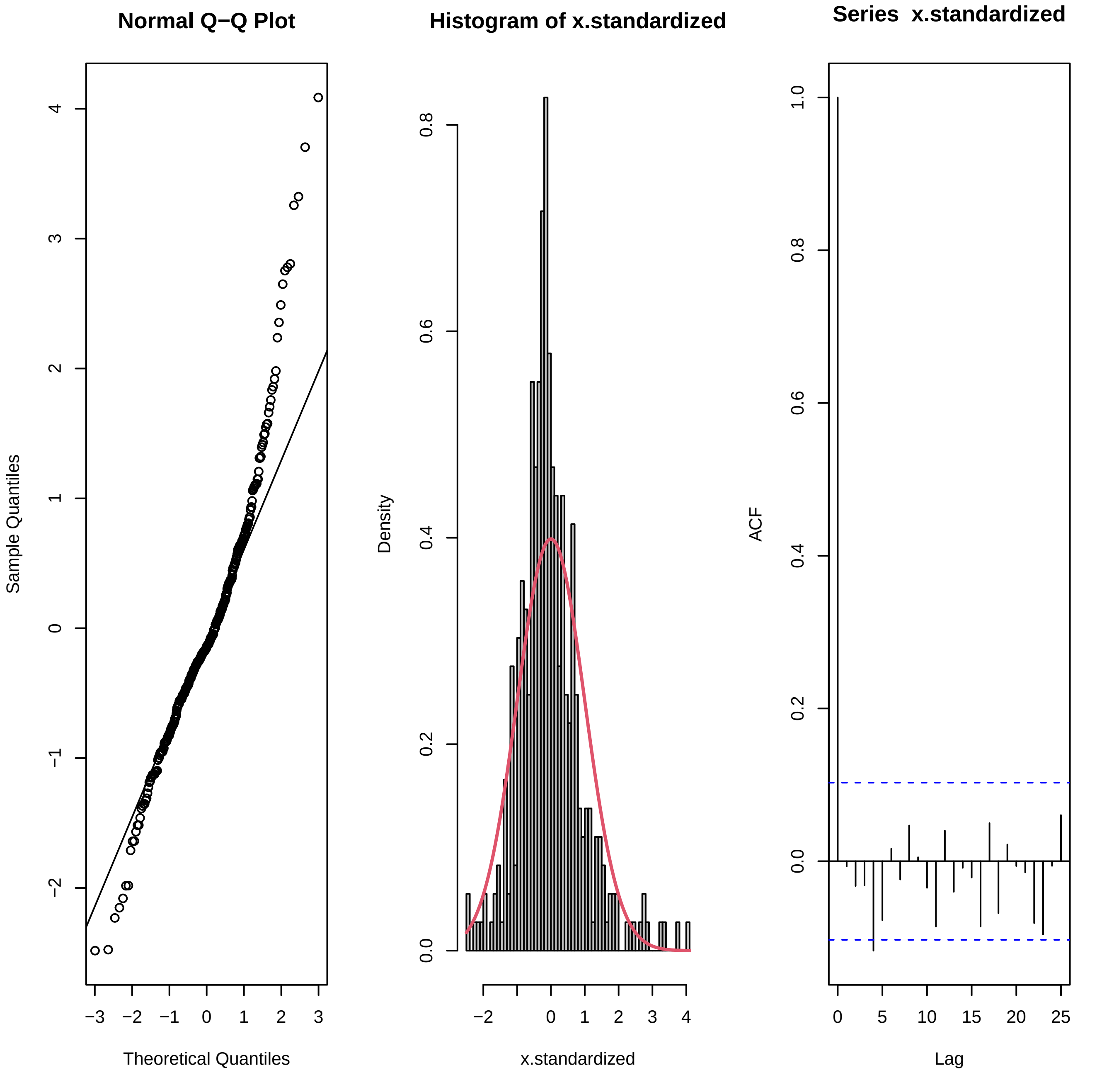
Normal Q−Q Plot
Sample Quantiles
Theoretical Quantiles
−3 −2 −1 0 1 2 3
−2 −1 0 1 2 3 4
Histogram of x.standardized
Density
x.standardized
0.0 0.2 0.4 0.6 0.8
−2 0 1 2 3 4
Series x.standardized
ACF
Lag
0.0 0.2 0.4 0.6 0.8 1.0
0 5 10 15 20 25

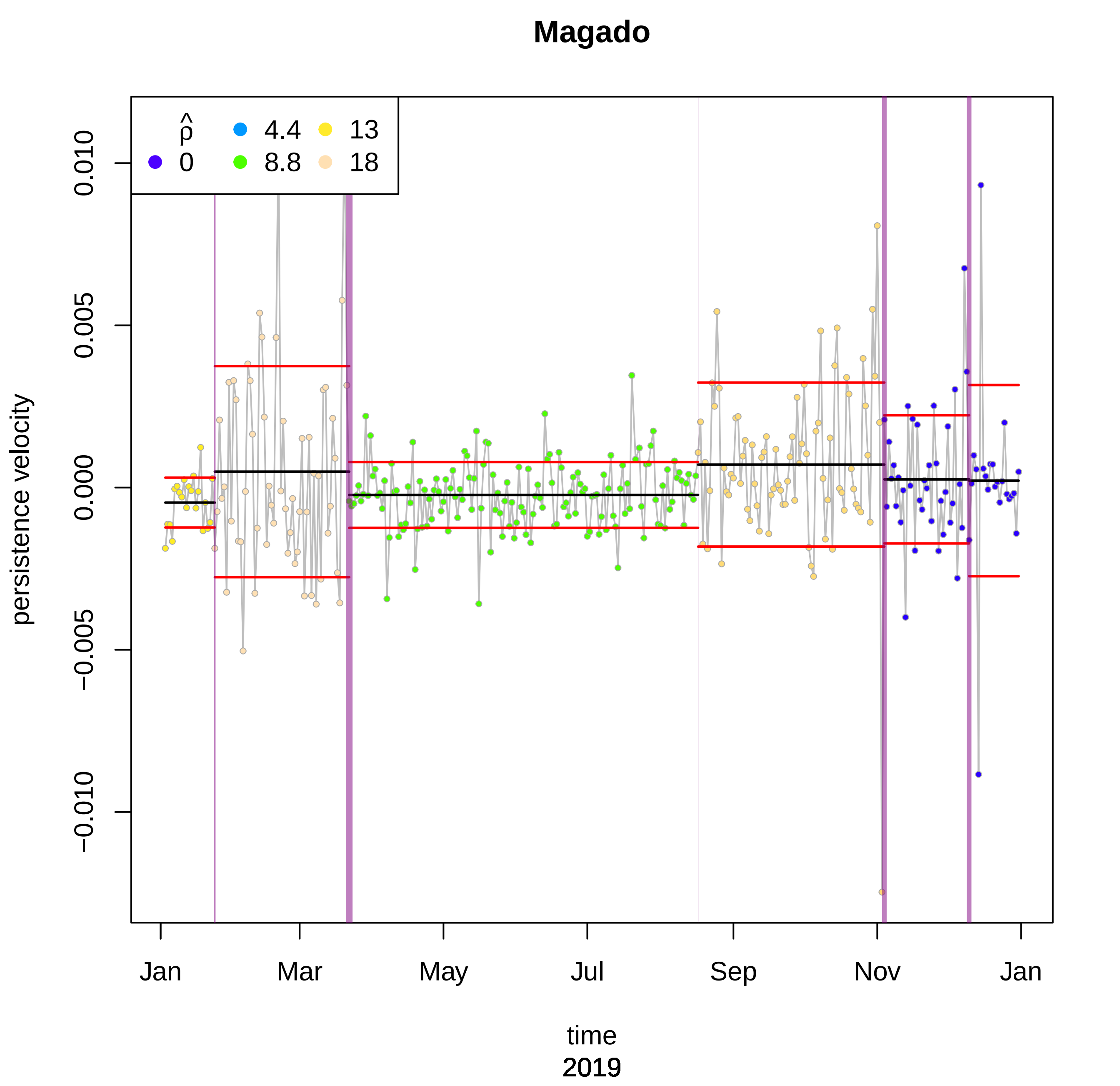

Magado
ρ̂
0
4.4
8.8
13
18
persistence velocity
0.010
0.005
0.000
−0.005
−0.010
Jan
Mar
May
Jul
Sep
Nov
Jan
time
2019

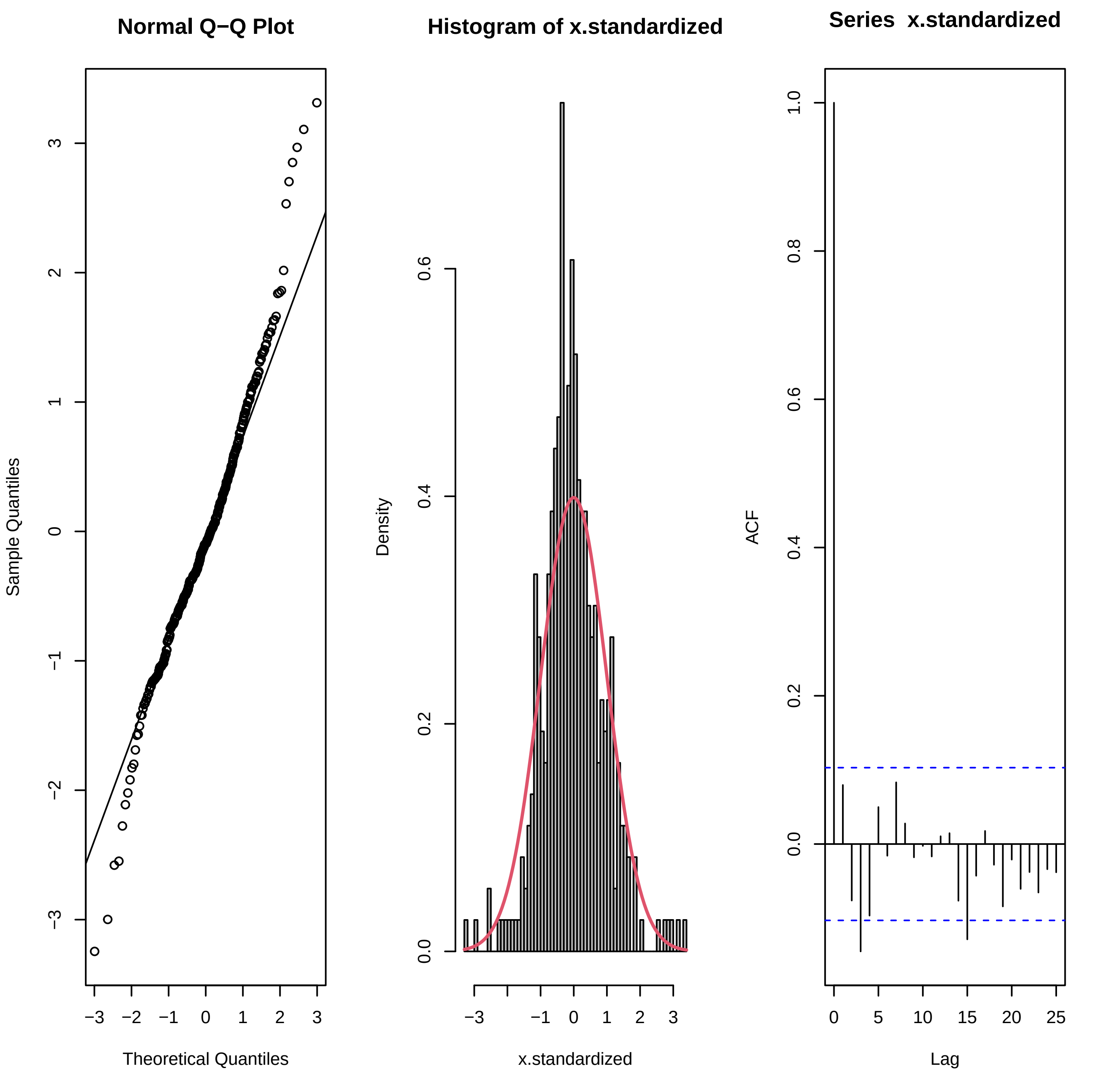

Normal Q−Q Plot
Sample Quantiles
Theoretical Quantiles
−3
−2
−1
0
1
2
3
Histogram of x.standardized
Density
x.standardized
0.0
0.2
0.4
0.6
Series x.standardized
ACF
Lag
0.0
0.2
0.4
0.6
0.8
1.0
0
5
10
15
20
25

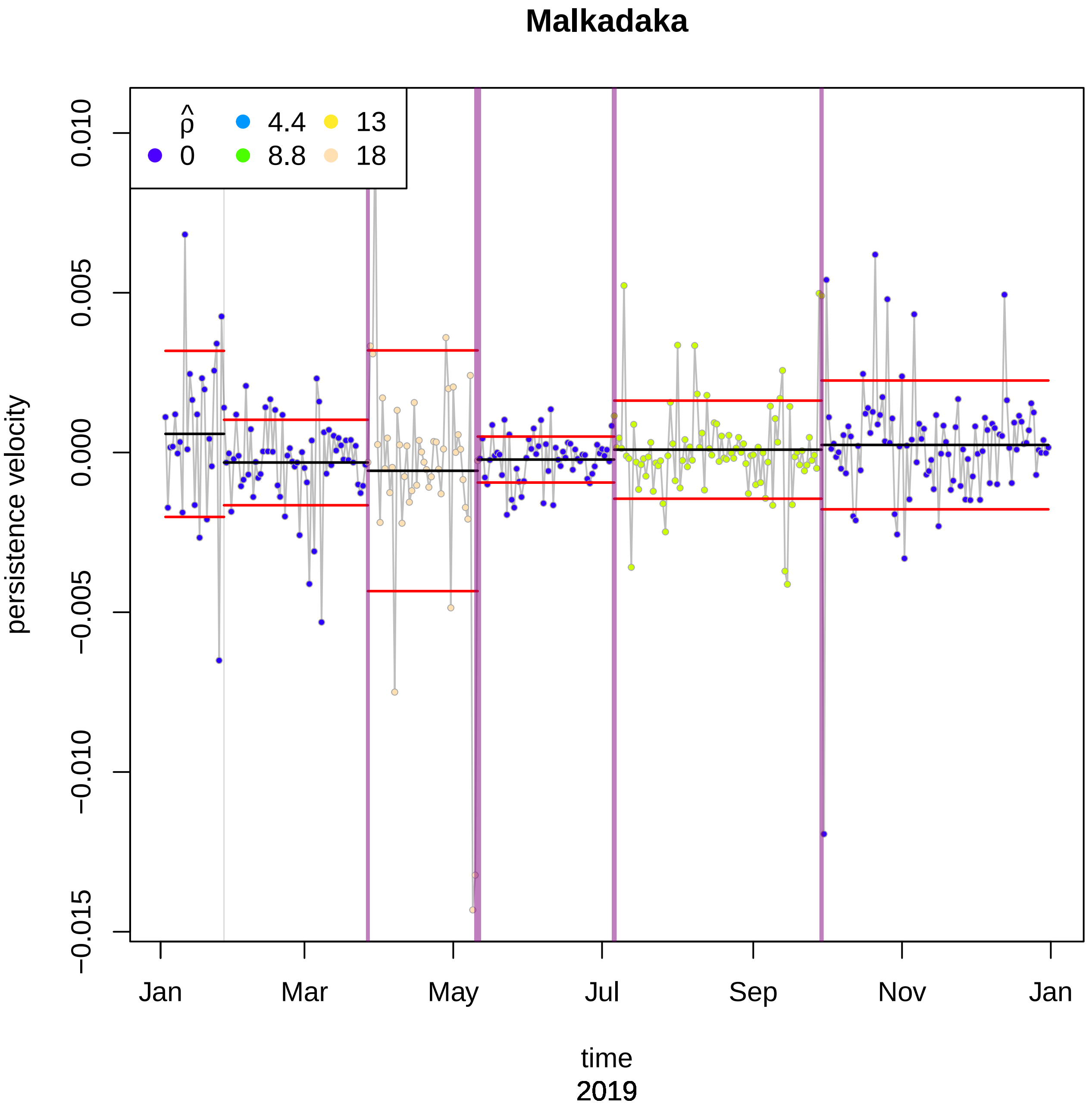

Malkadaka
ρ̂
0
4.4
8.8
13
18
0.010
0.005
0.000
−0.005
−0.010
−0.015
persistence velocity
Jan
Mar
May
Jul
Sep
Nov
Jan
time
2019

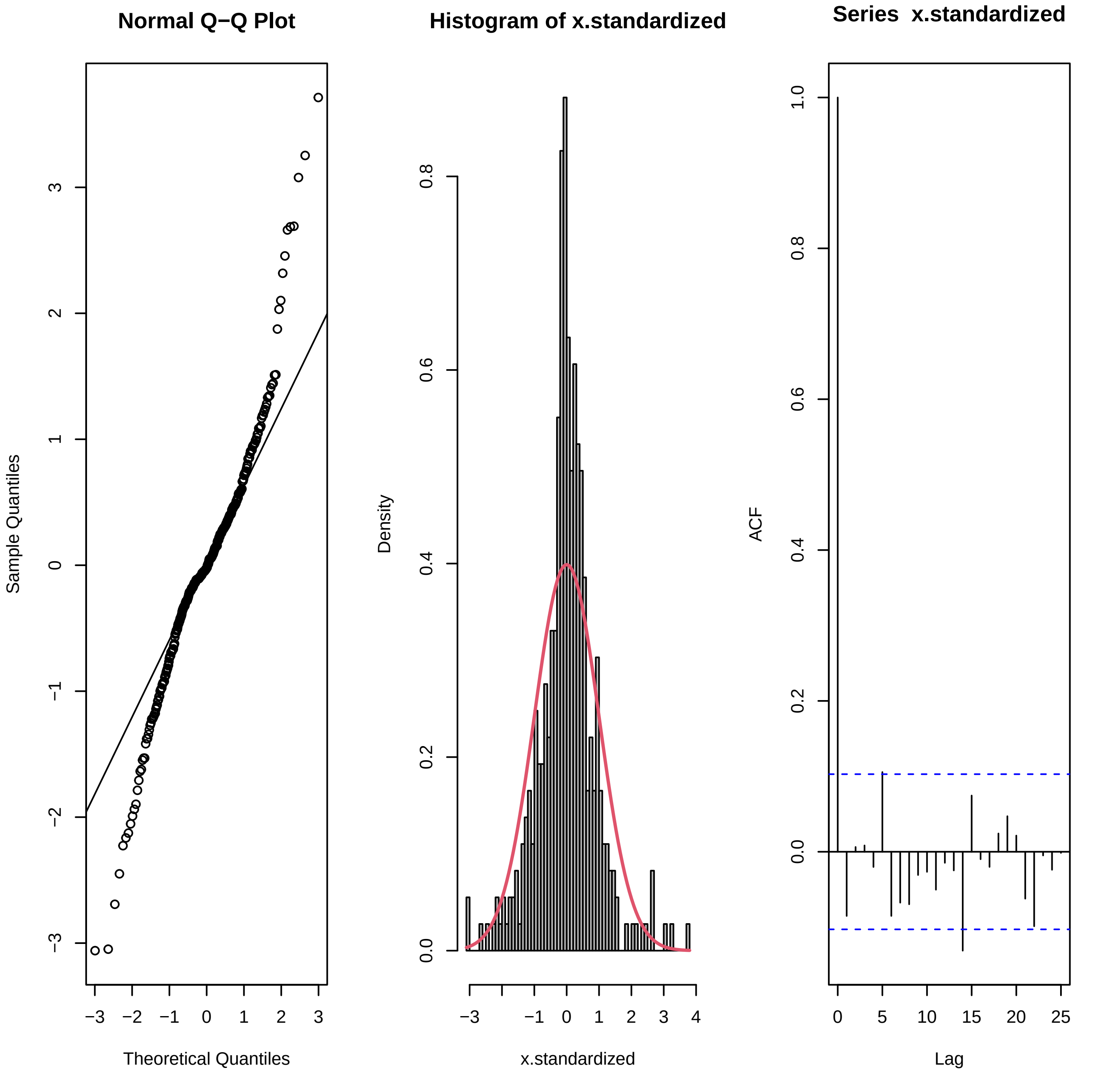

Normal Q−Q Plot
Sample Quantiles
Theoretical Quantiles
−3 −2 −1 0 1 2 3
Histogram of x.standardized
Density
x.standardized
0.0 0.2 0.4 0.6 0.8
−3 −1 0 1 2 3 4
Series x.standardized
ACF
Lag
0.0 0.2 0.4 0.6 0.8 1.0
0 5 10 15 20 25

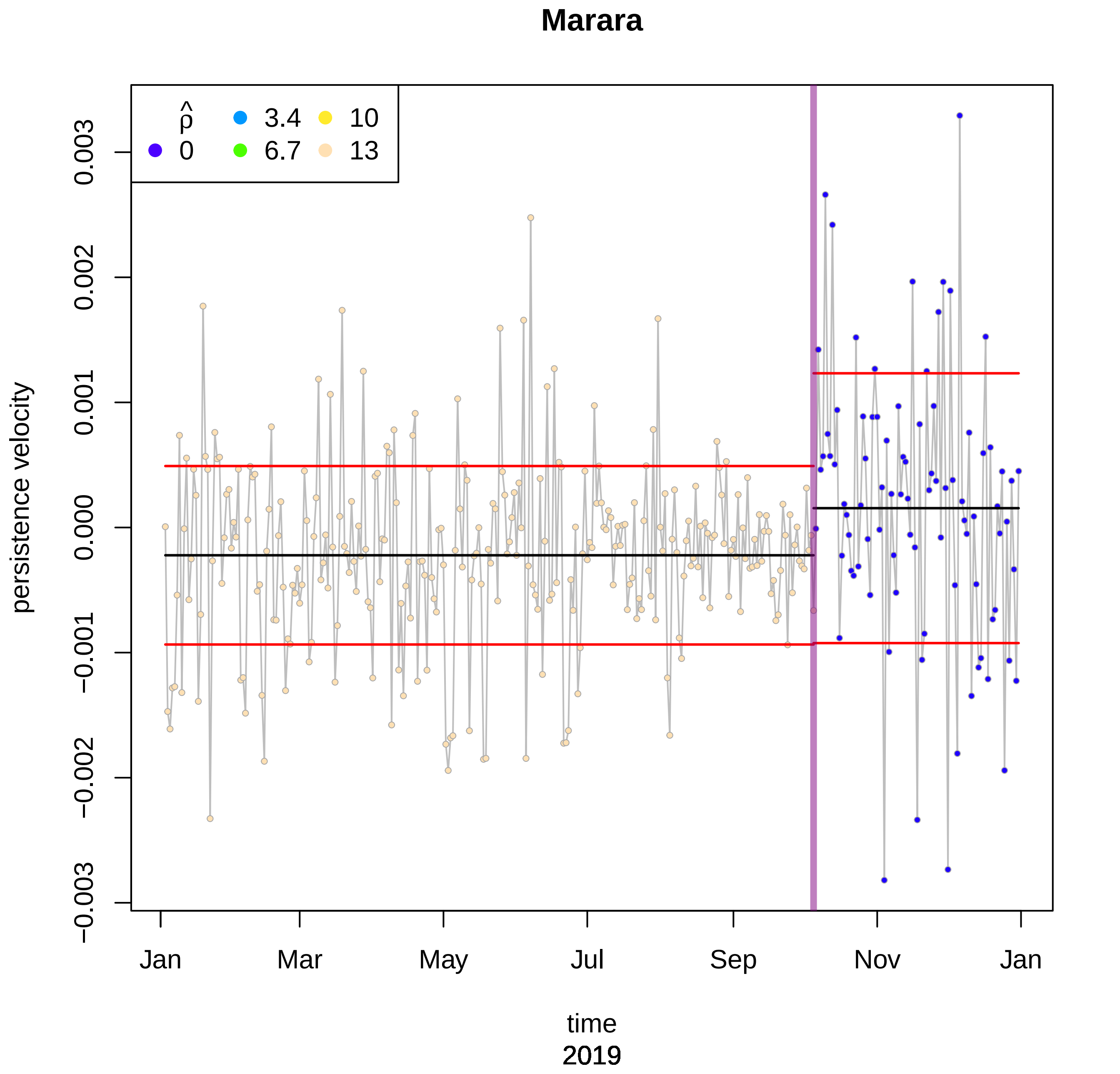

Marara
ρ̂
0
3.4
6.7
10
13
persistence velocity
0.003
0.002
0.001
0.000
−0.001
−0.002
−0.003
Jan
Mar
May
Jul
Sep
Nov
Jan
time
2019

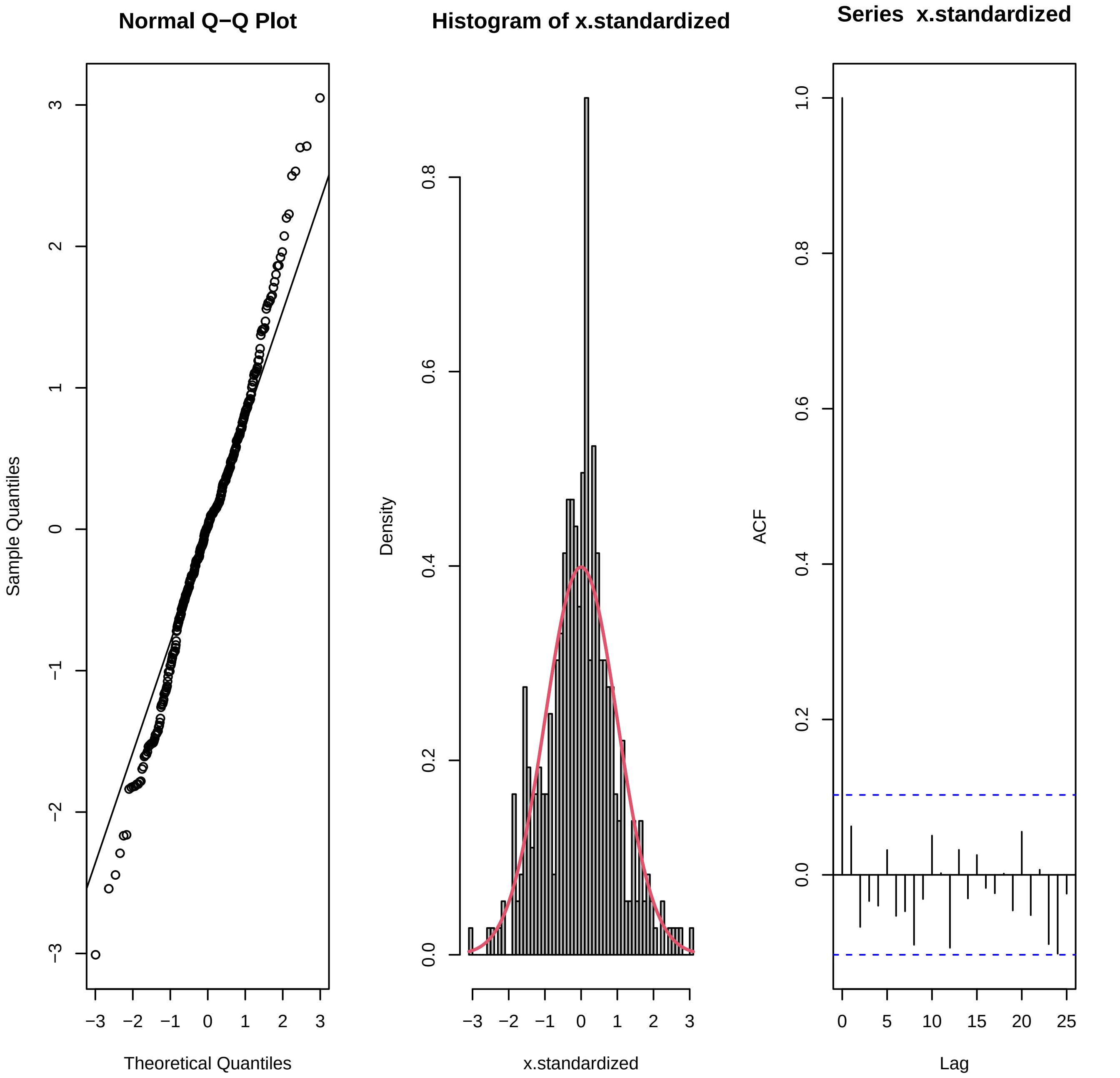

Normal Q−Q Plot
Sample Quantiles
Theoretical Quantiles
Histogram of x.standardized
Density
x.standardized
Series x.standardized
ACF
Lag

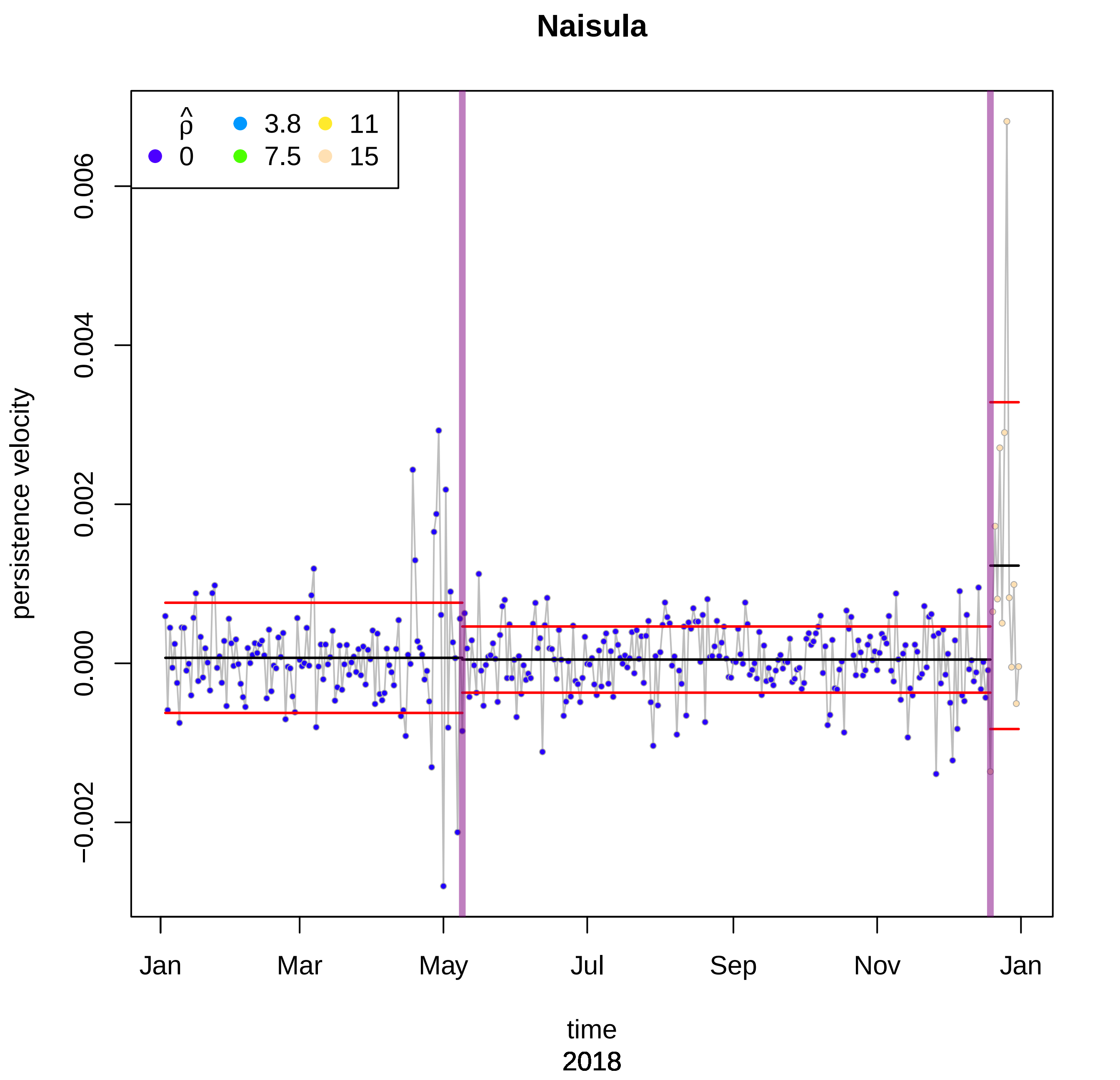

Naisula
ρ̂
0
3.8
7.5
11
15
persistence velocity
−0.002
0.000
0.002
0.004
0.006
Jan
Mar
May
Jul
Sep
Nov
Jan
time
2018

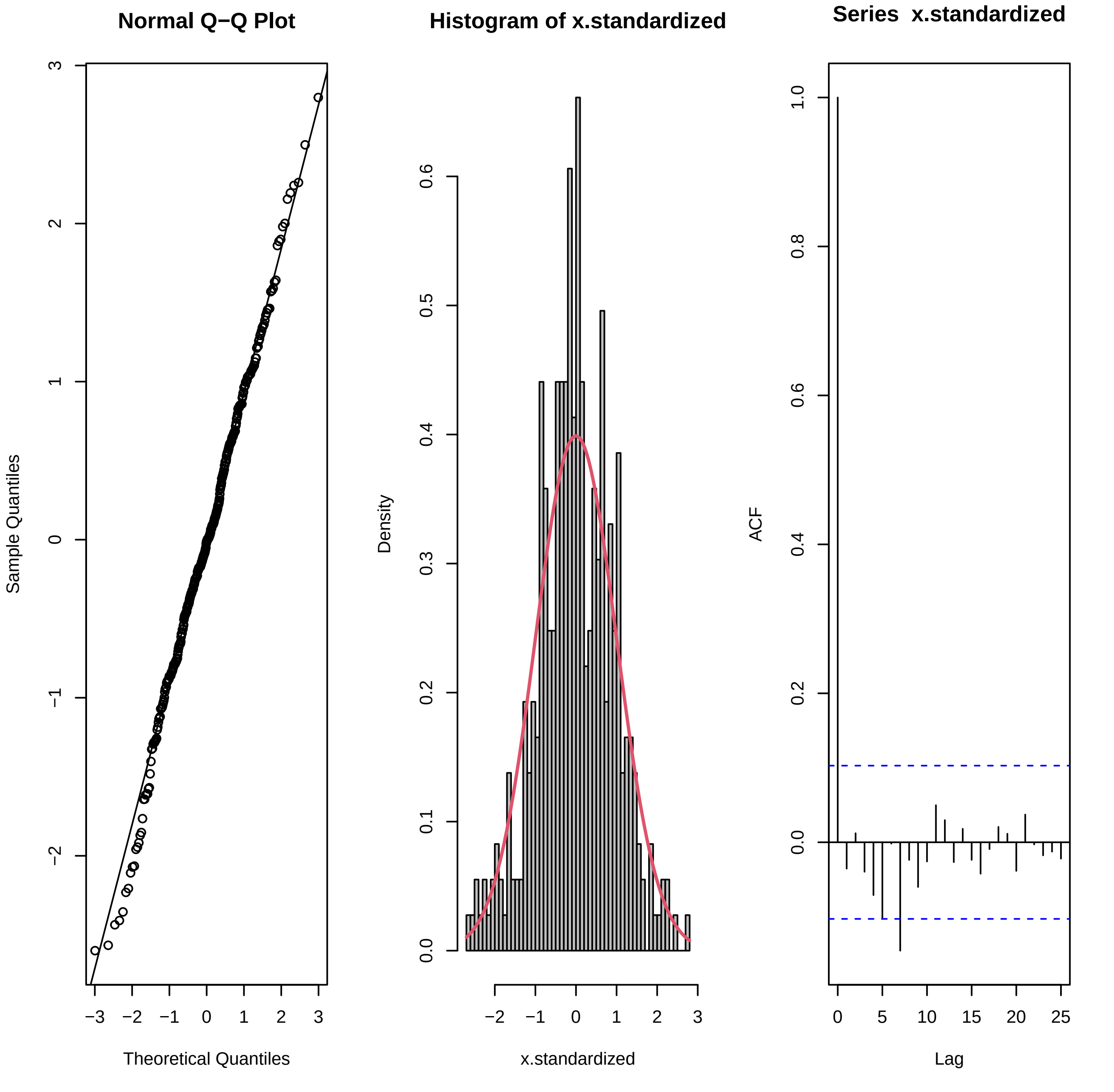

Normal Q−Q Plot
Sample Quantiles
Theoretical Quantiles
3
2
1
0
−1
−2
−3
Histogram of x.standardized
Density
x.standardized
0.0
0.1
0.2
0.3
0.4
0.5
0.6
Series x.standardized
ACF
Lag
1.0
0.8
0.6
0.4
0.2
0.0
5
10
15
20
25

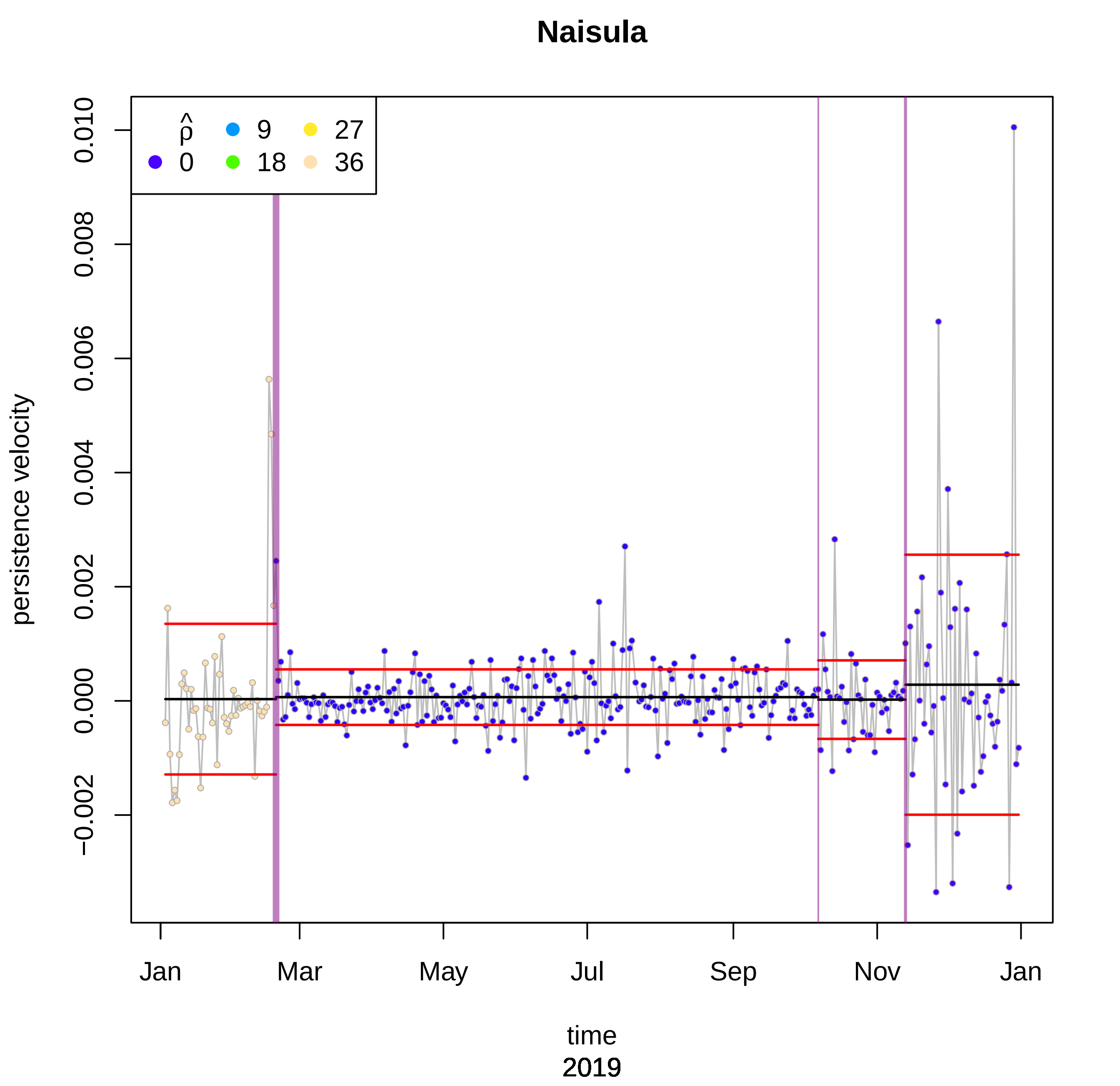

Naisula
ρ̂
0
9
18
27
36
persistence velocity
0.010
0.008
0.006
0.004
0.002
0.000
−0.002
Jan
Mar
May
Jul
Sep
Nov
Jan
time
2019

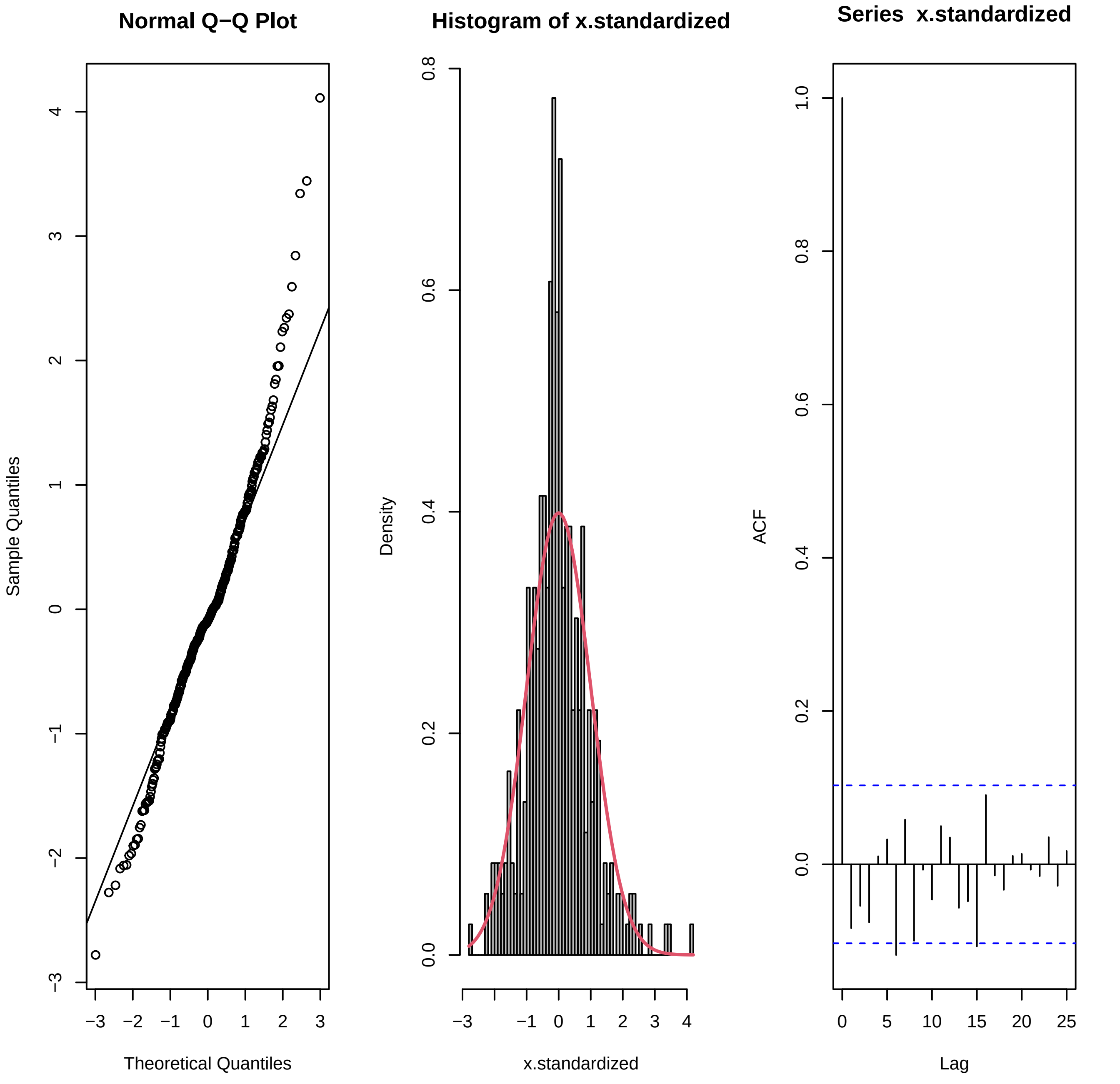
Normal Q−Q Plot
Theoretical Quantiles
Sample Quantiles
Histogram of x.standardized
x.standardized
Density
Series x.standardized
Lag
ACF

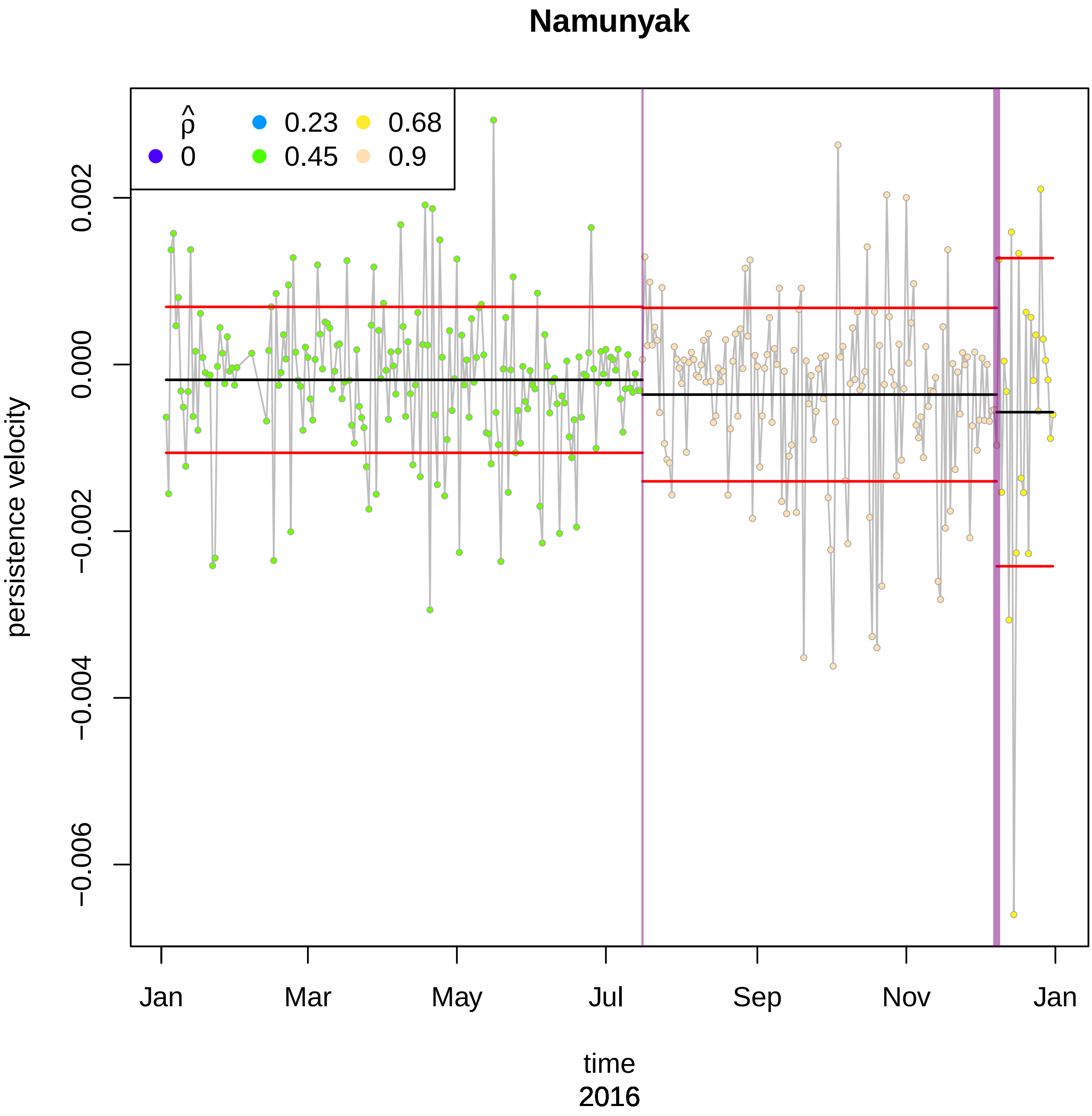

Namunyak
ρ̂
0
0.23
0.45
0.68
0.9
0.002
0.000
−0.002
−0.004
−0.006
persistence velocity
Jan
Mar
May
Jul
Sep
Nov
Jan
time
2016

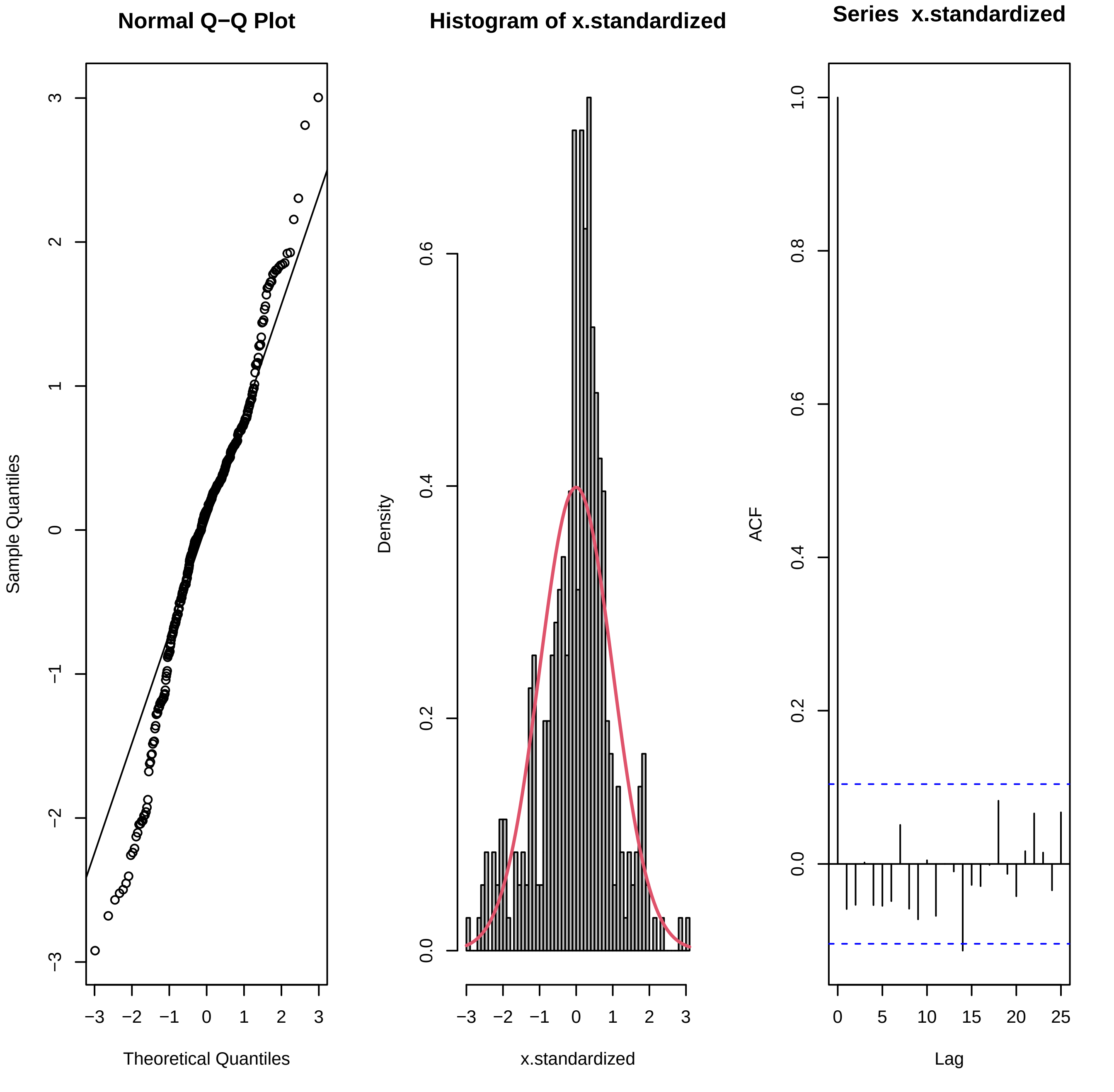

Normal Q−Q Plot
Sample Quantiles
Theoretical Quantiles
−3 −2 −1 0 1 2 3
Histogram of x.standardized
Density
x.standardized
0.0 0.2 0.4 0.6
Series x.standardized
ACF
Lag
0.0 0.2 0.4 0.6 0.8 1.0
0 5 10 15 20 25

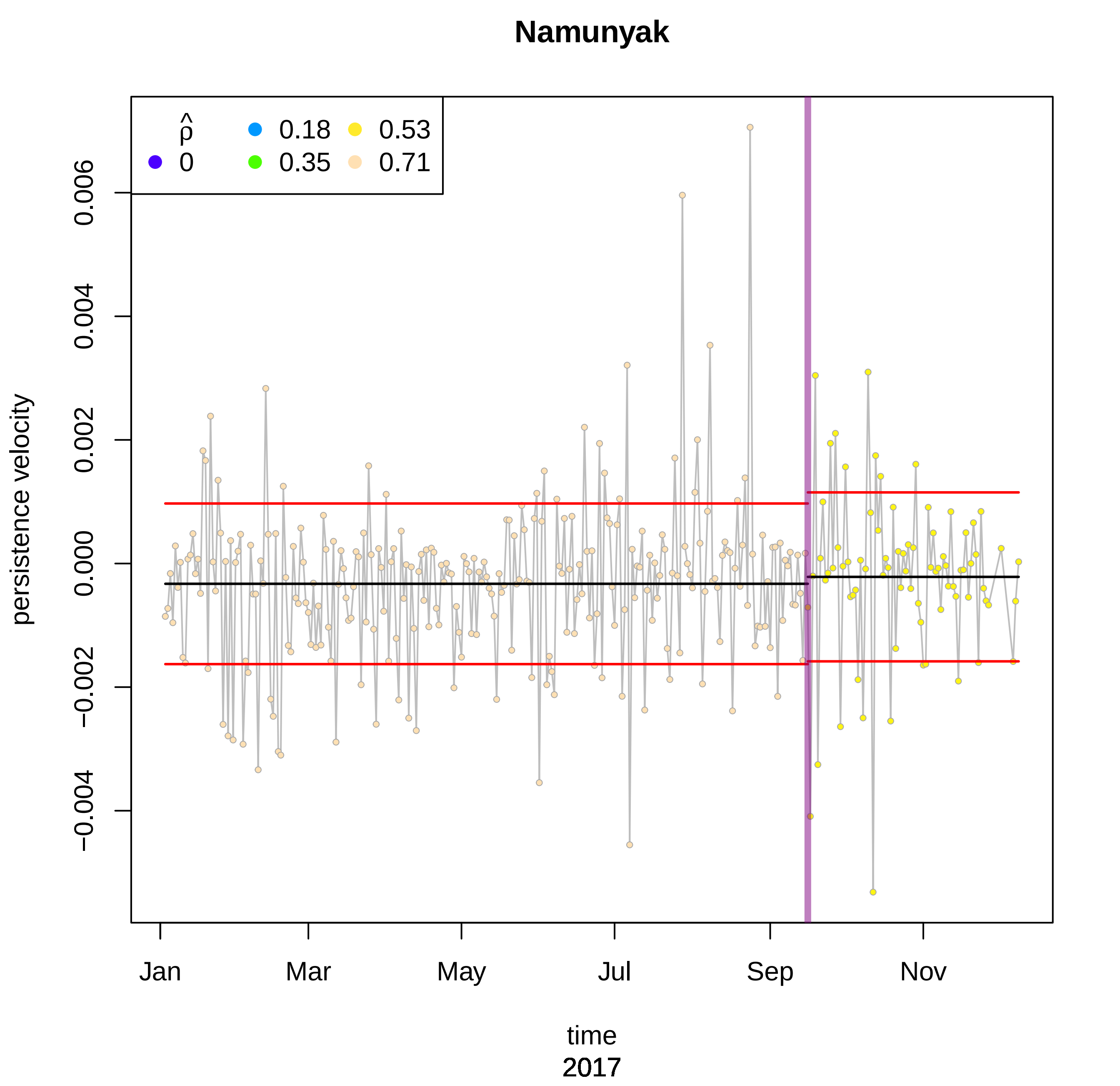

Namunyak
ρ̂
0
0.18
0.35
0.53
0.71
persistence velocity
0.006
0.004
0.002
0.000
−0.002
−0.004
Jan
Mar
May
Jul
Sep
Nov
time
2017

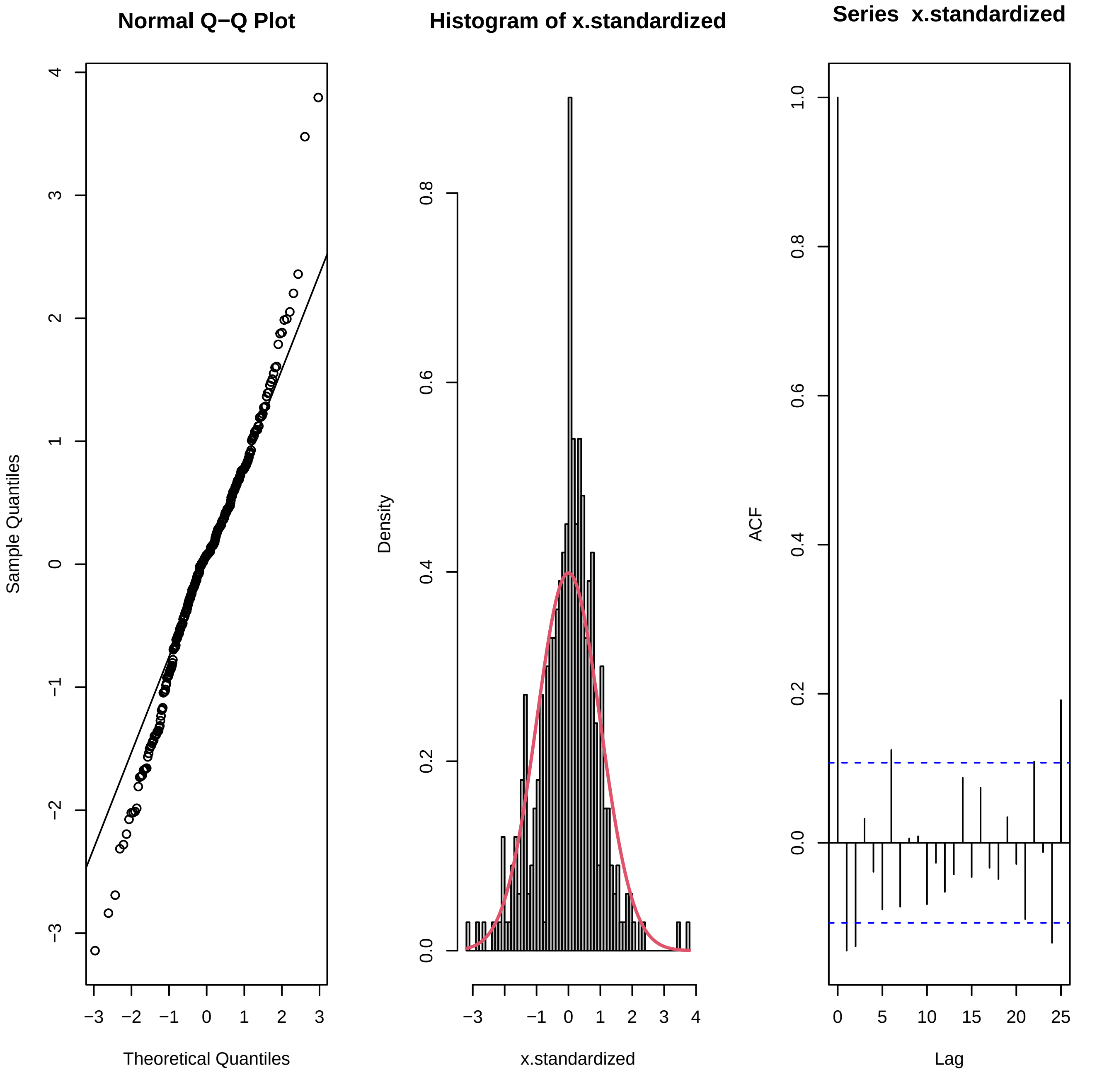

Normal Q−Q Plot
Sample Quantiles
Theoretical Quantiles
Histogram of x.standardized
Density
x.standardized
Series x.standardized
ACF
Lag

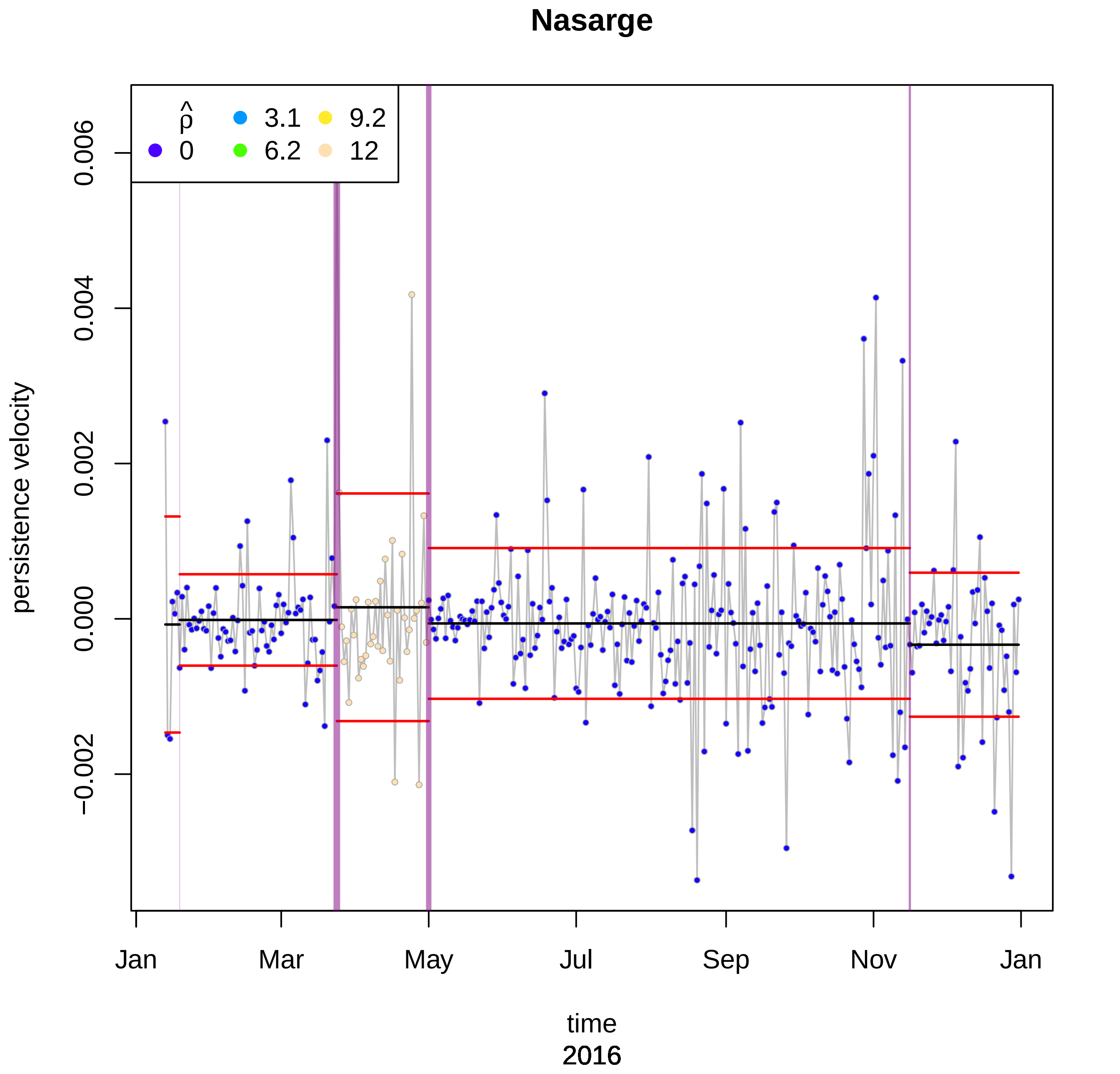

Nasarge
ρ̂
0
3.1
6.2
9.2
12
persistence velocity
−0.002
0.000
0.002
0.004
0.006
Jan
Mar
May
Jul
Sep
Nov
Jan
time
2016

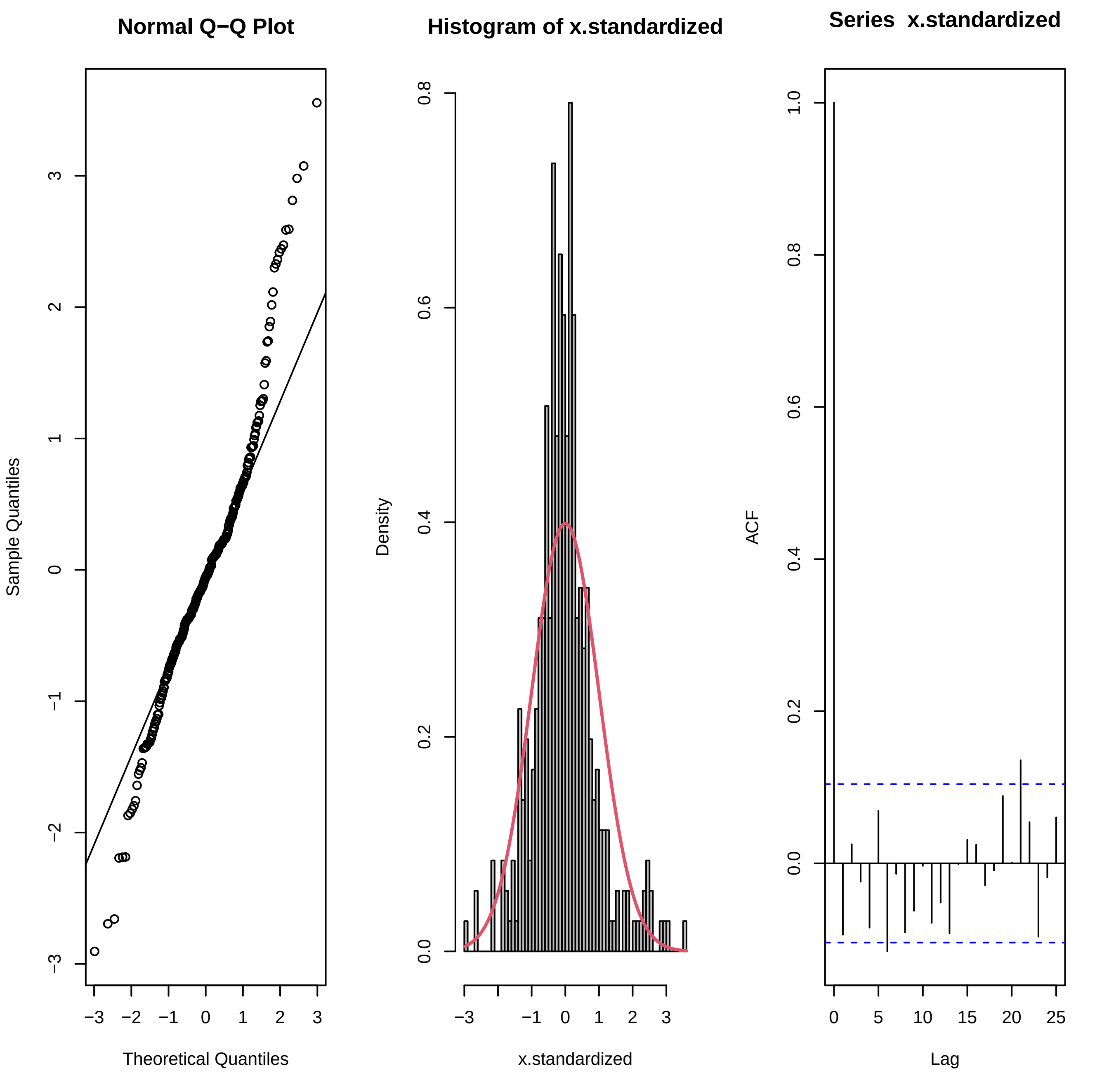

Normal Q−Q Plot
Sample Quantiles
Theoretical Quantiles
−3
−2
−1
0
1
2
3
Histogram of x.standardized
Density
x.standardized
0.0
0.2
0.4
0.6
0.8
Series x.standardized
ACF
Lag
0
5
10
15
20
25
1.0

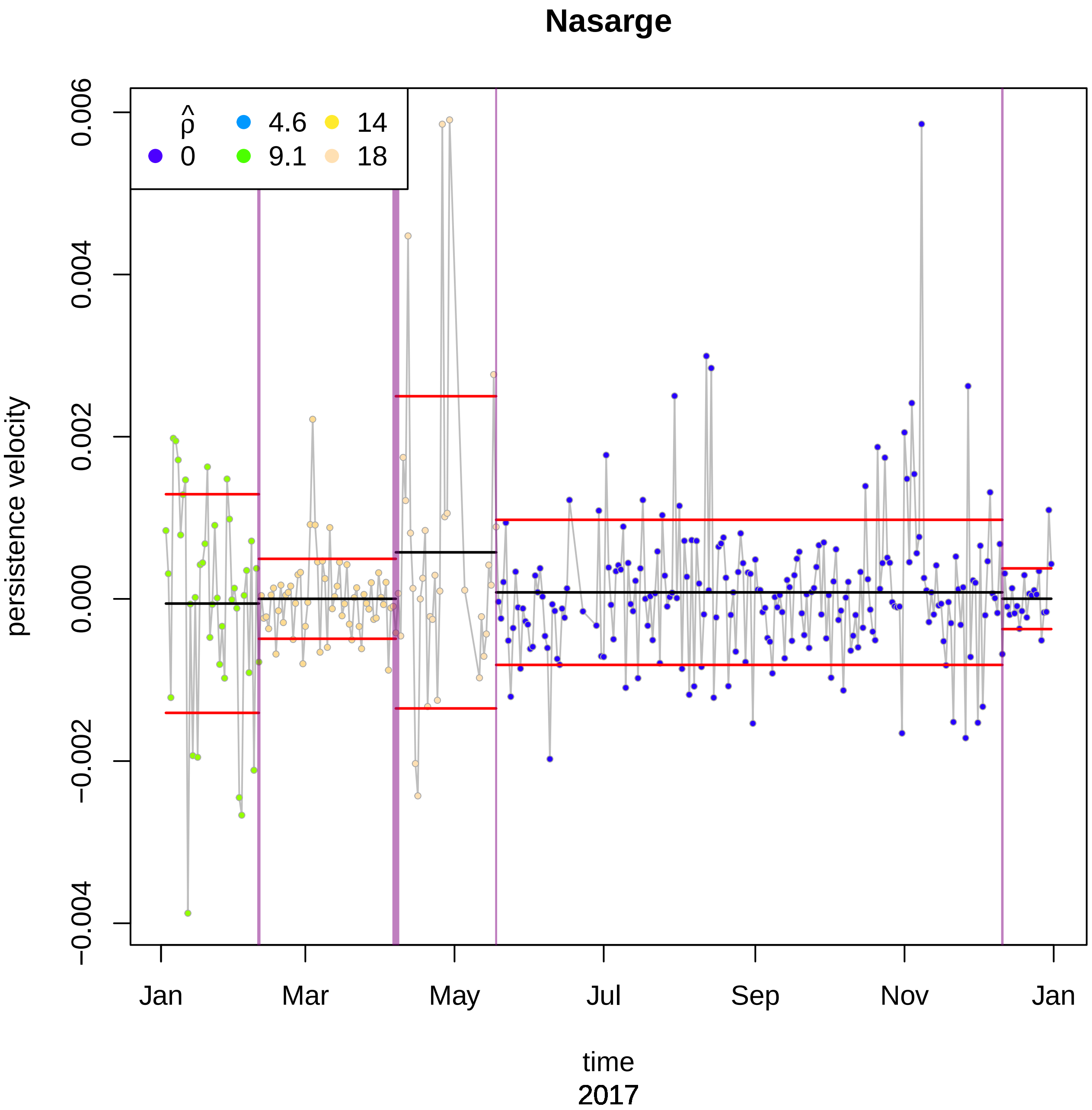

Nasarge
ρ̂
0
4.6
9.1
14
18
persistence velocity
0.006
0.004
0.002
0.000
−0.002
−0.004
Jan
Mar
May
Jul
Sep
Nov
Jan
time
2017

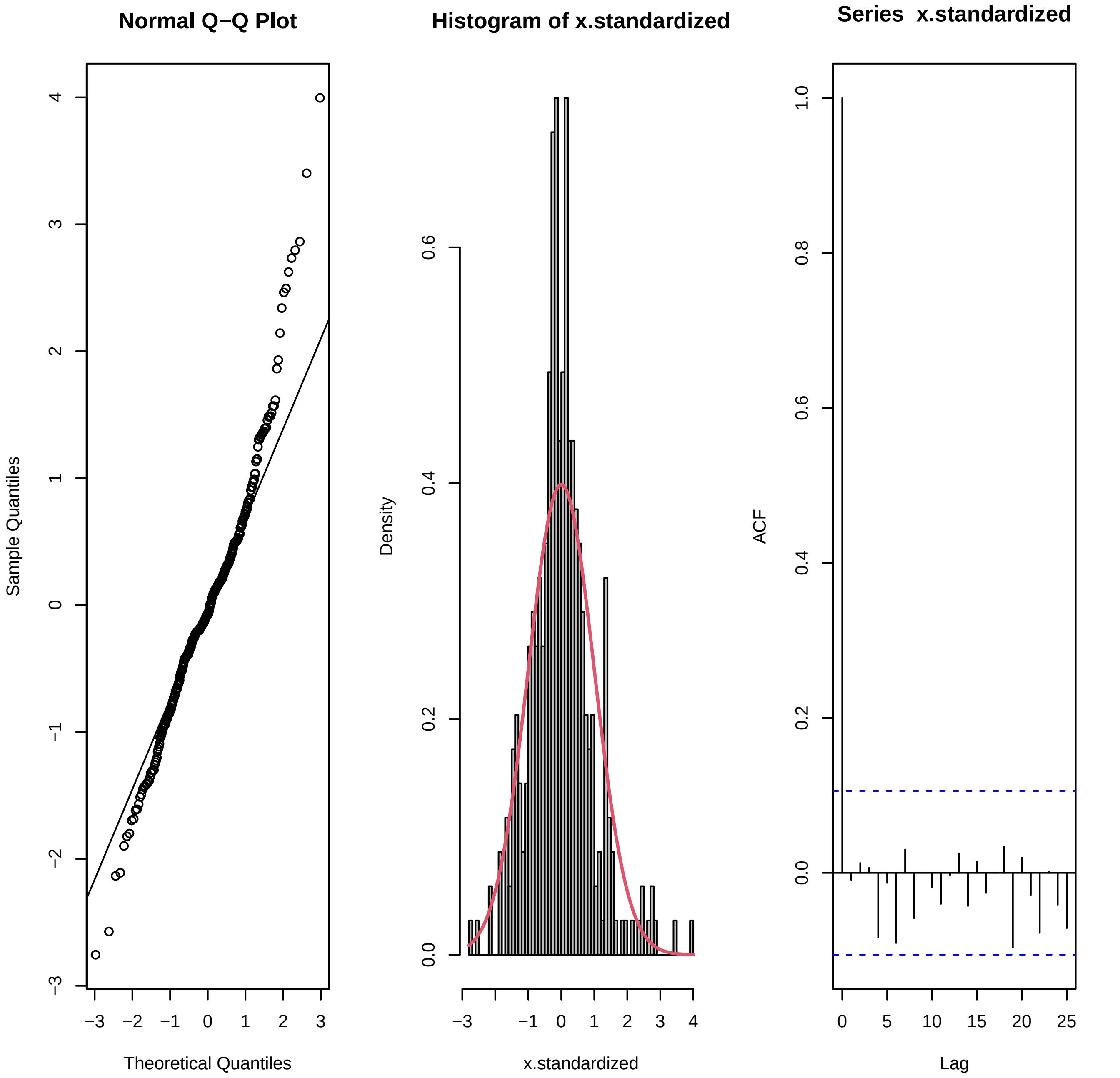
Normal Q−Q Plot
Sample Quantiles
Theoretical Quantiles
−3
−2
−1
0
1
2
3
4
Histogram of x.standardized
Density
x.standardized
0.0
0.2
0.4
0.6
Series x.standardized
ACF
Lag
0.0
0.2
0.4
0.6
0.8
1.0
0
5
10
15
20
25

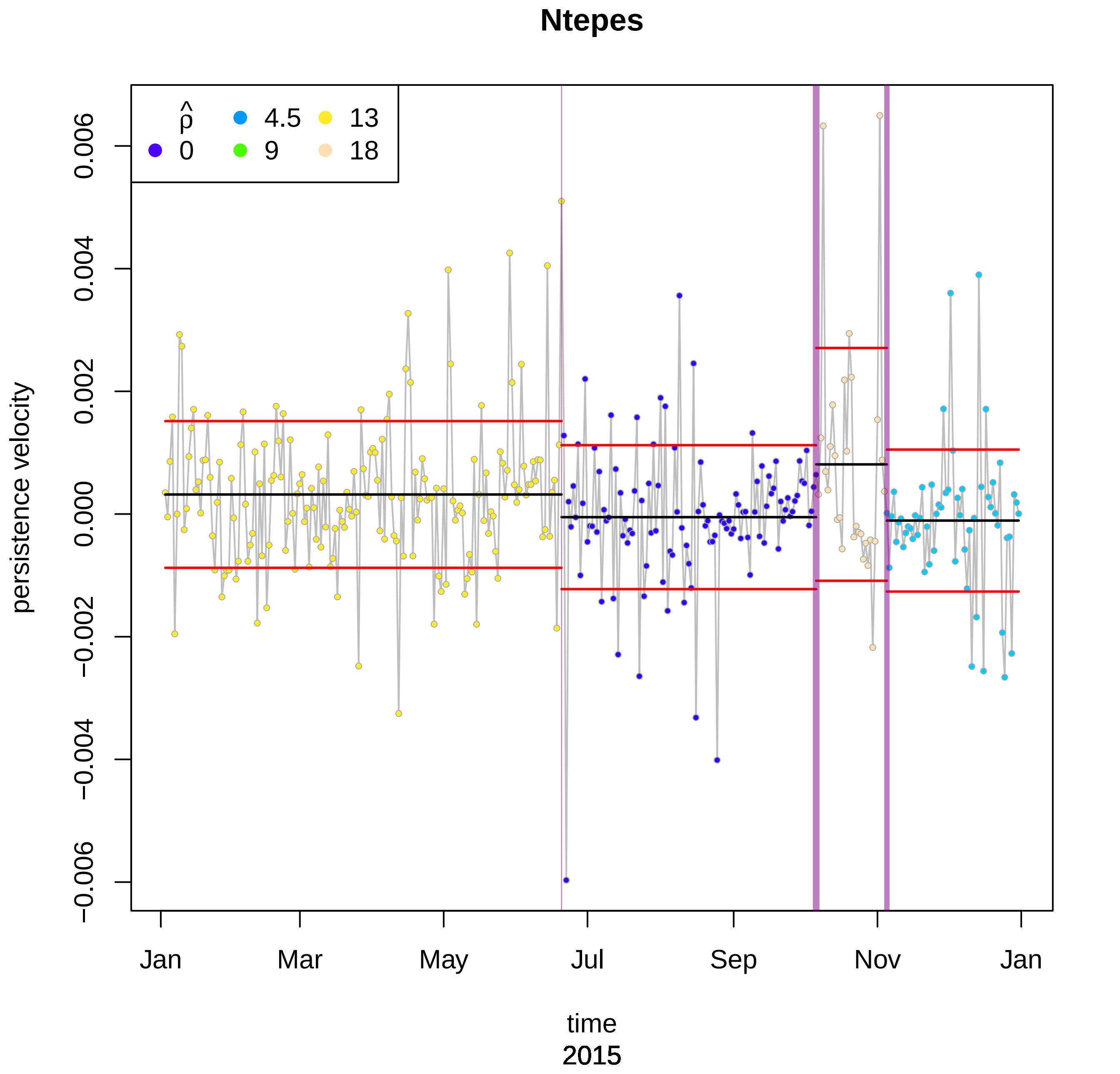

Ntepes
ρ̂
0
4.5
9
13
18
0.006
0.004
0.002
0.000
−0.002
−0.004
−0.006
persistence velocity
Jan
Mar
May
Jul
Sep
Nov
Jan
time
2015

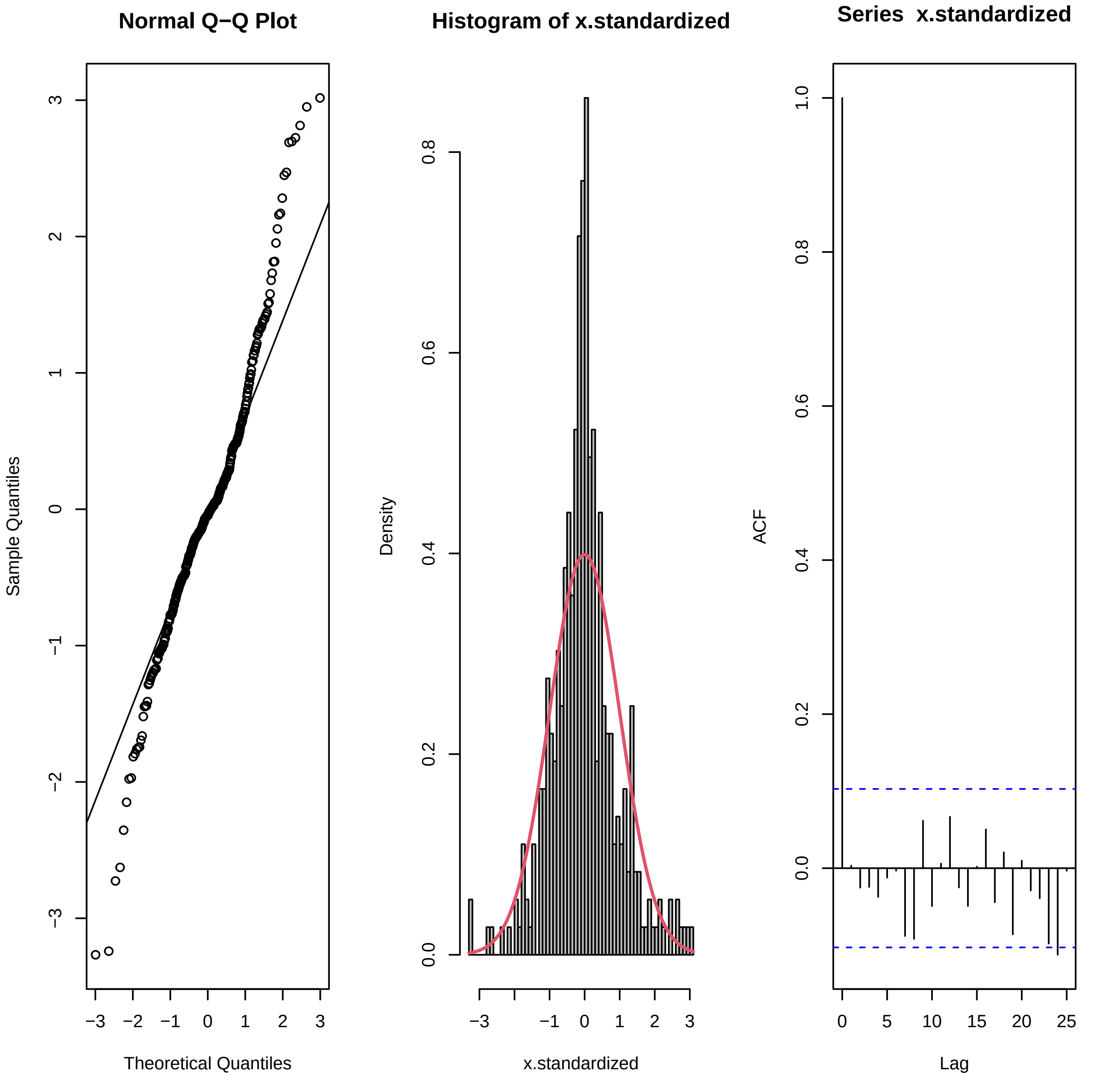

Normal Q−Q Plot
Sample Quantiles
Theoretical Quantiles
−3
−2
−1
0
1
2
3
Histogram of x.standardized
Density
x.standardized
0.0
0.2
0.4
0.6
0.8
Series x.standardized
ACF
Lag
0
5
10
15
20
25
1.0

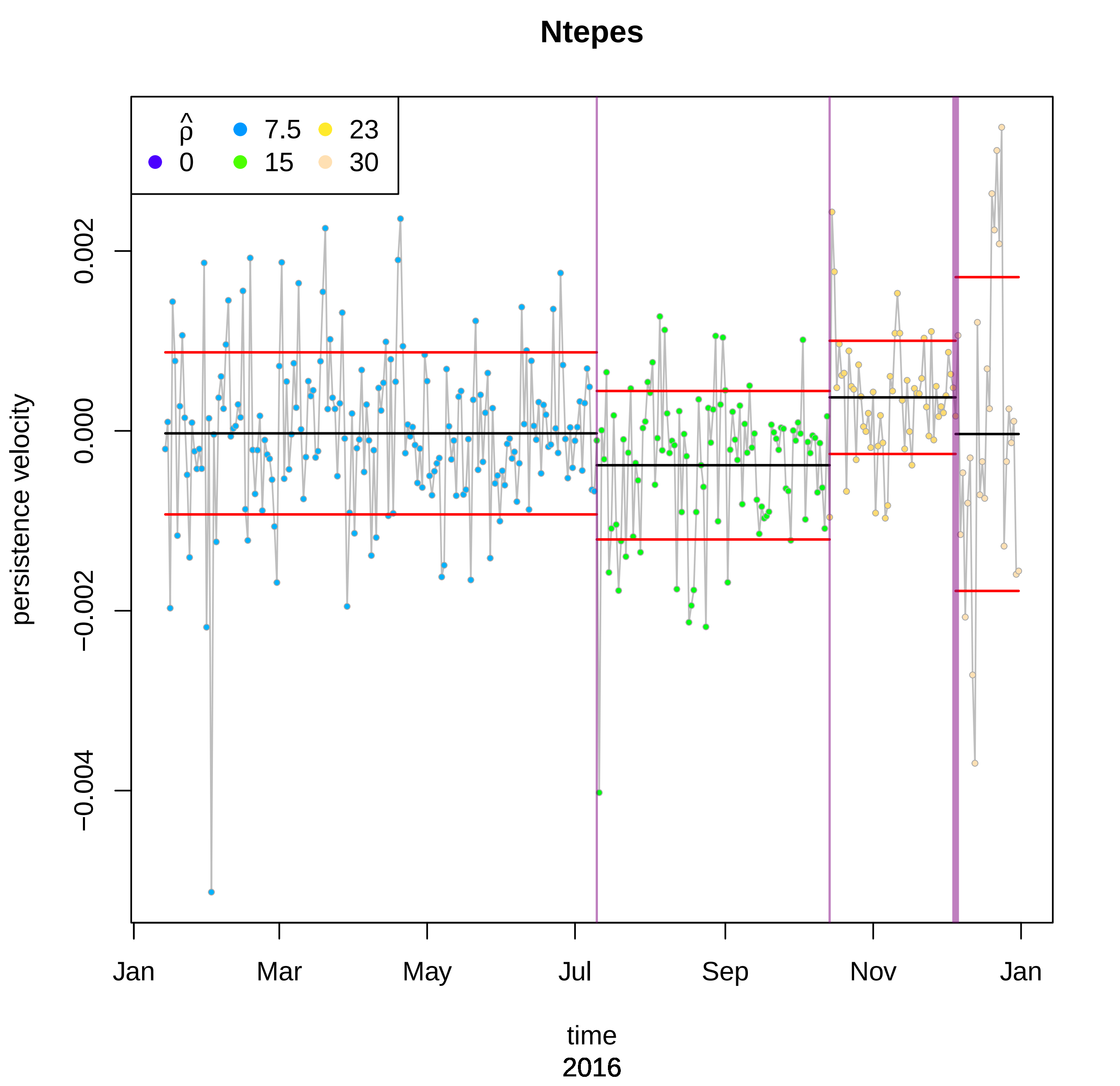

Ntepes
ρ̂
0
7.5
15
23
30
persistence velocity
0.002
0.000
−0.002
−0.004
Jan
Mar
May
Jul
Sep
Nov
Jan
time
2016

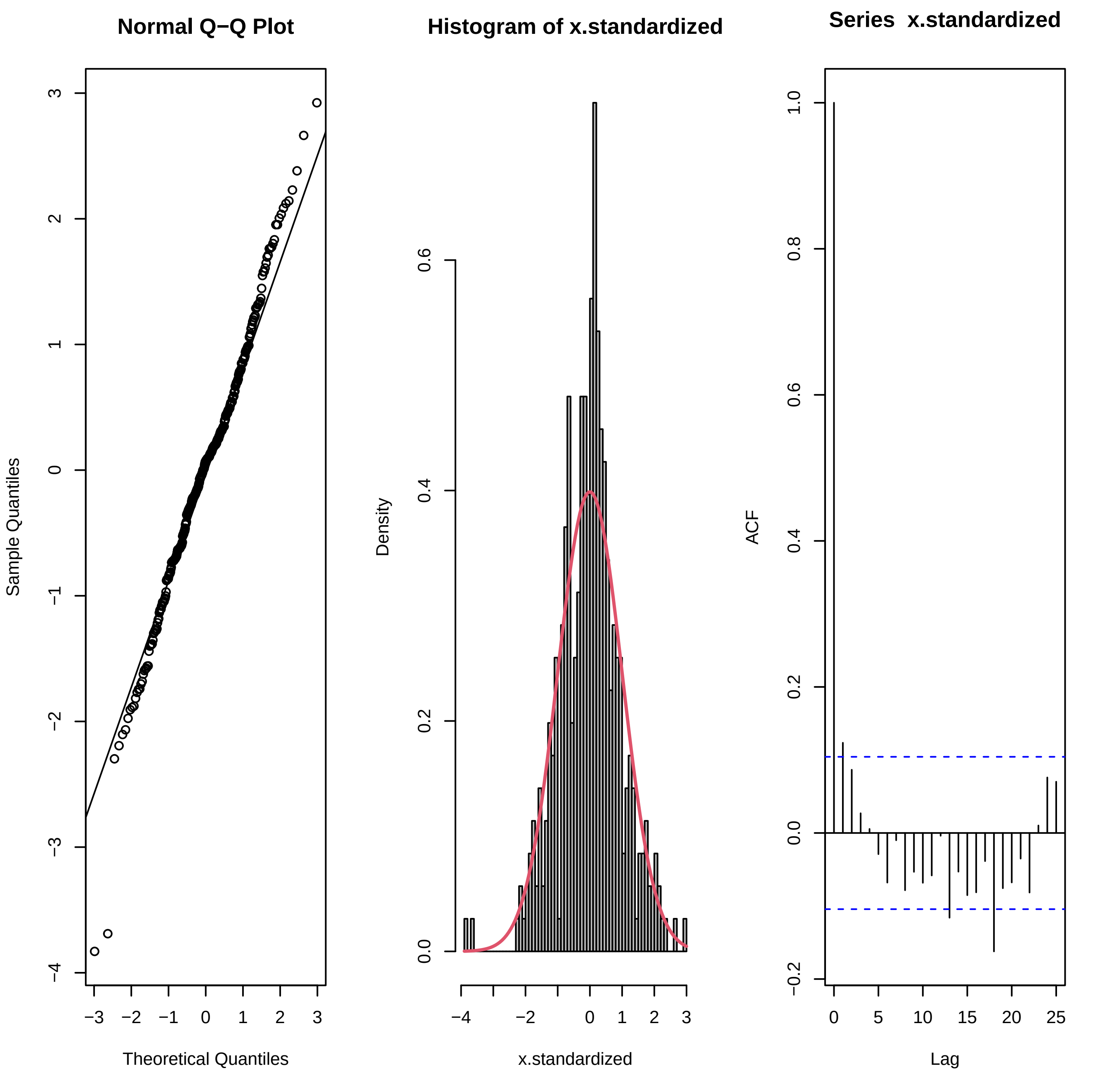

Normal Q−Q Plot
Sample Quantiles
Theoretical Quantiles
−3
−2
−1
0
1
2
3
−4
Histogram of x.standardized
Density
x.standardized
0.0
0.2
0.4
0.6
−4
−2
0
1
2
3
Series x.standardized
ACF
Lag
−0.2
0.0
0.2
0.4
0.6
0.8
1.0
0
5
10
15
20
25

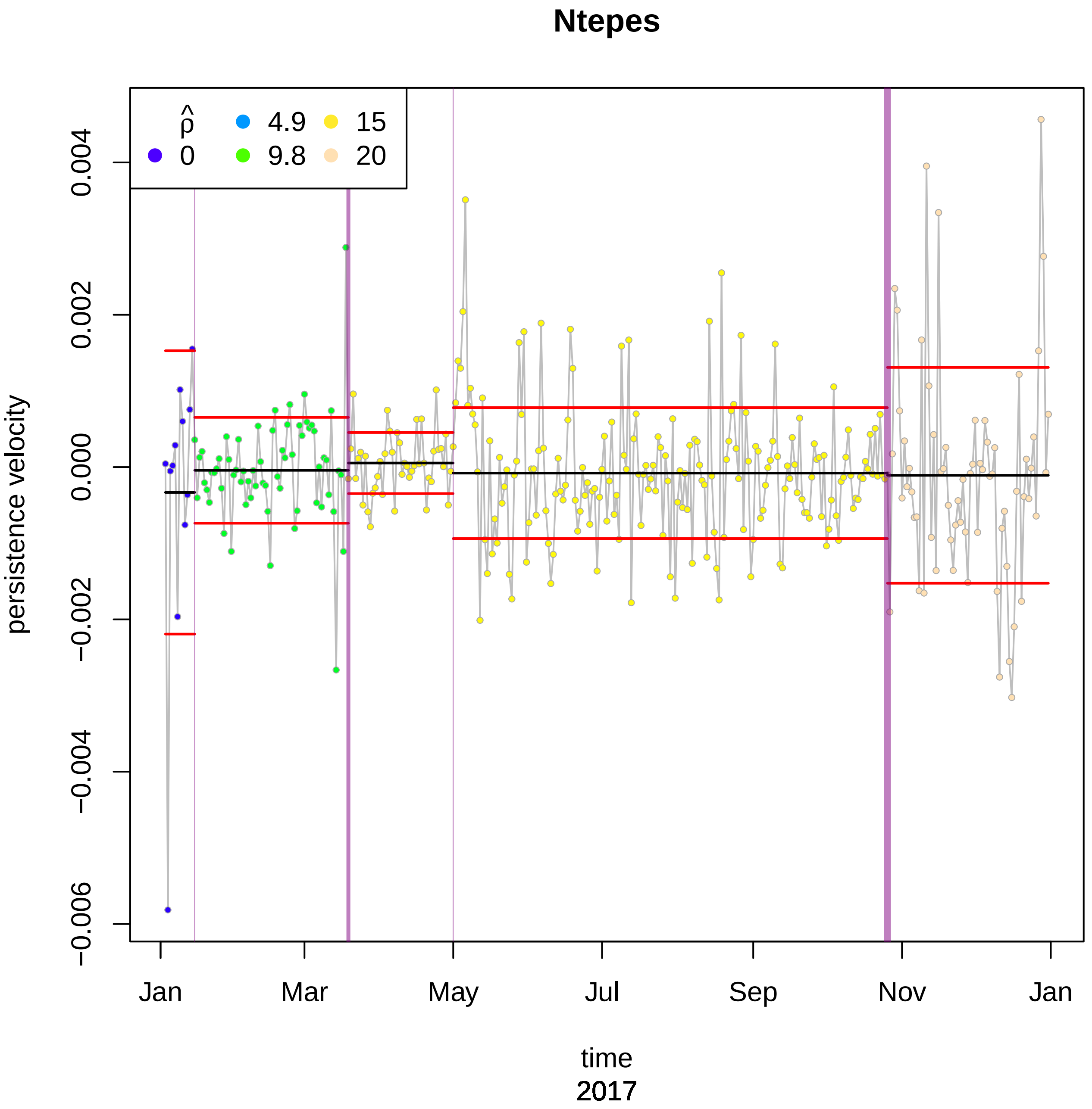
Ntepes
ρ̂
0
4.9
9.8
15
20
persistence velocity
0.004
0.002
0.000
−0.002
−0.004
−0.006
Jan
Mar
May
Jul
Sep
Nov
Jan
time
2017

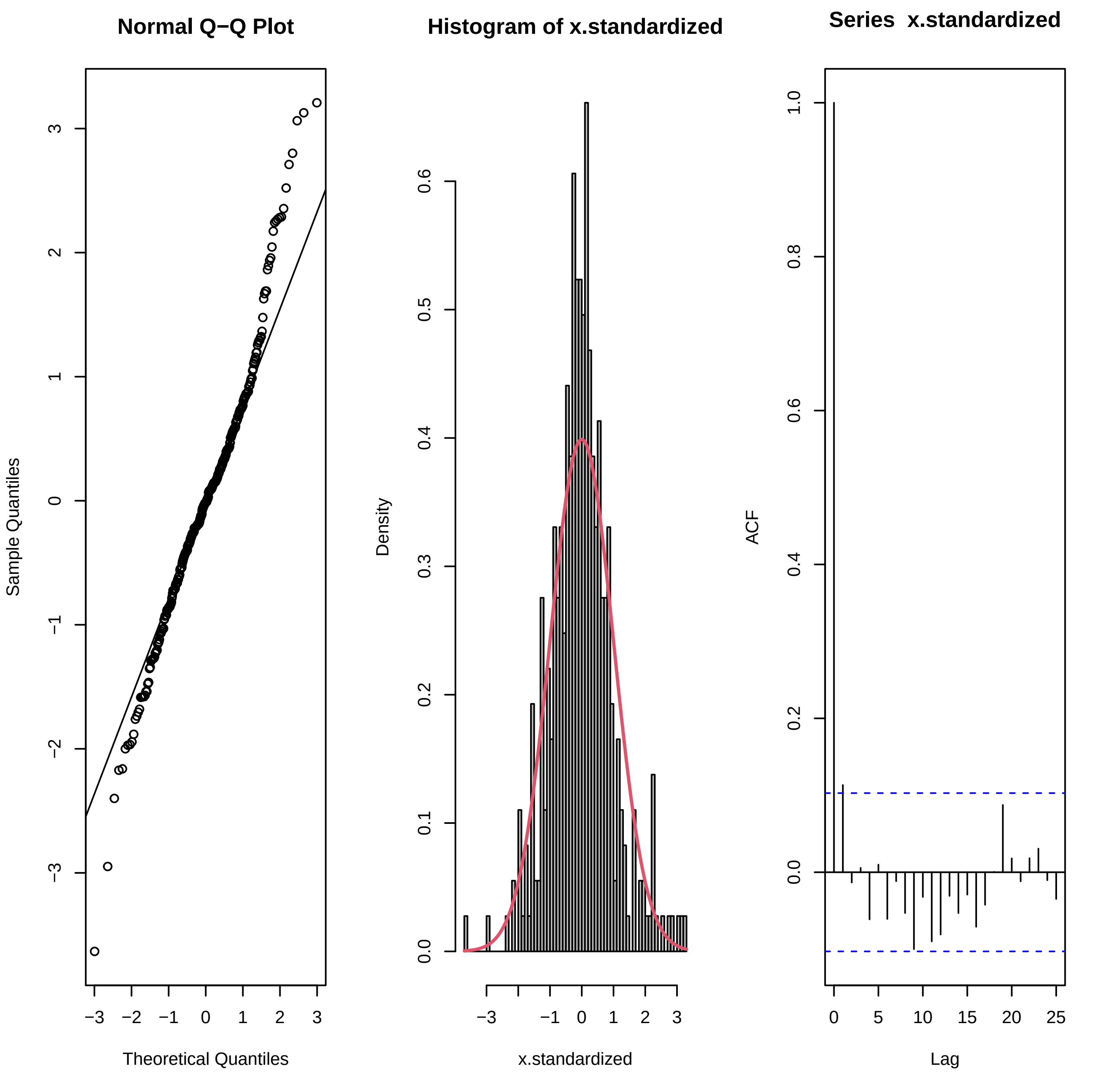
Normal Q−Q Plot
Sample Quantiles
Theoretical Quantiles
Histogram of x.standardized
Density
x.standardized
Series x.standardized
ACF
Lag

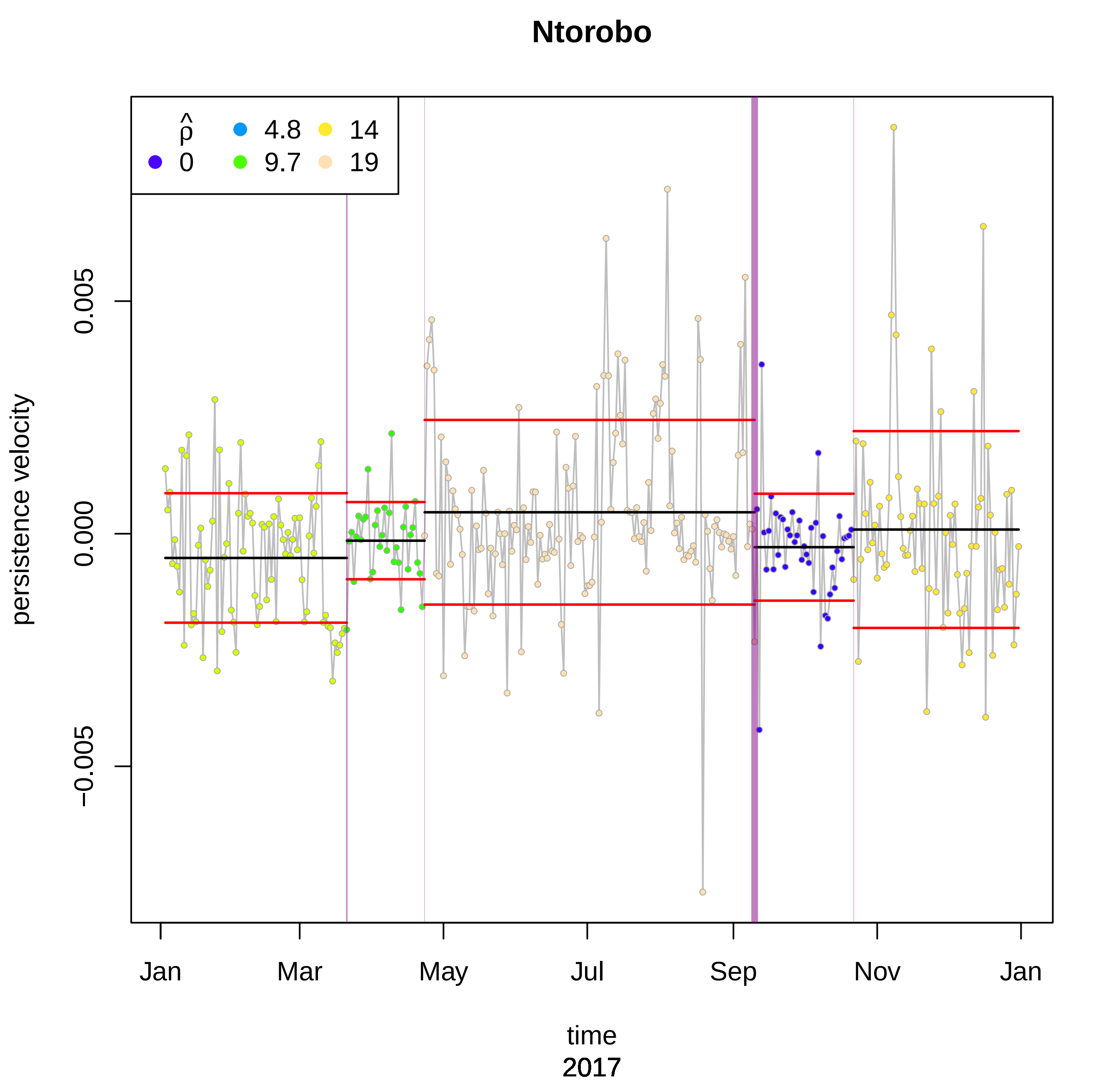
Ntorobo
ρ̂
0
4.8
9.7
14
19
persistence velocity
0.005
0.000
−0.005
Jan
Mar
May
Jul
Sep
Nov
Jan
time
2017

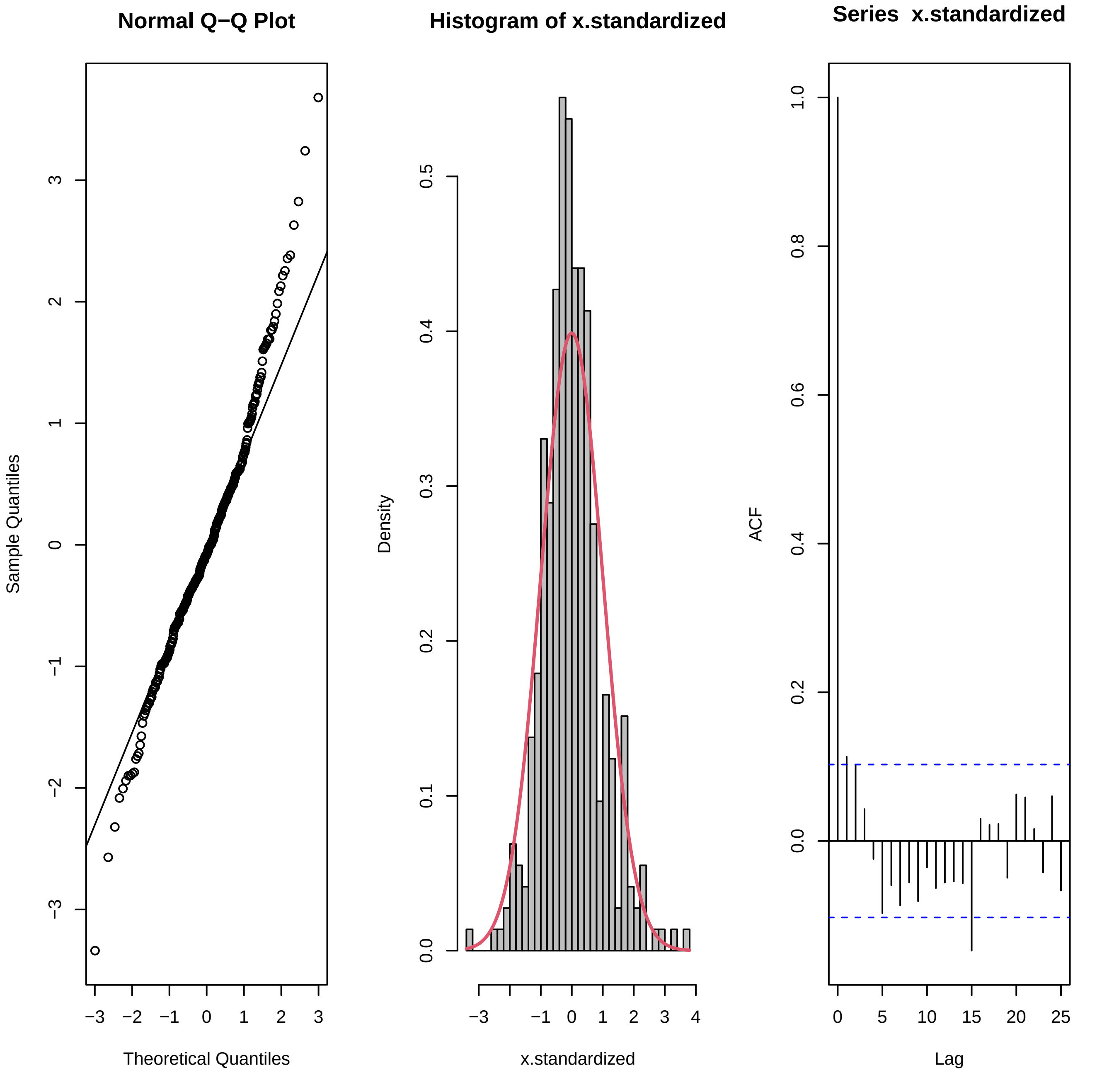

Normal Q−Q Plot
Sample Quantiles
Theoretical Quantiles
−3
−2
−1
0
1
2
3
Histogram of x.standardized
Density
x.standardized
0.0
0.1
0.2
0.3
0.4
0.5
−3
−1
0
1
2
3
4
Series x.standardized
ACF
Lag
0.0
0.2
0.4
0.6
0.8
1.0
0
5
10
15
20
25

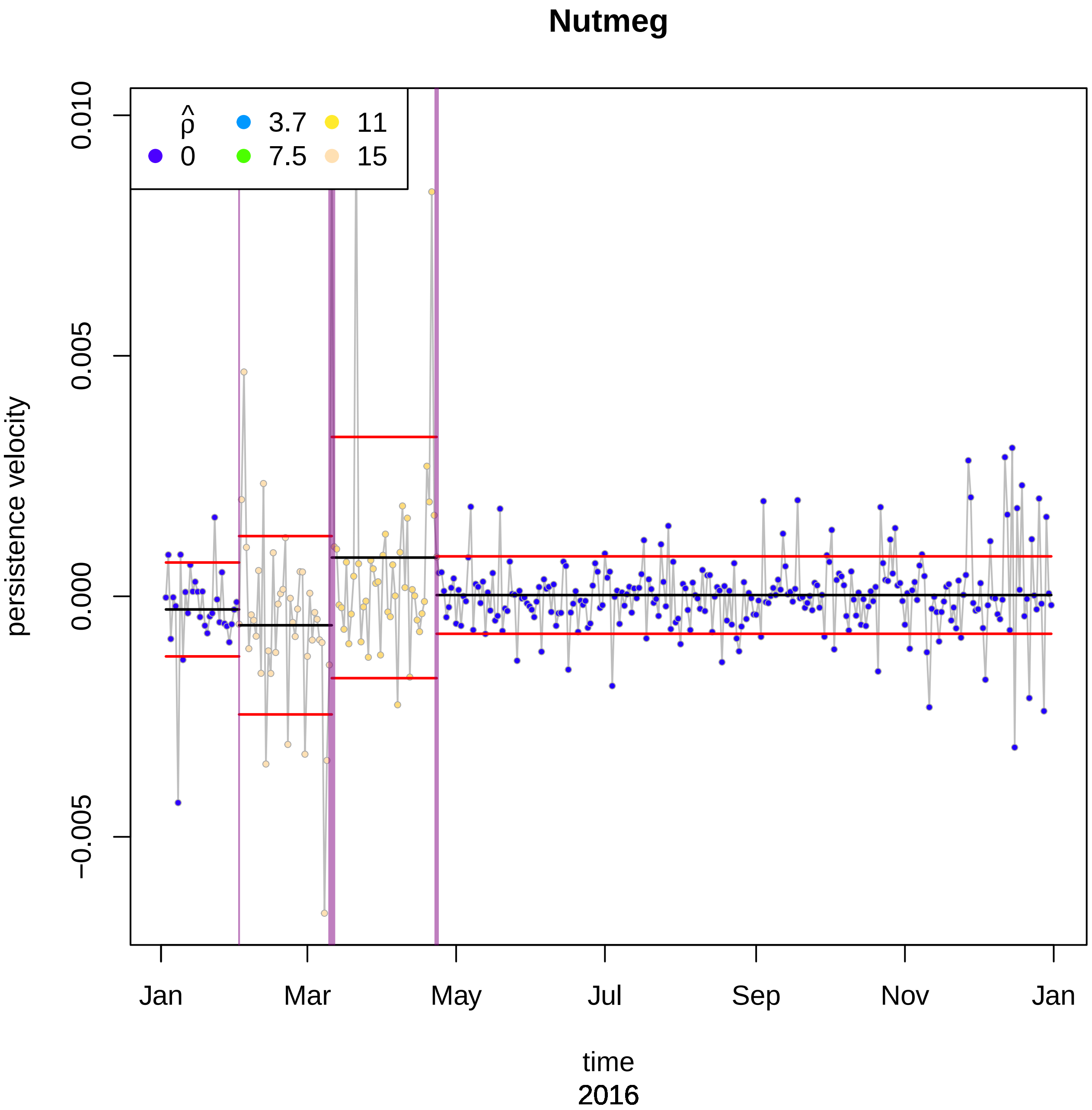
Nutmeg
ρ̂
0
3.7
7.5
11
15
0.010
0.005
0.000
−0.005
persistence velocity
Jan
Mar
May
Jul
Sep
Nov
Jan
time
2016

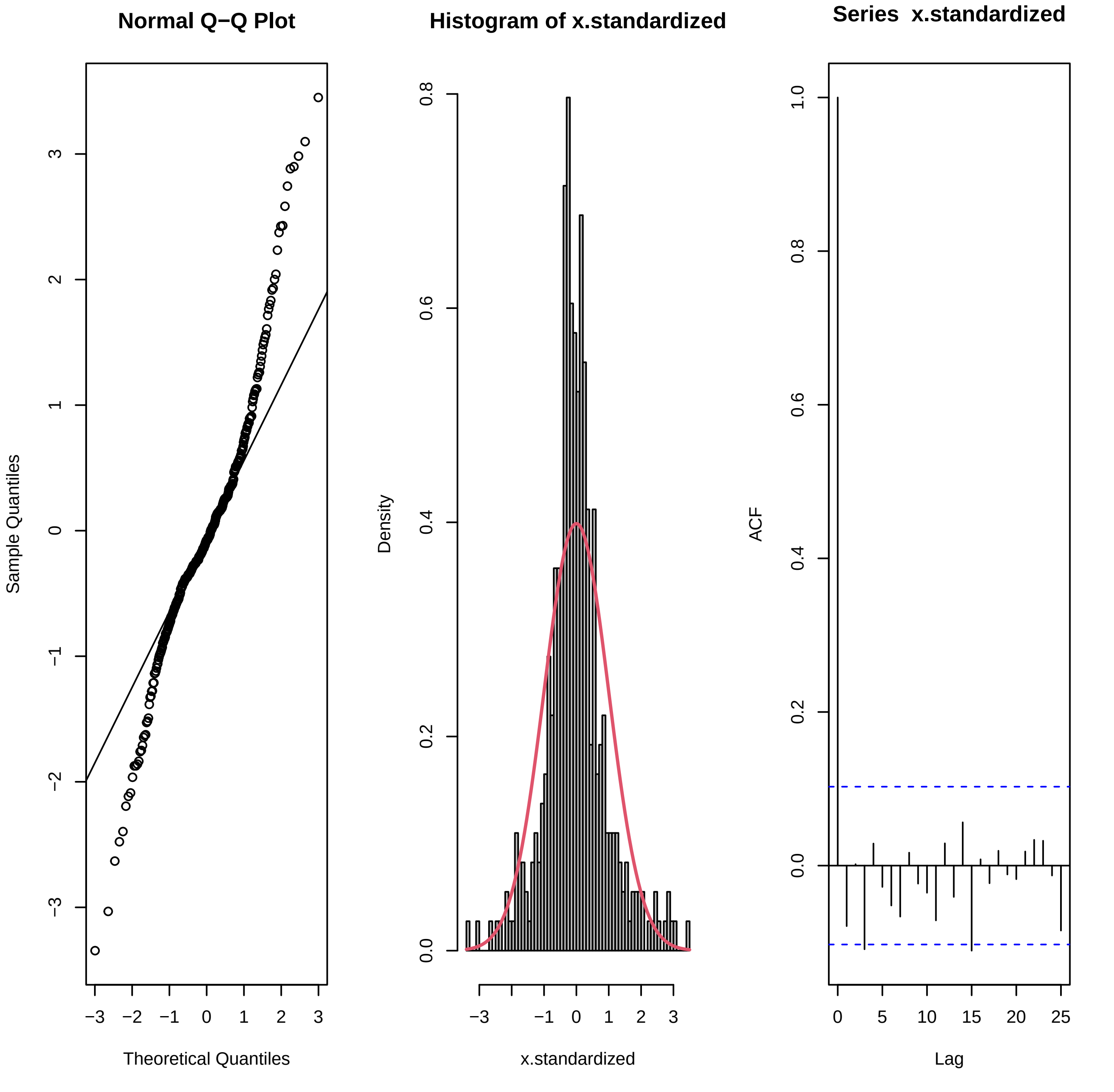

Normal Q−Q Plot
Theoretical Quantiles
Sample Quantiles
−3 −2 −1 0 1 2 3
Histogram of x.standardized
x.standardized
Density
0.0 0.2 0.4 0.6 0.8
Series x.standardized
Lag
ACF
0 5 10 15 20 25
0.0 0.2 0.4 0.6 0.8 1.0

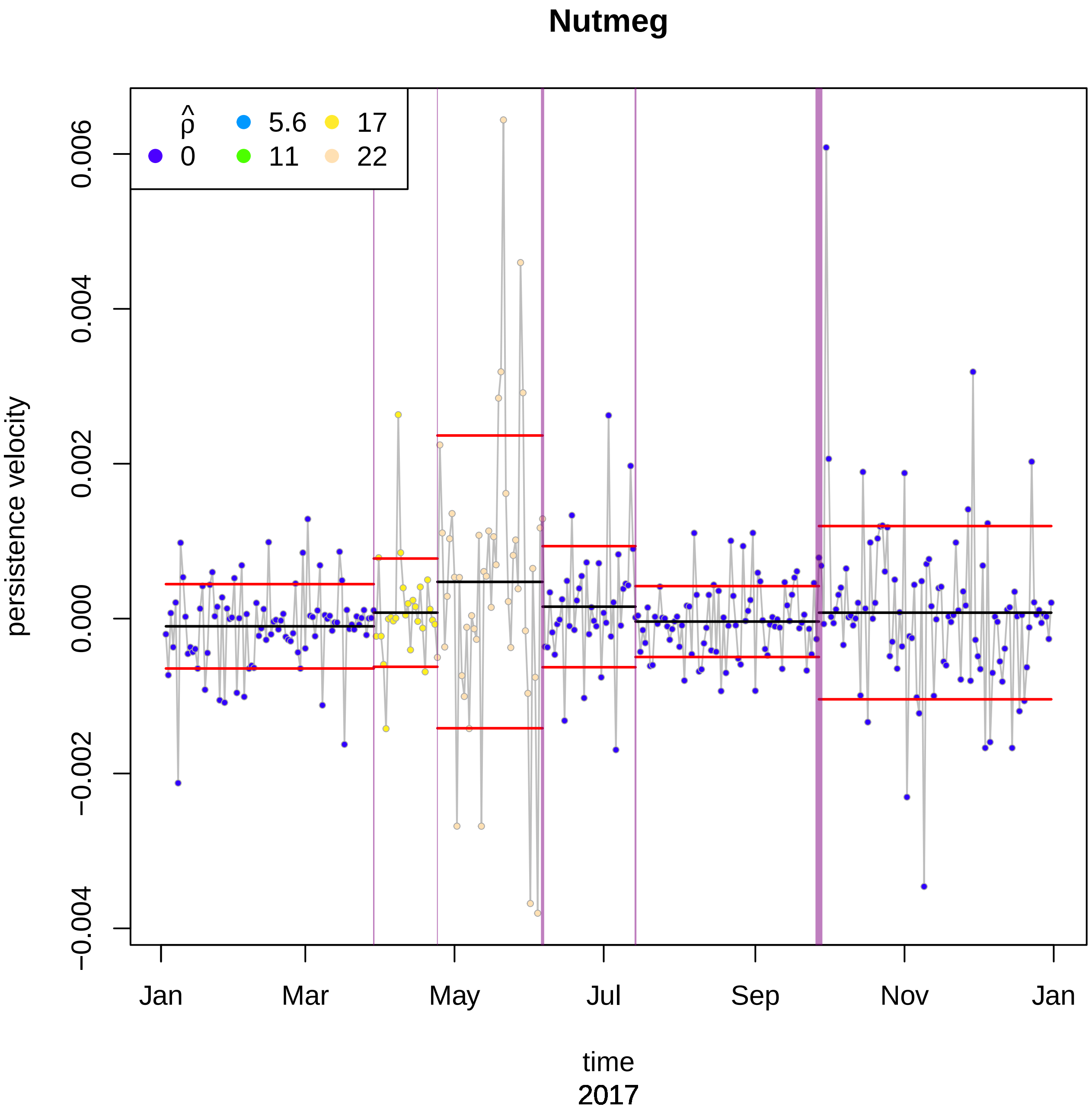

Nutmeg
ρ̂
0
5.6
11
17
22
persistence velocity
0.006
0.004
0.002
0.000
−0.002
−0.004
Jan
Mar
May
Jul
Sep
Nov
Jan
time
2017

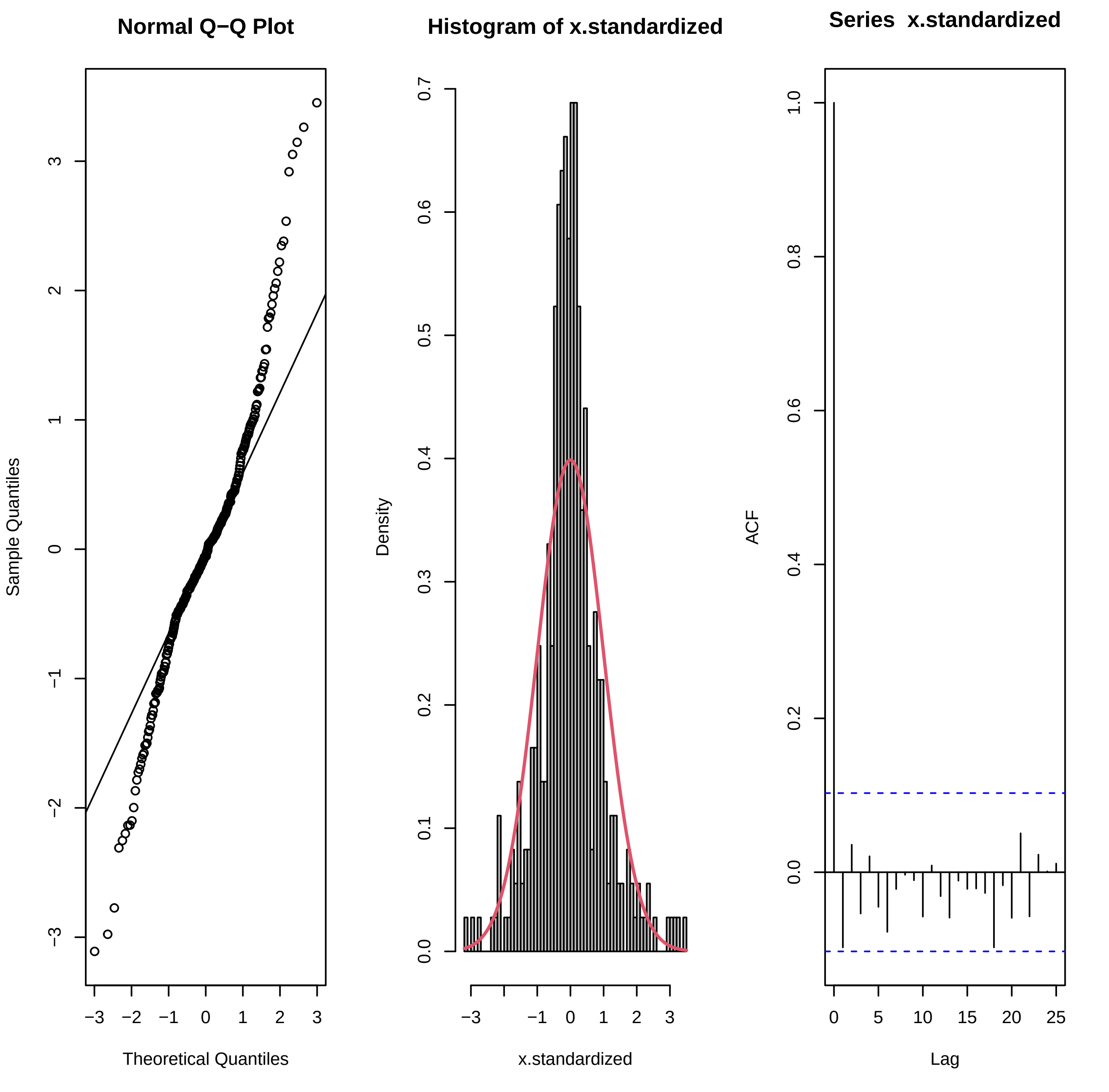

Normal Q−Q Plot
Sample Quantiles
Theoretical Quantiles
Histogram of x.standardized
Density
x.standardized
Series x.standardized
ACF
Lag

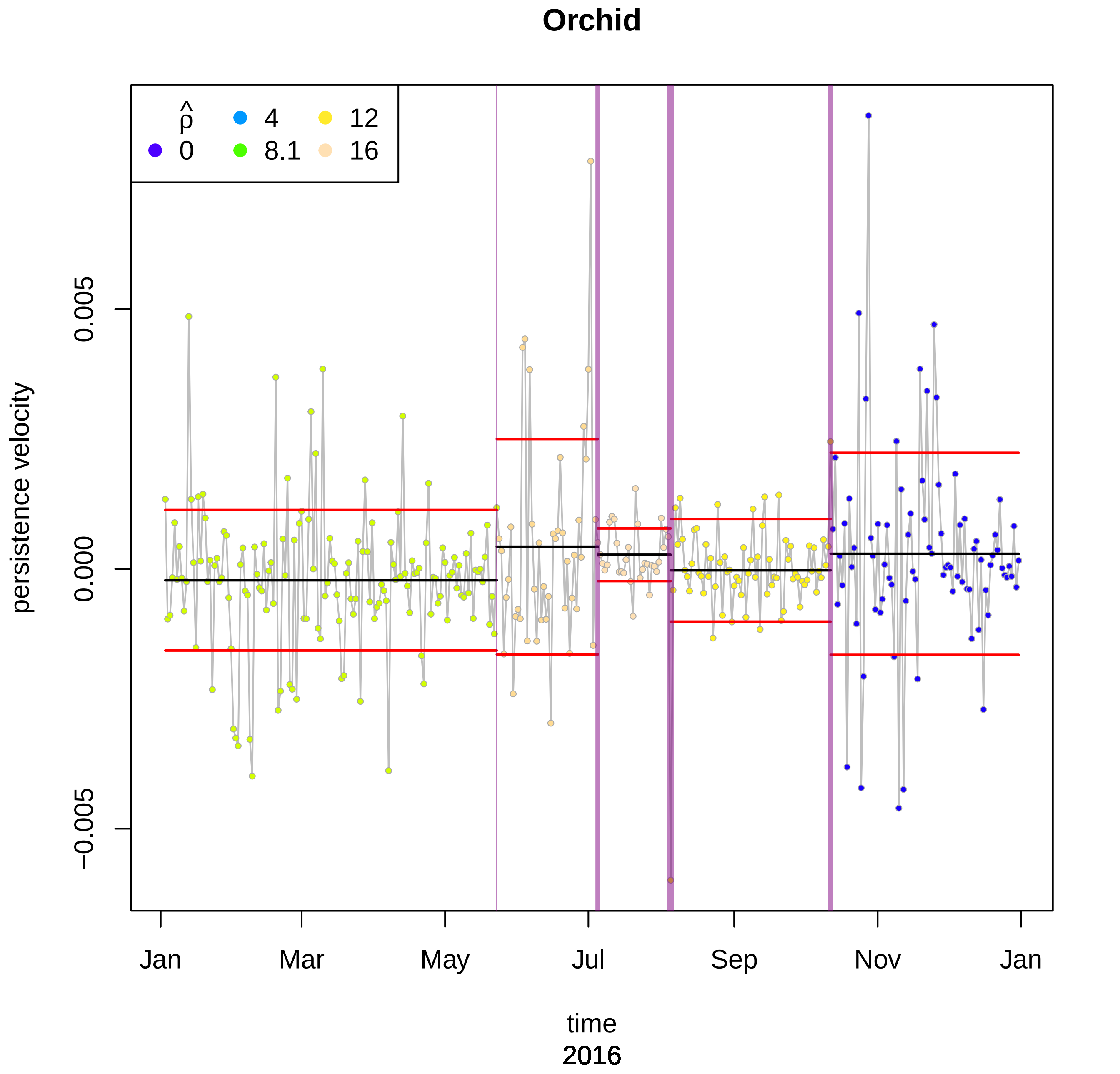

Orchid
ρ̂
0
4
8.1
12
16
persistence velocity
0.005
0.000
−0.005
Jan
Mar
May
Jul
Sep
Nov
Jan
time
2016

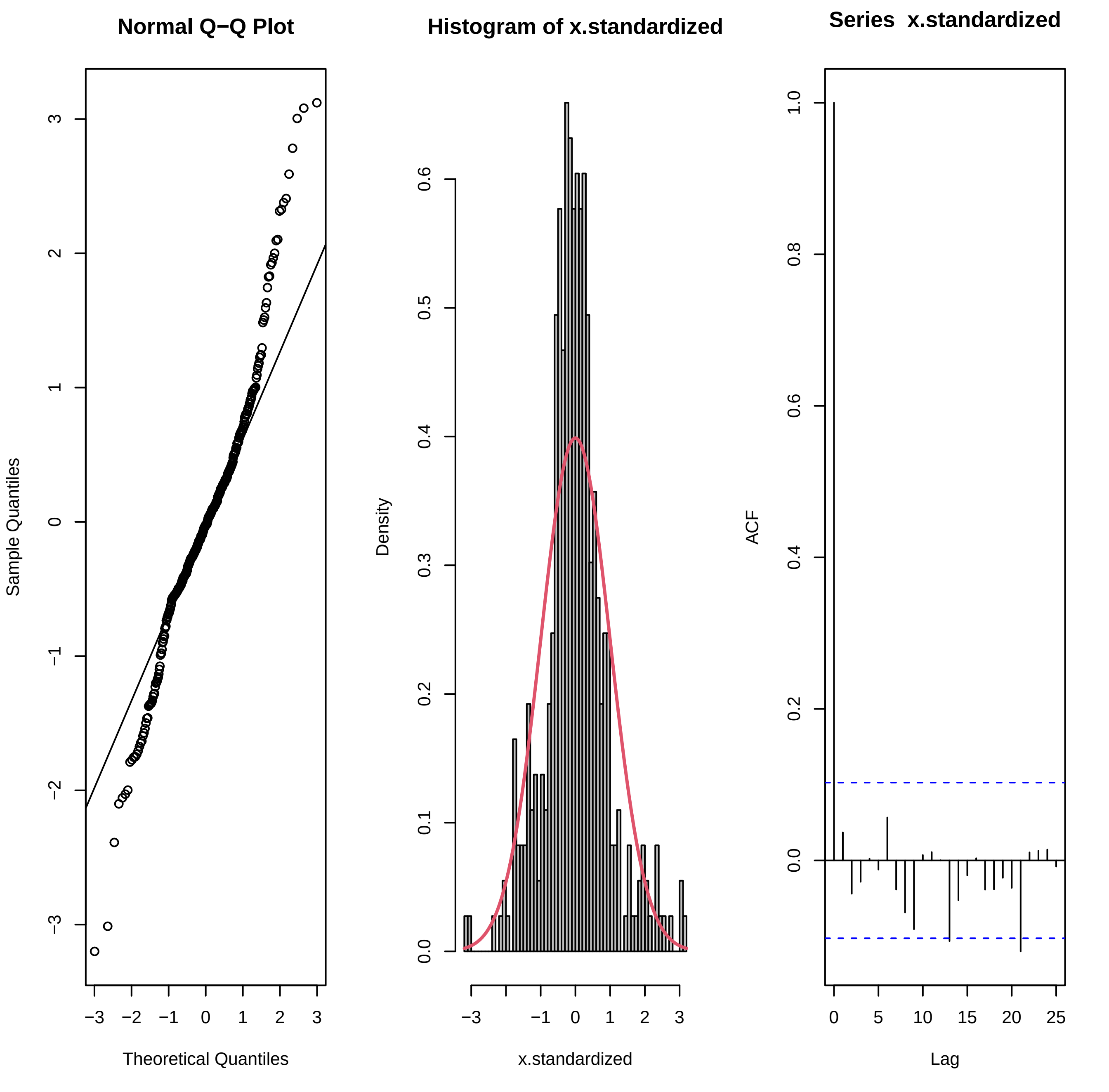

Normal Q−Q Plot
Sample Quantiles
Theoretical Quantiles
−3
−2
−1
0
1
2
3
Histogram of x.standardized
Density
x.standardized
0.0
0.1
0.2
0.3
0.4
0.5
0.6
Series x.standardized
ACF
Lag
0
5
10
15
20
25
0.0
0.2
0.4
0.6
0.8
1.0

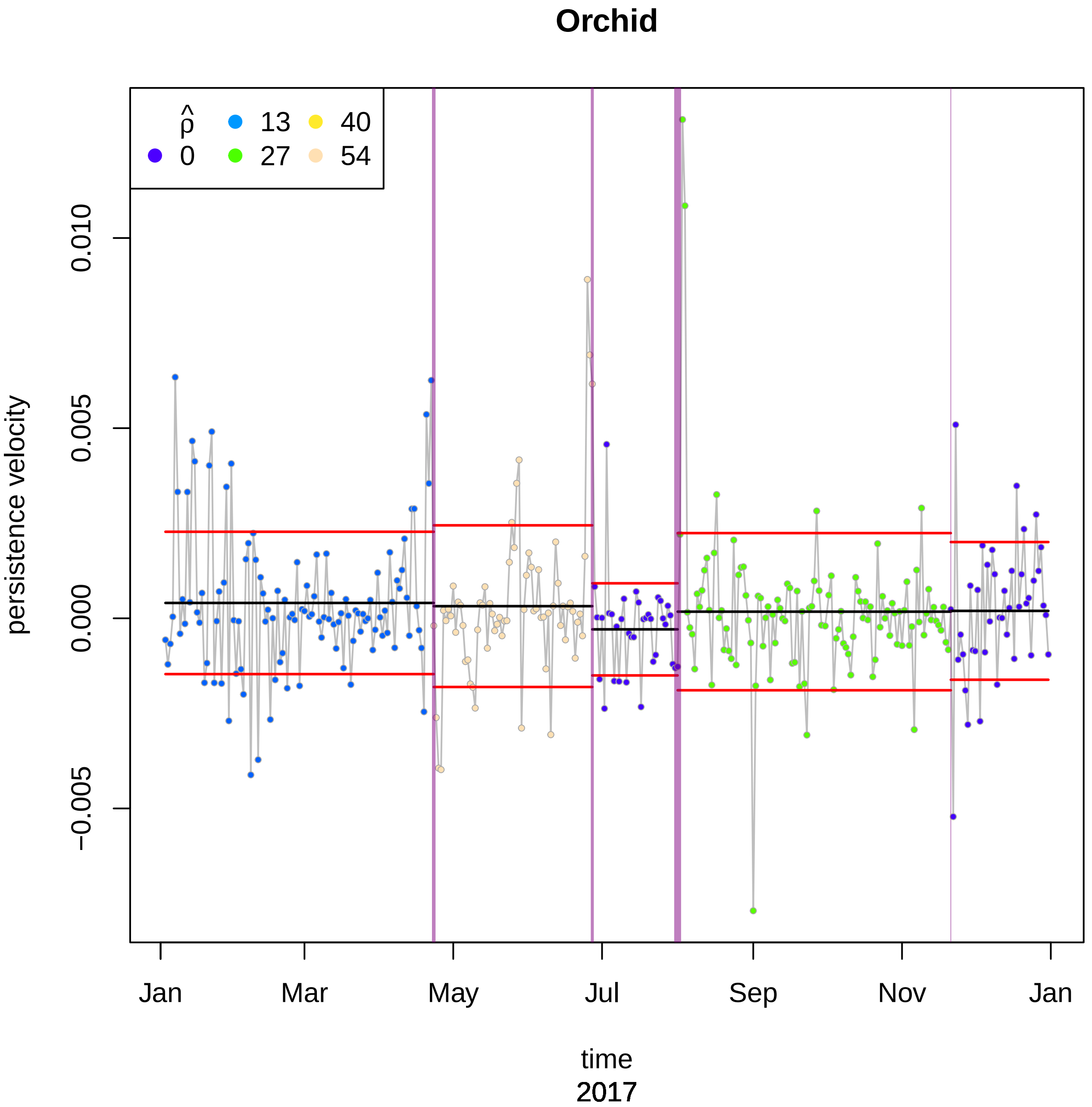

Orchid
ρ̂
0
13
27
40
54
0.010
0.005
0.000
−0.005
persistence velocity
Jan
Mar
May
Jul
Sep
Nov
Jan
time
2017

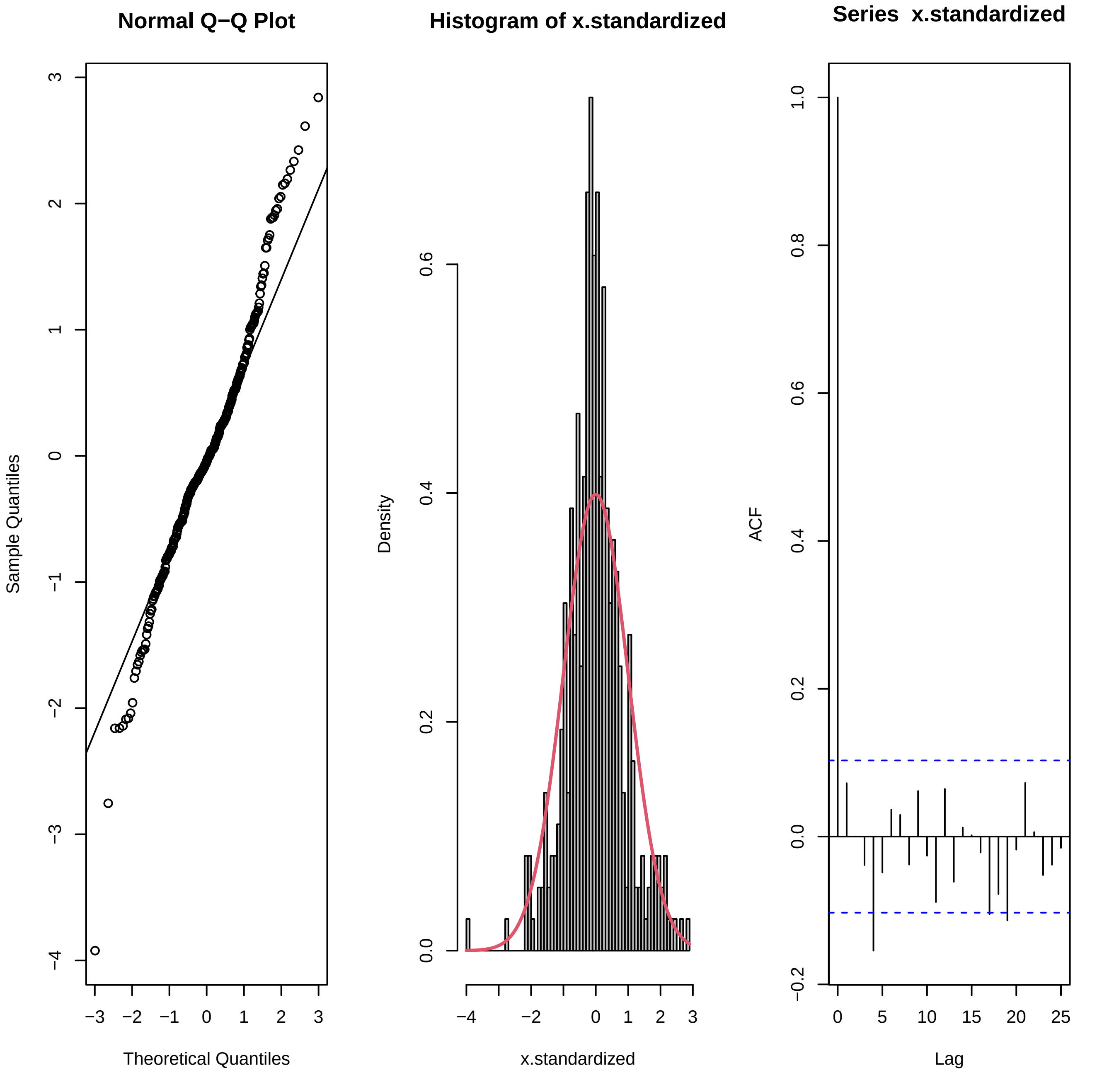

Normal Q−Q Plot
Sample Quantiles
Theoretical Quantiles
−4
−3
−2
−1
0
1
2
3
Histogram of x.standardized
Density
x.standardized
0.0
0.2
0.4
0.6
Series x.standardized
ACF
Lag
−0.2
0.0
0.2
0.4
0.6
0.8
1.0
0
5
10
15
20
25

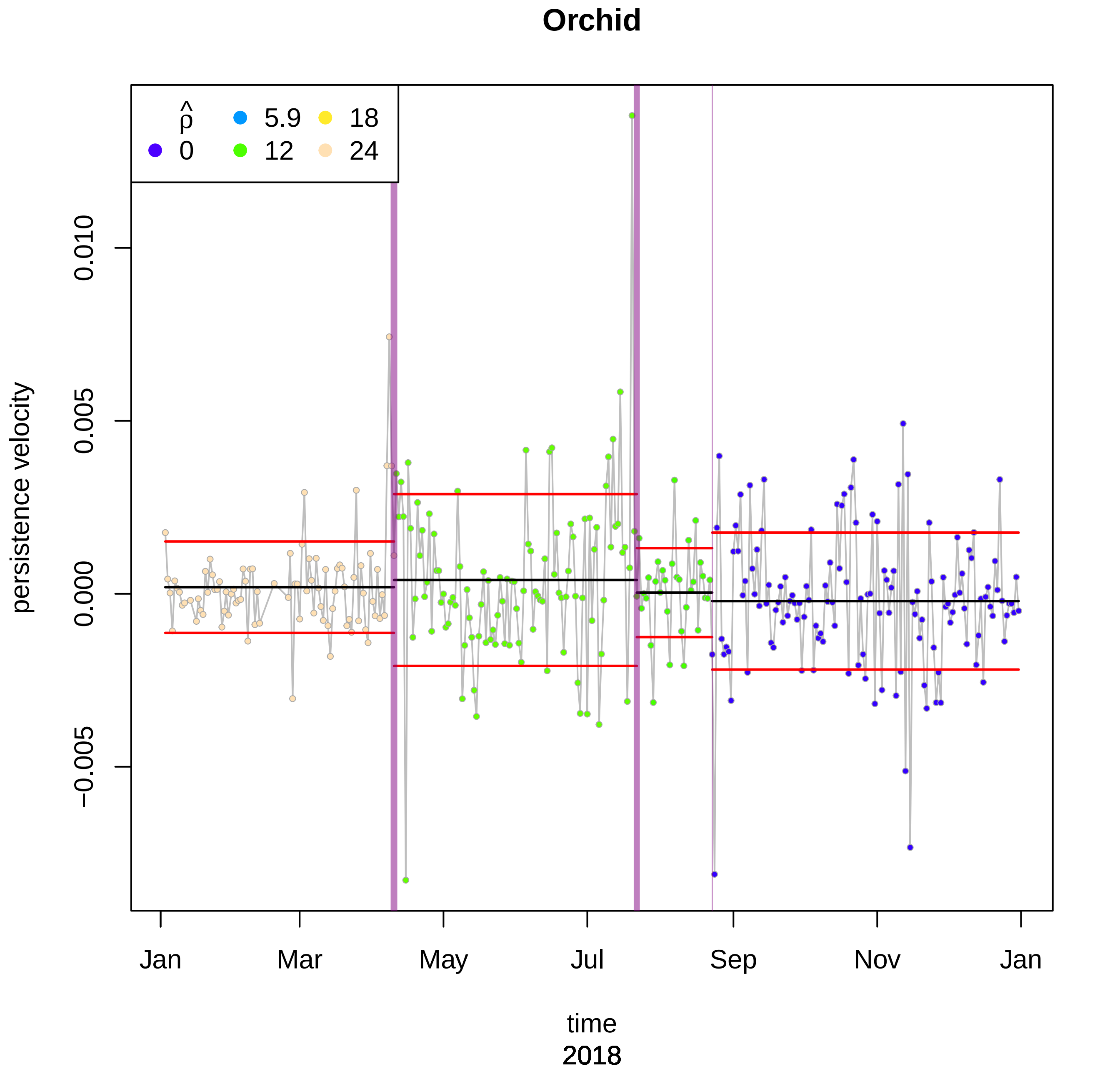
Orchid
ρ̂
0
5.9
12
18
24
persistence velocity
0.010
0.005
0.000
−0.005
Jan
Mar
May
Jul
Sep
Nov
Jan
time
2018

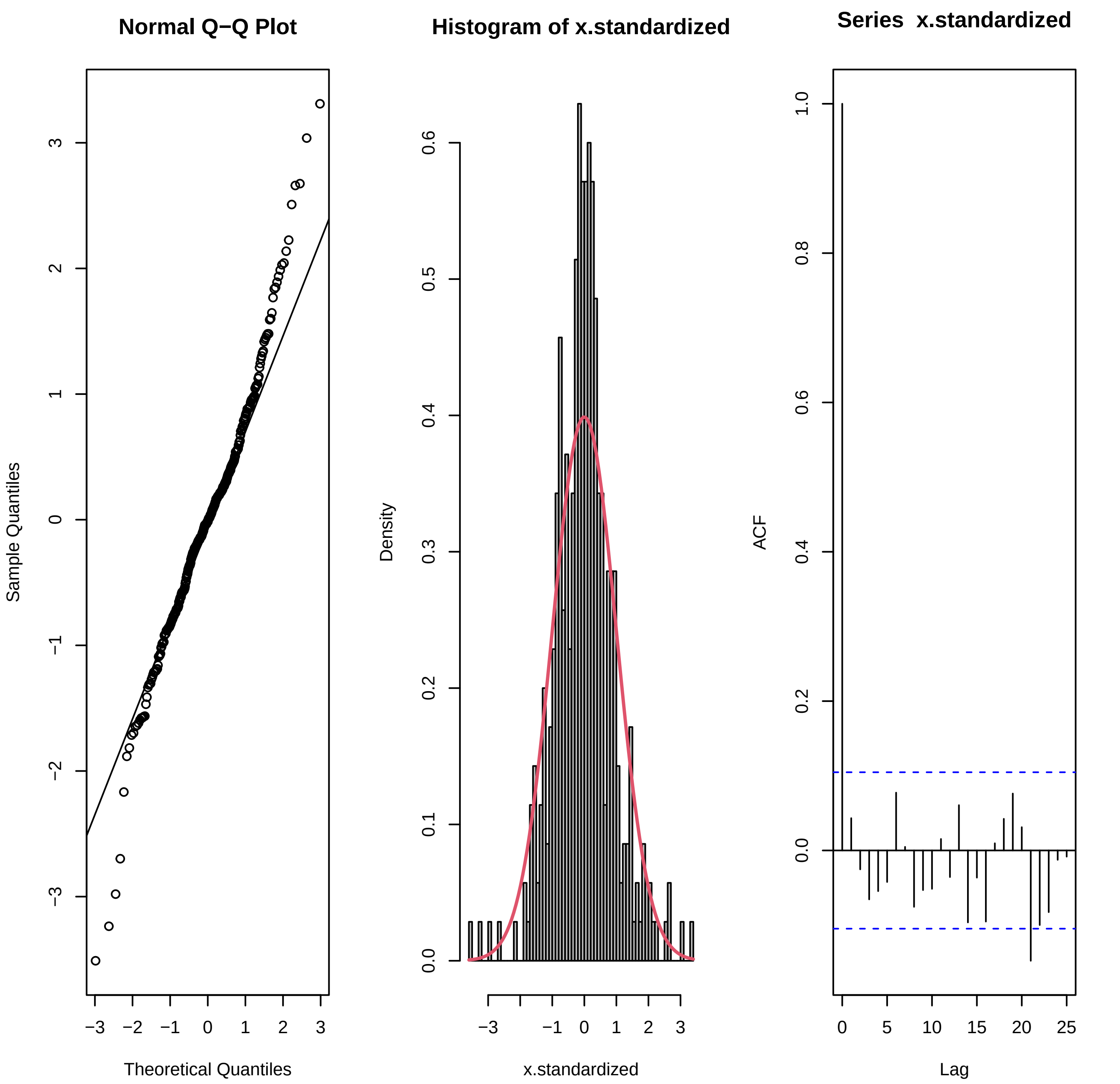

Normal Q−Q Plot
Theoretical Quantiles
Sample Quantiles
Histogram of x.standardized
x.standardized
Density
Series x.standardized
Lag
ACF

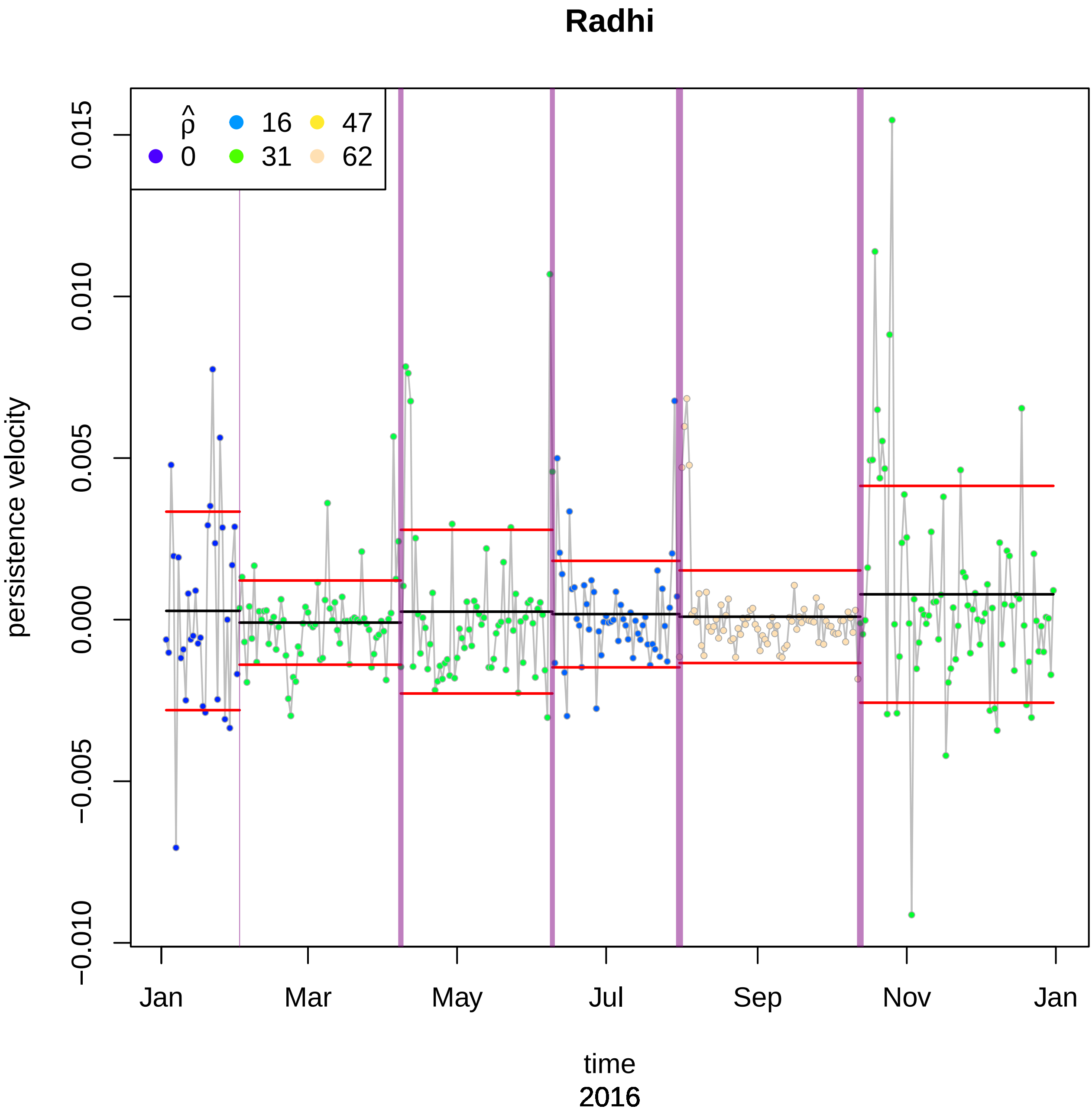

Radhi
ρ̂
0
16
31
47
62
persistence velocity
0.015
0.010
0.005
0.000
−0.005
−0.010
Jan
Mar
May
Jul
Sep
Nov
Jan
time
2016

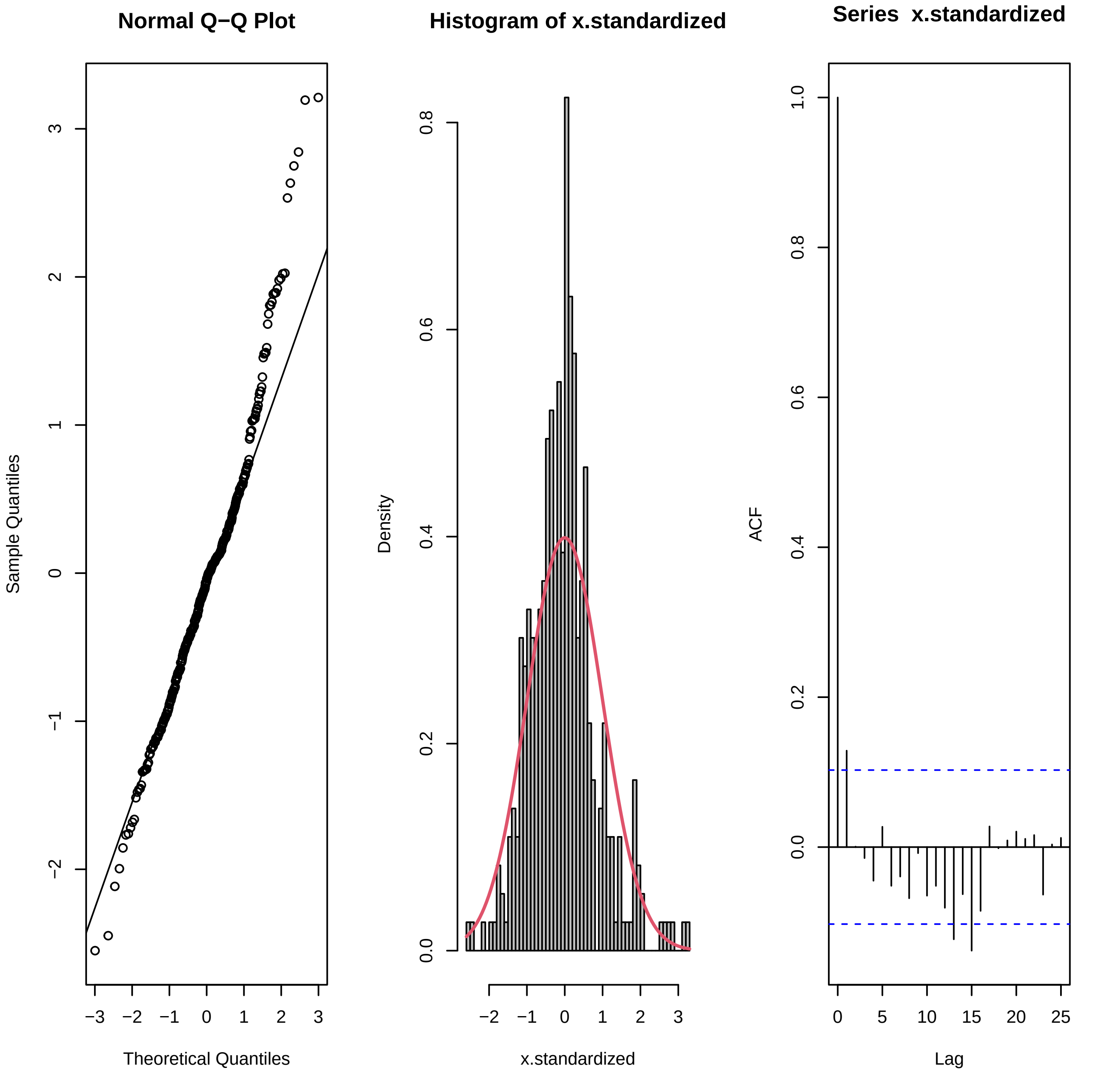
Normal Q−Q Plot
Sample Quantiles
Theoretical Quantiles
−3
−2
−1
0
1
2
3
Histogram of x.standardized
Density
x.standardized
0.0
0.2
0.4
0.6
0.8
Series x.standardized
ACF
Lag
0
5
10
15
20
25
1.0

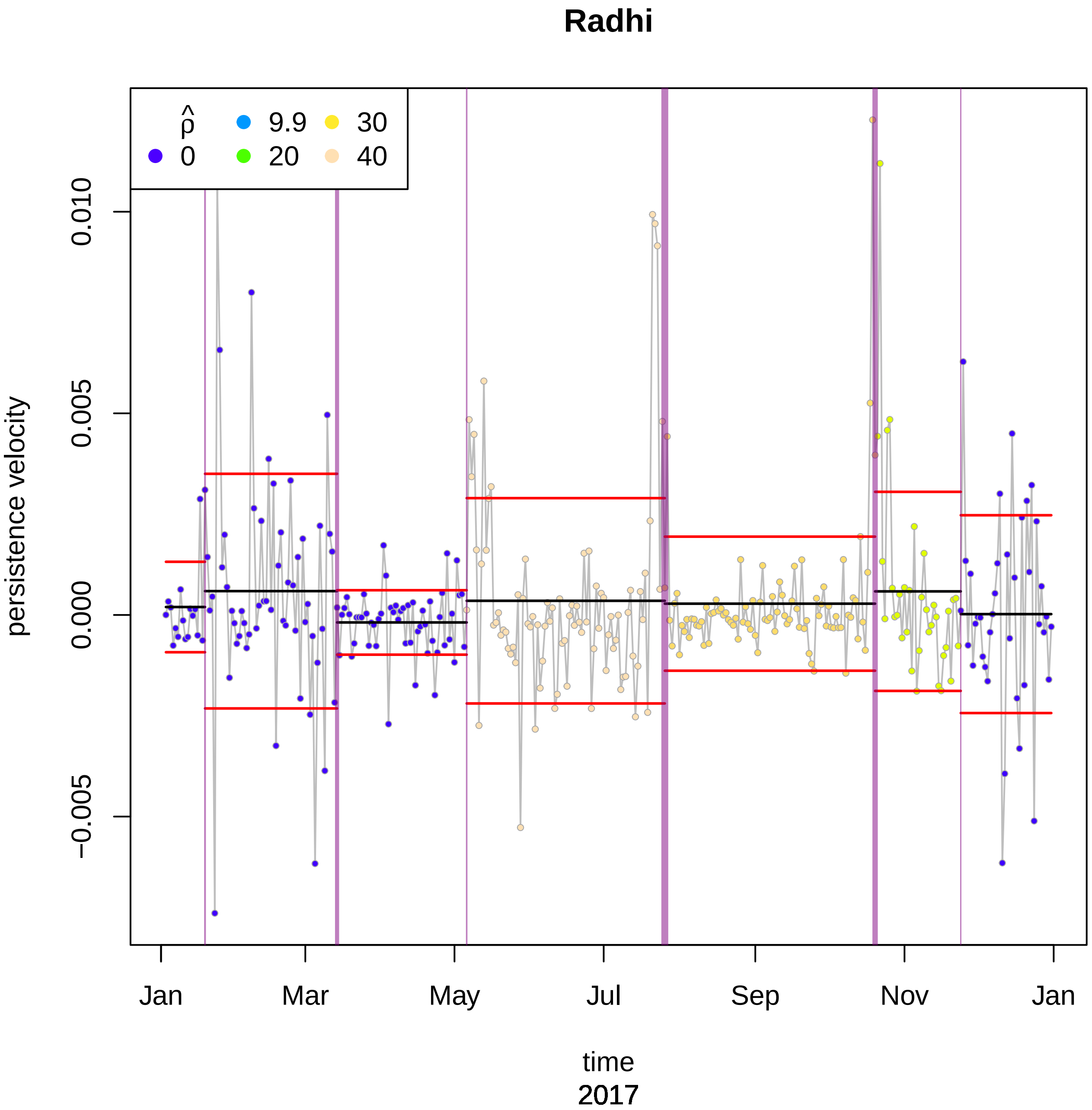

Radhi
ρ̂
0
9.9
20
30
40
persistence velocity
0.010
0.005
0.000
−0.005
Jan
Mar
May
Jul
Sep
Nov
Jan
time
2017

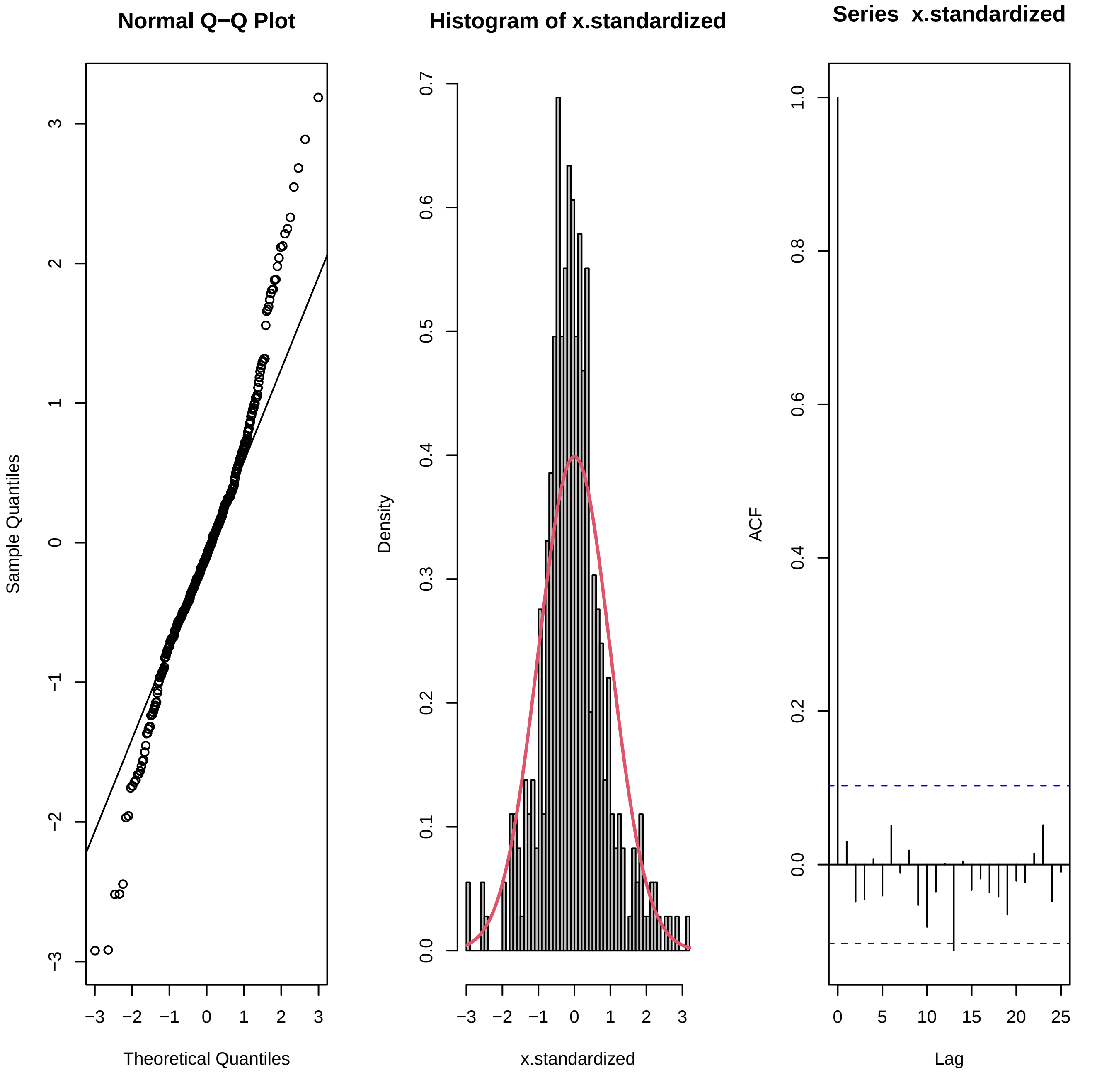
Normal Q−Q Plot
Histogram of x.standardized
Series x.standardized
Sample Quantiles
Theoretical Quantiles
Density
x.standardized
ACF
Lag

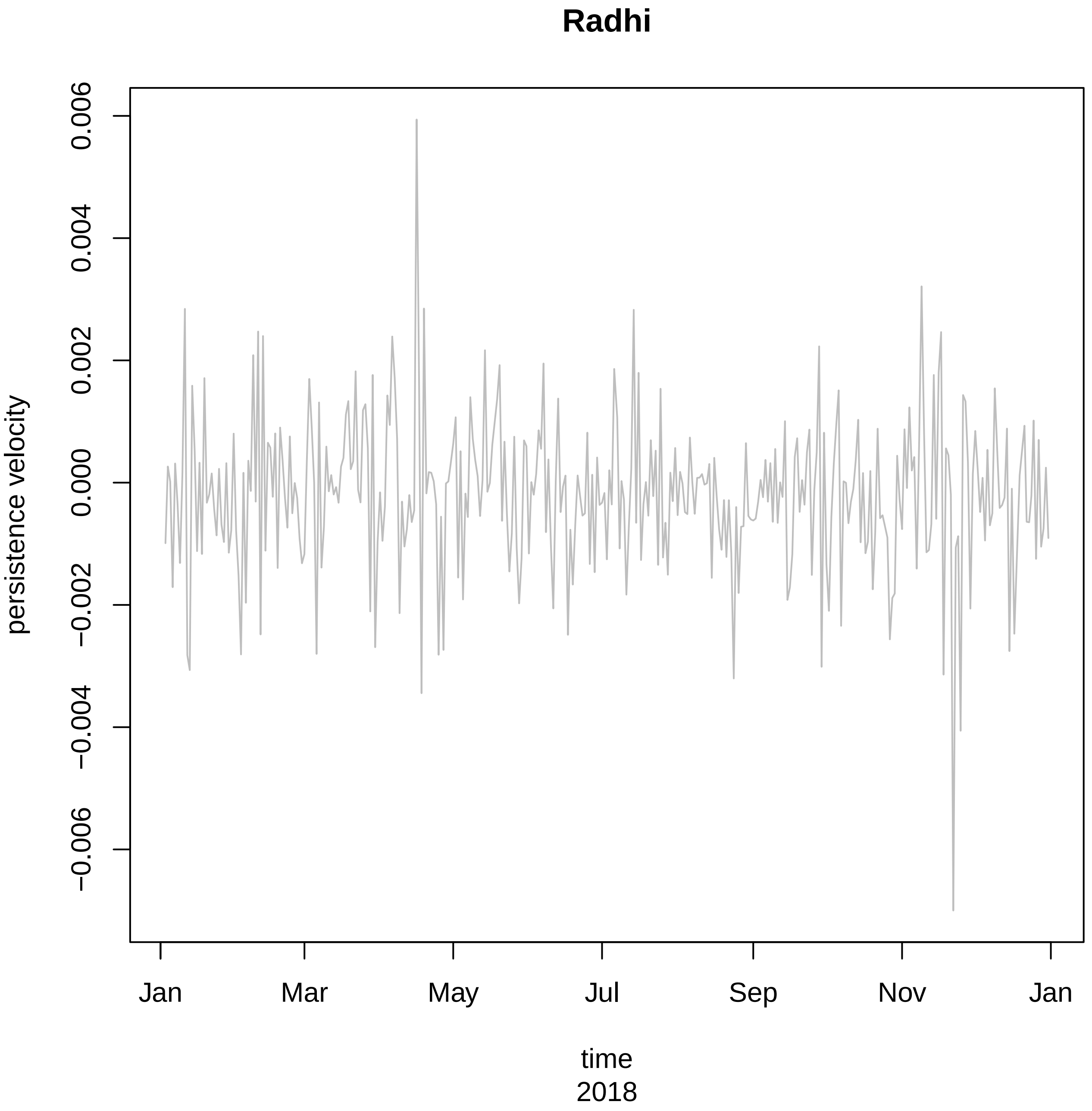
Radhi
0.006
0.004
0.002
0.000
−0.002
−0.004
−0.006
persistence velocity
Jan
Mar
May
Jul
Sep
Nov
Jan
time
2018

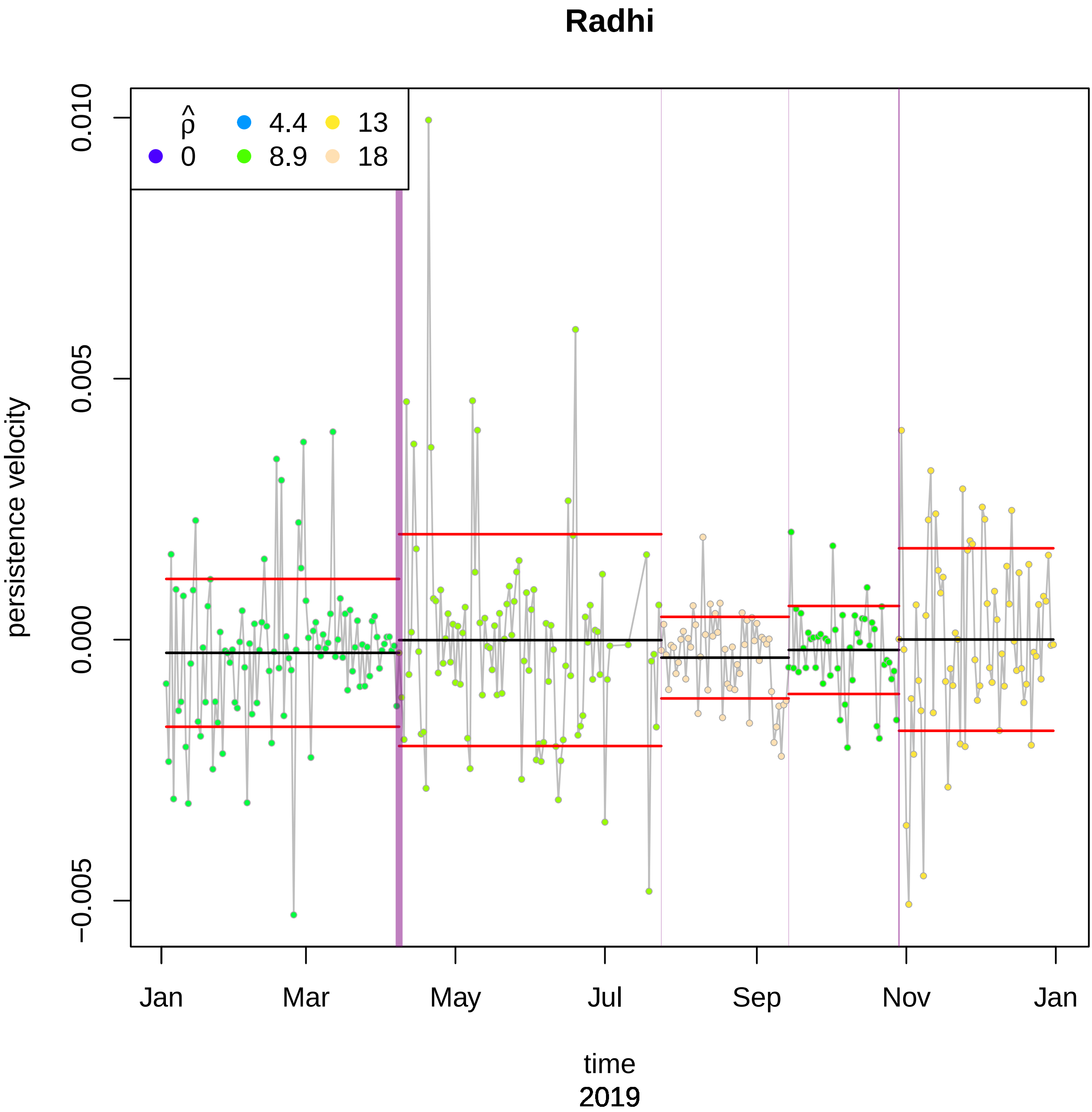
Radhi
ρ̂
0
4.4
8.9
13
18
0.010
0.005
0.000
−0.005
persistence velocity
Jan
Mar
May
Jul
Sep
Nov
Jan
time
2019

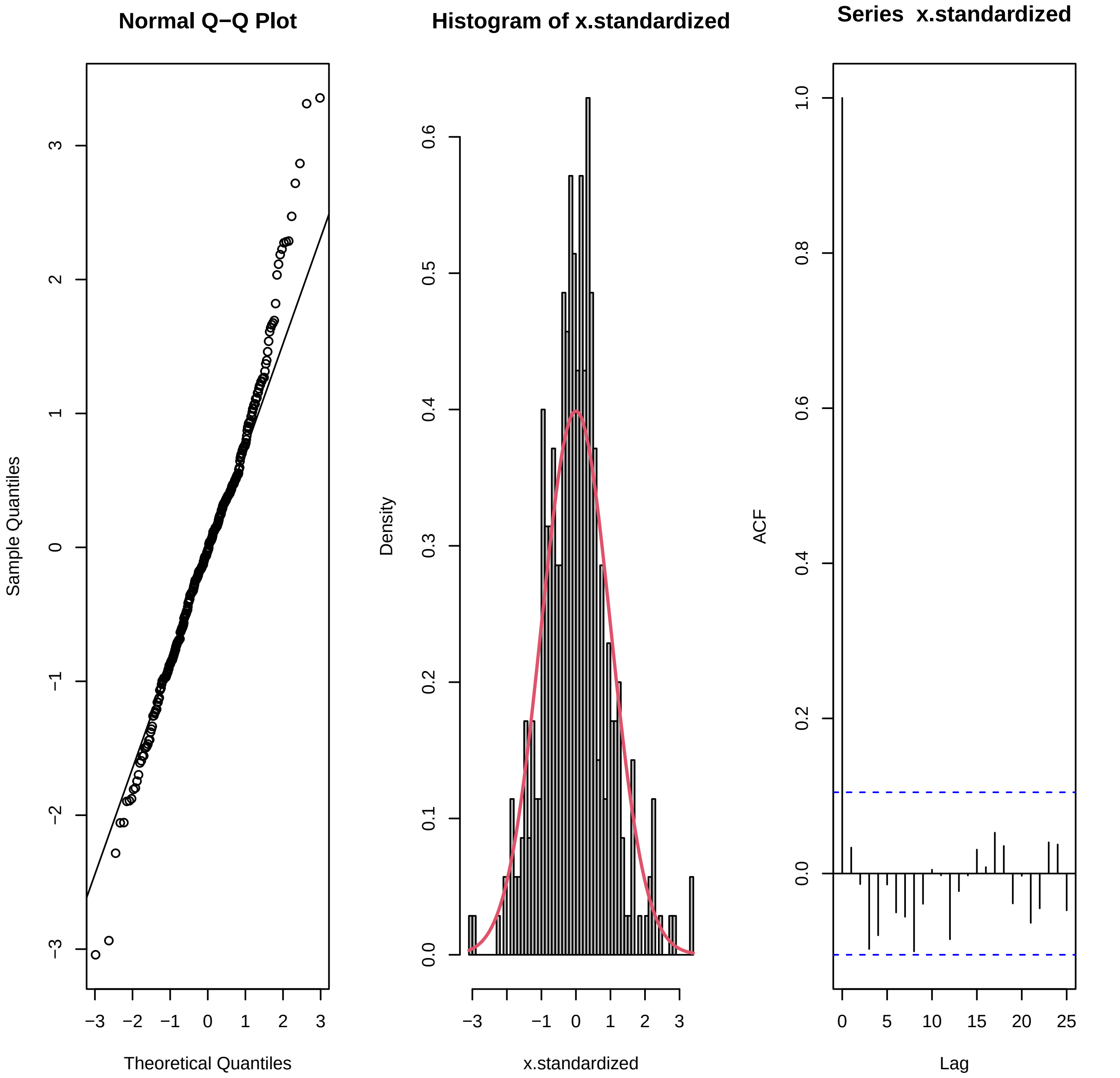
Normal Q−Q Plot
Theoretical Quantiles
Sample Quantiles
−3 −2 −1 0 1 2 3
Histogram of x.standardized
x.standardized
Density
0.0 0.1 0.2 0.3 0.4 0.5 0.6
Series x.standardized
Lag
ACF
0 5 10 15 20 25
0.0 0.2 0.4 0.6 0.8 1.0

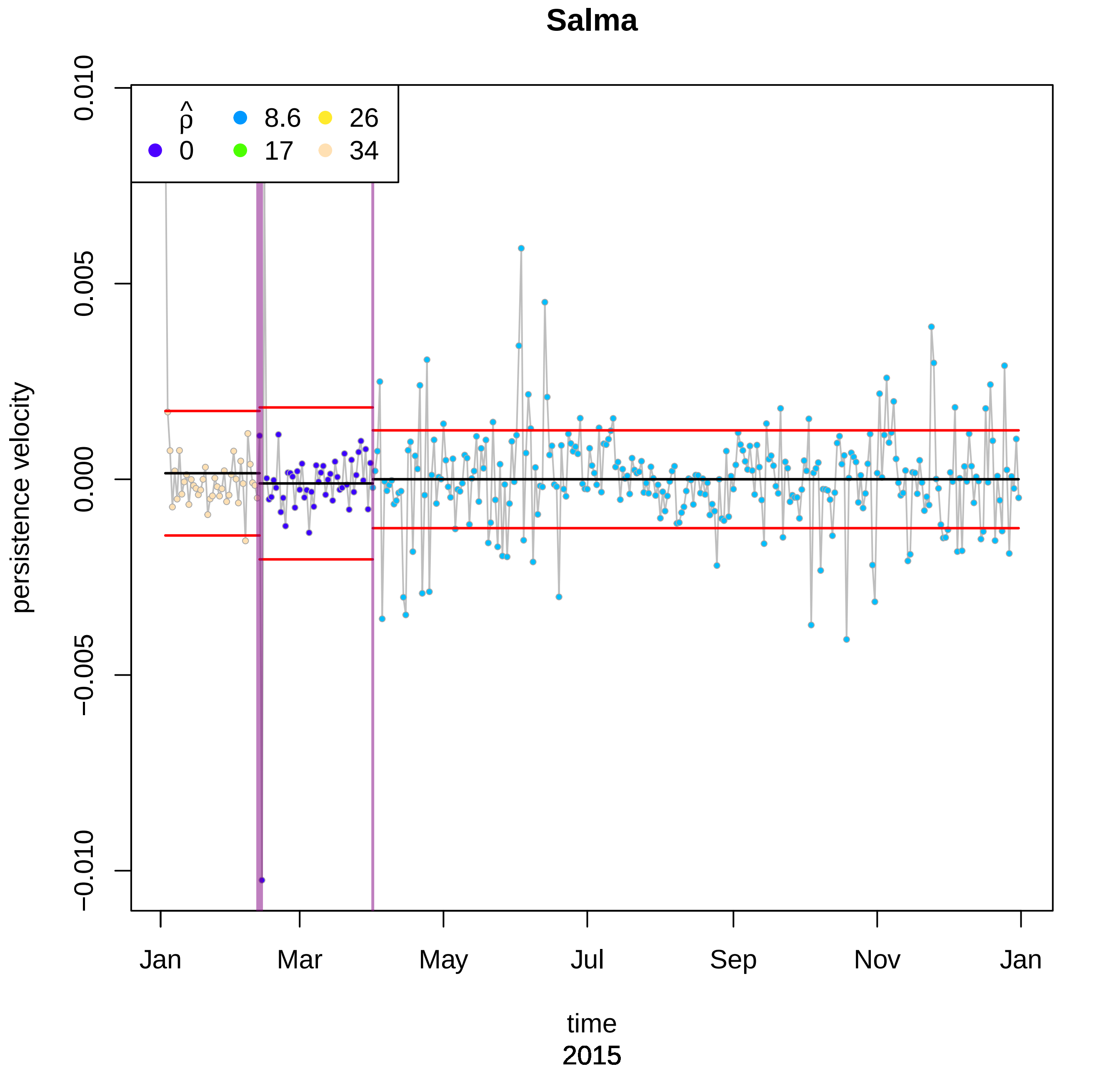
Salma
ρ̂
0
8.6
17
26
34
persistence velocity
0.010
0.005
0.000
−0.005
−0.010
Jan
Mar
May
Jul
Sep
Nov
Jan
time
2015

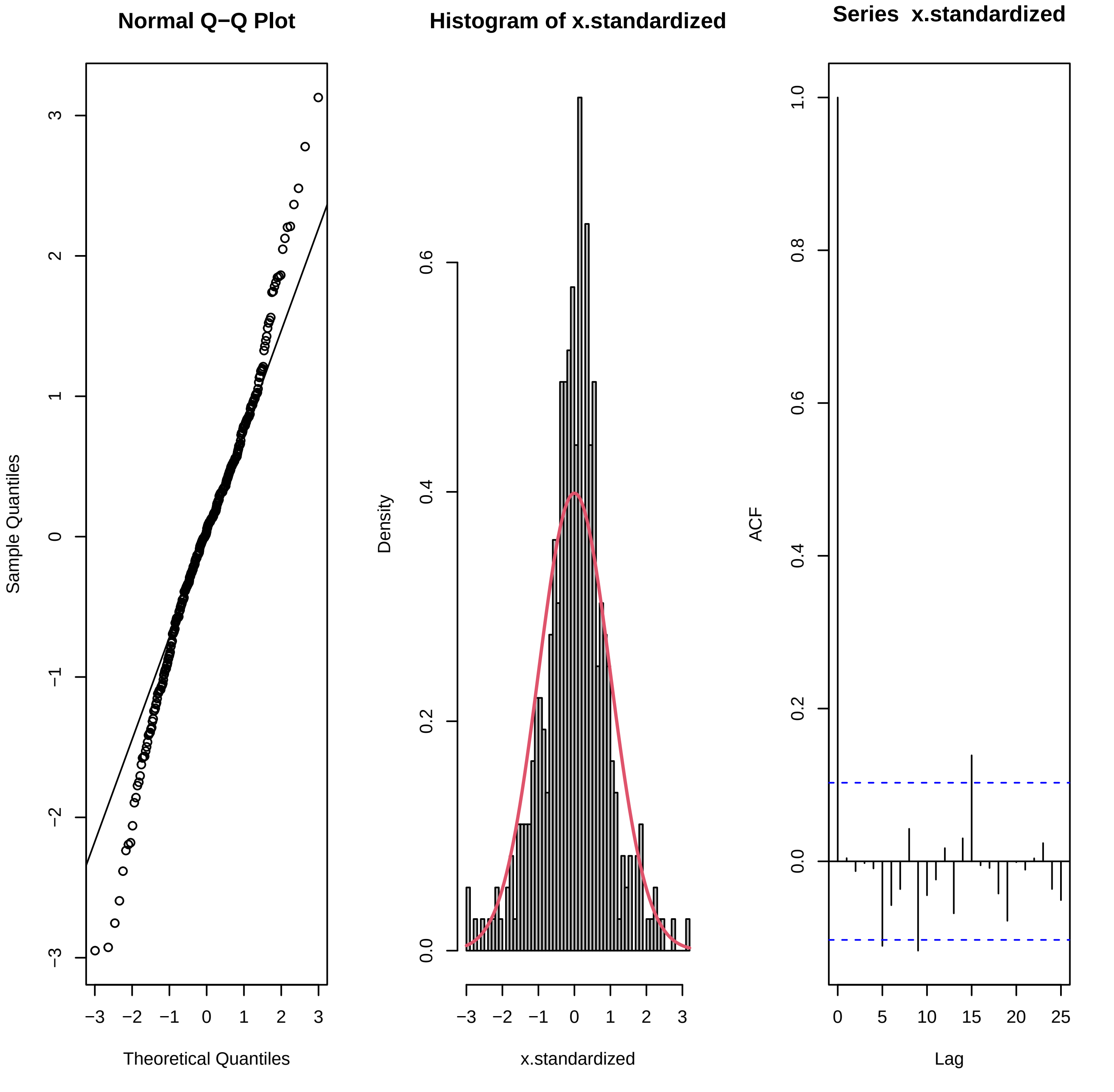

Normal Q−Q Plot
Sample Quantiles
Theoretical Quantiles
−3
−2
−1
0
1
2
3
Histogram of x.standardized
Density
x.standardized
0.0
0.2
0.4
0.6
Series x.standardized
ACF
Lag
0.0
0.2
0.4
0.6
0.8
1.0
0
5
10
15
20
25

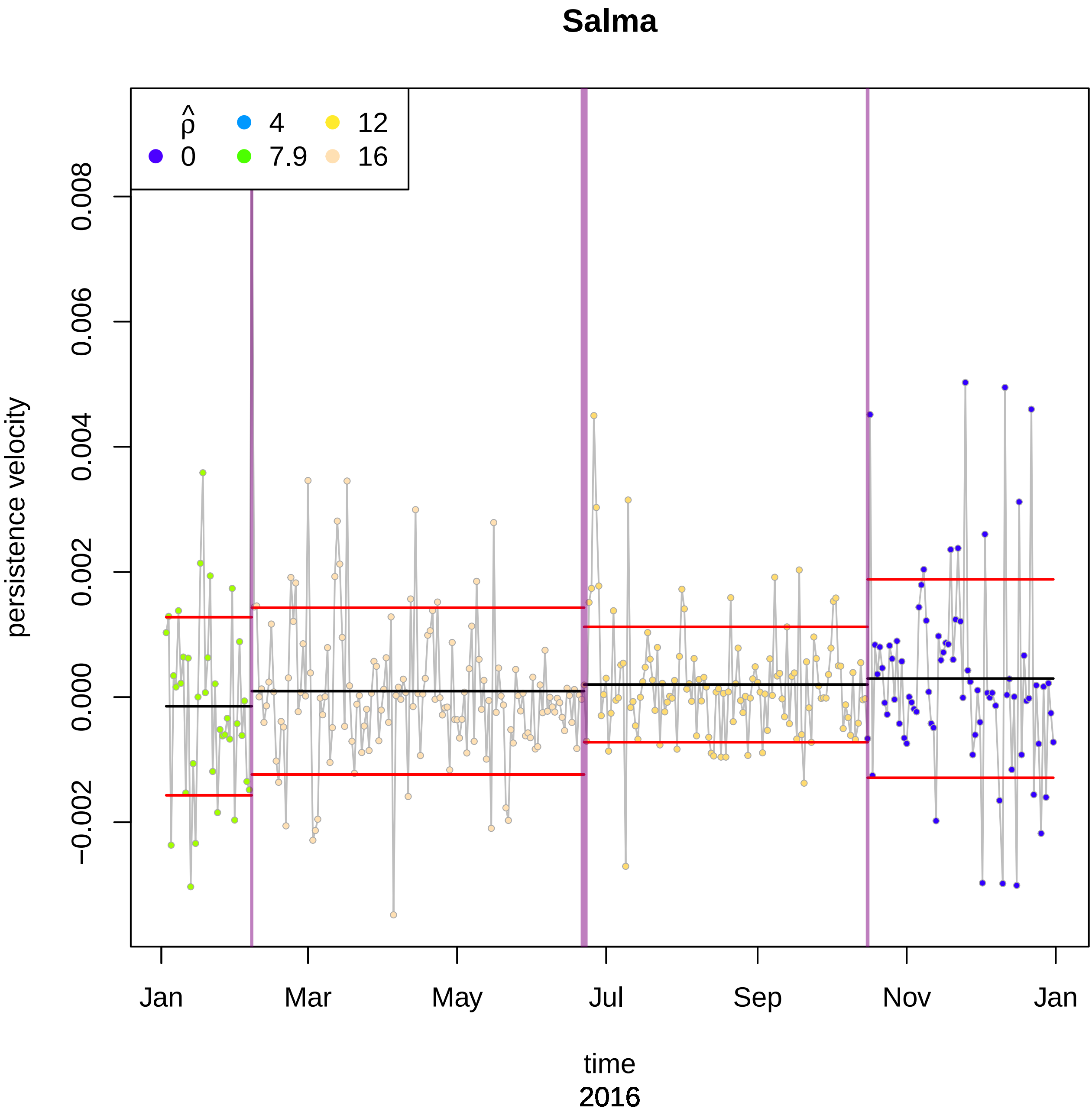

Salma
ρ̂
0
4
7.9
12
16
persistence velocity
0.008
0.006
0.004
0.002
0.000
−0.002
Jan
Mar
May
Jul
Sep
Nov
Jan
time
2016

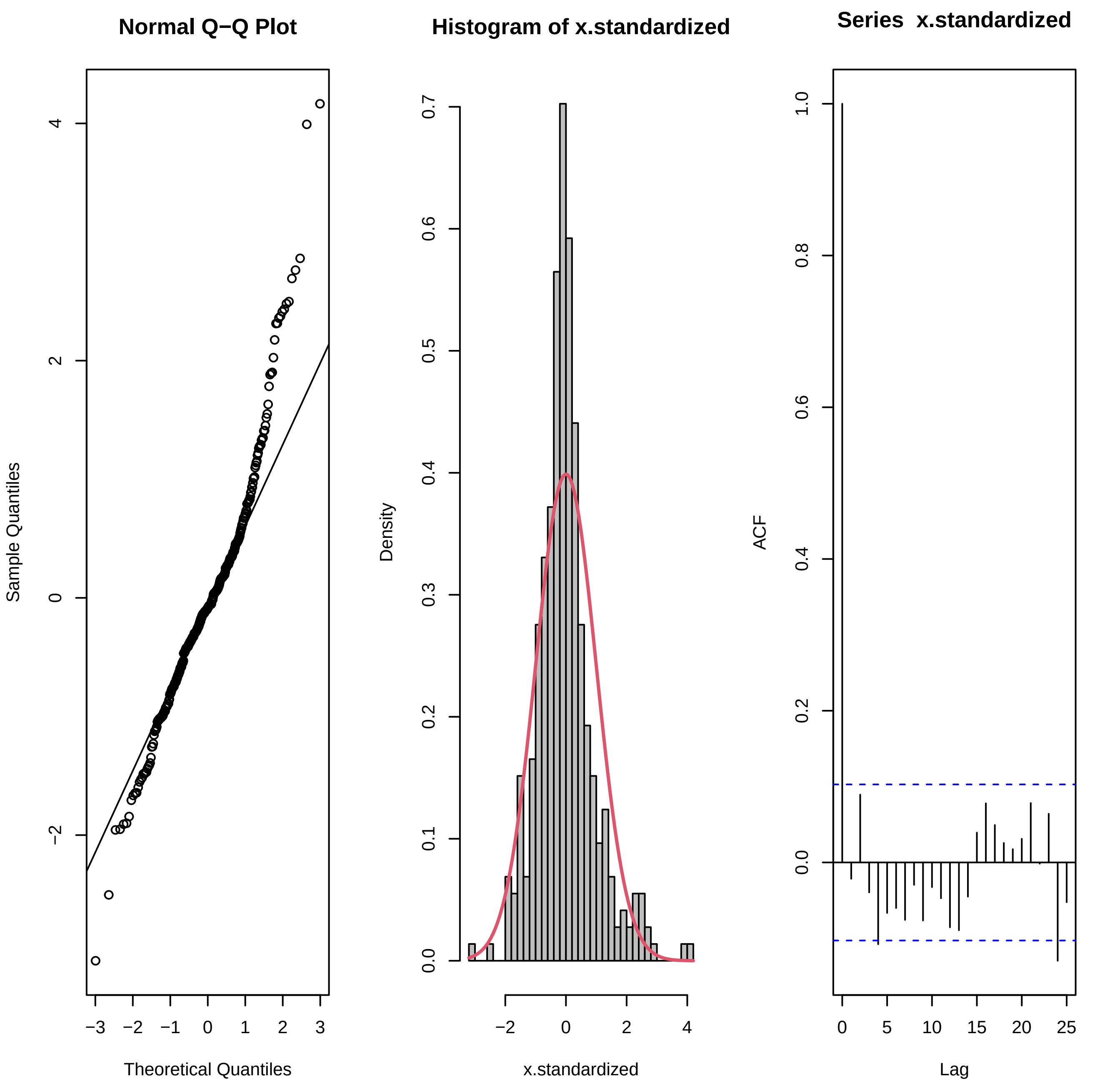

Normal Q−Q Plot
Sample Quantiles
Theoretical Quantiles
−3 −2 −1 0 1 2 3
−2 0 2 4
Histogram of x.standardized
Density
x.standardized
0.0 0.1 0.2 0.3 0.4 0.5 0.6 0.7
−2 0 2 4
Series x.standardized
ACF
Lag
0.0 0.2 0.4 0.6 0.8 1.0
0 5 10 15 20 25

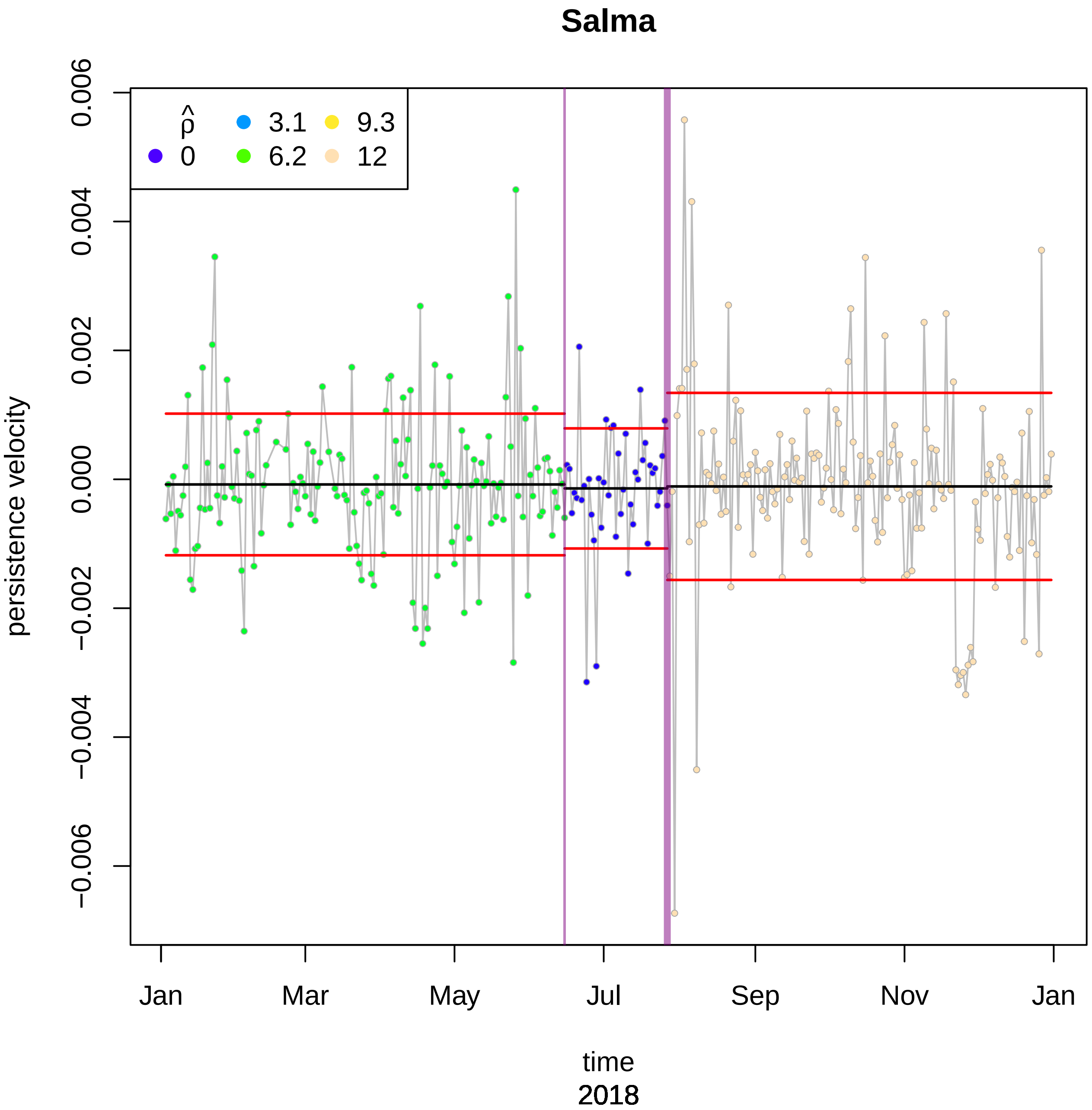

Salma
ρ̂
0
3.1
6.2
9.3
12
persistence velocity
0.006
0.004
0.002
0.000
−0.002
−0.004
−0.006
Jan
Mar
May
Jul
Sep
Nov
Jan
time
2018

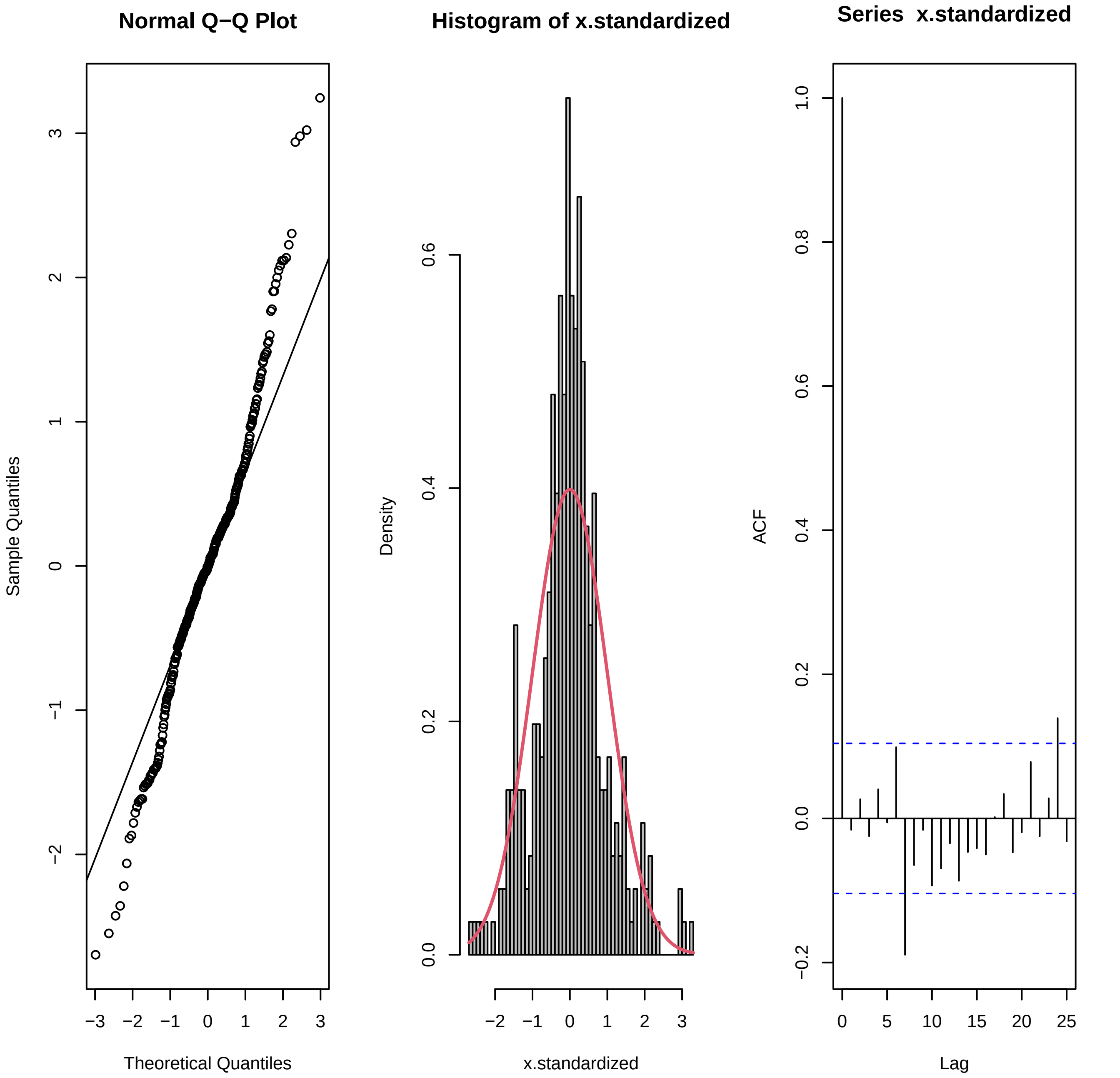

Normal Q−Q Plot
Sample Quantiles
Theoretical Quantiles
−3 −2 −1 0 1 2 3
Histogram of x.standardized
Density
0.0 0.2 0.4 0.6
−2 −1 0 1 2 3
x.standardized
Series x.standardized
ACF
−0.2 0.0 0.2 0.4 0.6 0.8 1.0
0 5 10 15 20 25
Lag

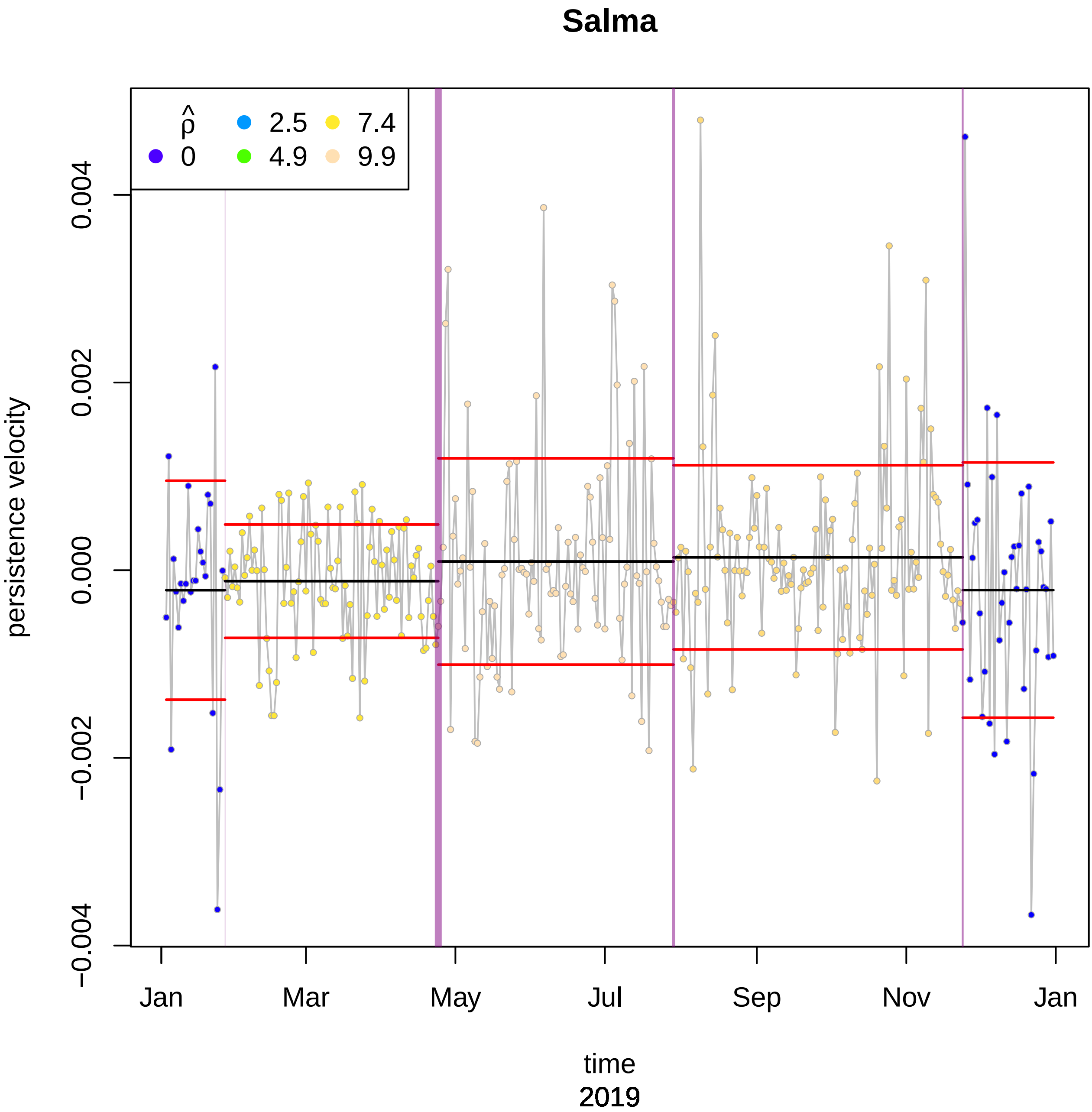

Salma
ρ̂
0
2.5
4.9
7.4
9.9
persistence velocity
0.004
0.002
0.000
−0.002
−0.004
Jan
Mar
May
Jul
Sep
Nov
Jan
time
2019

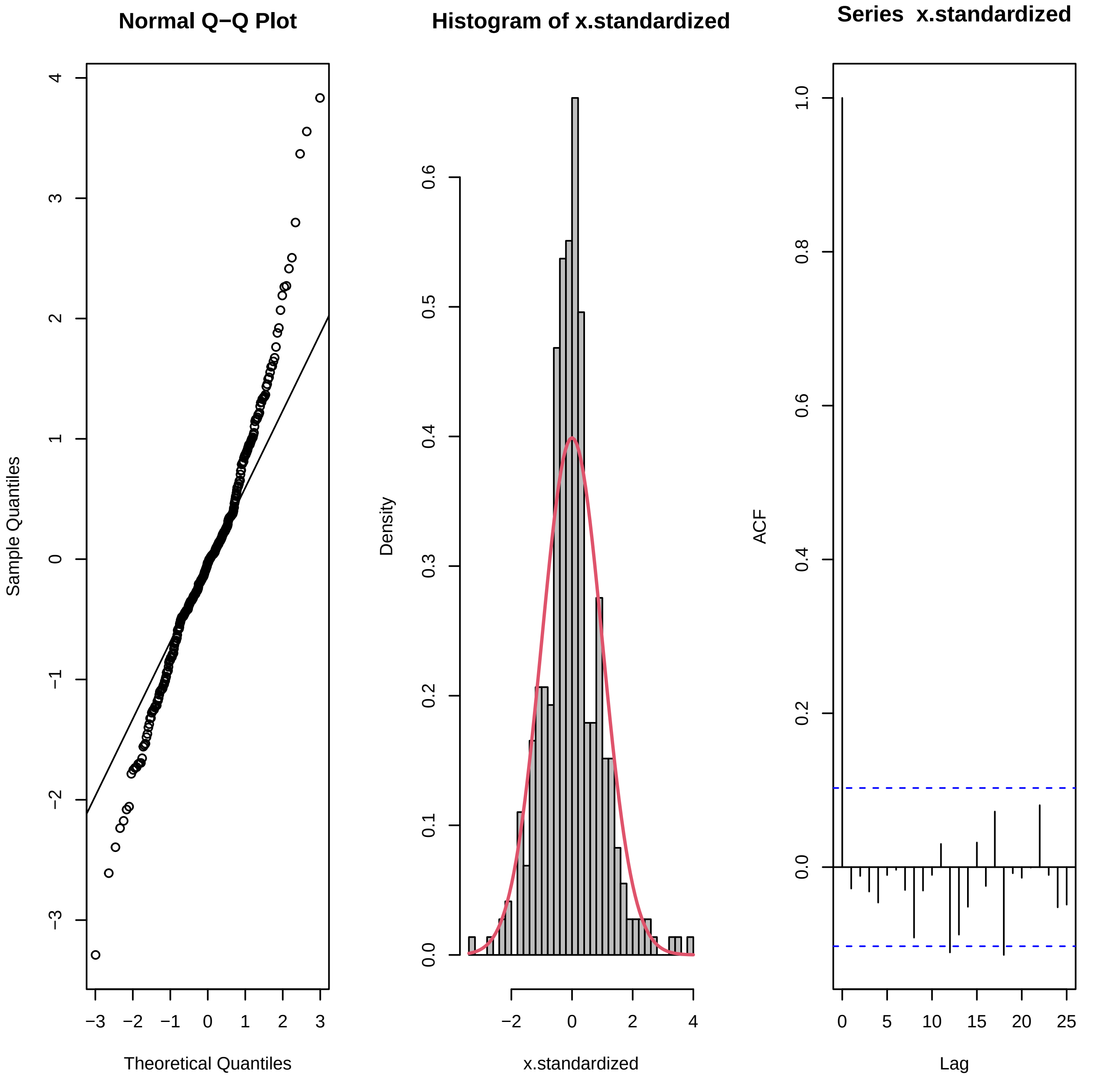
Normal Q−Q Plot
Sample Quantiles
Theoretical Quantiles
−3
−2
−1
0
1
2
3
4
Histogram of x.standardized
Density
x.standardized
0.0
0.1
0.2
0.3
0.4
0.5
0.6
−2
0
2
4
Series x.standardized
ACF
Lag
0.0
0.2
0.4
0.6
0.8
1.0
0
5
10
15
20
25

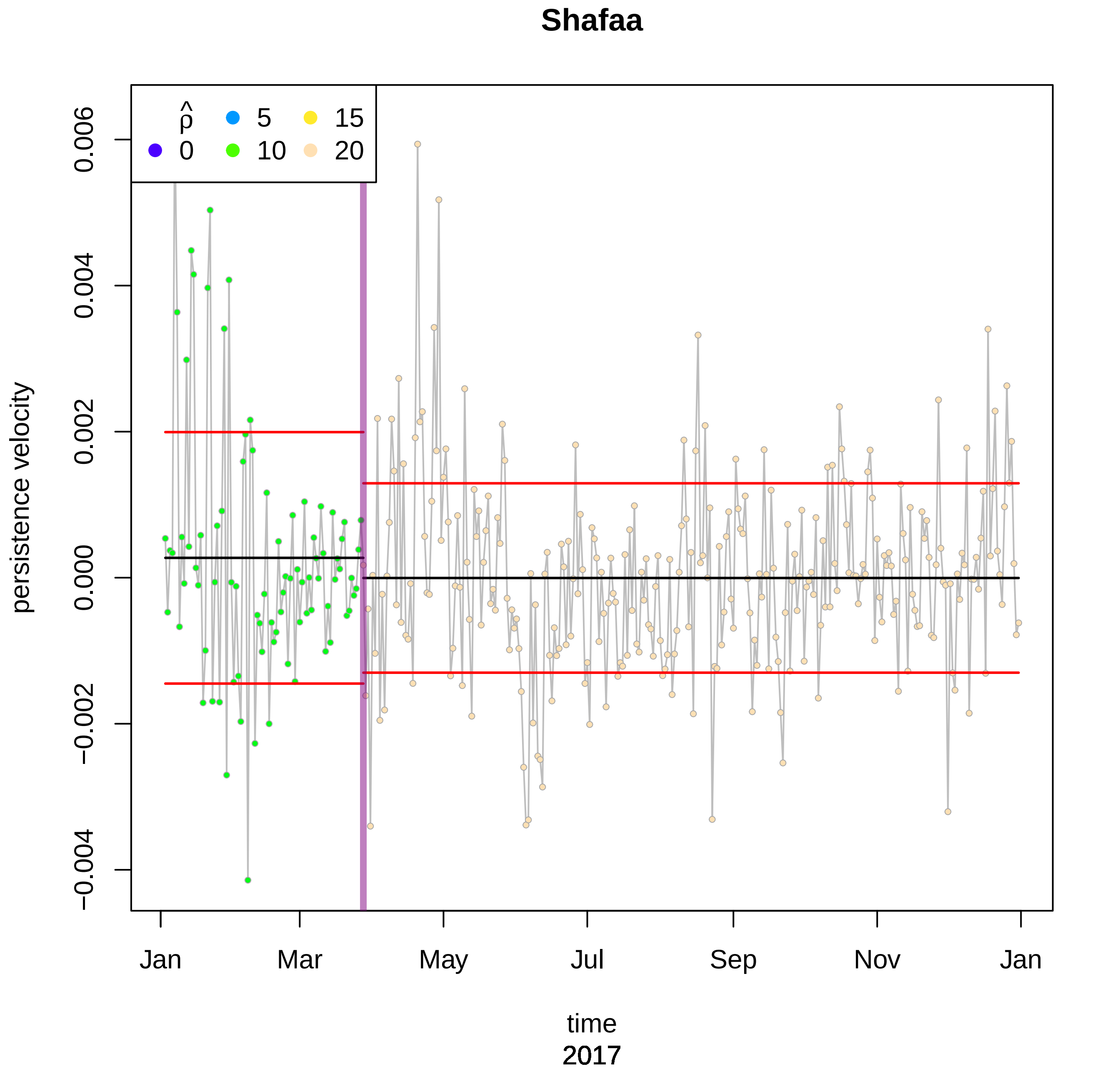

Shafaa
ρ̂
0
5
10
15
20
persistence velocity
0.006
0.004
0.002
0.000
−0.002
−0.004
Jan
Mar
May
Jul
Sep
Nov
Jan
time
2017

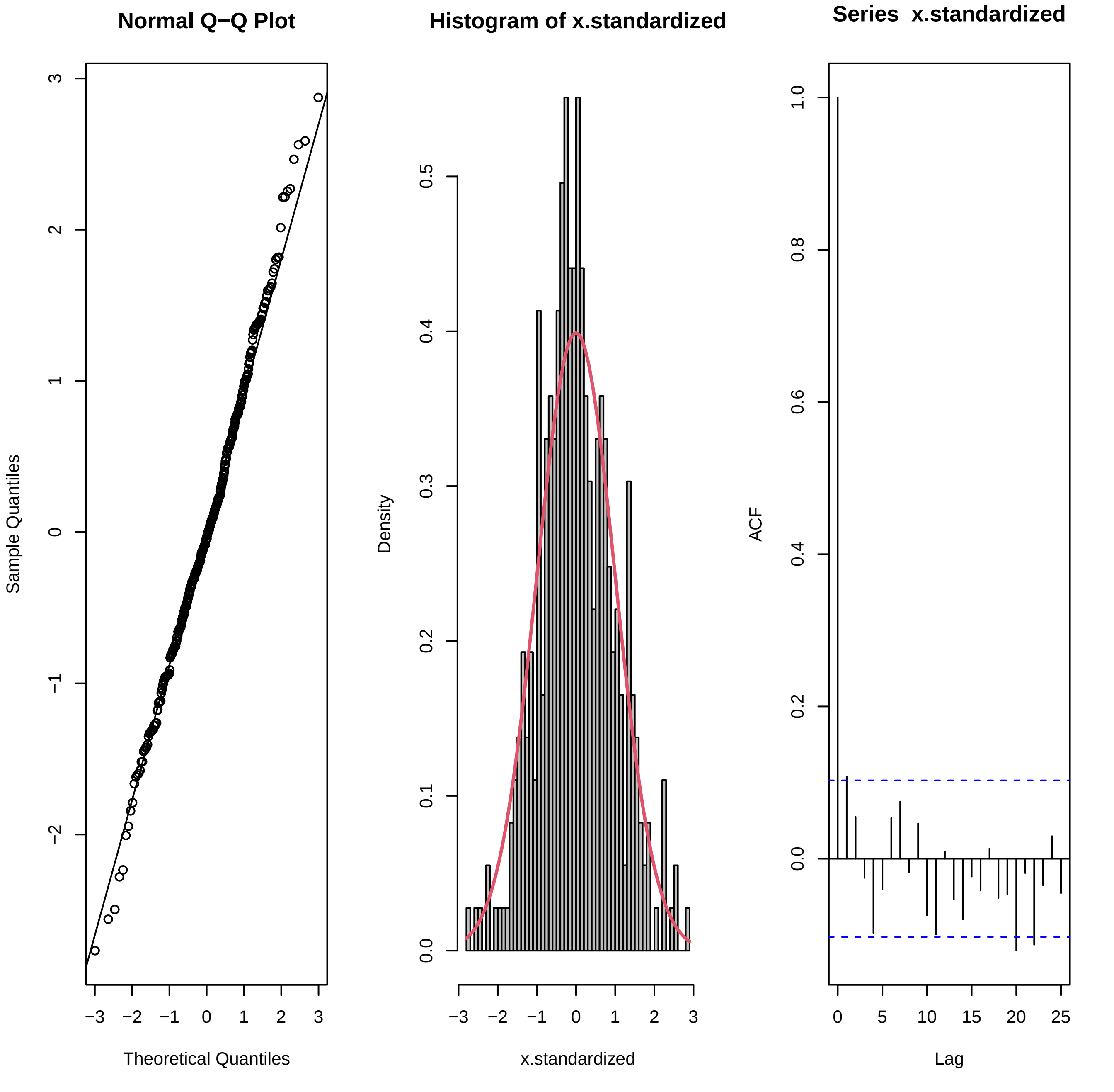

Normal Q−Q Plot
Sample Quantiles
Theoretical Quantiles
−3 −2 −1 0 1 2 3
Histogram of x.standardized
Density
x.standardized
0.0 0.1 0.2 0.3 0.4 0.5
Series x.standardized
ACF
Lag
0 5 10 15 20 25
0.0 0.2 0.4 0.6 0.8 1.0

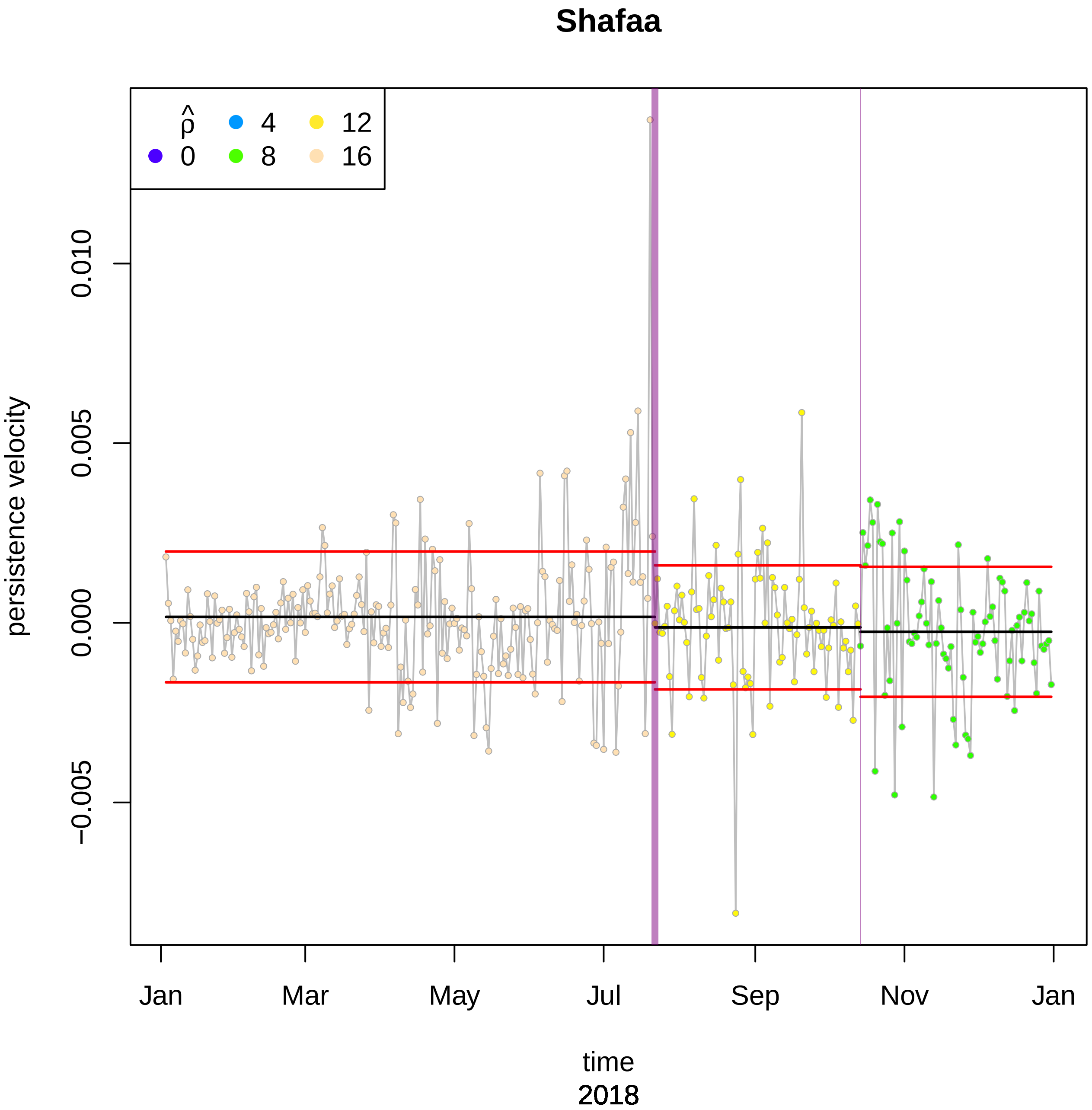

Shafaa
ρ̂
0
4
8
12
16
0.010
0.005
0.000
−0.005
persistence velocity
Jan
Mar
May
Jul
Sep
Nov
Jan
time
2018

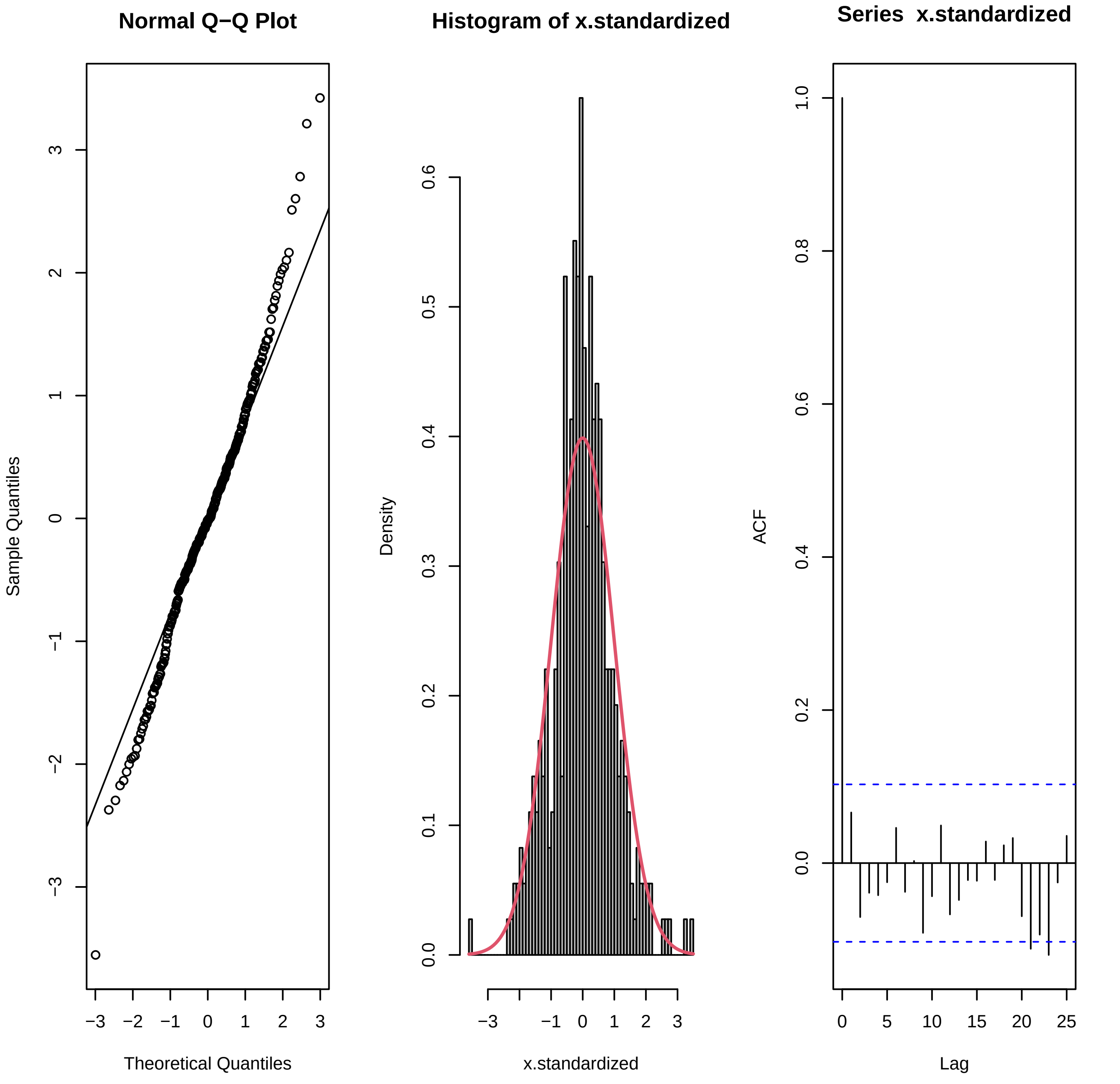

Normal Q−Q Plot
Sample Quantiles
Theoretical Quantiles
−3
−2
−1
0
1
2
3
Histogram of x.standardized
Density
x.standardized
0.0
0.1
0.2
0.3
0.4
0.5
0.6
Series x.standardized
ACF
Lag
0.0
0.2
0.4
0.6
0.8
1.0
0
5
10
15
20
25

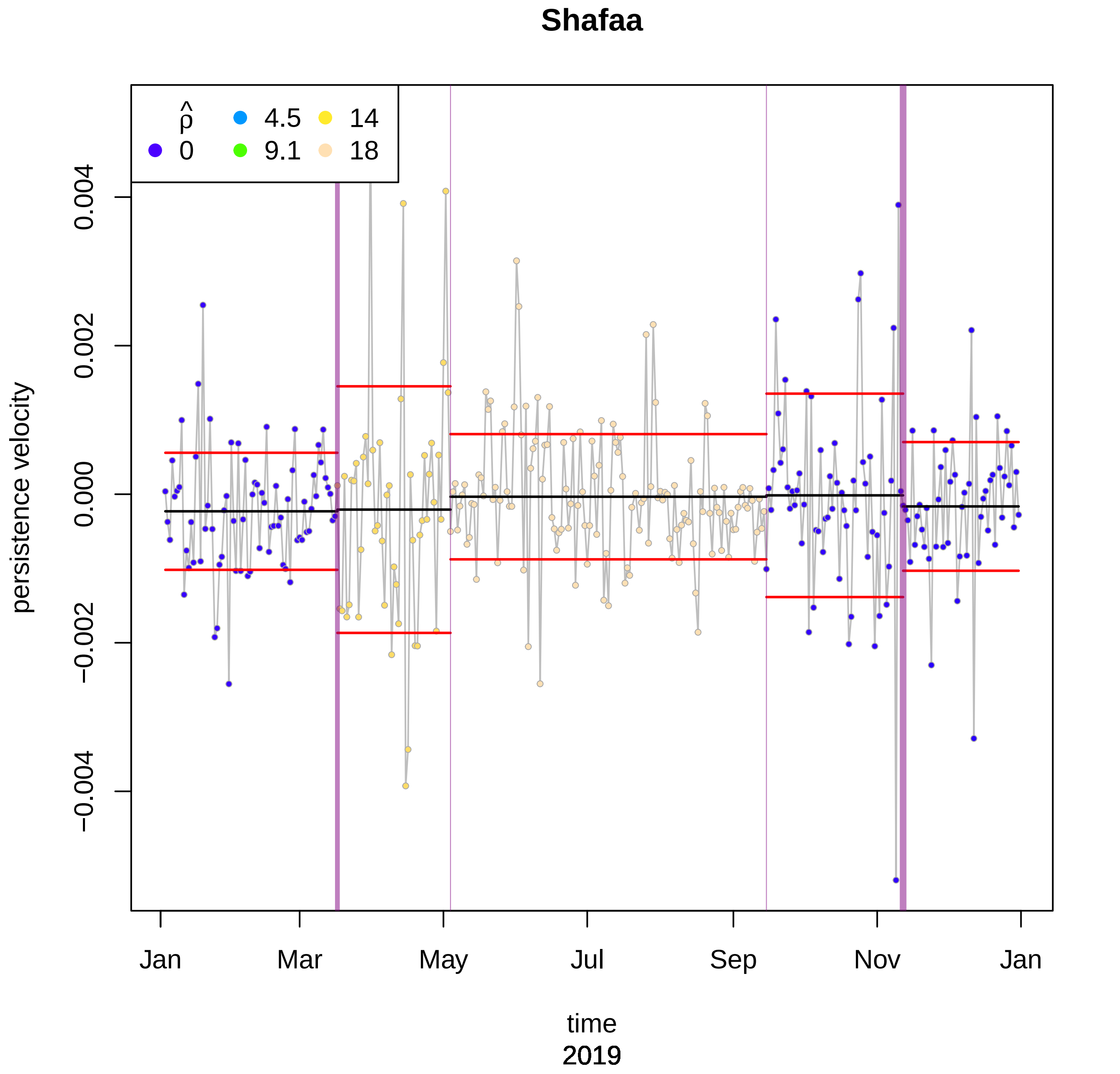
Shafaa
ρ̂
0
4.5
9.1
14
18
persistence velocity
0.004
0.002
0.000
−0.002
−0.004
Jan
Mar
May
Jul
Sep
Nov
Jan
time
2019

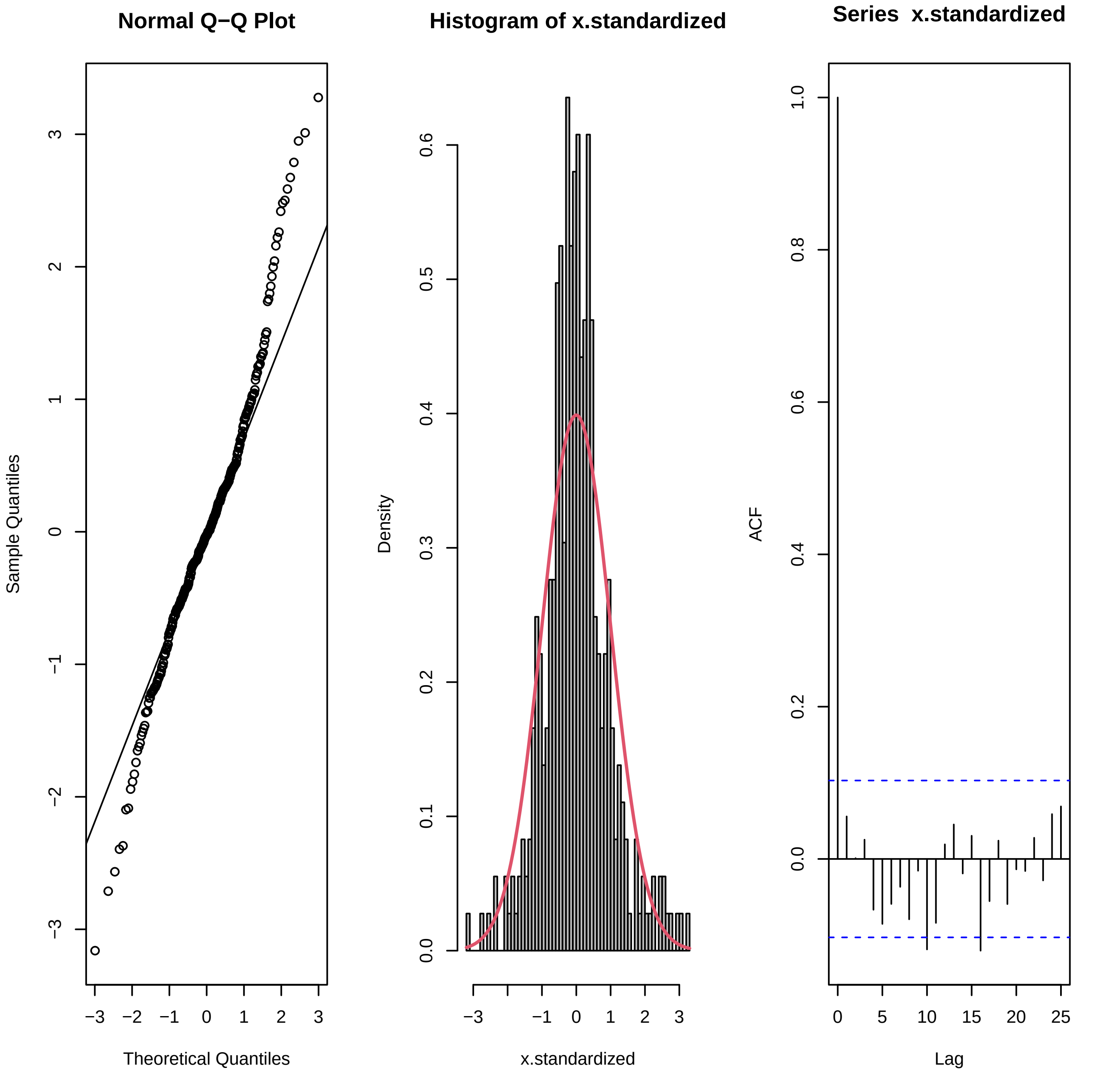
Normal Q−Q Plot
Sample Quantiles
Theoretical Quantiles
Histogram of x.standardized
Density
x.standardized
Series x.standardized
ACF
Lag

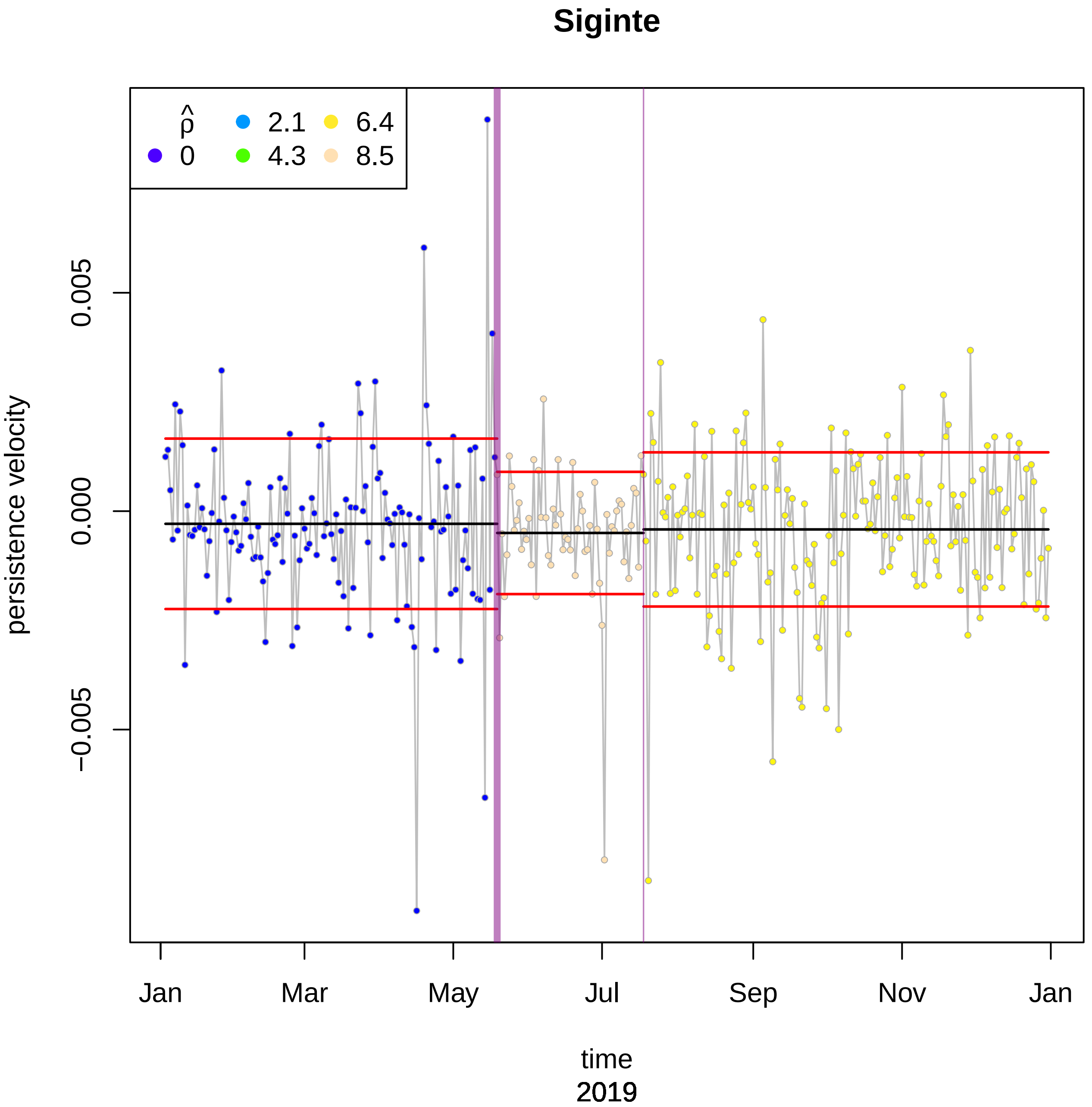

Siginte
$\hat{\rho}$
0
2.1
4.3
6.4
8.5
persistence velocity
0.005
0.000
−0.005
Jan
Mar
May
Jul
Sep
Nov
Jan
time
2019

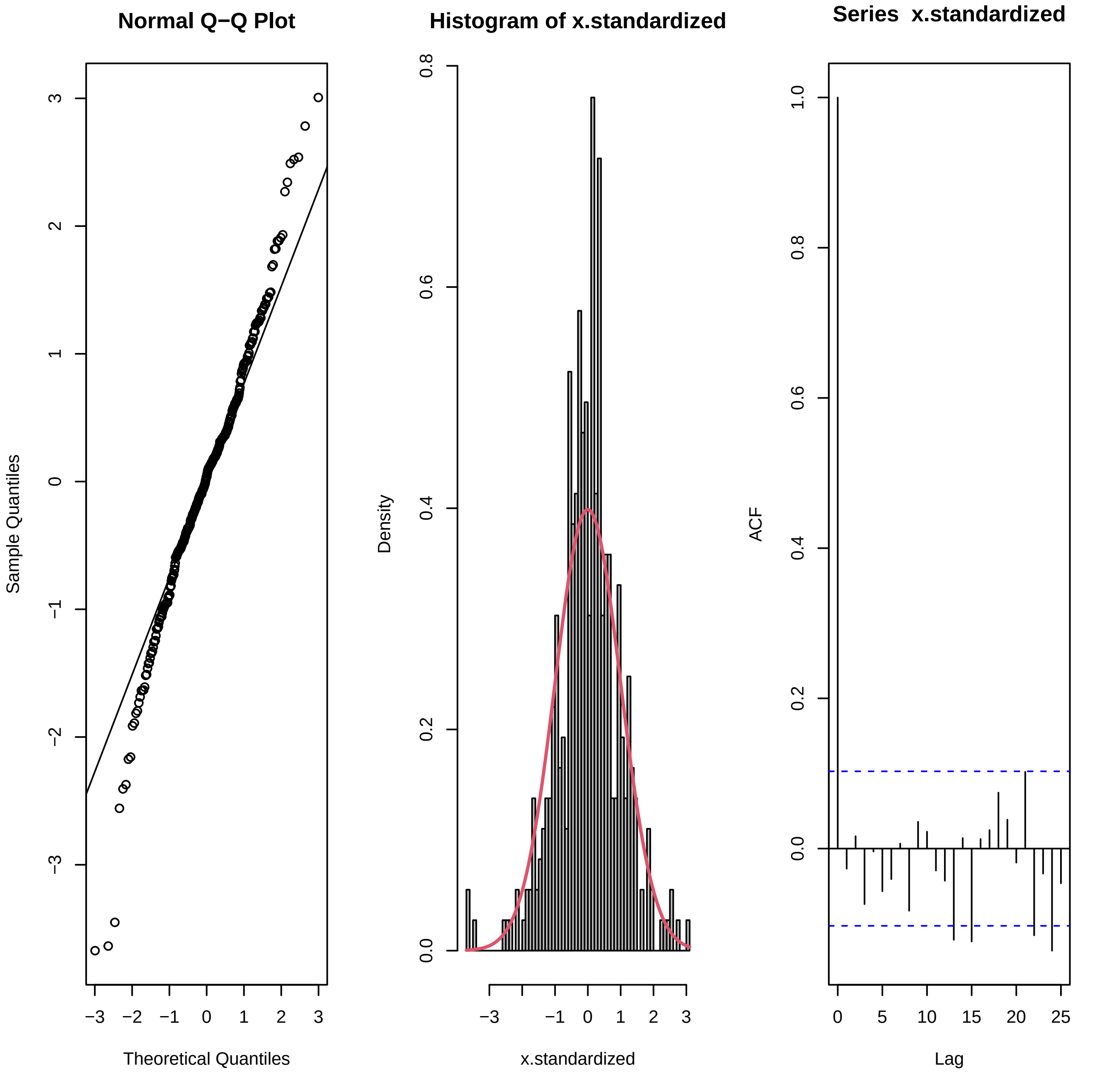

Normal Q−Q Plot
Sample Quantiles
Theoretical Quantiles
−3 −2 −1 0 1 2 3
Histogram of x.standardized
Density
x.standardized
0.0 0.2 0.4 0.6 0.8
Series x.standardized
ACF
Lag
0 5 10 15 20 25
0.0 0.2 0.4 0.6 0.8 1.0

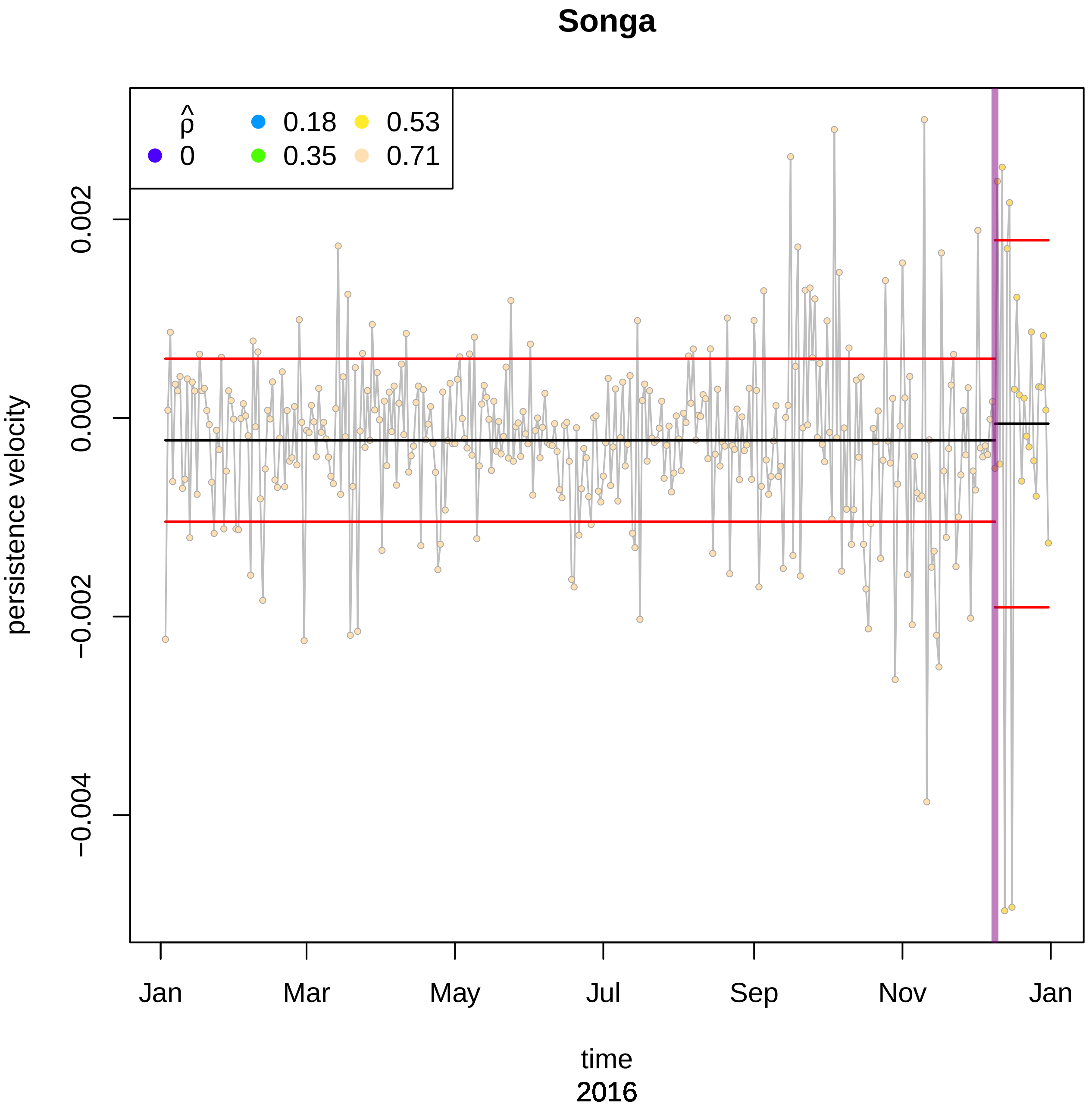
Songa
ρ̂
0
0.18
0.35
0.53
0.71
0.002
0.000
−0.002
−0.004
persistence velocity
Jan
Mar
May
Jul
Sep
Nov
Jan
time
2016

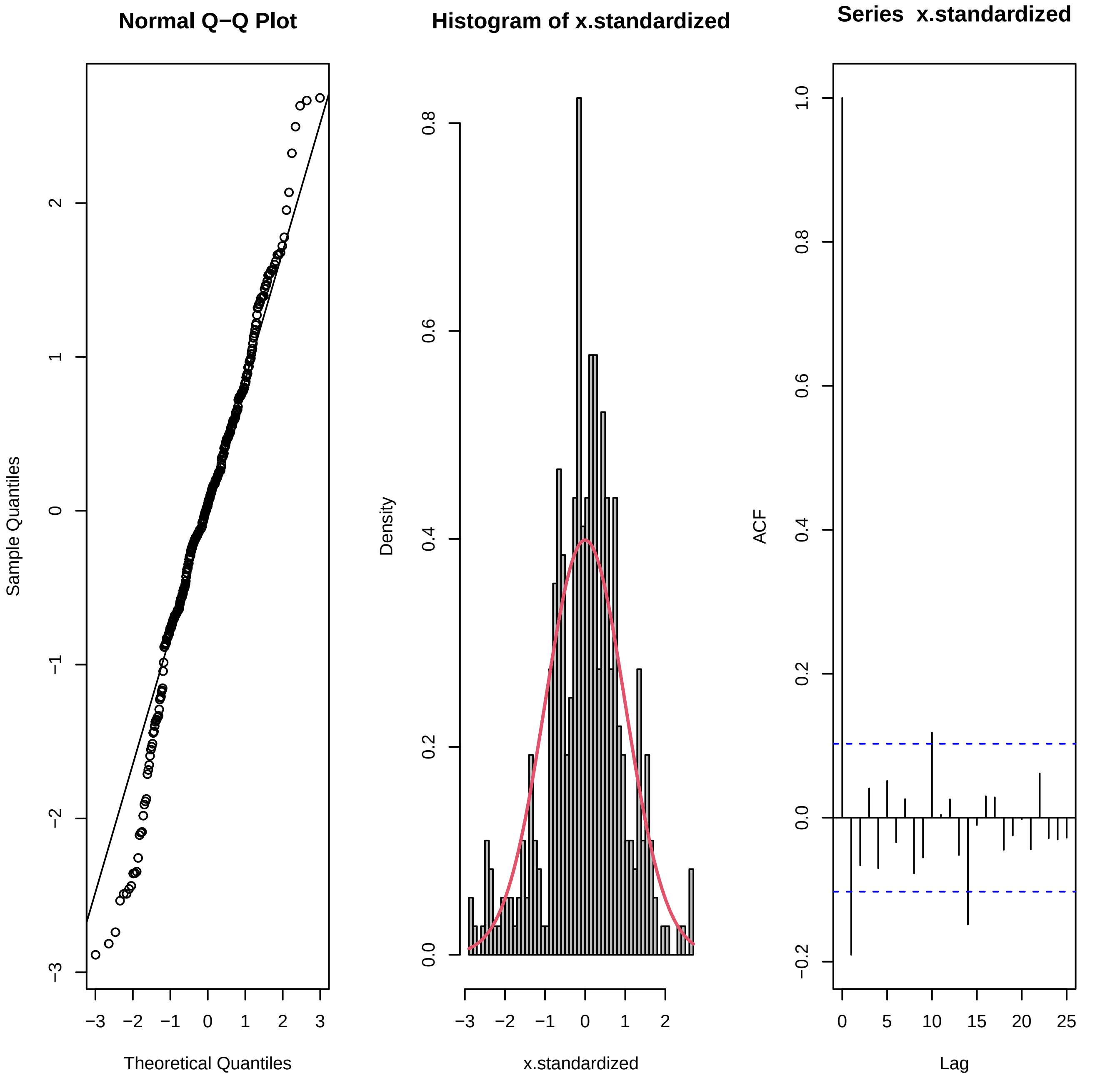
Normal Q−Q Plot
Sample Quantiles
Theoretical Quantiles
−3 −2 −1 0 1 2 3
Histogram of x.standardized
Density
x.standardized
0.0 0.2 0.4 0.6 0.8
Series x.standardized
ACF
Lag
0 5 10 15 20 25
−0.2 0.0 0.2 0.4 0.6 0.8 1.0

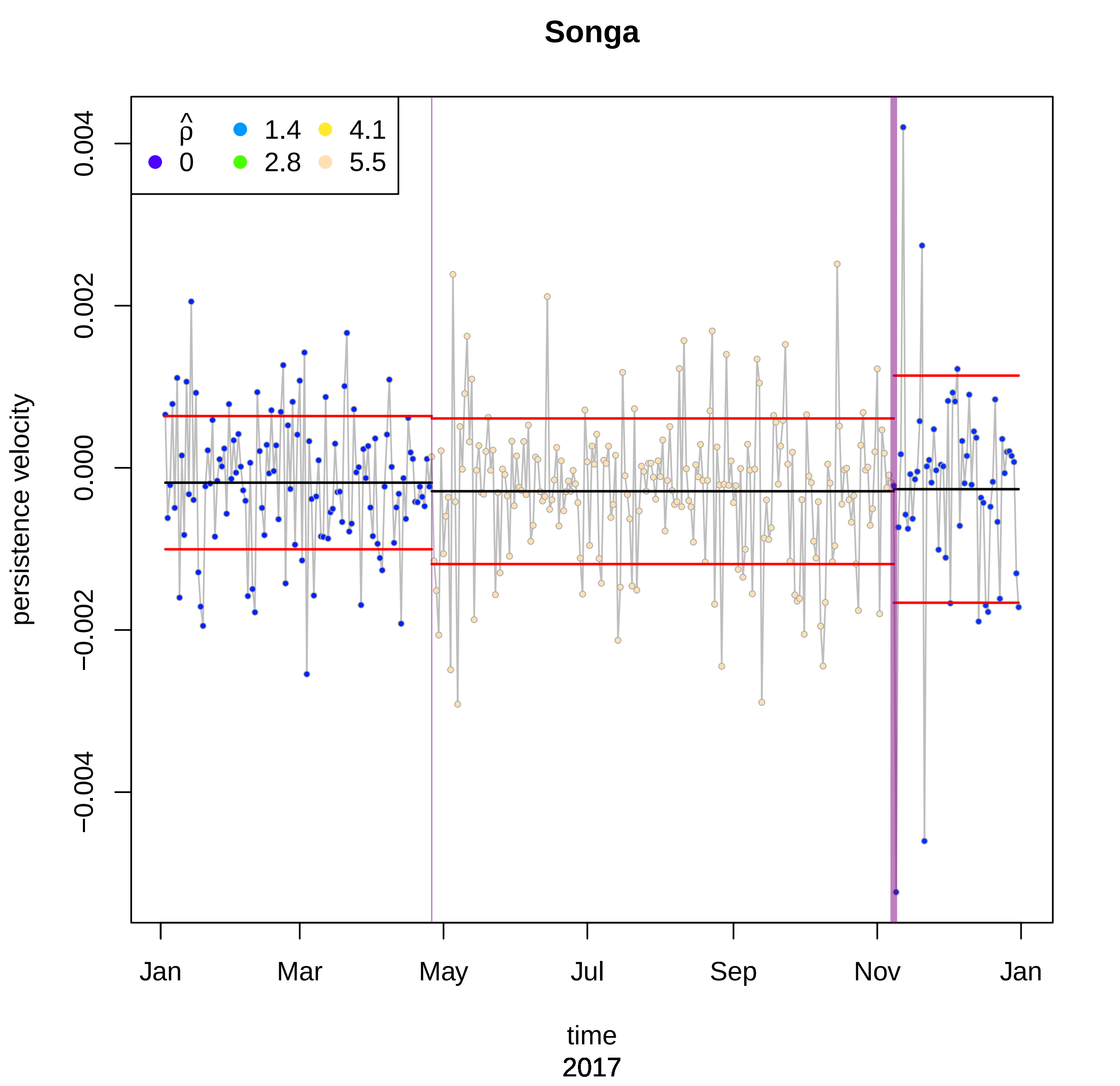
Songa
ρ̂
0
1.4
2.8
4.1
5.5
persistence velocity
0.004
0.002
0.000
−0.002
−0.004
Jan
Mar
May
Jul
Sep
Nov
Jan
time
2017

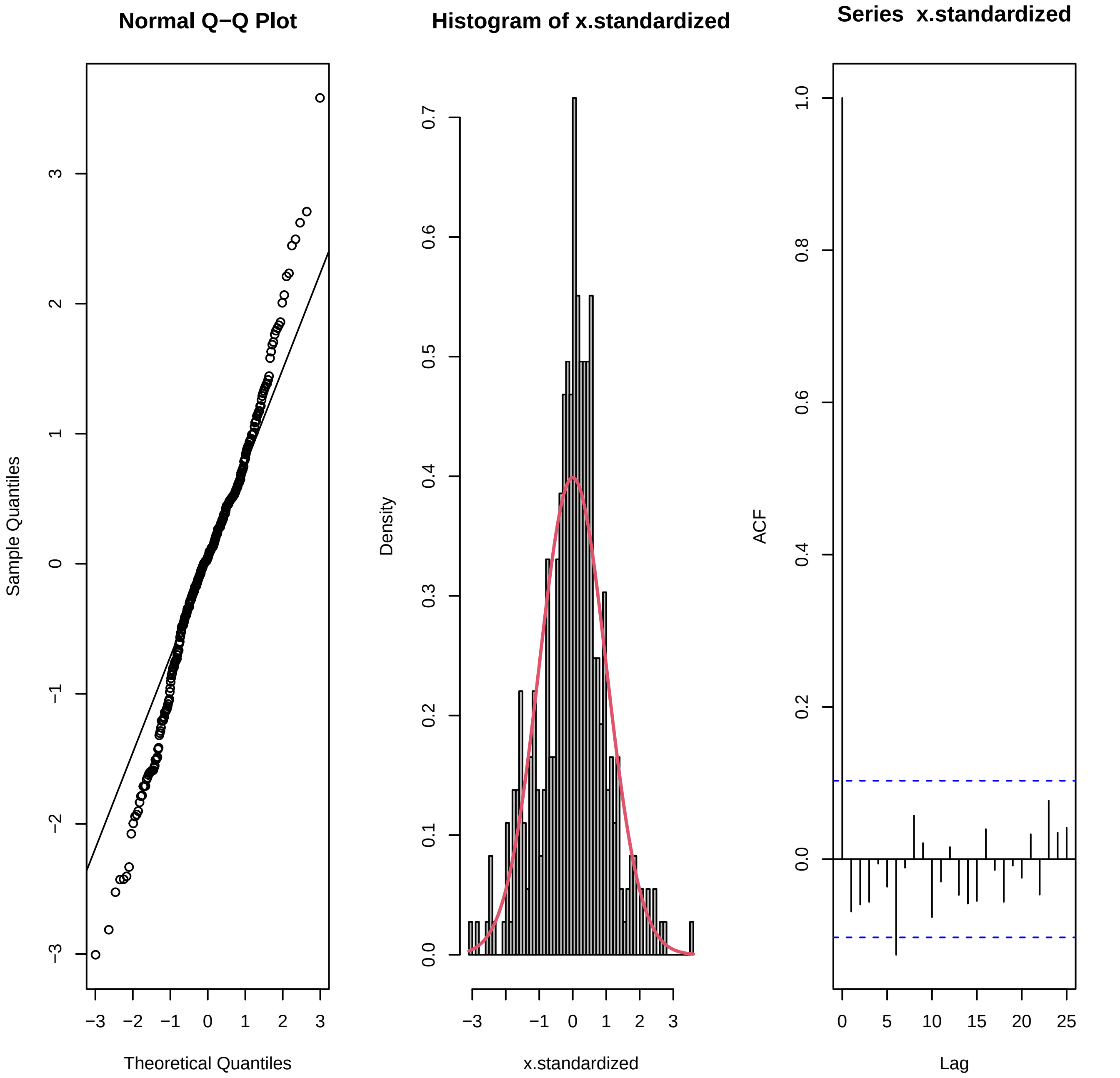

Normal Q−Q Plot
Sample Quantiles
Theoretical Quantiles
Histogram of x.standardized
Density
x.standardized
Series x.standardized
ACF
Lag

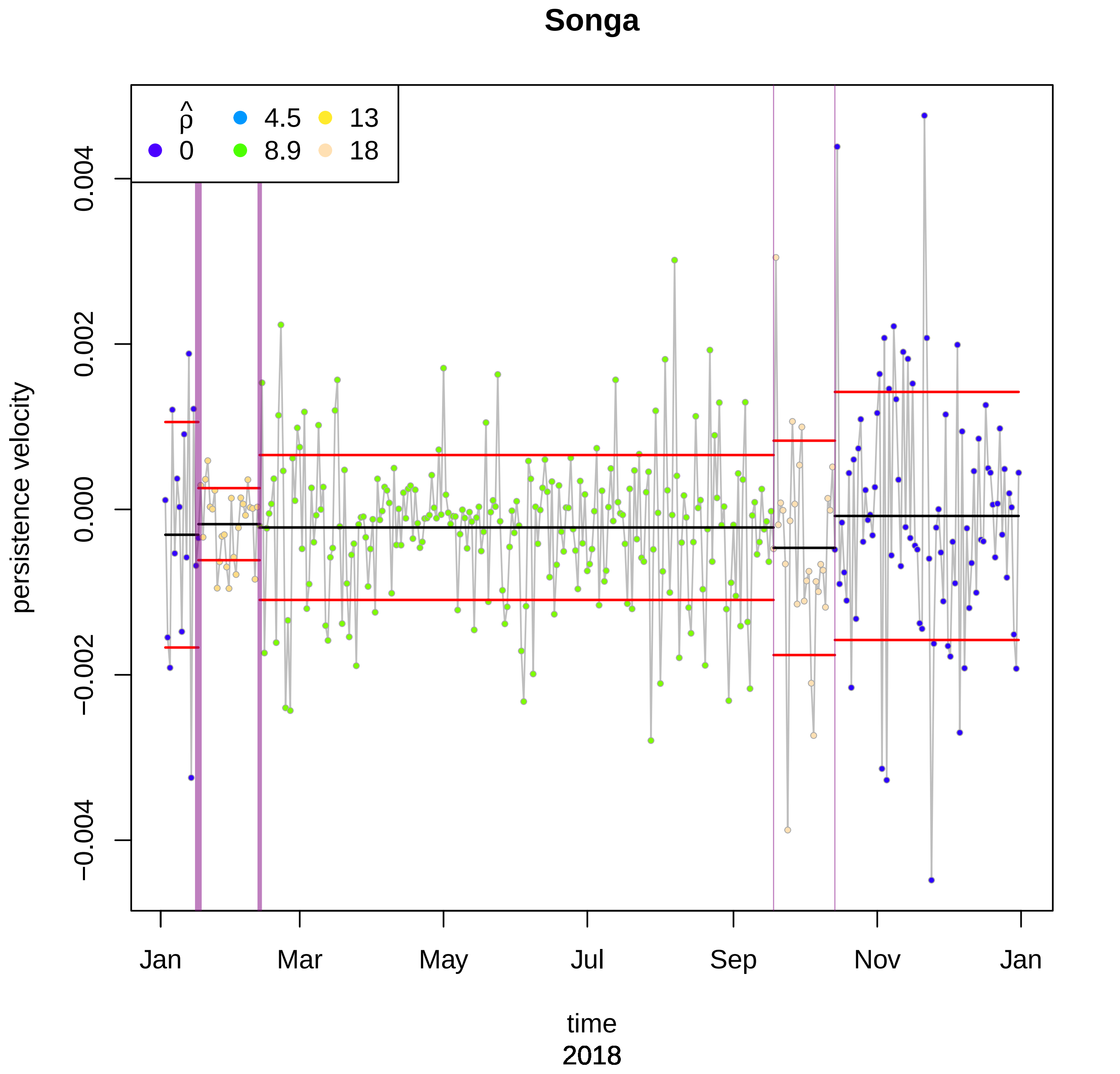

Songa
ρ̂
0
4.5
8.9
13
18
0.004
0.002
0.000
−0.002
−0.004
persistence velocity
Jan
Mar
May
Jul
Sep
Nov
Jan
time
2018

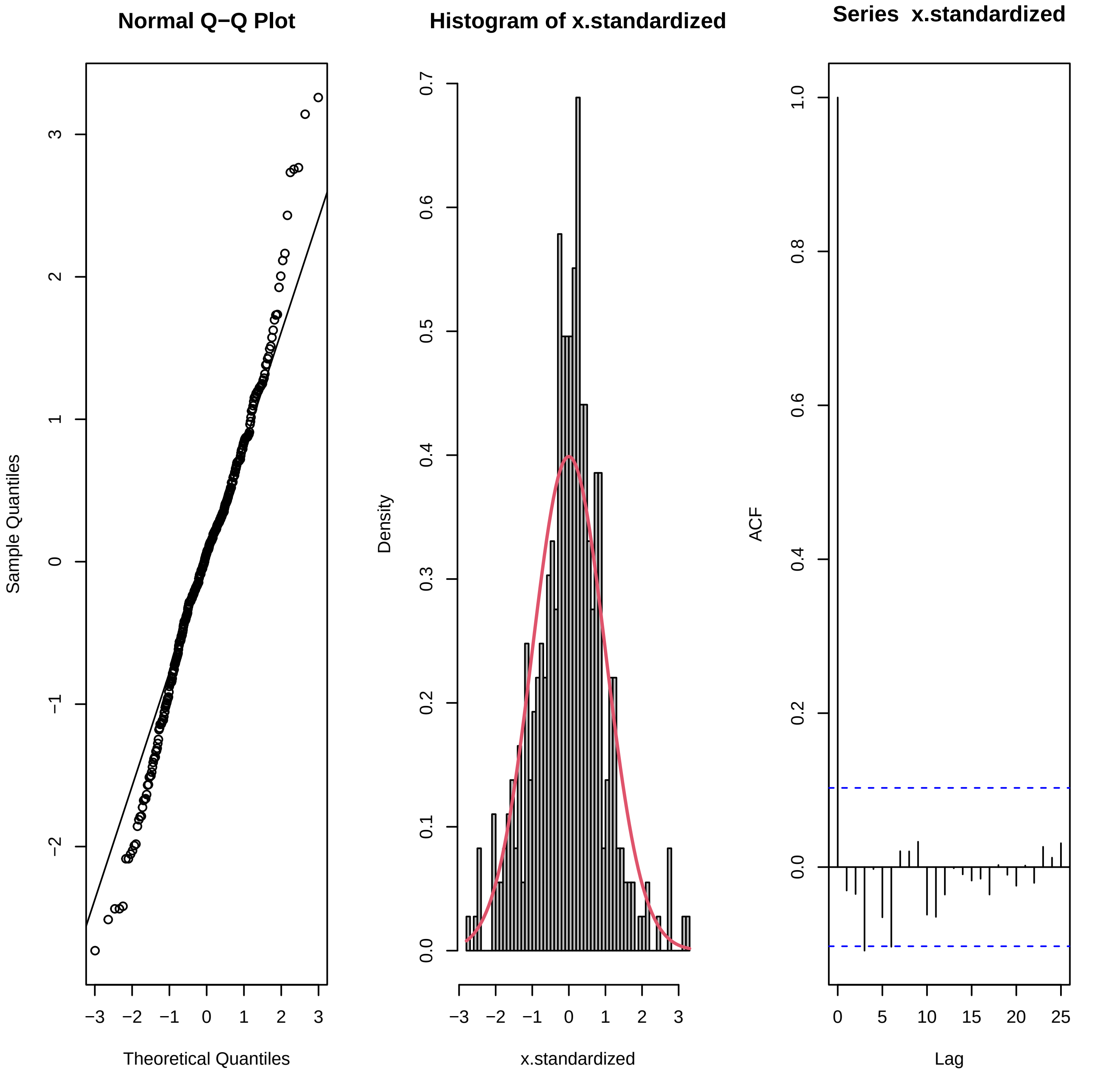

Normal Q−Q Plot
Sample Quantiles
Theoretical Quantiles
−3 −2 −1 0 1 2 3
Histogram of x.standardized
Density
x.standardized
0.0 0.1 0.2 0.3 0.4 0.5 0.6 0.7
Series x.standardized
ACF
Lag
0.0 0.2 0.4 0.6 0.8 1.0
0 5 10 15 20 25

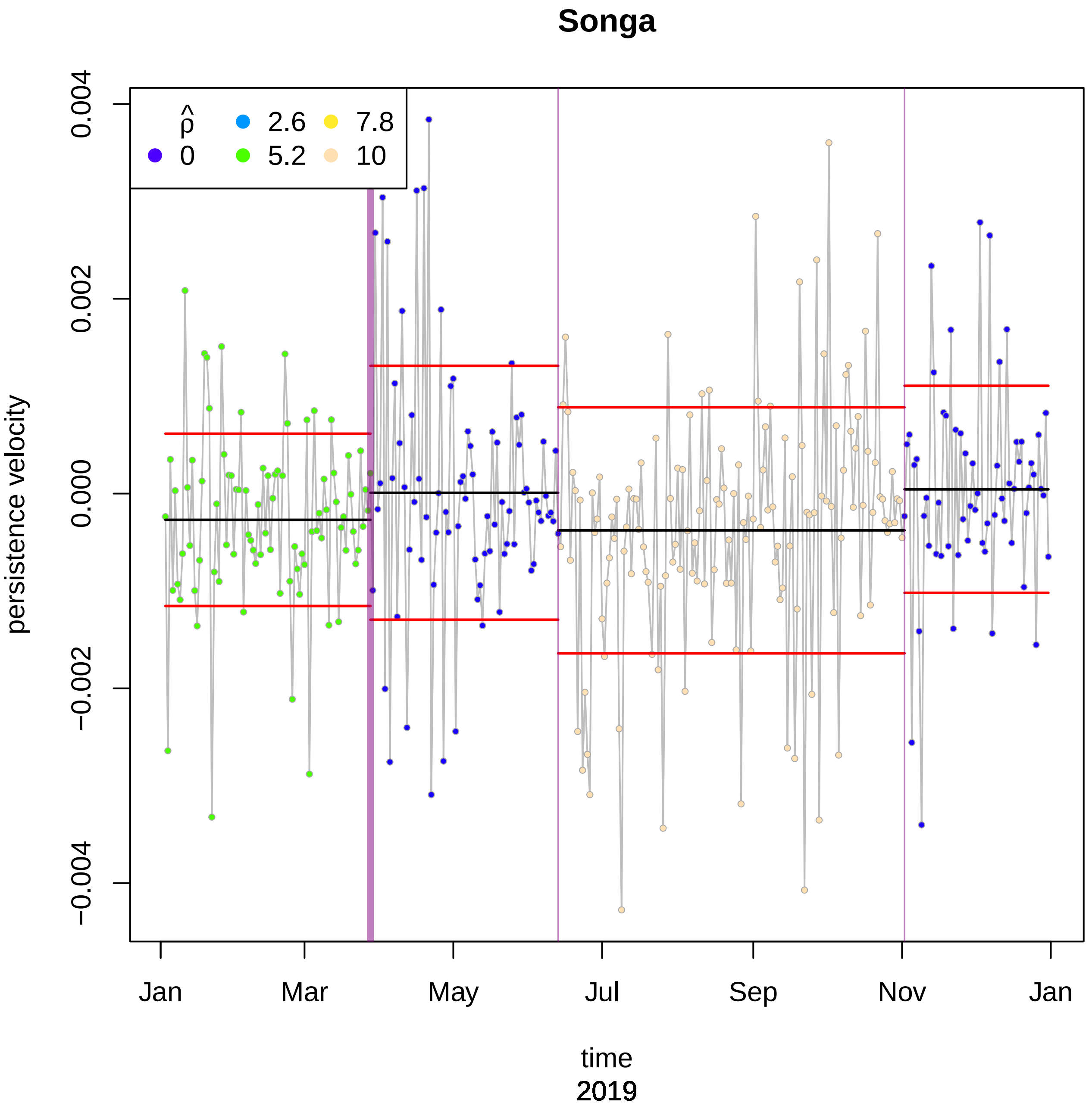

Songa
ρ̂
0
2.6
5.2
7.8
10
0.004
0.002
0.000
−0.002
−0.004
persistence velocity
Jan
Mar
May
Jul
Sep
Nov
Jan
time
2019

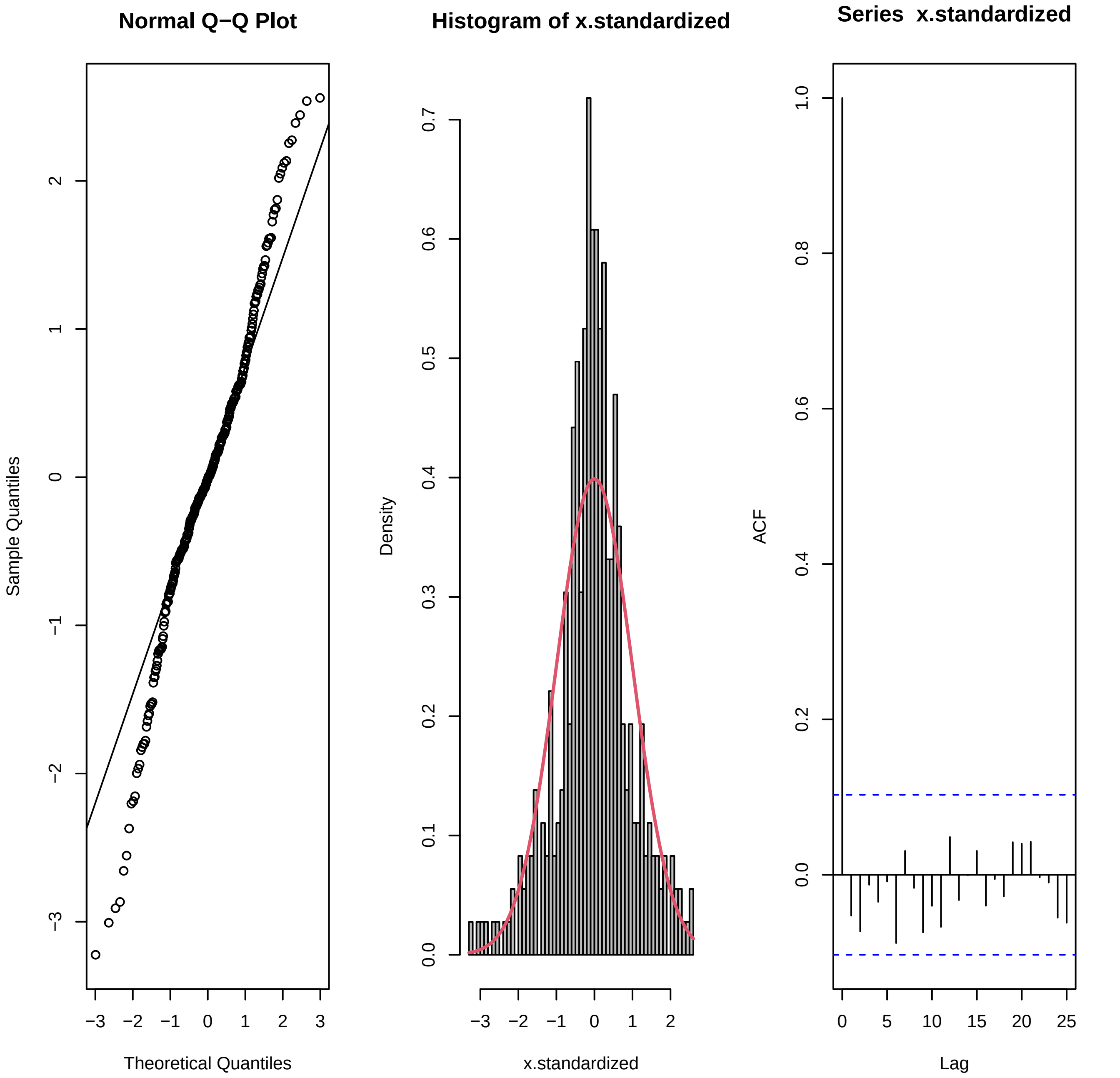

Normal Q−Q Plot
Sample Quantiles
Theoretical Quantiles
−3
−2
−1
0
1
2
3
Histogram of x.standardized
Density
x.standardized
0.0
0.1
0.2
0.3
0.4
0.5
0.6
0.7
Series x.standardized
ACF
Lag
0
5
10
15
20
25
0.0
0.2
0.4
0.6
0.8
1.0

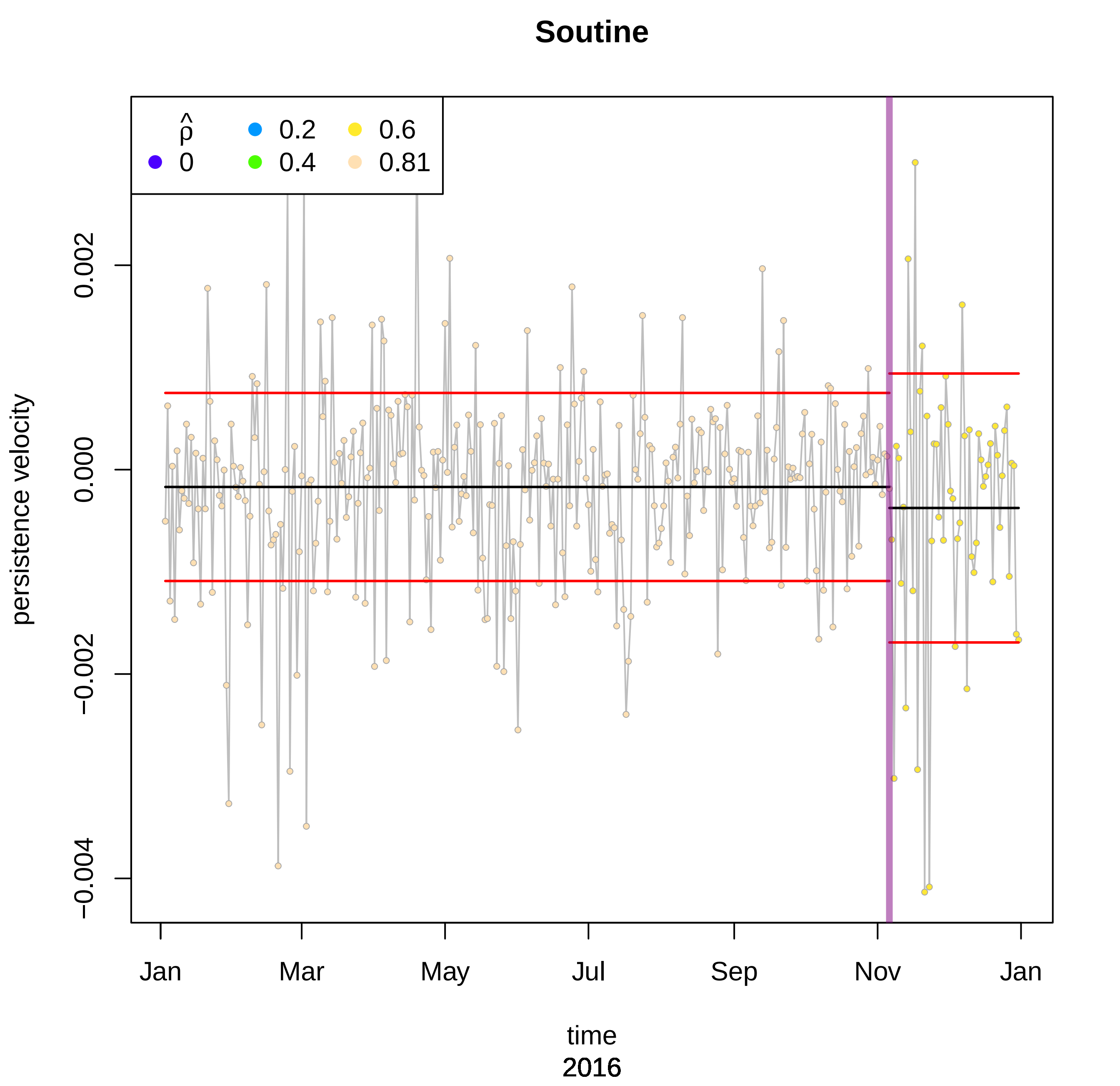

Soutine
ρ̂
0
0.2
0.4
0.6
0.81
0.002
0.000
−0.002
−0.004
persistence velocity
Jan
Mar
May
Jul
Sep
Nov
Jan
time
2016

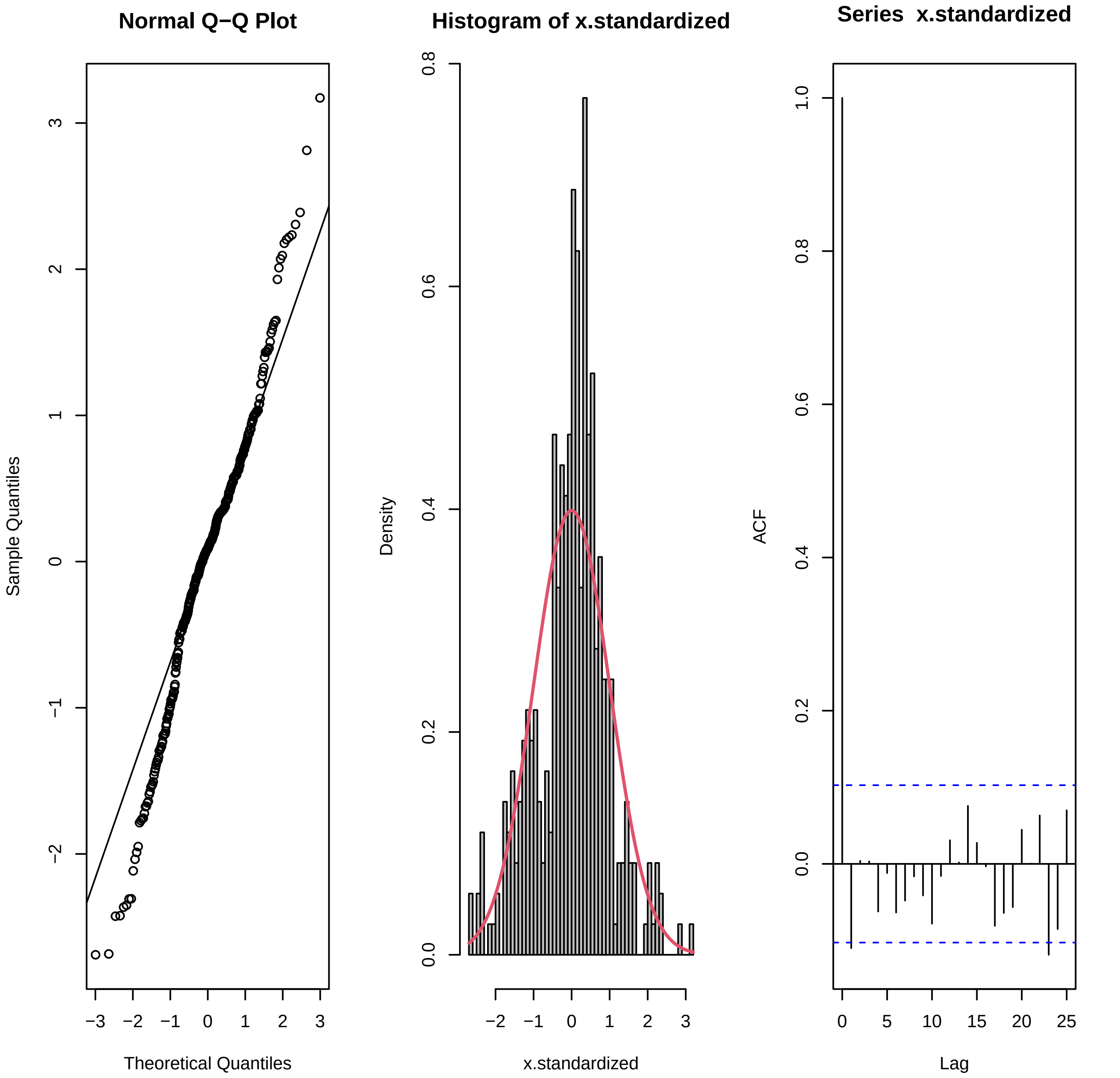

Normal Q−Q Plot
Sample Quantiles
Theoretical Quantiles
Histogram of x.standardized
Density
x.standardized
Series x.standardized
ACF
Lag

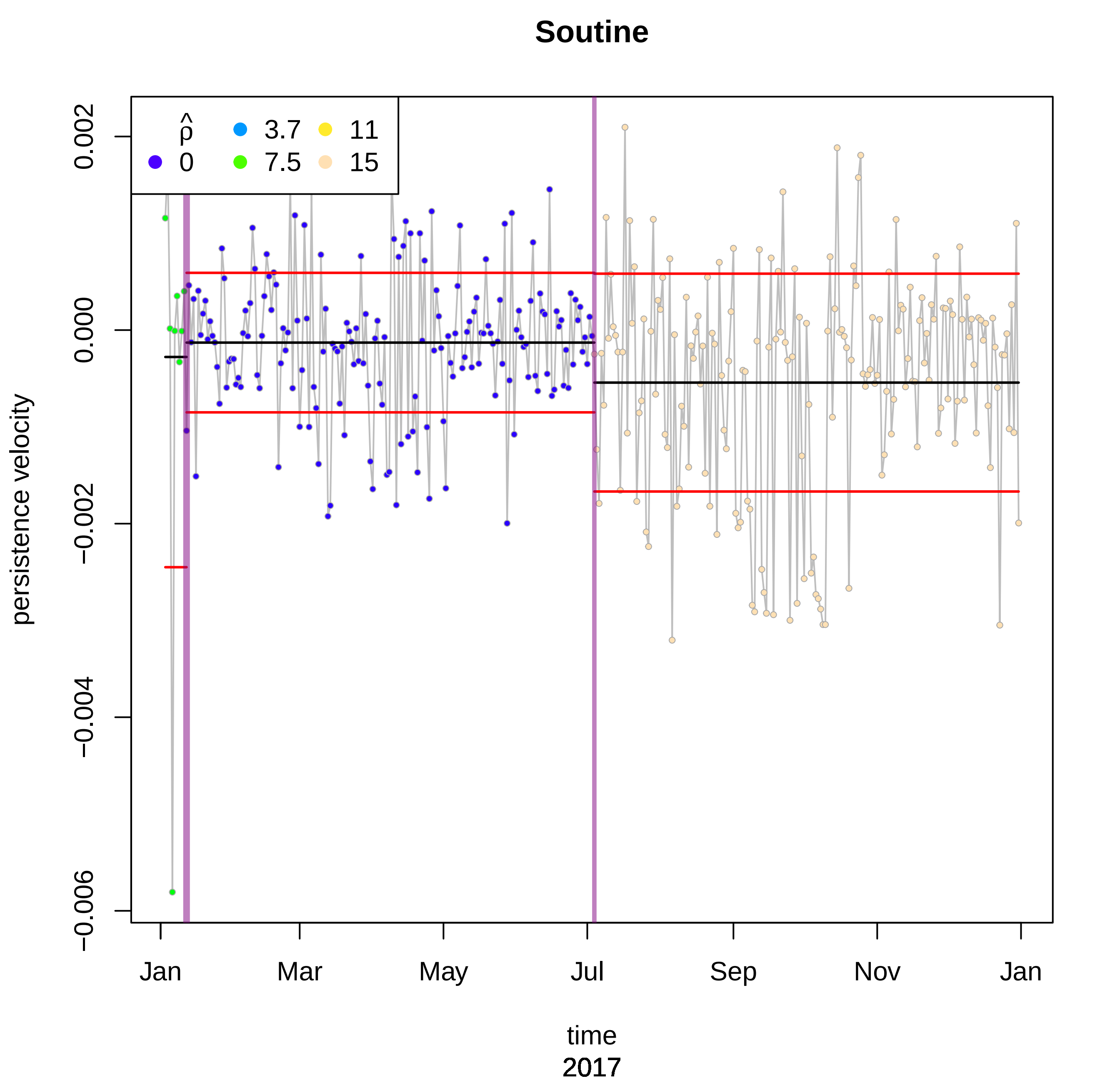

Soutine
ρ̂
0
3.7
7.5
11
15
0.002
0.000
−0.002
−0.004
−0.006
persistence velocity
Jan
Mar
May
Jul
Sep
Nov
Jan
time
2017

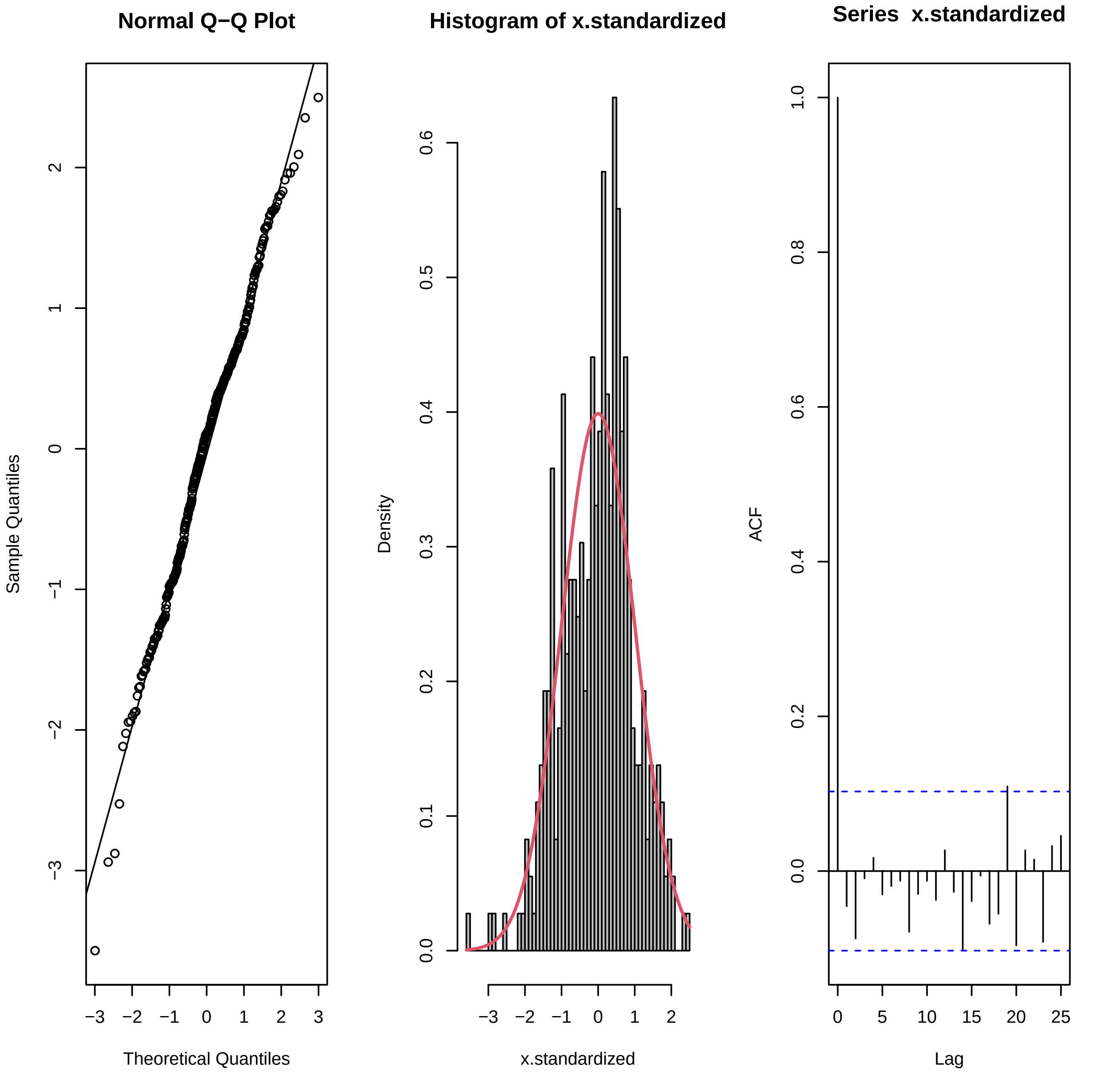

Normal Q−Q Plot
Sample Quantiles
Theoretical Quantiles
−3
−2
−1
0
1
2
3
Histogram of x.standardized
Density
x.standardized
0.0
0.1
0.2
0.3
0.4
0.5
0.6
Series x.standardized
ACF
Lag
0
5
10
15
20
25
0.0
0.2
0.4
0.6
0.8
1.0

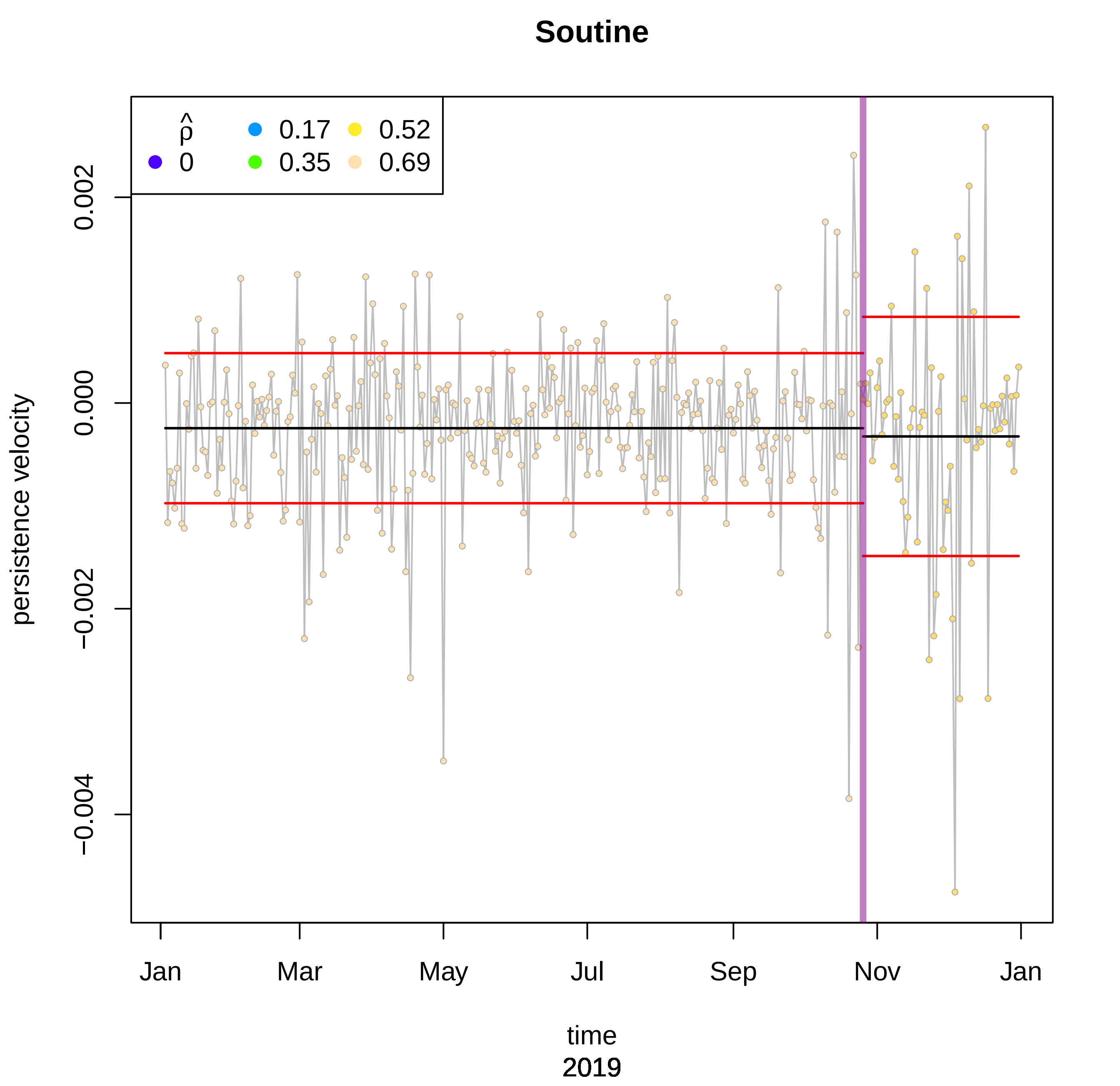

Soutine
ρ̂
0
0.17
0.35
0.52
0.69
0.002
0.000
−0.002
−0.004
persistence velocity
Jan
Mar
May
Jul
Sep
Nov
Jan
time
2019

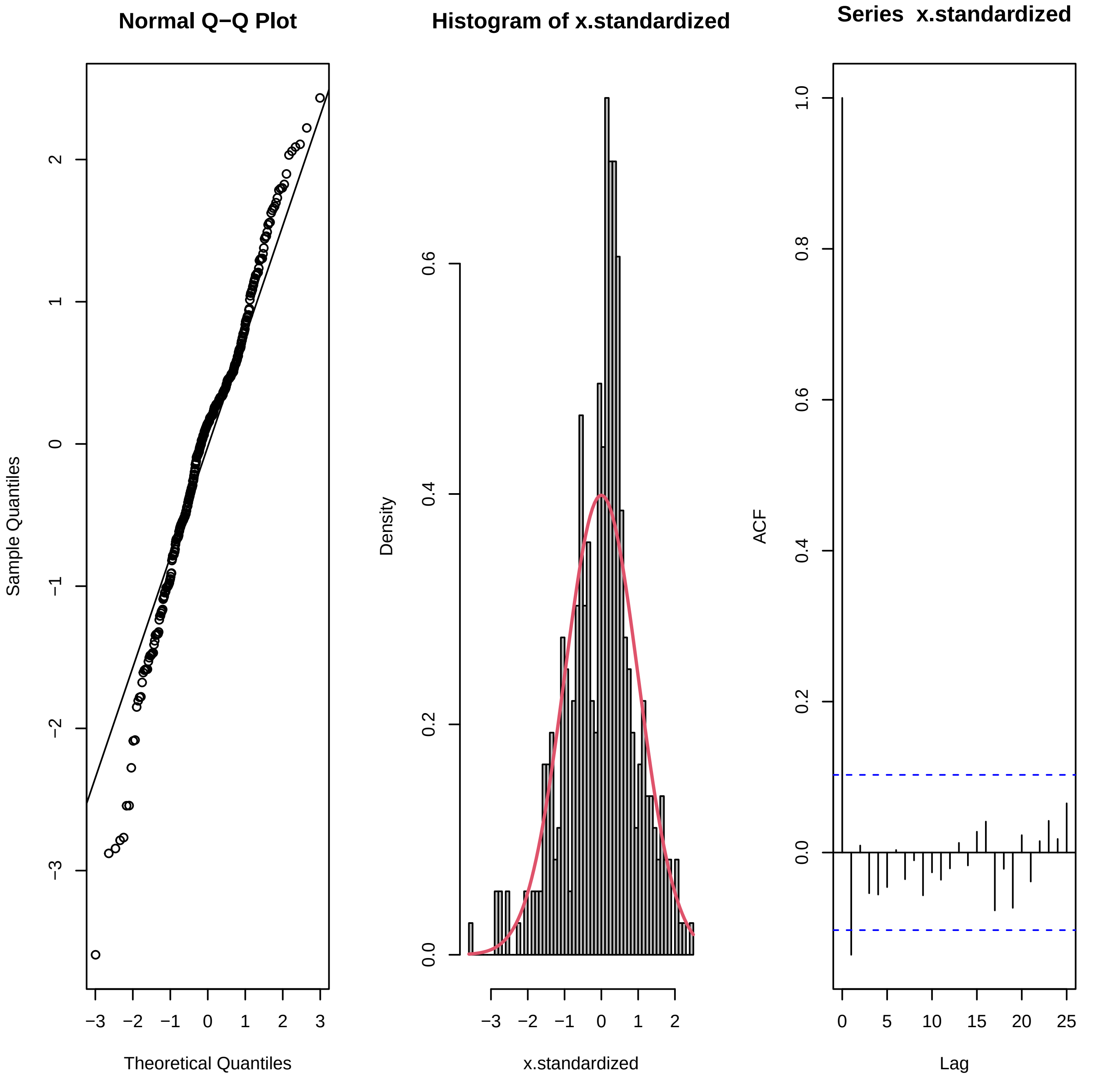

Normal Q−Q Plot
Sample Quantiles
Theoretical Quantiles
−3 −2 −1 0 1 2 3
Histogram of x.standardized
Density
x.standardized
0.0 0.2 0.4 0.6
Series x.standardized
ACF
Lag
0.0 0.2 0.4 0.6 0.8 1.0
0 5 10 15 20 25

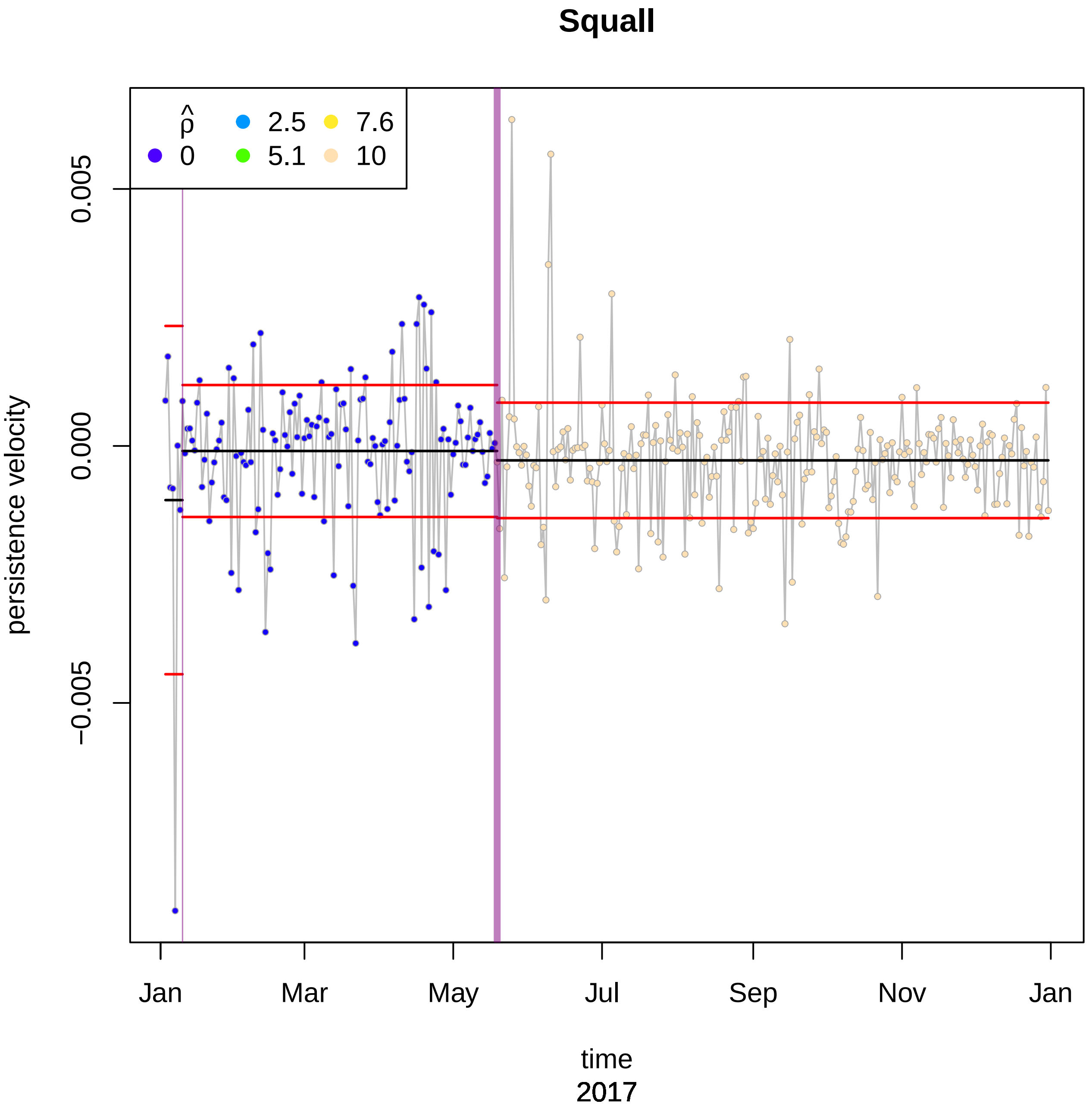
Squall
ρ̂
0
2.5
5.1
7.6
10
0.005
0.000
−0.005
persistence velocity
Jan
Mar
May
Jul
Sep
Nov
Jan
time
2017

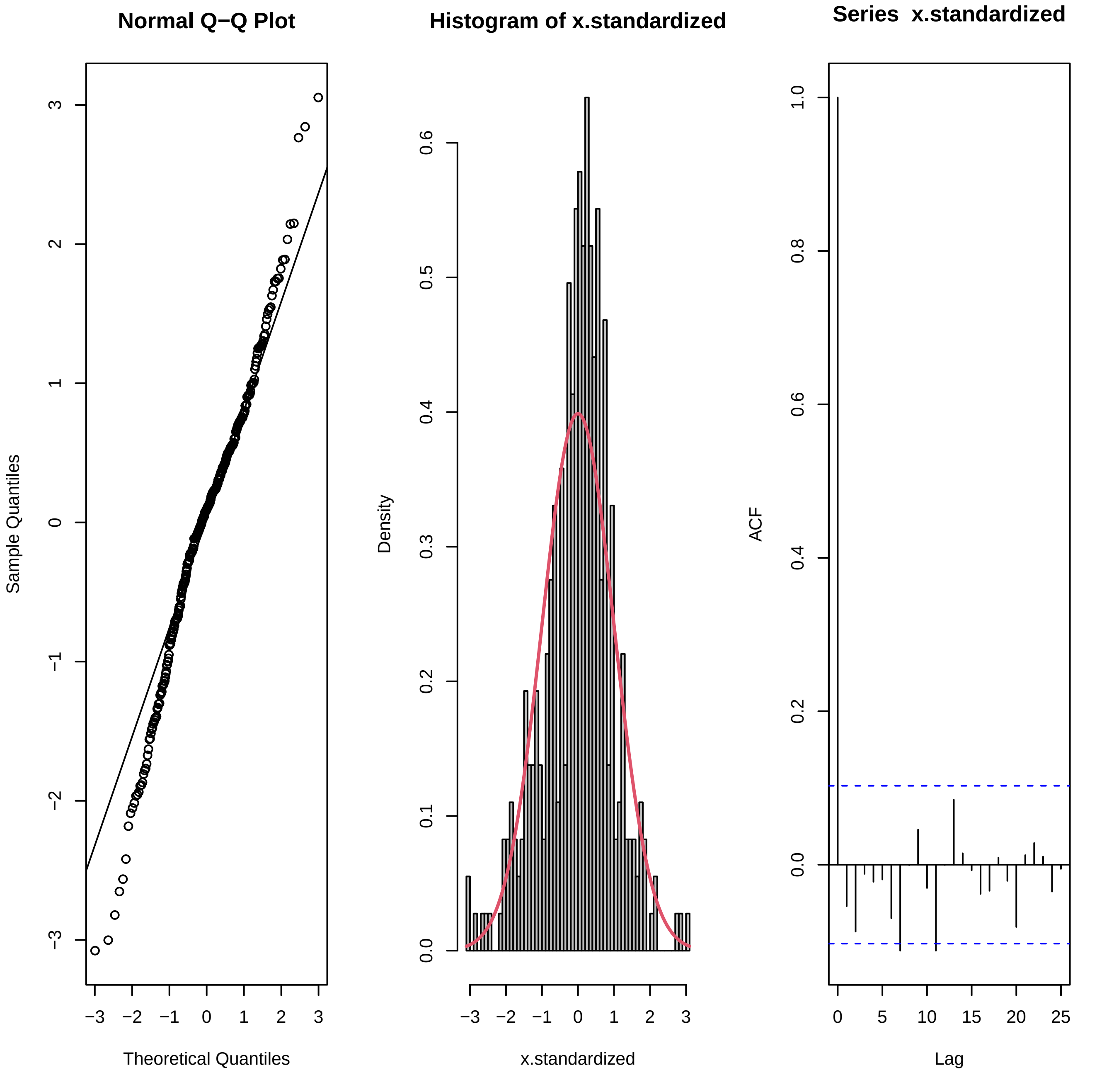

Normal Q−Q Plot
Sample Quantiles
Theoretical Quantiles
−3
−2
−1
0
1
2
3
Histogram of x.standardized
Density
x.standardized
0.0
0.1
0.2
0.3
0.4
0.5
0.6
Series x.standardized
ACF
Lag
0.0
0.2
0.4
0.6
0.8
1.0
0
5
10
15
20
25

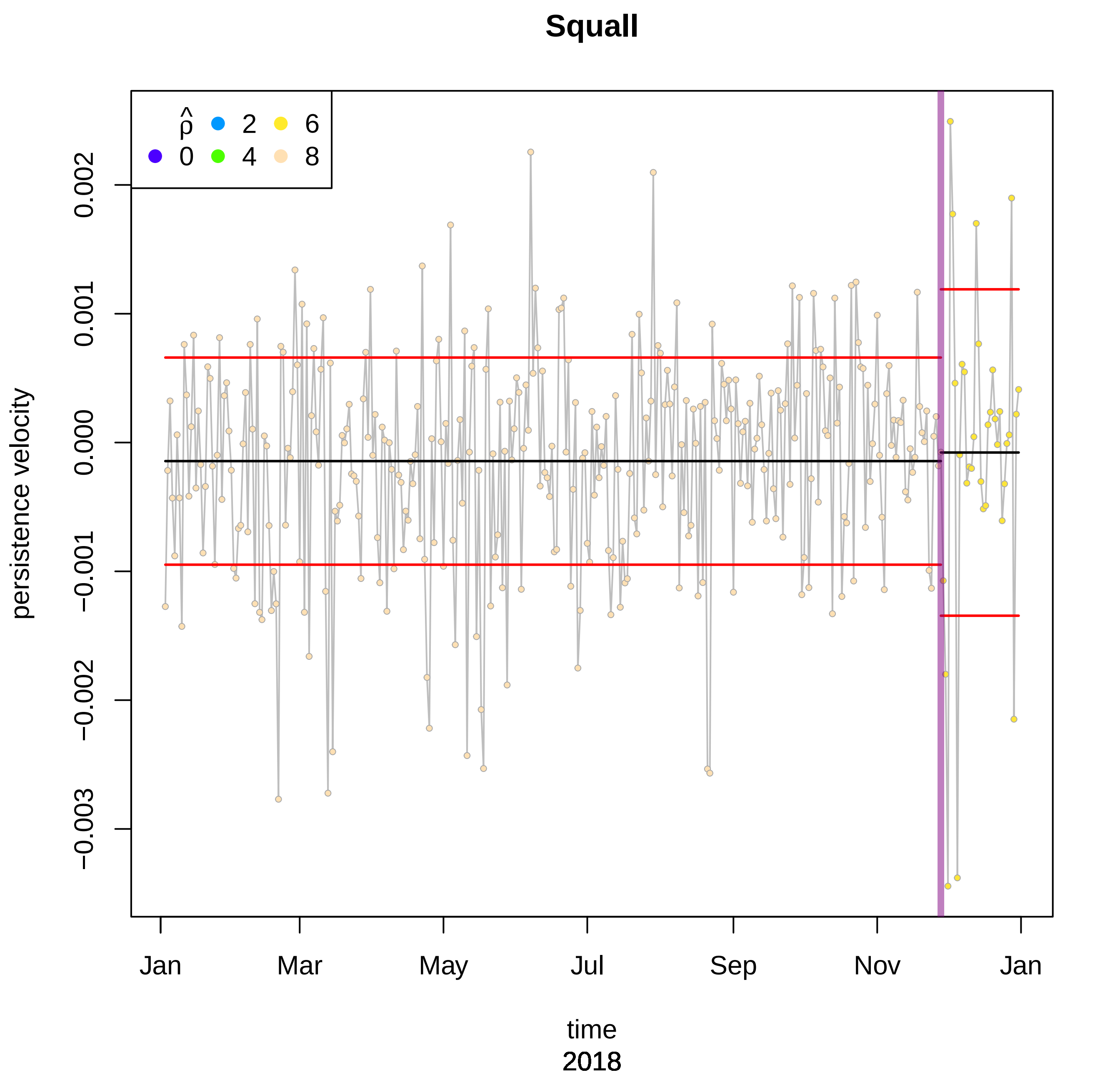

Squall
ρ̂
0
2
4
6
8
persistence velocity
0.002
0.001
0.000
−0.001
−0.002
−0.003
Jan
Mar
May
Jul
Sep
Nov
Jan
time
2018

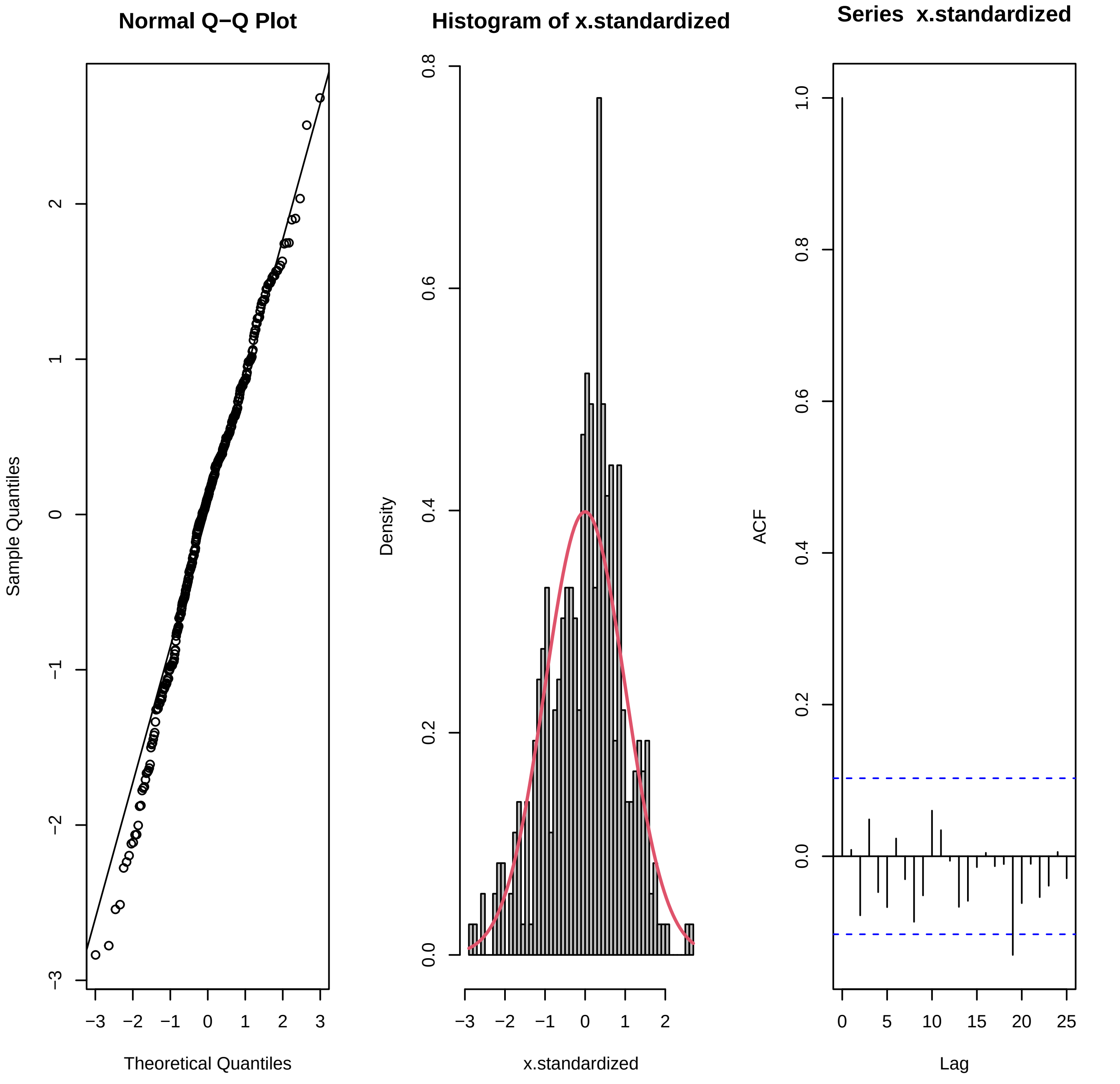

Normal Q−Q Plot
Sample Quantiles
Theoretical Quantiles
−3
−2
−1
0
1
2
3
Histogram of x.standardized
Density
x.standardized
0.0
0.2
0.4
0.6
0.8
Series x.standardized
ACF
Lag
1.0
5
10
15
20
25

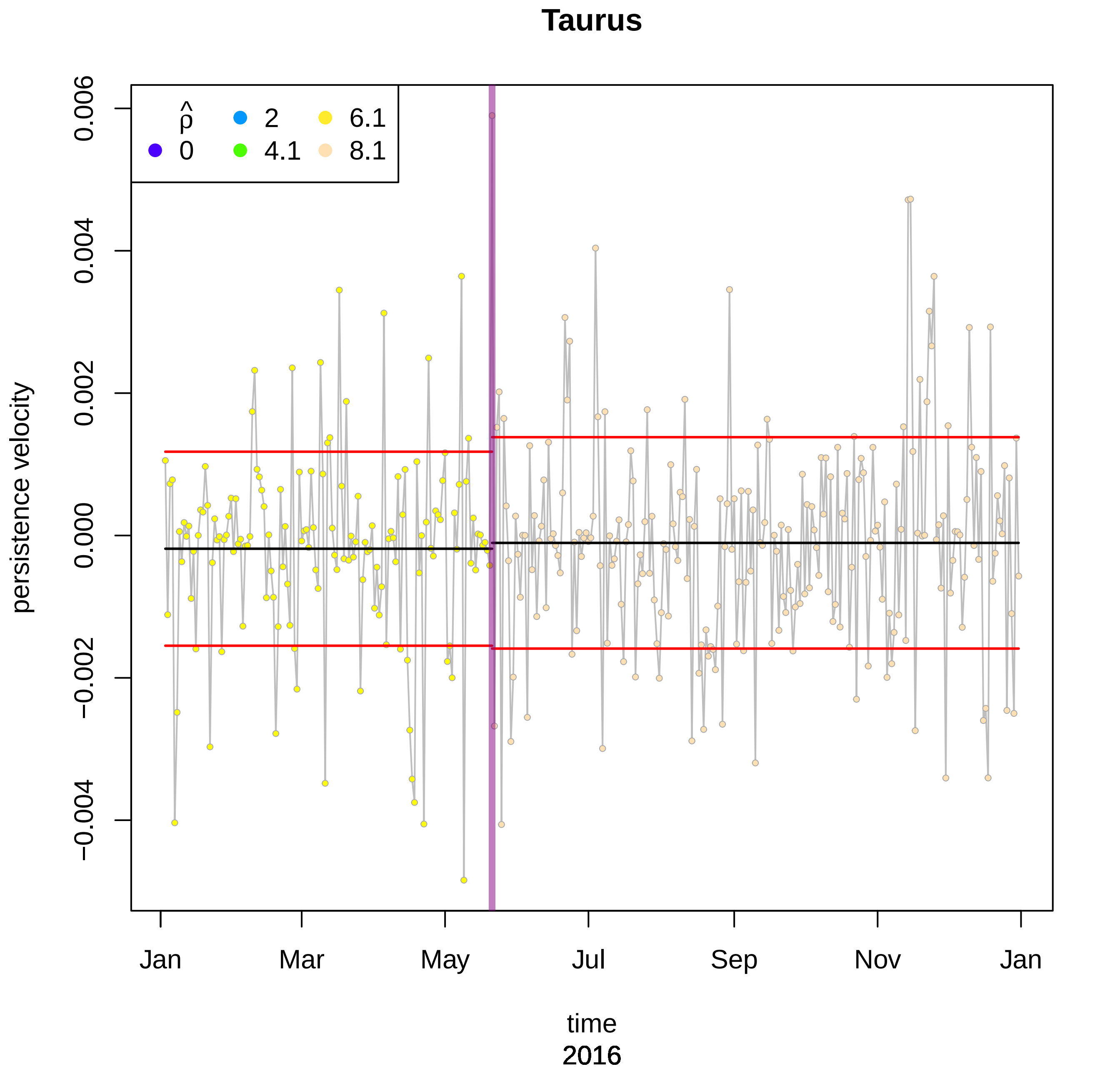

Taurus
ρ̂
0
2
4.1
6.1
8.1
0.006
0.004
0.002
0.000
−0.002
−0.004
persistence velocity
Jan
Mar
May
Jul
Sep
Nov
Jan
time
2016

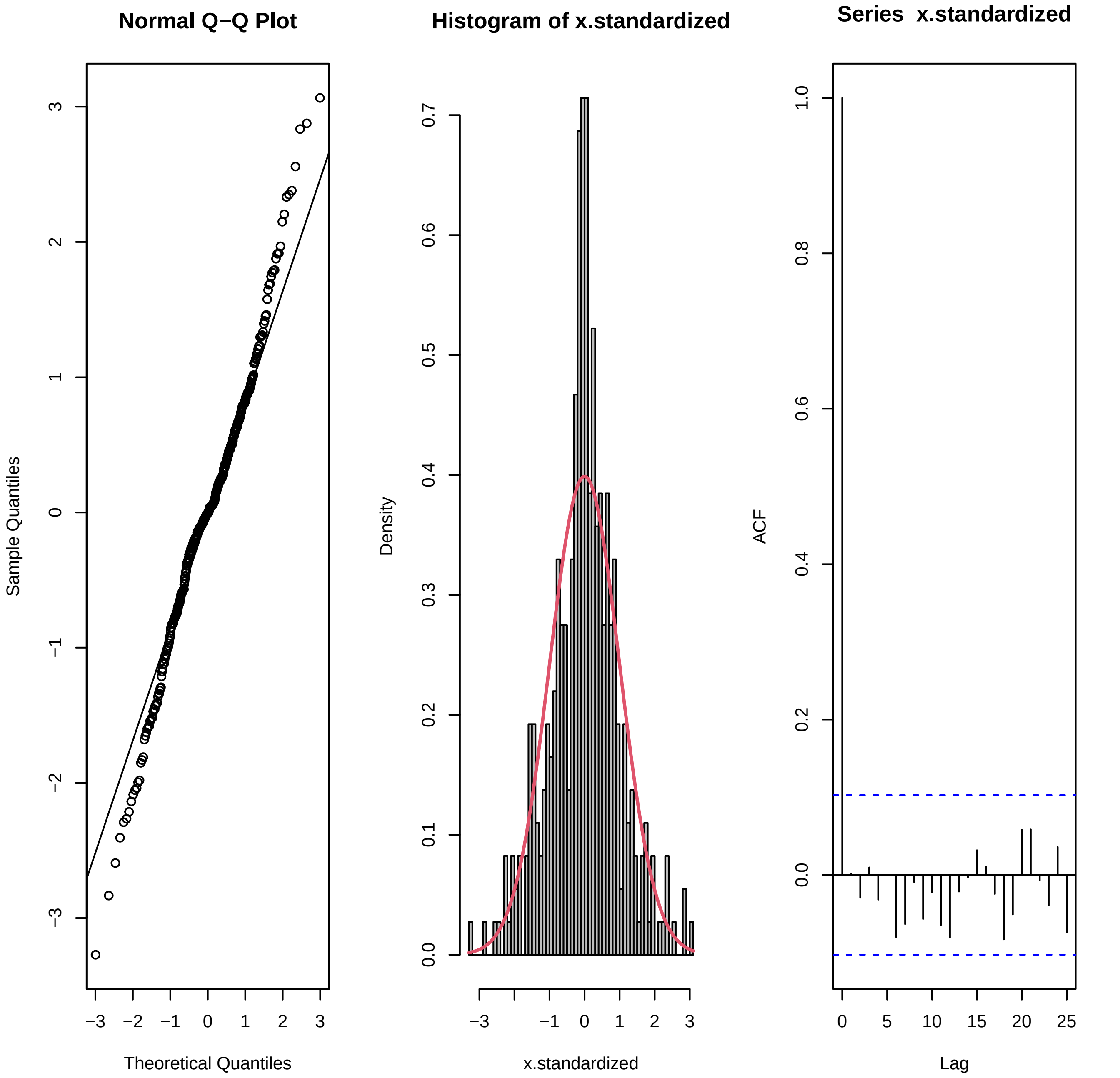

Normal Q−Q Plot
Sample Quantiles
Theoretical Quantiles
−3
−2
−1
0
1
2
3
Histogram of x.standardized
Density
x.standardized
0.0
0.1
0.2
0.3
0.4
0.5
0.6
0.7
Series x.standardized
ACF
Lag
0
5
10
15
20
25
0.0
0.2
0.4
0.6
0.8
1.0

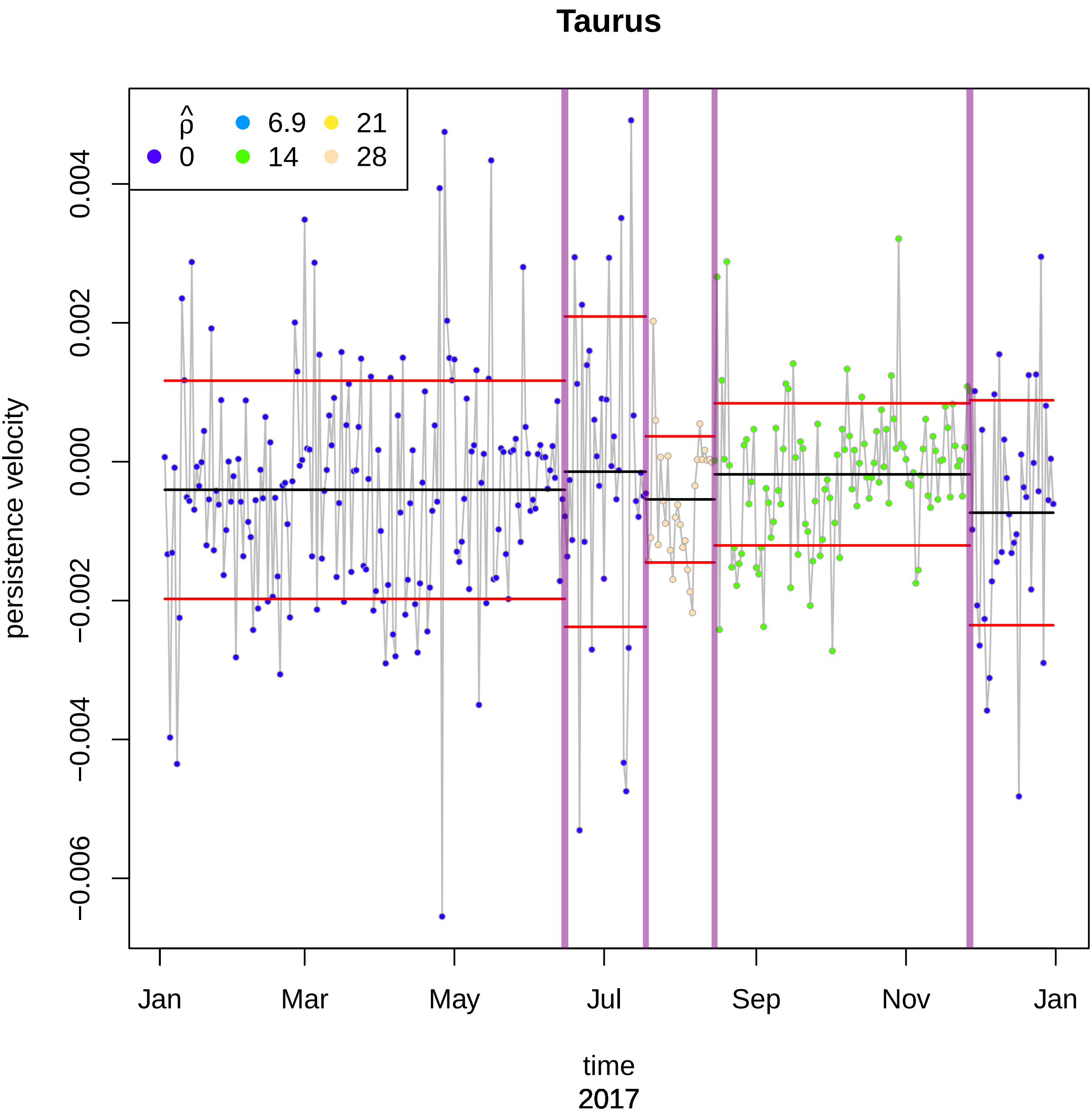
Taurus
ρ̂
0
6.9
14
21
28
0.004
0.002
0.000
−0.002
−0.004
−0.006
persistence velocity
Jan
Mar
May
Jul
Sep
Nov
Jan
time
2017

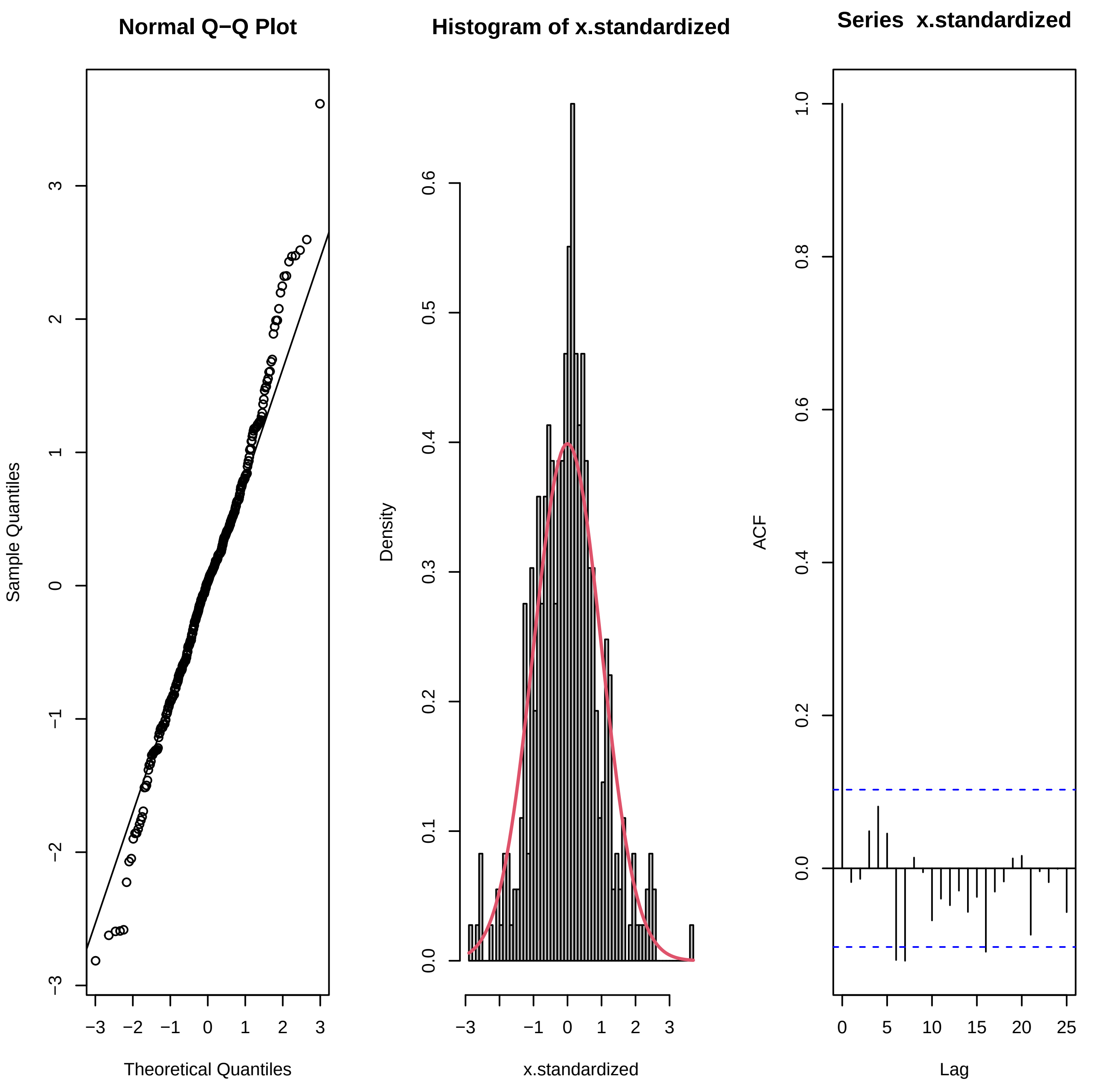

Normal Q−Q Plot
Sample Quantiles
Theoretical Quantiles
Histogram of x.standardized
Density
x.standardized
Series x.standardized
ACF
Lag

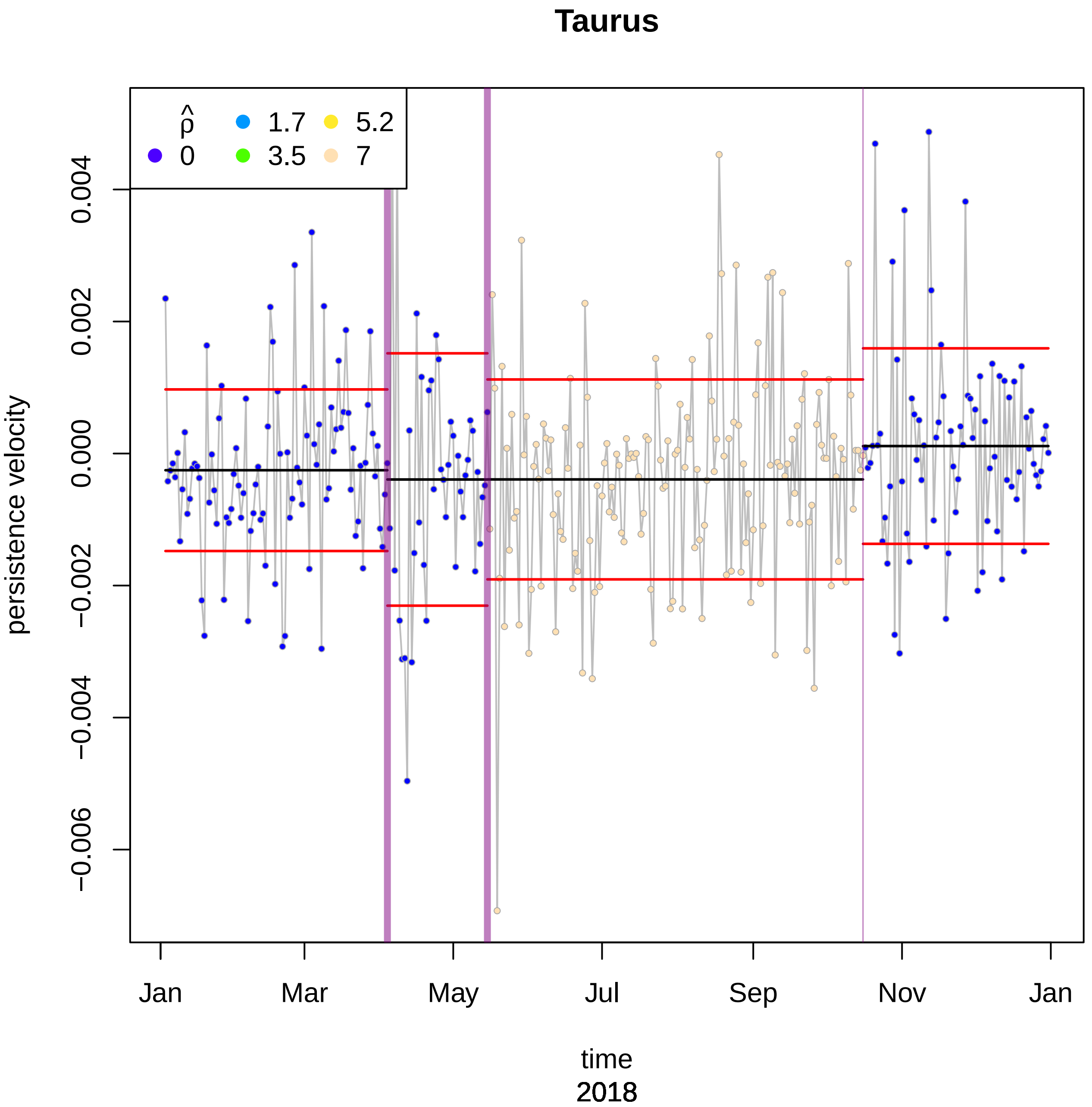

Taurus
ρ̂
0
1.7
3.5
5.2
7
persistence velocity
0.004
0.002
0.000
−0.002
−0.004
−0.006
Jan
Mar
May
Jul
Sep
Nov
Jan
time
2018

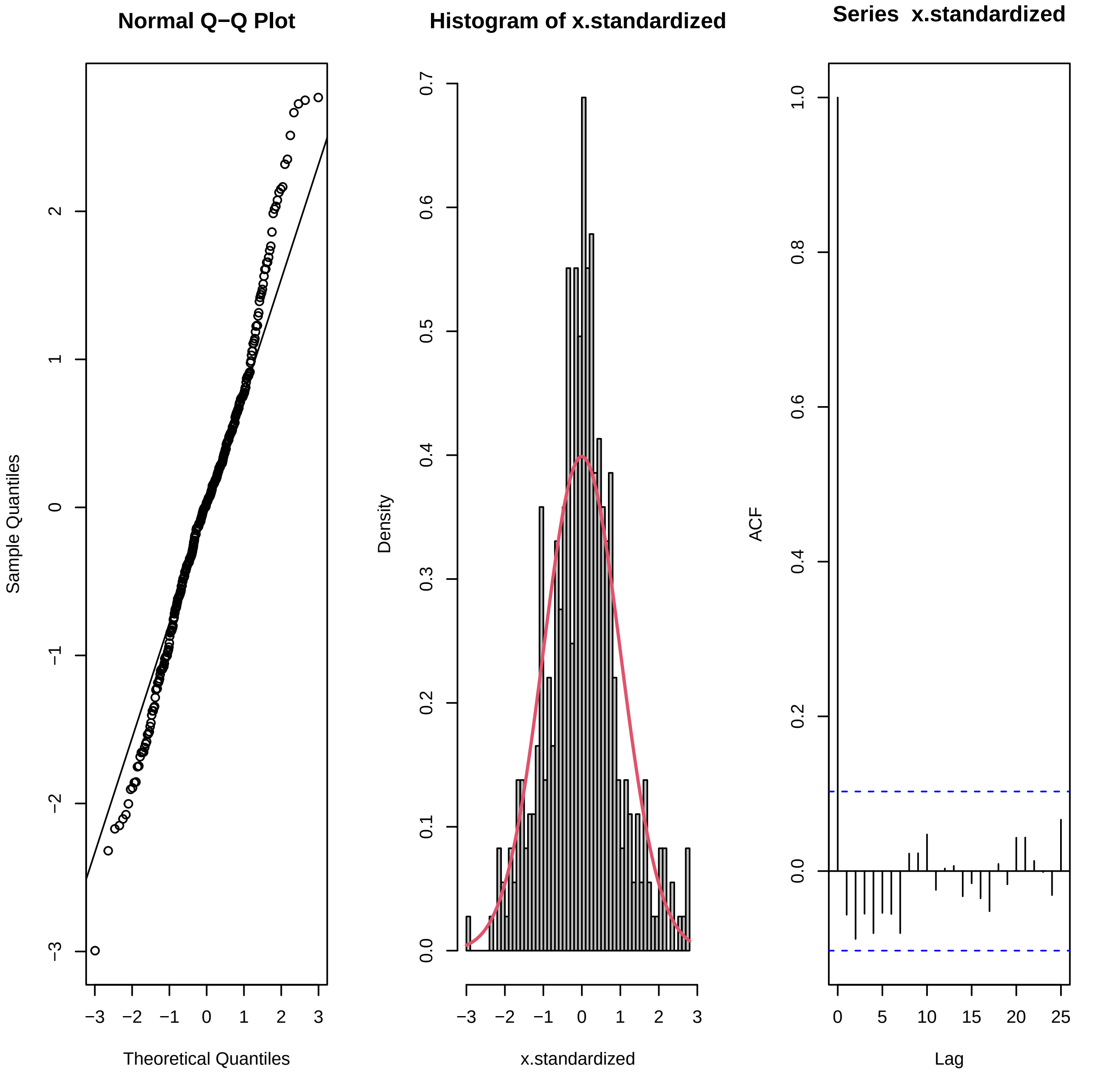
Normal Q−Q Plot
Sample Quantiles
Theoretical Quantiles
Histogram of x.standardized
Density
x.standardized
Series x.standardized
ACF
Lag

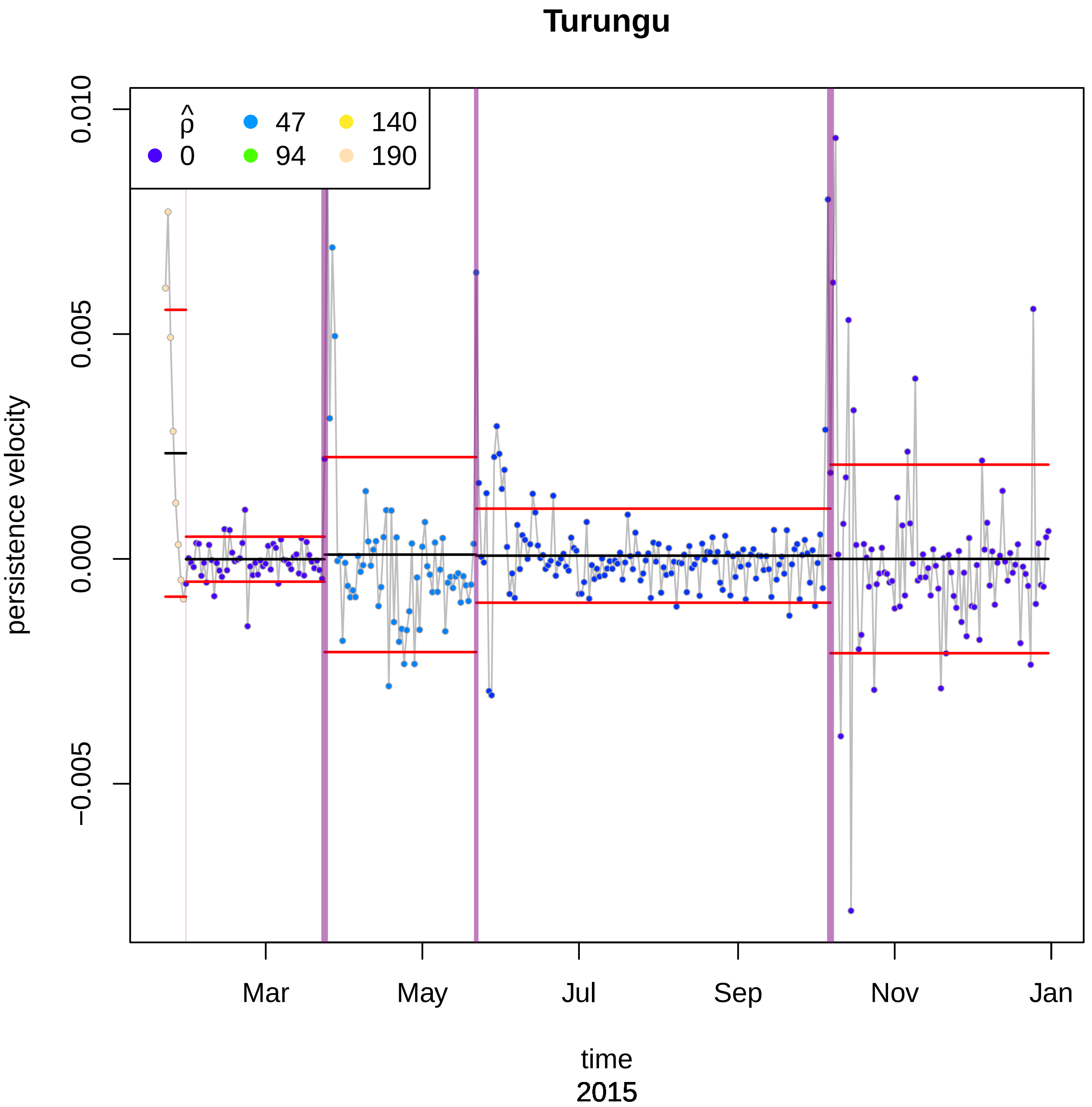

Turungu
ρ̂
0
47
94
140
190
0.010
0.005
0.000
−0.005
persistence velocity
Mar
May
Jul
Sep
Nov
Jan
time
2015

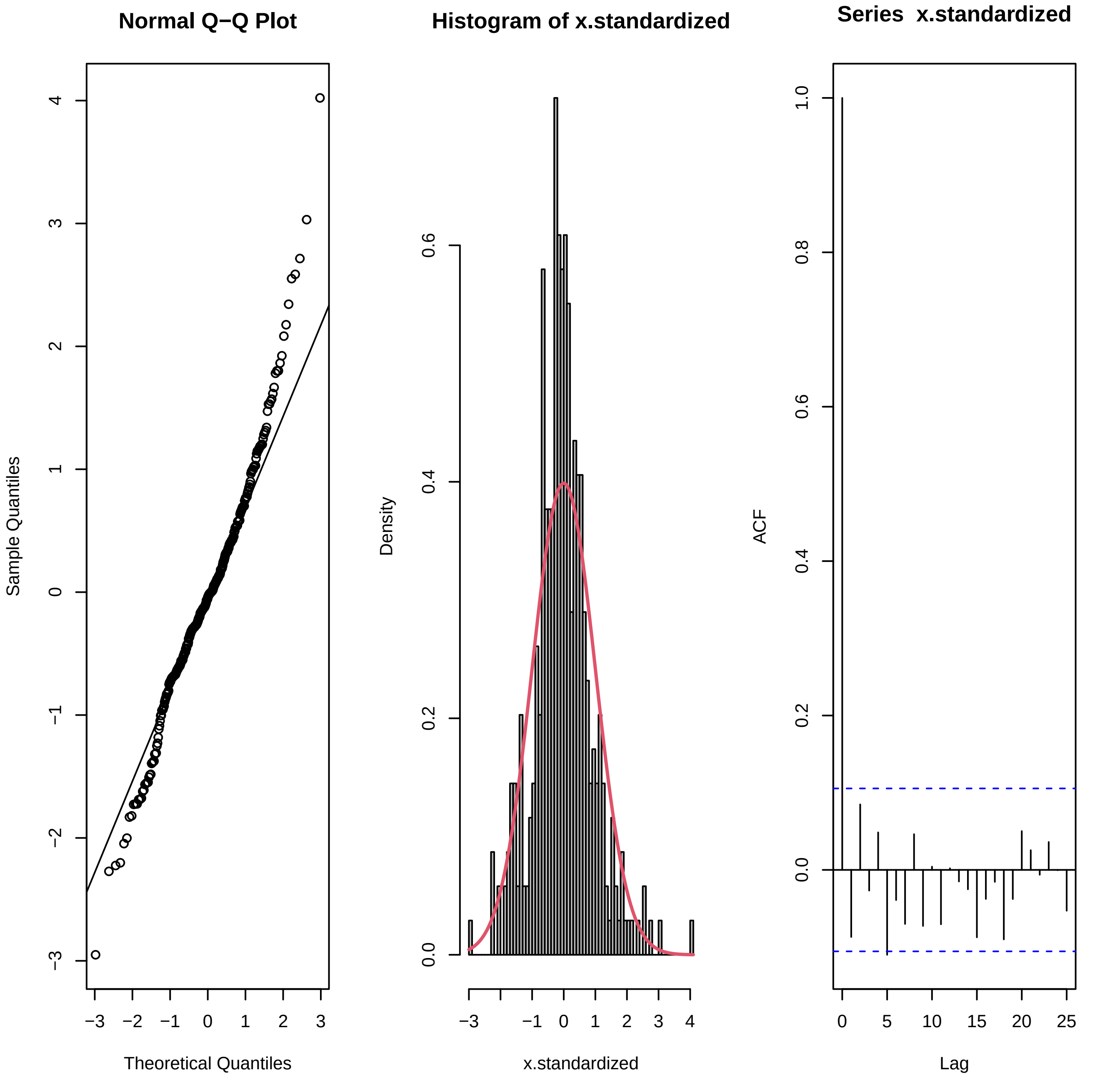

Normal Q−Q Plot
Sample Quantiles
Theoretical Quantiles
Histogram of x.standardized
Density
x.standardized
Series x.standardized
ACF
Lag

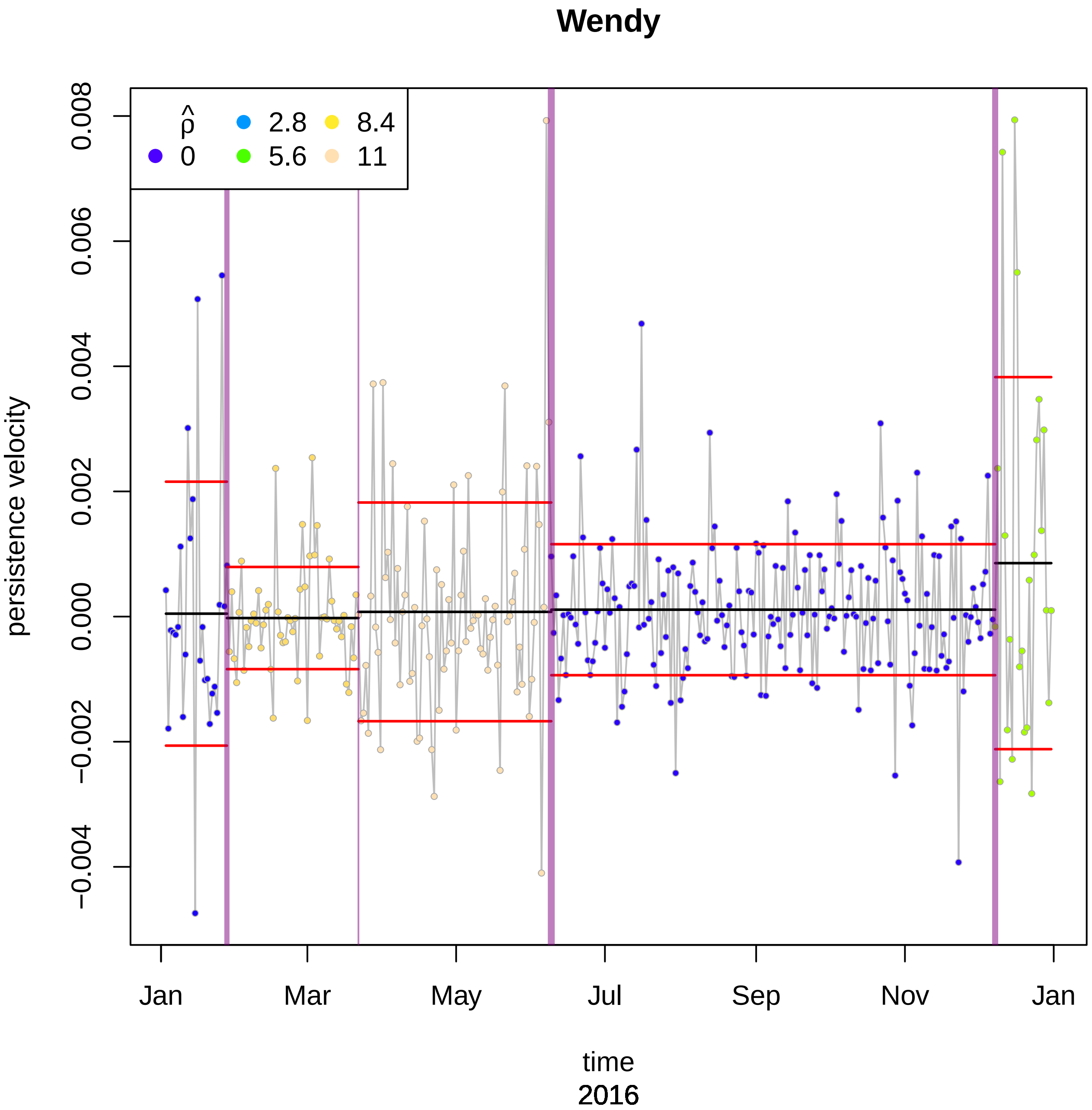

Wendy
ρ̂
0
2.8
5.6
8.4
11
0.008
0.006
0.004
0.002
0.000
−0.002
−0.004
persistence velocity
Jan
Mar
May
Jul
Sep
Nov
Jan
time
2016

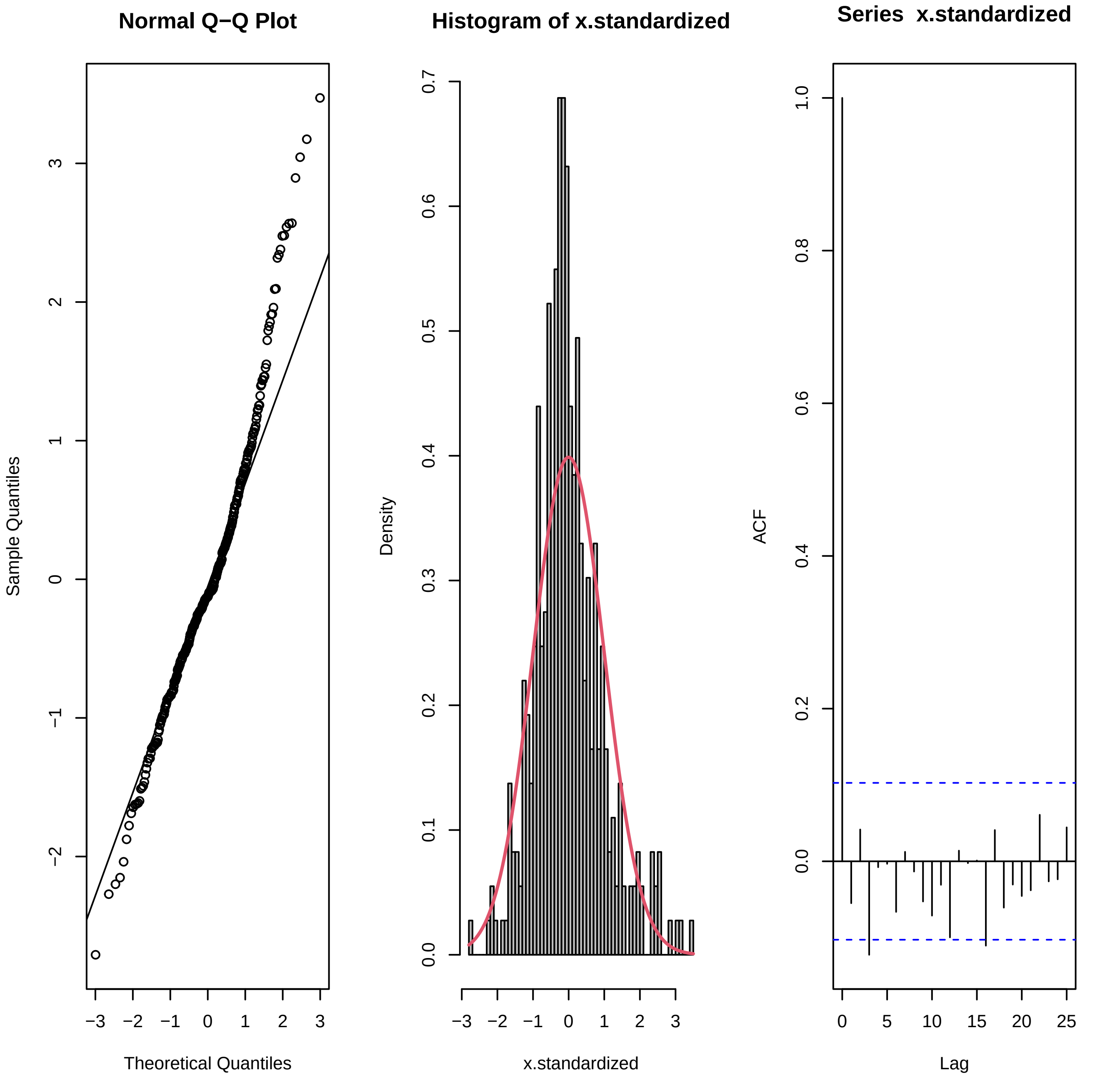

Normal Q−Q Plot
Sample Quantiles
Theoretical Quantiles
−3
−2
−1
0
1
2
3
Histogram of x.standardized
Density
x.standardized
0.0
0.1
0.2
0.3
0.4
0.5
0.6
0.7
Series x.standardized
ACF
Lag
0.0
0.2
0.4
0.6
0.8
1.0
0
5
10
15
20
25

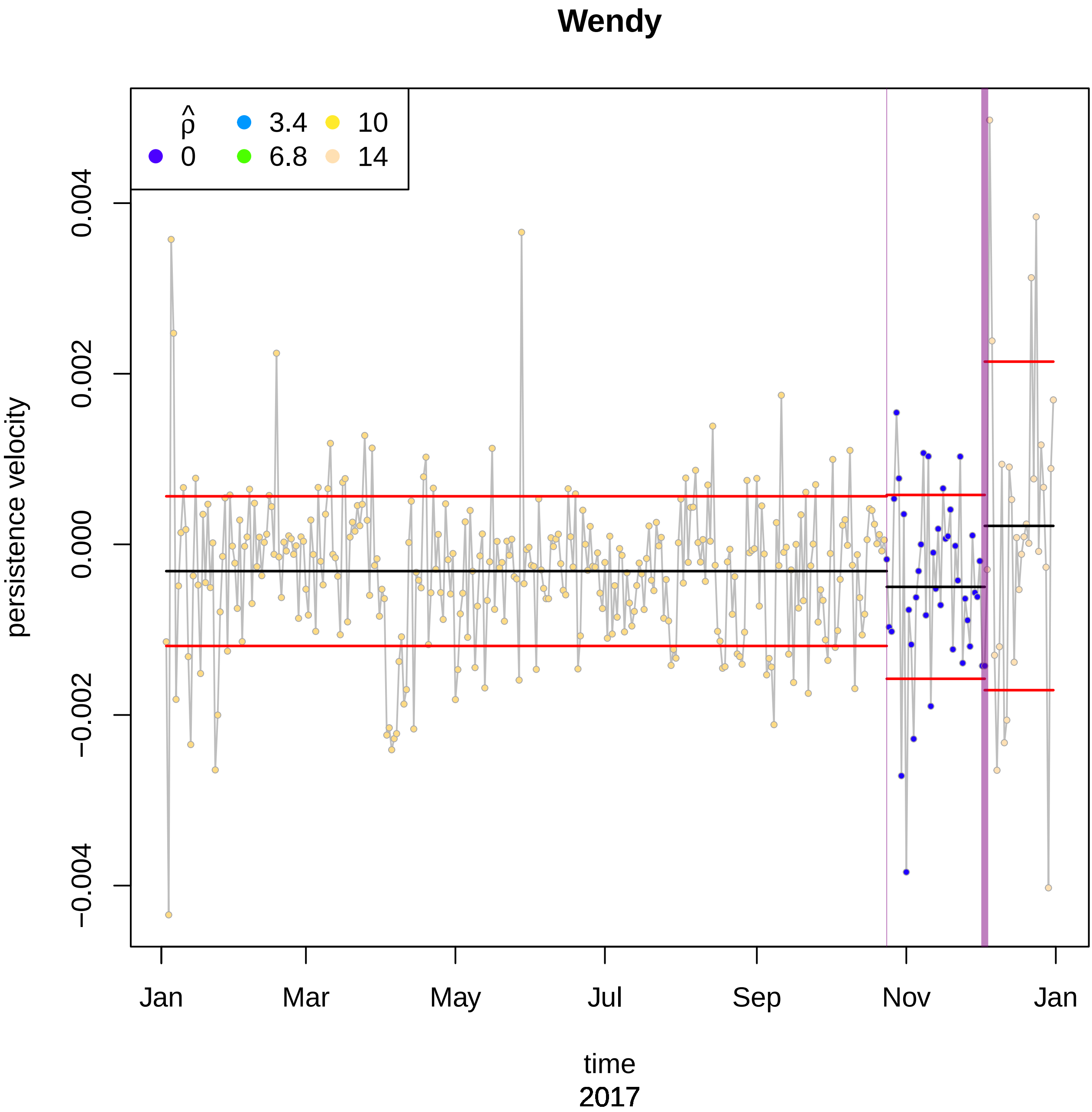

Wendy
ρ̂
0
3.4
6.8
10
14
persistence velocity
0.004
0.002
0.000
−0.002
−0.004
Jan
Mar
May
Jul
Sep
Nov
Jan
time
2017

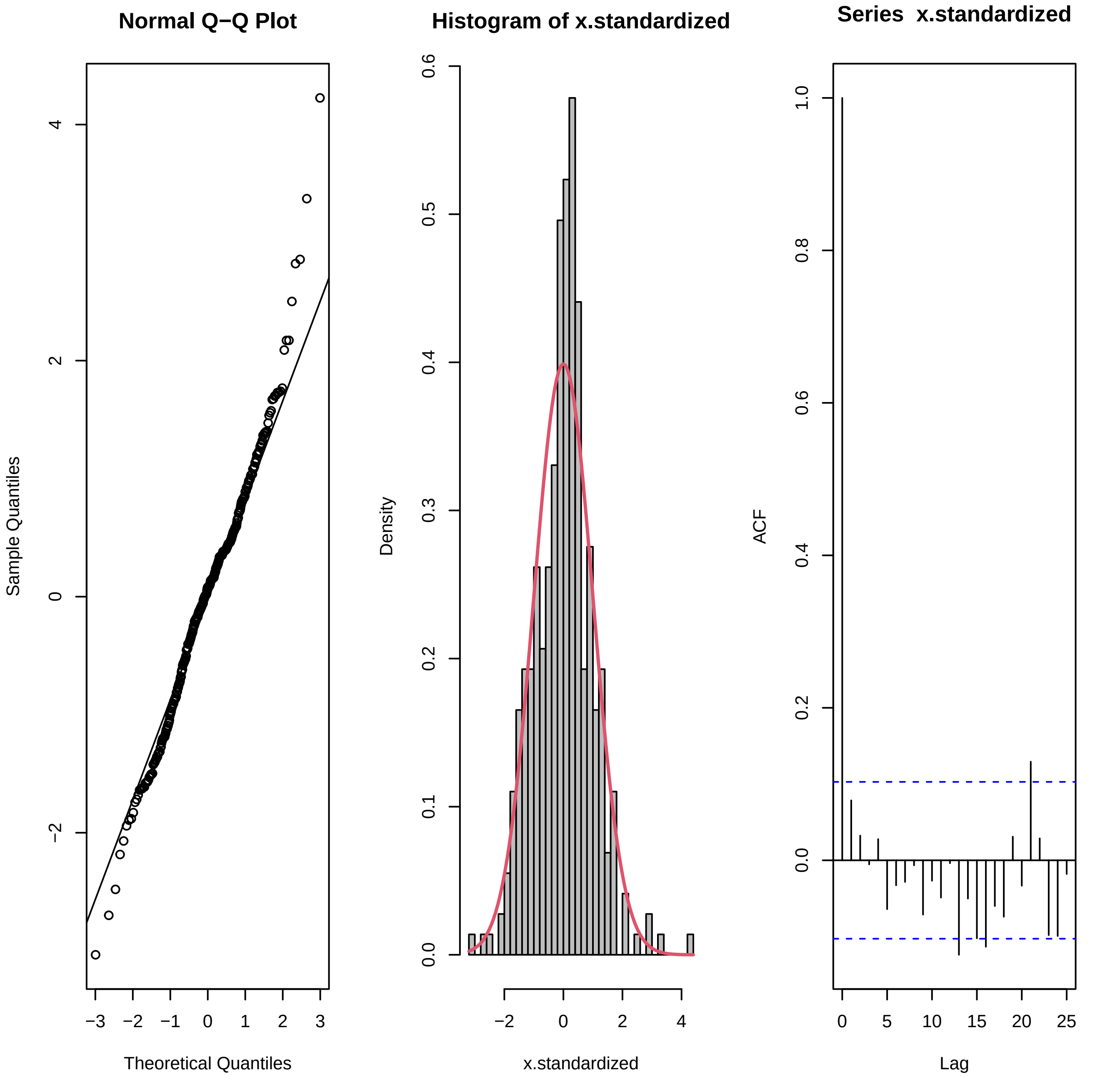

Normal Q−Q Plot
Sample Quantiles
Theoretical Quantiles
−3 −2 −1 0 1 2 3
−2 0 2 4
Histogram of x.standardized
Density
x.standardized
0.0 0.1 0.2 0.3 0.4 0.5 0.6
−2 0 2 4
Series x.standardized
ACF
Lag
0.0 0.2 0.4 0.6 0.8 1.0
0 5 10 15 20 25

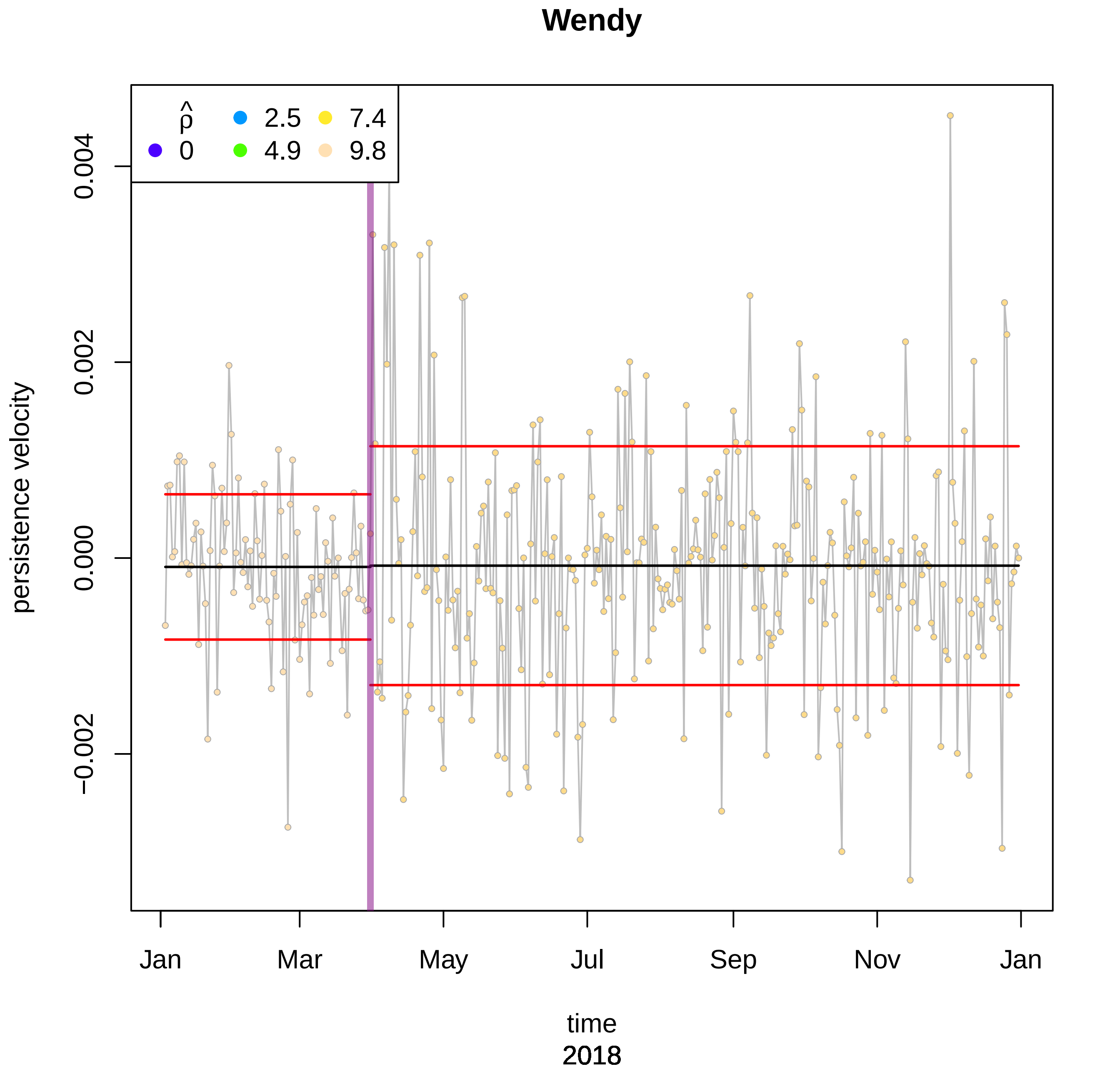

Wendy
ρ̂
0
2.5
4.9
7.4
9.8
0.004
0.002
0.000
−0.002
persistence velocity
Jan
Mar
May
Jul
Sep
Nov
Jan
time
2018

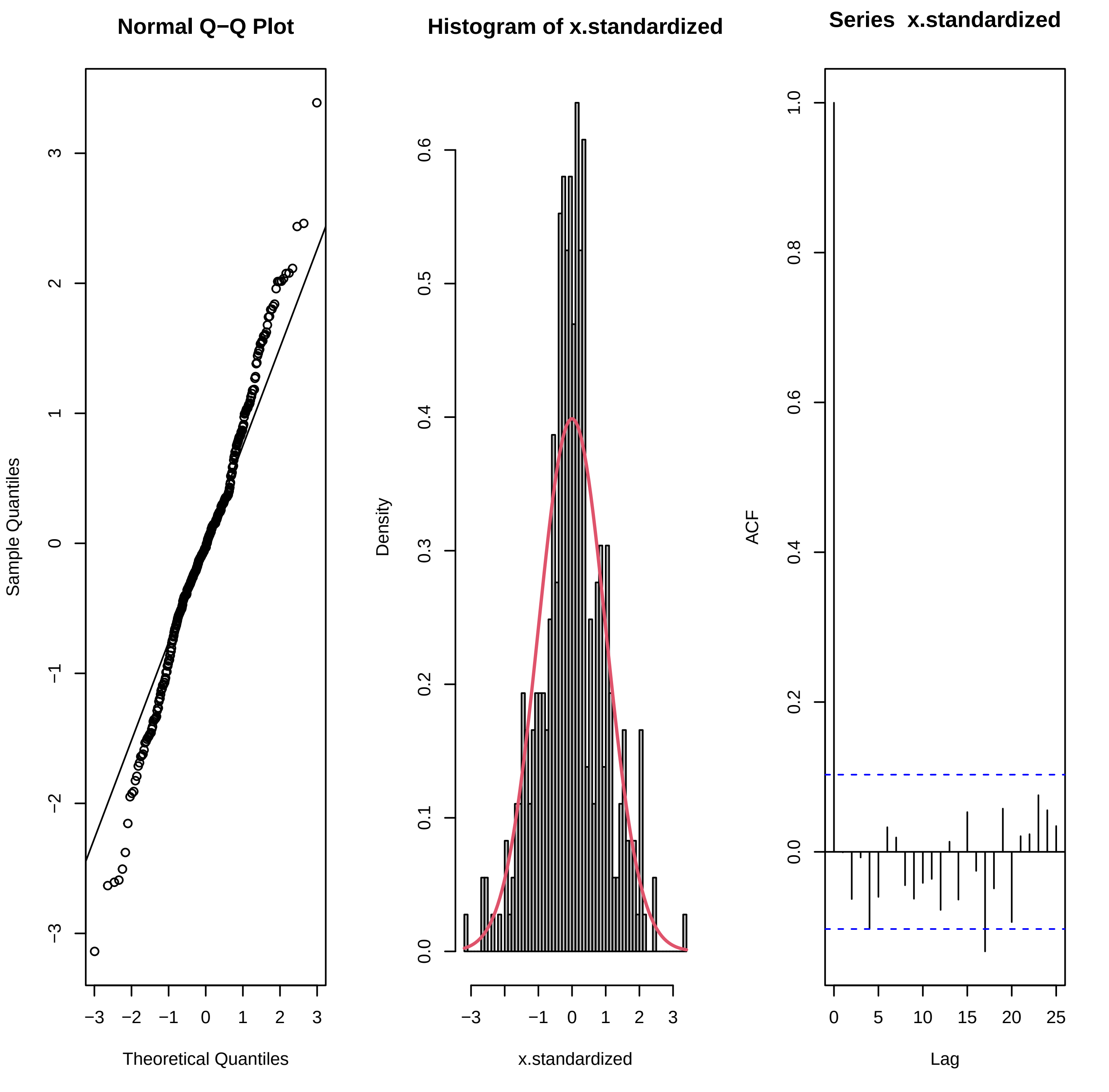

Normal Q−Q Plot
Theoretical Quantiles
Sample Quantiles
Histogram of x.standardized
x.standardized
Density
Series x.standardized
Lag
ACF

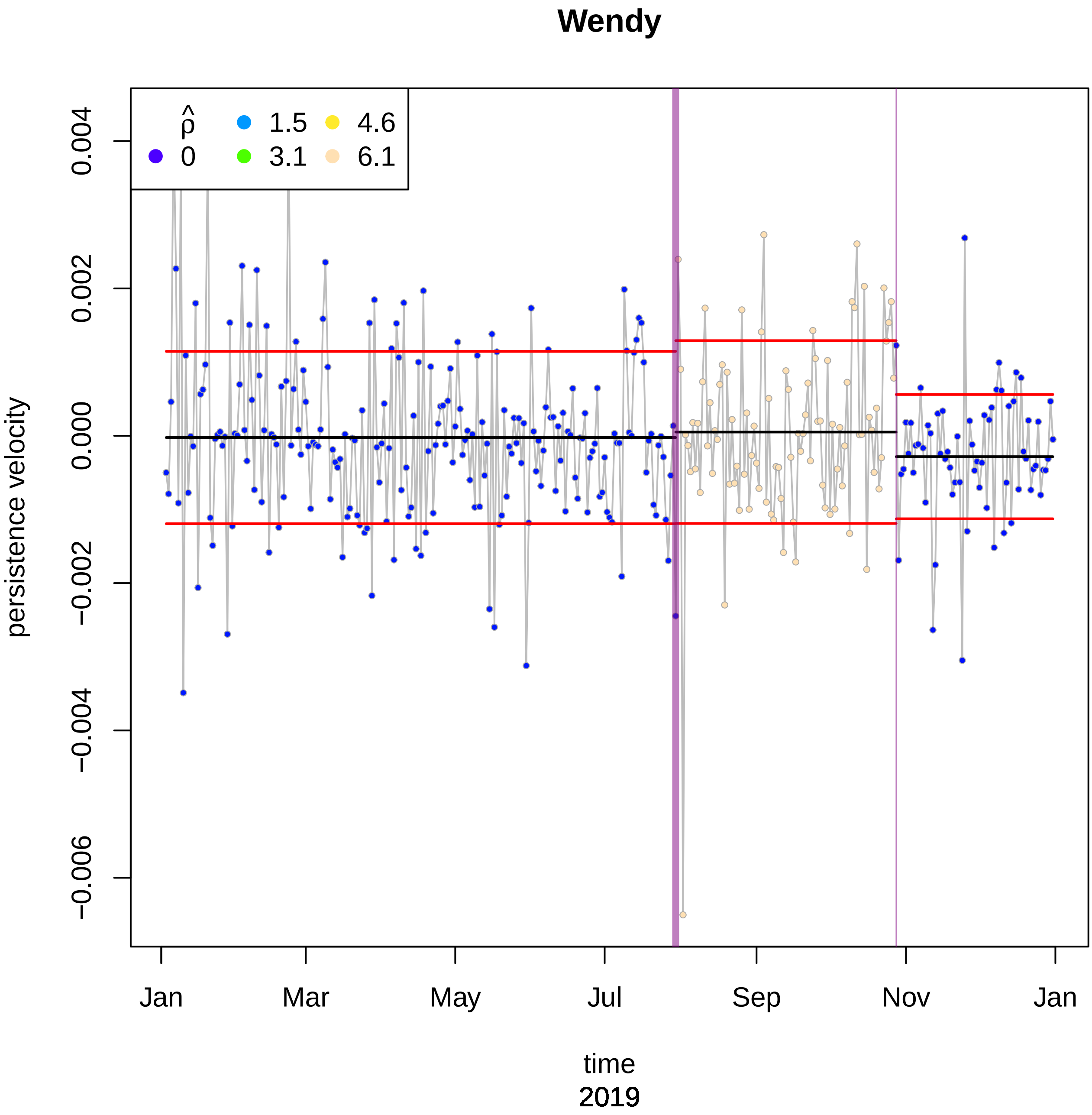

Wendy
ρ̂
0
1.5
3.1
4.6
6.1
0.004
0.002
0.000
−0.002
−0.004
−0.006
persistence velocity
Jan
Mar
May
Jul
Sep
Nov
Jan
time
2019

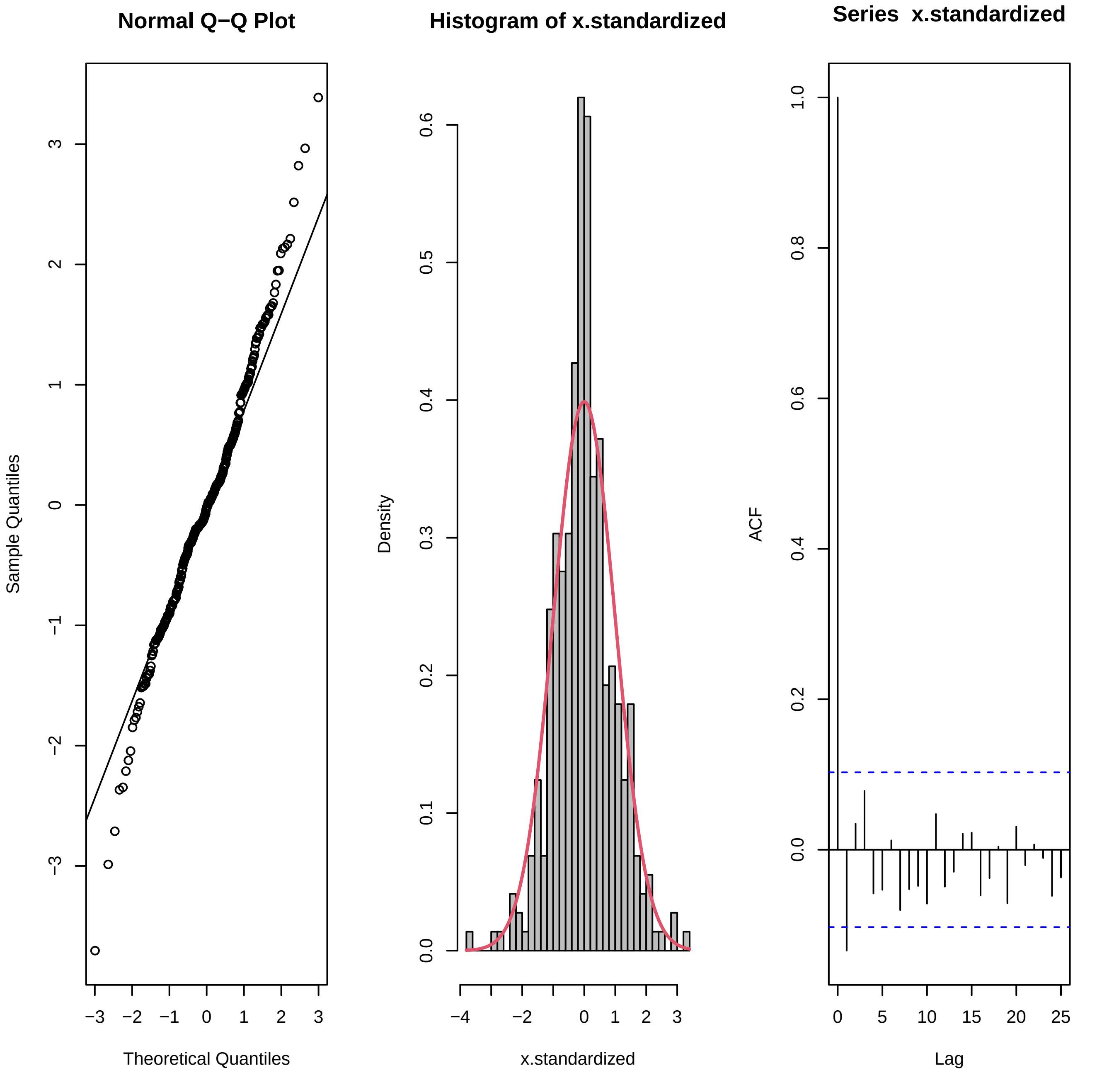

Normal Q−Q Plot
Sample Quantiles
Theoretical Quantiles
−3 −2 −1 0 1 2 3
Histogram of x.standardized
Density
x.standardized
0.0 0.1 0.2 0.3 0.4 0.5 0.6
−4 −2 0 1 2 3
Series x.standardized
ACF
Lag
0.0 0.2 0.4 0.6 0.8 1.0
0 5 10 15 20 25

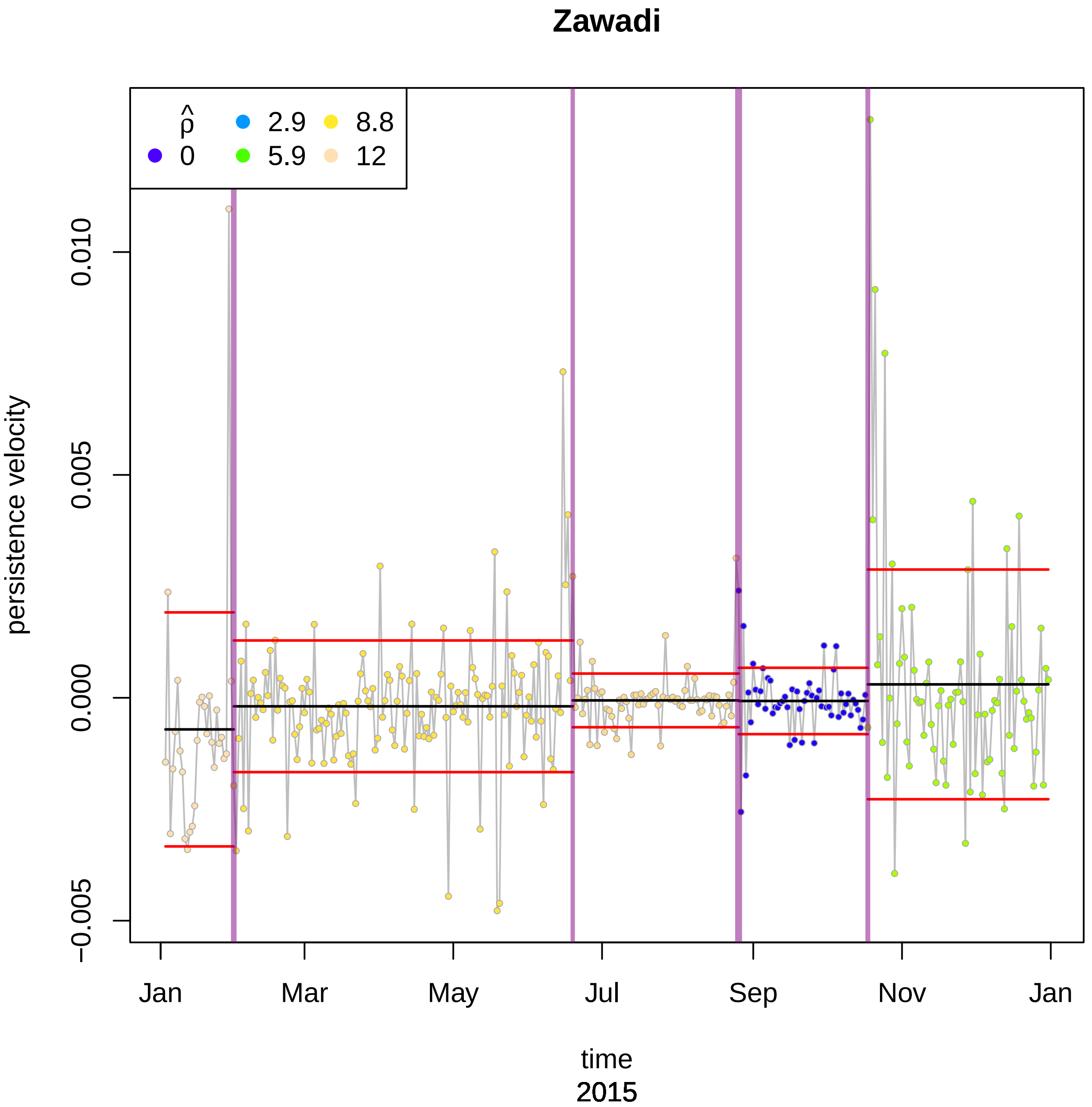

Zawadi
ρ̂
0
2.9
5.9
8.8
12
persistence velocity
0.010
0.005
0.000
−0.005
Jan
Mar
May
Jul
Sep
Nov
Jan
time
2015

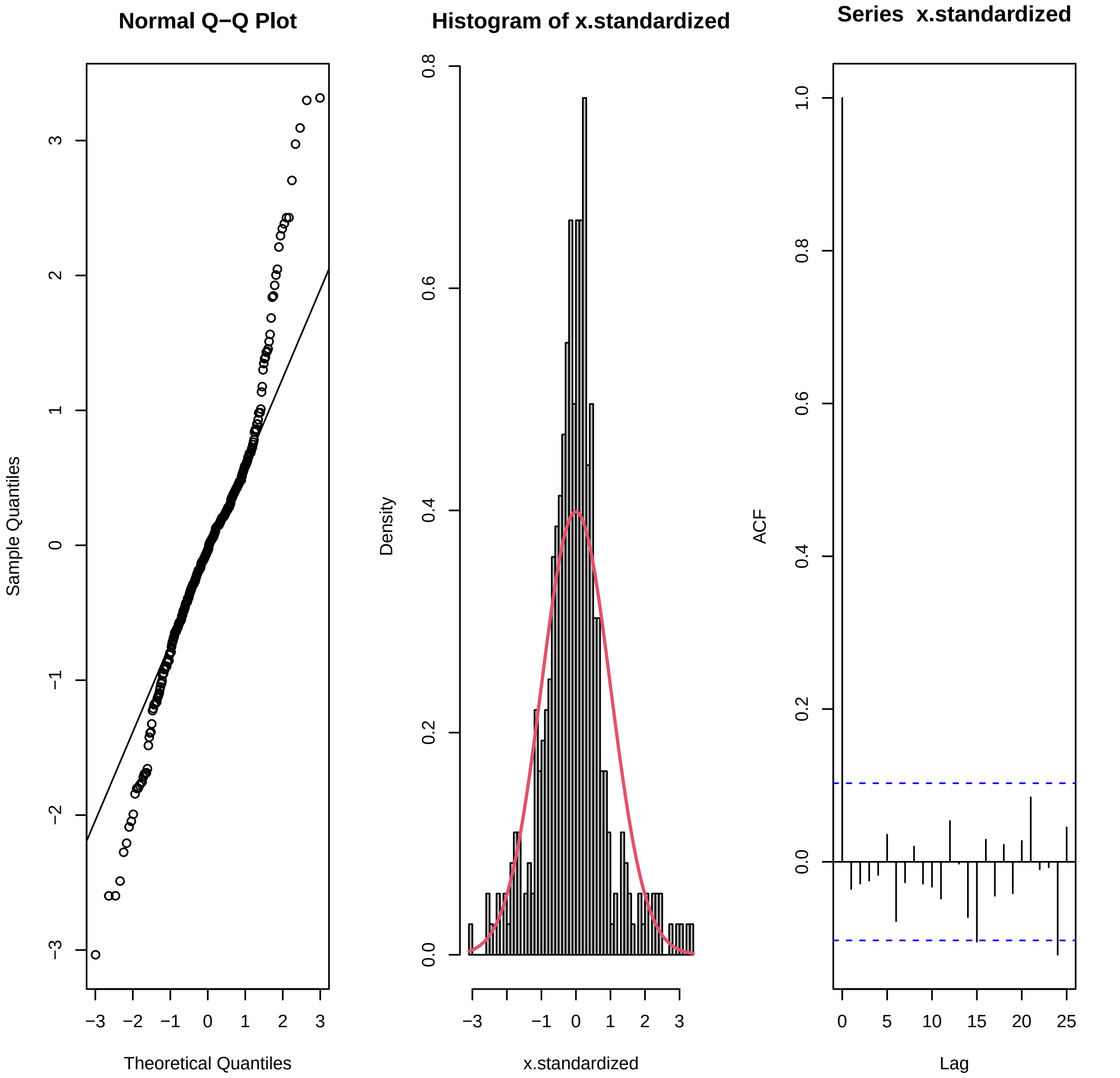
Normal Q−Q Plot
Sample Quantiles
Theoretical Quantiles
−3
−2
−1
0
1
2
3
Histogram of x.standardized
Density
x.standardized
0.0
0.2
0.4
0.6
0.8
Series x.standardized
ACF
Lag
0
5
10
15
20
25
1.0

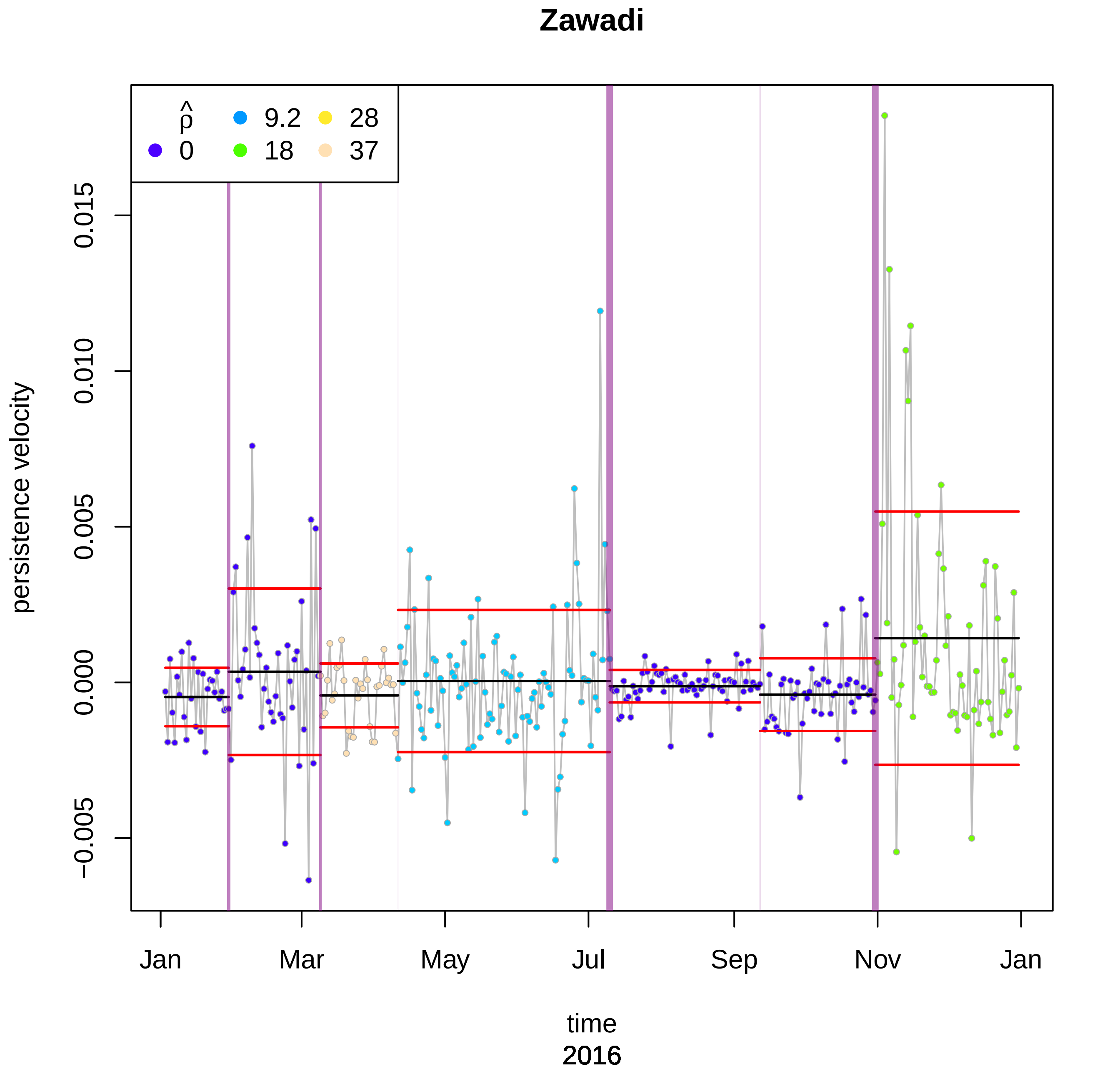

Zawadi
ρ̂
0
9.2
18
28
37
persistence velocity
0.015
0.010
0.005
0.000
−0.005
Jan
Mar
May
Jul
Sep
Nov
Jan
time
2016

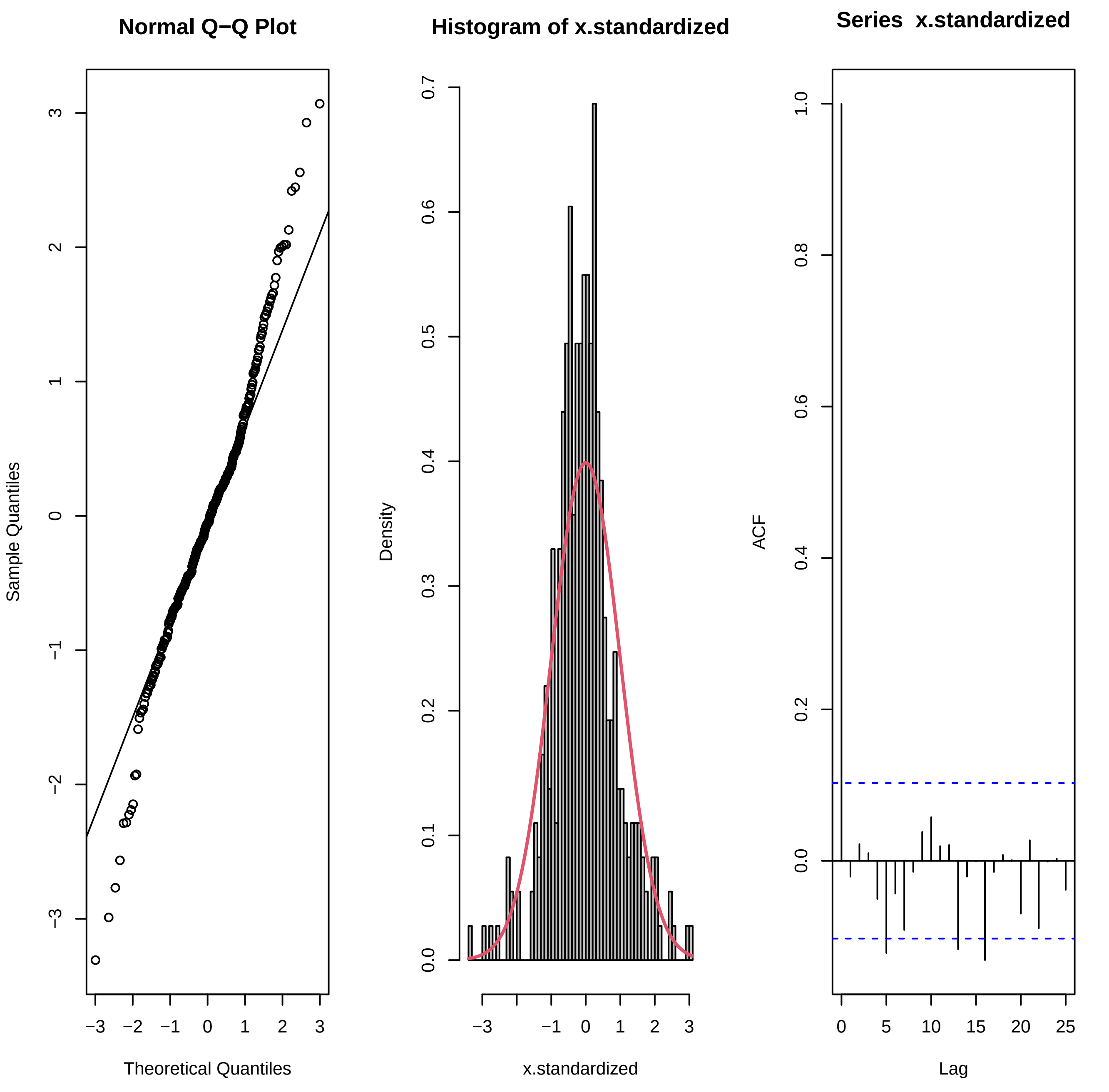
Normal Q−Q Plot
Sample Quantiles
Theoretical Quantiles
−3 −2 −1 0 1 2 3
Histogram of x.standardized
Density
x.standardized
0.0 0.1 0.2 0.3 0.4 0.5 0.6 0.7
Series x.standardized
ACF
Lag
0.0 0.2 0.4 0.6 0.8 1.0
0 5 10 15 20 25

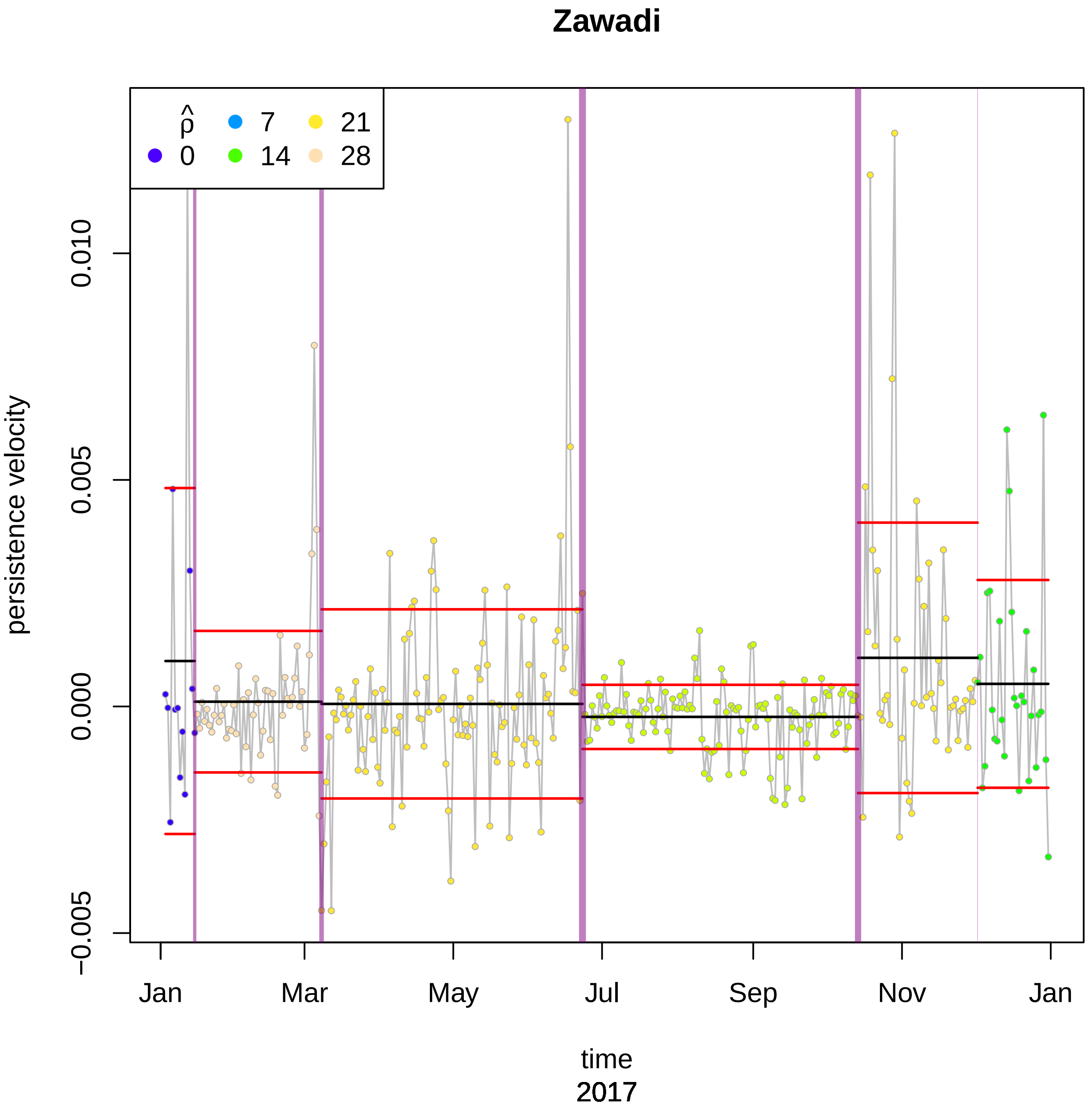

Zawadi
ρ̂
0
7
14
21
28
persistence velocity
0.010
0.005
0.000
−0.005
Jan
Mar
May
Jul
Sep
Nov
Jan
time
2017

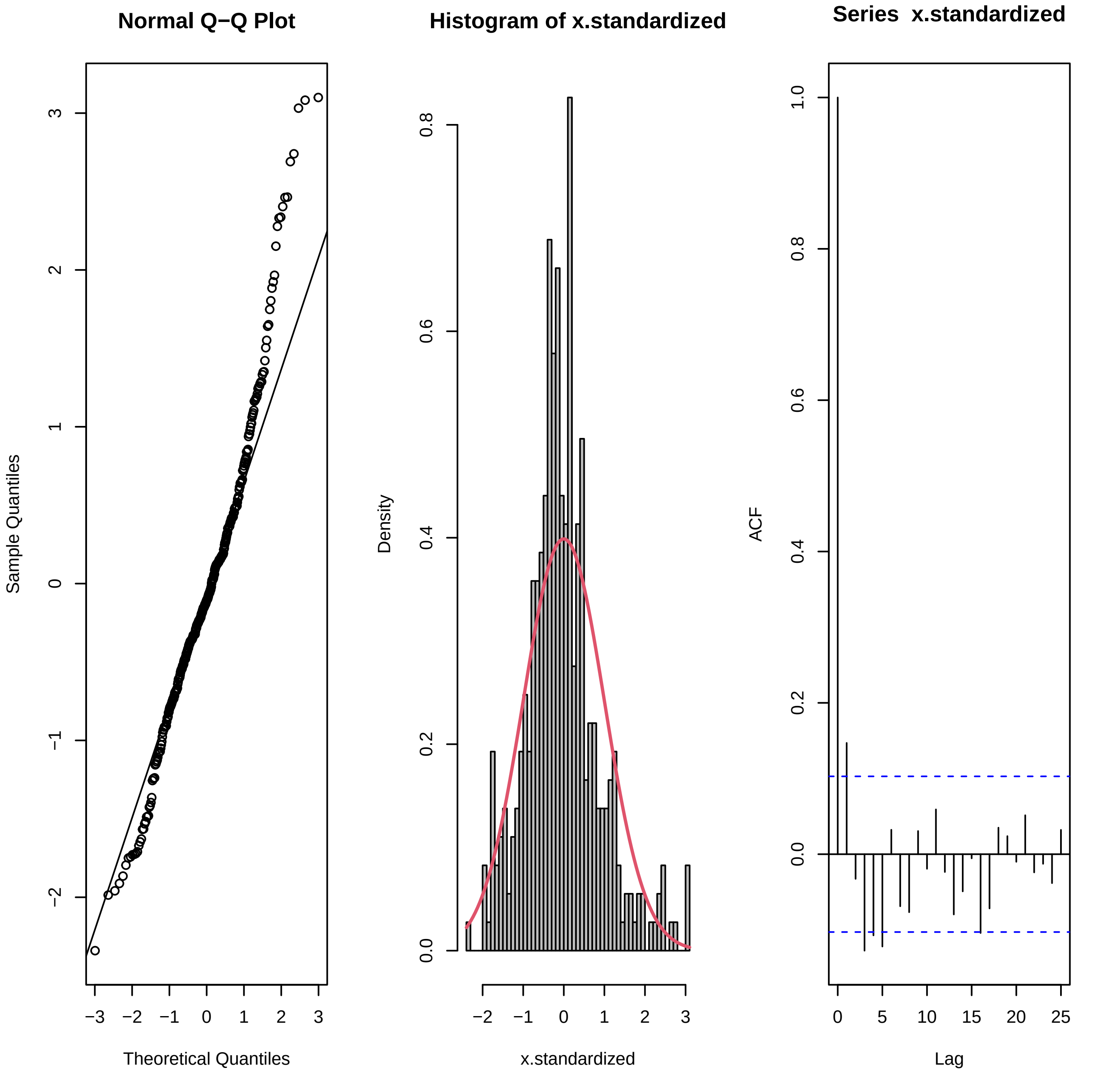

Normal Q−Q Plot
Sample Quantiles
Theoretical Quantiles
Histogram of x.standardized
Density
x.standardized
Series x.standardized
ACF
Lag

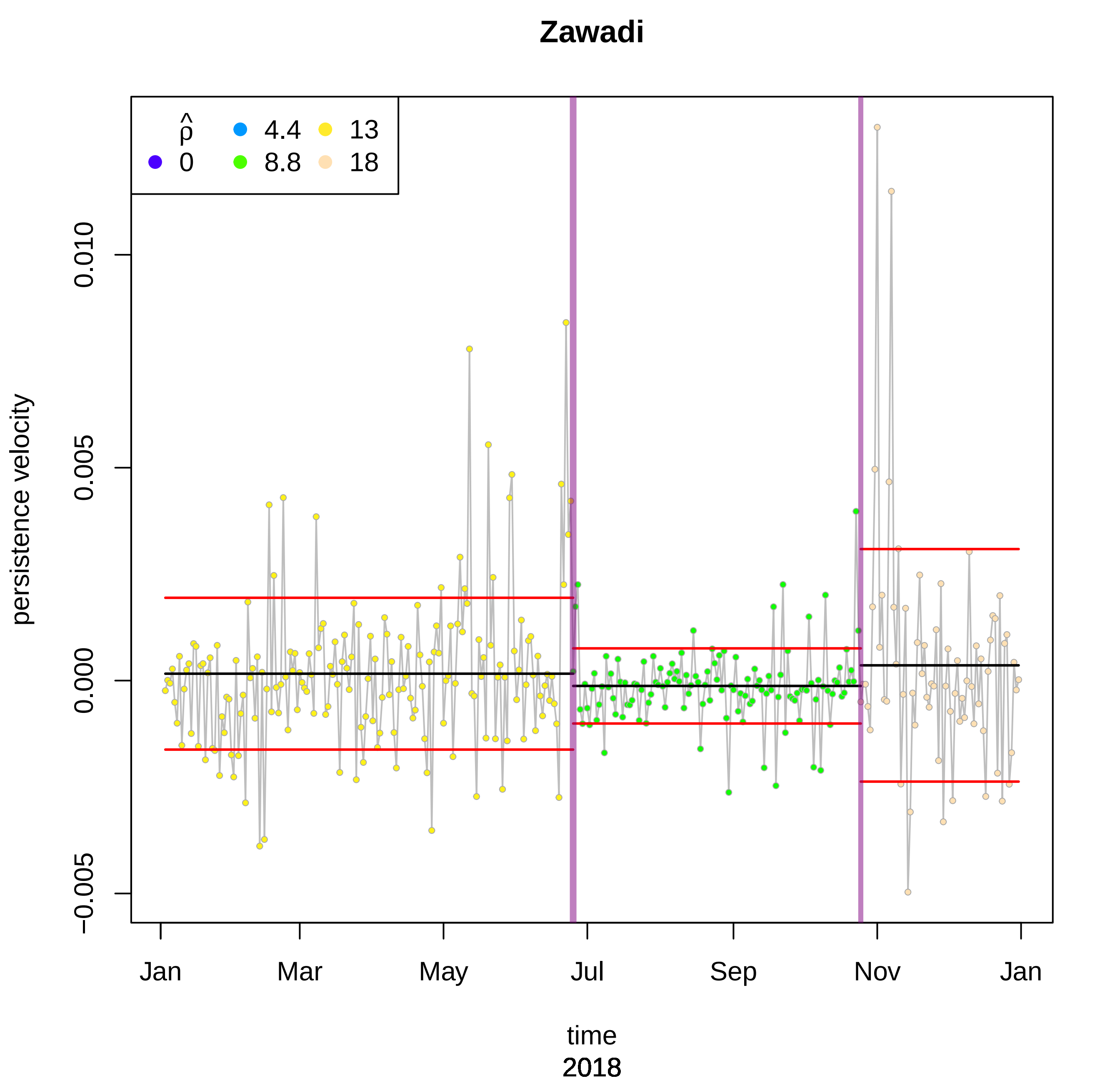

Zawadi
ρ̂
0
4.4
8.8
13
18
persistence velocity
0.010
0.005
0.000
−0.005
Jan
Mar
May
Jul
Sep
Nov
Jan
time
2018

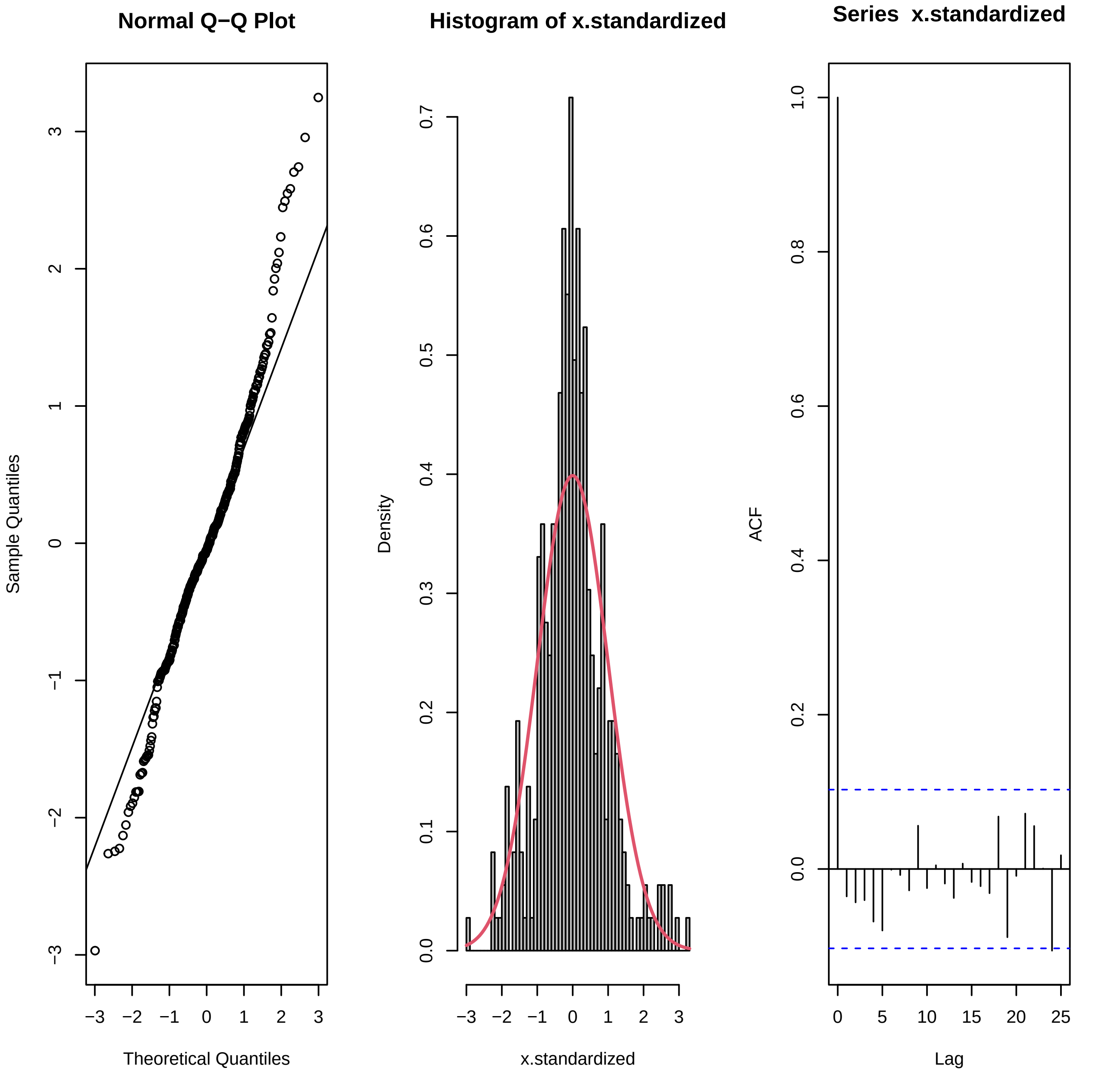

Normal Q−Q Plot
Sample Quantiles
Theoretical Quantiles
Histogram of x.standardized
Density
x.standardized
Series x.standardized
ACF
Lag

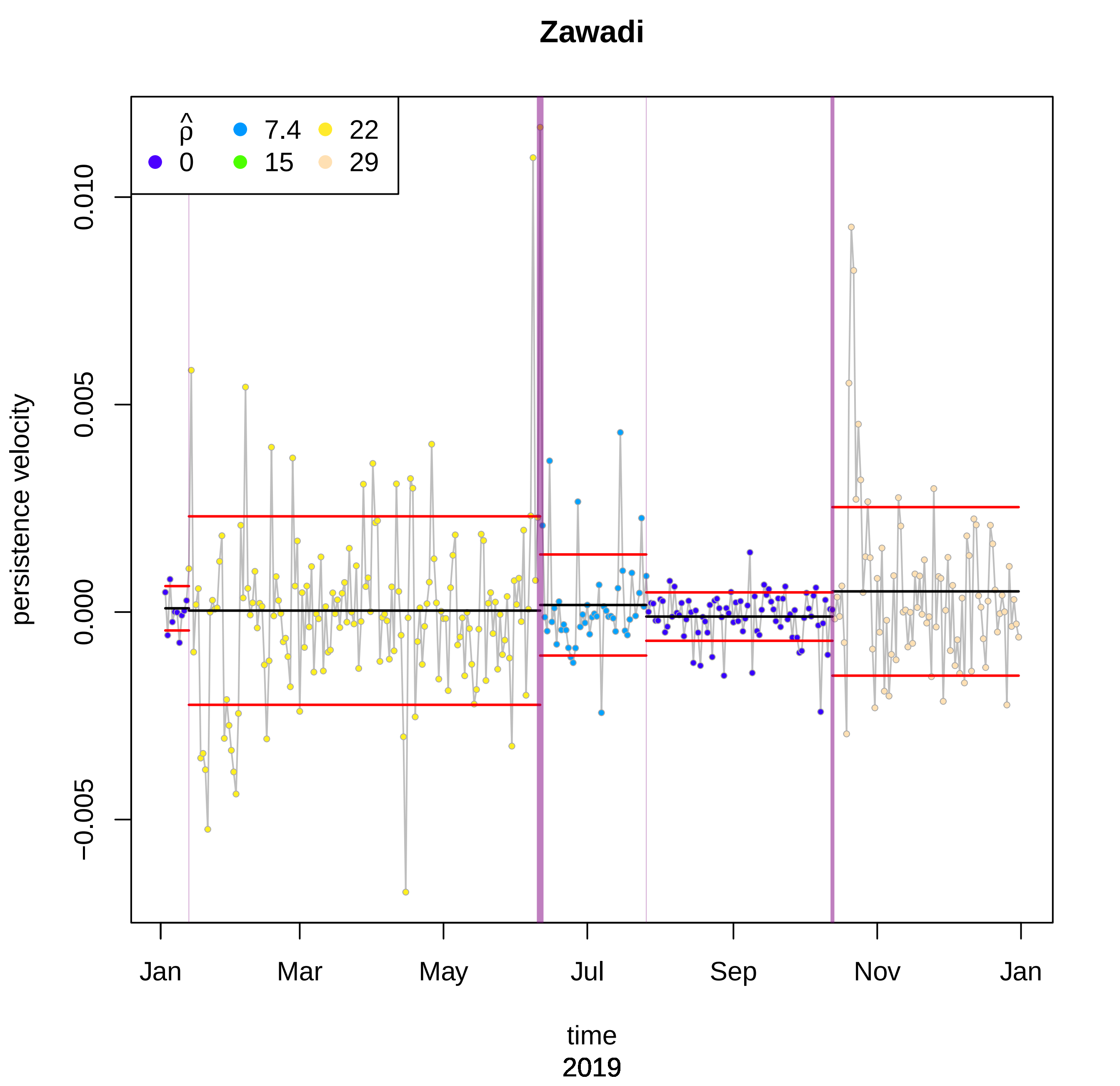

Zawadi
ρ̂
0
7.4
15
22
29
0.010
0.005
0.000
−0.005
persistence velocity
Jan
Mar
May
Jul
Sep
Nov
Jan
time
2019

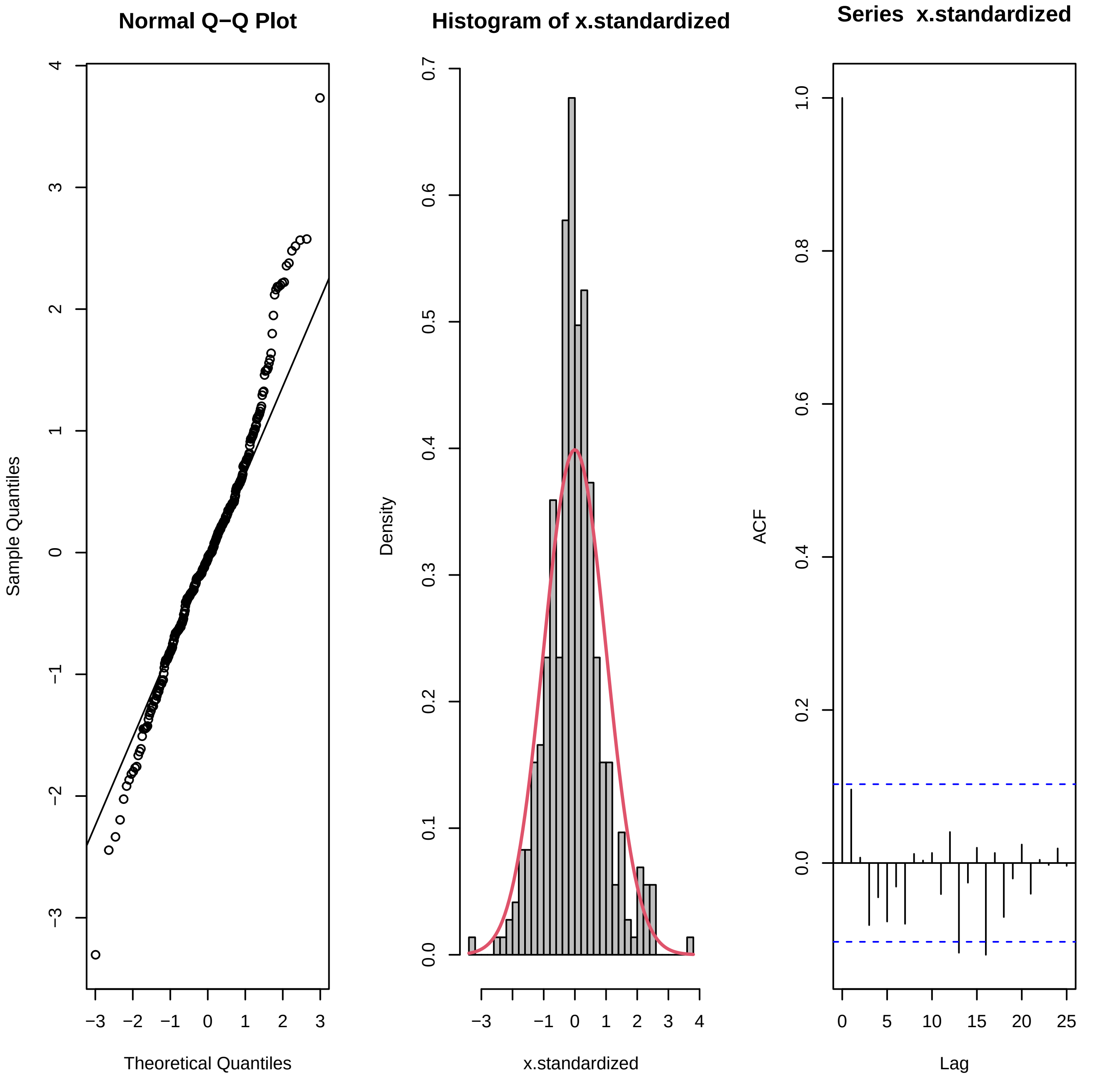
Normal Q−Q Plot
Sample Quantiles
Theoretical Quantiles
−3 −2 −1 0 1 2 3 4
Histogram of x.standardized
Density
x.standardized
0.0 0.1 0.2 0.3 0.4 0.5 0.6 0.7
−3 −1 0 1 2 3 4
Series x.standardized
ACF
Lag
0.0 0.2 0.4 0.6 0.8 1.0
0 5 10 15 20 25
