# Supplementary material for: Local rainfall is more likely than distant thunderstorms to affect movement behaviour in Northern Kenyan elephants
Source: PLoS One. 2024 Dec 23;19(12):e0307520. doi: 10.1371/journal.pone.0307520 (PMC11666045; doi:10.1371/journal.pone.0307520)
Supplement: S5 File — Blue and red markers respectively indicate position fixes from wet and dry period days. The areas populated with wet and dry period fixes are each outlined by their convex hull and shaded blue and red respectively. The black lined polygon indicates the total convex hull for all position fixes. The RHR itself is approximately visualised by the borders of the Latitude / Longitude border component in the figure. Scale bar indicates 0.2°, or approximately 22 km on the equator (53). (PDF) [file pone.0307520.s005.pdf]

Elephant ID: Amity

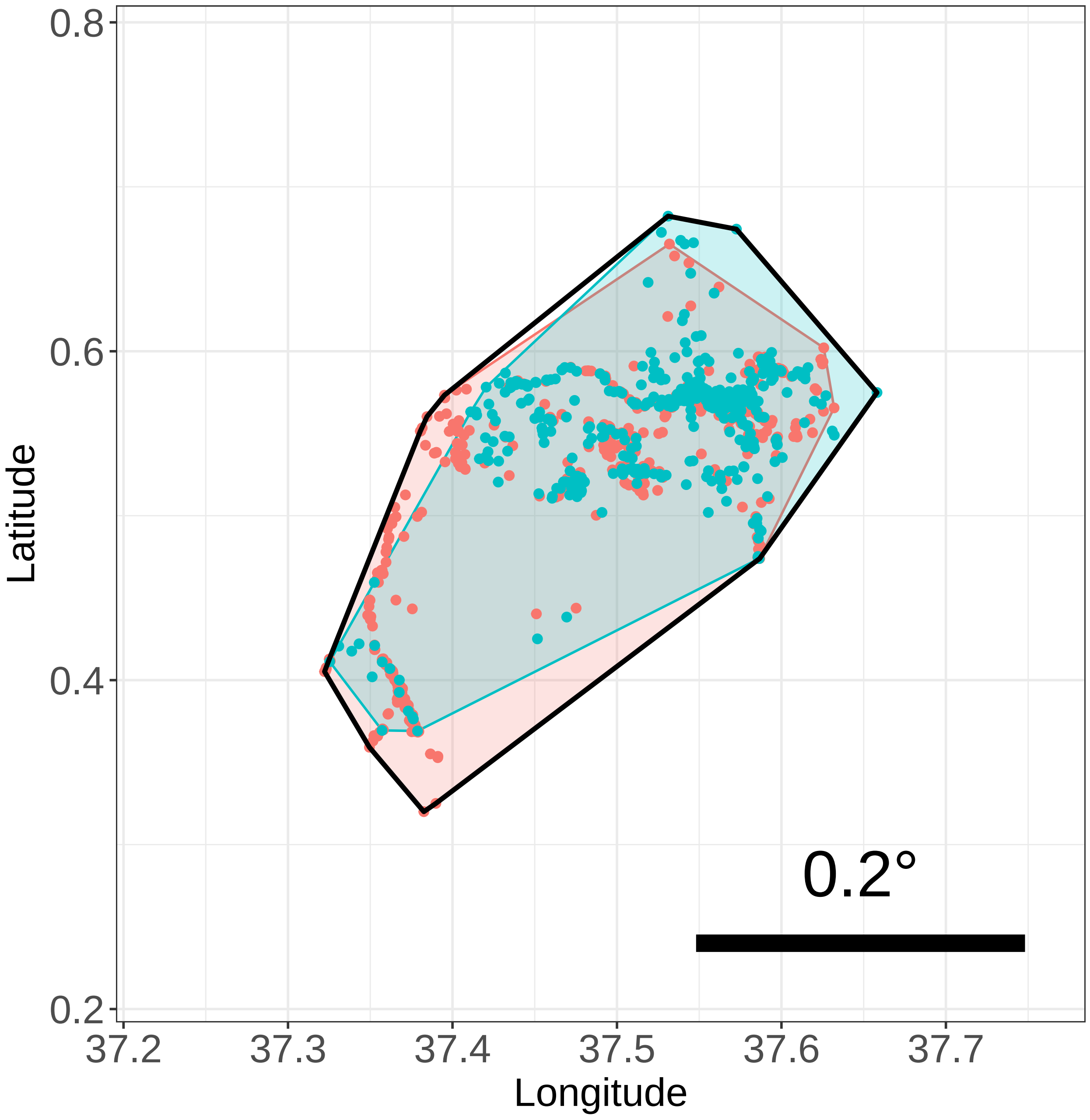

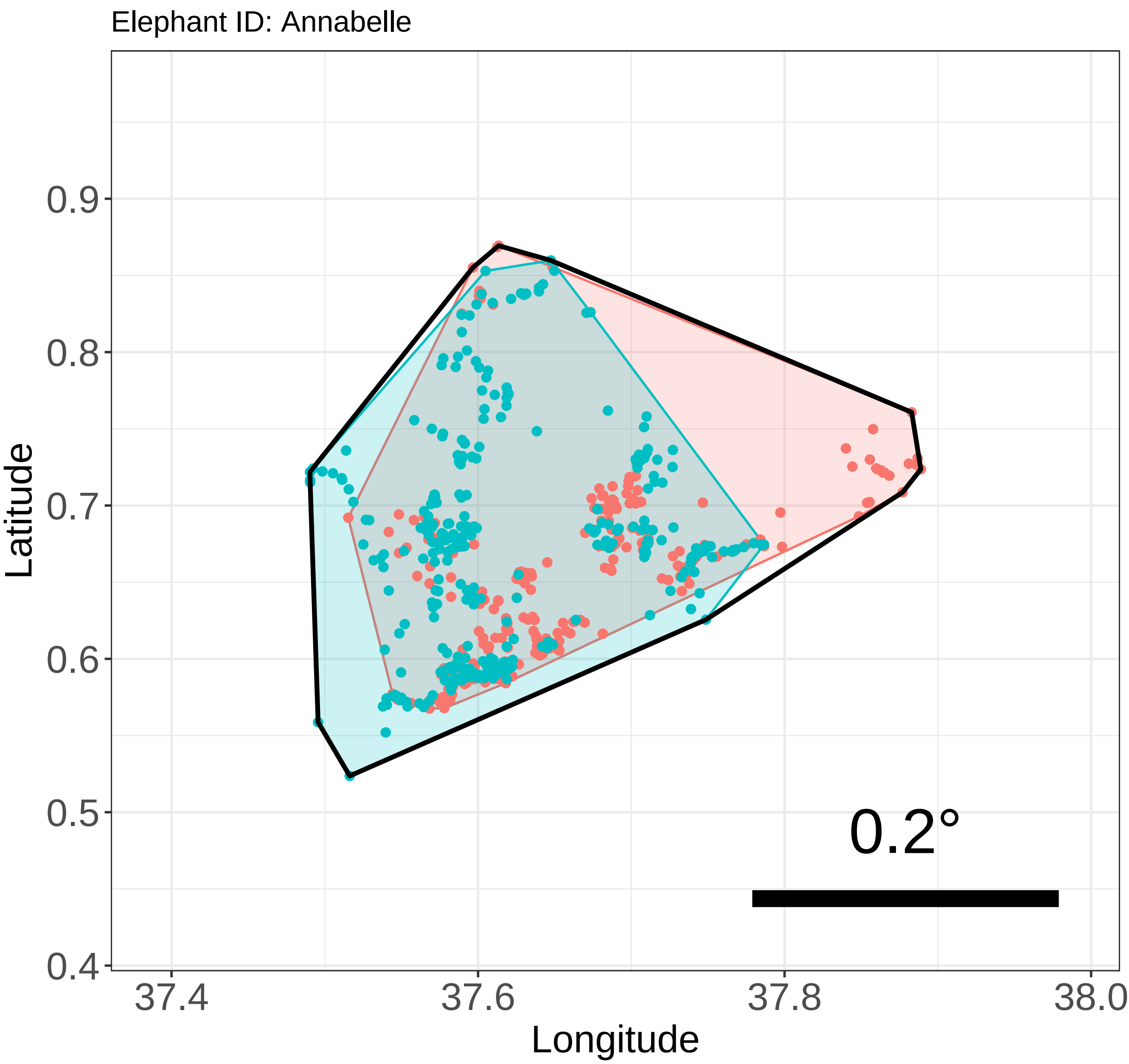

Elephant ID: Annabelle
0.9
0.8
0.7
0.6
0.5
0.4
Latitude
37.4
37.6
37.8
38.0
Longitude
0.2°

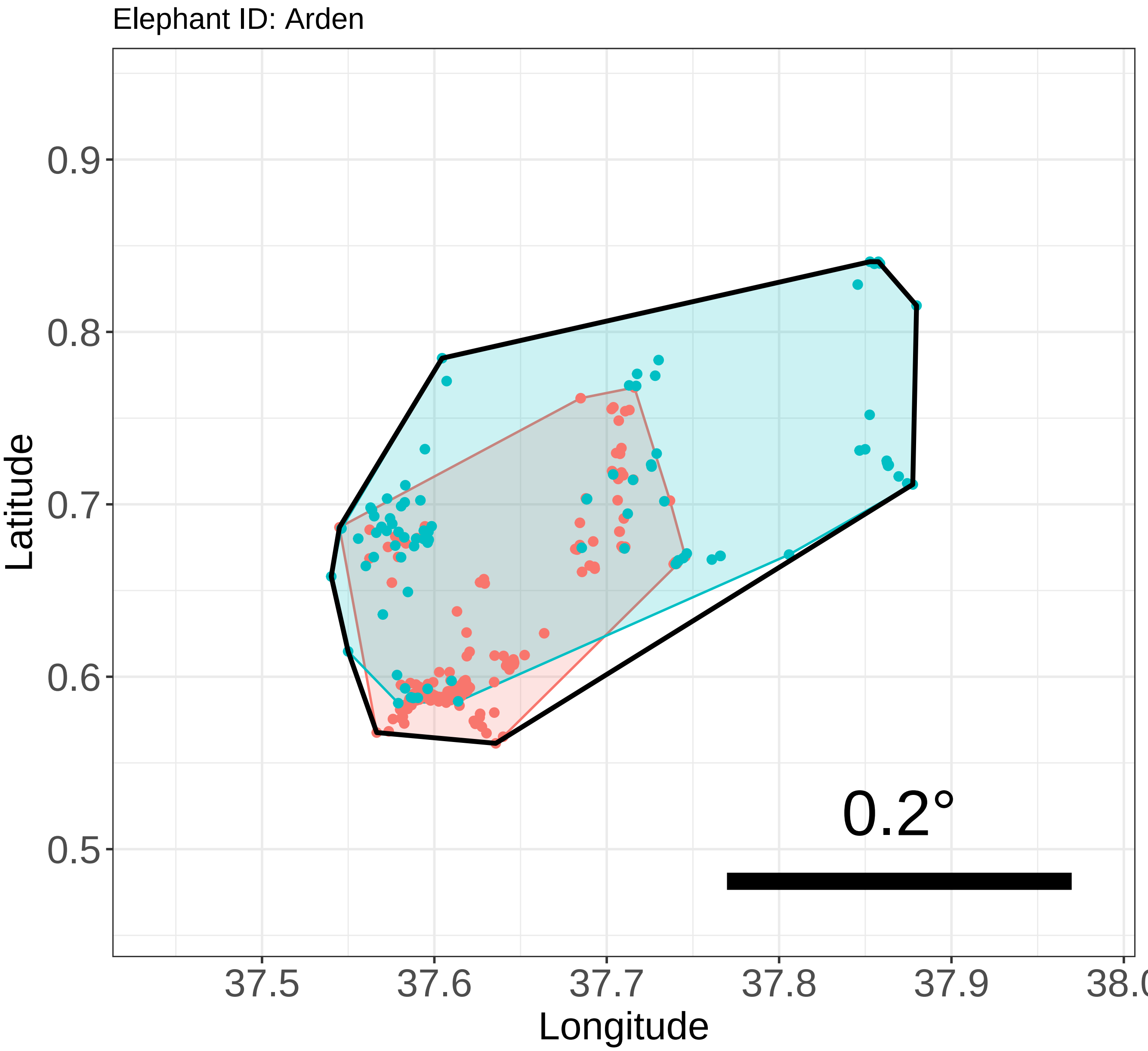

Elephant ID: Arden
0.9
0.8
0.7
0.6
0.5
Latitude
37.5
37.6
37.7
37.8
37.9
38.0
Longitude
0.2°

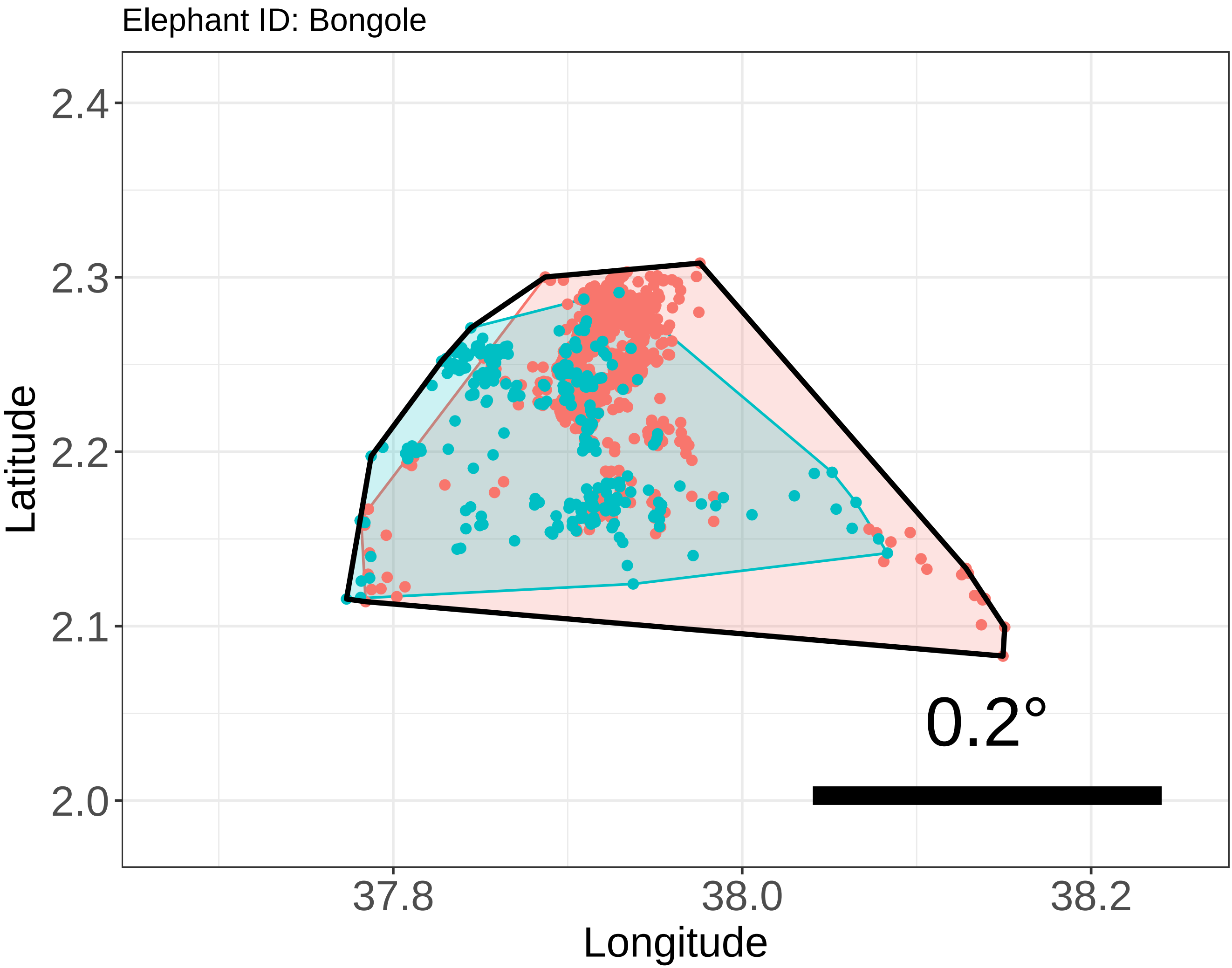

Elephant ID: Bongole
Latitude
2.4
2.3
2.2
2.1
2.0
37.8
38.0
38.2
Longitude
0.2°

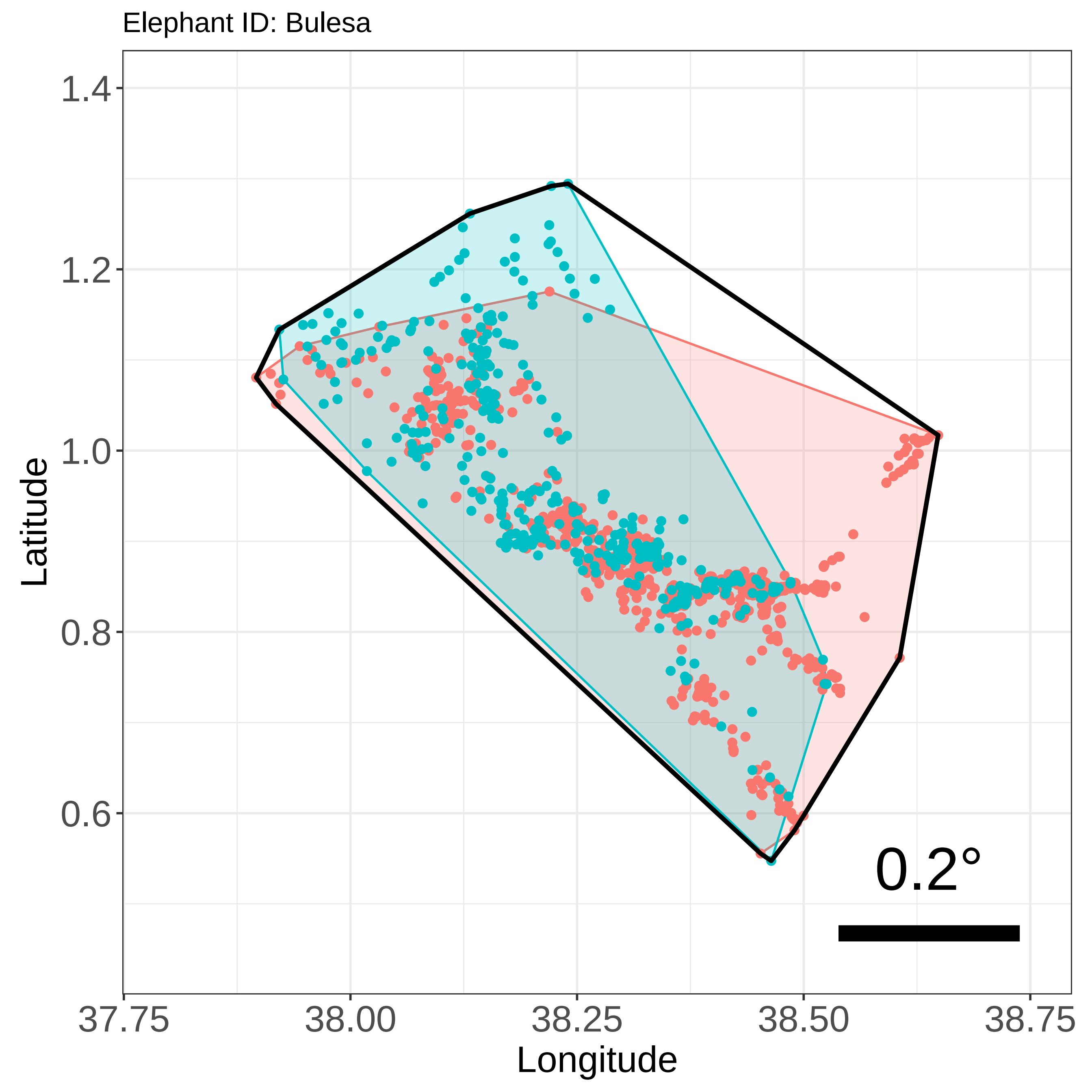
Elephant ID: Bulesa
Latitude
1.4
1.2
1.0
0.8
0.6
Longitude
37.75
38.00
38.25
38.50
38.75
0.2°

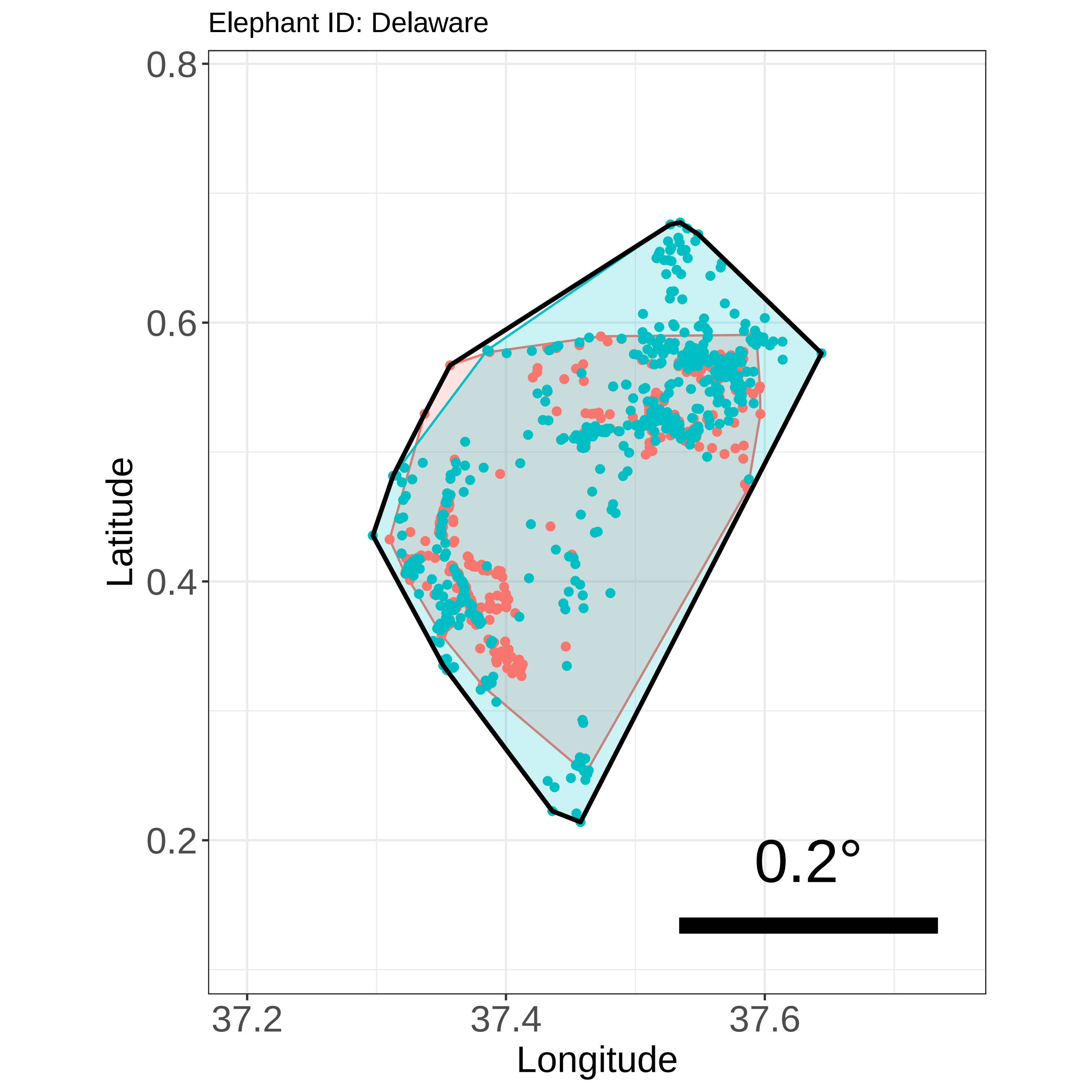

Elephant ID: Delaware
0.8
0.6
0.4
0.2
Latitude
37.2
37.4
37.6
Longitude
0.2°

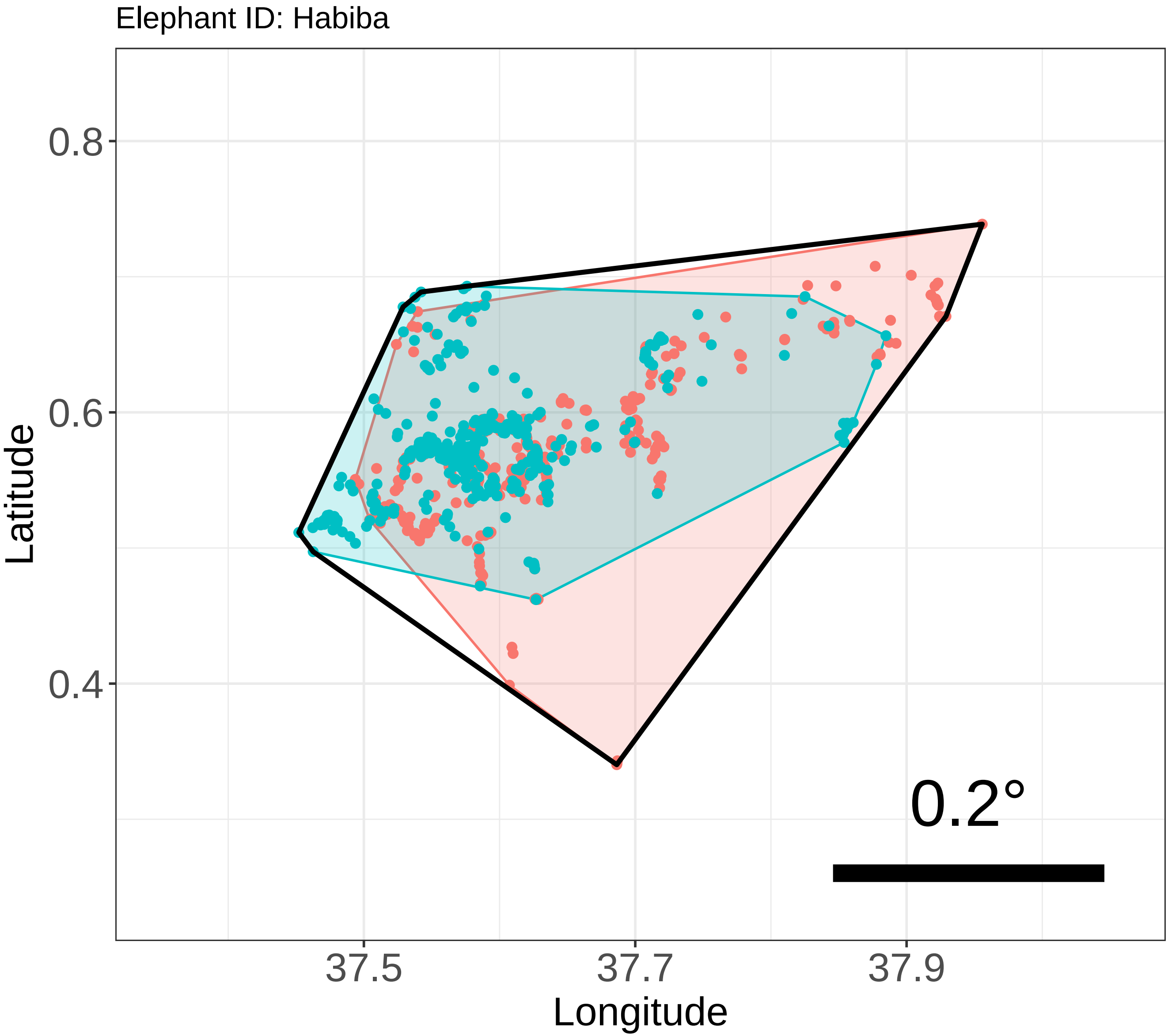

Elephant ID: Habiba
0.8
0.6
0.4
Latitude
37.5
37.7
37.9
Longitude
0.2°

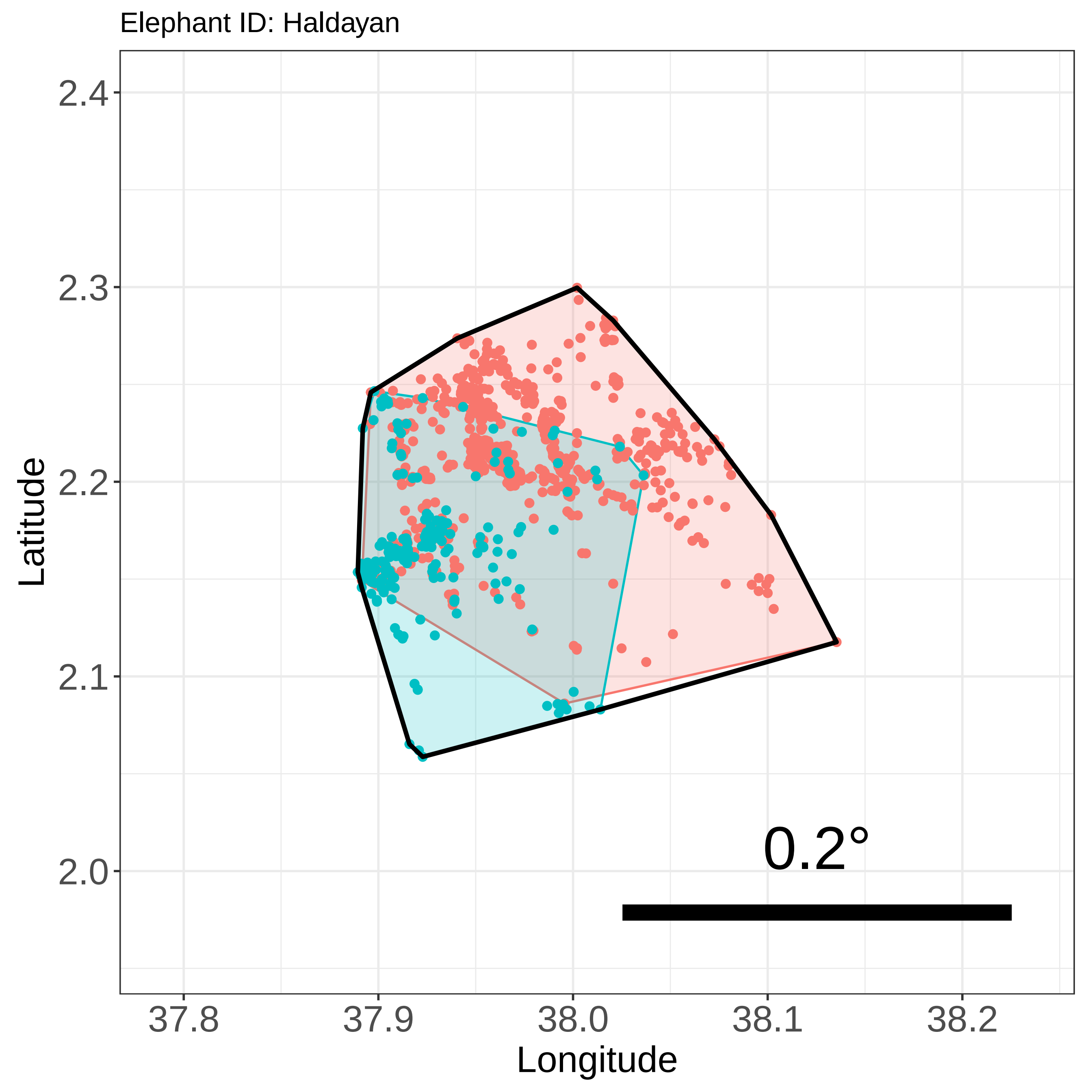

Elephant ID: Haldayan
Latitude
2.4
2.3
2.2
2.1
2.0
Longitude
37.8
37.9
38.0
38.1
38.2
0.2°

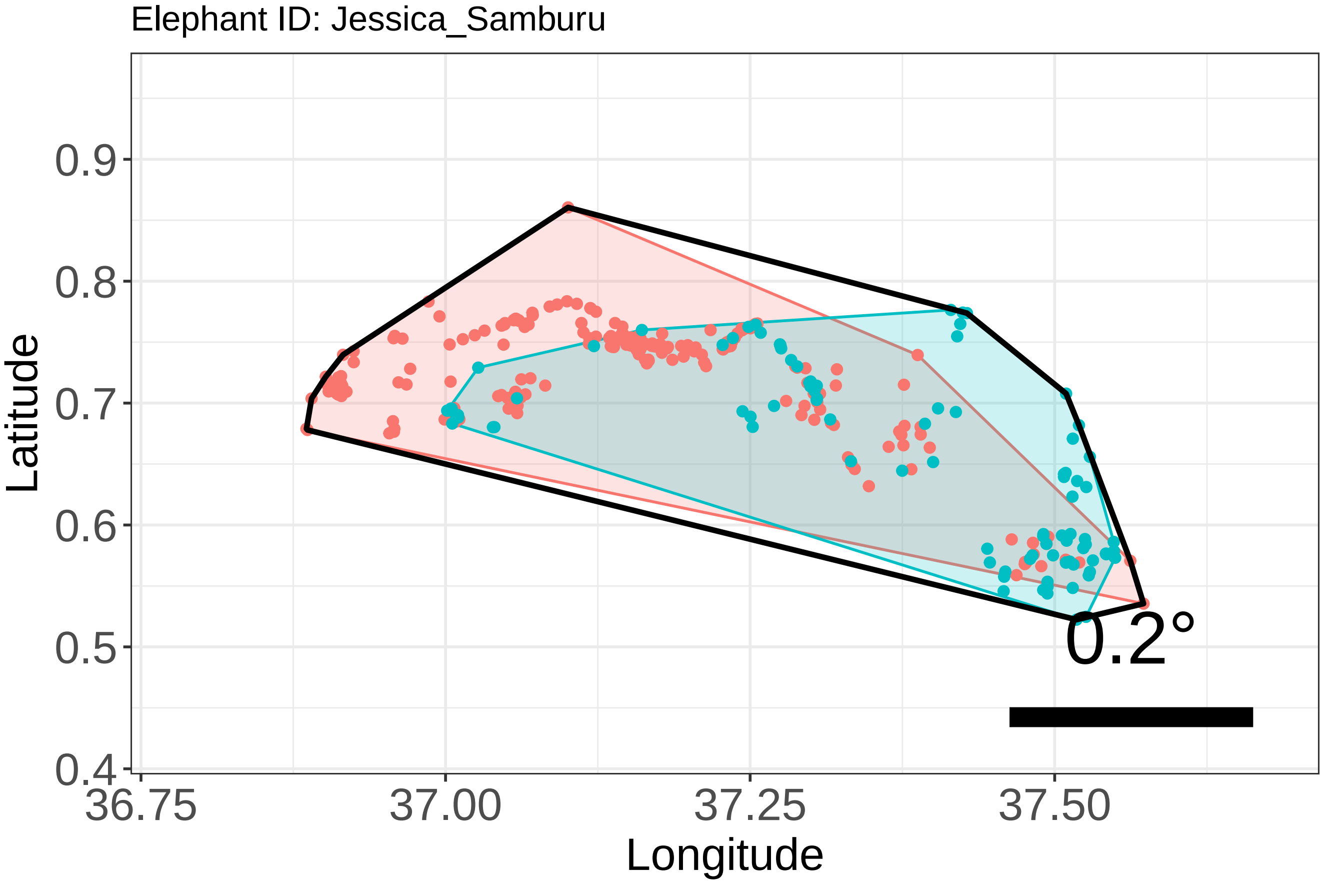

Elephant ID: Jessica_Samburu
0.9
0.8
0.7
0.6
0.5
0.4
Latitude
0.2°
36.75
37.00
37.25
37.50
Longitude

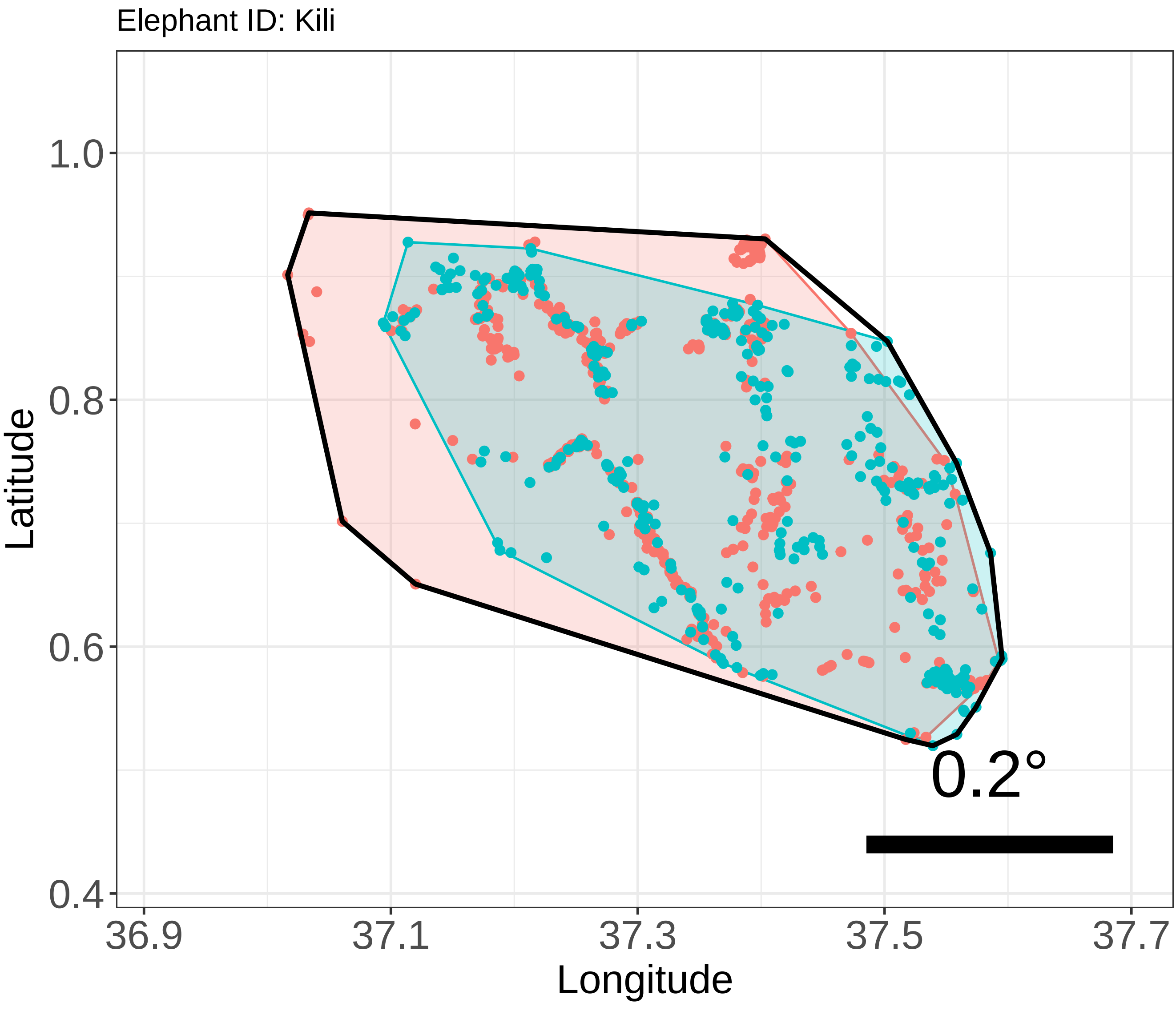

Elephant ID: Kili
1.0
0.8
0.6
0.4
Latitude
36.9
37.1
37.3
37.5
37.7
Longitude
0.2°

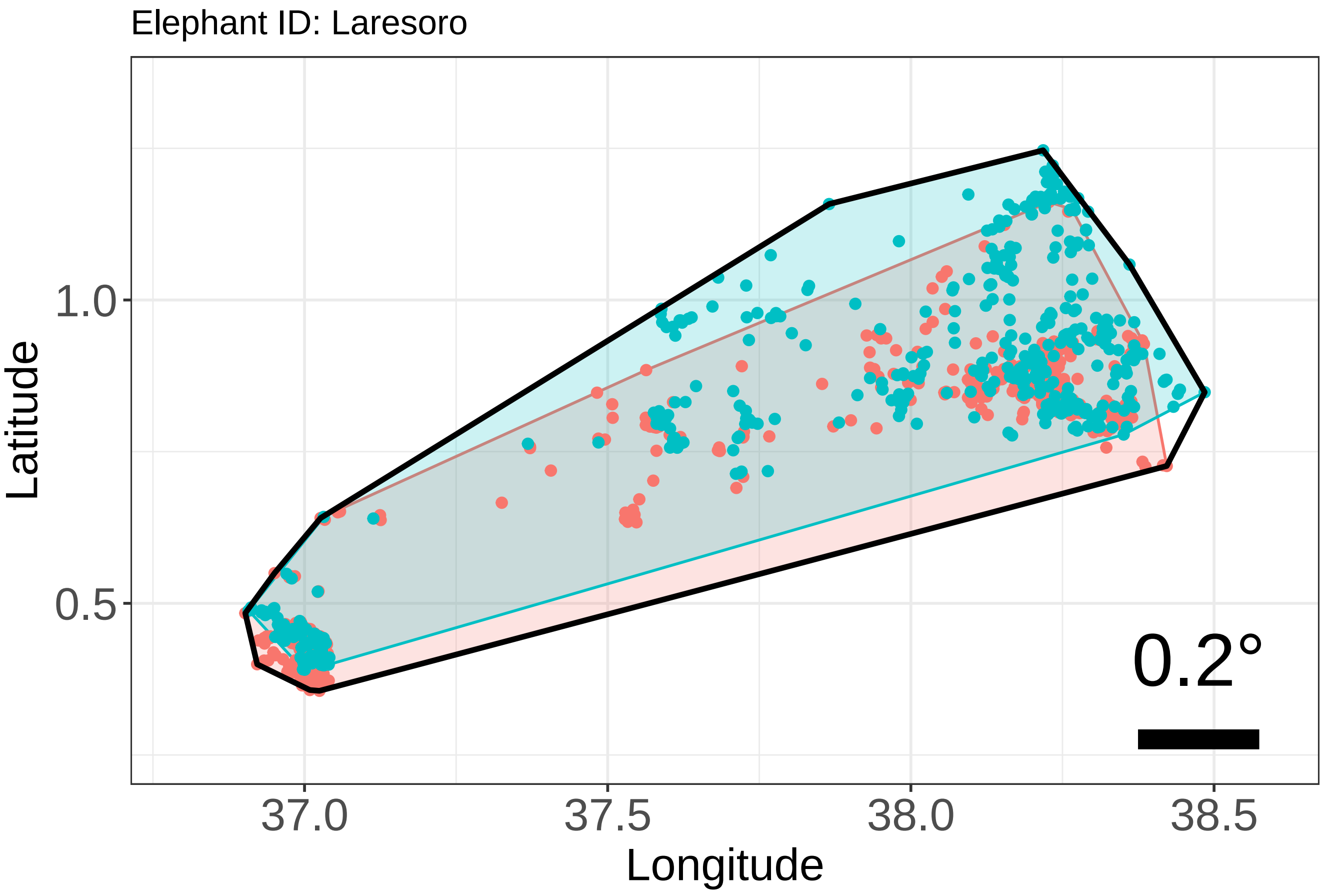
Elephant ID: Laresoro
Latitude
1.0
0.5
0.2°
37.0
37.5
38.0
38.5
Longitude

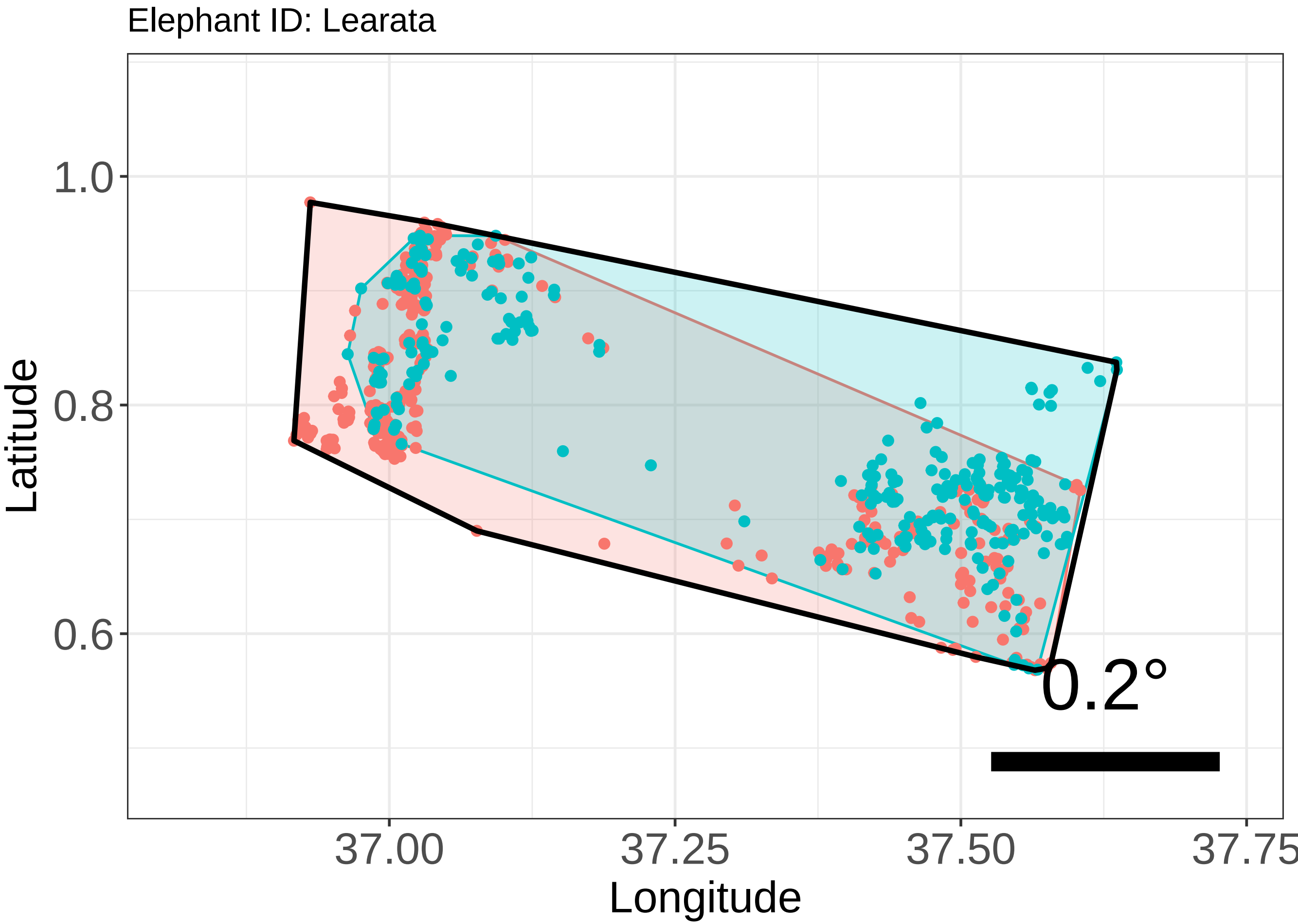

Elephant ID: Learata
1.0
0.8
0.6
Latitude
37.00
37.25
37.50
37.75
Longitude
0.2°

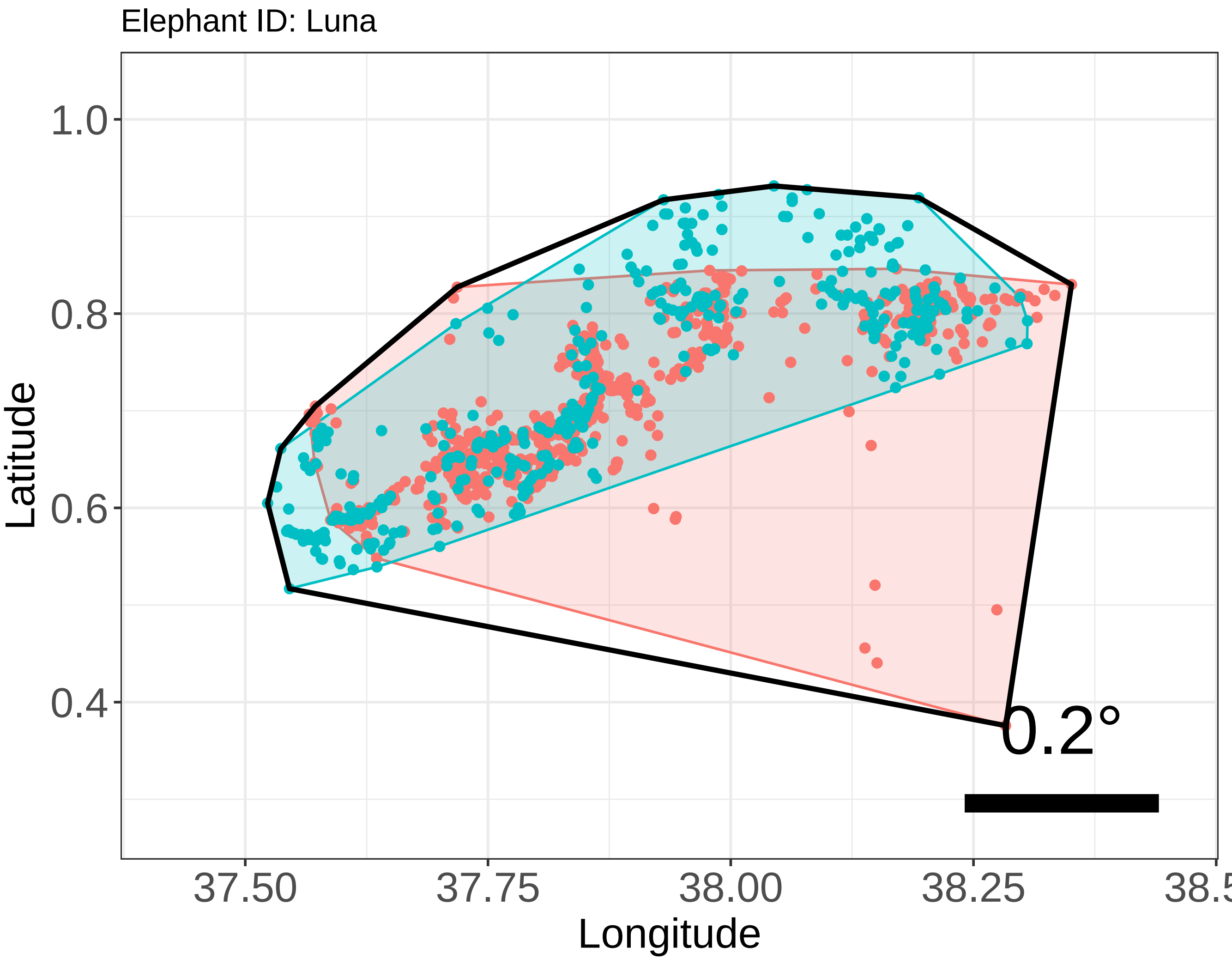

Elephant ID: Luna
1.0
0.8
0.6
0.4
Latitude
37.50
37.75
38.00
38.25
Longitude
0.2°

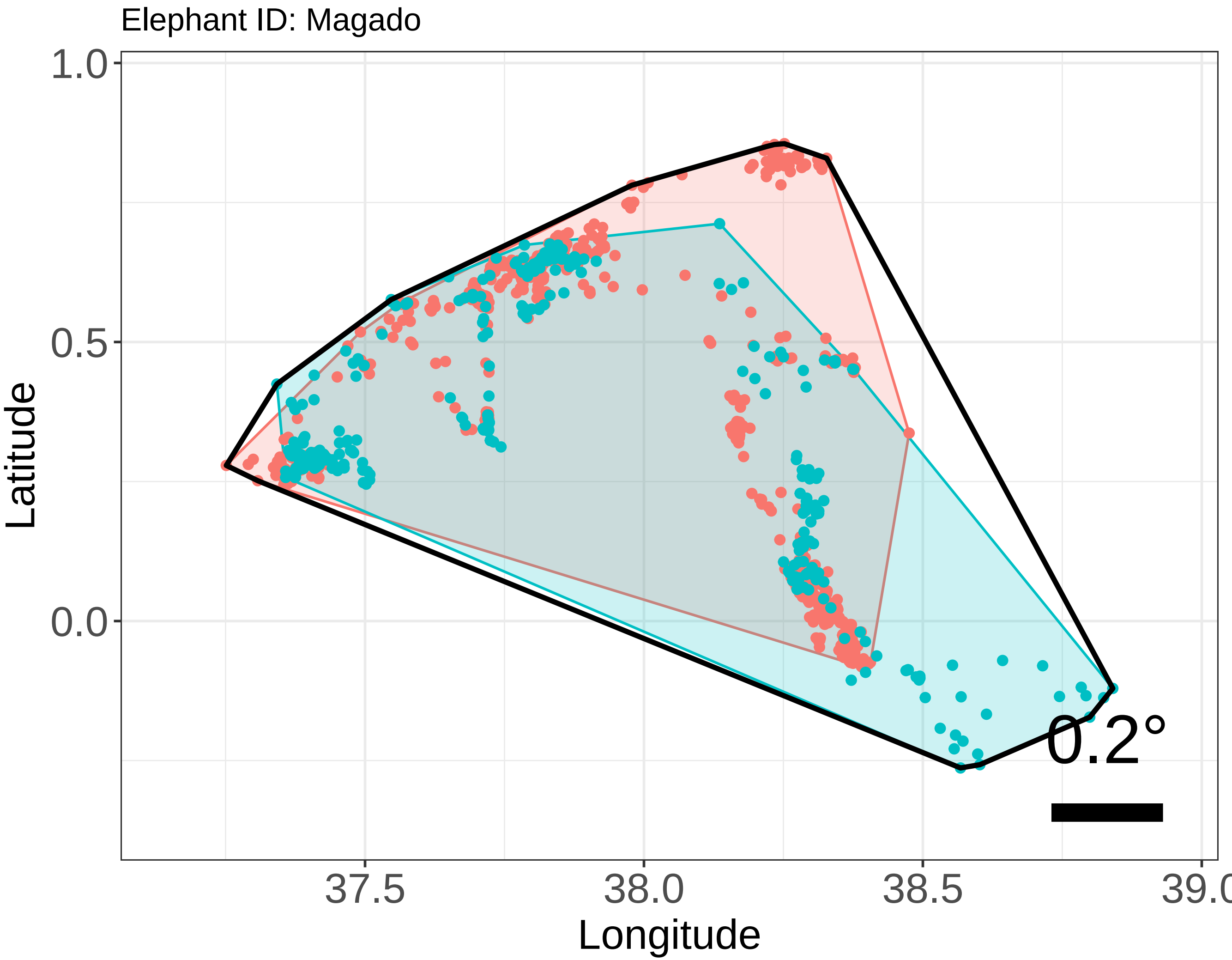

Elephant ID: Magado
1.0
0.5
0.0
Latitude
37.5
38.0
38.5
39.0
Longitude
0.2°

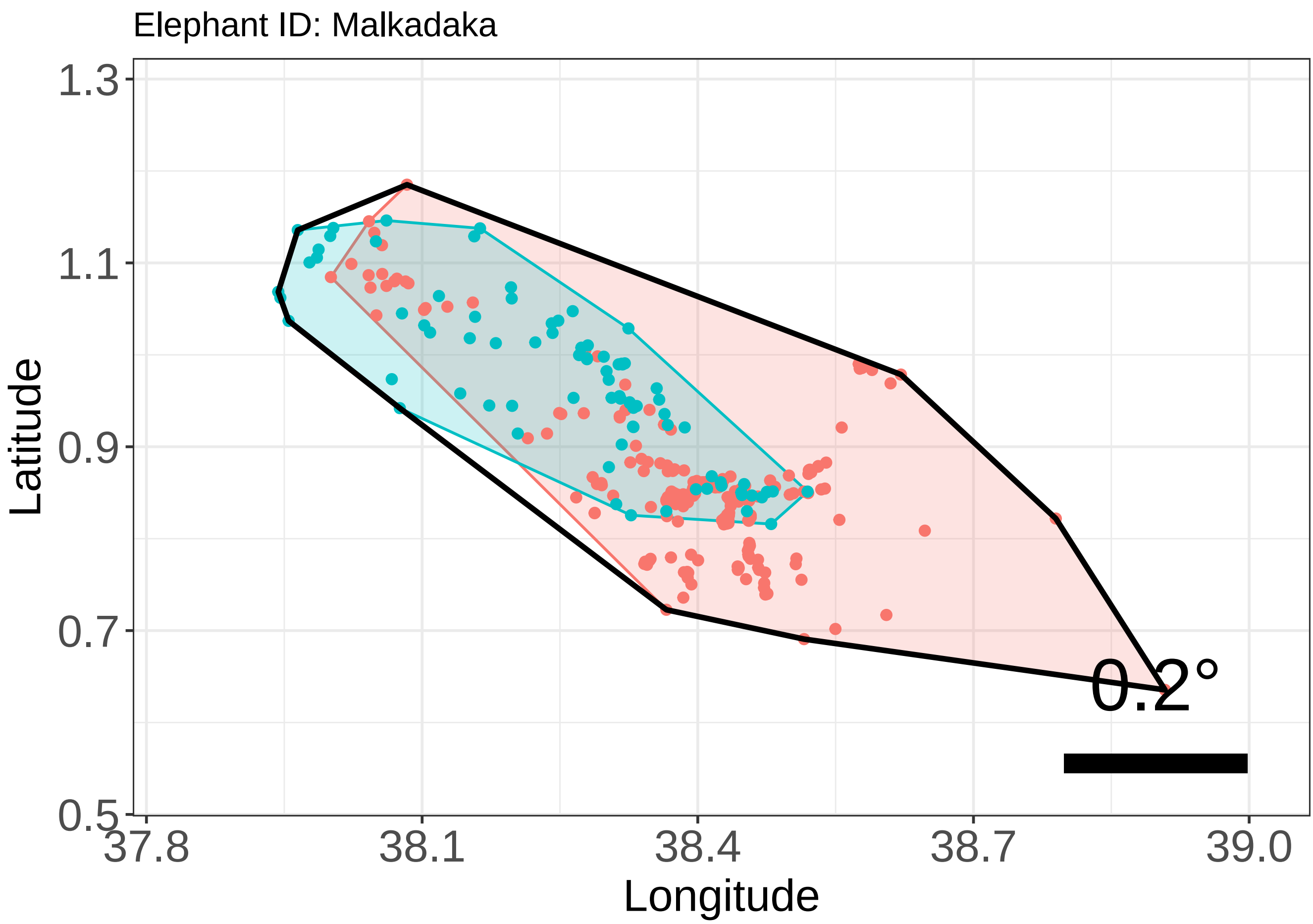

Elephant ID: Malkadaka
1.3
1.1
0.9
0.7
0.5
Latitude
37.8
38.1
38.4
38.7
39.0
Longitude
0.2°

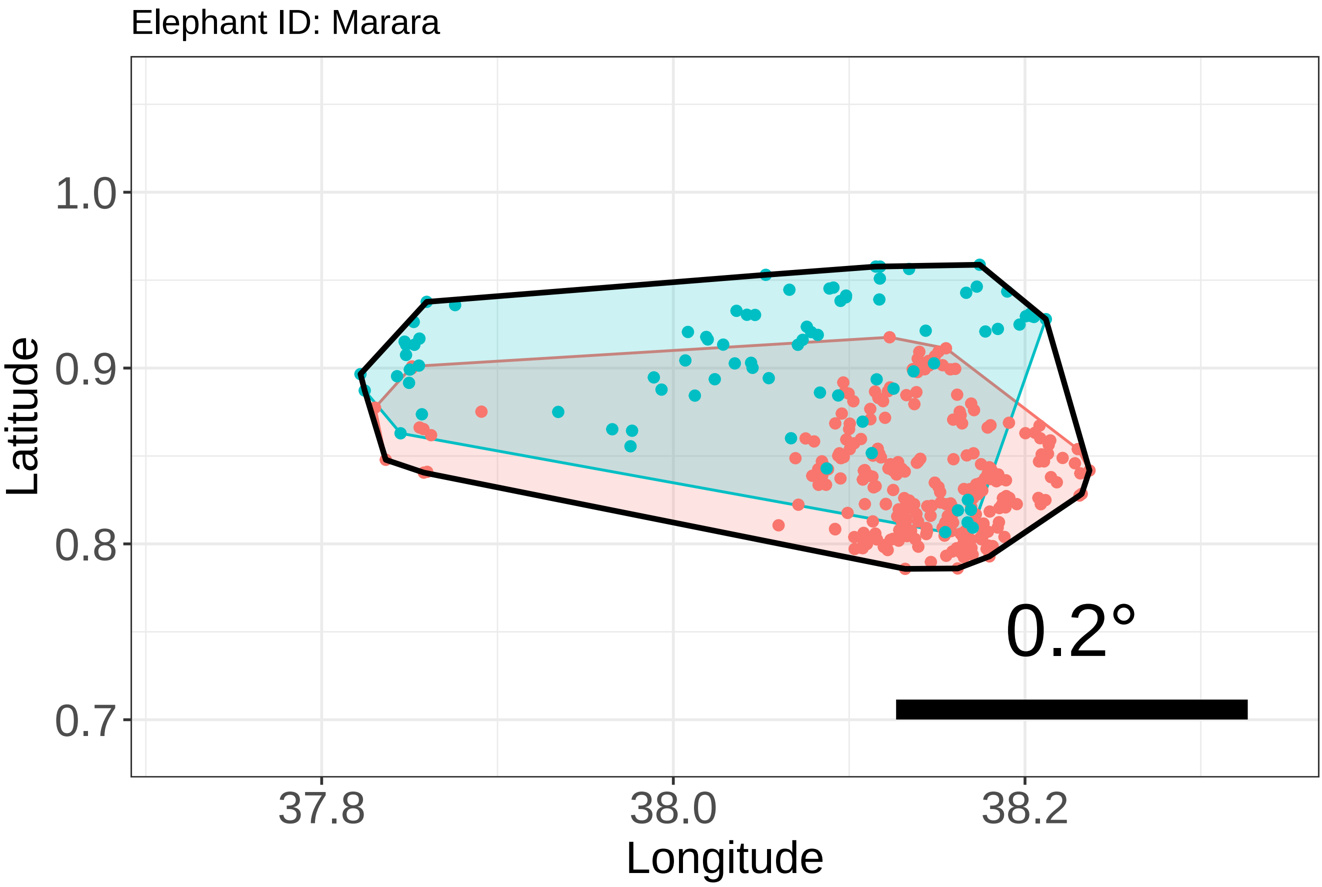

Elephant ID: Marara
1.0
0.9
0.8
0.7
Latitude
37.8
38.0
38.2
Longitude
0.2°

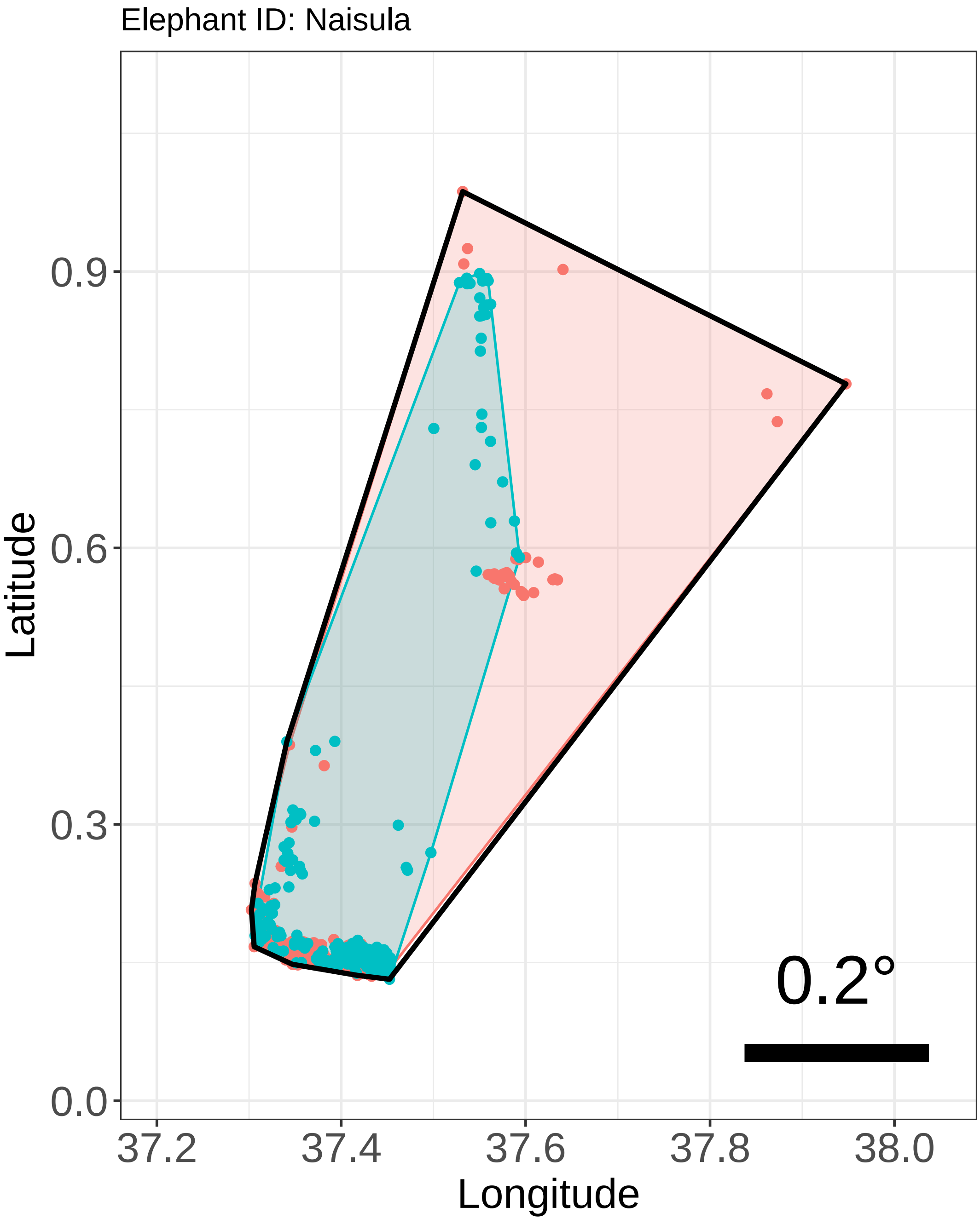

Elephant ID: Naisula
Latitude
0.9
0.6
0.3
0.0
Longitude
37.2
37.4
37.6
37.8
38.0
0.2°

Elephant ID: Namunyak

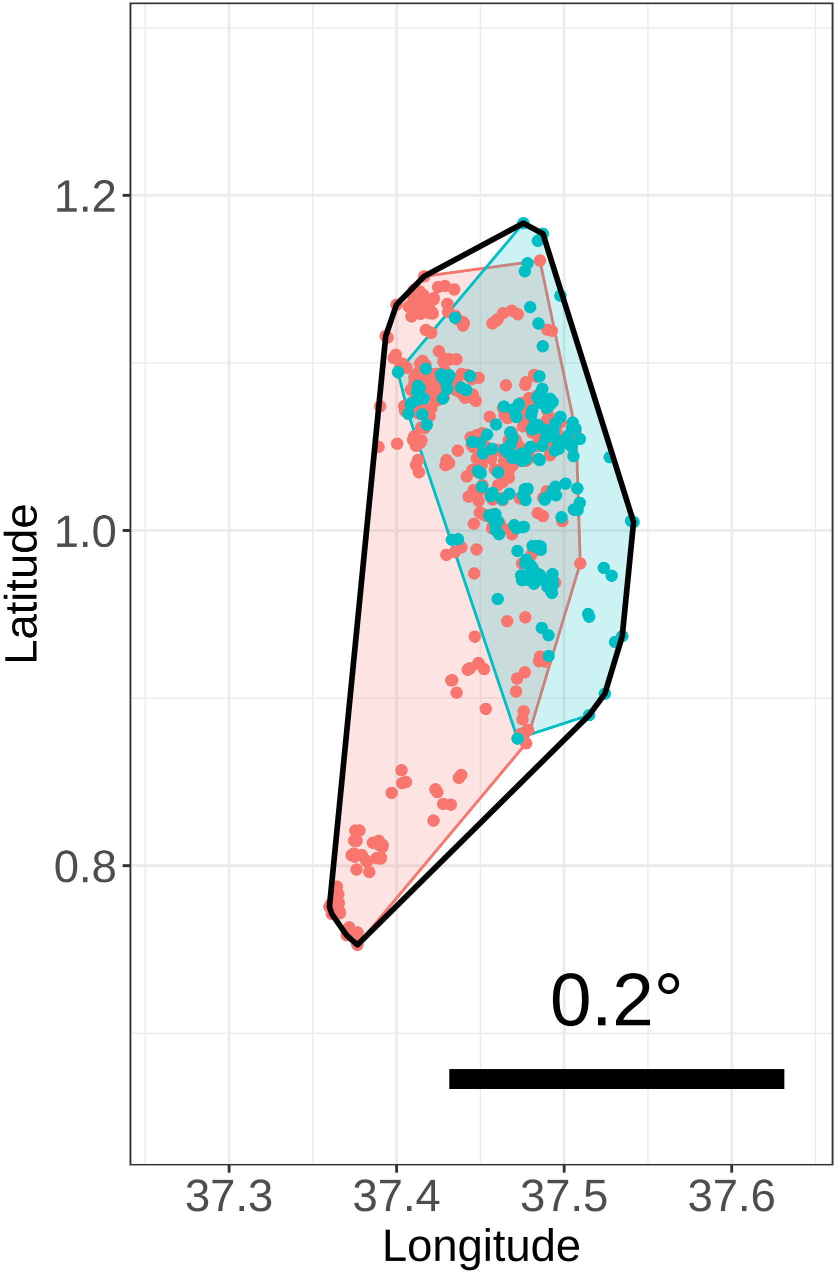

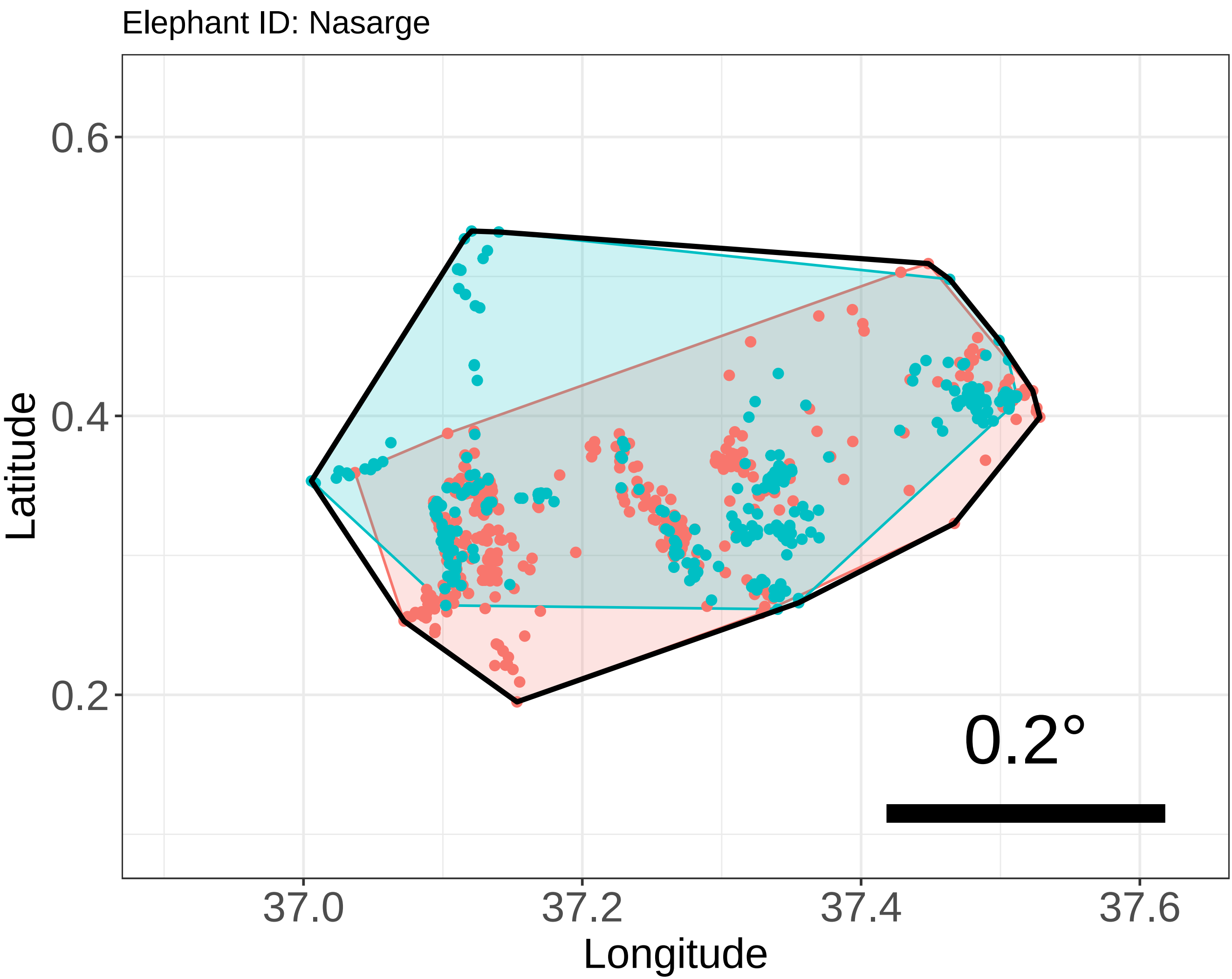
Elephant ID: Nasarge
0.6
0.4
0.2
Latitude
37.0
37.2
37.4
37.6
Longitude
0.2°

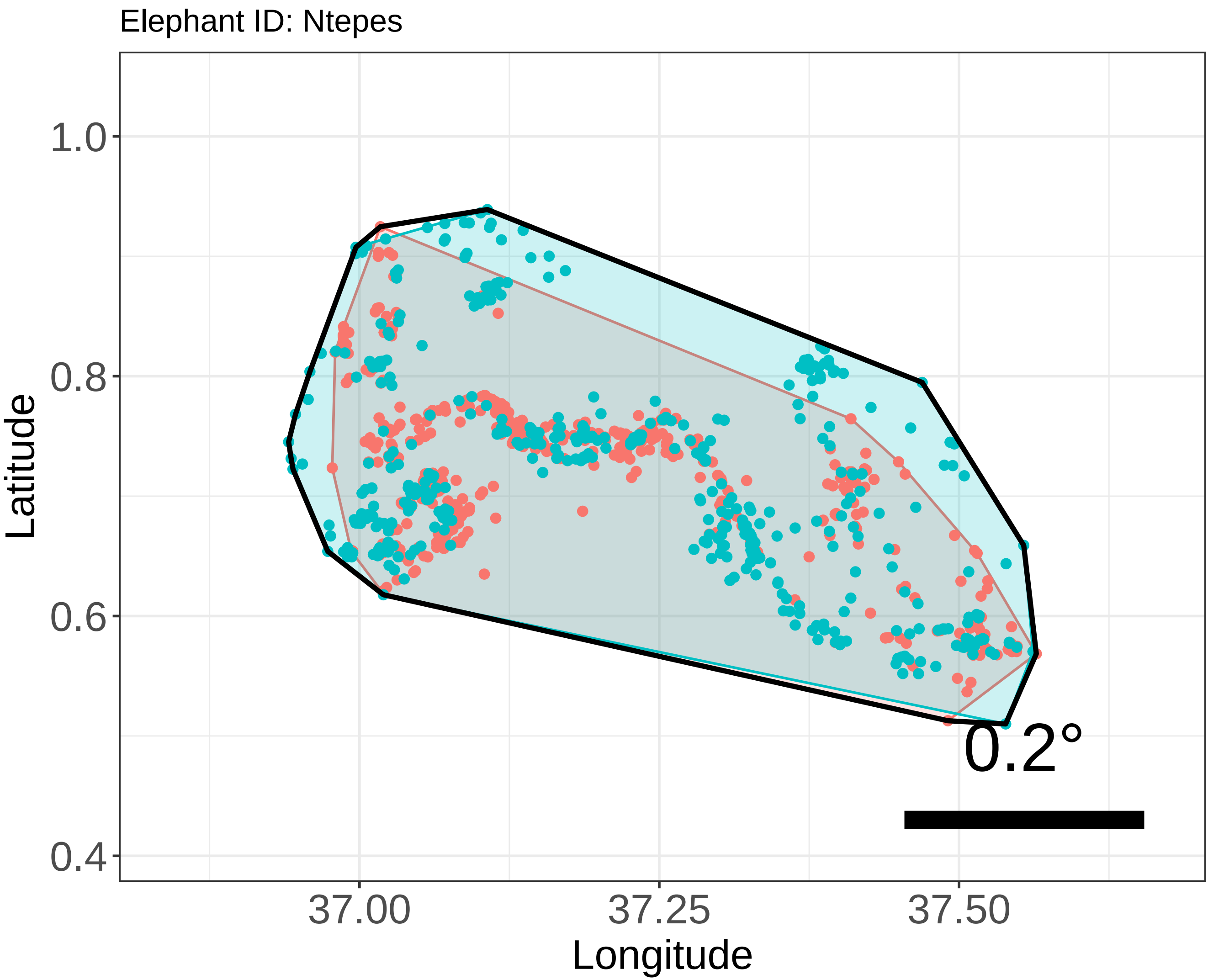

Elephant ID: Ntepes
1.0
0.8
0.6
0.4
Latitude
37.00
37.25
37.50
Longitude
0.2°

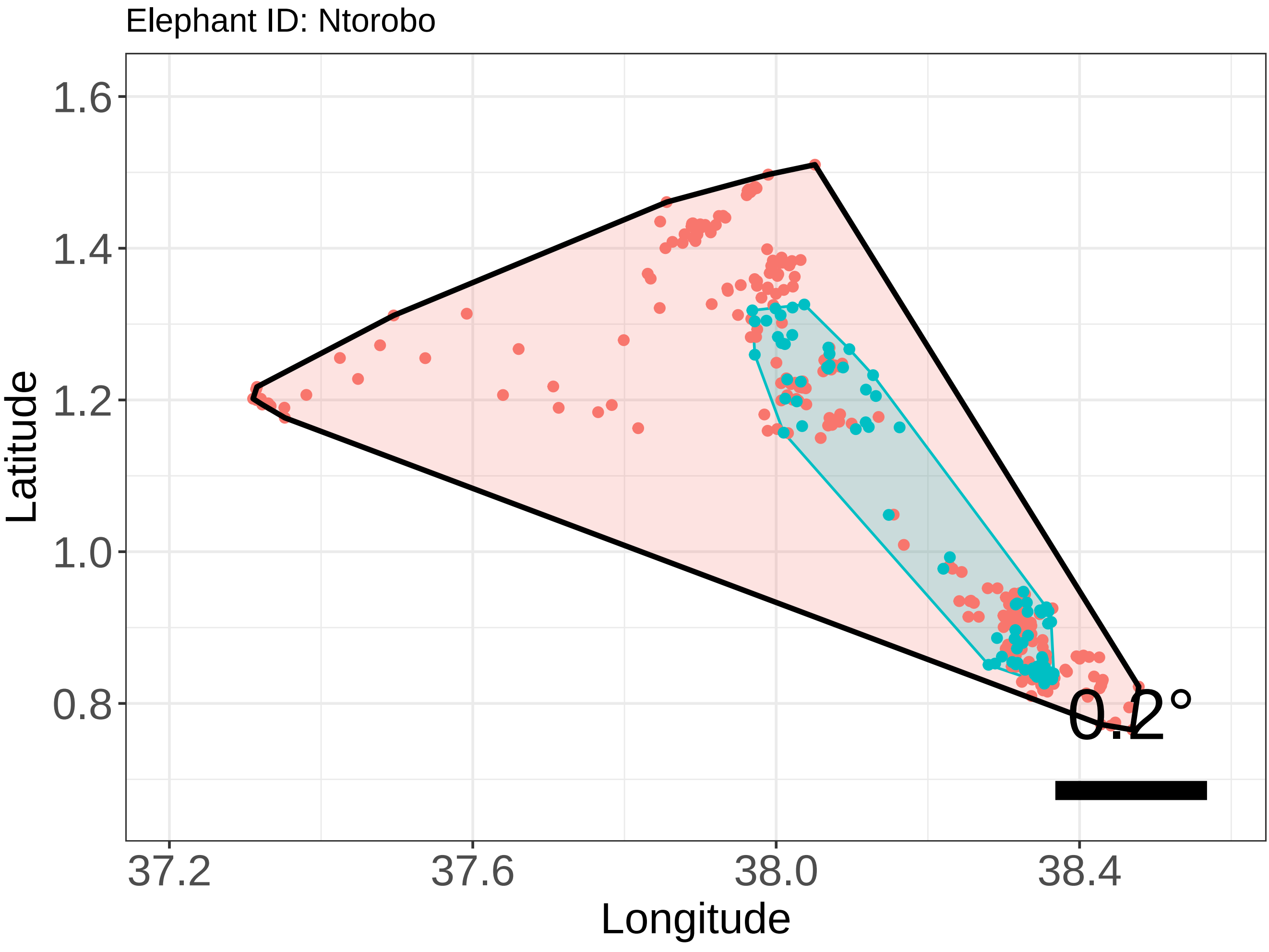

Elephant ID: Ntorobo
1.6
1.4
1.2
1.0
0.8
Latitude
37.2
37.6
38.0
38.4
Longitude
0.2°

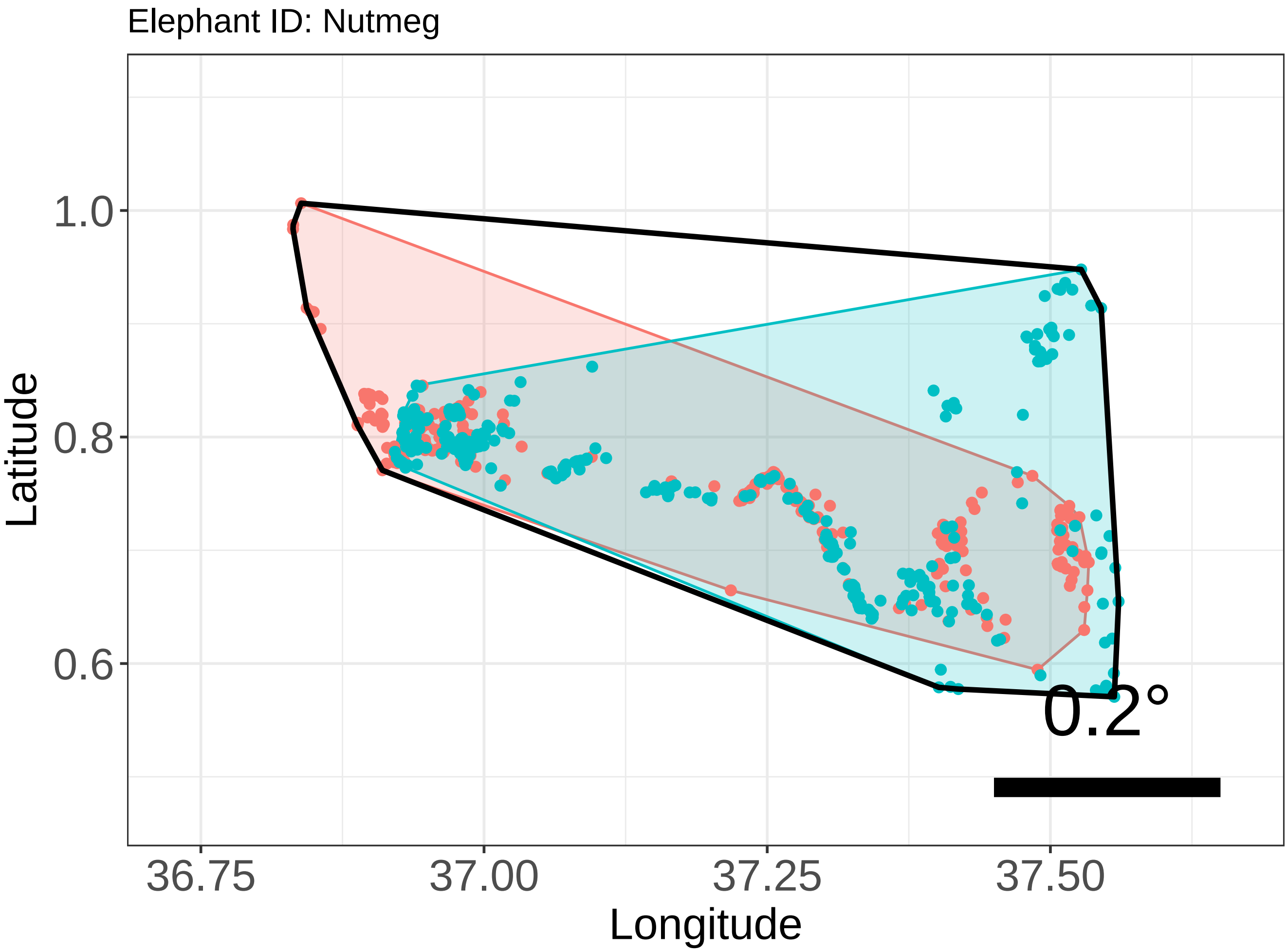

Elephant ID: Nutmeg
1.0
0.8
0.6
Latitude
36.75
37.00
37.25
37.50
Longitude
0.2°

Elephant ID: Orchid

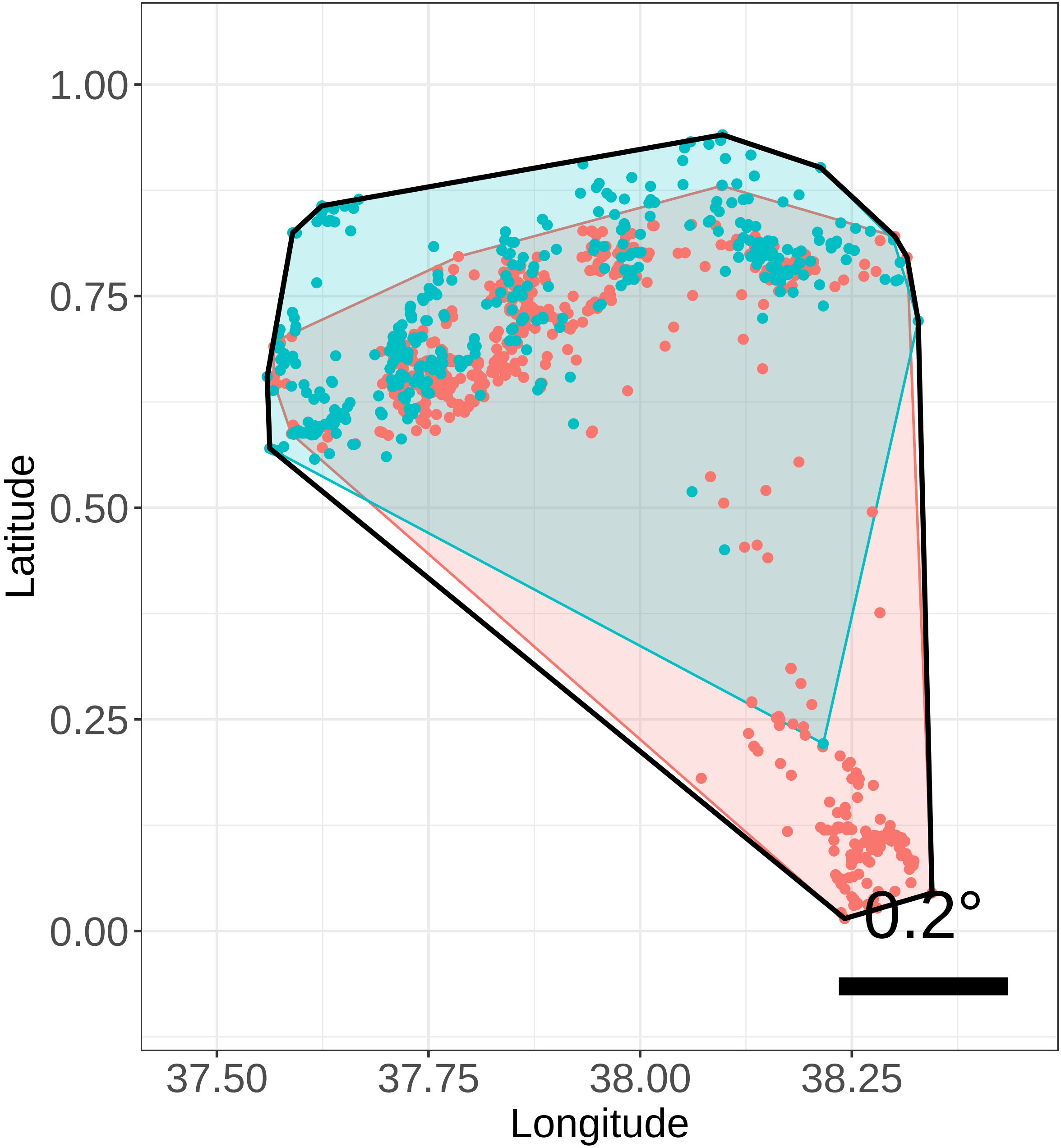

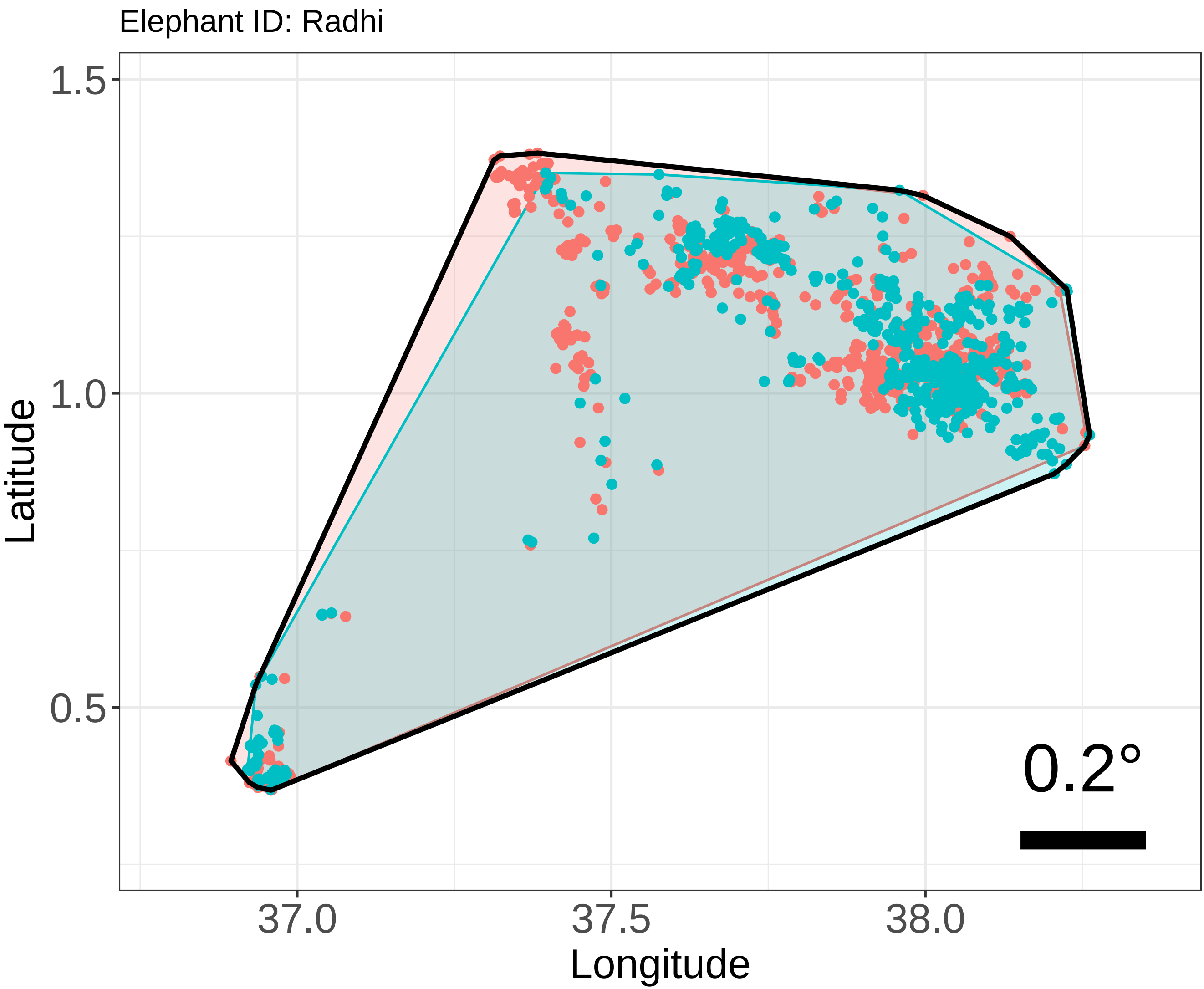

Elephant ID: Radhi
1.5
1.0
0.5
Latitude
37.0
37.5
38.0
Longitude
0.2°

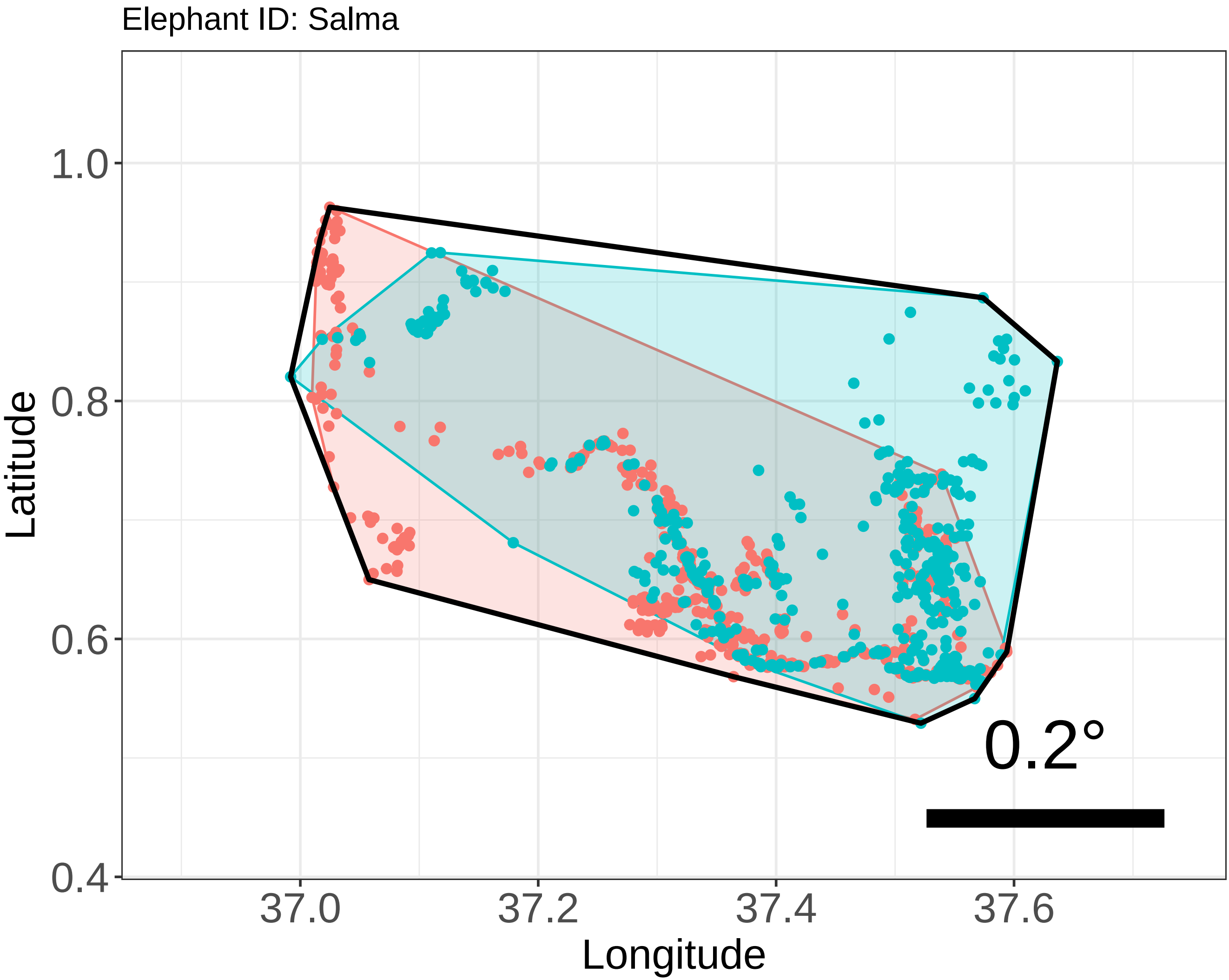

Elephant ID: Salma
1.0
0.8
0.6
0.4
Latitude
37.0
37.2
37.4
37.6
Longitude
0.2°

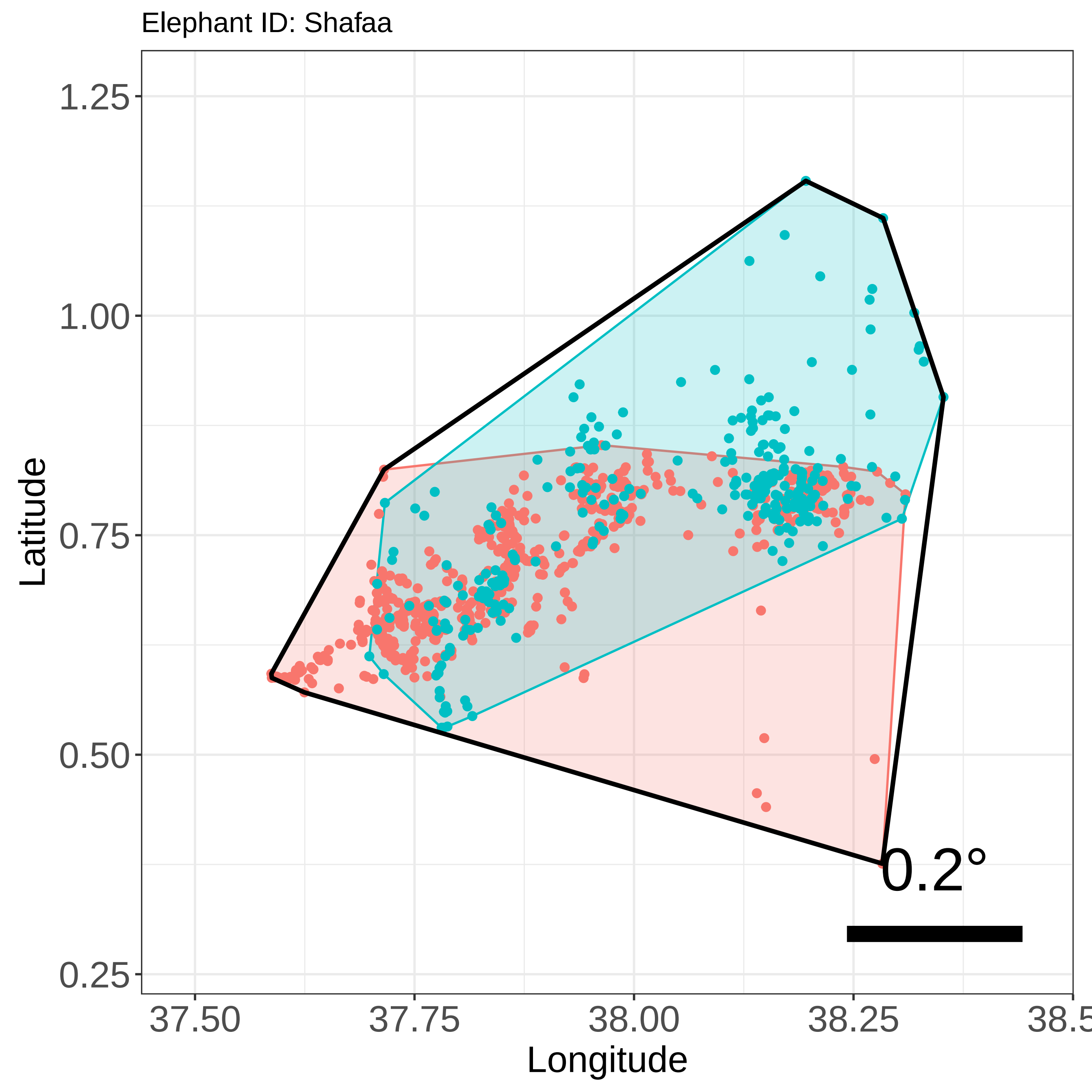

Elephant ID: Shafaa
1.25
1.00
0.75
0.50
0.25
Latitude
37.50
37.75
38.00
38.25
Longitude
0.2°

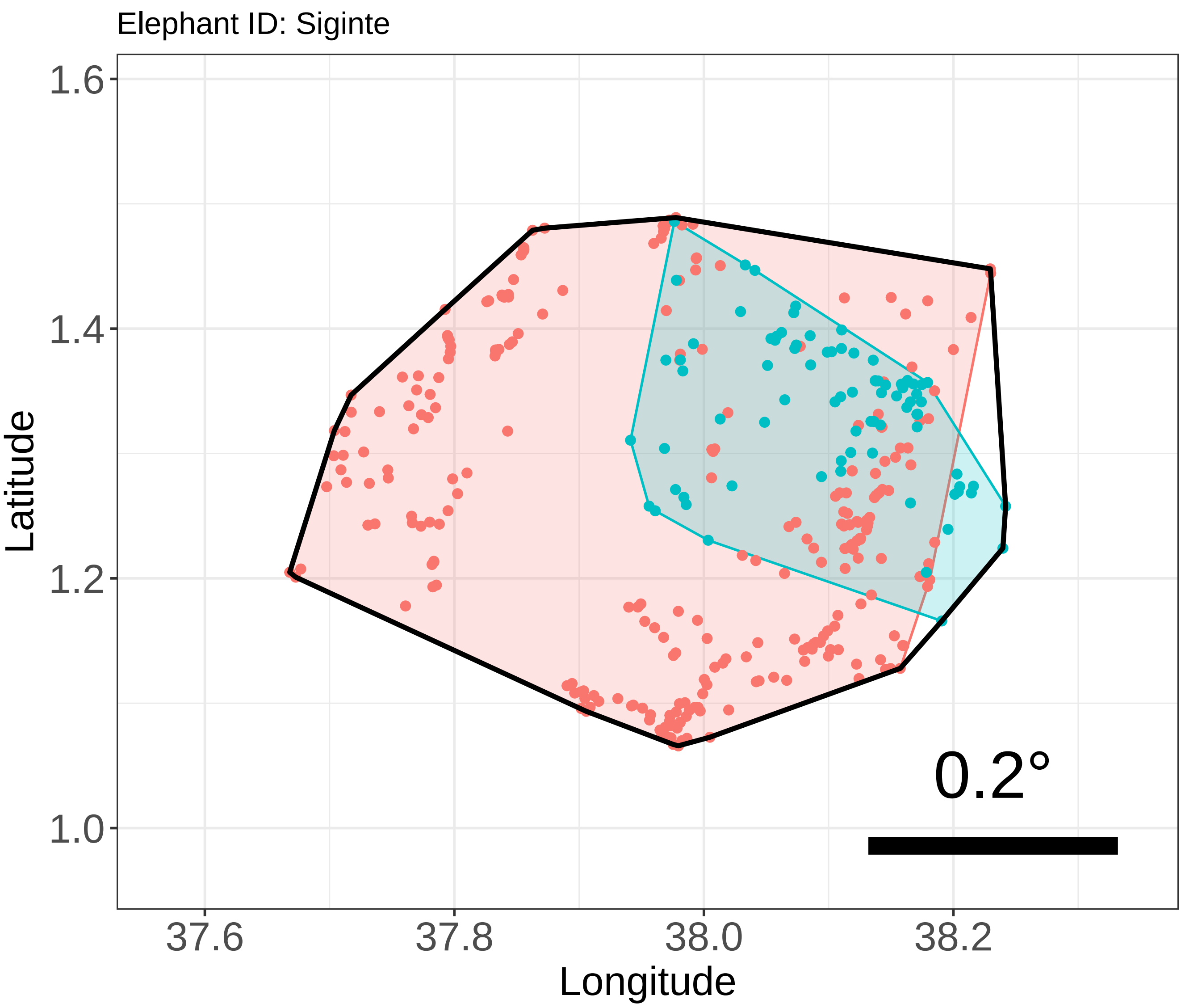
Elephant ID: Siginte
1.6
1.4
1.2
1.0
Latitude
37.6
37.8
38.0
38.2
Longitude
0.2°

Elephant ID: Songa

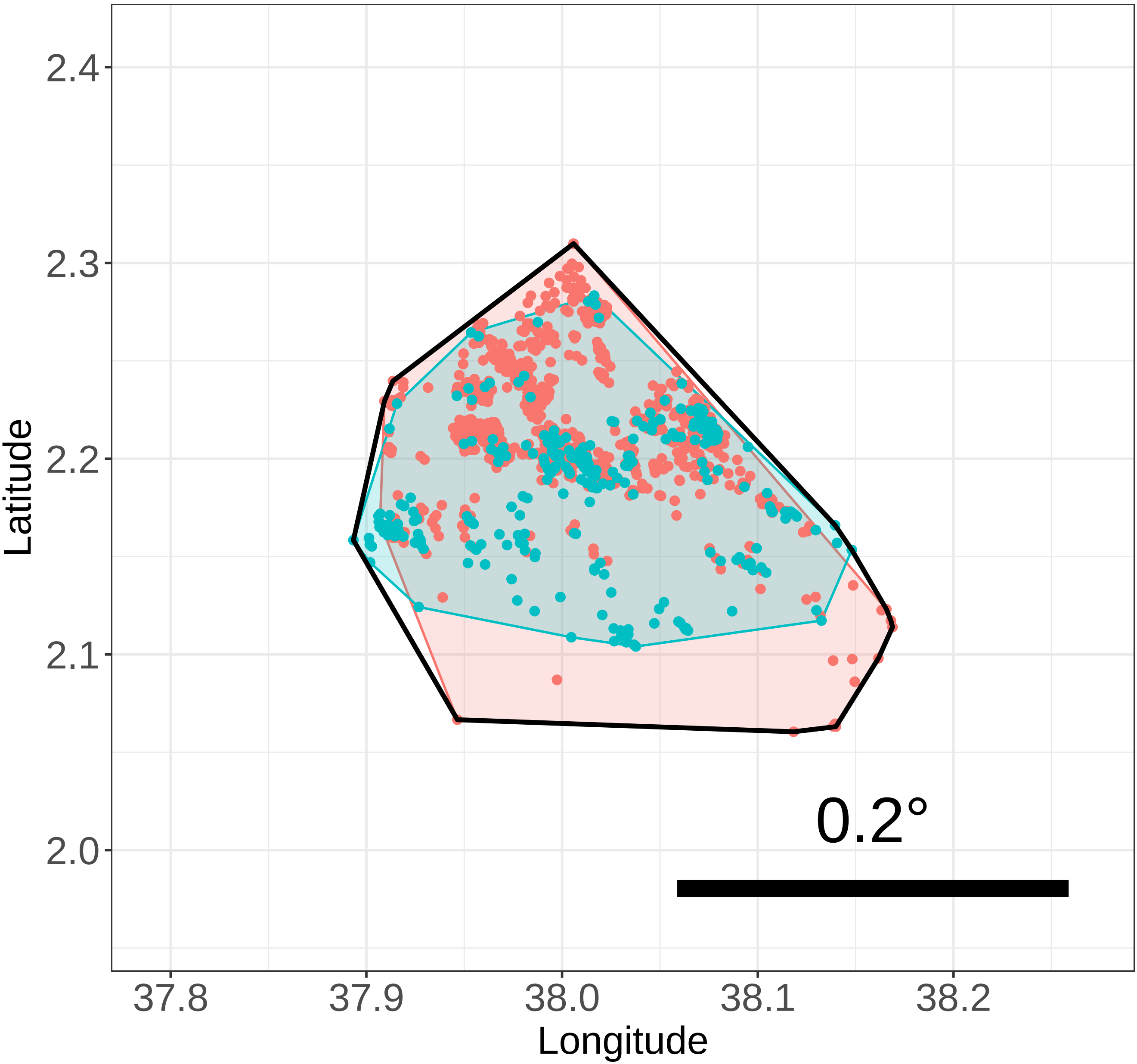

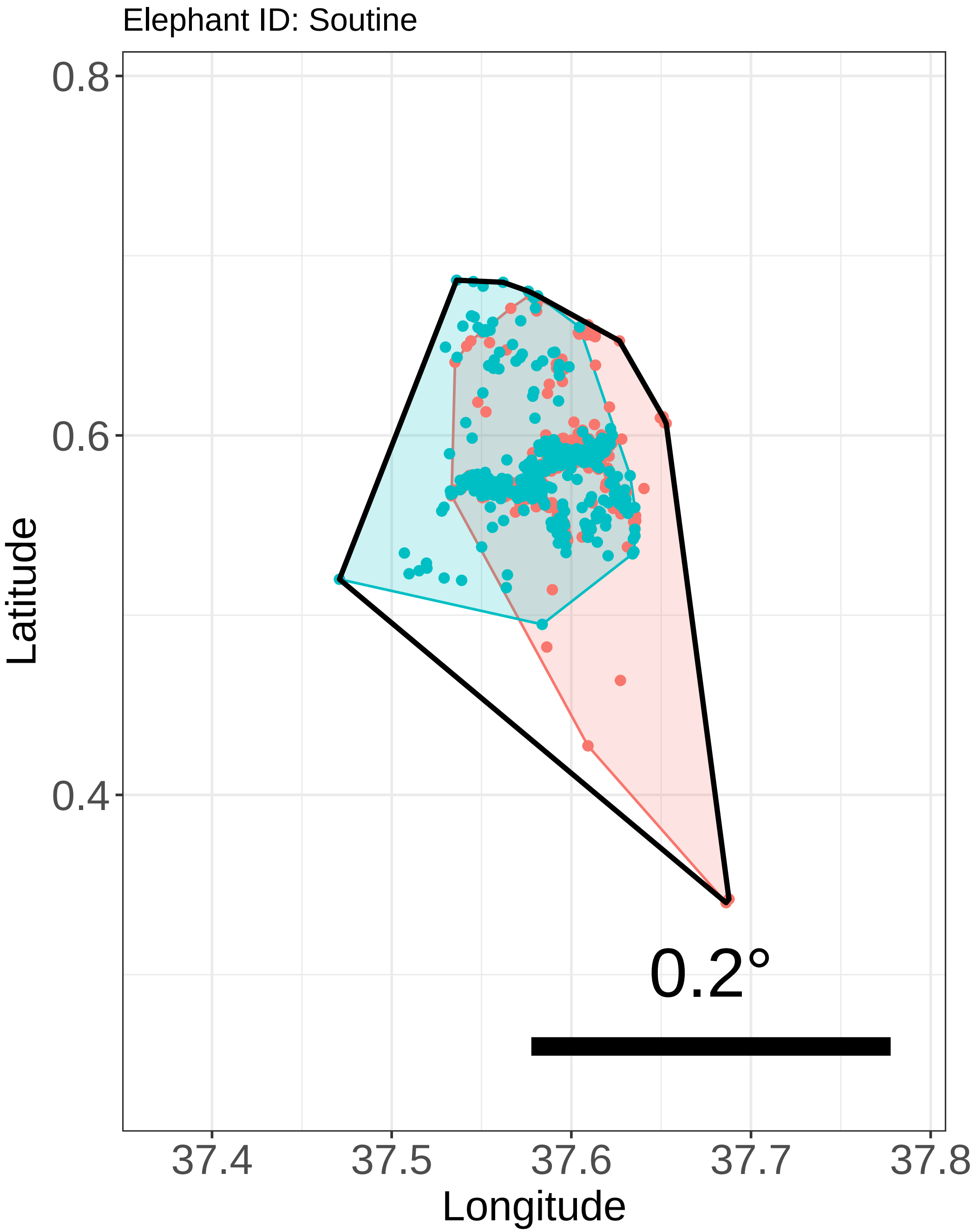

Elephant ID: Soutine
0.8
0.6
0.4
Latitude
0.2°
37.4
37.5
37.6
37.7
37.8
Longitude

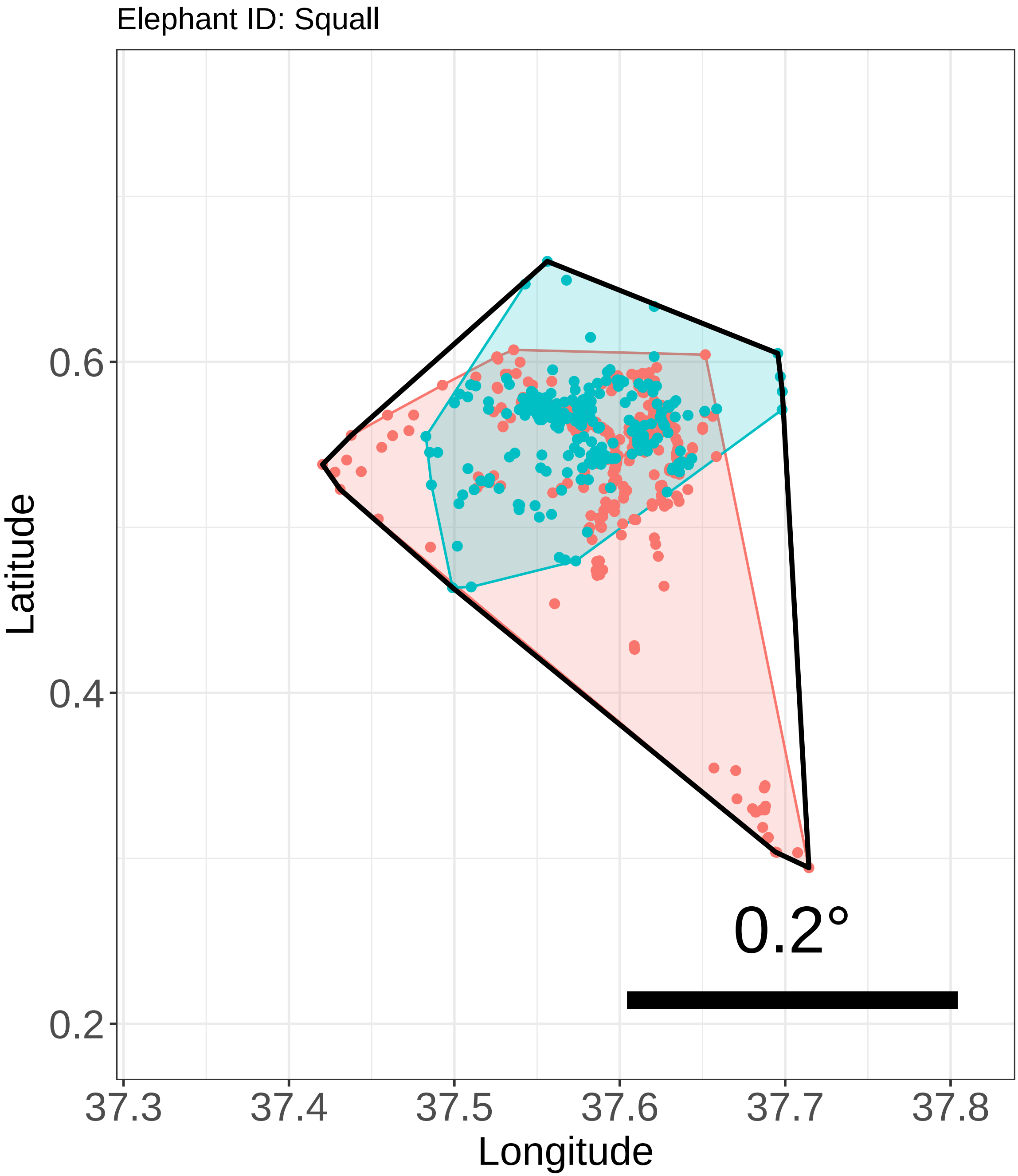

Elephant ID: Squall
0.2°
Latitude
0.6
0.4
0.2
Longitude
37.3
37.4
37.5
37.6
37.7
37.8

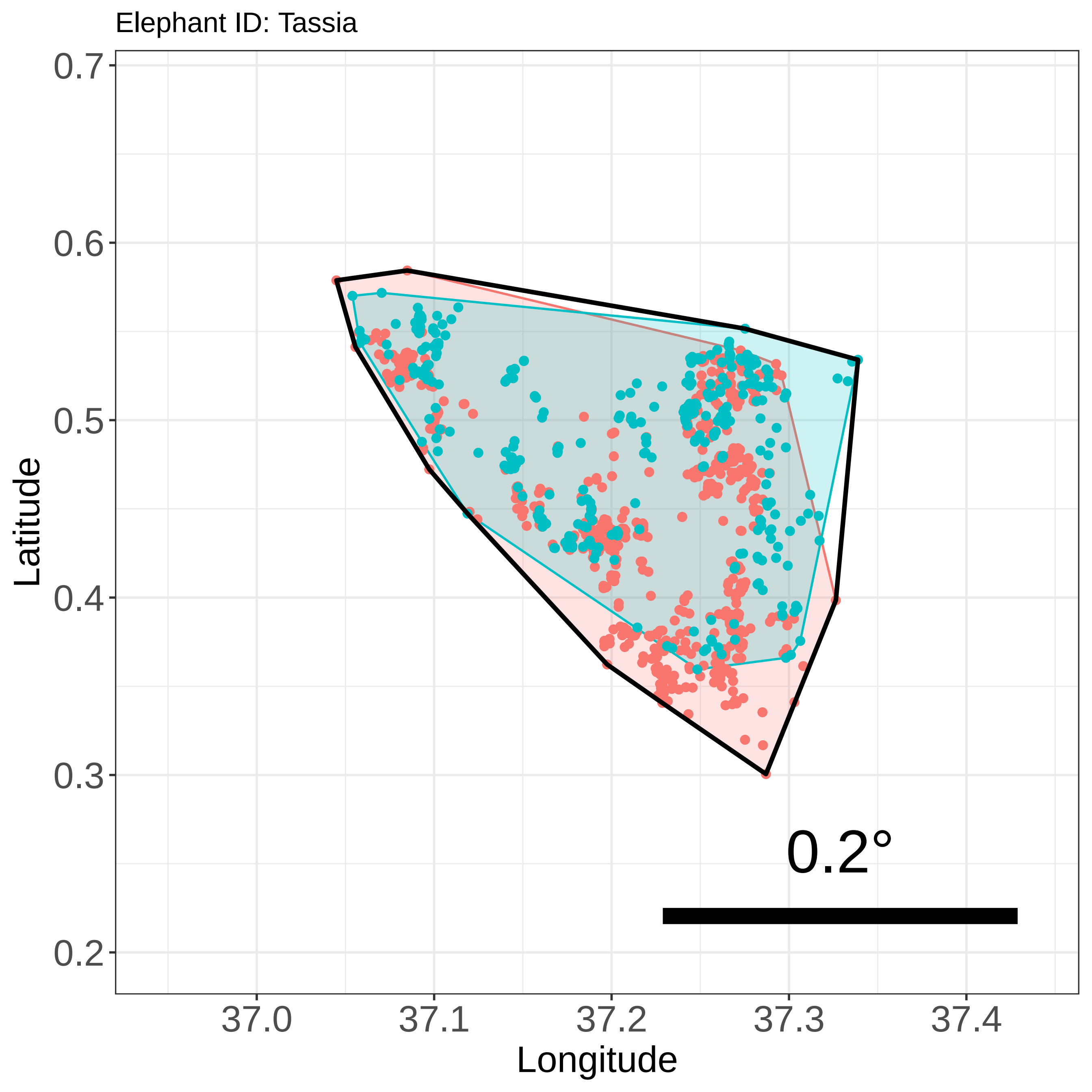
Elephant ID: Tassia
Latitude
0.7
0.6
0.5
0.4
0.3
0.2
Longitude
37.0
37.1
37.2
37.3
37.4
0.2°

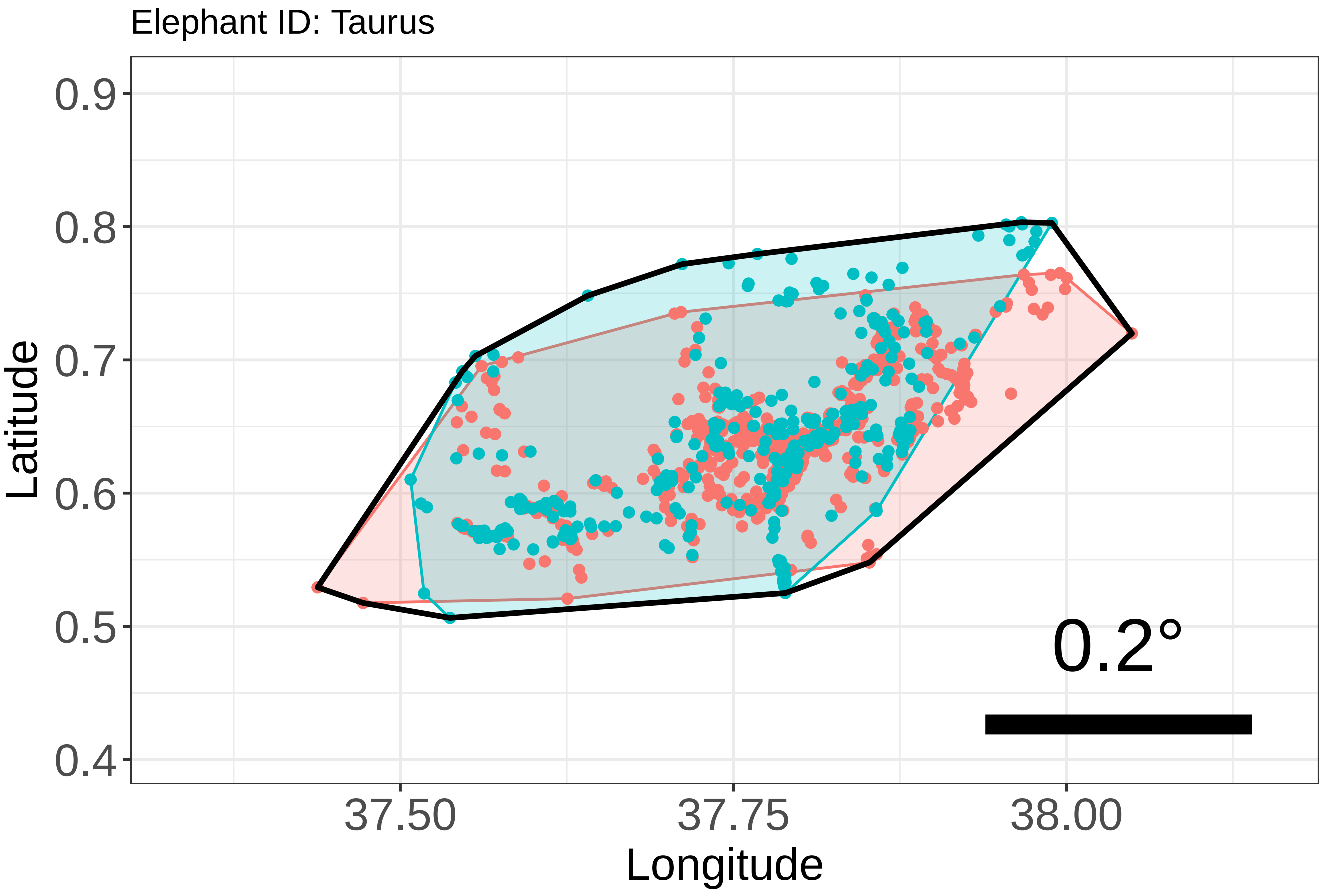

Elephant ID: Taurus
0.9
0.8
0.7
0.6
0.5
0.4
Latitude
37.50
37.75
38.00
Longitude
0.2°

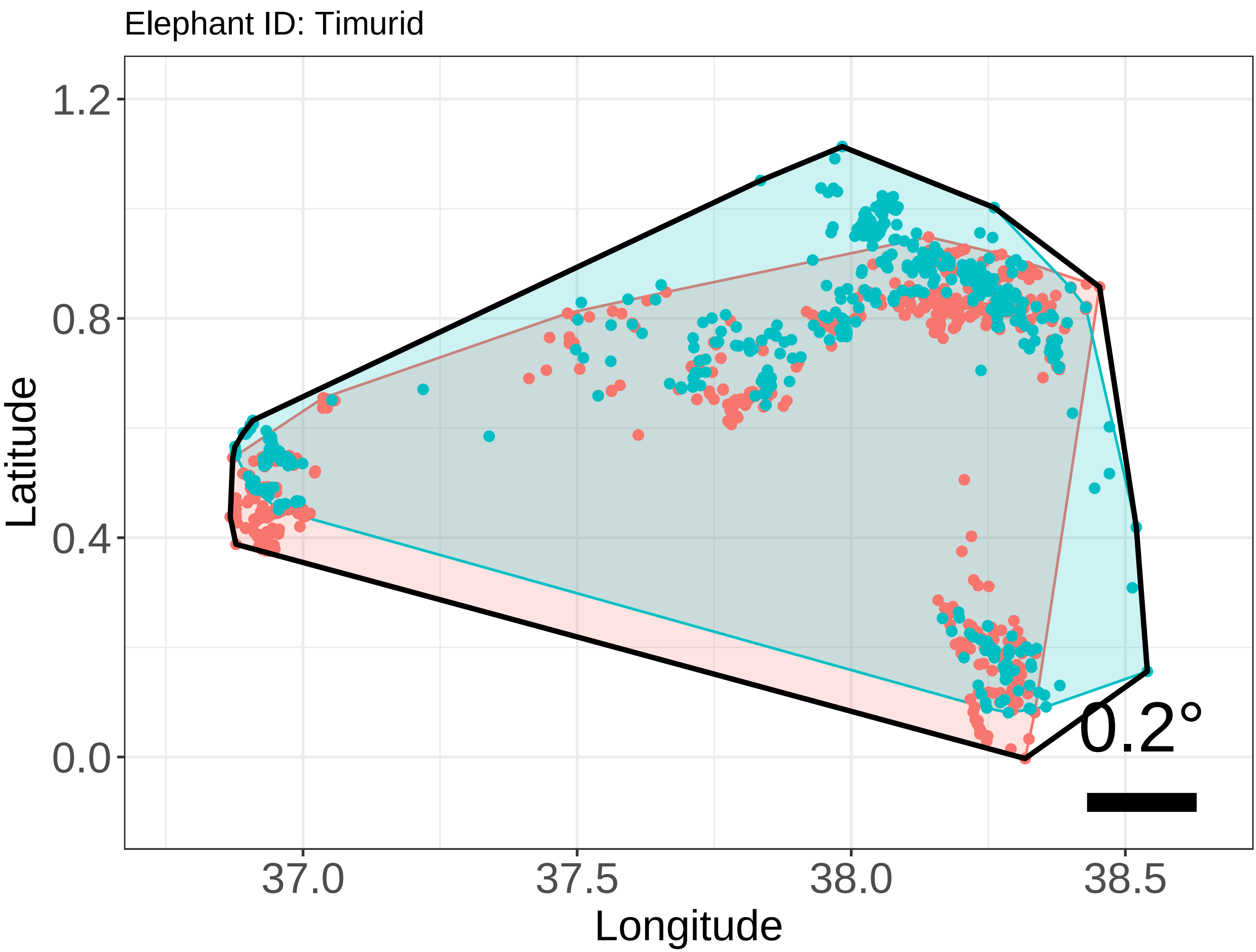
Elephant ID: Timurid
1.2
0.8
0.4
0.0
Latitude
37.0
37.5
38.0
38.5
Longitude
0.2°

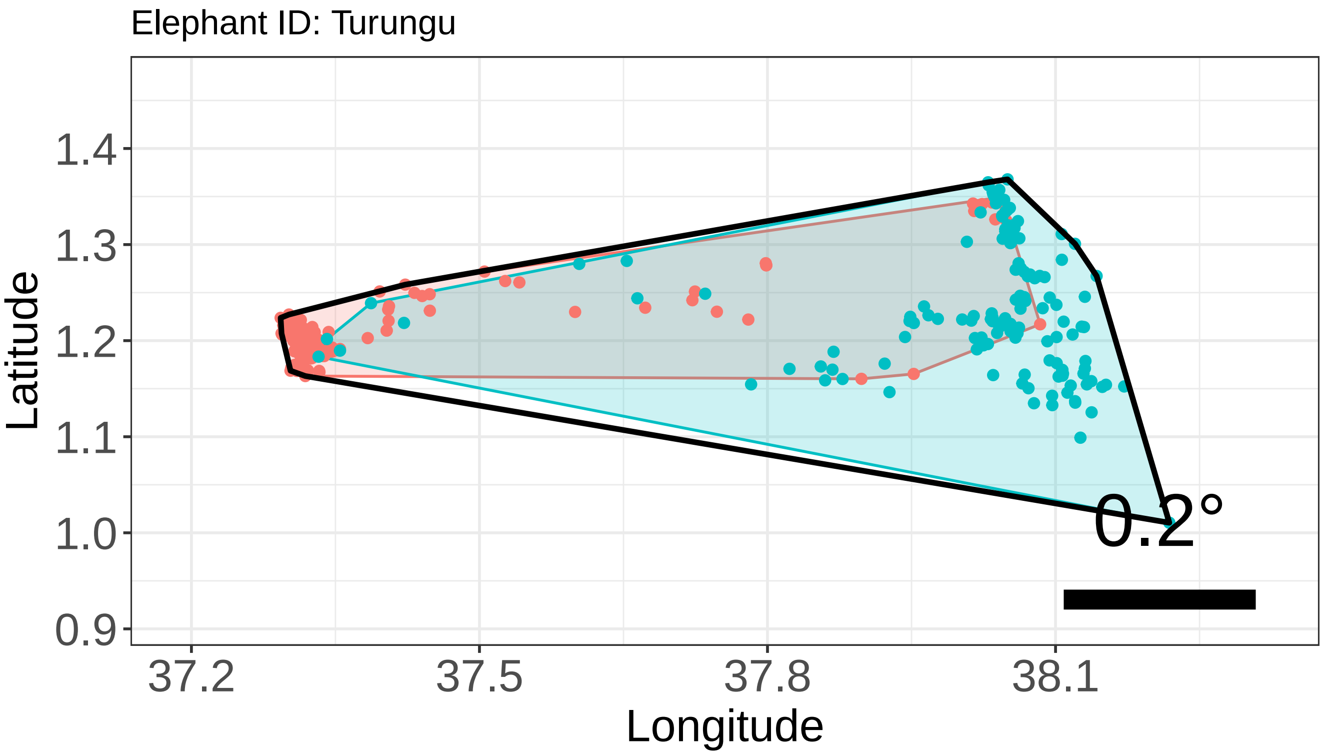

Elephant ID: Turungu
1.4
1.3
1.2
1.1
1.0
0.9
Latitude
37.2
37.5
37.8
38.1
Longitude
0.2°

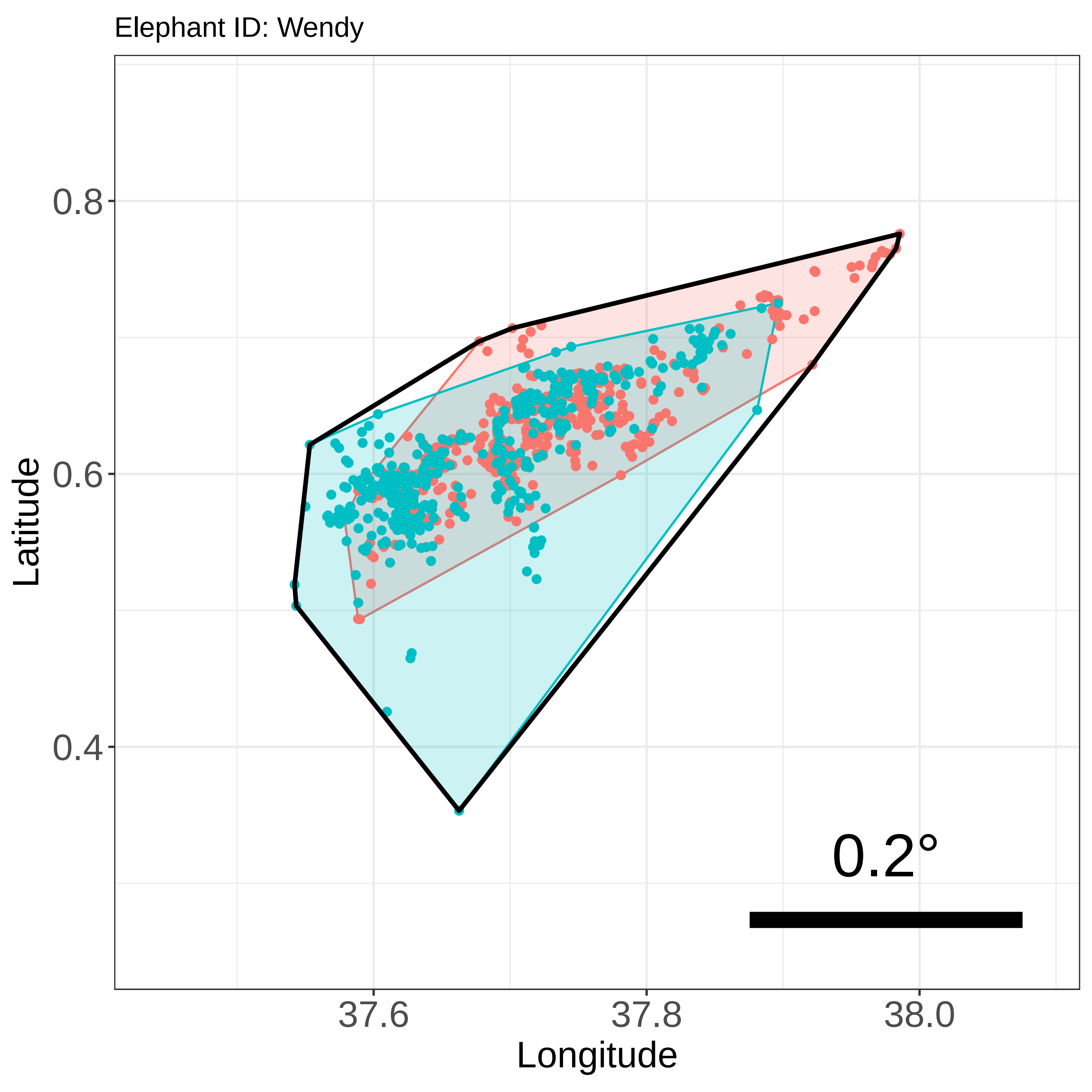

Elephant ID: Wendy
0.8
0.6
0.4
Latitude
37.6
37.8
38.0
Longitude
0.2°

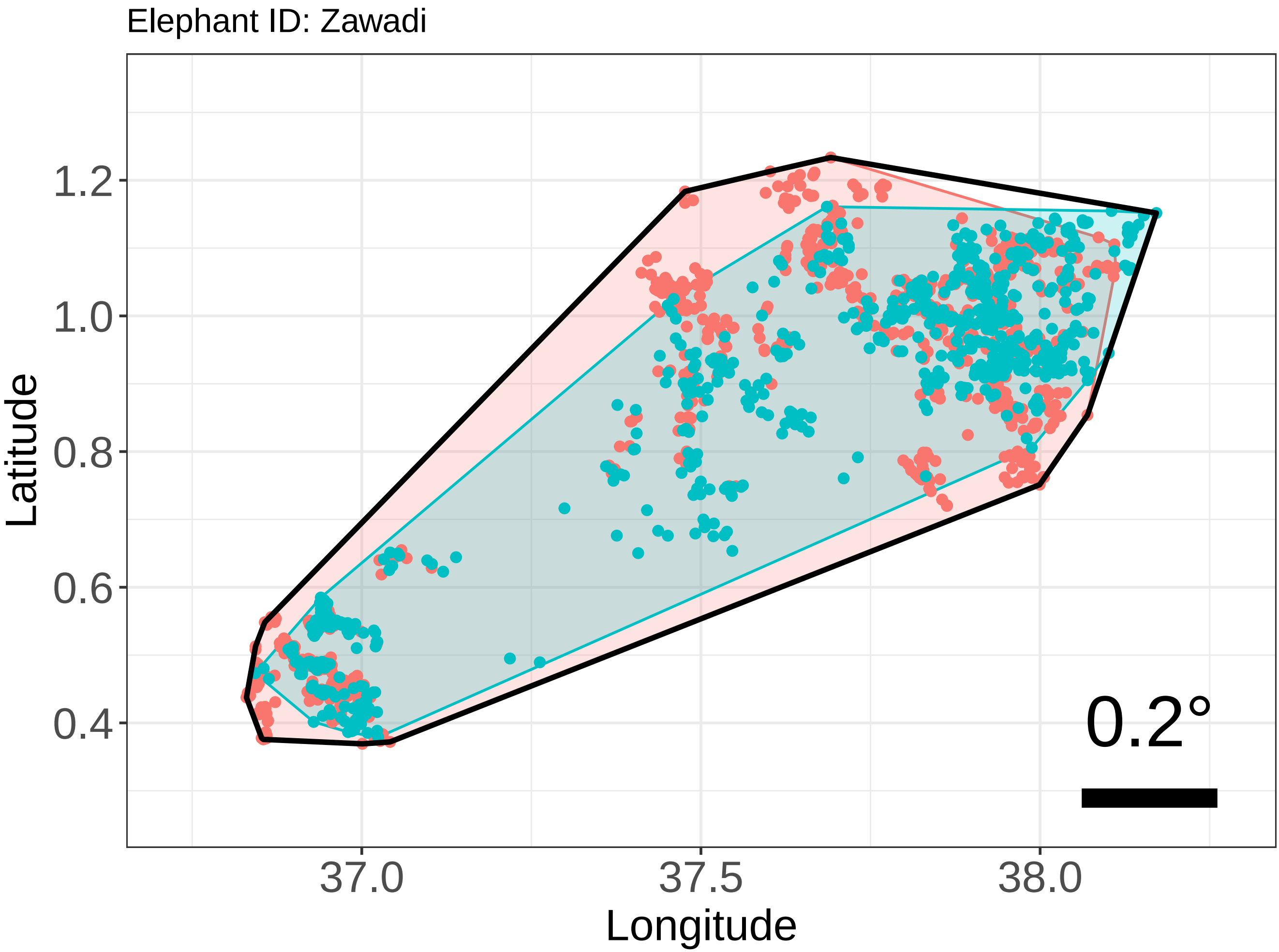

Elephant ID: Zawadi
1.2
1.0
0.8
0.6
0.4
Latitude
37.0
37.5
38.0
Longitude
0.2°
