# Supplementary figures and images for: Local rainfall is more likely than distant thunderstorms to affect movement behaviour in Northern Kenyan elephants
Source: PLoS One. 2024 Dec 23;19(12):e0307520. doi: 10.1371/journal.pone.0307520 (PMC11666045; doi:10.1371/journal.pone.0307520)

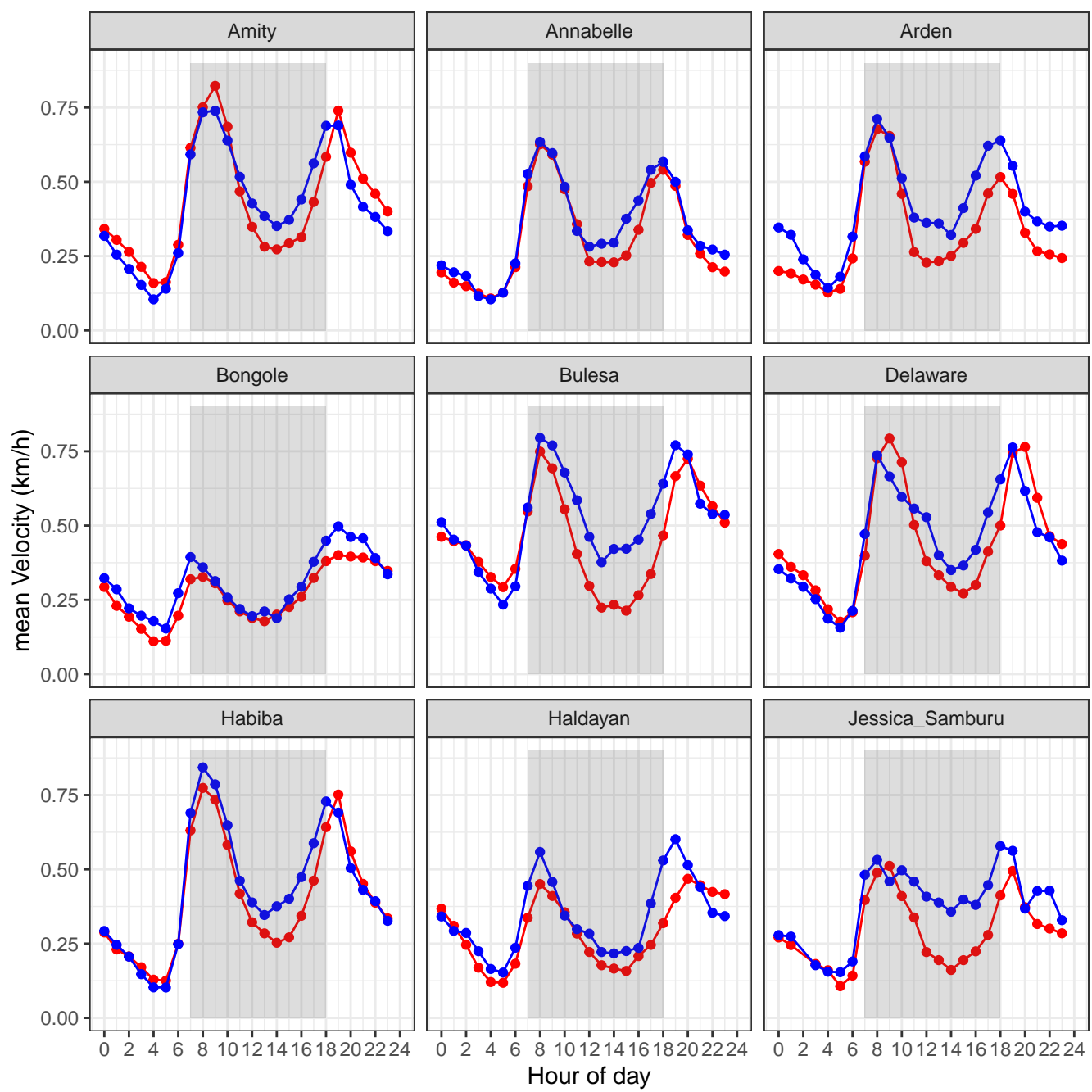

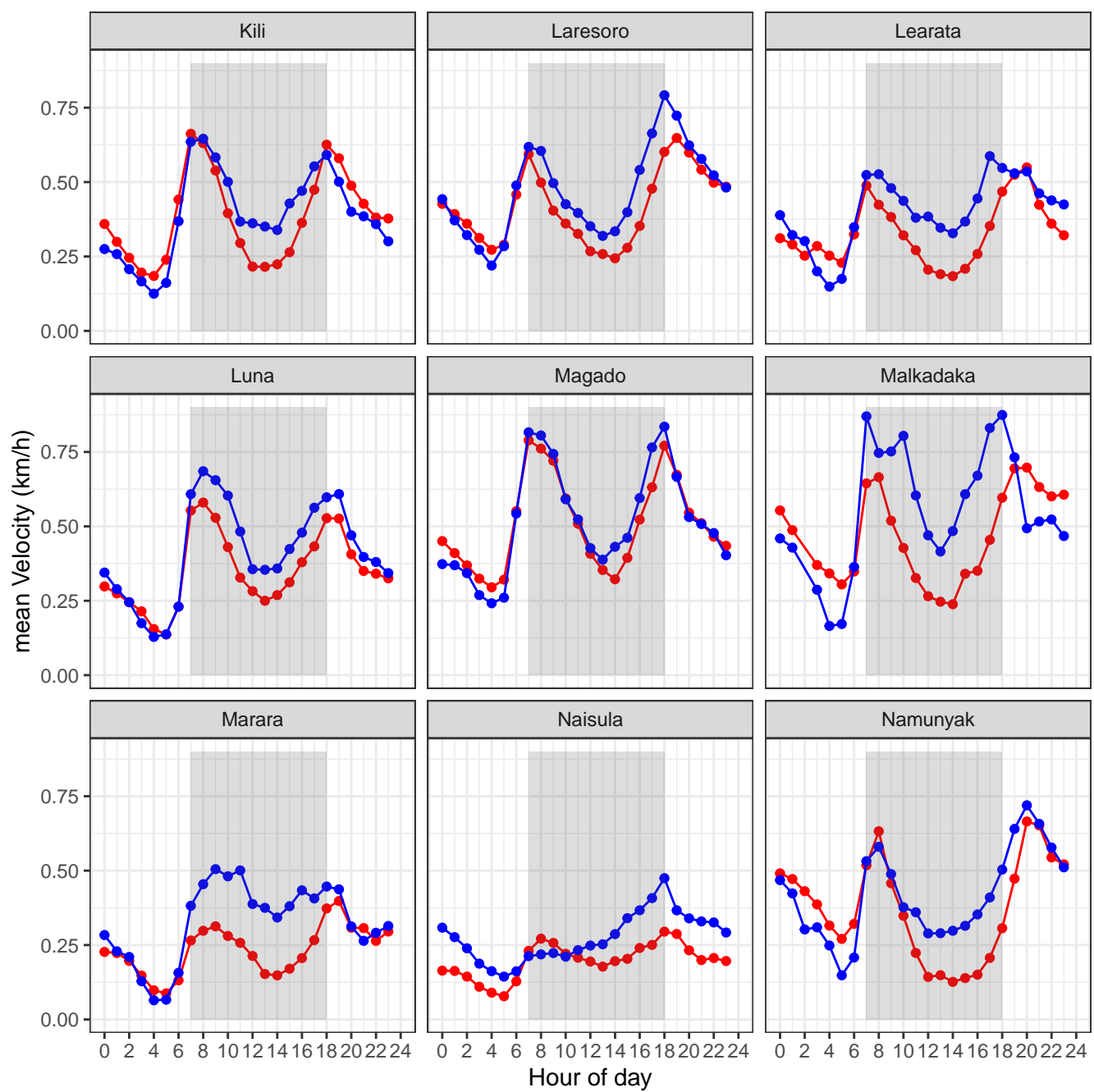

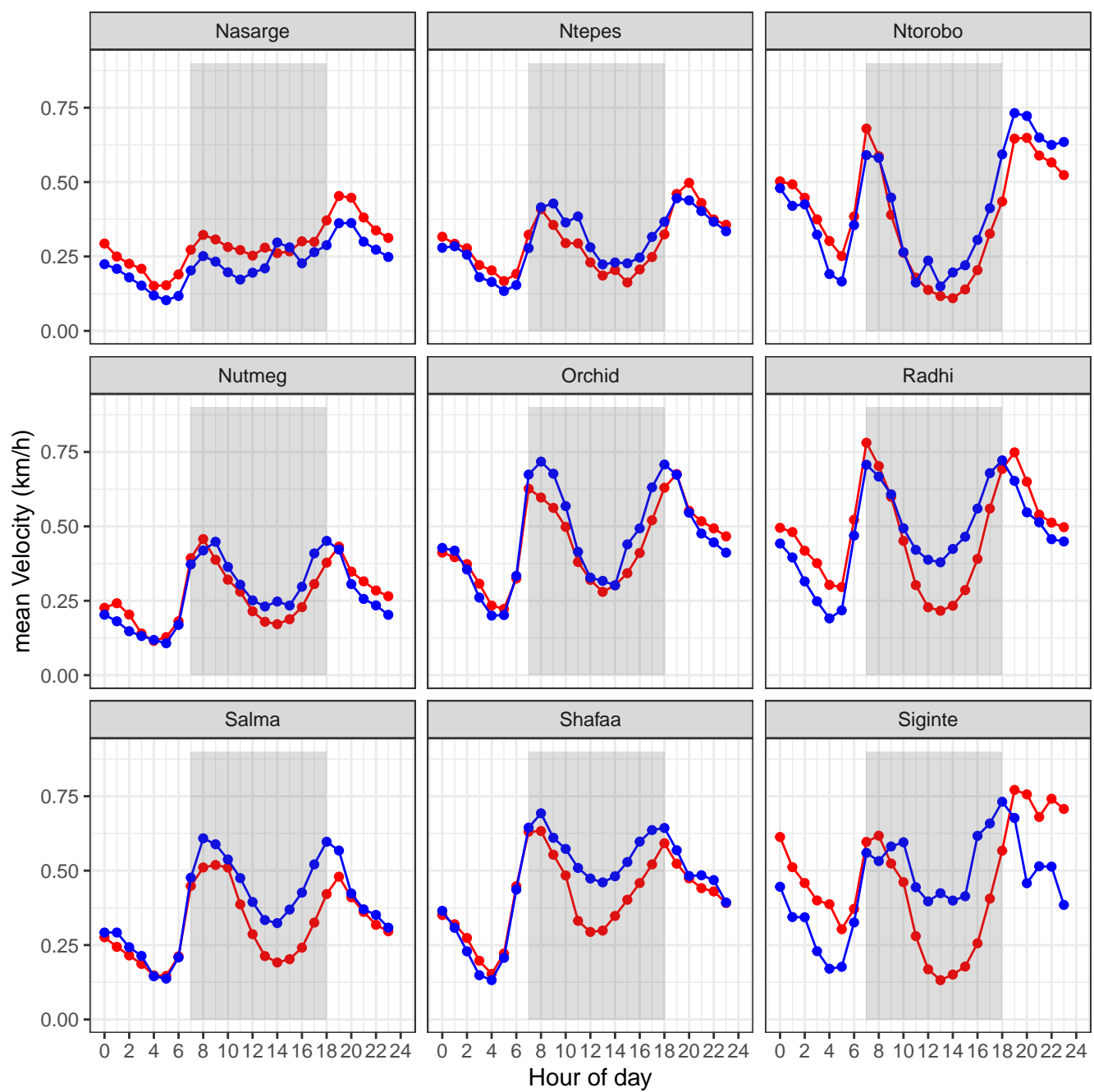

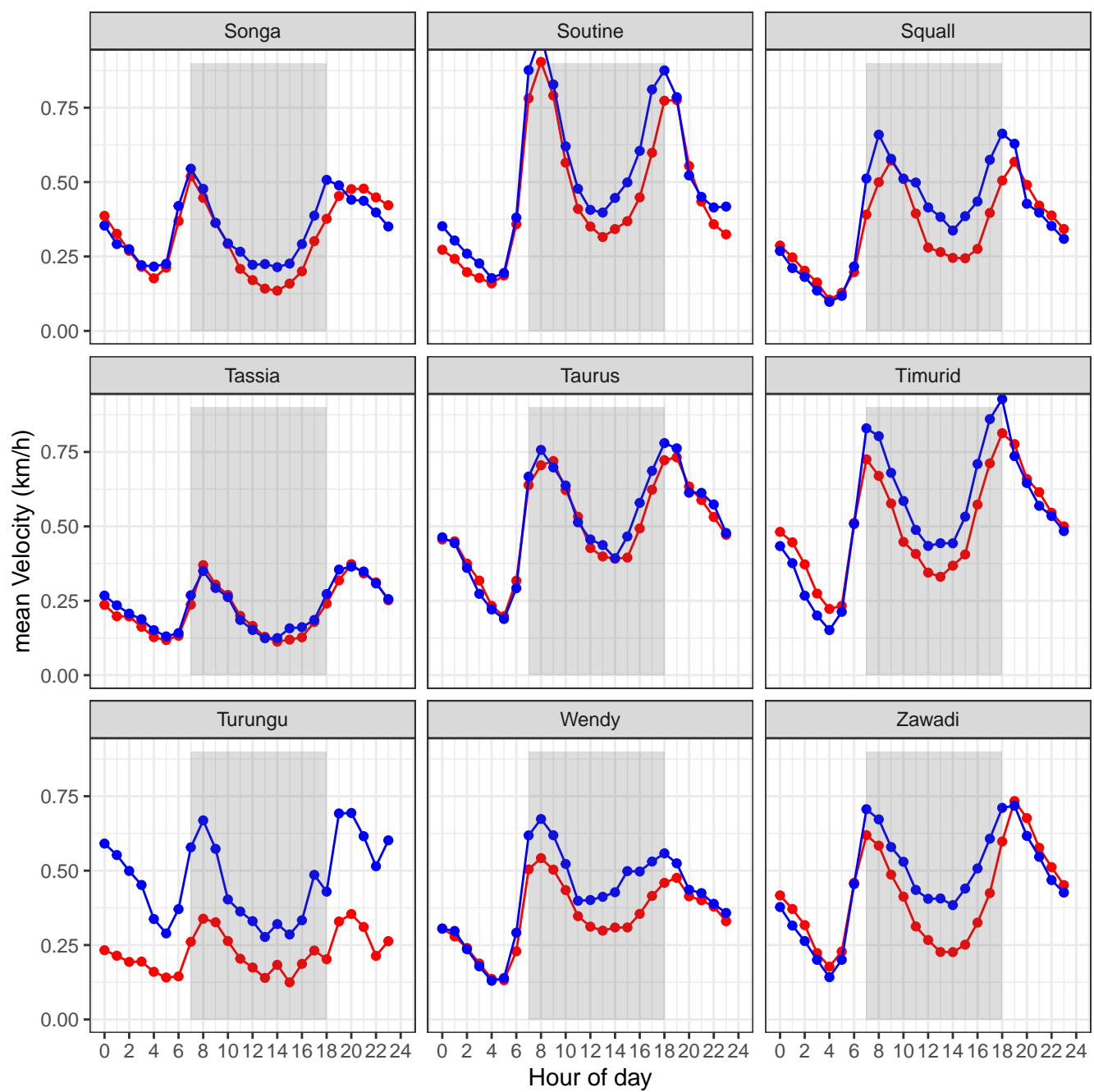

Supplement: S6 File — Blue indicates wet period velocity, and red indicates dry period velocity. Gray block indicates nighttime hours. (PDF) [file pone.0307520.s006.pdf]

### Delaware

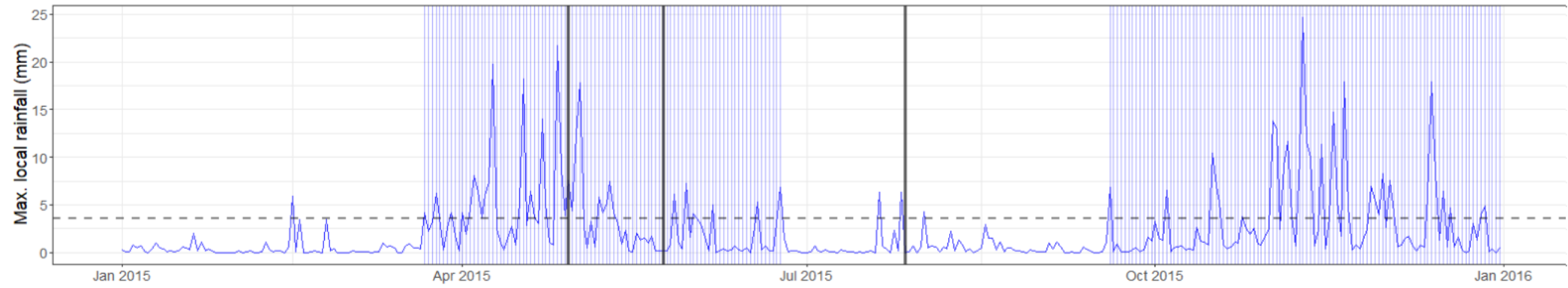

### Delaware

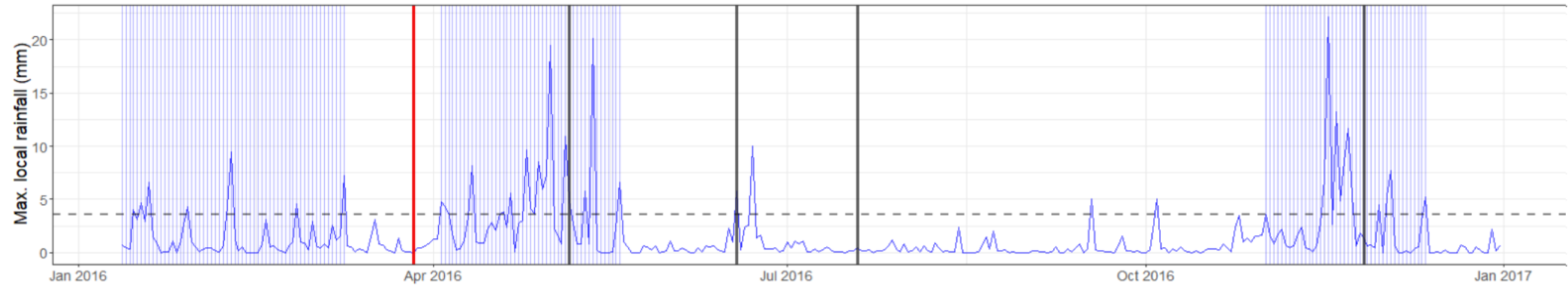

### Delaware

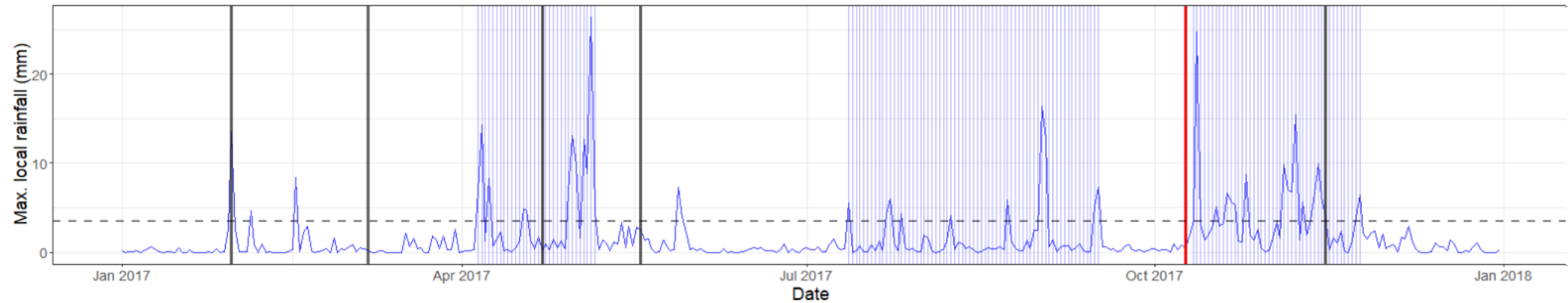

Delaware

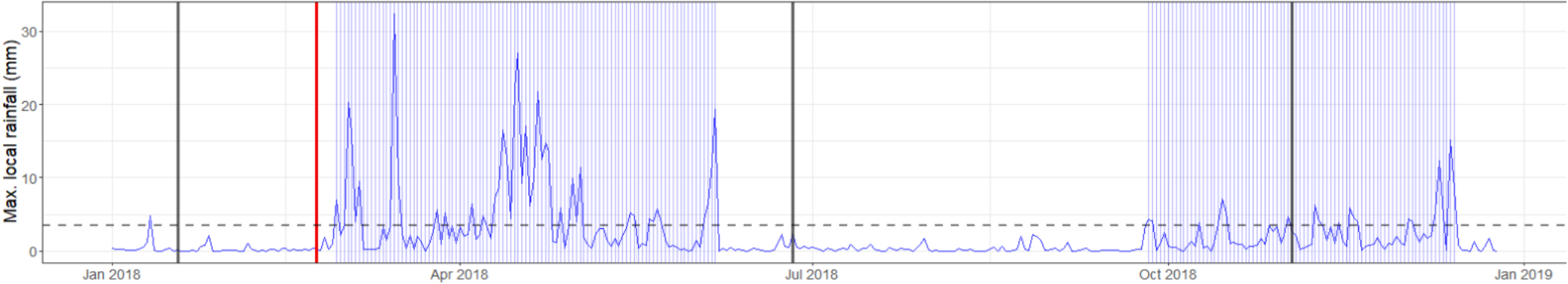

Laresoro

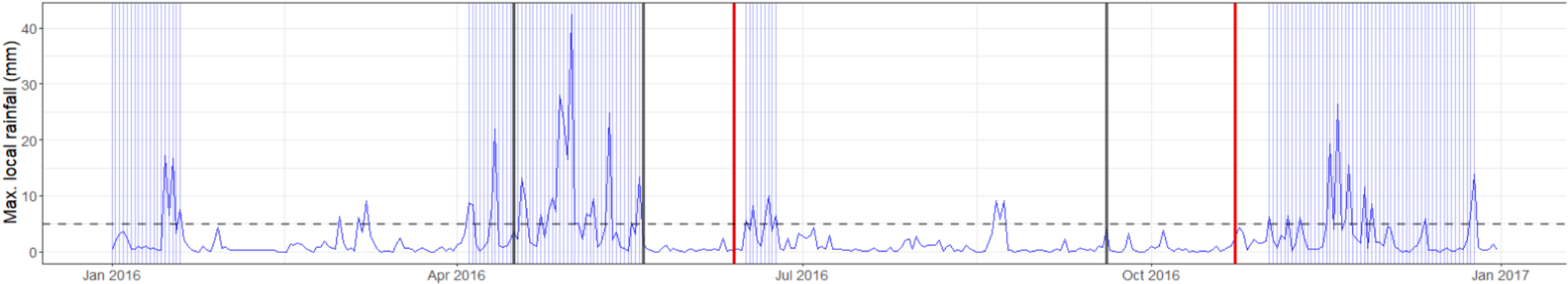

Laresoro

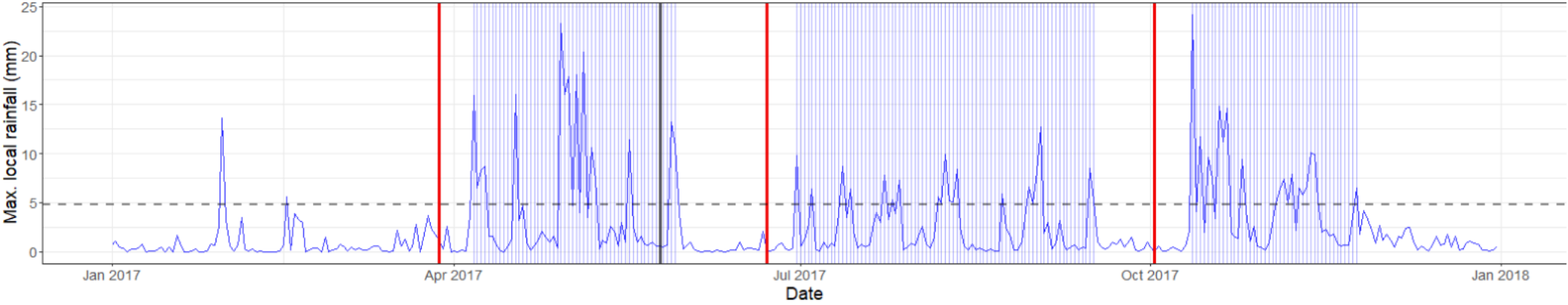

# Laresoro

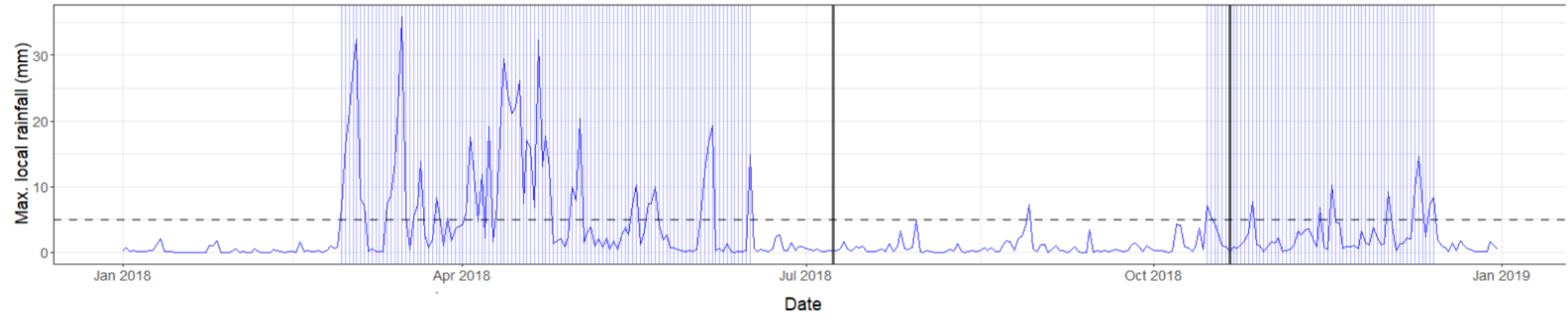

Supplement: S8 File — Blue blocks indicate wet periods. Red vertical lines indicate intra-window dry period BCPDs. Gray vertical lines indicate extra-window BCPDs. Blue line indicates daily rainfall amount. Horizontal dashed line indicates rainfall volume criterion for a day to be denoted as ‘rainy’. (PDF) [file pone.0307520.s008.pdf]

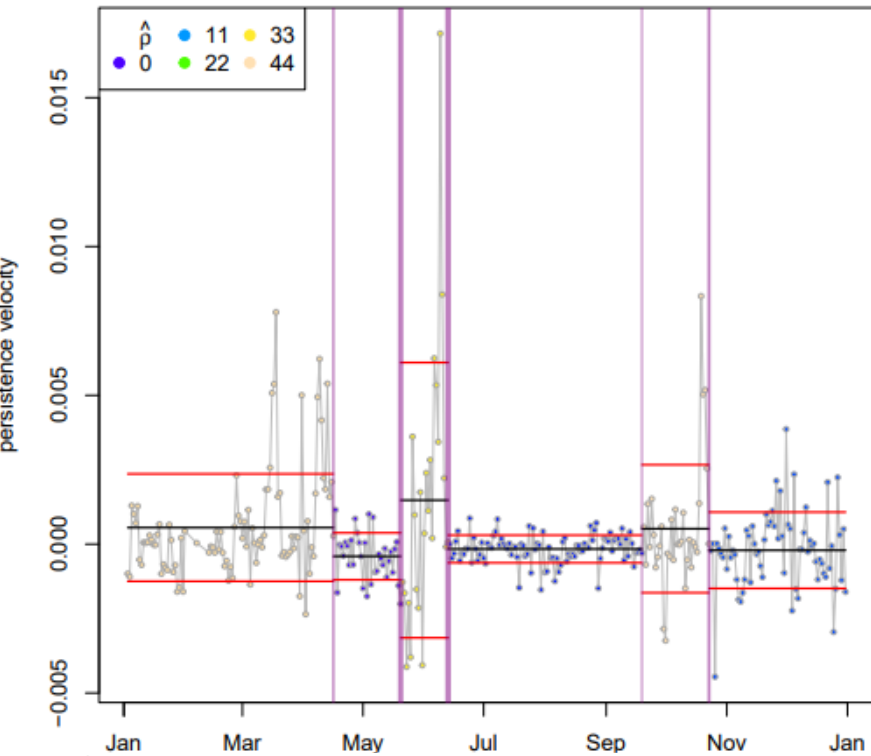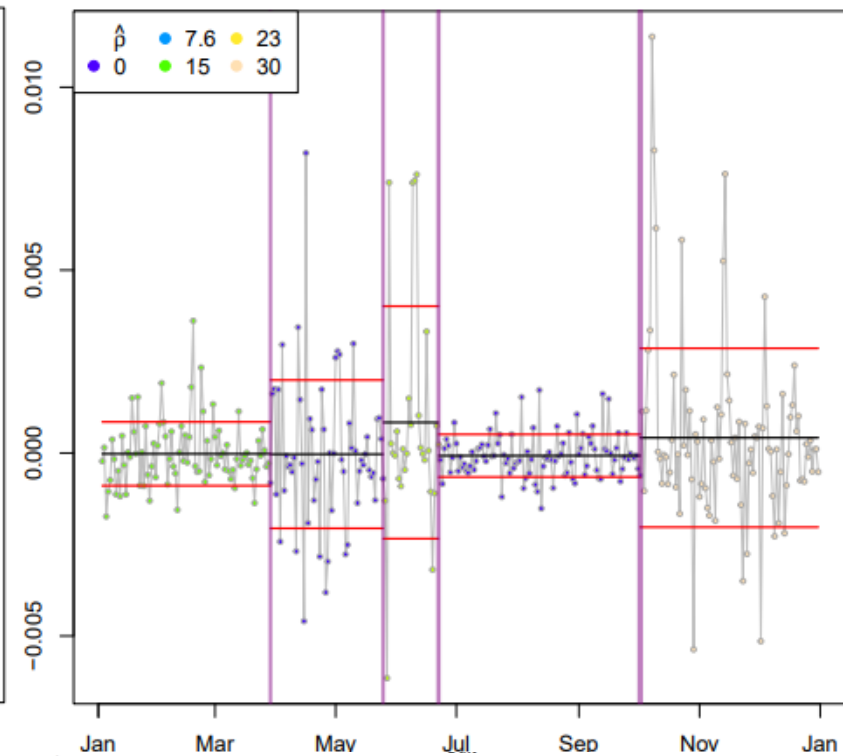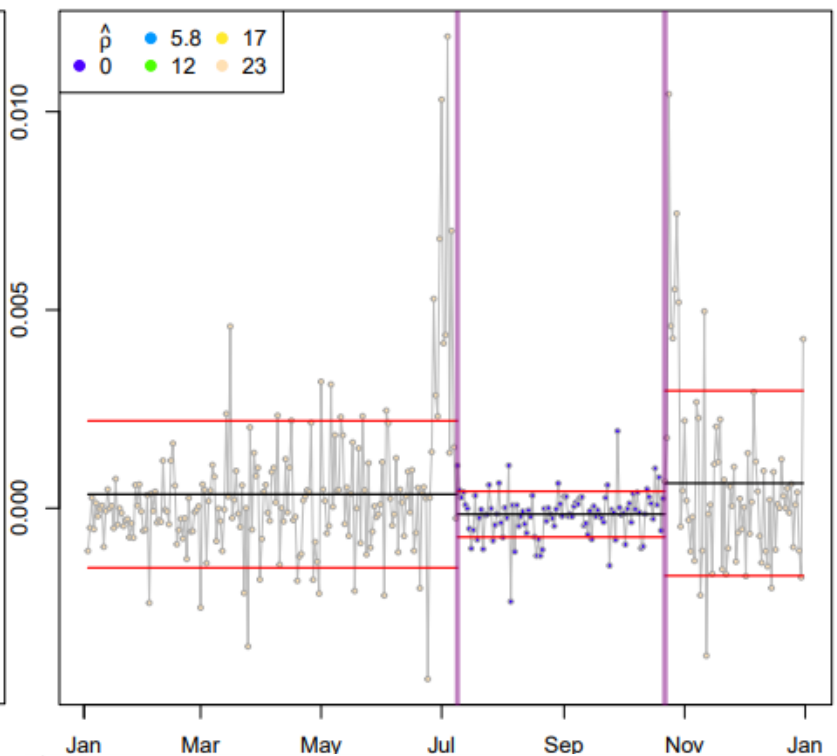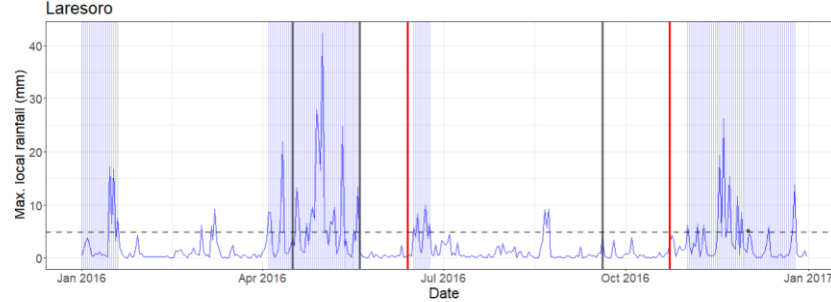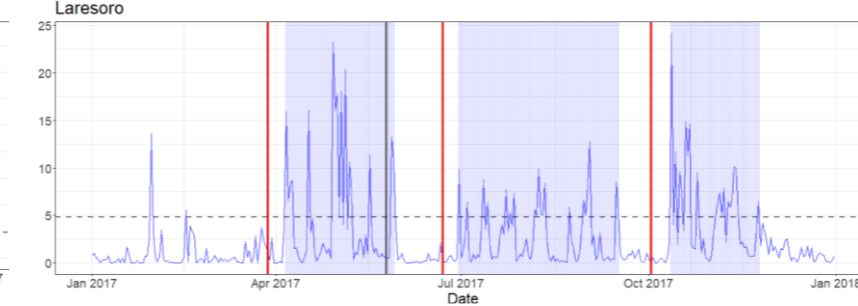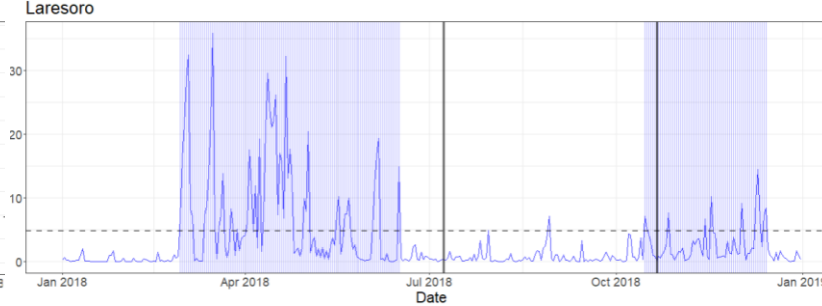

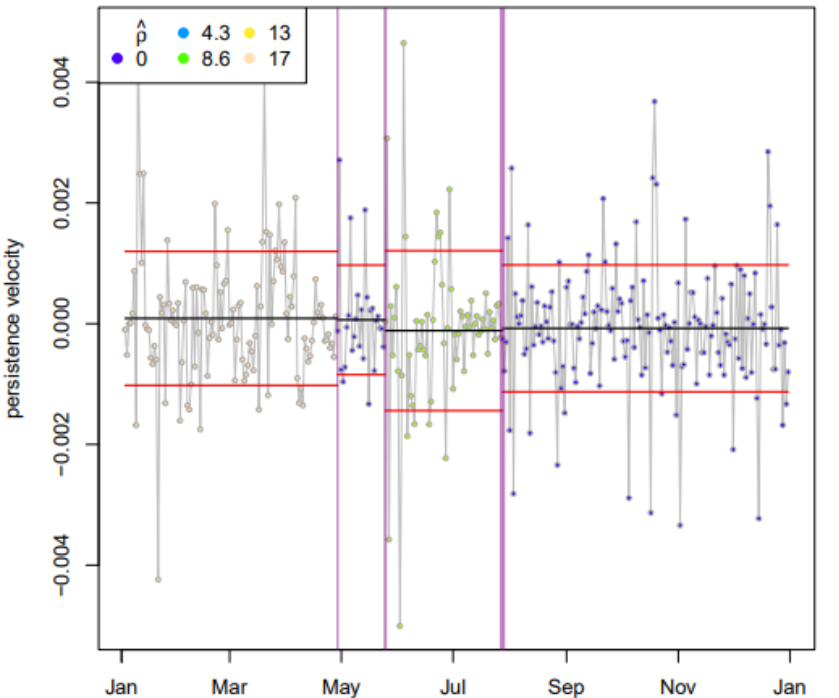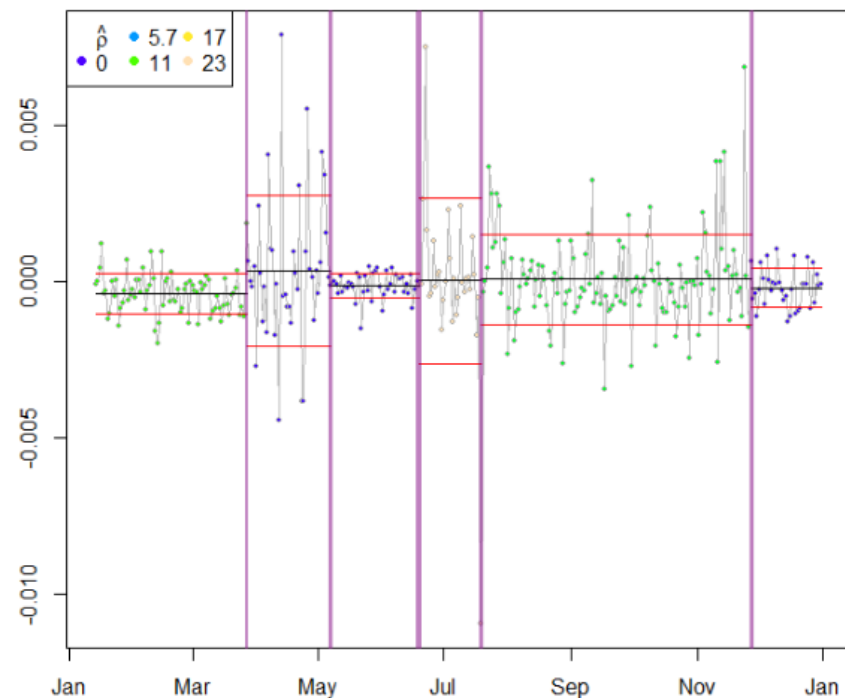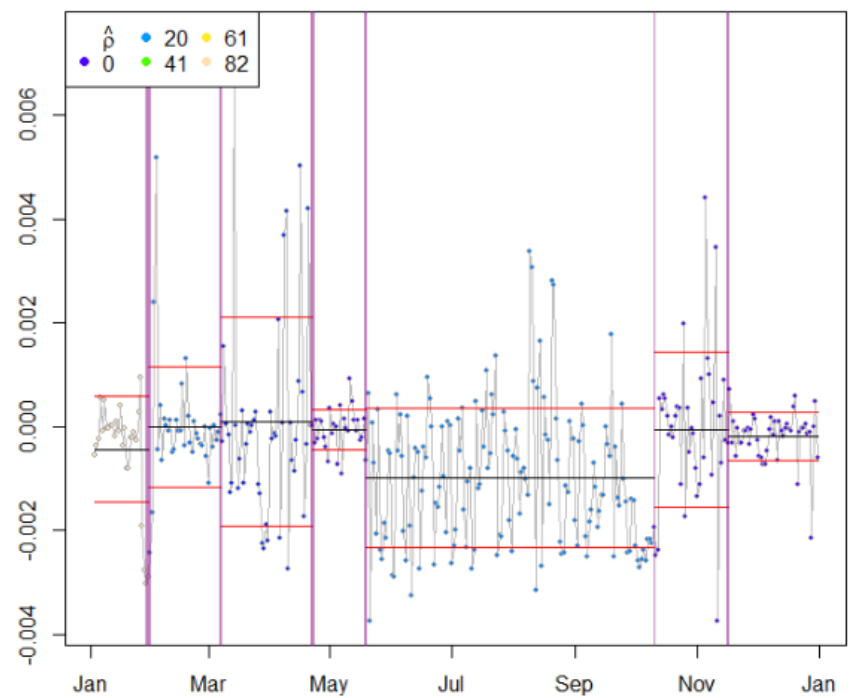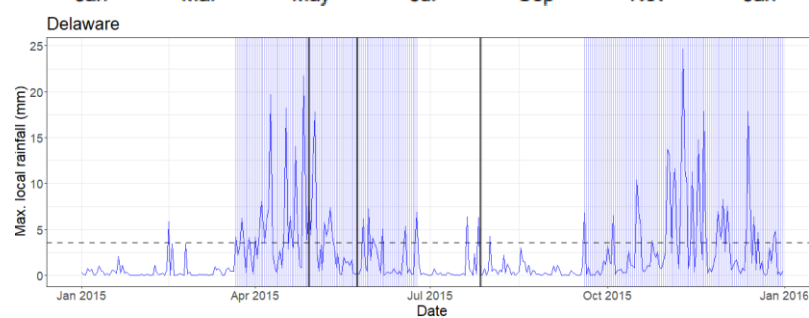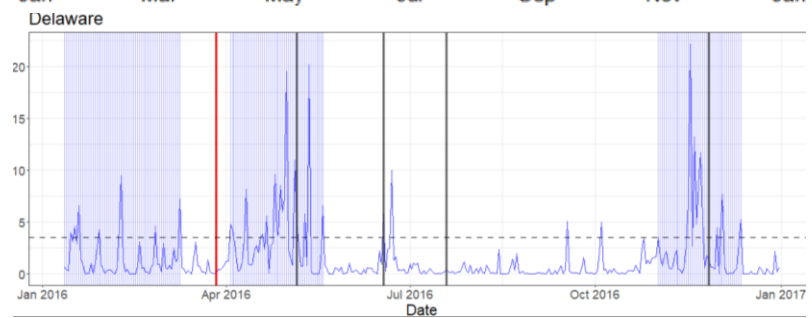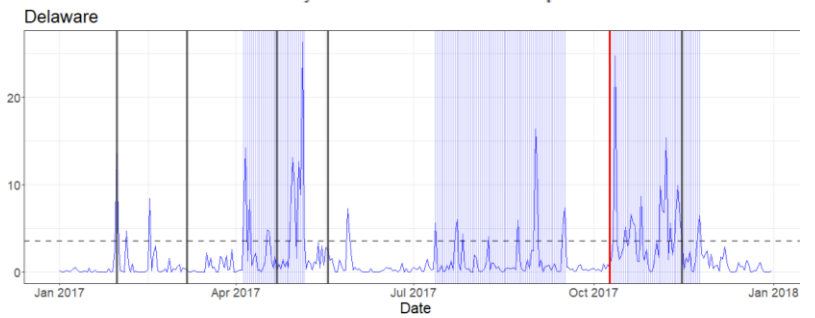

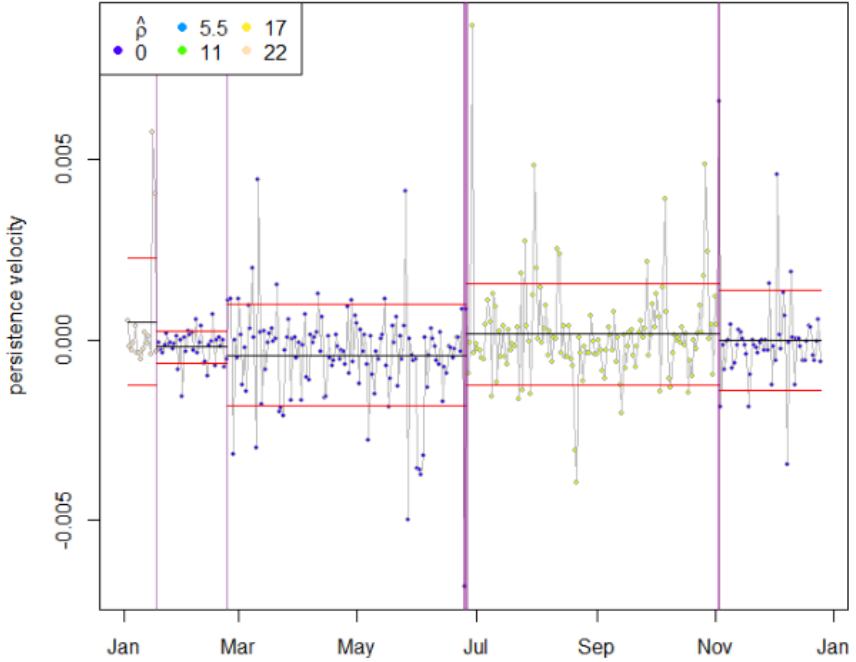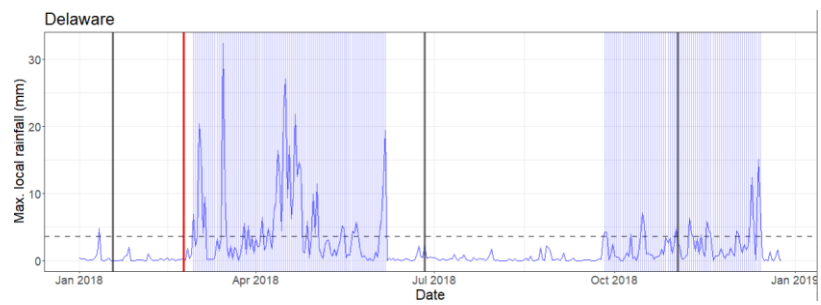

Supplement: S10 File — Upper plots on each page indicate the annual changes in persistence velocity for each elephant. The colours of points indicate the respective autocorrelation value. The lower plots indicate the associated local rainfall, with red vertical lines indicating intra-window BCPDs and gray vertical lines indicating extra-window BCPDs. (PDF) [file pone.0307520.s010.pdf]
