# Supplementary material for: Local rainfall is more likely than distant thunderstorms to affect movement behaviour in Northern Kenyan elephants
Source: PLoS One. 2024 Dec 23;19(12):e0307520. doi: 10.1371/journal.pone.0307520 (PMC11666045; doi:10.1371/journal.pone.0307520)
Supplement: S7 File — Range sizes in km2. (PDF) [file pone.0307520.s007.pdf]

	Elephant ID	RHR (km^2)	Total hull (km^2)	Dry hull (km^2)	Dry hull (km^2)	Hull coverage^ (%)
1	Amity	3711.573	775.6936	711.7288	651.6009	20.89932
2	Annabelle	4024.938	994.6929	803.6159	747.9272	24.71325
3	Arden	3186.997	745.8175	323.1502	680.1498	23.40189
4	Bongole	3025.029	662.0636	630.3303	431.9639	21.88619
5	Bulesa	11112.62	3699.035	3057.612	2551.63	33.28678
6	Delaware	4467.947	1072.559	796.0045	1053.404	24.00563
7	Habiba	5189.679	1348.77	1217.286	846.9154	25.98946
8	Haldayan	2423.063	470.3341	407.6016	258.3257	19.41072
9	JessicaSamburu	5881.886	1582.145	1260.59	994.4456	26.8986
10	Kili	6061.84	2105.534	2042.118	1533.156	34.73423
11	Laresoro	23957.36	8361.773	6826.232	7277.707	34.90274
12	Learata	6905.326	2240.664	1839.084	1843.54	32.44834
13	Luna	9569.77	4042.782	3281.797	2016.357	42.24535
14	Magado	29072.34	11666.3	8313.897	9443.793	40.12852
15	Malkadaka	10759.76	2978.472	2685.715	1180.639	27.68158
16	Marara	2824.314	656.865	451.8443	510.0391	23.25751
17	Naisula	10980.92	3236.89	3184.999	1366.447	29.47741
18	Namunyak	2964.343	603.5634	489.7436	316.7617	20.36078
19	Nasarge	4785.806	1468.042	1051.187	1247.102	30.67492
20	Ntepes	6381.715	2145.613	1689.361	2111.844	33.62125
21	Ntorobo	15922.11	4739.919	4739.919	847.7994	29.76942
22	Nutmeg	7276.34	2689.252	1595.999	1890.844	36.95886
23	Orchid	13667.57	5952.977	5036.813	4511.906	43.55551
24	Radhi	23442.9	9635.574	9390.43	8958.979	41.10232
25	Salma	6594.874	2459.223	1724.081	1988.584	37.28992
26	Shafaa	11630.85	4399.449	2945.534	2712.074	37.82568
27	Siginte	5940.82	2202.291	2093.93	689.5406	37.07049
28	Songa	2632.456	550.4868	522.5231	369.8408	20.91153
29	Soutine	2805.593	497.1676	333.9522	257.3788	17.72059
30	Squall	3447.282	718.5428	548.4858	318.3728	20.84375
31	Tassia	2944.707	588.9468	528.6151	473.6941	20.00019
32	Taurus	4967.68	1393.865	1143.801	1123.389	28.05866
33	Timurid	30376.91	14516.38	11727.24	12817.45	47.78754
34	Turungu	7730.312	2287.539	1227.424	2067.399	29.59181
35	Wendy	4936.78	1032.962	538.5769	845.9357	20.92379
36	Zawadi	20213.3	7472.038	7238.787	5675.81	36.96595

^ Percentage of RHR covered by Total hull.
