# Supplementary material for: Local rainfall is more likely than distant thunderstorms to affect movement behaviour in Northern Kenyan elephants
Source: PLoS One. 2024 Dec 23;19(12):e0307520. doi: 10.1371/journal.pone.0307520 (PMC11666045; doi:10.1371/journal.pone.0307520)
Supplement: S9 File — Four upper plots indicate the distance to lightning in each of the 4 quadrants (North, East, South, West) from the position of the focal elephant (up to 300 km), for each year. To facilitate easy relation to local rainfall, two copies of rainfall plots are also provided below these quadrants. Vertical red lines indicate an intra-window dry period BCPD. (PDF) [file pone.0307520.s009.pdf]

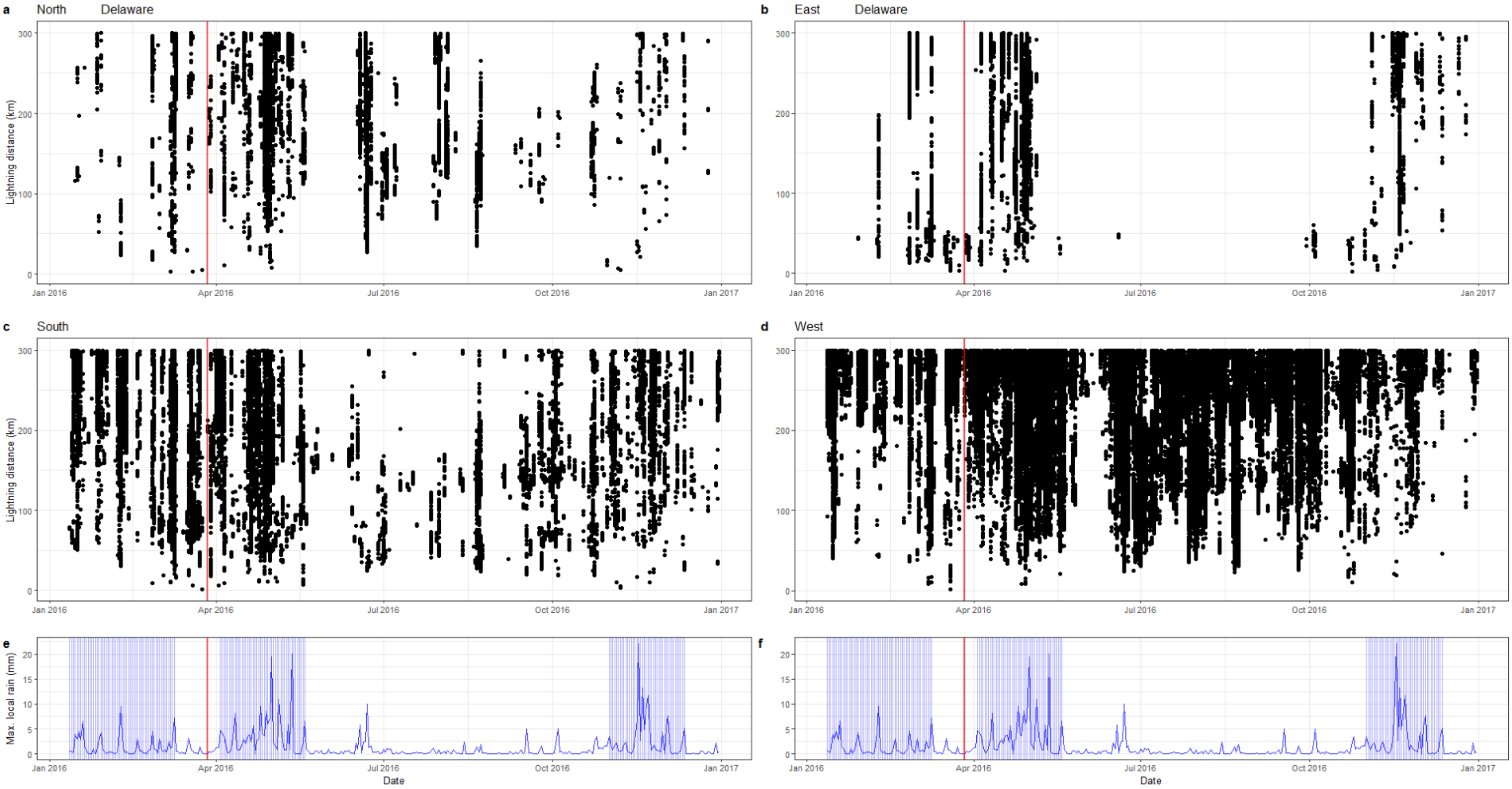

a
North
Delaware
b
East
Delaware
c
South
d
West
e
f
Lightning distance (km)
Max. local rain (mm)
0
100
200
300
0
5
10
15
20
Jan 2016
Apr 2016
Jul 2016
Oct 2016
Jan 2017
Date

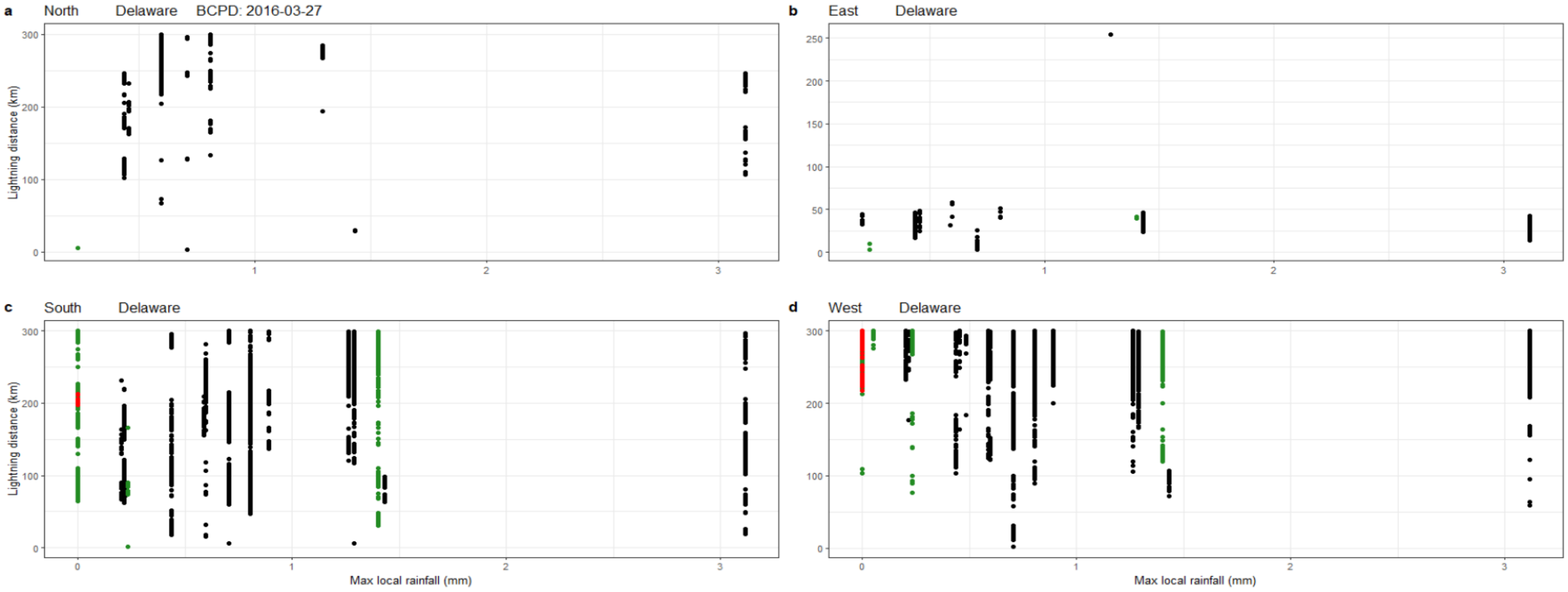
a
North
Delaware
BCPD: 2016-03-27
b
East
Delaware
c
South
Delaware
d
West
Delaware
Lightning distance (km)
Max local rainfall (mm)
300
200
100
0
250
150
50
1
2
3

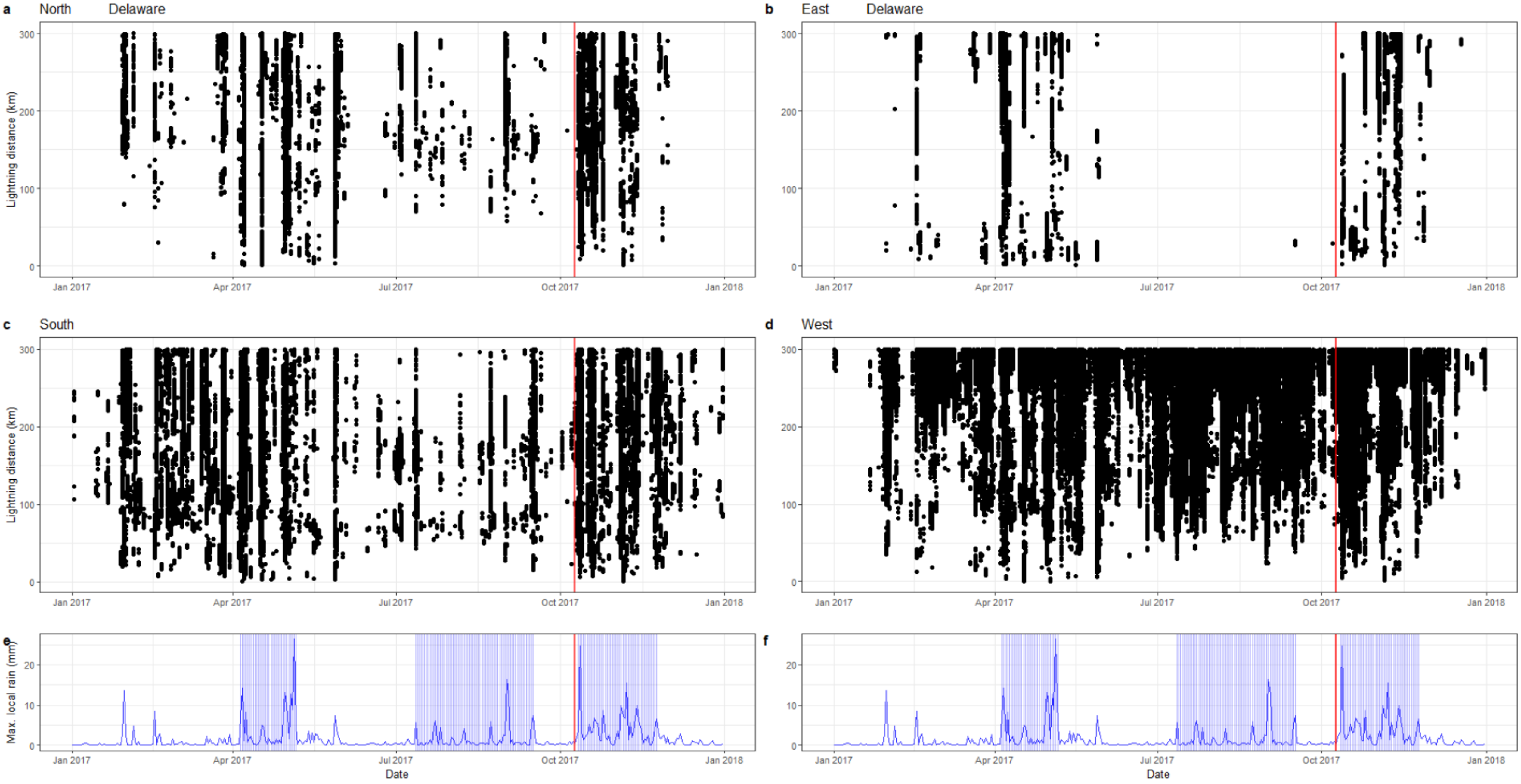

a
North
Delaware
b
East
Delaware
c
South
d
West
e
f
Lightning distance (km)
Max. local rain (mm)
Date
Jan 2017
Apr 2017
Jul 2017
Oct 2017
Jan 2018

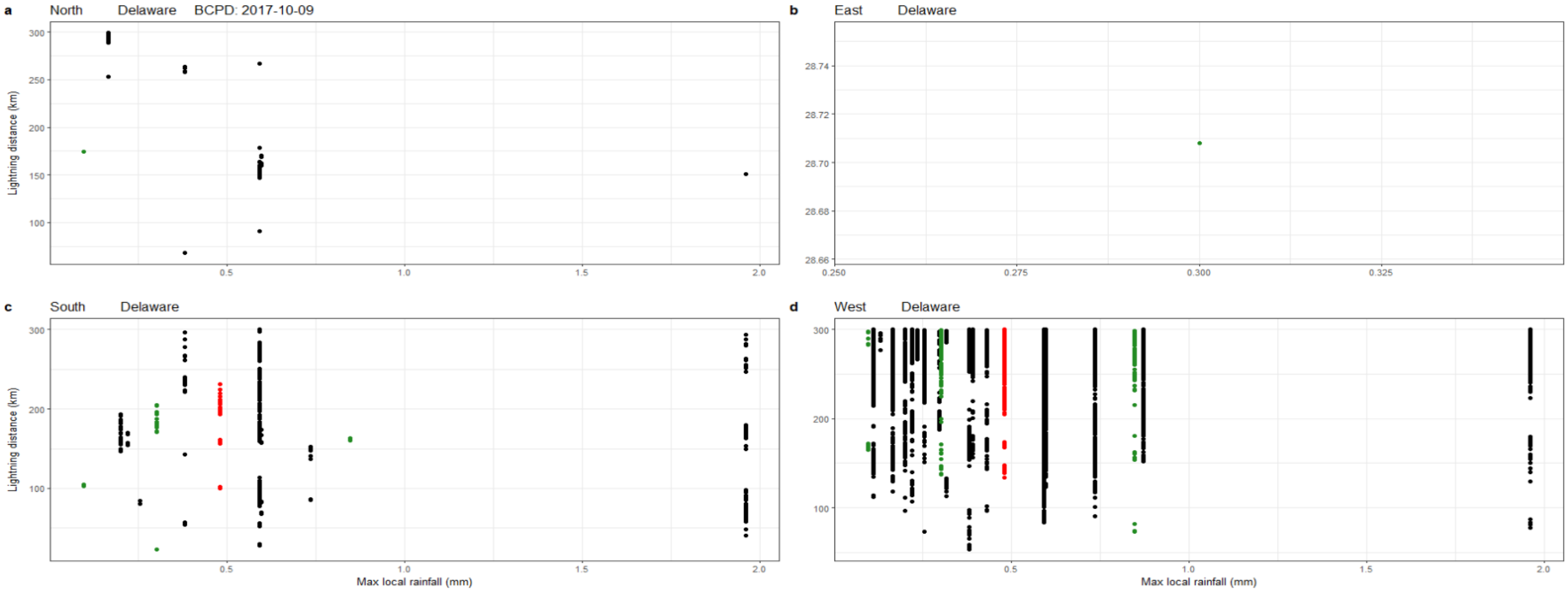
a
North
Delaware
BCPD: 2017-10-09
Lightning distance (km)
300
250
200
150
100
0.5
1.0
1.5
2.0
b
East
Delaware
28.74
28.72
28.70
28.68
28.66
0.250
0.275
0.300
0.325
c
South
Delaware
Lightning distance (km)
300
200
100
0.5
1.0
1.5
2.0
Max local rainfall (mm)
d
West
Delaware
300
200
100
0.5
1.0
1.5
2.0
Max local rainfall (mm)

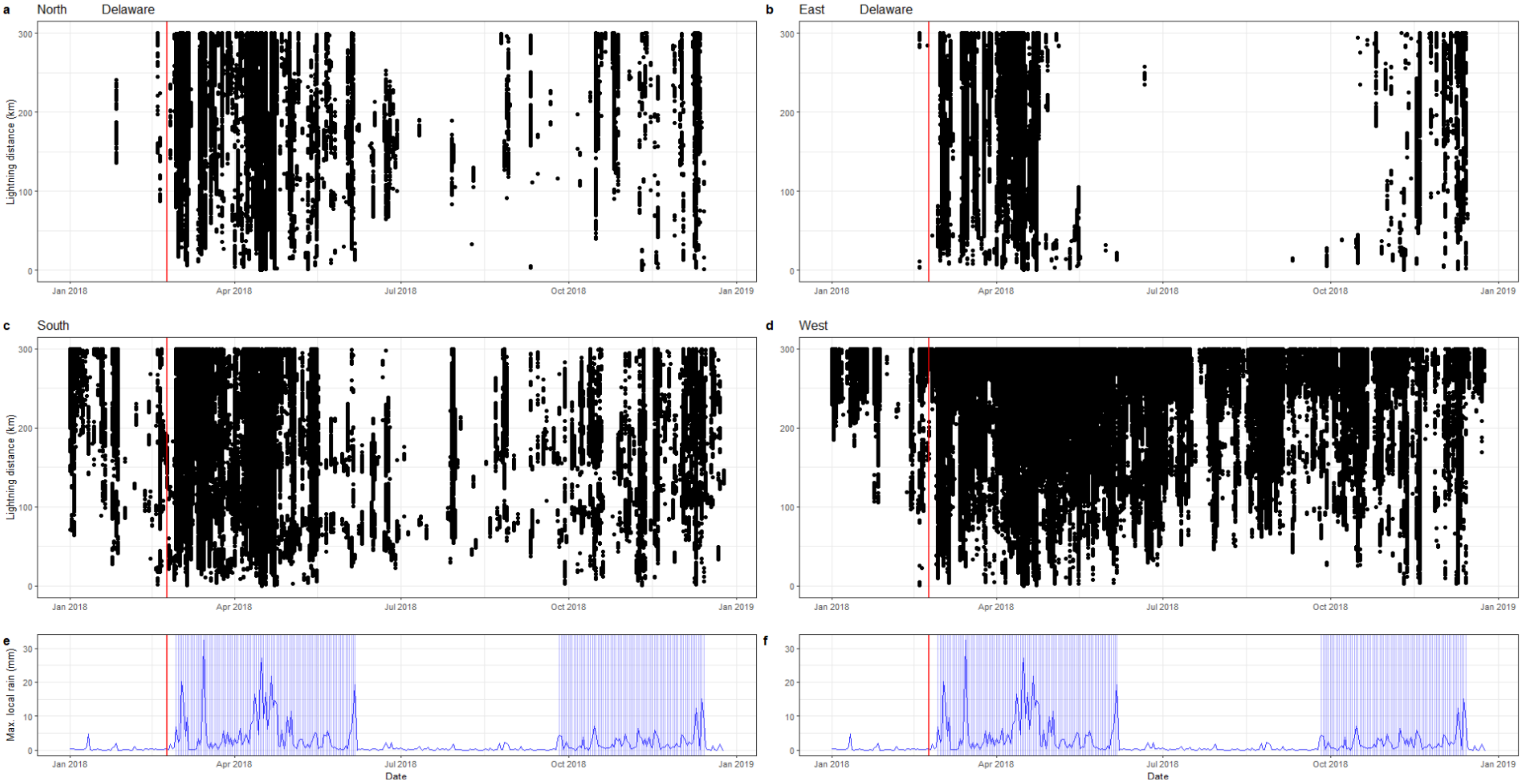

a
North
Delaware
b
East
Delaware
c
South
d
West
e
f
Lightning distance (km)
Max. local rain (mm)
Date
Jan 2018
Apr 2018
Jul 2018
Oct 2018
Jan 2019

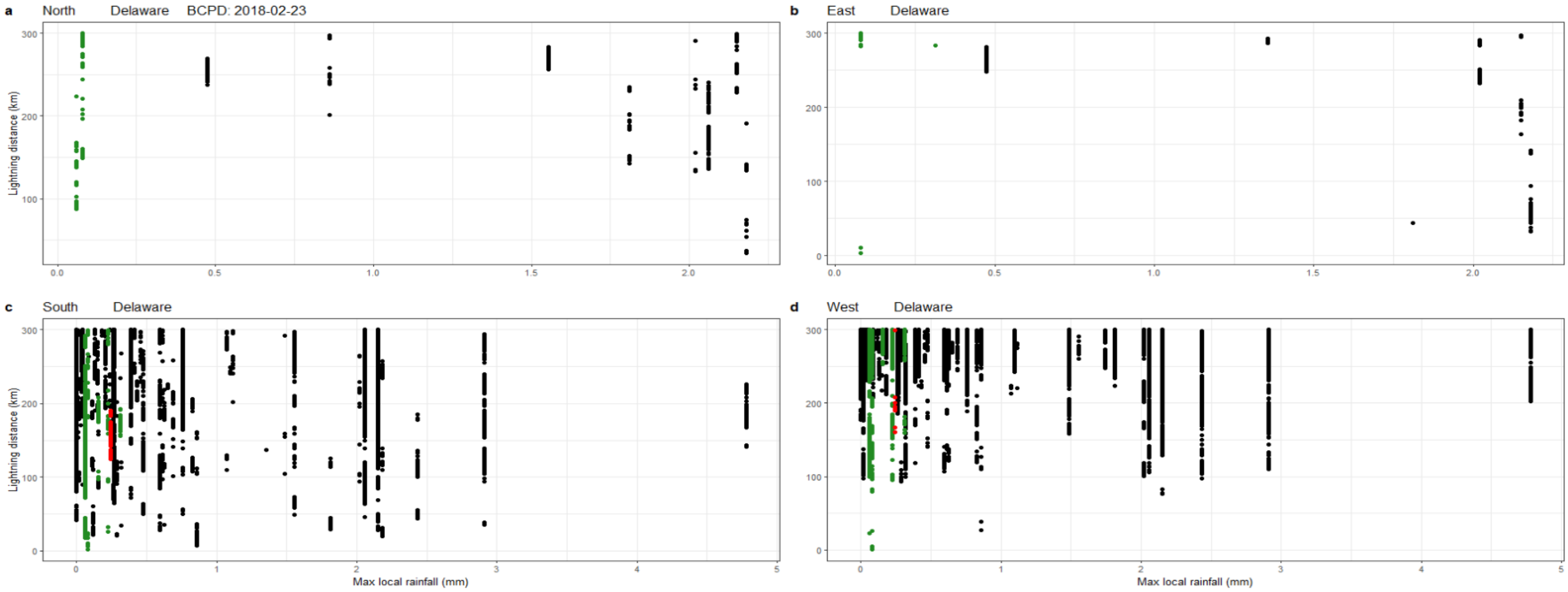

a
North
Delaware
BCPD: 2018-02-23
b
East
Delaware
c
South
Delaware
d
West
Delaware
Lightning distance (km)
Max local rainfall (mm)

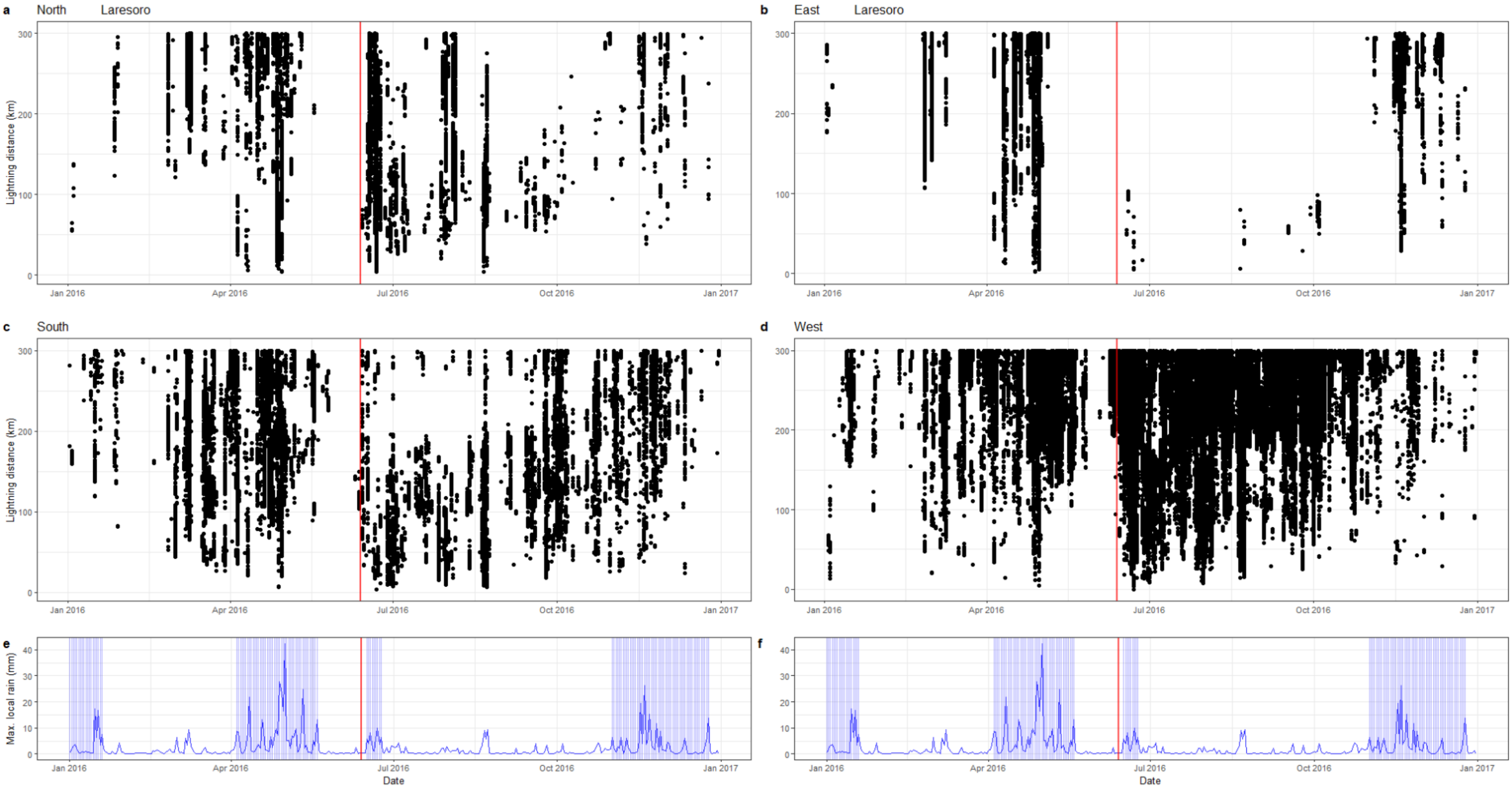

a North Laresoro
b East Laresoro
c South
d West
e
f
Lightning distance (km)
Max. local rain (mm)
Jan 2016
Apr 2016
Jul 2016
Oct 2016
Jan 2017
Date

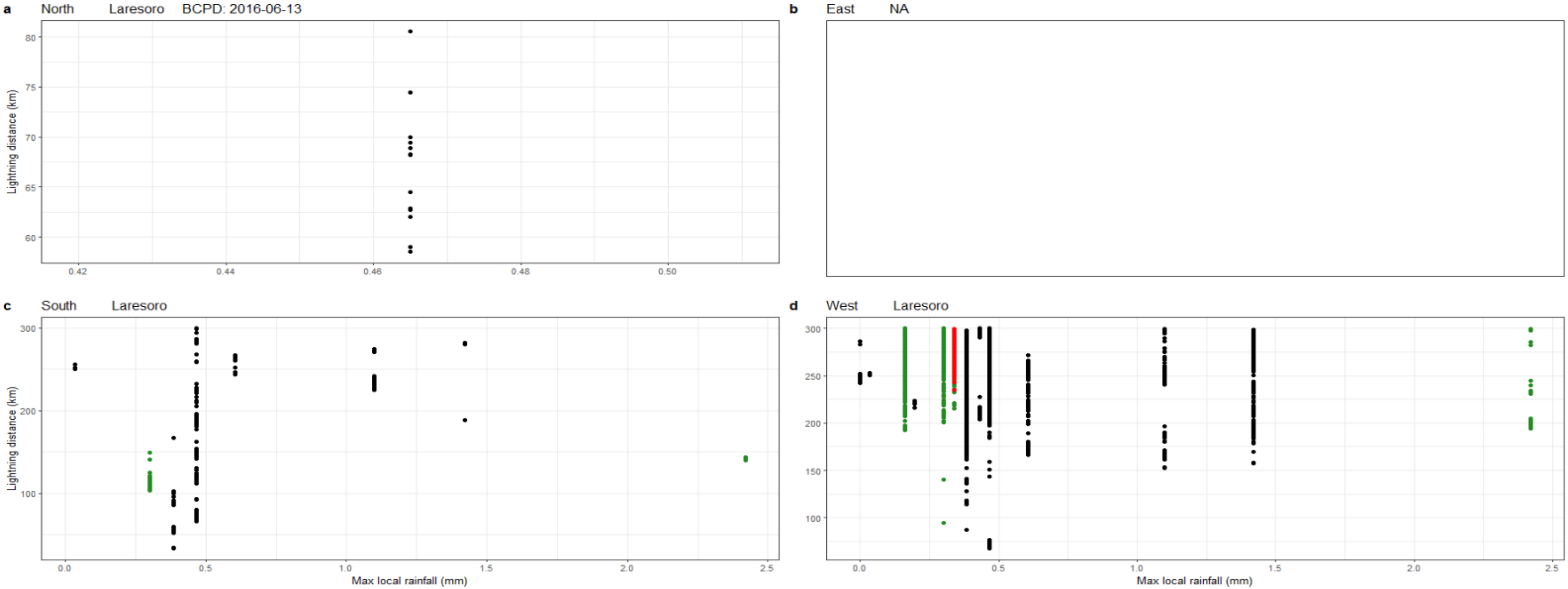

a
North
Laresoro
BCPD: 2016-06-13
Lightning distance (km)
80
75
70
65
60
0.42
0.44
0.46
0.48
0.50
b
East
NA
c
South
Laresoro
Lightning distance (km)
300
200
100
0.0
0.5
1.0
1.5
2.0
2.5
Max local rainfall (mm)
d
West
Laresoro
300
250
200
150
100
0.0
0.5
1.0
1.5
2.0
2.5
Max local rainfall (mm)

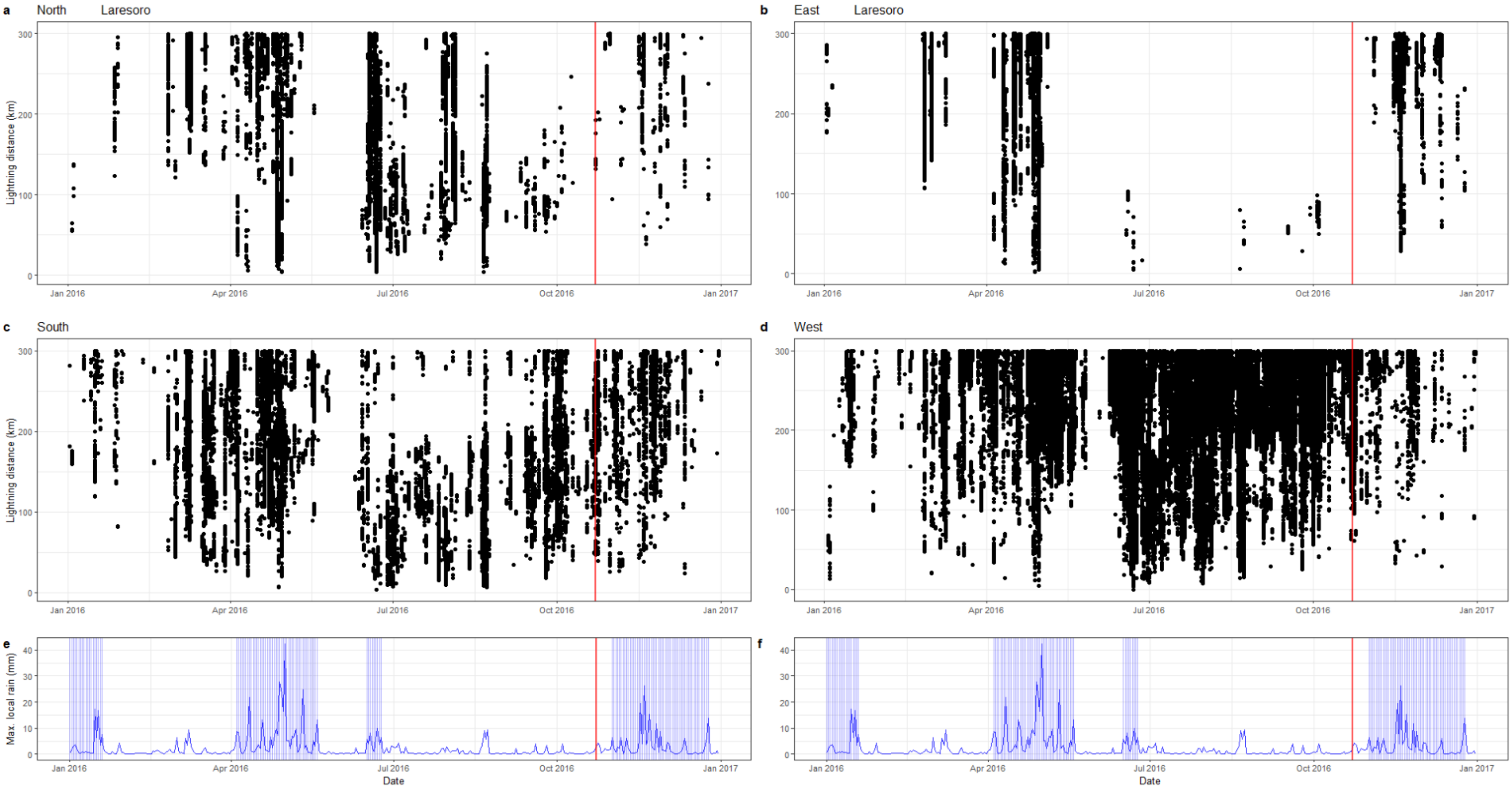

a North Laresoro
b East Laresoro
c South
d West
e
f
Lightning distance (km)
Max. local rain (mm)
Date
Jan 2016
Apr 2016
Jul 2016
Oct 2016
Jan 2017

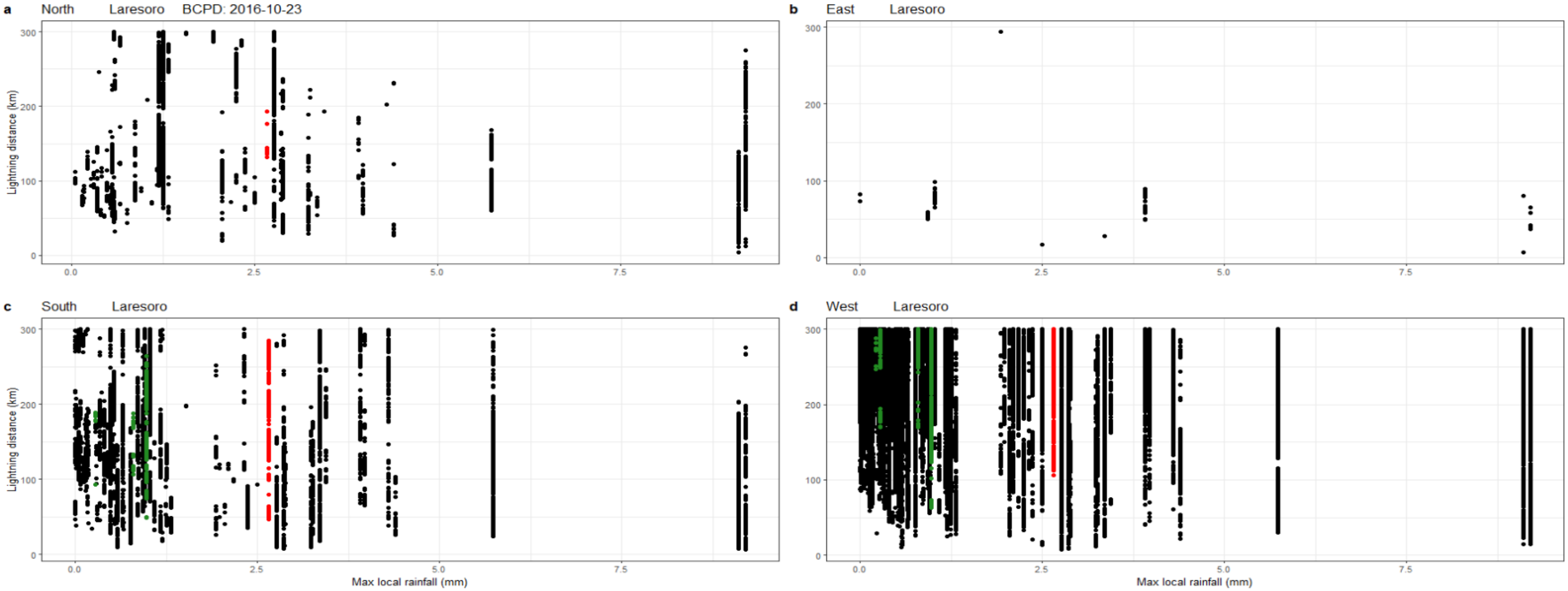
a
North
Laresoro
BCPD: 2016-10-23
b
East
Laresoro
c
South
Laresoro
d
West
Laresoro
Lightning distance (km)
Max local rainfall (mm)
0
100
200
300
0.0
2.5
5.0
7.5

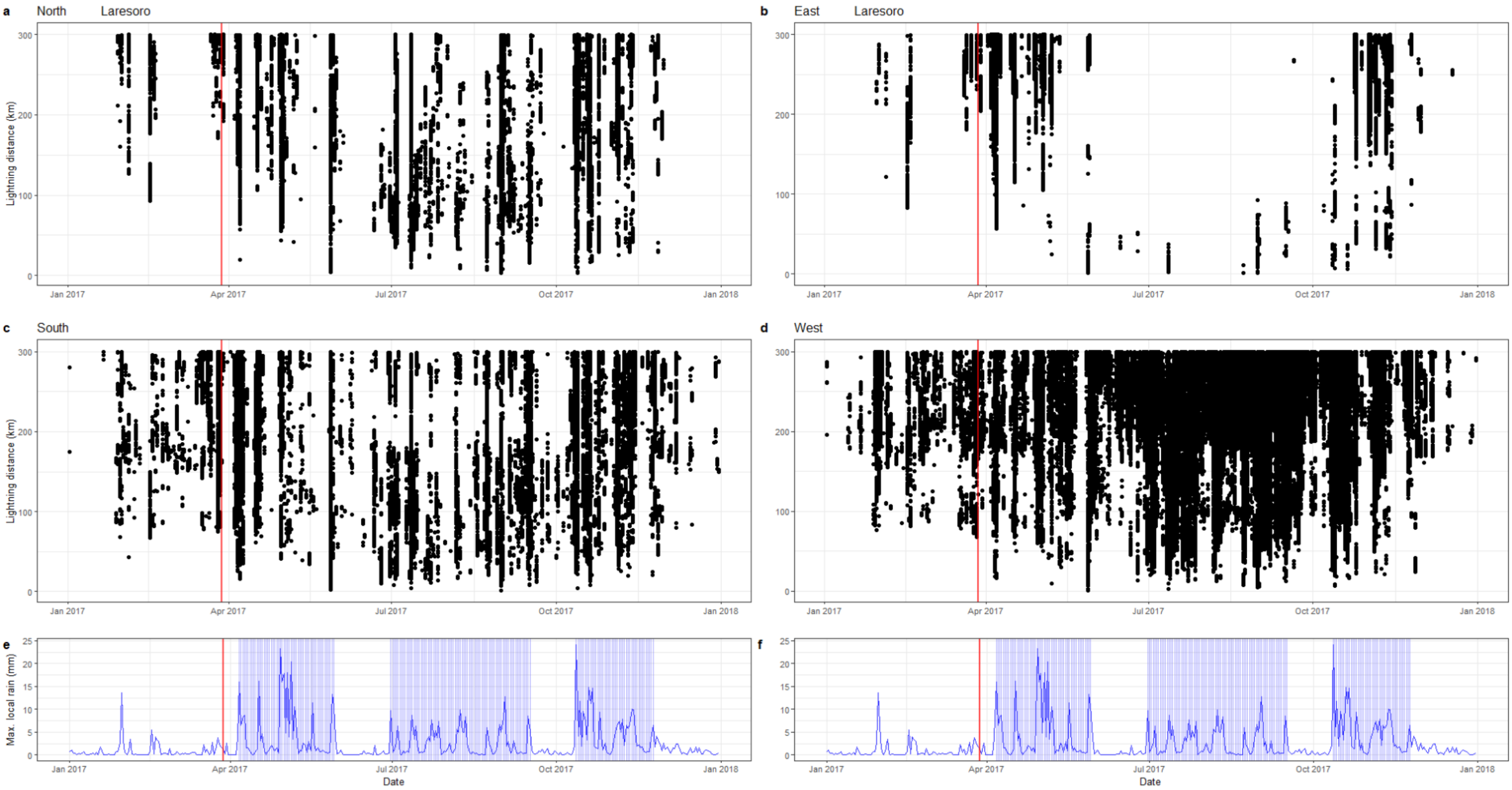

a North Laresoro
b East Laresoro
c South
d West
e
f
Lightning distance (km)
Max. local rain (mm)
Date
Jan 2017
Apr 2017
Jul 2017
Oct 2017
Jan 2018

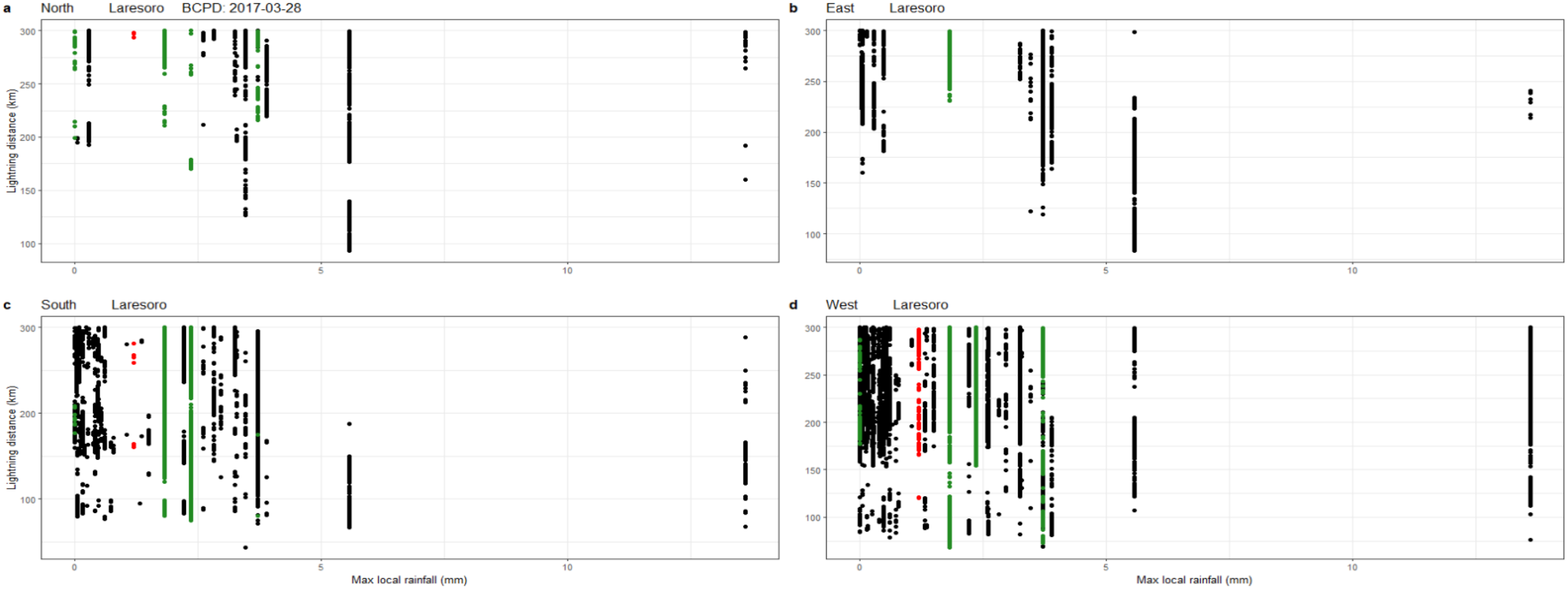

a
North
Laresoro
BCPD: 2017-03-28
b
East
Laresoro
c
South
Laresoro
d
West
Laresoro
Lightning distance (km)
Max local rainfall (mm)

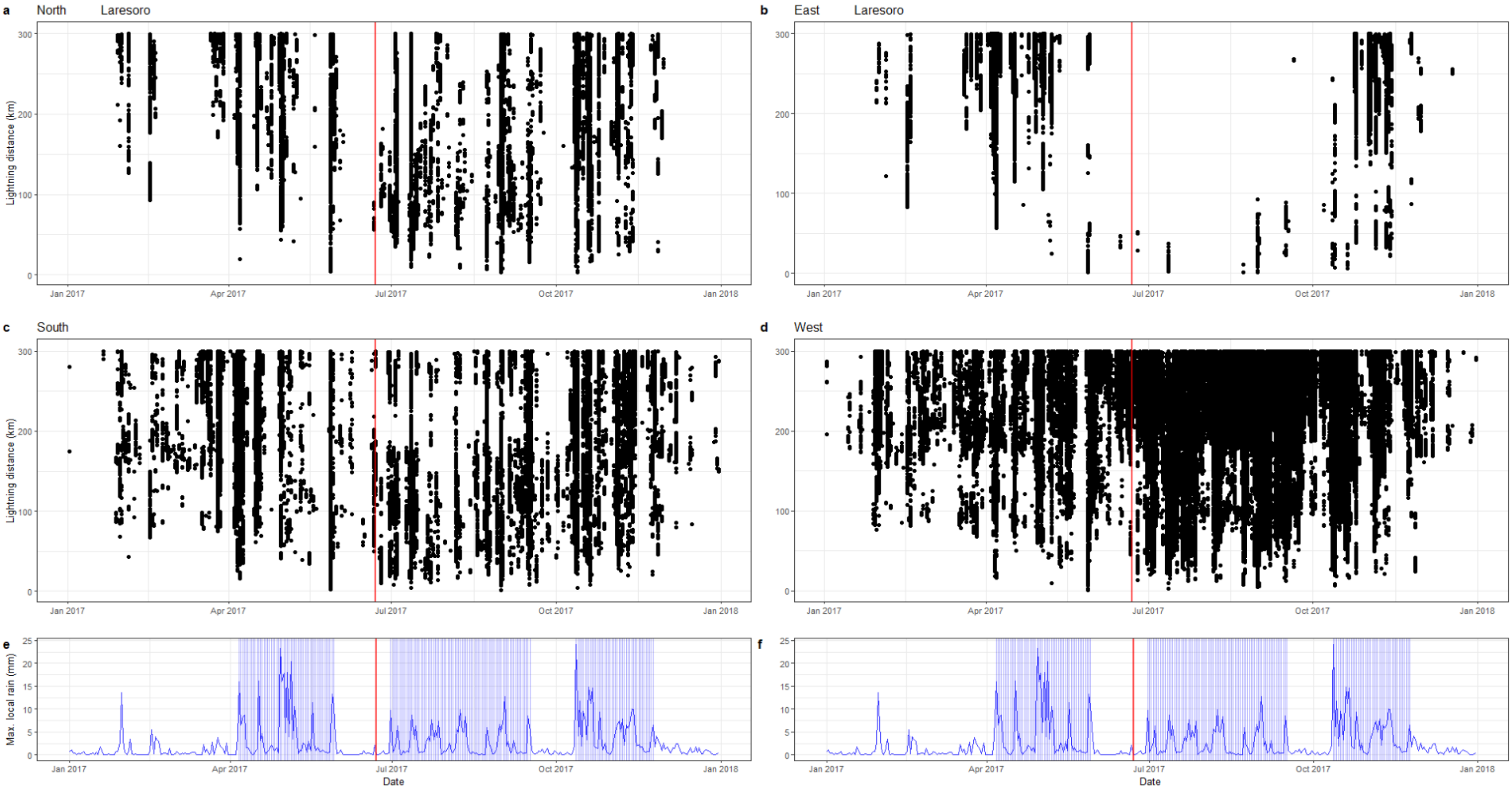

a North Laresoro
b East Laresoro
c South
d West
e
f
Lightning distance (km)
Max. local rain (mm)
Jan 2017
Apr 2017
Jul 2017
Oct 2017
Jan 2018
Date

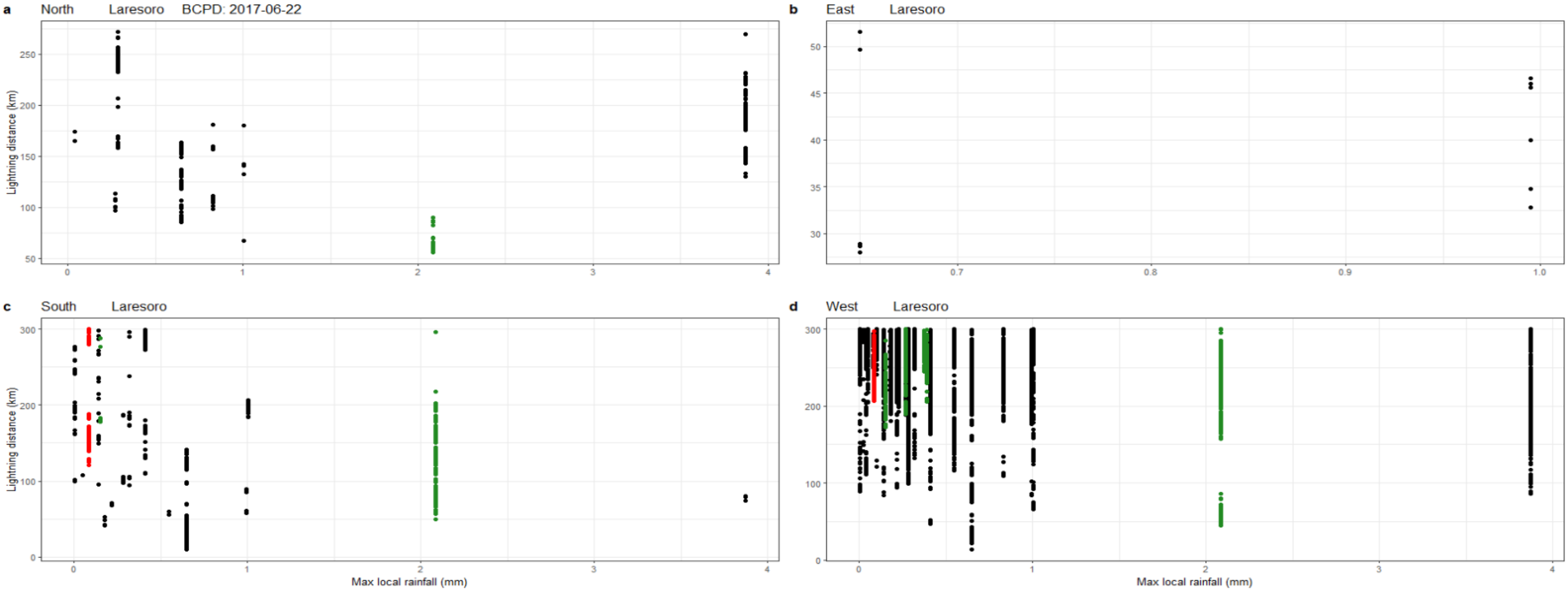

a North Laresoro BCPD: 2017-06-22
b East Laresoro
c South Laresoro
d West Laresoro
Lightning distance (km)
Max local rainfall (mm)

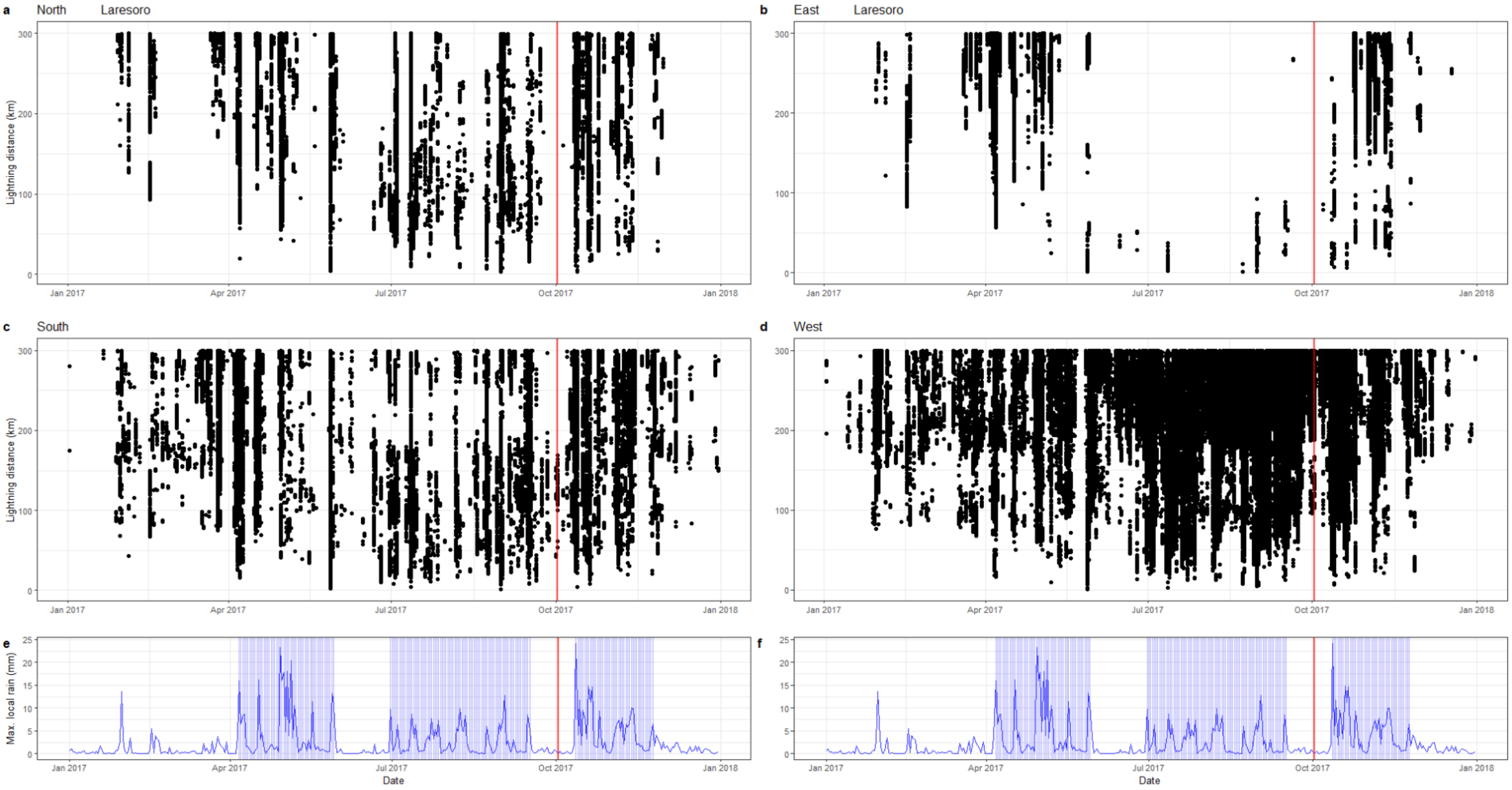

a North Laresoro
b East Laresoro
c South
d West
e
f
Lightning distance (km)
Max. local rain (mm)
0
100
200
300
0
5
10
15
20
25
Jan 2017
Apr 2017
Jul 2017
Oct 2017
Jan 2018
Date

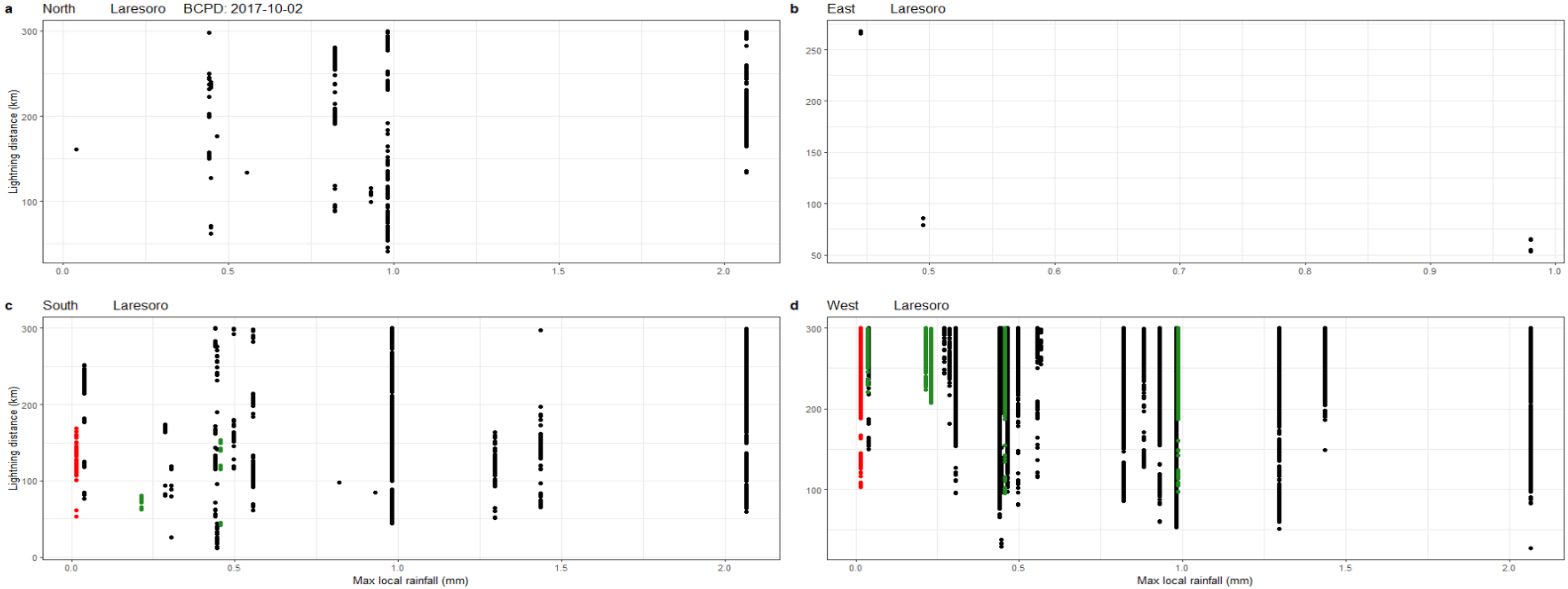
a
North
Laresoro
BCPD: 2017-10-02
b
East
Laresoro
c
South
Laresoro
d
West
Laresoro
Lightning distance (km)
Max local rainfall (mm)
300
200
100
250
150
50
0
0.0
0.5
1.0
1.5
2.0
0.6
0.7
0.8
0.9
