# Supplementary material for: Local rainfall is more likely than distant thunderstorms to affect movement behaviour in Northern Kenyan elephants
Source: PLoS One. 2024 Dec 23;19(12):e0307520. doi: 10.1371/journal.pone.0307520 (PMC11666045; doi:10.1371/journal.pone.0307520)
Supplement: S11 File — Detailed description of the theoretical behaviours based on the estimated behaviours of the BCPA states for Delaware and Laresoro. (PDF) [file pone.0307520.s011.pdf]

Laresoro

Foraging: For all years (2016-2018) intense foraging is detected from June/July - September/October. While in 2016 and 2017 these foraging stints are initiated by dry period intra-window BCPDs, in 2018 the intense foraging stint is initiated by a dry period extra-window BCPD and there is no wet period shortly thereafter. In 2016 another brief foraging stint is observed through April and May, which begins briefly after the start of the wet period and terminates in unison with that wet period (**Additional File 8**).

Directed travel: The stints of intense foraging were all preceded and terminated by a brief pulse of highly directed movement (extreme mean V_p peaks), captured within the longer stints of directed travel in January - April, June, and October 2016; June and October - December 2017; and January - July and October - December 2018. It should be noted that the extreme pulses following intense foraging stints were generally not detected as separate behavioural modes from directed foraging by the BCPA models because they were highly ephemeral (**Additional File 8**).

Directed foraging: January - March 2017 was marked by a slightly directional foraging strategy with a near-zero V_p mean but with a substantial increase in autocorrelation, suggesting some directionality (**Additional File 8**).

Active searching: October – December 2016, and April - May 2017 indicates a lot of random turns with little directionality and substantial distances covered as may be indicative of active searching (**Additional File 8**).

Delaware

Foraging: Foraging was observed May - June and November - December 2016, as well as April - May and November - December 2017, and was consistently initiated approximately 1 - 1.5 months following the onset of wet periods. The only exception is January – March 2018 where foraging was initiated in absence of a wet period (**Additional File 8**).

Directional travel: Directional travel was observed during June – July 2016, as well as January and June – October 2018. We additionally believe that directional travel occurred during January 2017. Although January 2017 is marked by a sub-zero V_p mean, upon closer inspection the low mean value is due to a deep sharp trough immediately at the end of January, despite mostly positive points like during January 2018. Notably, during stints of directional travel the V_p mean was substantially lower than observed for Laresoro; suggesting a lower travel speed and/or persistence in a given direction (**Additional File 8**).

Directed foraging: Directed foraging with a slightly elevated autocorrelation relative to regular foraging, was only observed in January – March and July – November 2016 (**Additional File 8**).

Active searching: All intra-window BCPDs by Delaware initiated active searching stints. However, it appears that Delaware partook in three variations of active searching. Firstly, the most common and relatively low intensity active random searching behaviour is observed in April – March 2017, as well as April – June and November – December 2018, with a relatively low variance (but higher than normal foraging), a near-zero V_p mean, and low autocorrelation. In 2017, the low intensity random searching behaviour is switched to a higher intensity random searching behaviour from March – April, where variance increases substantially whilst the mean and autocorrelation remain roughly equal – suggesting that the animal started covering greater distances and/or began taking more abrupt turns. Finally, one period of active searching did not appear to be random. May – October 2017 was marked by a strongly negative V_p mean, and high variance, indicating a behaviour with a

lot of turns in the backwards direction (i.e. turning back on itself). This may be indicative of an aggressive zig-zag style of active searching, instead of a random searching behaviour where turns in the forward and backward direction are equally common (**Additional File 8**).
